# Analysis of 1,000+ Type-Strain Genomes Substantially Improves Taxonomic Classification of **Alphaproteobacteria**

**DOI:** 10.3389/fmicb.2020.00468

**Published:** 2020-04-07

**Authors:** Anton Hördt, Marina García López, Jan P. Meier-Kolthoff, Marcel Schleuning, Lisa-Maria Weinhold, Brian J. Tindall, Sabine Gronow, Nikos C. Kyrpides, Tanja Woyke, Markus Göker

**Affiliations:** ^1^Department of Bioinformatics, Leibniz Institute DSMZ – German Collection of Microorganisms and Cell Cultures, Brunswick, Germany; ^2^Institute of Organic Chemistry and Biochemistry, Czech Academy of Sciences, Prague, Czechia; ^3^Department of Microorganisms, Leibniz Institute DSMZ – German Collection of Microorganisms and Cell Cultures, Brunswick, Germany; ^4^Department of Energy, Joint Genome Institute, Berkeley, CA, United States

**Keywords:** G+C content, genome size, Genome BLAST Distance Phylogeny, chemotaxonomy, morphology, phylogenetic systematics, phylogenomics

## Abstract

The class *Alphaproteobacteria* is comprised of a diverse assemblage of Gram-negative bacteria that includes organisms of varying morphologies, physiologies and habitat preferences many of which are of clinical and ecological importance. *Alphaproteobacteria* classification has proved to be difficult, not least when taxonomic decisions rested heavily on a limited number of phenotypic features and interpretation of poorly resolved 16S rRNA gene trees. Despite progress in recent years regarding the classification of bacteria assigned to the class, there remains a need to further clarify taxonomic relationships. Here, draft genome sequences of a collection of genomes of more than 1000 *Alphaproteobacteria* and outgroup type strains were used to infer phylogenetic trees from genome-scale data using the principles drawn from phylogenetic systematics. The majority of taxa were found to be monophyletic but several orders, families and genera, including taxa recognized as problematic long ago but also quite recent taxa, as well as a few species were shown to be in need of revision. According proposals are made for the recognition of new orders, families and genera, as well as the transfer of a variety of species to other genera and of a variety of genera to other families. In addition, emended descriptions are given for many species mainly involving information on DNA G+C content and (approximate) genome size, both of which are confirmed as valuable taxonomic markers. Similarly, analysis of the gene content was shown to provide valuable taxonomic insights in the class. Significant incongruities between 16S rRNA gene and whole genome trees were not found in the class. The incongruities that became obvious when comparing the results of the present study with existing classifications appeared to be caused mainly by insufficiently resolved 16S rRNA gene trees or incomplete taxon sampling. Another probable cause of misclassifications in the past is the partially low overall fit of phenotypic characters to the sequence-based tree. Even though a significant degree of phylogenetic conservation was detected in all characters investigated, the overall fit to the tree varied considerably.

## Introduction

The class *Alphaproteobacteria* is a diverse group of bacteria that is taxonomically assigned to the phylum *Proteobacteria* (Garrity et al., 2005a). At the time of writing the class comprises more than a dozen orders with validly published names. *Alphaproteobacteria* are cosmopolitan and colonize a wide range of habitats including soil, pelagic and benthic regions of the ocean, fresh water, and lichens. Frequently *Alphaproteobacteria* account for one of the most active and numerically dominant taxon of microbial communities (Brinkhoff et al., 2008; Bates et al., 2011; Schiaffino et al., 2016). The variety of habitats is illustrated by *Rhodobacteraceae* which is predominantly marine as for genera such as *Oceanicella* (Albuquerque et al., 2012) but also includes genera such as *Pannonibacter* (Borsodi et al., 2003; Biebl et al., 2007), which is found in lakes, and *Ketogulonigenium* (Urbance et al., 2001; Simon et al., 2017), found in soil.

Although the vast majority of *Alphaproteobacteria* are free-living, this class does include representatives associated with a broad range of hosts. *Rhizobium*, for example, establishes endosymbiotic nitrogen-fixing associations with roots of legumes (Pini et al., 2011). These bacteria are key players in the nitrogen turnover and have an important role in agriculture because they act as a natural fertilizer for plants (Fox et al., 2007) and for bioremediation and mineralization of industrial pollutants (Siddavattam et al., 2011). Other kinds of symbiosis are also established, such as the one between *Silicibacter* and marine phytoplankton (Belas et al., 2009). *Wolbachia* includes endosymbionts of arthropods (Hedges et al., 2008). Their host interactions are often complex and in some cases have evolved into a mutualistic rather than parasitic relationship (Hosokawa et al., 2010; Nikoh et al., 2014) giving their hosts resistance to viral infections (Teixeira et al., 2008). Other *Alphaproteobacteria*, such as *Bartonella* (Strong et al., 1915; Brenner et al., 1993; Birtles et al., 1995) and *Brucella* (Verger et al., 1985; Meyer and Shaw, 1920), are obligate intracellular parasites. Genera like *Rickettsia* can trigger serious diseases in plants, animals and humans (Fournier et al., 2000; Luis-Pantoja et al., 2015; Maina et al., 2016). *Alphaproteobacteria* also harbours opportunistic human pathogens such as *Roseomonas* (Rihs et al., 1993; Sánchez-Porro et al., 2009; Venkata Ramana et al., 2010).

*Alphaproteobacteria* are metabolically diverse, too. Most representatives of the class are chemoorganoheterotrophs but many others perform anoxygenic photosynthesis (Brinkmann et al., 2018), including the families *Rhodobacteraceae* or *Rhodospirillaceae*, the so-called purple non-sulfur bacteria (Imhoff et al., 1998). The phototrophic genera include *Porphyrobacter* (Fuerst et al., 1993; Coil et al., 2015), *Roseobacter* (Shiba, 1991; Martens et al., 2006), and *Rhodobacter* (Imhoff et al., 1984; Srinivas et al., 2007b; Wang et al., 2014). bacteriochlorophyll α and carotenoids are mostly present in phototrophic bacteria but can also be found in non-phototrophic bacteria like *Roseibium* (Zhong et al., 2014). While photoorganoheterotrophy is found in *Rhodovulum* (Hiraishi and Ueda, 1994a) and *Phaeospirillum* (Imhoff et al., 1998), chemolithoorganotrophy is present in *Elioraea* (Albuquerque et al., 2008) and facultative methylotrophy in *Methylarcula* (Doronina et al., 2000). *Magnetospirillum* (Schleifer et al., 1991), *Magnetococcus* (Bazylinski et al., 2013a) and *Magnetovibrio* (Bazylinski et al., 2013b) contain tiny chains of magnetite which support magnetotaxis (Schleifer et al., 1991). *Alphaproteobacteria* include obligate aerobic bacteria such as *Maribius* (Choi et al., 2007) as well as facultative aerobes, facultative anaerobes like *Pannonibacter* (Borsodi et al., 2003), and obligate anaerobes such as *Phaeobacterium* (Borsodi et al., 2003; Choi et al., 2007; Nupur et al., 2015). Yet the vast majority of *Alphaproteobacteria* are aerobes and to a lesser extent facultative anaerobes.

As for chemotaxonomy, the presence of sphingolipids is remarkable within *Alphaproteobacteria* since it appears to be restricted to *Sphingomonadales* (Kosako and Yabuuchi, 2005). Morphologically, *Alphaproteobacteria* are mostly found to be rod-, coccus- or ovoid-shaped. Yet some taxa deviate from this pattern, such as the spirilla-shaped *Rhodospirillaceae* (Pfennig and Trüper, 1971) including *Magnetospirillum* (Schleifer et al., 1991) and *Thalassospira* (López-López et al., 2002; Liu et al., 2007; Tsubouchi et al., 2014). *Caulobacter* (Henrici and Johnson, 1935; Abraham et al., 1999) and *Brevundimonas* (Segers et al., 1994; Abraham et al., 1999) of *Caulobacteraceae* (Henrici and Johnson, 1935), as well as *Litorimonas* (Jung et al., 2011; Nedashkovskaya et al., 2013), *Hellea* (Alain et al., 2008) and *Oceanibulbus* (Wagner-Döbler et al., 2004) of *Rhodobacteraceae* (Garrity et al., 2005b), are also unique as they form stalks. Many *Alphaproteobacteria* are motile by means of flagella, as exemplified by *Caulobacterales* (Henrici and Johnson, 1935) which mostly display flagella. Periplasmic flagella are present in some species, particularly in *Salinispira* (Ben Hania et al., 2015). Gliding motility has rarely been reported; examples are *Pacificimonas* (Liu K. et al., 2014) and *Acuticoccus* (Hou et al., 2015).

The class *Alphaproteobacteria* was proposed relatively recently (Garrity et al., 2005a) even though the first representatives of the group were isolated as early as 1898 (Beijerinck, 1898). As in other groups of bacteria the initial classification of *Alphaproteobacteria* into orders, families and genera was based on morphological and physiological characteristics, whereas advances in molecular systematics led to the view that taxonomic classification should be based on the integrated use of genotypic and phenotypic data (Wayne et al., 1987; Stackebrandt, 1992), an approach known as polyphasic taxonomy (Colwell, 1970; Vandamme et al., 1996; Gillis et al., 2005; Kämpfer and Glaeser, 2012). In particular, 16S rRNA gene sequences have been routinely applied to infer phylogenetic trees or in conjunction with simpler approaches such as pairwise distance or similarities (Meier-Kolthoff et al., 2013b; Kim and Chun, 2014; Yarza and Munoz, 2014). The technique named Multilocus Sequence Analysis or MLSA (Glaeser and Kämpfer, 2015) has widely been used to resolve the phylogeny of different taxonomic groups of *Alphaproteobacteria* like *Ensifer* (Martens et al., 2008) and *Bradyrhizobium* (Rivas et al., 2009). However, trees based on a few thousand nucleotides such as those based on a single phylogenetic marker (1400–1500 nucleotides in the case of the 16S rRNA gene), or even a few concatenated housekeeping genes as in the case of MLSA tend to have branches with low bootstrap values (Klenk and Göker, 2010).

Better resolved phylogenies based on the hundreds of housekeeping genes or even the core-genome has been used to elucidate the phylogenetic relationships among selected groups of closely related taxa (Williams et al., 2007; Wirth and Whitman, 2018). Given the rapid and ongoing progress in sequencing technologies (Mavromatis et al., 2012), classifications based on whole genome sequences and associated bioinformatic tools can be based on millions of characters. This provides a step change in reliability, as evidenced by high average bootstrap support in phylogenomic trees (Breider et al., 2014; Meier-Kolthoff et al., 2014a), even though the ordinary bootstrap is not necessarily the most reliable approach when dealing with supermatrices potentially comprised of genes with distinct histories (Siddall, 2010; Simon et al., 2017). Reclassifications at all levels of the taxonomic hierarchy can result from such approaches (Hahnke et al., 2016; Nouioui et al., 2018). It was also shown that DNA G+C composition values directly calculated from genome sequences have a significantly better fit to the phylogeny than the experimentally determined ones cited in many species descriptions (Hahnke et al., 2016). This is in line with the observation that within-species variation is at most 1% when G+C content is calculated from genome sequences (Meier-Kolthoff et al., 2014c) and that claims in the literature that the variation in G+C content within bacterial species is at most 3 mol% (Mesbah et al., 1989) or even 5% (Rosselló-Mora and Amann, 2001) can be attributed to experimental error in traditional methods (Mesbah et al., 1989; Moreira et al., 2011). Recent studies based on complete genomes also confirm that the distribution of the G+C content is phylogenetically conserved. While this also holds to a somewhat lesser degree for genome size (Nouioui et al., 2018), phylogenetic inertia of these features has not yet been measured for *Alphaproteobacteria*. Likewise, it is as yet unknown to which degree gene-content phylogenies (Huson and Steel, 2004) are in concordance with standard genome-scale phylogenies even though both approaches showed high agreement in subgroups of *Alphaproteobacteria* (Breider et al., 2014) and because the gene content is of relevance as it conveys phenotypic features (Zhu et al., 2015).

The aim of the present study is an improved phylogenetic framework for the classification of *Alphaproteobacteria*. Genome-scale phylogenetic trees were inferred for genome-sequenced type strains and augmented by analyses of a comprehensive collection of type-strain 16S rRNA gene sequences to address the following questions: (i) to what extent are phylogenies calculated from whole genome sequences still in conflict with the current classification of *Alphaproteobacteria* and with their 16S rRNA gene phylogenies? (ii) Which taxa need to be revised because they are evidently non-monophyletic? (iii) Which taxon descriptions should be modified because of inaccurate or missing G+C values? and (iv) How do G+C content, genome size, genomic gene content and routinely recorded phenotypic features of *Alphaproteobacteria* relate to their phylogeny and to which degree can they serve as a taxonomic marker?

## Materials and Methods

The approach to taxon sampling and analysis was in almost all respects the same as previously described (Hahnke et al., 2016; Nouioui et al., 2018). A total of 1104 annotated type-strain genome sequences (Supplementary Table S1) for *Alphaproteobacteria* (ingroup) and *Spirochaetes* (outgroup) were collected. While some originated from GenBank the majority was obtained *de novo* in the course of the KMG projects phase II (Mukherjee et al., 2017) and phase IV and deposited in the Integrated Microbial Genomes platform (Chen et al., 2019) and in the Type-Strain Genome Server database (Meier-Kolthoff and Göker, 2019). Among *Alphaproteobacteria* KMG-II mainly targeted *Rhodobacteraceae* but also representatives of other families. All newly generated KMG sequences underwent standard quality control at DSMZ and JGI documented on the respective web pages and had < 100 contigs. All accepted genome sequences had < 500 contigs and matched the 16S rRNA gene reference database described below. Structural annotation at JGI and DSMZ was done using Prodigal v. 2.6.2 (Hyatt et al., 2010). The features of all genome sequences that entered these analyses are provided in Supplementary Table S1. These annotated genome sequences were processed further as in our previous study using the high-throughput version of the Genome BLAST Distance Phylogeny (GBDP) approach in conjunction with BLAST+ v2.2.30 in blastp mode (Auch et al., 2006; Camacho et al., 2009; Meier-Kolthoff et al., 2014a) and FastME version 2.1.6.1 using the improved neighbor-joining algorithm BioNJ for obtaining starting trees followed by branch swapping under the balanced minimum evolution criterion (Desper and Gascuel, 2004) using the subtree-pruning-and-regrafting algorithm (Desper and Gascuel, 2006; Lefort et al., 2015). One hundred pseudo-bootstrap replicates (Meier-Kolthoff et al., 2013a, 2014a) were used to obtain branch-support values for these genome-scale phylogenies.

Trees were visualized using Interactive Tree Of Life (Letunic and Bork, 2019) in conjunction with the script deposited at https://github.com/mgoeker/table2itol. Outgroup-based rooting was compared with rooting using least-squares dating as implemented in LSD version 0.2 (To et al., 2016) after removing the outgroup taxa and inferring an accordingly reduced tree with FastME. Species and subspecies boundaries were investigated using digital DNA:DNA hybridization (dDDH) as implemented in the Genome-To-Genome Distance Calculator (GGDC) version 2.1 (Meier-Kolthoff et al., 2013a) and in TYGS, the Type (Strain) Genome Server (Meier-Kolthoff and Göker, 2019).

In addition to GBDP formula *d*_5_, which explores sequence (dis-)similarity and is the recommended one for phylogenetic inference (Auch et al., 2006; Meier-Kolthoff et al., 2014a) we here used formula *d*_3_, which compares the gene content of the investigated genomes after correcting for reduction in genome size (Henz et al., 2005). While this analysis was also done using the GBDP software, for consistency with previous work we will refer to the *d*_5_ phylogeny as GBDP tree and to the *d*_3_ tree as gene-content analysis. There are various reasons why a gene-content phylogeny may fail to recover the true tree, as detailed below, hence the gene-content analysis is not intended to lend phylogenetic support. However, it may nevertheless be of taxonomic interest whether or not a certain branch is supported by gene-content data, particularly since the gene content conveys metabolic capabilities (Zhu et al., 2015) and yield independent evidence for conclusions from standard genome-scale phylogenies (Breider et al., 2014).

Full-length 16S rRNA gene sequences were extracted from the genomes using RNAmmer version 1.2 (Lagesen et al., 2007) and compared with the 16S rRNA gene reference database using BLAST and phylogenetic trees to verify the taxonomic affiliation of genomes. Non-matching genome sequences were discarded from further analyses. A comprehensive sequence alignment was generated with MAFFT version 7.271 with the “localpair” option (Katoh et al., 2005) using either the sequences extracted from the genome sequences or the previously published 16S rRNA gene sequences, depending on the length and number of ambiguous bases. Trees were inferred from the alignment with RAxML (Stamatakis, 2014) under the maximum-likelihood (ML) criterion and with TNT (Goloboff et al., 2008) under the maximum-parsimony (MP). In addition to unconstrained, comprehensive 16S rRNA gene trees (UCT), constrained comprehensive trees (CCT) were inferred with ML and MP using the bipartitions of the GBDP tree with ≥95% support as backbone constraint, as previously described (Hahnke et al., 2016; Nouioui et al., 2018).

Taxa were analyzed to determine whether they were monophyletic, paraphyletic or polyphyletic (Farris, 1974; Wood, 1994) Taxa non-monophyletic according to the GBDP tree were tested for evidence for their monophyly in the UCT and the 16S rRNA gene trees, if any, in the original publication. In the case of a significant conflict (i.e., high support values for contradicting bipartitions) between trees or low support in the GBDP tree, additional phylogenomic analyses of selected taxa were conducted. To this end, protein sequences of those taxa with the reciprocal best hits from GBDP/BLAST were clustered with MCL (Markov Chain Clustering) version 14-137 (Enright et al., 2002) under default settings and an e-value filter of 10^–5^ in analogy to OrthoMCL (Li et al., 2003). The resulting sets of orthologous proteins were aligned with MAFFT and concatenated to form a supermatrix after discarding the few clusters that still contained more than a single protein for at least one genome. Comprehensive supermatrices were compiled from all the orthologs that occurred in at least four genomes, whereas core-genome supermatrices were constructed for the orthologs that occurred in all of the genomes. Supermatrices were analyzed with TNT, and with RAxML under the “PROTCATLGF” model, in conjunction with 100 partition bootstrap replicates (Siddall, 2010; Simon et al., 2017)

Additionally, selected phenotypic features relevant for the taxonomic classification of *Alphaproteobacteria* were as comprehensively as possible collected from the taxonomic literature: motility by flagella, absence or presence of carotenoids, absence or presence of bacteriochlorophyll α, absence or presence of sphingolipids, average number of isoprene residues of the major ubiquinones, and relationship to oxygen. To avoid circular reasoning, missing features of a species were only inferred from features of its genus when species and genus were described in the same publication or when the species description had explicitly been declared as adding to the features of the genus. For the binary chemotaxonomic characters an alternative coding was also investigated that treated all missing values as indicating absence. Ubiquinone percentages would be more informative than just statements about being “major” but mostly only the latter are provided in the literature. Oxygen conditions were coded as ordered multi-state character: (1) strictly anaerobic; (2) facultatively aerobic, facultatively anaerobic, or microaerophilic; (3) strictly aerobic. Among all nine coding options tested, this yielded the highest fit to the tree (Supplementary Table S1) but the differences between the coding options were not pronounced. Phylogenetic conservation of selected phenotypic and genomic characters with respect to the GBDP tree (reduced to represent each set of equivalent strains by only a single genome) was evaluated using a tip-permutation test in conjunction with the calculation of maximum-parsimony scores with TNT as previously described (Simon et al., 2017; Carro et al., 2018) and 10,000 permutations. TNT input files were generated with opm (Vaas et al., 2013). The proportion of times the score of a permuted tree was at least as low as the score of the original tree yielded the *p*-value. Maximum-parsimony retention indices (Farris, 1989; Wiley and Lieberman, 2011) were calculated to further differentiate between the fit of each character to the tree.

Taxa that were unambiguously non-monophyletic according to the genome-scale analyses were screened for published evidence of their monophyly. The published evidence was judged as inconclusive when based on unsupported branches in phylogenetic trees, based on probably homoplastic characters or on probable plesiomorphic character states. Plesiomorphies might well be “diagnostic” but just for paraphyletic groups (Hennig, 1965; Wiley and Lieberman, 2011; Montero-Calasanz et al., 2017) hence “diagnostic” features alone are insufficient in phylogenetic systematics.

For fixing the obviously non-monophyletic taxa taxonomic consequences were proposed if new taxon delineations could be determined that were sufficiently supported by the CCT. In these cases, the uncertain phylogenetic placement of taxa whose genome sequences were not available at the time of writing would not affect the new proposals. Where necessary taxa were tentatively place in newly delineated groups.

## Results

The presentation of the results is organized as follows. After a brief overview on the figures and tables the outcome of the tests for the phylogenetic conservation are illustrated. Next, the phylogenetic results for the outgroup taxa are described and put in the context of their current taxonomic classification. Finally, the hierarchical classification of the class *Alphaproteobacteria* itself, arranged according to the orders in which it is currently subdivided and then according to the taxonomic categories, is compared to the phylogenomic trees. These sections motivate the need for a variety of reclassifications, whereas the actual taxonomic consequences are listed at the end of the section “Discussion.” Finally, the outcome of the tests for the phylogenetic conservation are illustrated.

**FIGURE 1:**
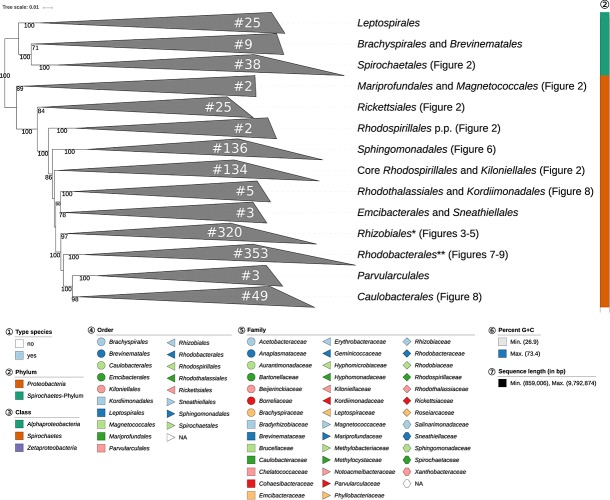
Overview of the phylogenomic tree inferred with FastME from GBDP distances calculated from whole proteomes. The numbers above branches are GBDP pseudo-bootstrap support values from 100 replications. Collapsed clades are displayed as triangles whose side lengths are proportional to the branch-length distances to least and most distant leave, respectively. The total number (#) of leaves per collapsed clade is shown within the triangles. The legend indicates the symbols and colors used in all subsequent figures, which show details of all clades of interest. In Figure 1 itself only the phylum is annotated, while Figures 2–9 show specific sections of the same tree in greater detail with the same underlying topology yet some differences in clade ordering. Clades not referred to in the text are not shown in detail in the subsequent figures but are displayed in Supplementary File S2. *phylogenetically including some taxa placed in *Rhodobacterales*, ** phylogenetically including some taxa placed in *Rhizobiales*.

**FIGURE 2:**
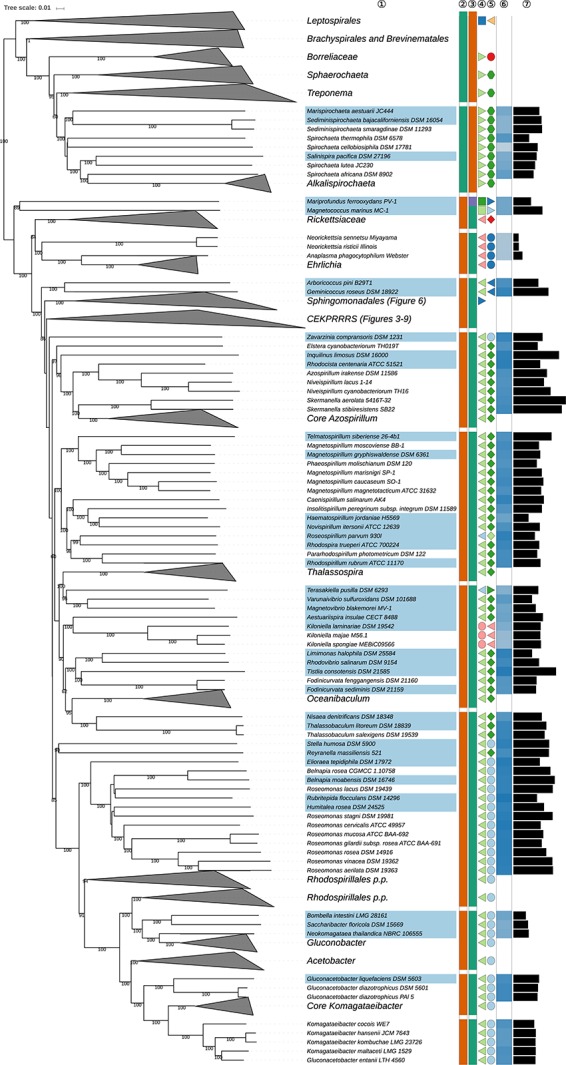
First part of the GBDP tree shown in Figure 1, focussing on the phylum *Spirochaetes* as well as *Rhodospirillales, Rickettsiales* and *Kiloniellales* within the class *Alphaproteobacteria.* The clade labeled CEKPRRRS comprises the orders *Caulobacterales, Emcibacterales, Kordiimonadales, Parvularculales, Rhizobiales* (*Hyphomicrobiales*), *Rhodobacterales, Rhodothalassiales*, and *Sneathiellales*, details for which are shown in the subsequent figures. Tip labels with light-blue background indicate type species of genera, colors and symbols to the right of the tips indicate, from left to right, phylum, class, order, and family; for details and abbreviations see Figure 1. The blue color gradient indicates the G+C content as calculated from the genome sequences, followed by black bars indicating the (approximate) genome size in base pairs.

**FIGURE 3:**
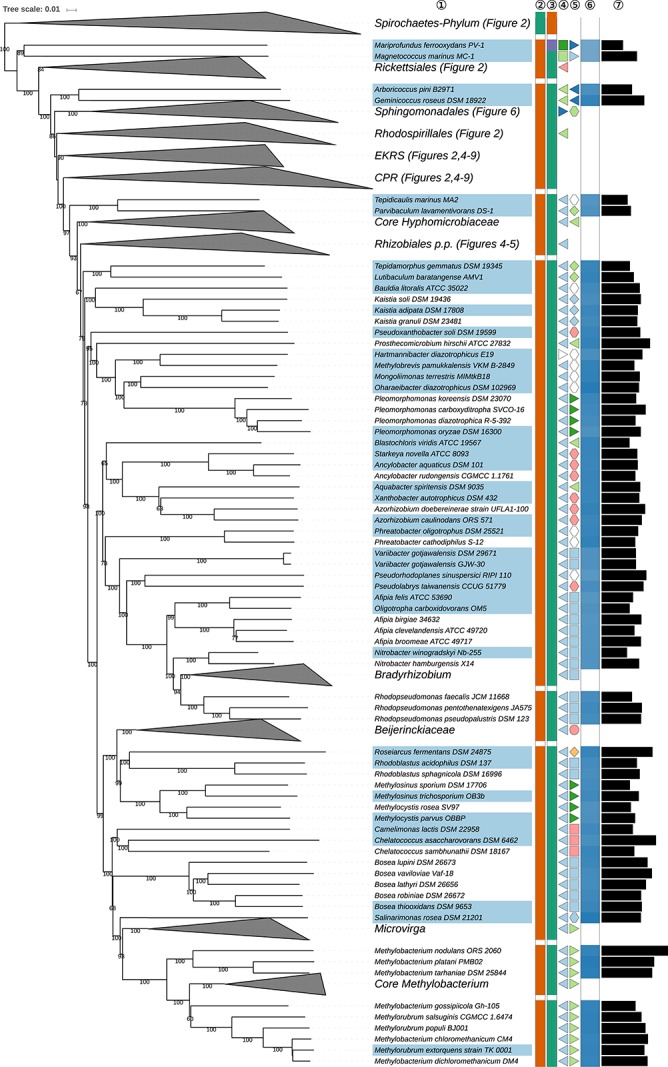
Second part of the GBDP tree shown in Figure 1, focussing on parts of *Rhizobiales* (*Hyphomicrobiales*). The clade labeled EKRS comprises the orders *Emcibacterales, Kordiimonadales, Rhodothalassiales*, and *Sneathiellales*, whereas CPR denotes the clade including the orders *Caulobacterales*, *Parvularculales*, and *Rhodobacterales*, details for which are shown in the subsequent figures. Tip labels with light-blue background indicate type species of genera, colors and symbols to the right of the tips indicate, from left to right, phylum, class, order and family; for details and abbreviations see Figure 1. The blue color gradient indicates the G+C content as calculated from the genome sequences, followed by black bars indicating the (approximate) genome size in base pairs.

**FIGURE 4:**
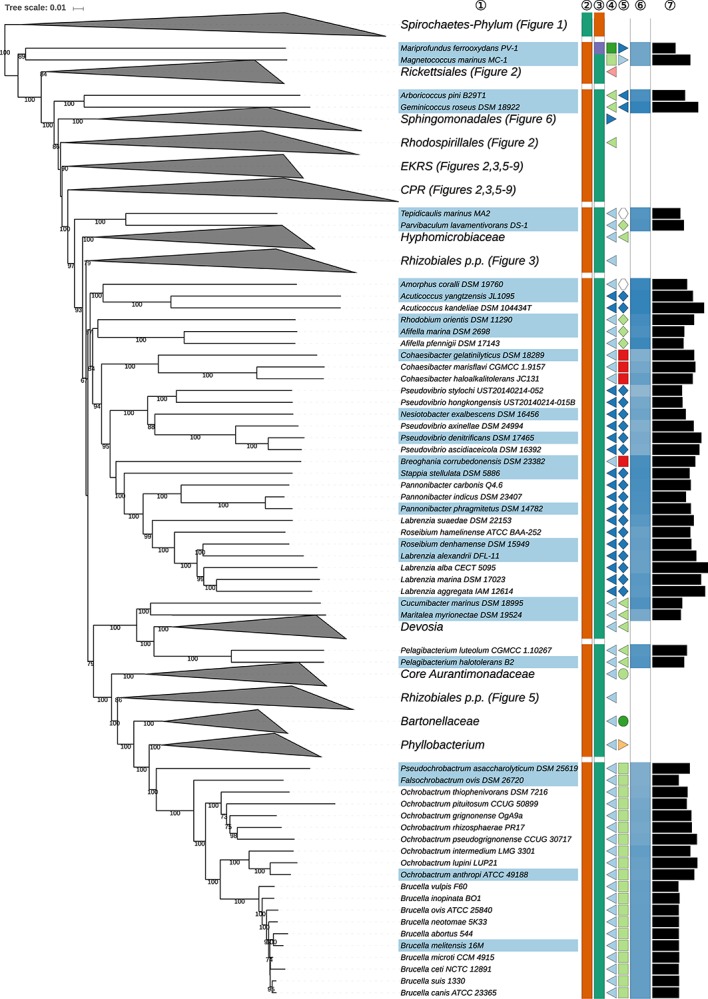
Third part of the GBDP tree shown in Figure 1, focussing on parts of *Rhizobiales* (*Hyphomicrobiales*). The clade labeled EKRS comprises the orders *Emcibacterales, Kordiimonadales, Rhodothalassiales* and *Sneathiellales*, whereas CPR denotes the clade including the orders *Caulobacterales*, *Parvularculales*, and *Rhodobacterales*, details for which are shown in the subsequent figures. Tip labels with light-blue background indicate type species of genera, colors and symbols to the right of the tips indicate, from left to right, phylum, class, order and family; for details and abbreviations see Figure 1. The blue color gradient indicates the G+C content as calculated from the genome sequences, followed by black bars indicating the (approximate) genome size in base pairs.

**FIGURE 5:**
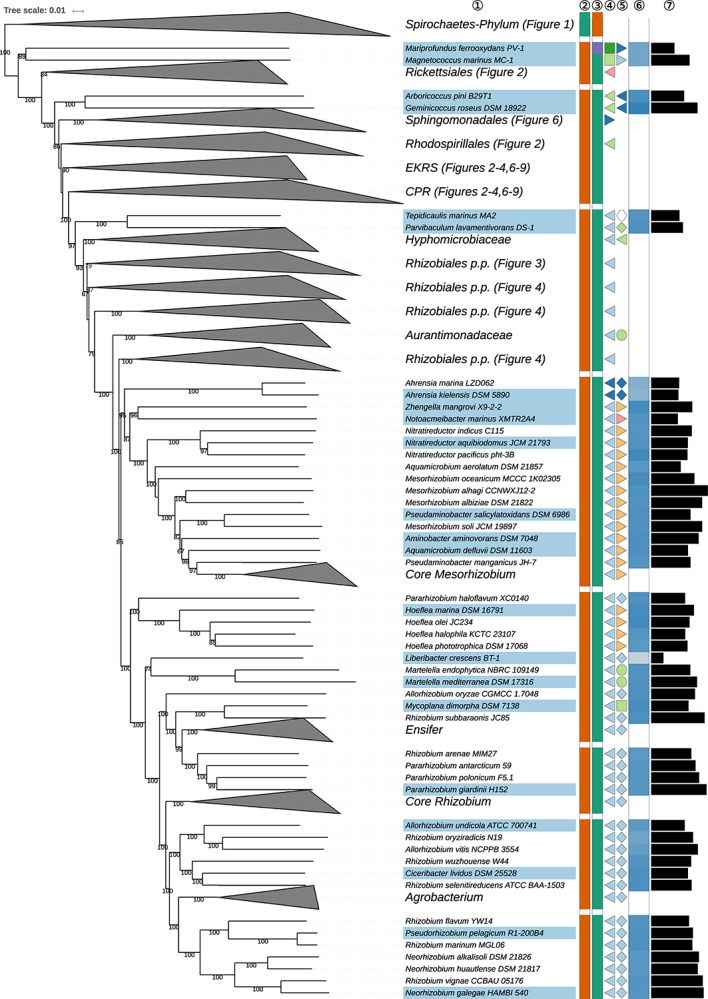
Fourth part of the GBDP tree shown in Figure 1, focussing on parts of *Rhizobiales* (*Hyphomicrobiales*). The clade labeled EKRS comprises the orders *Emcibacterales, Kordiimonadales, Rhodothalassiales* and *Sneathiellales*, whereas CPR denotes the clade including the orders *Caulobacterales*, *Parvularculales*, and *Rhodobacterales*, details for which are shown in the subsequent figures. Tip labels with light-blue background indicate type species of genera, colors and symbols to the right of the tips indicate, from left to right, phylum, class, order, and family; for details and abbreviations see Figure 1. The blue color gradient indicates the G+C content as calculated from the genome sequences, followed by black bars indicating the (approximate) genome size in base pairs.

**FIGURE 6:**
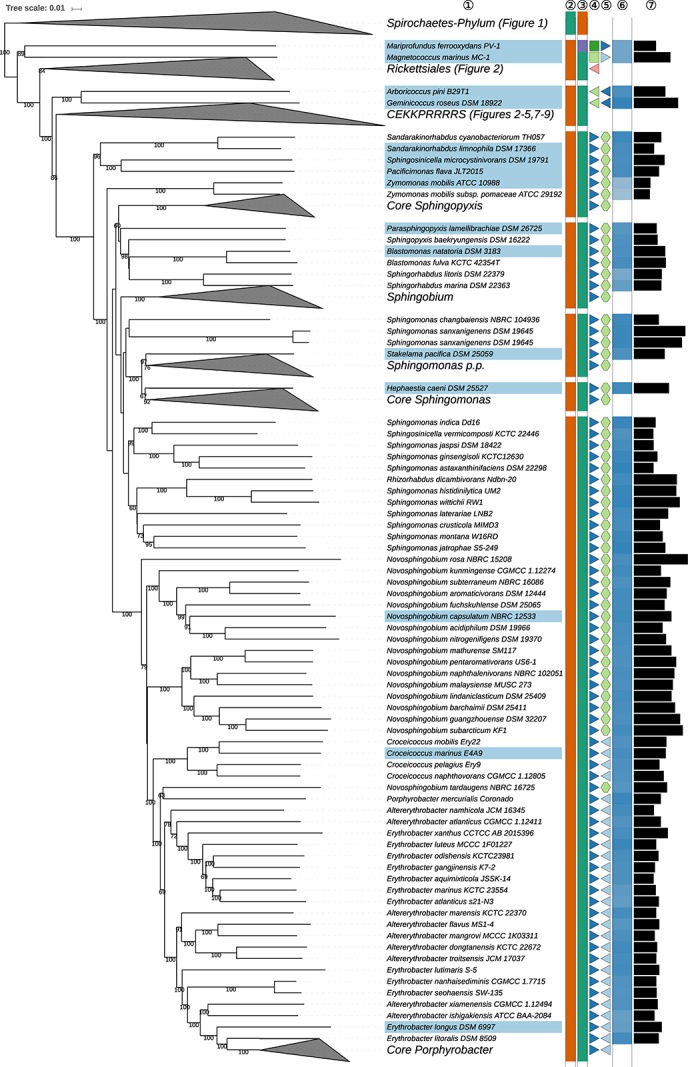
Fifth part of the GBDP tree shown in Figure 1, focussing on *Sphingomonadales*. The clade labeled CEKKPRRRRS comprises the orders *Caulobacterales, Emcibacterales, Kiloniellales, Kordiimonadales, Parvularculales, Rhizobiales* (*Hyphomicrobiales*), *Rhodobacterales, Rhodospirillales, Rhodothalassiales*, and *Sneathiellales*, details for which are shown in other figures. Tip labels with light-blue background indicate type species of genera, colors and symbols to the right of the tips indicate, from left to right, phylum, class, order, and family; for details and abbreviations see Figure 1. The blue color gradient indicates the G+C content as calculated from the genome sequences, followed by black bars indicating the (approximate) genome size in base pairs. *Sphingomonas sanxanigenens* DSM 19645 is represented by two genome sequences (SAMN02641489, above; SAMN02745820, below).

**FIGURE 7:**
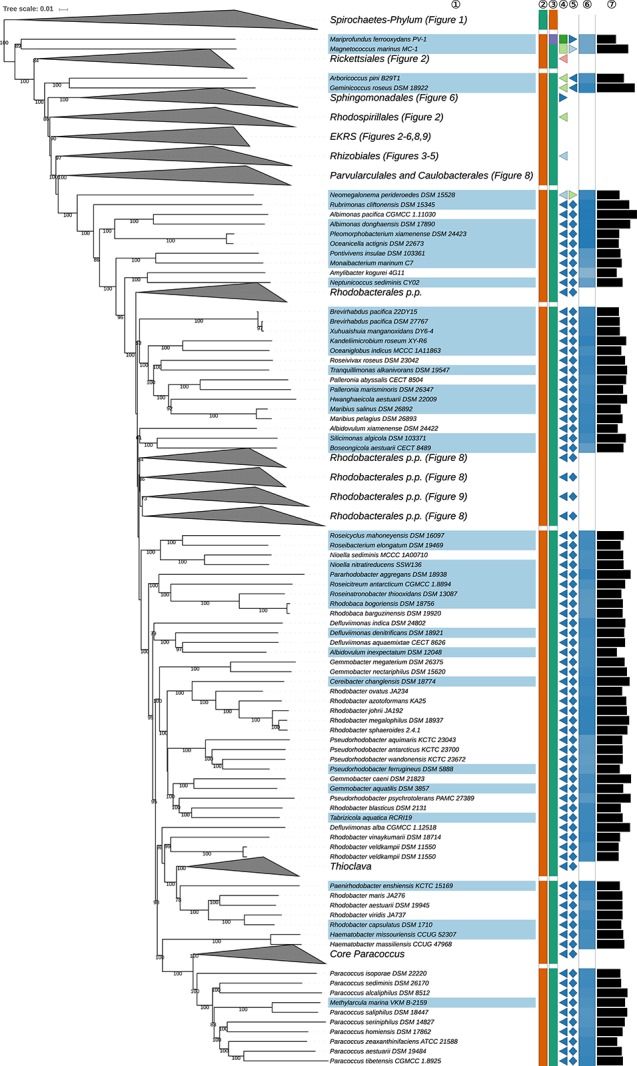
Sixth part of the GBDP tree shown in Figure 1, focussing on parts of *Rhodobacterales*. The clade labeled EKRS comprises the orders *Emcibacterales, Kordiimonadales, Rhodothalassiales*, and *Sneathiellales*, details for which are shown in other figures. Tip labels with light-blue background indicate type species of genera, colors and symbols to the right of the tips indicate, from left to right, phylum, class, order and family; for details and abbreviations see Figure 1. The blue color gradient indicates the G+C content as calculated from the genome sequences, followed by black bars indicating the (approximate) genome size in base pairs. *Rhodobacter veldkampii* DSM 11550 is represented by two genome sequences (SAMN10866319, above; SAMN08535030, below).

**FIGURE 8:**
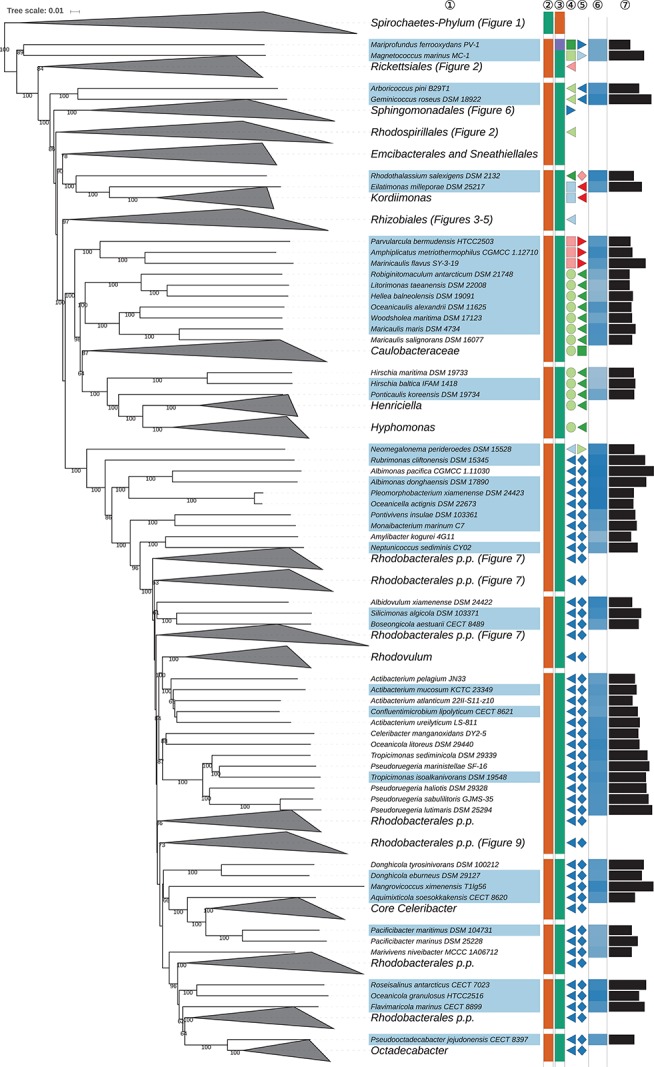
Seventh part of the GBDP tree shown in Figure 1, focussing on *Caulobacterales*, *Parvularculales* and parts of *Rhodobacterales*. Tip labels with light-blue background indicate type species of genera, colors and symbols to the right of the tips indicate, from left to right, phylum, class, order, and family; for details and abbreviations see Figure 1. The blue color gradient indicates the G+C content as calculated from the genome sequences, followed by black bars indicating the (approximate) genome size in base pairs.

The GBDP tree is shown in Figures 1–8; Figure 1 provides an overview and explains which specific sections of the same tree are displayed in greater detail in Figures 2–8. Table 1 shows dDDH results for pairs of type strains of interest, while Table 2 displays the results of the tests for phylogenetic inertia. Phenotypic information for groups of taxa whose taxonomic classification is treated in detail below is summarized in Supplementary Table S1. This Supplementary Table S1 also contains the complete list of genome sequences used in this study, including their GenBank and IMG accession numbers. Additional phylogenetic trees, including the GBDP tree in a single figure and with phenotypic annotation and the results from the gene-content analysis, are found in Supplementary File S2.

**TABLE 1:** Outcome of applying GGDC to calculate intergenomic dDDH values.

Strain 1	Strain 2	dDDH	Consequence
*Acetobacter* p*asteurianus* LMG 1262	*Acetobacter pasteurianus* subsp. *ascendens* LMG 1590	49	New species from subspecies
Acetobacter pasteurianus LMG 1262	*Acetobacter pasteurianus* subsp. *paradoxus* LMG 1591	49.8	New species from subspecies
*Acetobacter pasteurianus* subsp. *paradoxus* LMG 1591	*Acetobacter pasteurianus* subsp. *ascendens* LMG 1590	96.0	*A. pasteurianus* subsp. *paradoxus* is later heterotypic synonym
*Acetobacter peroxydans* ATCC 12874	*Acetobacter pasteurianus* LMG 1262	19	Not heterotypic synonyms (see discussion)
*Acetobacter peroxydans* ATCC 12874	*Acetobacter pasteurianus subsp*. *ascendens* LMG 1590	19	Not heterotypic synonyms (see discussion)
*Acetobacter* peroxydans ATCC 12874	*Acetobacter pasteurianus subsp*. *paradoxus* LMG 1591	19.3	Not heterotypic synonyms (see discussion)
*Acidiphilium angustum* ATCC 35903	*Acidiphilium rubrum* ATCC 35905	99.9	*A*. *angustum* is later heterotypic synonym
*Agrobacterium meteori* CECT 4293	*Ruegeria atlantica* CECT 4292	62.9	*A. meteori* is not later heterotypic synonym
*Alkalispirochaeta alkalica* DSM 8900	*Alkalispirochaeta sphaeroplastigenens* JC133	86.1	*A. sphaeroplastigenens* is later heterotypic synonym
*Borrelia bavariensis* PBi	*Borrelia garinii* CIP 103362	77.1	New subspecies of *B. garinii* from *B. bavariensis*
*Brucella melitensis* 16M	*Brucella ceti* NCTC 12891	97.8	*B. ceti* is later heterotypic synonym
*Brucella melitensis* 16M	*Brucella inopinata* BO1	81.2	*B. inopinata* is later heterotypic synonym
*Brucella melitensis* 16M	*Brucella microti* CCM 4915	98.1	*B. microti* is later heterotypic synonym
*Brucella melitensis* 16M	*Brucella vulpis* F60	80.5	*B. vulpis* is later heterotypic synonym
*Epibacterium mobile* DSM 23403	*Ruegeria pelagia* NBRC102038	76.9	New subspecies of *E. mobile* from *R. pelagia*
*Gluconobacter japonicus* LMG 1373	*Gluconobacter nephelii* LMG 26773	72.3	New subspecies of *G. japonicus* from species *G. nephelii*
*Hyphomonas neptunium* ATCC 15444	*Hyphomonas hirschiana* VP5	99.2	*H. hirschiana* is later heterotypic synonym
*Mameliella alba* DSM 26384	*Alkalimicrobium pacificum* F15	84.7	*A*. *pacificum* is later heterotypic synonym
*Mameliella alba* DSM 26384	*Mameliella atlantica* L6M1-5	84.2	*M. atlantica* is later heterotypic synonym
*Mameliella alba* DSM 26384	*Mameliella phaeodactyli* KD53	84.4	*M. phaeodactyli* is later heterotypic synonym
*Mameliella alba* DSM 26384	*Ponticoccus lacteus* JL351	100.0	*M*. *alba* is later heterotypic synonym
*Mameliella atlantica* L6M1-5	*Alkalimicrobium pacificum* F15	98.2	*M. atlantica* is later heterotypic synonym
*Mameliella phaeodactyli* KD53	*Alkalimicrobium pacificum* F15	83.9	*A. pacificum* is later heterotypic synonym
*Mameliella phaeodactyli* KD53	*Mameliella atlantica* L6M1-5	83.9	*M. atlantica* is later heterotypic synonym
*Mameliella phaeodactyli* KD53	*Ponticoccus lacteus* JL351	84.5	*M. phaeodactyli* is later heterotypic synonym
*Methylobacterium dichloromethanicum* DM4	*Methylobacterium chloromethanicum* CM4	73.6	New subspecies of *M. dichloromethanicum* from *M. chloromethanicum*
*Methylobacterium dichloromethanicum* DM4	*Methylorubrum extorquens* TK 0001	73.6	New subspecies of *M. dichloromethanicum* from *M. extorquens*
*Methylobacterium oryzae* CBMB20	*Methylobacterium phyllosphaerae* CBMB27	90.3	*M. phyllosphaerae* is later heterotypic synonym
*Methylobacterium radiotolerans* JCM 2831	*Methylobacterium organophilum* DSM 760	92.2	*M. organophilum* is later heterotypic synonym
*Oceanicella actignis* DSM 22673	*Pleomorphobacterium xiamenense* DSM 24423	88.4	*P. xiamenense* is later heterotypic synonym
*Ochrobactrum anthropi* ATCC 49188	*Ochrobactrum lupini* LUP21	83.9	*O. lupini* is later heterotypic synonym
*Paracoccus denitrificans* DSM 413	*Paracoccus pantotrophus* DSM 2944	42	Not heterotypic synonyms (see discussion)
*Paracoccus versutus* DSM 582	*Paracoccus bengalensis* DSM 17099	81.9	*P. bengalensis* is later heterotypic synonym
*Rhizobium marinum* MGL06	*Pseudorhizobium pelagicum* R1-200B4	76.3	New subspecies of *R. marinum* from *P. pelagicum*
*Rhizobium mongolense* USDA 1844	*Rhizobium loessense* CGMCC 1.3401	70.0	New subspecies of *R. mongolense* from species *R. loessense*
*Rhodobacter sphaeroides* 2.4.1	*Rhodobacter megalophilus* DSM 18937	81.6	*R. megalophilus* is later heterotypic synonym
*Rhodovulum viride* JA756	*Rhodovulum kholense* DSM 19783	84.1	*R. viride i*s later heterotypic synonym
*Rickettsia conorii* Malish 7	*Rickettsia gravesii* ATCC VR-1664	73.0	New subspecies of *R. conorii* from species *R. gravesii*
*Rickettsia conorii* Malish 7	*Rickettsia heilongjiangensis* 054	76.7	New subspecies of *R. conorii* from species *R. heilongjiangensis (also LHT of subsp. Japonica – see below)*
*Rickettsia conorii* Malish 7	*Rickettsia honei* RB	85	*R. honei* is later heterotypic synonym
*Rickettsia conorii* Malish 7	*Rickettsia japonica* YH	77.1	New subspecies of *R. conorii* from species *R. japonica*
*Rickettsia conorii* Malish 7	*Rickettsia raoultii* Khabarovsk	74.7	New subspecies of *R. conorii* from species *R*. *raoultii*
*Rickettsia conorii* Malish 7	*Rickettsia sibirica* 246	90	*R. sibirica* is later heterotypic synonym
*Rickettsia conorii* Malish 7	*Rickettsia slovaca* 13-B	90.7	*R. slovaca* is later heterotypic synonym
*Rickettsia heilongjiangensis* 054	*Rickettsia japonica* YH	92.4	*R. heilongjiangensis* is later heterotypic synonym
*Rickettsia buchneri* ISO-7	*Rickettsia tamurae* AT-1	73	New subspecies of *R. tamurae* from species *R. buchneri*
*Roseivivax atlanticus* 22II-S10s	*Roseivivax marinus* DSM 27511	82.8	*R. atlanticus* is later heterotypic synonym
*Sphingobium indicum* MTCC 6364	*Sphingobium chinhatense* MTCC8598	82.9	*Sphingobium chinhatense* is later heterotypic synonym
*Sphingobium indicum* MTCC 6364	*Sphingobium lucknowense* CCM 7544	82.7	*Sphingobium lucknowense* is later heterotypic synonym
*Thalassobaculum salexigens* DSM 19539	*Thalassobaculum litoreum* DSM 18839	90.5	*T. salexigens* is later heterotypic synonym
*Xuhuaishuia manganoxidans* DY6-4	*Brevirhabdus pacifica* DSM 27767	99.5	*X. manganoxidans* is later heterotypic synonym

*Only results that yield taxonomic consequences are shown.*

**TABLE 2:** *P*-values from the tip-permutation test of the GBDP tree shown in Figures 1–9 and other results obtained for the selected genomic and phenotypic features.

Feature	Data type	Coverage	RI	*P*-value
Percent G+C content	Continuous	100%	0.736	1e-04
Approximate genome size in bp	Continuous	100%	0.627	1e-04
Cell length in μm	Continuous	74%	0.422	1e-04
Cell width in μm	Continuous	71%	0.303	1e-04
Motility by flagella	Discrete, binary	72%	0.584	1e-04
Relationship to oxygen	Discrete, ordered multi-state	99%	0.511	1e-04
Carotenoids	Discrete, binary	18%	0.513	1e-04
Bacteriochlorophyll	Discrete, binary	30%	0.454	1e-04
Average number of isoprene residues in major ubiquinones	Continuous	57%	0.476	1e-04

*Genome size in base pairs is necessarily approximate in many cases because of unfinished genome sequences. The retention index (RI) can be used to compare the fit of distinct characters to a tree. The RI is bound between 0.0 and 1.0; the maximum of 1.0 indicates a perfect fit without any homoplasies.*

### Classes and Orders

The taxon sampling used in the present study was not mainly intended to provide support for or against the monophyly of the class *Alphaproteobacteria*, or of the phylum *Proteobacteria* in general. The choice of the outgroup in the present study was not intended to indicate that the phylum *Spirochaetes* represents the sister group of *Alphaproteobacteria* but was motivated by uncertainty regarding the monophyly of *Proteobacteria* (Yarza et al., 2014). Inferring the tree depicted in Figure 1 again after removing the outgroup and rooting this reduced tree using least-squares dating yielded the same branching order for the ingroup, i.e., the root was located between the clade formed by *Magnetococcus* and *Mariprofundus* on the one hand and the remainder of the tree on the other hand. *Spirochaetes* may thus not be the ideal outgroup for *Alphaproteobacteria* phylogeny but the alternative rooting confirmed the depicted branching order.

Only a single issue regarding the classes became apparent in this study, and most of the orders of the class *Alphaproteobacteria* appeared as monophyletic in our analysis (Figure 1). The exceptions were mainly caused by specific genera taxonomically assigned to *Rhodospirillales* and particularly genera assigned to *Rhodobacterales* that were phylogenetically intermixed with the order currently called *Rhizobiales*.

*Alphaproteobacteria* appeared as paraphyletic in the GBDP tree (Figures 1, 2) since *Mariprofundus ferrooxydans* of *Zetaproteobacteria* (Emerson et al., 2007) formed a strongly supported clade together with the alphaproteobacterium *Magnetococcus marinus* (Bazylinski et al., 2013a). The clade even obtained reasonable support in the gene-content analysis (Supplementary File S2) and its two representatives displayed almost the same G+C content. In the original description of *M. ferrooxydans* (Emerson et al., 2007) a new class (*Zetaproteobacteria*), order (*Mariprofundales*) and family (*Mariprofundaceae*) were proposed in the Supplementary Material only. None of these names became validly published so far even though a corrected name, *Mariprofundia*, was suggested for *Zetaproteobacteria* in the meantime (Oren, 2017a).

In the originally presented 16S rRNA gene trees the placement of *Mariprofundus* has no strong support and *Magnetococcus marinus* could not yet be considered. The additionally presented protein phylogenies (RecA, GyrB) only partially showed support for the placement of *M. ferrooxydans* separate from *Alphaproteobacteria*. In the CCT we did not find strong support for the placement of *M. ferrooxydans* branching first within the ingroup (Supplementary File S2). Phenotypically, alphaproteobacterial taxa such as *Magnetococcus marinus* are capable of forming iron oxides much like *M. ferrooxydans*. Even though the filamentous iron oxyhydroxide and branched-chain fatty acids produced by this species may differentiate it from taxa with a similar ecology, this alone provides no evidence for a separate class. It thus makes sense to again propose a separate order and family for *Mariprofundus* but to tentatively assign it to the class *Alphaproteobacteria*; an alternative arrangement is to remove *Magnetococcus* from *Alphaproteobacteria*.

Within *Alphaproteobacteria*, *Rhodospirillales* appeared as paraphyletic in the GBDP tree and in the CCT (Figure 2 and Supplementary File S2) because *Kiloniella* (Wiese et al., 2009; Yang S.-H. et al., 2015) of *Kiloniellales* as well as *Roseospirillum* (Glaeser and Overmann, 1999) and *Terasakiella* (Satomi et al., 2002; Han et al., 2016) of the order currently called *Rhizobiales* were nested within *Rhodospirillales* with high support. As this also affects the monophyly of the family *Rhodospirillaceae*, we will below propose the reclassification of these three genera into *Rhodospirillales* as the preferred way to restore a monophyletic order and family. Moreover, the distant position of *Geminicoccaceae* (Proença et al., 2018) also conflicts with the monophyly of *Rhodospirillales*. Because this conflict was only poorly supported, we do not propose taxonomic changes for *Geminicoccaceae* based on the here examined data.

*Rhodobacterales* were shown as non-monophyletic in various ways, most of which also affect families and will thus be treated below. *Rhizobiales* (Kuykendall, 2005) appeared as paraphyletic in GBDP tree (Figures 3–5 and Supplementary File S2) because *Acuticoccus* (Hou et al., 2015) *Ahrensia* (Uchino et al., 1998; Liu J. et al., 2016) *Labrenzia* (Biebl et al., 2007*;* Bibi et al., 2014) *Nesiotobacter* (Donachie et al., 2006) *Pannonibacter* (Borsodi et al., 2003; Biebl et al., 2007) *Pseudovibrio* (Shieh et al., 2004) *Roseibium* (Suzuki et al., 2000) and *Stappia* (Uchino et al., 1998; Biebl et al., 2007) all of which are currently classified in *Rhodobacterales*, were nested within *Rhizobiales*. According taxonomic solutions are suggested below for the affected families. *Hartmannibacter* (Suarez et al., 2014) which was not explicitly assigned to an order in its original description, is also treated below.

It should also be noted that *Rhizobiales* (Kuykendall, 2005) is validly published but illegitimate (i.e., not in accordance with the rules of the *International Code of Nomenclature of Prokaryotes*) as this order includes *Hyphomicrobium* of *Hyphomonadaceae*, type genus of *Hyphomicrobiales* (Douglas, 1957) which has priority. Our analyses do not call for placing *Rhizobium* and *Hyphomicrobium* in distinct orders (Figure 4 and Supplementary File S2), hence we will below propose an emended description of *Hyphomicrobiales* to replace the illegitimate *Rhizobiales*. The following description of the results will nevertheless use the name *Rhizobiales* throughout because this is the name used in our taxonomic input data although the name will be marked as in need of a replacement.

*Neomegalonema* (Oren, 2017b) of *Rhizobiales* (*Hyphomicrobiales*) was phylogenetically placed as sister group of *Rhodobacteraceae* with strong support to the exclusion of *Rhizobiales* (Figure 7), an arrangement that also obtained reasonable support in the gene-content analysis (Supplementary File S2). *Neomegalonema* was originally proposed as *Meganema* (Thomsen et al., 2006) which was later on regarded as an illegitimate name, and supposed to be related to the “*Methylobacterium*/*Xanthobacter* group” within *Alphaproteobacteria* based on a 16S rRNA gene analysis with a reduced taxon sampling and without calculating branch support. Given the phylogenetic evidence presented here and the lack of evidence to the contrary, the genus should be transferred from *Rhizobiales* (*Hyphomicrobiales*) to *Rhodobacterales*. Instead of assigning the genus to the already highly divergent and mainly marine family *Rhodobacteraceae* we suggest to place *Neomegalonema* into a family of its own within *Rhodobacterales*. This proposal is in accordance with the major phenotypic features of the genus (Supplementary Table S1).

### Outgroup Families and Genera

A revision of the phylum *Spirochaetes* is beyond the scope of the current study but some cautionary remarks on its taxonomic classification seem appropriate.

*Spirochaetaceae* (Swellengrebel, 1907; Abt et al., 2012; Gupta et al., 2013) were shown as rather heterogeneous assemblage not particularly well supported as monophyletic in the GBDP tree and in the CCT (Figure 2 and Supplementary File S2). While *Borreliaceae* (Gupta et al., 2013) were already separated from *Spirochaetaceae*, the overall genomic and phenotypic divergence of the group still calls for splitting *Spirochaetaceae*. The current taxonomic placement of *Sphaerochaeta* (Abt et al., 2012; Ritalahti et al., 2012; Miyazaki et al., 2014a; Arroua et al., 2017) which does not form spiral-shaped cells (Supplementary Table S1), makes *Spirochaetaceae* phenotypically heterogeneous. Spiral-shaped cells are apparently an apomorphy of the phylum and thus plesiomorphic within the phylum, hence this feature does not provide evidence for grouping *Treponema* within *Spirochaetaceae*.

An earlier taxonomic study already proposed the family *Treponemataceae* (Robinson, 1948) to accommodate the type genus *Treponema* (Schaudinn, 1905; Abt et al., 2013). Hence, we suggest to reuse the validly published name *Treponemataceae* and to place *Sphaerochaeta* in a phenotypically homogeneous family of its own. According to the CCT and UCT (Supplementary File S2), *Pleomorphochaeta* (Arroua et al., 2017) which like *Sphaerochaeta* displays spherical cells (Supplementary File S1), should be assigned to *Sphaerochaetaceae* fam. nov., too, whereas *Rectinema* (Koelschbach et al., 2017) should be placed in *Treponemataceae*. *Rectinema* produces non-motile rods but, according to its phylogenetic position (Supplementary File S2), the development of non-helical cells occurred independently of *Pleomorphochaeta* and *Sphaerochaeta.* In contrast to *Spirochaetaceae sensu stricto* as proposed here, *Sphaerochaetaceae* fam. nov. and *Treponemataceae* were strongly supported by the gene-content analysis (Supplementary File S2).

As for the outgroup genera, *Spirochaeta* (Pikuta et al., 2009; Miyazaki et al., 2014b; Shivani et al., 2015) of *Spirochaetaceae* was shown as paraphyletic in the GBDP tree and in the CCT (Figure 2 and Supplementary File S2) because *Alkalispirochaeta* (Sravanthi et al., 2016) and *Salinispira* (Ben Hania et al., 2015) were nested within *Spirochaeta*. These problems were already visible in the original literature sources whose 16S rRNA gene analyses indicated that the proposal of these new genera would render *Spirochaeta* paraphyletic. A major obstacle in treating the genus with modern taxonomic methods is that the type species of *Spirochaeta*, *Spirochaeta plicatilis*, is not represented by a type strain (Skerman et al., 1980). One potential albeit radical solution is to place all other *Spirochaeta* species into genera of their own. Because of overall insufficient resolution in the 16S rRNA gene trees (Supplementary File S2), we refrain from a taxonomic revisions of the genus because genome sequences of relevance are not yet available. The classification of *Spirochaetaceae sensu stricto* should be reconsidered once more genome sequences become available.

### *Rickettsiales* Families

*Rickettsiales* is an order of *Alphaproteobacteria* that comprises pathogens with reduced genomes and a reduced G+C content (Figure 2). Our analysis did not call the monophyly of the order into question, but a nomenclatural issue became apparent in the course of this study.

We found that *Anaplasmataceae* (Philip, 1957; Dumler et al., 2001) is illegitimate if one takes its last emendation into account as this family now includes *Ehrlichia* (Moshkovski, 1945) which is the type genus of *Ehrlichiaceae* (Moshkovski, 1945) which has priority. Our analyses do not argue for placing the two genera, which form a clade strongly supported even by the gene-content analysis, into distinct families (Figure 1 and Supplementary File S2), hence we will propose below an emended description of *Ehrlichiaceae* as the correct name for the family of the genera currently included in *Anaplasmataceae.*

### *Rhodospirillales* Families

A variety of familie*s* of *Rhodospirillales* appeared in need of a taxonomic revision in the light of our analyses (Figure 2). These discrepancies were not only caused by *Rhodospirillales* appearing to be intermixed with *Kiloniellales*.

*Acetobacteraceae* (Gillis and De Ley, 1980) were shown as non-monophyletic in the GBDP tree and in the CCT (Figure 2 and Supplementary File S2) because of the relatively isolated position of *Stella* (Vasilyeva, 1985) and the even more distinct position of *Zavarzinia* (Meyer et al., 1993). In the original description of *Zavarzinia*, a phylogenetic analysis could not yet be conducted, and the genus was not assigned to any family. Later the genus was assigned to *Acetobacteraceae* (Boone et al., 2001b) but the reasoning behind this remained unclear. Here *Zavarzinia* was shown as branching first within *Rhodospirillales*. While the backbone support within the order was low, strong support indicates that *Zavarzinia* does not belong to core *Acetobacteraceae*. Given its isolated position in the GBDP and 16S rRNA gene trees, the genus is best assigned to a family of its own. This taxonomic consequence did not appear in conflict with the major phenotypic features of *Zavarzinia* (Supplementary Table S1).

*Stella* appeared as sister group of the equally deviating genus of *Rhodospirillaceae*, *Reyranella* (Pagnier et al., 2011) with moderate support, and separated from core *Acetobacteraceae* by long branches. The original description of *Stella* did not explicitly assign the genus to a family. *Stella* was placed in *Acetobacteraceae* in Bergey’s manual (Boone et al., 2001a); whether this placement was based on a phylogenetic assessment is unclear. Phenotypically the genus is rather unique because of its star-like morphology (Supplementary Table S1). All results thus suggest placing *Stella* into a family of its own. The same holds for *Reyranella*, which is treated below; the gene-content analysis does not support the sister-group relationship of the two genera.

When *Constrictibacter* (Yamada et al., 2011) was proposed it was placed in *Rhodospirillaceae* in a 16S rRNA gene tree with low support values. In the CCT (Supplementary File S2) with its much broader taxon sampling *Constrictibacter* formed a clade together with *Stella* albeit with low support. We thus tentatively include *Constrictibacter* in the family newly proposed to accommodate *Stella* (see above). Although phenotypic differences in morphology and respiration might suggest to alternatively place *Constrictibacter* into a family of its own this solution should be postponed until a *Constrictibacter* genome sequence is available.

*Rhodospirillaceae* (Pfennig and Trüper, 1971) appeared as paraphyletic in GBDP tree and in the CCT (Figure 2 and Supplementary File S2) for a variety of reasons. For instance, *Roseospirillum* (Glaeser and Overmann, 1999) of *Rhodobiaceae* was nested within *Rhodospirillaceae*. *Roseospirillum* was placed as sister group of *Rhodospira* (Pfennig et al., 1997) with high support (Figure 2), a clade that in turn formed, with high support, the sister group of a clade containing the type species of the type genus of the family, *Rhodospirillum rubrum* (Molisch, 1907) these arrangements are even supported by the gene-content analysis (Supplementary File S2). For these reasons, we propose to include *Roseospirillum* into *Rhodospirillaceae*, which is also supported by the high phenotypic agreement between them (Supplementary Table S1). When *Roseospirillum* was proposed, bootstrapping was not conducted, and only few species could be included in the phylogenetic analysis at that time.

Furthermore, *Ferrovibrio* (Sorokina et al., 2012) *Taonella* (Xi et al., 2013) and *Marinibaculum* (Yu et al., 2016) formed an isolated but strongly supported clade with *Sneathiellaceae* (Kurahashi et al., 2008) in the CCT (Supplementary File S2). When *Ferrovibrio* was proposed, it was already placed in the order *Sneathiellales* but not explicitly in *Sneathiellaceae*. When *Taonella* and *Marinibaculum* were proposed both publications lacked strong support for the positioning of the respective taxa and both publications did not sample *Sneathiella* (Jordan et al., 2007). Therefore, we propose to tentatively assign *Ferrovibrio*, *Taonella*, and *Marinibaculum* to *Sneathiellaceae*. These taxonomic consequences are not precluded by the phenotypic features of the involved genera (Supplementary Table S1).

Similarly, *Terasakiella* (Satomi et al., 2002; Han et al., 2016) of *Methylocystaceae* and *Kiloniella* (Wiese et al., 2009; Yang S.-H. et al., 2015) of *Kiloniellaceae* were nested within *Rhodospirillaceae* (Figure 2). However, even if these genera were included in *Rhodospirillaceae*, the family would lack any support. For this reason, to address the remaining causes of the non-monophyly of *Rhodospirillaceae* other measures are advisable. *Rhodospirillaceae* would not appear monophyletic even if these three genera were included. *Rhodospirillaceae* showed high genomic divergence (Figure 2) and unsupported by the gene-content analysis (Supplementary File S2). The family is phenotypically heterogeneous, as, e.g., most of its representatives are phototrophic whereas some genera are chemoorganoheterotrophs (Supplementary Table S1). Splitting *Rhodospirillaceae* into several families corresponding to well-supported clades can also solve this issue.

*Azospirillaceae* fam. nov. is thus proposed in agreement with the GBDP tree (Figure 2 and Supplementary File S2) to accommodate *Niveispirillum* (Lin et al., 2014) *Azospirillum* (Tarrand et al., 1978; Falk et al., 1985) *Rhodocista* (Kawasaki et al., 1993) and *Skermanella* (Sly and Stackebrandt, 1999; Luo et al., 2012; Weon et al., 2007) *Nitrospirillum* (Lin et al., 2014) and *Desertibacter* (Liu et al., 2011) are also assigned to this family based on the 16S rRNA gene analyses (Supplementary File S2). Even though the gene-content analysis does not support the clade but only its two major subclades, this conclusion fits well to the major phenotypic features of these genera (Supplementary Table S1), most of which also show larger genomes than the other genera currently included in *Rhodospirillaceae*. *Azospirillum* and *Rhodocista* were placed in *Rhodospirillaceae* in Bergey’s manual (Boone et al., 2001a) but it remained unclear to us whether this was based on a phylogenetic assessment. The proposals of these genera directly assigned them to *Rhodospirillaceae* but phylogenetic support for the monophyly of the family was not presented. The situation regarding genera such as *Elstera* (Rahalkar et al., 2012) and *Inquilinus* (Coenye et al., 2002) is less clear in the GBDP tree and particularly in the 16S rRNA gene trees, even the constrained ones. This holds also for *Lacibacterium* (Sheu et al., 2013) which lacks a published genome but was found to be the sister taxon of *Elstera* according to 16S rRNA gene analyses when it was proposed (Sheu et al., 2013) and in our findings (Supplementary File S2). These genera are tentatively assigned to the new family *Azospirillaceae*, too. Further reclassifications should be attempted once more genome sequences become available.

When *Terasakiella* was originally described, it was not assigned to any family. *Terasakiella* was placed in *Methylocystaceae* later on (Garrity et al., 2003b) but this proposal did not appear to be based on a phylogenetic analysis. The last emendation of the genus (Yoon and Kang, 2018) still assigned *Terasakiella* to *Methylocystaceae* but did not include the type species of the family in the phylogenetic analysis. We conclude that the taxonomic literature contains no phylogenetic evidence for an affiliation of the genus to the family. Similarly, *Magnetovibrio* (Williams et al., 2012) *Thalassospira* (López-López et al., 2002), and *Varunaivibrio* (Patwardhan and Vetriani, 2016) were placed in *Rhodospirillaceae* based on largely unresolved 16S rRNA gene trees with a reduced taxon sampling. Given the phylogenetic evidence presented here (Figure 2 and Supplementary File S2), we propose *Thalassospiraceae* fam. nov. to accommodate *Magnetovibrio* (Bazylinski et al., 2013b) *Terasakiella*, *Thalassospira* (López-López et al., 2002; Liu et al., 2007; Tsubouchi et al., 2014) and *Varunaivibrio*. *Magnetospira* (Williams et al., 2012) is also tentatively assigned to this family based on to the 16S rRNA gene analyses (Supplementary File S2). These taxonomic consequences are not precluded by the phenotypic features of the involved genera (Supplementary Table S1). The gene-content analysis does not support the newly proposed family but genome size and G+C content are rather homogeneous (Figure 2 and Supplementary File S2).

When *Kiloniella, Kiloniellaceae* and *Kiloniellales* were proposed (Wiese et al., 2009) the phylogenetic analysis was characterized by low support at the backbone, and the monophyly of the resulting orders and families was not immediately evident. The most conservative solution for the observed discrepancy (Figure 2) is to retain *Kiloniellaceae* but to place it in *Rhodospirillales*. *Aestuariispira* (Park et al., 2014e) should according to our phylogenetic results also be placed in the family. *Aestuariispira* was placed in *Rhodospirillaceae* when the genus was originally proposed but this was based on a partially rather unresolved 16S rRNA gene tree that lacked a representative sampling of *Alphaproteobacteria*. Among the known major phenotypic features, it differs from *Kiloniella* only regarding motility (Supplementary Table S1). The clade comprising *Kiloniella* and *Aestuariispira* also receives strong support in the gene-content analysis (Supplementary File S2).

*Marivibrio* (Chen S. et al., 2017b) was originally placed in *Rhodospirillaceae* based on a 16S rRNA gene analysis with low taxon sampling in which *Marivibrio* was grouped together with *Pelagibius* of *Rhodospirillaceae*. In the CCT *Marivibrio* was isolated from *Rhodospirillaceae* but formed a clade together with *Kiloniella* of *Kiloniellaceae* and *Aestuariispira* as its sister taxon. Because of low support in the CCT we propose to tentatively include *Marivibrio* into *Kiloniellaceae.* Although phenotypic differences might suggest the alternative of assigning *Marivibrio* to its own family we here refrain from this solution due to a lack of genomic data.

When *Thalassocola* (Lin et al., 2015) was proposed it was placed in *Phyllobacteriaceae* in a 16S rRNA gene tree with low support. In the CCT (Supplementary File S2) *Kiloniella* of *Kiloniellaceae* appeared as the sister taxon of *Thalassocola* albeit with low support. We thus propose to tentatively include *Thalassocola* into *Kiloniellaceae* which is taxonomically more favorable to its current inclusion in *Phyllobacteriaceae.* Although phenotypic differences might suggest the alternative of assigning *Thalassocola* to its own family we refrain from this solution because of the low resolution and because it would be taxonomically less conservative.

Given that the non-monophyly of *Rhodospirillaceae* should be solved by splitting rather than by merging, the trees inferred from genome-scale data (Figure 2 and Supplementary File S2) also call for the proposal of *Rhodovibrionaceae* fam. nov. to accommodate *Fodinicurvata* (Wang Y. et al., 2009) *Limimonas* (Amoozegar et al., 2013) *Rhodovibrio* (Imhoff et al., 1998) and *Tistlia* (Díaz-Cárdenas et al., 2010) *Limibacillus* (Kim et al., 2015) and *Pelagibius* (Choi et al., 2009) are also tentatively assigned to this family based on the 16S rRNA gene analyses (Supplementary File S2). The creation of this family is neither contradicted by the gene-content analysis (even though it does not provide support) nor by major phenotypic features of the involved genera (Supplementary Table S1). For instance, while the resulting family is heterogeneous regarding the occurrence of phototrophy and related pigments, these character states appear relatively scattered across the phylogeny (Figure 2 and Supplementary File S2). *Rhodovibrio* was placed in *Rhodospirillaceae* in Bergey’s manual (Boone et al., 2001a) but it remained unclear to us whether this was based on a phylogenetic assessment. As in the case of the deviating genera of *Rhodospirillaceae* treated above, the original proposals of *Fodinicurvata*, *Limibacillus, Limimonas*, and *Tistlia* assigned these genera to the family, each time based on 16S rRNA gene trees that were either largely unresolved or lacked a representative taxon sampling, i.e., strong phylogenetic evidence for the monophyly of *Rhodospirillaceae* including these genera was not presented. In contrast, at least moderate bootstrap support was obtained in the taxonomic literature (Amoozegar et al., 2013) for a clade comprising *Fodinicurvata*, *Limibacillus, Limimonas*, *Rhodovibrio*, and *Tistlia*, in accordance with our results.

Based on the phylogenetic results (Figure 2 and Supplementary File S2), *Thalassobaculaceae* fam. nov. is proposed to contain *Nisaea* (Urios et al., 2008) *Oceanibaculum* (Lai et al., 2009; Dong et al., 2010) and *Thalassobaculum* (Zhang G.I. et al., 2008; Urios et al., 2010). These genera were placed in *Rhodospirillaceae* when they were originally proposed, each time based on 16S rRNA gene trees that were either largely unresolved or lacked a representative taxon sampling, i.e., there was no strong phylogenetic evidence for the monophyly of *Rhodospirillaceae* including these genera in the taxonomic literature. We did not find any significant phenotypic differences between these genera (Supplementary Table S1). The monophyly of this family obtained no support in the gene-content analysis but moderate support in the 16S rRNA gene analyses (Supplementary File S2).

While taxonomically assigned to *Rhodospirillaceae*, *Reyranella massiliensis* (Pagnier et al., 2011) displayed an isolated position in the phylogenomic tree (Figure 2), distant to the type genus of the family. When *Reyranella* was proposed, it appeared as sister group of *Magnetospirillum magnetotacticum* (Schleifer et al., 1991) of *Rhodospirillaceae* but with low support only. More importantly, the low taxon sampling in that study did not allow for safely assigning *Reyranella* to a family; the same holds for the subsequent emendations of the genus (Kim et al., 2013; Lee H. et al., 2017) *Reyranella* is best assigned to a family of its own, which is not contradicted by the phenotype (Supplementary Table S1).

For several genera of *Rhodospirillaceae*, namely *Aliidongia* (Chen et al., 2017a), *Dongia* (Liu Y. et al., 2010), *Constrictibacter* (Yamada et al., 2011), *Defluviicoccus* (Maszenan et al., 2005), *Marivibrio* (Chen S. et al., 2017b), *Tagaea* (Jean et al., 2016), and *Tistrella* (Shi et al., 2002) our CCT results (Supplementary File S2) questioned their assignment to *Rhodospirillaceae*. *Aliidongia* was placed in *Rhodospirillaceae* when the genus was originally proposed but this was based on a 16S rRNA gene analysis with low taxon sampling in which *Aliidongia* was grouped together with *Inquilinus* and *Dongia* of *Rhodospirillaceae* with low support. When *Dongia* was originally proposed, it grouped together in a 16S rRNA gene tree with *Rhodospirillaceae* genera including *Azospirillum*, *Rhodocista* and *Skermanella*, but with overall low taxon sampling. When *Tagaea* was originally proposed it was placed in a 16S rRNA gene tree in a well supported clade together with *Oceanibaculum*, *Nisaea*, and *Thalassobaculum* of *Rhodospirillaceae*. Yet the placement of the clade itself showed low support and overall taxon sampling lacked the type genus of *Rhodospirillaceae*. None of these genera could safely be placed in a family in the CCT (Supplementary File S2) hence we recommend to regard *Aliidongia, Dongia*, and *Tagaea* as genera *incertae sedis* until their phylogenetic position can be clarified once more genome sequences become available.

*Defluviicoccus* (Maszenan et al., 2005) was placed in *Rhodospirillaceae* (Garrity et al., 2007) after its original description but based on the original 16S rRNA gene analysis wherein *Defluviicoccus* was placed together with *Rhodospirillaceae* genera such as *Rhodospirillum, Azospirillum*, and *Magnetospirillum* yet with low taxon sampling. *Tistrella* was originally not placed in any family but was placed in *Rhodospirillaceae* later on in Bergey’s Manual (Garrity et al., 2003b) which cited the original description of *Tistrella* even though it had cautioned against an assignment to a family because of low support in 16S rRNA gene analysis. *Defluviicoccus* and *Tistrella* were isolated from *Rhodospirillaceae* in the far better sampled CCT and formed a clade together with *Geminicoccus* and *Arboricoccus* of *Geminicoccaceae*. Support for this arrangement was also low, hence we suggest the tentative inclusion of *Defluviicoccus* and *Tistrella* in *Geminicoccaceae.* Although phenotypic differences might suggest to alternatively assign both genera to their own family we refrain from this solution due to a lack of genomic data.

### *Rhodospirillales* Genera

Some genera of *Rhodospirillales* also appeared in need of a taxonomic revision in the light of our analyses (Figure 2) although to a lesser extent than the families of the order.

Within *Rhodospirillales*, *Azospirillum* (Tarrand et al., 1978; Falk et al., 1985) was shown as paraphyletic in the GBDP and in the 16S trees (Figure 2 and Supplementary File S2) because *Azospirillum irakense* (Khammas et al., 1989) was placed as sister group of *Niveispirillum* (Cai et al., 2015, 2018b) with high support. While its genome sequence was lacking at the time of writing, the CCT showed that the type species of *Niveispirillum, N. fermenti* (Lin et al., 2014) was also placed in this clade, whereas the type species of *Azospirillum*, *A. lipoferum*, was placed in a clade together with the four other *Azospirillum* species represented in the GBDP tree. These arrangements also obtained support in the gene-content analysis (Supplementary File S2). It was already proposed to reclassify *A. irakense* as *N. irakense* (Lin et al., 2014) but this name was not validly published, and hence *Niveispirillum* remained non-monophyletic. While *A. irakense* differs from *Niveispirillum* by its microaerophilic lifestyle (Supplementary Table S1), this alone is not a sufficient reason to separate two genera. We accordingly propose to place *A. irakense* in *Niveispirillum.*

*Magnetospirillum* (Schleifer et al., 1991) appeared as paraphyletic in the GBDP tree (Figure 2) because *Phaeospirillum molischianum* (Giesberger, 1947; Imhoff et al., 1998) was nested within *Magnetospirillum. Telmatospirillum siberiense* (Sizova et al., 2007) formed the sister group of this clade. In addition, in the CCT, *P. chandramohanii* (Anil Kumar et al., 2009), *P. fulvum* (van Niel, 1944; Imhoff et al., 1998), *P. oryzae* (Lakshmi et al., 2011), and *P. tilakii* (Raj et al., 2012) were also nested within *Magnetospirillum* (Supplementary File S2). When *Magnetospirillum* was proposed, the phylogenetic analysis was characterized by a low number of included taxa and by a lack of support values. When *Phaeospirillum* was proposed, a well resolved tree was presented that included only few species; in particular, *Magnetospirillum* was not considered (Imhoff et al., 1998). Hence, phylogenetic evidence for the separation of the two genera is lacking. *P. chandramohanii, P. fulvum, P. molischianum, P. oryzae*, and *P. tilakii* display a phenotype similar to the one of *Magnetospirillum* (Supplementary Table S1). Consequently, it is proposed that *P. chandramohanii, P. fulvum, P. molischianum, P. oryzae* and *P. tilakii* be classified in *Magnetospirillum.*

*Roseomonas* (Rihs et al., 1993; Sánchez-Porro et al., 2009; Venkata Ramana et al., 2010) was shown as polyphyletic in the GBDP tree (Figure 2) because *Roseomonas stagni* (Furuhata et al., 2008) and *Roseomonas lacus* (Jiang et al., 2006; Sánchez-Porro et al., 2009) were placed as sister group of *Humitalea rosea* (Margesin and Zhang, 2013) and *Rubritepida flocculans* (Alarico et al., 2002) respectively, albeit with low support. In the CCT, species from further genera, such as *Rhodovarius lipocyclicus* (Kämpfer et al., 2004) were nested within the main *Roseomonas* clade while *Roseomonas fauriae* (Rihs et al., 1993) was placed in a remote position as sister group of *Azospirillum formosense* (Lin et al., 2012) (Supplementary File S2). A previous study concluded that *R. fauriae* is a later heterotypic synonym of *A. formosense* (Helsel et al., 2006). While a genome sequence of the type strain of the type species of *Roseomonas*, *R. gilardii*, was lacking at the time of writing, it is represented by *R. gilardii* subsp. *rosea* in the GBDP tree and safely placed in the CCT. It would be premature, however, to propose new genera for *Roseomonas lacus* and *R. stagni* since the low resolution in even the constrained 16S rRNA gene trees currently hinders the assignment of those species not represented by a genome sequence to the resulting set of genera.

*Gluconacetobacter* (Yamada et al., 1997) appeared as polyphyletic in the GBDP tree (Figure 2) because *G. entanii* (Schüller et al., 2000) was nested within *Komagataeibacter* (Yamada et al., 2012) with high support both in the GBDP and the 16S rRNA gene trees (Figure 2 and Supplementary File S2); even the gene-content analysis provided support. When *Komagataeibacter* was proposed to harbour species formerly placed in *Gluconacetobacter* (Yamada et al., 2012), *G. entanii* could not be transferred to the new genus because the type strain seemed unavailable from any culture collection. As long as this problem remains unsolved a new combination for *G. entanii* cannot be proposed (Parker et al., 2019).

### *Rhizobiales* (*Hyphomicrobiales*) Families

Many families of *Rhizobiales* (*Hyphomicrobiales*) appeared to be in need of a taxonomic revision according to our results (Figures 3–5), even more so than in *Rhodospirillales*. The discrepancies were on the one hand caused by *Rhizobiales* (*Hyphomicrobiales*) families which appeared intermixed and on the other hand caused by genera taxonomically assigned to *Rhodobacteraceae* within *Rhodobacterales* but phylogenetically placed within *Rhizobiales* (*Hyphomicrobiales*). Both kinds of cases are treated in this section.

Within *Rhizobiales* (*Hyphomicrobiales*), *Rhodobiaceae* (Garrity et al., 2005f) were shown as non-monophyletic in the GBDP tree and in the CCT (Figures 3–5 and Supplementary File S2) because representatives of this family were placed into phylogenetically quite distant clades. *Parvibaculum* (Schleheck et al., 2004) was placed together with *Tepidicaulis* (Takeuchi et al., 2015) in a clade that appeared as sister group (Figure 3) of the remaining *Rhizobiales* (*Hyphomicrobiales*). When *Parvibaculum* was proposed, a phylogenetic tree was not presented, and the genus was not assigned to any family. The original description of *Tepidicaulis* showed it as sister group of *Parvibaculum* with strong support. The gene-content analysis also strongly supported their sister-group relationship (Supplementary File S2). Thus placing this clade into a family separate from *Rhodobiaceae* appeared as most appropriate solution. This is not precluded by the phenotype of the involved genera (Supplementary Table S1). Additional genera may need to be added to the newly proposed family once genome sequences provide sufficient resolution.

Similarly, *Lutibaculum* (Anil Kumar et al., 2012) and *Tepidamorphus* (Albuquerque et al., 2010) formed a strongly supported clade of their own (Figure 3) within *Rhizobiales* (*Hyphomicrobiales*), without any obvious affiliation to an already proposed family. The phylogenies presented in the original descriptions of *Lutibaculum* and *Tepidamorphus* already suffered from low support at the backbone; our analyses of the 16S rRNA gene did not show any significant conflict with the phylogenomic tree either. Thus placing these two genera into a family separate from *Rhodobiaceae* appears as most appropriate solution. According to the 16S rRNA gene analyses (Supplementary File S2), *Butyratibacter* (Wang et al., 2017) and *Microbaculum* (Su et al., 2017) should also be placed in this family. This is not precluded by the phenotype of the involved genera (Supplementary Table S1). The gene-content analysis did not support the sister-group relationship of *Lutibaculum* and *Tepidamorphus* but they display quite similar G+C content values and genome sizes (Supplementary File S2).

The type genus of the family, *Rhodobium* (Hiraishi et al., 1995; Urdiain et al., 2008) was placed in an isolated position relative to the rest of the family (Figure 4), only showing a weakly supported sister-group relationship to *Afifella* (Urdiain et al., 2008). The original description of *Afifella* was characterized by the lack of branch support and low taxon sampling of the phylogenetic analysis; an assignment of the genus to a family was not proposed. Later on (Su et al., 2017) *Afifella* was regarded as affiliated to *Rhodobiaceae*. A sister-group relationship of *Afifella* and *Rhodobium* is possible but only supported by the gene-content analysis, which is not the phylogenetic method of choice, while unsupported in the GBDP and 16S rRNA gene trees (Figure 4 and Supplementary File S2). Additional supermatrix analyses indicated that *Afifella* and *Rhodobium* do not form a clade (Supplementary File S2). For this reason, a new family is proposed to accommodate *Afifella*, which is not in disagreement with its phenotype (Supplementary Table S1).

The CCT called the assignment of *Anderseniella* (Brettar et al., 2007) and *Rhodoligotrophos* (Fukuda et al., 2012) to *Rhodobiaceae* into question (Supplementary File S2). *Anderseniella* was regarded as affiliated to *Rhodobiaceae* only after its original description (Su et al., 2017) *Rhodoligotrophos* was placed in *Rhodobiaceae* when it was originally proposed, which was based on a 16S rRNA gene tree that grouped *Rhodoligotrophos* together with *Parvibaculum* of *Rhodobiaceae*. Yet this tree showed a non-monophyletic *Rhodobiaceae*. In the CCT *Anderseniella* and *Rhodoligotrophos* formed a clade together with *Parvibaculum* (Schleheck et al., 2004) and *Tepidicaulis* (Takeuchi et al., 2015) which we propose to transfer to the new family *Parvibaculaceae* (see above). *Anderseniella* and *Rhodoligotrophos* may only tentatively be assigned to *Parvibaculaceae* because of low support in the CCT. As *Pyruvatibacter* (Wang G. et al., 2016) has not been assigned to a family yet and is located in the same clade we would also tentatively assign *Pyruvatibacter* to *Parvibaculaceae.*

*Rhizobiaceae* (Conn, 1938) were also shown as non-monophyletic in the GBDP tree and in the CCT (Figure 3 and Supplementary File S2) for several reasons. The genus *Kaistia* (Im et al., 2004) formed a well-supported subtree distinct from core *Rhizobiaceae* and close to *Bauldia* (Yee et al., 2010) which was not yet assigned to a family. The phylogenetic analysis of the 16S rRNA gene used for the proposal of *Bauldia* was characterized by low support at the backbone of the tree. In its original description *Kaistia* was not assigned to a family (Im et al., 2004); the genus was placed in *Rhizobiaceae* only later on (Garrity et al., 2007) but the rationale behind this decision remained obscure. The phenotype of the two genera is largely in agreement (Supplementary Table S1) and there is a certain amount of support for the clade in the gene-content analysis (Supplementary File S2). We accordingly propose a new family *Kaistiaceae*, fam. nov., to accommodate *Kaistia* and *Bauldia.*

As for *Hyphomicrobiaceae*, it should be mentioned that although *Gemmiger* (Gossling and Moore, 1975) was listed in Bergey’s Manual (Boone et al., 2001a) as a genus of *Hyphomicrobiaceae* it was not considered in our analysis because a later study (Yarza et al., 2013) showed that *Gemmiger* does phylogenetically not even belong to the phylum *Proteobacteria*. Its taxonomic classification is beyond the scope of the current study.

Even apart from that genus, *Hyphomicrobiaceae* (Babudieri, 1950) appeared as non-monophyletic in the GBDP and in 16S gene rRNA trees (Figure 3 and Supplementary File S2) in other respects because *Aquabacter* (Irgens et al., 1991), *Blastochloris* (Hiraishi, 1997) and *Prosthecomicrobium* (Staley, 1984) as well as a clade comprising the genera *Cucumibacter* (Hwang and Cho, 2008b), *Devosia* (Nakagawa and Yokotat, 1996; Rivas et al., 2003), *Maritalea* (Hwang et al., 2009b) and *Pelagibacterium* (Xu et al., 2011) were placed apart from the core *Hyphomicrobiaceae* clade that comprised the type genus of *Hyphomicrobiaceae.* Although the genome sequence of the type strain of the type species of the type genus of the family, *Hyphomicrobium vulgare* (Stutzer and Hartleb, 1899), was not available at the time of writing, the CCT and UCT placed the species with strong support within the clade comprising *H. nitrativorans* and *H. zavarzini* (Supplementary File S2). *Hyphomicrobiaceae* also appeared as the taxonomic home for *Methyloceanibacter* (Vekeman et al., 2016), which was originally not assigned to a family but was here located within core *Hyphomicrobiaceae* in Figure 3.

When *Aquabacter* was proposed (Irgens et al., 1991), a phylogenetic tree was not presented, and the publication did not assign the genus to a family. *Aquabacter* was placed in *Hyphomicrobiaceae* in Bergey’s manual (Boone et al., 2001a) but the reason for doing so was not obvious to us. Including *Aquabacter* into *Xanthobacteraceae* appears to be the most conservative solution, which is not precluded by the phenotype (Supplementary Table S1). The *Aquabacter*-*Azorhizobium*-*Xanthobacter* clade obtained high support in the gene-content analysis while the 16S rRNA gene analyses even indicated that *Aquabacter*, *Xanthobacter* and *Azorhizobium* are difficult to discern as currently circumscribed (Supplementary File S2). This problem should be addressed once more type-strain genome sequences from the group become available.

The original description of *Prosthecomicrobium* and its type species, *P. hirschii*, did not include a 16S rRNA gene analysis (Staley, 1968, 1984). The current assignment of this genus to *Hyphomicrobiaceae* was called into question in the literature (Lee et al., 2005) and could not be confirmed by our study either. *Prosthecomicrobium* was first placed in *Hyphomicrobiaceae* in Bergey’s manual (Boone et al., 2001b) but this assignment may not be based on a phylogenetic analysis. In the CCT (Supplementary File S2) *Prosthecomicrobium* forms a strongly supported clade with the genera *Ancalomicrobium* and *Pinisolibacter* (Staley, 1968; Dahal et al., 2018). Therefore we propose to place *Prosthecomicrobium* in *Ancalomicrobiaceae* (Dahal et al., 2018) which is not precluded by the phenotypic characteristics of these genera (Supplementary Table S1).

*Phreatobacter* (Tóth et al., 2014; Lee S.D. et al., 2017) was as yet not assigned to a family, whereas *Blastochloris* (Hiraishi, 1997) was assigned by some authors to *Hyphomicrobiaceae* (Garrity et al., 2003b), although other studies concluded that this genus cannot safely be assigned to a family based on 16S rRNA gene data (Lee et al., 2005). The GBDP tree showed both genera located within a highly supported clade also comprising *Bradyrhizobiaceae* and *Xanthobacteraceae* but the interrelationships between these four subclades remained unresolved (Figure 3). Similarly, the 16S rRNA gene analyses did not resolve the placement of these two isolated genera and did not indicate the affiliation to any existing family (Supplementary File S2). For this reason, we propose to assign each of the two genera to a family of its own. This solution is not precluded by the phenotype of the two genera (Supplementary Table S1).

The CCT (Supplementary File S2) called the assignment of *Rhodoplanes* (Hiraishi and Ueda, 1994b) to *Hyphomicrobiaceae* into question. *Rhodoplanes* was placed in *Hyphomicrobiaceae* by Bergey’s Manual (Boone et al., 2001b) but the reason behind this decision remained unclear. In the CCT *Rhodoplanes* formed a clade together with genera of *Bradyrhizobiaceae* (Garrity et al., 2005e) such as *Variibacter* (Kim et al., 2014), *Afipia* (Brenner et al., 1991) and *Nitrobacter* (Winogradsky, 1892) *Pseudolabrys* (Kämpfer et al., 2006) and *Pseudorhodoplanes* (Tirandaz et al., 2015) appeared as closest relatives in the CCT. Because of low clade support we propose to tentatively assign *Rhodoplanes* to the same family as these genera; the nomenclature of *Bradyrhizobiaceae* is treated in detail below.

In the CCT (Supplementary File S2) *Angulomicrobium* (Vasil’eva et al., 1980) and *Methylorhabdus* (Doronina et al., 1995) of *Hyphomicrobiaceae* formed a strongly supported clade within *Xanthobacteraceae*. When *Angulomicrobium* was proposed it was not assigned to a family but later on Bergey’s manual (Boone et al., 2001a) listed *Angulomicrobium* as a genus of *Hyphomicrobiaceae.* This was confirmed by 16S rRNA gene and lipid-composition analysis (Fritz et al., 2004) but branch support in the presented 16S rRNA gene tree was not shown and the analysis did not consider *Xanthobacter* (Wiegel et al., 1978), which is now the type genus of *Xanthobacteraceae*. When *Methylorhabdus* was proposed it was not assigned to a family but later on Bergey’s manual (Boone et al., 2001a) listed *Methylorhabdus* as a genus of *Hyphomicrobiaceae.* A more recent edition of Bergeys manual (Brenner et al., 2005) mentioned that the highest similarity in DNA:DNA hybridization of *Methylorhabdus* was observed with *Xanthobacter* and only 10% with *Hyphomicrobium*. Due to their position in the CCT we propose to assign *Angulomicrobium* and *Methylorhabdus* to *Xanthobacteraceae.* This solution is not in conflict with their phenotype (Supplementary Table S1).

*Xanthobacteraceae* (Lee et al., 2005) appeared as both paraphyletic and polyphyletic in the GBDP tree and the CCT (Figure 3 and Supplementary File S2) because *Pseudoxanthobacter* (Arun et al., 2008) was placed apart from the remaining *Xanthobacteraceae* while genera of other families, such as *Aquabacter spiritensis* of *Hyphomicrobiaceae*, were placed within *Xanthobacteraceae*. The original description of *Pseudoxanthobacter* did not explicitly assign the genus to a family, and the shown phylogeny suffered from low support at the backbone. The assignment of *Pseudoxanthobacter* to *Xanthobacteraceae* occurred only later (Ueki et al., 2010) based on mere sequence similarity search. According to the relatively isolated and not maximally supported position of *Pseudoxanthobacter* in the phylogenomic trees (Figure 3 and Supplementary File S2) and the lack of evidence supporting its inclusion in *Xanthobacteraceae* it is proposed to assign it to a family of its own. This solution is not in conflict with the phenotype (Supplementary Table S1) and not in conflict with the gene-content analysis (Supplementary File S2).

While *Pleomorphomonas* (Xie and Yokota, 2005) was taxonomically assigned to *Methylocystaceae*, it here appeared as only distantly related to the type genus of the family (Figure 3). In contrast, a highly supported clade included both *Pleomorphomonas* and a set of genera not yet assigned to family, *Hartmannibacter* (Suarez et al., 2014), *Methylobrevis* (Poroshina et al., 2015), *Mongoliimonas* (Xi et al., 2017) and *Oharaeibacter* (Lv et al., 2017). The 16S rRNA gene analyses (Supplementary File S2) also provided support for this clade, additionally including *Chthonobacter* (Kim et al., 2017). In line with the taxonomic consequences proposed above, the phylogenetic results call for suggesting a new family for these genera. Although the gene-content analysis did not provide support for the group, the establishment of this new family did not appear to be in conflict with the phenotype of the involved genera (Supplementary Table S1).

*Bradyrhizobiaceae* (Garrity et al., 2005e) and *Xanthobacteraceae* (Lee et al., 2005) were shown as non-monophyletic in the GBDP tree (Figure 3) due to the position of *Pseudolabrys* (Kämpfer et al., 2006) of *Xanthobacteraceae*, which was placed within *Bradyrhizobiaceae* together with *Pseudorhodoplanes* (Tirandaz et al., 2015), a genus that was as yet not assigned to a family. *Bradyrhizobiaceae* is actually illegitimate as this family includes *Nitrobacter* (Buchanan, 1917), which is the type genus of *Nitrobacteraceae* (Buchanan, 1917), which has priority (Tindall, 2019a). Our analyses do not call for placing *Nitrobacter* and *Bradyrhizobium* in distinct families (Figure 3 and Supplementary File S2), hence we will below propose an emended description of *Nitrobacteraceae* as the correct name for the family of the genera currently assigned to the illegitimate *Bradyrhizobiaceae*. The following description of the results will nevertheless use the name *Bradyrhizobiaceae* throughout because this is the name used in our input data but the name will be marked as in need of a replacement.

*Bradyrhizobiaceae* (*Nitrobacteraceae*) also appeared as non-monophyletic in the GBDP tree (Figure 3) because the genera *Rhodoblastus* (Imhoff, 2001) and *Bosea* (Das et al., 1996; La Scola et al., 2003) were placed apart from core *Bradyrhizobiaceae* (*Nitrobacteraceae*). *Rhodoblastus* formed the sister group of *Roseiarcus* (Kulichevskaya et al., 2014) which is currently taxonomically assigned to its own family. This clade in turn formed the sister group of core *Methylocystaceae* including its type genus *Methylocystis* (Bowman et al., 1993), whereas the subsequent sister taxon was *Beijerinckiaceae* (Garrity et al., 2005d). In the CCT, *Rhodoblastus* was shown as closely related to *Roseiarcus* with strong support whereas the relationship of this clade to *Alsobacter* (Bao et al., 2014) was only poorly supported. While *Roseiarcus* was taxonomically placed in *Roseiarcaceae* when it was originally proposed, in its original description *Rhodoblastus* was not assigned to a family, and a phylogenetic analysis was not performed. *Rhodoblastus* was placed in *Bradyrhizobiaceae* (*Nitrobacteraceae*) in a later study (Garrity et al., 2003b) but the rationale behind this decision remained obscure. When *Roseiarcaceae* was proposed, *Rhodoblastus* was considered but the backbone of the 16S rRNA gene tree was only partially resolved. *Roseiarcaceae* has priority over *Alsobacteraceae* (Sun et al., 2018), hence the possibility of a later unification of the family does not preclude the assignment of *Rhodoblastus* to *Roseiarcaceae*, which we propose below. This solution is not conflict with the phenotype either (Supplementary File S2).

*Bradyrhizobiaceae* (*Nitrobacteraceae*) also appeared in the CCT as the taxonomic home for *Pseudorhodoplanes* (Tirandaz et al., 2015), which was originally not assigned to a family, as well as for *Pseudolabrys* (Kämpfer et al., 2006) of *Xanthobacteraceae.* When the latter was proposed the 16S rRNA tree presented showed poor support for the specific placement and the genus was only assigned to the class *Alphaproteobacteria* in general. *Pseudolabrys* was later on placed in *Xanthobacteraceae* (Lin et al., 2015) based on a 16S rRNA gene analysis with low support. The CCT (Supplementary File S2) indicated that *Pseudolabrys* forms a clade with *Bradyrhizobiaceae* (*Nitrobacteraceae*) genera (Garrity et al., 2005e) namely *Variibacter* (Kim et al., 2014), *Afipia* (Brenner et al., 1991) and *Nitrobacter* (Winogradsky, 1892) *Pseudorhodoplanes* (Tirandaz et al., 2015) appeared as the sister genus of *Pseudolabrys* with strong support, even in the gene-content analysis. We thus propose to include *Pseudolabrys* in *Bradyrhizobiaceae* (*Nitrobacteraceae*), which is not precluded by its phenotype (Supplementary Table S1).

*Bosea* as well as *Salinarimonas* (Liu J.-H. et al., 2010) which was originally (Cai et al., 2011b) also assigned to *Bradyrhizobiaceae* (*Nitrobacteraceae*) but later on to a family of its own (Cole et al., 2018), were placed within a strongly supported clade (Figure 3 and Supplementary File S2) containing *Methylobacteriaceae*, *Camelimonas* (Kämpfer et al., 2010b) *Chelatococcus asaccharovorans* (Auling et al., 1993), and *Chelatococcus sambhunathii* (Panday and Das, 2010). A similar arrangement was observed in the CCT with high support (Supplementary File S2). In the original description of *Bosea*, bootstrapping was not conducted, and only few species could be considered at that time. Neither its original description nor its emendation explicitly assigned *Bosea* to a family. The genus was placed in *Bradyrhizobiaceae* (*Nitrobacteraceae*) in Bergey’s manual (Boone et al., 2001a) but the rationale was unclear. Other studies emphasized the uncertain placement of *Bosea* in 16S rRNA gene trees, much like the placement of *Chelatococcus* (Lee et al., 2005). When *Chelatococcus* was proposed and emended (Auling et al., 1993; Yoon et al., 2008) it already appeared as closely related to *Methylobacteriaceae* with moderate support. *Chelatococcus* was placed in *Beijerinckiaceae* in Bergey’s manual but the reason for this decision remained unclear. *Camelimonas* was regarded as belonging to *Beijerinckiaceae* when *Thalassocola* was proposed (Lin et al., 2015) but the included 16S rRNA gene tree did not show a monophyletic *Beijerinckiaceae*. In contrast, the recent proposal to place *Chelatococcus* and *Camelimonas* in a separate family *Chelatococcaceae* (Dedysh et al., 2016) is in agreement with our results. Since the phylogenetic placement of *Bosea* does not allow for an inclusion of the genus into any of the families within the clade, the taxonomically most conservative solution is to create a new family, *Boseaceae* fam. nov.

When *Enterovirga* (Chen et al., 2017d) was proposed it was acknowledged that this genus shows high 16S rRNA similarities with several *Methylobacteriaceae* genera but also with *Chelatococcus* and *Pseudochelatococcus* of *Beijerinckiaceae* and *Bosea* of *Bradyrhizobiaceae* (*Nitrobacteraceae*). However, the presented 16S rRNA gene tree had insufficient support for assigning *Enterovirga* to a family. In the CCT (Supplementary File S2) *Enterovirga* was nested within *Methylobacteriaceae* (Garrity et al., 2005c) in a moderately supported clade. Given its phylogenetic position, it is thus proposed to tentatively include *Enterovirga* in *Methylobacteriaceae*, which is not precluded by its phenotype (Supplementary Table S1).

The original descriptions of *Cucumibacter*, *Maritalea* and *Pelagibacterium* (Figure 4) provided strong support for a clade comprising these genera and *Devosia*. In these studies, the overall taxon sampling was insufficient to safely assign these genera to a family, even though the affiliation to *Hyphomicrobiaceae* was not called into question. When *Devosia* was originally proposed it was not assigned to a family, and even its later emendations (Rivas et al., 2003; Yoo et al., 2006; Yoon et al., 2007c; Zhang D.C. et al., 2012) hesitated to explicitly suggest an affiliation of the genus to a family. Previous studies already concluded that *Devosia* and *Prosthecomicrobium* cannot safely be assigned to a family based on 16S rRNA gene data (Lee et al., 2005). The CCT and UCT provided strong support for a clade comprising *Arsenicitalea* (Mu et al., 2016) *Cucumibacter*, *Devosia*, *Maritalea*, *Methyloterrigena* (Kim H.S. et al., 2016), *Paradevosia* (Geng et al., 2014), *Pelagibacterium* and *Youhaiella* (Wang Y.X. et al., 2015). It makes phylogenetic sense to taxonomically remove these genera from *Hyphomicrobiaceae* and assign them to a new family, which is not precluded by their phenotype (Supplementary Table S1).

*Rhodobacteraceae* (Garrity et al., 2005b) of *Rhodobacterales* were shown as non-monophyletic in the GBDP tree because the genera *Acuticoccus* (Hou et al., 2015), *Ahrensia* (Uchino et al., 1998; Liu J. et al., 2016), *Labrenzia* (Biebl et al., 2007; Bibi et al., 2014), *Nesiotobacter* (Donachie et al., 2006; Garrity et al., 2007), *Pannonibacter* (Borsodi et al., 2003; Biebl et al., 2007), *Pseudovibrio* (Shieh et al., 2004), *Roseibium* (Suzuki et al., 2000) and *Stappia* (Uchino et al., 1998; Biebl et al., 2007) were placed apart from the remaining *Rhodobacteraceae* and within *Rhizobiales* (*Hyphomicrobiales*) instead where they also caused the non-monophyly of some families (Figure 4). It should be noted that *Rhodobacteraceae* as originally proposed is illegitimate because the family included *Hyphomonas* (Moore et al., 1984), the type genus of *Hyphomonadaceae* (Lee et al., 2005), which has priority.

*Acuticoccus* showed a relatively isolated position (Figure 4) but also strong support for its sister-group relationship to *Amorphus* (Zeevi Ben Yosef et al., 2008), which was as yet not assigned to a family. It is questionable whether the restricted taxon sampling in the phylogenetic analysis that accompanied the original description of *Acuticoccus* really allowed for an assignment to a family but the genus was taxonomically placed in *Rhodobacteraceae* (Hou et al., 2015). Phenotypically, the features of *Acuticoccus* may be rather rare in *Alphaproteobacteria* as gliding motility was reported for the genus, whereas *Amorphus* was described as not-motile (Supplementary Table S1). For reasons of taxonomic conservatism, the two genera are best assigned to the same family, which is supported by the gene-content analysis (Supplementary File S2).

The original description of *Stappia* did not assign it to a family; this was done in an edition of Bergey’s manual (Boone et al., 2001a) but it is not obvious whether this was based on a phylogenetic analysis. The same holds for the assignment of *Labrenzia* (Cai et al., 2011a), *Pannonibacter* (Garrity et al., 2003b), and *Pseudovibrio* (Garrity et al., 2007) to *Rhodobacteraceae*. Later studies, such as the proposal of *Nesiotobacter* (Donachie et al., 2006), also failed to conduct a comparison with the type genus of *Rhodobacteraceae*. Rather, these studies assigned newly proposed genera to *Rhodobacteraceae* based on the closeness of these genera to genera that now appeared to phylogenetically not belong to the family. *Labrenzia*, *Nesiotobacter*, *Pannonibacter*, and *Roseibium* formed a moderately to well supported clade even in the unconstrained 16S rRNA gene analyses (Supplementary File S2). The GBDP topology (Figure 4) differs regarding the positioning of *Breoghania* (Gallego et al., 2010) but this discrepancy was not well supported. When *Breoghania* was proposed and assigned to *Cohaesibacteraceae*, none of the genera with which *Cohaesibacteraceae* now appeared intermixed were included in the taxon sampling. Additional supermatrix analyses (Supplementary File S2) indicated that *Breoghania* forms the sister group of a clade comprising the six genera, whereas *Cohaesibacter* (Hwang and Cho, 2008a) branches first. Given the results from the analysis of the 16S rRNA gene and genome-scale data it seems advisable to propose a new family to accommodate *Breoghania* as well as another new family to harbour *Labrenzia*, *Pannonibacter*, *Pseudovibrio* (including *Nesiotobacter* as proposed below), *Roseibium* and *Stappia*. These solutions are not in conflict with the phenotype of the involved genera (Supplementary Table S1).

Among the misplaced *Rhodobacteraceae, Ahrensia* formed an independent lineage with relatively uncertain affiliations to other families (Figure 5). The original description of *Ahrensia* did not assign it to a family, whereas its last emendation assigned it to *Phyllobacteriaceae* (Liu J. et al., 2016) Bergey’s manual placed *Ahrensia* in *Rhodobacteraceae* (Boone et al., 2001a) but whether this was based on a phylogenetic assessment remained unclear to us. Similarly, the assignment of *Nesiotobacter* to *Rhodobacteraceae* (Garrity et al., 2007) may not have been based on phylogenetic argumentation. Given the phylogenomic results presented here, *Ahrensia* is best assigned to a separate family, which is not precluded by its phenotype (Supplementary Table S1). According to the 16S rRNA gene analyses, *Pseudahrensia* (Jung et al., 2012c) should also be assigned to this family (Supplementary File S2).

*Phyllobacteriaceae* (Mergaert and Swings, 2005) appeared as paraphyletic in the GBDP tree (Figure 5) because the genera *Aminobacter* (Urakami et al., 1992; Kämpfer et al., 2002a), *Nitratireductor* (Labbé et al., 2004; Jang et al., 2011), *Pseudaminobacter* (Kämpfer et al., 1999), *Aquamicrobium* (Bambauer et al., 1998; Lipski and Kämpfer, 2012; Wu Z.-G. et al., 2014), *Mesorhizobium* (Jarvis et al., 1997), *Hoeflea* (Peix et al., 2005; Rahul et al., 2015) and *Zhengella* (Liao et al., 2018) were placed apart from the clade containing the type genus of the family, *Phyllobacterium*. *Zhengella* formed the sister group of *Notoacmeibacter* (Huang et al., 2017) of *Notoacmeibacteraceae* with reasonable support, even in the gene-content analysis (Supplementary File S2). The taxonomically most conservative solution is to also assign *Zhengella* to this family, which is not precluded by the major phenotypic features found in the genus (Supplementary Table S1).

The original descriptions of *Mesorhizobium* and *Pseudaminobacter* (Figure 5) did not explicitly assign these genera to a family. The recent emendations of *Aquamicrobium* (Figure 5) were accompanied by phylogenetic trees with low support at the backbone and a taxon sampling which we tend to regard as insufficient for safely assigning a genus to a family. The three genera were placed in *Phyllobacteriaceae* in an edition of Bergey’s manual (Boone et al., 2001a) but it remained unclear to us whether this was based on a phylogenetic assessment. *Nitratireductor* (Figure 5) was assigned to *Phyllobacteriaceae* when it was originally proposed (Labbé et al., 2004) but this was based on a poorly resolved 16S rRNA gene tree with reduced taxon sampling. When *Aquamicrobium* (Bambauer et al., 1998) was proposed, bootstrapping was not conducted, and at that time only few species could be considered in the phylogenetic analysis. Insufficient taxon sampling was also present in the original description of *Nitratireductor*, in addition to poor branch support; the most recent emendation of the genus did not include the type species of the type genus of the family either. In the CCT, *Nitratireductor*, *Pseudaminobacter*, *Aminobacter*, *Aquamicrobium*, and *Mesorhizobium* formed a clade together with further genera such as *Chelativorans* (Doronina et al., 2010; Kämpfer et al., 2015a) and *Carbophilus* (Meyer et al., 1993) but branch support was extraordinarily low throughout (Supplementary File S2). For this reason, we refrain from taxonomic conclusions for these genera. The issue should be tackled once more type-strain genome sequences from the clade become available.

During our investigation we noticed that *Chelativorans intermedius* (Kämpfer et al., 2015a) (Supplementary File S2) was described as Gram-positive although the opposite is mentioned in the abstract of the same publication. Furthermore the description for *Chelativorans* (Doronina et al., 2010; Kämpfer et al., 2015a) defines the genus as Gram-negative. Gram-positivity would be unlikely for the whole family of *Phyllobacteriaceae* (Brenner et al., 2005) in general. We suspect this was an oversight of the authors and *Chelativorans intermedius* is most likely Gram-negative.

When *Hoeflea* was proposed, it was included in the family *Phyllobacteriaceae* even though this conclusion remained phylogenetically unsupported (Peix et al., 2005). Our phylogenomic analysis suggests the inclusion of *Hoeflea* in *Rhizobiaceae* (Figure 5 and Supplementary File S2). While the marine bacterium *Hoeflea* differs from *Rhizobiaceae* also regarding the presence of photosynthetic pigments, this character is highly homoplastic within the class *Alphaproteobacteria*. Assigning *Hoeflea* to *Rhizobiaceae* is not in conflict with its phenotype (Supplementary Table S1) and more conservative than generating a new family to accommodate the genus.

In the CCT (Supplementary File S2) *Lentilitoribacter* (Park et al., 2013b) of *Phyllobacteriaceae* (Mergaert and Swings, 2005) was nested within *Hoeflea* (Peix et al., 2005; Rahul et al., 2015) with reasonable support. When *Lentilitoribacter* was originally proposed it was placed in *Phyllobacteriaceae* in a 16S rRNA gene tree which lacked support and did not include *H. phototrophica*, which formed the sister group of *Lentilitoribacter* in the CCT. Although we refrain from resolving the paraphyly of *Hoeflea* until additional genomic data become available we propose to include *Lentilitoribacter* in *Rhizobiaceae*, which is not precluded by its phenotype (Supplementary File S1).

*Aurantimonadaceae* (Garrity et al., 2003a) were shown as polyphyletic in the GBDP tree (Figures 4, 5) because *Martelella endophytica* (Bibi et al., 2013) and *Martelella mediterranea* (Rivas et al., 2005) were placed apart from core *Aurantimonadaceae* (Figure 4) and within a strongly supported clade (Figure 5) that included *Rhizobiaceae* and *Mycoplana* of *Brucellaceae*. When *Martelella* and its type species, *M. mediterranea*, were proposed, they were not assigned to any family and phylogenetically not placed within *Aurantimonadaceae* but rather within *Rhizobiaceae. Martelella* was placed in *Aurantimonadaceae* later on (Garrity et al., 2007) but the rationale behind this decision remained obscure. The CCT did not indicate the monophyly of *Aurantimonadaceae* either (Supplementary File S2). Consequently, it is proposed to include *Martelella* into *Rhizobiaceae.* This is not contradicted by the phenotypic characteristics (Supplementary Table S1). *Aurantimonadaceae* was originally proposed without a formal description and is not a validly published name. Therefore we newly propose *Aurantimonadaceae*, fam. nov., with a revised (reduced) set of genera to belong to this family.

The position of *Mycoplana dimorpha* (Urakami et al., 1990) which appeared nested within *Rhizobiaceae* (Conn, 1938) in Figure 5, also rendered *Brucellaceae* (Breed et al., 1957) polyphyletic in the GBDP tree; core *Brucellaceae* is shown in Figure 4. In the CCT, in addition to *Mycoplana dimorpha*, *Mycoplana ramosa* (Urakami et al., 1990) was also placed within the clade containing *Rhizobiaceae* (Supplementary File S2). *Mycoplana* (Gray and Thornton, 1928; Urakami et al., 1990) was originally proposed on basis of phenotypic characteristics without taking into account the 16S rRNA gene sequence as phylogenetic marker; it was even supposed to belong to *Mycobacteriaceae* (*Actinobacteria*). Later on *Mycoplana* was placed in *Brucellaceae* (Boone et al., 2001a) but the rationale behind this assignment remained unclear. *Mycoplana* displays phenotypic characteristics similar to those of *Rhizobiaceae* (Supplementary Table S1). Given its phylogenetic positioning, it is thus proposed to include *Mycoplana* in *Rhizobiaceae.*

*Notoacmeibacter* (Huang et al., 2017) not only formed a strongly supported clade together with *Mabikibacter* of *Mabikibacteraceae* (Choi et al., 2017) in the CCT but the distance between the two 16S rRNA gene sequences was exceptionally low (Supplementary File S2). The monotypic family *Mabikibacteraceae* was proposed briefly after *Notoacmeibacteraceae* hence neither publication was able to account for the respective other taxon. The high similarity of 99.9% in the 16S rRNA gene sequences (Meier-Kolthoff et al., 2013b) and the highly similar phenotypic characteristics (Supplementary File S1) could even indicate identity at the species level but solving this issue required genome sequencing of the type strain of *Mabikibacter*. We thus here only propose to transfer *Mabikibacter* to *Notoacmeibacteraceae*, which has priority. This transfer would also avoid the non-monophyly of *Notoacmeibacteraceae* that resulted if only *Zhengella* were to be added to this family (as treated above).

### *Rhizobiales* (*Hyphomicrobiales*) Genera

In addition to some families of *Rhizobiales* (*Hyphomicrobiales*), a variety of genera assigned to the order were found in need of a taxonomic revision, while the majority of the genera was shown as monophyletic (Figures 3–5). This section also treats the *Rhodobacterales* genera that needed to be taxonomically assigned to *Rhizobiales* (*Hyphomicrobiales*) as explained above in case their genus boundaries also needed to be reconsidered.

Within *Rhizobiales* (*Hyphomicrobiales*), *Afipia* (Brenner et al., 1991; La Scola et al., 2002) was shown as paraphyletic in the GBDP tree (Figure 3) because *Oligotropha carboxidovorans* (Meyer et al., 1993) formed the sister group of the type species of *Afipia*, *Afipia felis* (Brenner et al., 1991). *O. carboxidovorans* was originally proposed as a new species in a new genus on the basis of morphological, physiological and biochemical characteristics, as well as 16S rRNA signature oligonucleotides. However, in the original publication no phylogenetic tree was inferred and it was not clarified which of the character states represent apomorphies. The known phenotypic differences between the two genera are not pronounced (Supplementary Table S1). To include *O. carboxidovorans* in *Afipia*, which has priority, seems to be the taxonomically most conservative solution.

*Methylobacterium* (Patt et al., 1976) appeared non-monophyletic in the GBDP tree (Figure 3) because *Methylorubrum* (Green and Ardley, 2018) species such as *Methylorubrum salsuginis, M. populi* and *M. chloromethanicum* were nested within *Methylobacterium*. All species were only recently transferred from *Methylobacterium* to *Methylorubrum* (Green and Ardley, 2018). The purpose of splitting *Methylobacterium* was to obtain phenotypically more homogeneous genera. Yet if such an approach yields apparently non-monophyletic groups it is in conflict with the principles of phylogenetic systematics (Hennig, 1965; Klenk and Göker, 2010; Wiley and Lieberman, 2011). In particular, the cited study emphasized that only certain clades of *Methylobacterium sensu lato* should be assigned to either *Methylobacterium sensu stricto* or to *Methylorubrum* but new genera and new combinations for the remaining clades were not proposed. Since species names cannot lack a genus name, it would come as no surprise if this solution rendered *Methylobacterium* non-monophyletic. The utilization of methylamine as sole carbon source in *Methylorubrum* was suggested as the main feature for distinguishing it from *Methylobacterium sensu stricto* (Green and Ardley, 2018). But a single binary character cannot properly separate two taxa according to the principles of phylogenetic systematics because only one of the two character states is an apomorphy (Nouioui et al., 2018). Because of the low resolution in the 16S rRNA gene trees (Supplementary File S2) it seems more appropriate to treat the *Methylorubrum* species as species of *Methylobacterium* until more type-strain genome sequences from the group become available and the splitting of the genus can be completed.

*Pseudovibrio* (Shieh et al., 2004) was shown as paraphyletic in the GBDP tree (Figure 4) because *Nesiotobacter exalbescens* (Donachie et al., 2006) was nested within *Pseudovibrio* with moderate support. When *Nesiotobacter* was proposed, the genus *Pseudovibrio* was not considered for the phylogenetic analysis of the 16S rRNA gene. While our 16S gene analyses (Supplementary File S2) showed *Nesiotobacter* as sister group of *Pseudovibrio*, *Nesiotobacter exalbescens* and *Pseudovibrio* display similar phenotypic characteristics (Supplementary Table S1). The gene-content analysis provided support for the entire clade but not for all of the subclades (Supplementary File S2). Therefore, we propose to include *N. exalbescens* into *Pseudovibrio* because the rationale of separating the two genera was not obvious.

*Labrenzia* (Biebl et al., 2007; Bibi et al., 2014) appeared as paraphyletic in the GBDP tree (Figure 4) because *Roseibium denhamense* (Suzuki et al., 2000; Biebl et al., 2007) and *R. hamelinense*as (Suzuki et al., 2000; Biebl et al., 2007) were nested within *Labrenzia*, with *L. suaedae* (Bibi et al., 2014) branching first. *Pannonibacter* (Borsodi et al., 2003; Biebl et al., 2007) formed the sister group of the entire clade. In the CCT, *Roseibium aquae* (Zhong et al., 2014) and *R. sediminis* (Liu et al., 2017) were also placed within *Labrenzia* (Supplementary File S2). While the original proposals of *Roseibium* and *L. suaedae* showed phylogenetic trees with 83–93% support for the monophyly of *Roseibium*, the species now included in *Labrenzia* could not be considered when *Roseibium* was introduced. Support was already weak for the monophyly of *Labrenzia* when it was originally proposed. The paraphyly of *Labrenzia* was already evident in a more recent study (Camacho et al., 2016), which also showed an uncertain position of *L. suaedae* relative to the remaining genus. The *Roseibium* species display phenotypic characteristics similar to those of *Labrenzia* (Supplementary Table S1) while the gene-content analysis did not yield well-supported subgroups that could be proposed as genera (Supplementary File S2). For this reason, the best solution appears to be to merge the two genera, which is also the taxonomically most conservative approach.

*Ochrobactrum* (Holmes et al., 1988) was shown as paraphyletic in the GBDP tree (Figure 4) because the entire genus *Brucella* (Meyer and Shaw, 1920) was nested within *Ochrobactrum*. *O. thiophenivorans* (Kämpfer et al., 2008), *O. grignonense* (Lebuhn et al., 2000), *O. pituitosum* (Huber et al., 2010), *O. rhizosphaerae* (Kämpfer et al., 2008), *O. pseudogrignonense* (Kämpfer et al., 2007a) formed a clade that branched first, whereas the type species of *Ochrobactrum* appeared more closely related to *Brucella* than to this clade. In the CCT, *O. haematophilum* (Kämpfer et al., 2007a) and *O. pecoris* (Kämpfer et al., 2011) also belonged to the clade that branched first. The 16S rRNA gene data neither provided evidence for other *Ochrobactrum* species to form a clade with the type species to the exclusion of *Brucella*, including species such as *O. pseudintermedium* (Teyssier et al., 2007), *O. oryzae* (Tripathi et al., 2006), *O. gallinifaecis* (Kämpfer et al., 2003) and *O. endophyticum* (Li L. et al., 2016) (Supplementary File S2). When *Ochrobactrum* was proposed, an analysis of the 16S rRNA gene could not yet be conducted. When *Brucella* was proposed, a phylogenetic analysis was not conducted; it was speculated that the genus may belong to *Enterobacteriaceae* but an assignment to a family was not explicitly suggested. Among those taxonomic studies available to us that showed a phylogenetic analysis with a sufficient number of *Brucella* and *Ochrobactrum* species, a paraphyletic *Ochrobactrum* was always evident, particularly in the recent proposals of new species (Kämpfer et al., 2007a, b, 2008, 2009a, 2010a, 2013b, 2014; Lebuhn et al., 2000; Trujillo et al., 2005; Teyssier et al., 2007; Romanenko et al., 2008; Xu H.Y. et al., 2009; Huber et al., 2010; Imran et al., 2010; Woo et al., 2011; Li L. et al., 2016; Liu B.B. et al., 2016).

It is well known that in terms of DDH similarity, *Brucella* is only a single species (Verger et al., 1985); this is also reflected in the newly calculated dDDH values (Table 1). The overall genomic divergence of the *Brucella*-*Ochrobactrum* clade was lower than in many clades harboring a single genus only. *Brucella* differs from *Ochrobactrum* regarding its pathogenic lifestyle, which may be reflected in the lower genome size of *Brucella* (Figure 4). However, *Ochrobactrum* species are also known from clinical specimens, including its type species (Holmes et al., 1988), and a more pronounced genome-size reduction of pathogenic species nested within a partially non-pathogenic genus was observed elsewhere, as, e.g., in *Mycobacterium leprae* (Akinola, 2013). *Mycobacterium* can also serve as an example for a genus that harbours distinct risk groups (Bundesanstalt für Arbeitsschutz und Arbeitsmedizin, 2015), much like *Burkholderia* and *Yersinia*. Hence the difference between *Brucella* and *Ochrobactrum* regarding their risk-group assignment could hardly be used as an argument against their inclusion in the same genus. Known phenotypic differences, if any, appeared to be restricted to autapomorphies of *Brucella* that may well be linked to its evolutionary adaptation to pathogenesis (Supplementary Table S1). Despite the differences in genome size, the gene-content analysis provided more support for the combined *Brucella*–*Ochrobactrum* clade than for the subclades (Supplementary File S2). For these reasons, we propose to include *Ochrobactrum* in *Brucella*, which has priority. According to the available phenotypic information (Supplementary Table S1), the inclusion of the entire genus *Ochrobactrum* does not cause a need for the emendation of *Brucella* (Meyer and Shaw, 1920; Verger et al., 1985).

*Mesorhizobium* (Jarvis et al., 1997) appeared as non-monophyletic in the GBDP tree and in the CCT (Figure 5 and Supplementary File S2) since several *Mesorhizobium* species were intermixed with genera such as *Aquamicrobium* (Bambauer et al., 1998; Lipski and Kämpfer, 2012; Wu Z.-G. et al., 2014) *Nitratireductor* (Labbé et al., 2004) and *Pseudaminobacter* (Kämpfer et al., 1999) which were also shown as non-monophyletic. However, due to the low support of the branches, particularly in the comprehensive 16S rRNA gene trees, it is difficult to propose taxonomic conclusions for *Aquamicrobium*, *Mesorhizobium, Nitratireductor*, and *Pseudaminobacter*. These genera belong to a deviating branch of *Phyllobacteriaceae* that even proved to be difficult to classify at the family level, as explained above. We suppose that a more satisfying classification of these four genera can only be obtained once more genome sequences become available.

*Allorhizobium* (de Lajudie et al., 1998) and *Pararhizobium* (Mousavi et al., 2015) appeared as polyphyletic in GBDP and 16S rRNA gene trees (Figure 5 and Supplementary File S2) because several species are mixed up with representatives of *Rhizobium* (Frank, 1889). When *Allorhizobium* was proposed, a tree with rather low support at backbone was presented which did not yield well-defined relationships between the genera. Later studies of the group presented much better resolved multi-gene analyses but restricted the taxon sampling to rhizobial and agrobacterial strains (Mousavi et al., 2015). The CCT included a well-supported clade including the type species of *Allorhizobium*, *A. undicola*, the other *Allorhizobium* species except for *A. oryzae*, as well as *Rhizobium oryziradicis* (Zhao et al., 2017), and *R. taibaishanense* (Yao et al., 2012). *Rhizobium* (Frank, 1889; Young et al., 2001) was shown as non-monophyletic for various reasons in the GBDP tree (Figure 5) and the CCT, including distantly placed species such as *R. arenae* (Zhang S. et al., 2017), *R. flavum* (Gu et al., 2014), *R. gei* (Shi et al., 2016), *R. marinum* (Liu Y. et al., 2015), *R. selenitireducens* (Hunter et al., 2007), *R. naphthalenivorans* (Kaiya et al., 2012), *R. subbaraonis* (Ramana C.V. et al., 2013) which were placed apart from the well-supported core *Rhizobium* clade that contained the type species of the genus.

The taxonomic literature disagreed on whether a broad concept of *Rhizobium*, i.e., including genera such as *Allorhizobium* and *Agrobacterium* (Conn, 1942), should be preferred (Young et al., 2001, 2003) or whether *Rhizobium* should be split into various genera (Farrand et al., 2003; Mousavi et al., 2015). Apparently distinct authors of new species names followed either one or the other of the two competing concepts (Willems, 2006), thus leading to the considerable mix-up of the species of *Rhizobium* (Figure 5). Given the overall genomic divergence of the group revealed here it appeared advisable to follow a narrow concept for *Rhizobium*. Further dissecting *Rhizobium* does not imply introducing many new genus names because names for the respective clades were already proposed in the literature. For instance, *R. oryziradicis* and *R. taibaishanense* can be assigned to the already existing genus *Allorhizobium* (Figure 5 and Supplementary File S2), which is not in disagreement with their phenotype (Supplementary Table S1) while the group is even supported in the gene-content analysis (Supplementary File S2). The new combination *Allorhizobium taibaishanense* was already proposed (Mousavi et al., 2015) but the name does not appear to be validly published.

On a similar vein, *R. subbaraonis* (Ramana C.V. et al., 2013) should be assigned to *Mycoplana* (Urakami et al., 1990; Gray and Thornton, 1928) whereas the phylogenetically relatively isolated *Pararhizobium haloflavum* (Shen et al., 2018) should be placed into a genus of its own (Figure 5, Supplementary File S2, and Supplementary Table S1). The inclusion of *Rhizobium subbaraonis* does not cause a need for the emendation of *Mycoplana* (Supplementary Table S1). The original description of *R. subbaraonis* did not include *Mycoplana*, whereas only a limited total number of species was considered when *P. haloflavum* was originally proposed.

The taxonomy of deviating species of *Allorhizobium* such as *A. borbori, A. oryzae* and *A. pseudoryzae* as well as of the deviating *Pararhizobium* species *P. capsulatum* (Mousavi et al., 2015) cannot currently be improved because of low resolution in the comprehensive 16S rRNA gene trees (Supplementary File S2). Similarly, while *Rhizobium* species such as *R. smilacinae* (Zhang et al., 2014b), *R. cellulosilyticum* (García-Fraile et al., 2007), *R. zeae* (Celador-lera et al., 2017), *R. wenxiniae* (Gao et al., 2017) and *R. yantingense* (Chen W. et al., 2015) were nested within *Neorhizobium* (Mousavi et al., 2014), the clade containing both *Neorhizobium* and *Pseudorhizobium* (Kimes et al., 2015) was particularly poorly supported (Supplementary File S2). While *Rhizobium* species such as *R. naphthalenivorans* and R. *selenitireducens* could possibly be placed in *Ciceribacter* (Kathiravan et al., 2013) and others such as *R. arenae* and *R. gei* could potentially be included in *Pararhizobium*, these arrangements were also poorly resolved. Therefore it would currently be difficult to infer taxonomic conclusions. We suppose that a more satisfying classification of these genera can be obtained once more genome sequences become available. As an exception, *Rhizobium vignae* (Ren et al., 2011), which was placed as sister group of *N. galegae* (Mousavi et al., 2014) with strong support, can be assigned to *Neorhizobium*. While they are closely related, our dDDH results (41.4% similarity) values indicate that *R. vignae* and *N. galegae* are independent species. Therefore, we propose to include *R. vignae* in *Neorhizobium.* This proposal is not contradicted by phenotypic characteristics and the transfer of *R. vignae* does not cause a need for the emendation of *Neorhizobium* (Supplementary Table S1). The removal of *R. oryziradicis*, *R. taibaishanense*, *R. subbaraonis* and *R. vignae* does neither cause a need for the emendation of *Rhizobium* (Frank, 1889).

### *Rhodobacterales* Genera

**FIGURE 9:**
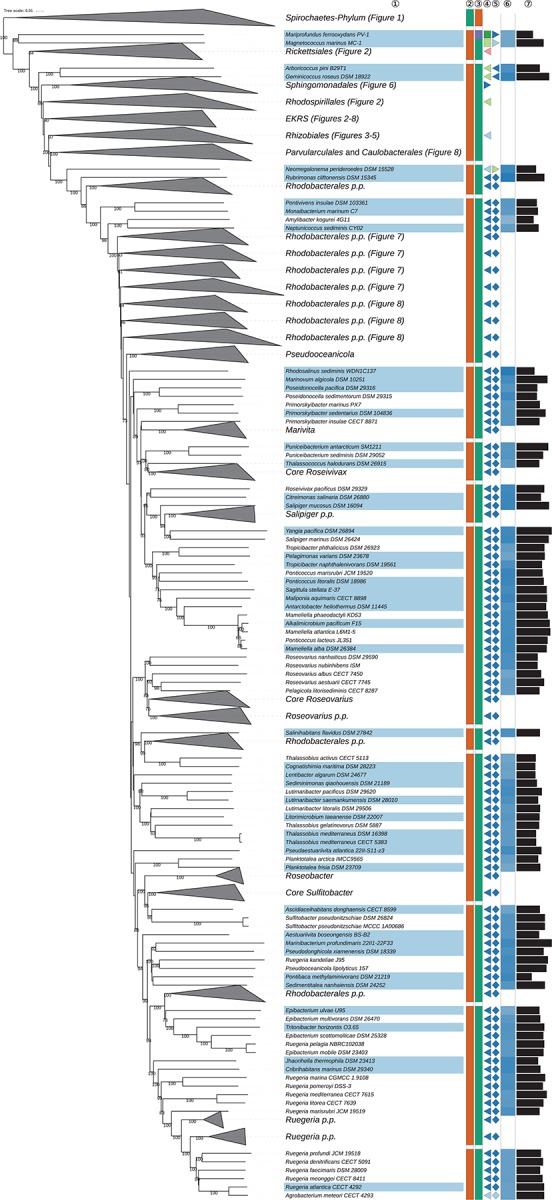
Eighth part of the GBDP tree shown in Figure 1, focussing on parts of *Rhodobacterales*. The clade labeled EKRS comprises the orders *Emcibacterales, Kordiimonadales, Rhodothalassiales*, and *Sneathiellales*, details for which are shown in other figures. Tip labels with light-blue background indicate type species of genera, colors and symbols to the right of the tips indicate, from left to right, phylum, class, order, and family; for details and abbreviations see Figure 1. The blue color gradient indicates the G+C content as calculated from the genome sequences, followed by black bars indicating the (approximate) genome size in base pairs.

*Rhodobacterales* has so far been a monotypic order that only included *Rhodobacteraceae*. As mentioned above, this family as originally proposed is illegitimate because the family included *Hyphomonas* (Moore et al., 1984), the type genus of *Hyphomonadaceae* (Lee et al., 2005), which has priority. Except for the genera taxonomically assigned to *Rhodobacterales* but phylogenetically placed in *Rhizobiales* (*Hyphomicrobiales*), which were treated above, *Rhodobacteraceae* was monophyletic. However, whereas most of its genera were also shown as monophyletic in our analyses (Figures 7–9), some were shown to be in need of a taxonomic revision.

*Roseivivax* (Suzuki et al., 1999a; Park et al., 2010; Chen M.-H. et al., 2012) was shown as non-monophyletic in the GBDP tree and the CCT (Figures 7, 9 and Supplementary File S2) because *R. roseus* (Zhang et al., 2014c) formed a clade (Figure 7) together with *Tranquillimonas alkanivorans* (Harwati et al., 2008) while *R. pacificus* (Wu et al., 2013) appeared (Figure 9) as sister group of *Citreimonas salinaria* (Choi and Cho, 2006). When *R. roseus* was originally proposed, it formed a group with *R. pacificus* which received only poor support in 16S rRNA gene trees, much like its relationship to core *Roseivivax*. Similarly, the phylogenetic position of *T. alkanivorans* and *C. salinaria* remained unresolved in these trees. The isolated position of the species in the 16S rRNA gene and genomic trees calls for the proposal of a new genus to accommodate *R. pacificus.* In addition, we propose to include *R. roseus* in *Tranquillimonas*. This is not contradicted by the phenotypic characteristics of these taxa (Supplementary Table S1) even though the gene-content analysis does not lend support (while core *Roseivivax* is reasonably supported; Supplementary File S2). The removal of *R. roseus* and *R. pacificus* does not cause a need for the emendation of *Roseivivax* (Supplementary Table S1).

*Defluviimonas* (Foesel et al., 2011; Math et al., 2013) appeared as paraphyletic in the GBDP tree (Figure 7) because particularly *Defluviimonas alba* (Pan et al., 2015) but also *D. indica* (Jiang et al., 2014) were placed apart from the type species of *Defluviimonas, D. denitrificans*, which appeared more closely related to the type species of *Albidovulum*, *A. inexpectatum* (Albuquerque et al., 2002) whereas *Albidovulum xiamenense* (Yin et al., 2012) was in turn placed apart from its type species. In the original proposal of *A. xiamenense*, only weak support was obtained for the monophyly of *Albidovulum*. When *D. indica* was proposed, 90% support was obtained for the monophyly of *Defluviimonas* in a 16S rRNA gene analysis based on the neighbor-joining algorithm and a simplistic evolutionary model. We could not reproduce this finding here based on our ML analysis with a much larger taxon sampling (Supplementary File S2) which, even when unconstrained, placed *D. indica* as sister group of the clade comprising *A. inexpectatum* and core *Defluviimonas*. In the 16S rRNA gene trees (Supplementary File S2) *D. alba* was placed as sister group of *Frigidibacter albus* (Li and Zhou, 2015). When *D. alba* was proposed, no support for the monophyly of *Defluviimonas* was obtained, as the phylogenetic position of *D. alba* remained uncertain; *Albidovulum* was not considered. *D. alba* and *F. albus* show similar phenotypic characteristics (Supplementary Table S1). Therefore, one could propose to include *D. alba* in *Frigidibacter*. However, the 16S rRNA gene similarity between the two type strains is 99.79% when calculated using the recommended settings, which indicates that DNA:DNA hybridization should be carried out to rule out that the strains are conspecific (Meier-Kolthoff et al., 2013b). In contrast, we conclude that a new genus should be proposed to accommodate *D. indica*, which is also in accordance with the gene-content analysis (Supplementary File S2) as it yielded strong support for the remaining clade after removal of *D. indica*. Including, *D. aestuarii*, *D. aquaemixtae*, *D. denitrificans*, and *D. nitratireducens* in *Albidovulum* is a phylogenetically obvious solution but would render the remaining species of the genus, *D. alba* and *D. pyrenivorans*, illegitimate, whose phylogenetic position is still uncertain. In contrast, its isolated phylogenetic position indicates that *Albidovulum xiamenense* can safely be placed into a genus of its own, which is not precluded by its phenotype. The removal of *A. xiamenense* does not cause a need for the emendation of *Albidovulum* (Supplementary Table S1).

*Gemmobacter* (Rothe et al., 1987; Chen W.-M. et al., 2013) was shown as polyphyletic in the GBDP tree (Figure 7) because *G. nectariphilus* (Tanaka et al., 2004; Chen W.-M. et al., 2013) and *G. megaterium* (Liu J.-J. et al., 2014) were placed in a distant position relative to the type species, *G. aquatilis*. In addition, in the CCT *G. intermedius* (Kämpfer et al., 2015b) and *G. straminiformis* (Kang et al., 2017) formed an unsupported group together with *G. nectariphilus* and *G. megaterium* (Supplementary File S2). When these *Gemmobacter* species were proposed the monophyly of the genus was already unsupported in 16S rRNA gene trees. We did not detect an obvious synapomorphy for the genus as currently circumscribed (Supplementary Table S1). Therefore, a new genus is proposed to accommodate *G. megaterium* and *G. nectariphilus*; *G. intermedius*, and *G. straminiformis* should also be assigned to this genus once their genome sequences confirm their position, which is currently uncertain in 16S rRNA gene trees. The removal of *G. megaterium* and *G. nectariphilus* does not cause a need for the emendation of *Gemmobacter* (Supplementary Table S1). The two resulting genera received high support in the gene-content analysis (Supplementary File S2).

*Pseudorhodobacter* (Uchino et al., 2002; Jung et al., 2012a; Chen C.-X. et al., 2013; Lee et al., 2013, 2016) appeared as paraphyletic in the GBDP tree (Figure 7) because *P. psychrotolerans* (Lee et al., 2016) was placed as sister group of *Rhodobacter blasticus* and *Tabrizicola.* In the CCT and UCT, *P. aquaticus* (Li A.-H. et al., 2016), *P. collinsensis* (Zhang et al., 2016), *P. psychrotolerans* (Lee et al., 2016) and *P. sinensis* (Li A.-H. et al., 2016) formed a reasonably well supported clade separate from core *Pseudorhodobacter* (Supplementary File S2) and showed similar phenotypic characteristics (Supplementary Table S1). The descriptions of these species were accompanied by poorly resolved 16S rRNA gene trees which did not clearly indicate the monophyly of *Pseudorhodobacter*. Given the results presented here, it is proposed to place these deviating *Pseudorhodobacter* species in a separate genus.

*Rhodobacter* (Imhoff et al., 1984; Srinivas et al., 2007b; Wang et al., 2014) was shown to be non-monophyletic in the GBDP tree with strong support (Figure 7) because a clade containing *R. veldkampii* (Hansen and Imhoff, 1985) and *R. vinaykumarii* (Srinivas et al., 2007b) was placed apart from the clade containing the type species of the genus. In addition, *R. azotoformans* (Hiraishi et al., 1996), *R. johrii* (Girija et al., 2010), *R. megalophilus* (Arunasri et al., 2008), *R. ovatus* (Srinivas et al., 2008) and *R. sphaeroides* (van Niel, 1944; Imhoff et al., 1984) were placed apart from each of these two clades and formed the sister group of *Cereibacter* (Suresh et al., 2015) instead. Finally, *R. blasticus* (Kawasaki et al., 1993) appeared as sister group of *Tabrizicola aquatica* (Tarhriz et al., 2013). *Rhodobacter* already appeared non-monophyletic in the 16S rRNA gene trees presented in recent taxonomic studies such as the one that proposed *Tabrizicola* although phylogenetic resolution remained limited. The five *Rhodobacter* species placed adjacent to *Cereibacter* are phenotypically in agreement with this genus (Supplementary Table S1), which was also supported by the gene-content analysis (Supplementary File S2). Assigning these deviating *Rhodobacter* species to *Cereibacter* is more conservative than establishing a separate genus. Based on dDDH values, *R. megalophilus* is a later heterotypic synonym of *R. sphaeroides* (Table 1). Consequently, we propose to place *R. azotoformans*, *R. johrii*, *R. ovatus*, and *R. sphaeroides* in *Cereibacter*. Given their relatively isolated phylogenetic position a separate genus is proposed to accommodate *R. vinaykumarii* and *R. veldkampii*, which is not in conflict with their phenotype (Supplementary Table S1) even though the group is not supported by the gene-content analysis. Similarly, *R. blasticus* can be placed in *Tabrizicola*; *Xinfangfangia* (Hu et al., 2018) was also shown to be closely related to this group in the 16S rRNA gene trees (Supplementary File S2) but *Tabrizicola* has priority. The removal of *R. azotoformans, R. johrii, R. ovatus, R. sphaeroides, R. vinaykumarii, R. veldkampii* and *R. blasticus* does not cause a need for the emendation of *Rhodobacter* (Supplementary Table S1).

*Paracoccus* (Davis et al., 1969; Ludwig et al., 1993; Liu et al., 2008) appeared as paraphyletic in the GBDP tree (Figure 7) because *Methylarcula marina* (Doronina et al., 2000) was placed as sister group of *Paracoccus saliphilus* (Wang Y.-X. et al., 2009). In the CCT, *Methylarcula terricola* (Doronina et al., 2000) was also nested within the genus *Paracoccus*. When *M. marina and M. terricola* were proposed, a 16S rRNA gene phylogenetic analysis showed strong support for a clade comprising *Methylarcula* and *Paracoccus* but only weak support for the monophyly of *Paracoccus* to the exclusion of *Methylarcula.* The known phenotypic features of the two genera also fit excellently to each other (Supplementary Table S1). Thus, we conclude that *Methylarcula* should be included in *Paracoccus*, which has priority. However, since the types strains of both *Methylarcula* species appear to have been deposited in a single culture collection only, alternative species names cannot currently be proposed (Parker et al., 2019).

*Actibacterium* (Lucena et al., 2012; Guo et al., 2017) appeared as paraphyletic in the GBDP tree (Figure 8) because *Confluentimicrobium lipolyticum* (Park et al., 2014d) was placed as sister group of *Actibacterium ureilyticum* (Lin et al., 2016). In the CCT, in addition to *C. lipolyticum, C. naphthalenivorans* (Jeong et al., 2015) also was placed within *Actibacterium* (Supplementary File S2). When *C. lipolyticum* was proposed, its phylogenetic position could not fully be resolved by 16S rRNA gene analysis, as it appeared external to *Actibacterium* but with low support. *C. lipolyticum* and *C. naphthalenivorans* show phenotypic characteristics similar to those of *Actibacterium* (Supplementary Table S1). Therefore, it is proposed to assign *C. lipolyticum* and *C. naphthalenivorans* to *Actibacterium*, which has priority.

*Celeribacter* (Ivanova et al., 2010; Lee et al., 2012) was shown as paraphyletic in the GBDP tree (Figure 8) because *C. manganoxidans* (Wang L. et al., 2015) was placed in a position relatively distant to a clade harboring *Pacificibacter marinus* (Park et al., 2014c) together with core *Celeribacter* including the type species, *C. marinus* (Baek et al., 2014). In the CCT and UCT, *C. manganoxidans* was also placed apart from the other *Celeribacter* species without any obvious phylogenetic affiliation to another genus (Supplementary File S2). When *C. manganoxidans* was proposed, its phylogenetic placement was already only poorly supported. Although *C. manganoxidans* and *Celeribacter* do not display consistent phenotypic differences from each other (Supplementary Table S1), the phylogenomic results coupled with the lack of phylogenetic evidence to the contrary indicate that a new genus should be proposed to accommodate *C. manganoxidans.*

*Celeribacter* also appeared as paraphyletic in the CCT because *Vadicella arenosi* (Romanenko et al., 2011c) was placed within this genus with high support. When *Vadicella* was proposed the only phylogenetic evidence was a 16S rRNA gene tree with low backbone support. In addition at that time only *Celeribacter neptunius* (Ivanova et al., 2010) was considered for comparison in the 16S rRNA gene tree. Taking all other *Celeribacter* species and the phenotype of these species (Supplementary File S1) into account, the preferred solution is to include *Vadicella* in *Celeribacter.*

*Tropicimonas* (Harwati et al., 2009a; Oh et al., 2012) was shown as paraphyletic in GBDP tree (Figure 8) because *Pseudoruegeria marinistellae* (Zhang Y. et al., 2017) was placed as sister group of type species of *Tropicimonas*, *T. isoalkanivorans* (Harwati et al., 2009a). In addition, in CCT *Pseudoruegeria aestuarii* was also nested within the *Tropicimonas* clade (Supplementary File S2). When *P. marinistellae* was proposed, *T. isoalkanivorans* was not included in the phylogenetic analysis. Furthermore, species such as *P. aestuarii* (Cha et al., 2016), *P. haliotis* (Hyun et al., 2013b), *P. lutimaris* (Jung et al., 2010), and *P. sabulilitoris* (Park et al., 2014a) were also apart from the clade harboring the type species of the genus. However, the lack of a genome sequence for the type species of *Pseudoruegeria, P. aquimaris*, currently precludes re-classifications of *Pseudoruegeria* species, particularly because *Pseudoruegeria* has priority over *Tropicimonas*. In the CCT, *Tropicimonas arenosa* (Oh et al., 2016) was placed as sister group of *Oceanicola litoreus* (Park et al., 2013c) but supported with a low bootstrap value (Supplementary File S2). Due to the lack the genome sequences of *T. arenosa*, *w*e refrain from taxonomic consequences for this species.

*Oceanicola* (Cho and Giovannoni, 2004) appeared as polyphyletic in the GBDP tree and CCT (Figure 8 and Supplementary File S2) because *Oceanicola granulosus* (Cho and Giovannoni, 2004) was placed as sister group of *Roseisalinus antarcticus* (Labrenz et al., 2005) with strong support (even in the gene-content analysis) whereas *Oceanicola litoreus* (Park et al., 2013c) was placed in a distinct clade as sister group of *Celeribacter manganoxidans*. While this clade had only moderate support in the GBDP tree, the tree clearly indicated that *O. litoreus* does not form a clade together with *O. granulosus*, which is the type species of the genus. Similarly, *O. litoreus* was placed distantly to *O. granulosus* in the CCT, in that case close to *Tropicimonas arenosa* with low support (Supplementary File S2). When *O. litoreus* was proposed, it was placed as sister group of the other *Oceanicola* species with low support in a 16S rRNA gene tree. When *Roseisalinus antarcticus* was originally proposed, the presented tree displayed low support at the backbone, and the sampling did not include *Oceanicola*. The DSMZ nomenclature database indicates that a total of nine *Oceanicola* species had been proposed in the literature all except two were assigned to other genera in later studies. As revealed here it makes sense to taxonomically assign *O. litoreus* to a genus of its own, which is not precluded by its phenotype (Supplementary Table S1).

*Primorskyibacter* (Romanenko et al., 2011b) was shown as non-monophyletic in the GBDP tree (Figure 9) because *P. insulae* (Park et al., 2015a) occupied a relatively isolated position, distant from core *Primorskyibacter* including the type species, *P. sedentarius* (Romanenko et al., 2011b); this arrangement was also shown in the CCT (Supplementary File S1). When *P. insulae* was originally proposed (Park et al., 2015a), the analysis of the 16S rRNA gene yielded only low branch support for the monophyly of *Primorskyibacter*. As the sister-group relationship between *P. insulae* and *Marivita* (Hwang et al., 2009a) is not supported in the phylogenomic tree, *P. insulae* should rather be placed in a genus of its own, which is not contradicted by the phenotype (Supplementary Table 1).

*Salipiger* (Martínez-Cánovas et al., 2004) appeared as paraphyletic in GBDP tree (Figure 9) because *Yangia pacifica* (Dai et al., 2006) was placed as sister group of *S. marinus* (Wirth and Whitman, 2018) with strong support. The taxonomic history of the genera is characterized by a decrease of support in 16S rRNA gene trees with an increasing number of species. When *Yangia* was proposed, 96% support for a clade comprising *Salipiger* and *Roseivivax* to the exclusion of *Yangia* was obtained. However, when *Citreicella marina* was proposed (Lai et al., 2011a), which was later on included in *Salipiger*, 98% support for a sister-group relationship with *C. thiooxidans* was obtained while *Yangia* was shown as sister group of this clade with low support. Because the type species of *Salipiger*, *S. mucosus*, was branching first within the *Salipiger–Yangia* clade in the GBDP tree, generating separate genera would imply reclassifying all *Salipiger* species except for the type species. For this reason, it is taxonomically more conservative to include *Yangia* in *Salipiger*, which is supported by the lack of significant phenotypic differences between the two genera (Supplementary Table S1), while the gene-content analysis is inconclusive (Supplementary File S2). *Paraphaeobacter pallidus* (Cai et al., 2017) may also have to be placed in *Salipiger* according to the 16S rRNA gene analyses. But due to low support and since the genome sequence of *P. pallidus* is not yet available, we refrain from taxonomic consequences for this species.

*Tropicibacter* (Harwati et al., 2009b) formed a paraphyletic group in the GBDP tree and the CCT (Figure 9 and Supplementary File S2) because *T. phthalicicus* (Iwaki et al., 2012) formed the sister group of *Pelagimonas* with strong support. When *T. phthalicicus* was proposed, the 16S rRNA gene tree showed only poor support for a sister-group relationship between *T. phthalicicus* and the type species, *T. naphthalenivorans*. While the gene-content analysis is inconclusive in this respect, assigning *T. phthalicicus* to *Pelagimonas* is certainly the taxonomically most conservative solution to solve the discrepancy. This is not precluded by the known major phenotypic features of these taxa, as the only known difference is motility in *T. phthalicicus* (Supplementary Table 1). The removal of T. *phthalicicus* does not cause a need for the emendation of *Tropicibacter* (Supplementary Table S1).

*Roseovarius* (Labrenz et al., 1999) was shown as paraphyletic in the GBDP tree and in the CCT (Figure 9 and Supplementary File S2) because *Pelagicola litorisediminis* (Park et al., 2013a) was nested within *Roseovarius* with a high bootstrap value. When *P. litorisediminis* was proposed, a phylogenetic analysis was presented that was unresolved at the backbone; in particular, the monophyly of *Roseovarius* was not supported at all. *P. litorisediminis* and *Roseovarius* show similar phenotypic characteristics (Supplementary Table S1). Consequently, we propose to include *P. litorisediminis* in *Roseovarius*, which is the taxonomically most conservative solution. The clade obtains some support even in the gene-content analysis (Supplementary File S2), and the inclusion of *P. litorisediminis* does not cause a need for the emendation of *Roseovarius* (Supplementary Table S1).

*Lutimaribacter* (Yoon et al., 2009) appeared as non-monophyletic in the GBDP tree (Figure 9) because *Lutimaribacter litoralis* (Iwaki et al., 2013) was placed within a strongly supported clade containing *Litorimicrobium taeanense* (Jin et al., 2011) and core *Thalassobius* (Arahal et al., 2005) to the exclusion of the type species of *Lutimaribacter*. The original proposal of *Lutimaribacter litoralis* was accompanied by a 16S rRNA gene analysis with 90% support for a clade comprising *Lutimaribacter* together with *Oceanicola pacificus*. When *Litorimicrobium* was proposed (Jin et al., 2011), the presented tree displayed low support at backbone and the relationships between the genera remained ambiguous. This problem was already evident in the study that proposed *Thalassobius.* Given the phylogenomic results and the lack of evidence to the contrary in the 16S rRNA gene data, it is proposed to include *Lutimaribacter litoralis* and *Litorimicrobium taeanense* in *Thalassobius*, which has priority. Even though the clade obtains no support in the gene-content analysis, this solution is not precluded by the known phenotypic features (Supplementary Table S1). The removal of *Lutimaribacter litoralis* does not cause a need for the emendation of *Lutimaribacter* (Supplementary Table S1).

*Thalassobius* (Arahal et al., 2005) was shown as non-monophyletic in the GBDP and 16S rRNA gene trees (Figure 9 and Supplementary File S2) because *T. activus* appeared more closely related to *Cognatishimia* (Wirth and Whitman, 2018) than to the type species, *T. mediterraneus*. When *T. activus* was proposed (Pujalte et al., 2018), it already did not form a clade together with the type species of the genus in a phylogenetic analysis of the 16S rRNA gene. Given their close phylogenetic relationship, which is strongly supported in the GBDP tree albeit unresolved in the gene-content analysis, it is proposed to include *T. activus* in *Cognatishimia*, which is not precluded by the known phenotypic features (Supplementary Table S1); among the major phenotypic features, the sole difference appears to be the lack of flagella in *T. activus*. *Thalassobius activus* was placed in *Cognatishimia* (Arahal et al., 2019) while the current study was under revision, hence no further taxonomic proposal needs to be made although it appears advisable to provide an emended description of *Cognatishimia*.

*Sulfitobacter* (Sorokin, 1995) was shown as a paraphyletic group in the GBDP tree and the CCT (Figure 9 and Supplementary File S2) because *S. pseudonitzschiae* (Hong et al., 2015) was shown to be more closely related to *Ascidiaceihabitans* (Kim et al., 2016b) than to the type species of *Sulfitobacter*, albeit with low support. The original description of *S. pseudonitzschiae* showed a 16S rRNA gene in which *Sulfitobacter* did not appear monophyletic. Two *Roseobacter* species were even shown as more closely related as *S. pseudonitzschiae* to the type species of *Sulfitobacter*, *S. pontiacus*, with strong support, whereas *Ascidiaceihabitans* could not be considered. The original description of *Ascidiaceihabitans* in turn did not consider *S. pseudonitzschiae*. The CCT indicated *Pseudoseohaeicola* (Park et al., 2015b) as sister group of *S. pseudonitzschiae* to the exclusion of *Ascidiaceihabitans* but since the support was only moderate we here refrain from taxonomic proposals for *Pseudoseohaeicola*. The issue should be revisited once the genome of the type strain of *Pseudoseohaeicola caenipelagi* becomes available. Similarly, as *Ascidiaceihabitans* has priority over *Pseudoseohaeicola*, it would be safe to place *S. pseudonitzschiae* in *Ascidiaceihabitans*, but resolution is low even in the GBDP tree.

*Pseudooceanicola* (Lai et al., 2015) formed a polyphyletic group in the GBDP tree (Figure 9) as *P. lipolyticus* (Huang et al., 2018) appeared as the sister taxon of *Ruegeria kandeliae* (Zhang L. et al., 2018) with strong support. *R. kandeliae* was in turn phylogenetically located apart from core *Ruegeria*. The CCT showed the same relationships while the UCT was unresolved (Supplementary File S2). Given their relatively isolated position in the genome-based phylogeny, we would propose to transfer *P. lipolyticus* and *R. kandeliae* to a single new genus, which is not contradicted by their phenotype (Supplementary Table S1). While not supported by the gene-content analysis (Supplementary File S2), this solution is certainly more conservative than assigning them to two distinct genera. The removal of *R. kandeliae* does not cause a need for the emendation of *Ruegeria* (Uchino et al., 1998; Martens et al., 2006; Yi et al., 2007). In the case of *P. lipolyticus*, however, we observed a significant discrepancy between the 16S rRNA gene tree and the GBDP tree (Supplementary File S2). While the remainder of the used GenBank genome sequence did not show signs of contamination, the rRNA genes, which covered almost the complete contig NZ_PGTB01000197, yielded a distinct phylogenetic location. For this reason, we cannot rule out that the protein-coding genes of the genome sequence do not, in contrast to the 16S rRNA gene, originate from *P. lipolyticus*. Hence, we only propose a new genus for *Ruegeria kandeliae.*

*Epibacterium* (Penesyan et al., 2013) was shown as a paraphyletic group in the GBDP tree and the CCT (Figure 9 and Supplementary File S2) because all *Epibacterium* species except for the type species, *E. ulvae*, formed a strongly supported clade together with *Tritonibacter* (Klotz et al., 2018) and *Ruegeria pelagia* (Lee et al., 2007c). *R. pelagia* in turn appeared only distantly related to the type species of *Ruegeria*, *R. atlantica*. The taxonomically most conservative solution to this discrepancy between phylogeny and classification is to assign all deviating *Epibacterium* species – including *E. scottomollicae* (Vandecandelaere et al., 2008a; Wirth and Whitman, 2018) *–* to *Tritonibacter*, which is not precluded by their major phenotypic features (Supplementary Table S1) even though the gene-content analysis is inconclusive (Supplementary File S2). The inclusion of *E. scottomollicae* does not cause a need for the emendation of *Tritonibacter* (Supplementary Table S1).

*Agrobacterium* (Conn, 1942; Sawada et al., 1993) appeared as polyphyletic in the GBDP and 16S rRNA gene trees (Figure 9 and Supplementary File S2) because *Agrobacterium meteori* (Rüger and Höfle, 1992) was placed as a sister group of *Ruegeria atlantica* (Rüger and Höfle, 1992; Uchino et al., 1998; Muramatsu et al., 2007; Yi et al., 2007; Vandecandelaere et al., 2008a) with high support. *A. meteori* was originally proposed on basis of phenotypic characteristics without taking into account the 16S rRNA gene sequence as phylogenetic marker. The phenotype of *A. meteori* is quite similar to the one of *Ruegeria atlantica* (Supplementary Table S1), and according to an earlier study (Uchino et al., 1998) *A. meteori* is a later heterotypic synonym of *R. atlantica* (=*Agrobacterium atlanticum*). However, the dDDH value between their genome sequences was lower than the species boundary of 70% (Table 1). Accordingly, it is proposed to include *A. meteori* in *Ruegeria* as *R. meteori.* The removal of *A. meteori* does not cause a need for the emendation of *Agrobacterium* (Supplementary Table S1) and the inclusion of *A. meteori* does not cause a need for the emendation of *Ruegeria* (Uchino et al., 1998; Martens et al., 2006; Yi et al., 2007).

*Aminobacter* (Urakami et al., 1992; Kämpfer et al., 2002a) appeared as paraphyletic in the CCT (Supplementary File S2) because the monotypic genus *Carbophilus* (Meyer et al., 1993) was nested within *Aminobacter* with strong support. When *Carbophilus* was originally proposed *Aminobacter* was not taken into account. As the two share most phenotypic characteristics and mostly differ regarding their minor fatty acids (Supplementary Table S1) we propose to transfer *Carbophilus* to *Aminobacter*, which has priority; this is also the taxonomically most conservative solution.

### *Sphingomonadales* Families

*Sphingomonadales* appeared to be a monophyletic order of *Alphaproteobacteria* in our analyses (Figure 6), which is in agreement with the presence of sphingolipids (Supplementary Table S1 and Supplementary File S2), a likely apomorphy of the group. The arrangement of the order into families seemed to be in need of a revision, however.

Within *Sphingomonadales, Sphingomonadaceae* (Kosako et al., 2000) appeared paraphyletic in the GBDP tree (Figure 6) because the clade comprising *Sandarakinorhabdus cyanobacteriorum* (Cai et al., 2018a), *S. limnophila* (Gich and Overmann, 2006; Kim M.C. et al., 2016), *Sphingosinicella microcystinivorans* (Maruyama et al., 2006; Geueke et al., 2007) and *Pacificimonas flava* (Liu K. et al., 2014), which appeared as sister group of the remaining taxa. These genera should better be placed in a separate family, which is supported by high overall genomic divergence within *Sphingomonadales* (Figure 6 and Supplementary File S2). The gene-content analysis did not resolve the backbone of the *Sphingomonadales* tree but did not yield significant conflict either. In published 16S rRNA gene trees *Sphingosinicella* was placed with only low support in *Sphingomonadaceae* (Maruyama et al., 2006). The original descriptions of *Sandarakinorhabdus* (Gich and Overmann, 2006) and *Pacificimonas* (Liu K. et al., 2014) were also accompanied by 16S rRNA gene trees with low support. In the CCT the additional genera *Polymorphobacter* (Fukuda et al., 2014), *Sandaracinobacter* (Yurkov et al., 2017) and *Sphingoaurantiacus* (Kim M.C. et al., 2016) appeared intermixed with the genera represented by genome sequences in the same clade and thus should tentatively also be assigned to the new family. The establishment of a new family for these genera is not in conflict with their phenotype (Supplementary Table S1).

The second conflict within *Sphingomonadales* was due to the fact that *Erythrobacteraceae* (Lee et al., 2005; Xu X.-W. et al., 2009) was nested within *Sphingomonadaceae* with high support in the GBDP tree (Figure 6 and Supplementary File S2). In particular, *Novosphingobium* (Takeuchi et al., 2001) appeared intermixed with *Erythrobacteraceae*. *Sphingomonadaceae* originally encompassed the genera currently placed in *Erythrobacteraceae* such as *Erythrobacter* and *Porphyrobacter* (Kosako et al., 2000) before *Erythrobacteraceae* were proposed (Lee et al., 2005) on the basis of the analysis of 16S rRNA gene and chemotaxonomic data. The high branch support obtained in that study for the monophyly of both *Erythrobacteraceae* and *Sphingomonadaceae sensu stricto* could not be confirmed by our analyses, which are based on a much larger taxon sampling (Supplementary File S2). The presence of pigments (including bacteriochlorophyll α) in *Erythrobacteraceae* which are absent in genera such as *Sphingomonas* was also used as argument for the separation of the two families (Lee et al., 2005). However, a single character with two character states cannot properly be used to separate two taxa because this character would yield an apomorphy for at most one of the two taxa (Nouioui et al., 2018). The sizeable overall genomic divergence within *Sphingomonadales* (Figure 6 and Supplementary File S2) argues against placing all of its genera into a single family. For this reason, we propose to include *Novosphingobium* in *Erythrobacteraceae.* Considering that only apomorphies can be used to justify a taxon (Hennig, 1965; Wiley and Lieberman, 2011), this proposal did not appear to be in conflict with the phenotype of the involved taxa (Supplementary Table S1).

Additionally the positioning of a clade comprised of *Zymomonas mobilis* (Kluyver and van Niel, 1936) including *Zymomonas mobilis* subsp. *pomaceae* (De Ley and Swings, 1976; Coton et al., 2006) caused conflict regarding *Sphingomonadaceae* in the GBDP tree (Figure 6). *Zymomonas* (Kluyver and van Niel, 1936) also formed a branch isolated from the remaining *Sphingomonadaceae* in the CCT (Supplementary File S2). While the resolution of the backbone within *Sphingomonadales* was partially low in the GBDP tree, an additional supermatrix analysis (Supplementary File S2) confirmed the placement of *Zymomonas* apart from the type species of the family, *Sphingomonas*. In contrast to most *Sphingomonadaceae, Zymomonas* was described as facultatively anaerobic (Supplementary File S2). While this deviation, much like the lower G+C content and genome size, is probably an autopomorphy of *Zymomonas*, there is no obvious phenotypic argument for placing the genus within *Sphingomonadaceae* as previously suggested (Kosako et al., 2000). Therefore we propose to transfer *Zymomonas* to a family of its own.

### *Sphingomonadales* Genera

Within *Sphingomonadales*, *Novosphingobium* (Takeuchi et al., 2001) appeared as non-monophyletic in the GBDP tree (Figure 6) as well as in the CCT (Supplementary File S2) because *N. tardaugens* (Fujii et al., 2003) was placed in a relatively isolated position with respect to core *Novosphingobium*, more closely related to *Altererythrobacter*, *Erythrobacter*, and *Porphyrobacter*. When *N. tardaugens* was proposed the reduced taxon sampling in the presented 16S rRNA gene tree did not allow for an assessment of the monophyly of the genus. Given the overall genomic divergence of *Erythrobacteraceae* (Lee et al., 2005; Xu X.-W. et al., 2009), into which we propose to assign *Novosphingobium* as explained above, solving the non-monophyly of the major genera of the family by merging these genera does not seem taxonomically advisable. For this reason, we suggest a new genus to accommodate *N. tardaugens*, which is not contradicted by its phenotype (Supplementary Table S1). *N. tardaugens* also displayed a genome size more in accordance with the one found in other genera of *Erythrobacteraceae* than with the larger genomes of core *Novosphingobium*.

*Erythrobacter* (Shiba and Smidu, 1982; Subhash et al., 2013) was shown as non-monophyletic in the GBDP tree (Figure 6) because species such as *E. gangjinensis* (Lee et al., 2010), *E. luteus* (Lei et al., 2015), *E. atlanticus* (Zhuang et al., 2015), *E. marinus* (Jung et al., 2012b), *E. seohaensis* (Yoon et al., 2005b) and *E. nanhaisediminis* (Xu et al., 2010) were placed apart from the type species of *Erythrobacter*, *E. longus* (Shiba and Smidu, 1982) causing *Altererythrobacter* (Kwon et al., 2007; Xue et al., 2012, 2016) to appear intermixed with *Erythrobacter.* Moreover, *E. longus* was placed more closely to *Porphyrobacter* (Fuerst et al., 1993; Coil et al., 2015) than to the majority of the *Erythrobacter* species. *Porphyrobacter* appeared as polyphyletic in the GBDP tree (Figure 6) because *P. mercurialis* (Coil et al., 2015) was placed apart from the remaining *Porphyrobacter* specie*s.* Additionally, the single representative of *Qipengyuania, Q. sediminis* (Feng et al., 2015), was nested in the CCT within *Altererythrobacter* but with low support as in the original publication. *Blastomonas marina* (Meng et al., 2017) also was nested in the CCT within *Altererythrobacter.* These taxonomic problems were already observed by other authors as *Erythrobacter, Altererythrobacter*, and *Porphyrobacter* appeared intermixed in 16S rRNA gene phylogenies (Coil et al., 2015). Thus, we are well aware of the fact that after this modification *Altererythrobacter*, *Erythrobacter* and *Porphyrobacter* would still be intermixed (Figure 6) but given the low support in the 16S rRNA gene analyses (Supplementary File S2) the taxonomy of the family should be revisited once more type-strain genome sequences are available. For instance, the genome sequence of the type strain of the type species of *Altererythrobacter, A. epoxidivorans* (Kwon et al., 2007), was not yet available at the time of writing. We thus refrain from proposing taxonomic changes for these three genera.

*Sphingosinicella* (Maruyama et al., 2006; Geueke et al., 2007; Yasir et al., 2010) was shown as polyphyletic in the GBDP tree and in the CCT (Figure 6 and Supplementary File S2) because *S. vermicomposti* (Yasir et al., 2010) was placed as sister group of *Sphingomonas indica* (Niharika et al., 2012) supported by a high bootstrap value. When *S. vermicomposti* was proposed, 98% support was obtained for the monophyly of *Sphingosinicella* in a 16S rRNA gene analysis based on the neighbor-joining algorithm and a simplistic evolutionary model. We could not reproduce this finding here based on our ML and MP analyses with a much larger taxon sampling (Supplementary File S2), as even the unconstrained analyses placed *S. vermicomposti* in a position quite distinct from core *Sphingosinicella*. The proposal of *Sphingomonas indica* was accompanied by a 16S rRNA gene tree that did not resolved the monophyly of *Sphingomonas* (Yabuuchi et al., 1990, 1999, 2001; Takeuchi et al., 1993, 2001; Busse et al., 2003; Chen et al., 2012). Given the overall genomic divergence of *Sphingomonadaceae*, solving the non-monophyly of the major genera of the family by merging these genera does not seem taxonomically advisable. Our phylogenetic analyses thus suggested that *S. vermicomposti* and *Sphingomonas indica* are best assigned to an independent genus. The two species also display similar phenotypic characteristics (Supplementary Table S1) even though the gene-content analysis did not provide support (Supplementary File S2). The removal of *Sphingomonas indica* does not cause a need for the emendation of *Sphingomonas* (Supplementary Table S1).

*Sphingopyxis* (Takeuchi et al., 2001; Baik et al., 2013) appeared as paraphyletic in the GBDP tree and in and in the CCT (Figure 6 and Supplementary File S2) because *Sphingopyxis baekryungensis* (Yoon et al., 2005a) was placed not within core *Sphingopyxis* but in a clade together with *Blastomonas* (Sly and Cahill, 1997) and two species of *Sphingorhabdus* (Jogler et al., 2013; Yang et al., 2017) with high support. Since these two *Sphingorhabdus* species were placed apart from the type species of the genus in the 16S rRNA gene analyses and because of the unclear assignment of *S. baekryungensis* to either *Blastomonas* or *Sphingorhabdus* we propose to place *S. baekryungensis* into a genus of its own, which is not precluded by its phenotype (Supplementary Table S1). When *S. baekryungensis* was originally proposed (Yoon et al., 2005a) a phylogenetic analysis with low taxon sampling was presented which lacked any support for the monophyly of *Sphingopyxis*; other evidence for the monophyly of the genus was not detected either. Taxonomic consequences for *Sphingorhabdus* cannot currently be drawn because of the lack of a genome sequence for the type strain of its type species, *S. planktonica*, and the lack of resolution in even the constrained 16S rRNA gene analyses.

We are aware of the fact that after this modification *Sphingomonas* still remained non-monophyletic because genera such as *Hephaestia* (Felföldi et al., 2014), *Rhizorhabdus* (Francis et al., 2014), and *Stakelama* (Chen C. et al., 2010) were nested within its range (Figure 6). Given the overall genomic divergence of the group, solving the non-monophyly by including these genera in *Sphingomonas* does not seem taxonomically advisable. When *Rhizorhabdus* was proposed, a couple of *Sphingomonas* species already appeared more closely related to it than to the type species of *Sphingomonas*, *S. paucimobilis* (Yabuuchi et al., 1990), but they were not included in the new genus. The type species of *Rhizorhabdus*, *R. argentea*, formed in the CCT and UCT a well-supported clade together with *R. dicambivorans* (Yao et al., 2016), *S. histidinilytica* (Nigam et al., 2010), *S. starnbergensis* (Chen H. et al., 2013) and *S. wittichii* (Yabuuchi et al., 2001; Kim M.C. et al., 2016). It thus makes sense to include these *Sphingomonas* species in *Rhizorhabdus*, which is not precluded by their phenotype (Supplementary Table S1). Given the otherwise low support in the 16S rRNA gene analyses (Supplementary File S2) we cannot propose analogous taxonomic consequences for *Hephaestia* and *Stakelama*. The taxonomy of *Sphingomonas* should be revisited once more type-strain genome sequences are available. The removal of *S. histidinilytica*, *S. starnbergensis* and *S. wittichii* does not cause a need for the emendation of *Sphingomonas* (Supplementary Table S1).

### *Kordiimonadales* and *Caulobacterales* Families

Only few discrepancies between taxonomic classification and phylogeny were observed in these relative small orders.

When *Kordiimonas* and with it the order *Kordiimonadales* was proposed (Kwon et al., 2005) no family to accommodate *Kordiimonas* was given. *Kordiimonadaceae* was later proposed (Xu et al., 2014) but has not been validly published yet. *Kordiimonas* formed a strongly supported clade together with *Eilatimonas* (Paramasivam et al., 2013) in the GBDP tree (Figure 8) and in the CCT (Supplementary File S2). When *Eilatimonas* was proposed, the genus already was shown in a 16S rRNA gene analysis as the sister group of *Kordiimonas* with strong support. Furthermore, *Temperatibacter* (Teramoto and Nishijima, 2014) of *Temperatibacteraceae* appeared as nested within *Kordiimonadaceae* in the CCT (Supplementary File S2), wherein the three genera formed a clade with strong support. The taxonomically most conservative solution is to include all these genera in an emended family *Temperatibacteraceae*, which is not precluded by their respective phenotypes (Supplementary Table S1).

Within *Caulobacterales, Hyphomonadaceae* (Lee et al., 2007d) appeared as paraphyletic in the GBDP tree because a clade comprising *Robiginitomaculum* to *Maricaulis* branched first, rendering core *Hyphomonadaceae* the sister group of *Caulobacteraceae* (Figure 8 and Supplementary File S2). However, support against the monophyly of *Hyphomonadaceae* was low. In the CCT an according clade was apparent that was composed of the genera *Algimonas* (Fukui et al., 2013), *Fretibacter* (Cho et al., 2013), *Glycocaulis* (Abraham et al., 2013; Lv et al., 2014), *Hellea* (Alain et al., 2008), *Hyphobacterium* (Sun et al., 2017), *Litorimonas* (Jung et al., 2011), *Maricaulis* (Abraham et al., 1999), *Marinicauda* (Zhang et al., 2013), *Oceanicaulis* (Strömpl et al., 2003), *Robiginitomaculum* (Lee et al., 2007d), and *Woodsholea* (Abraham et al., 2004), but this clade formed the sister group of core *Hyphomonadaceae* with moderate support. For this reason, we refrain from re-classifying these eleven genera into a separate family, which is not precluded by their phenotype (Supplementary Table S1).

### Species and Subspecies

Values of dDDH similarity (Meier-Kolthoff et al., 2013a; Meier-Kolthoff and Göker, 2019) found to be higher or lower than expected given the current species and subspecies thresholds of 70% (Wayne et al., 1987) and 79%, respectively (Meier-Kolthoff et al., 2014b), as well as known and confirmed heterotypic synonyms, are shown in Table 1 for pairs of closely related strains. Multiple species and subspecies displayed a value above the 79% threshold, hence it is proposed that the according taxa be recognised as heterotypic synonyms at the subspecies level (see Table 1 for proposed synonyms). Conversely, some subspecies were shown to merit species status (Tindall, 2019b), specifically *Acetobacter pasteurianus* subsp. a*scendens* (De Ley and Frateur, 1974) and *Acetobacter pasteurianus* subsp. *paradoxus* (De Ley and Frateur, 1974). The name *Acetobacter ascendens* was already proposed (Kim et al., 2018) but the name does not appear to be validly published.

*Brevirhabdus pacifica* (Wu et al., 2015) was shown as paraphyletic in the phylogenomic analysis (Figure 7) because *Xuhuaishuia manganoxidans* (Wang L. et al., 2016) was nested within the two genome-sequenced type-strain deposits of the species. The dDDH analysis confirmed this result, as *X. manganoxidans* appeared as a later heterotypic synonym of *B. pacifica*. As the genus *Brevirhabdus* was not considered in the study where *X. manganoxidans* was proposed, the phylogenetic relationship of those taxa could not be elucidated back then.

*Mameliella* (Zheng et al., 2010; Chen Z. et al., 2015) appeared as paraphyletic in the GBDP and in the 16S trees (Figure 9 and Supplementary File S2) because *Alkalimicrobium pacificum* (Zhang D.-C. et al., 2015) and *Ponticoccus lacteus* (Yang Y. et al., 2015) were nested within *Mameliella*. When *A. pacificum* was proposed, it appeared as sister group of *Mameliella alba* (Zheng et al., 2010). When *P. lacteus* was proposed, the genus *Mameliella* was not considered in the study, and hence their phylogenetic relationship could not be elucidated. According to a recent study (Liu et al., 2018), *A. pacificum* and *P. lacteus* are later heterotypic synonyms of *Mameliella alba*, as is *Mameliella atlantica* (Xu et al., 2015). This was confirmed by the here calculated dDDH values (Table 1).

Some dDDH values between pairs of species were found to be higher than 70%, the currently accepted threshold to differentiate among species (Wayne et al., 1987) and lower than 79%, the threshold defined to differentiate among subspecies (Meier-Kolthoff et al., 2014b). Based on the dDDH values we here concluded that *Rickettsia japonica* (Uchida et al., 1992) is best assigned to a subspecies of *Rickettsia conorii* (Brumpt, 1932). However, the type strain of *Rickettsia japonica* is only deposited in a single culture collection, which prevents us from proposing an according new combination. In addition, we propose that *Borrelia bavariensis* (Margos et al., 2013), *Gluconobacter nephelii* (Kommanee et al., 2011), *Methylobacterium chloromethanicum* (McDonald et al., 2001), *Methylorubrum extorquens* (Urakami and Komagata, 1984; Green and Ardley, 2018), *Pseudorhizobium pelagicum* (Kimes et al., 2015), *Rhizobium loessense* (Wei et al., 2003), *Rickettsia gravesii, Rickettsia heilongjiangensis, Rickettsia raoultii* (Mediannikov et al., 2008) *Rickettsia buchneri* (Kurtti et al., 2015), and *Ruegeria pelagia* (Lee et al., 2007c) be classified as *Borrelia garinii* subsp. *bavariensis*, subsp. nov., *Gluconobacter japonicus* subsp. *nephelii*, subsp. nov., *Methylobacterium dichloromethanicum* subsp. *chloromethanicum*, subsp. nov., *Methylobacterium dichloromethanicum* subsp. *extorquens*, subsp. nov., *Rhizobium marinum* subsp. *pelagicum*, subsp. nov., *Rhizobium mongolense* subsp. *loessense*, subsp. nov., *Rickettsia conorii* subsp. *gravesii*, subsp. nov., *Rickettsia conorii* subsp. *heilongjiangensis*, subsp. nov., *Rickettsia conorii* subsp. *raoultii*, subsp. nov., *Rickettsia tamurae* subsp. *buchneri*, subsp. nov., and *Tritonibacter mobilis* subsp. *pelagius*, subsp. nov., respectively (Table 1).

Finally, in the present study, all of the pairs of strains considered to represent distinct deposits of the same type strain were found to have dDDH similarities of 99.0% or above with the exception of *Celeribacter indicus* (Lai et al., 2014) strain MCCC 1A01112 and DSM 27257 (87.8%), *Celeribacter marinus* (Baek et al., 2014) strain IMCC12053 and DSM 100036 (94.8%), *Gluconacetobacter diazotrophicus* (Yamada et al., 1997) strain PAl 5 and DSM 5601 (92.1%), *Thalassobius mediterraneus* (Arahal et al., 2005) strain CECT 5383 and DSM 16398 (98.3%), *Thalassospira xiamenensis* (Liu et al., 2007) strain M-5 and DSM 17429 (94.7%), results which may account for the separation of each pair of these strains.

### Phylogenetic Conservation of Genomic and Phenotypic Markers of Interest

Table 2 shows the *p*-values obtained by the tip-permutation test and the retention values of selected phenotypic and genomic features. All investigated characters showed a significant phylogenetic conservation (α = 0.001) but the fit of each character to the tree varied considerably, as indicated by the retention index. A relatively high correspondence between G+C content and phylogeny and genome size and phylogeny was observed. Genome size varied between 0.85 Mbp in *Neorickettsia sennetsu* and 9.79 Mbp in *Bradyrhizobium arachidis* while G+C content varied between 26.86% in *Brachyspira alvinipulli* and 73.38% in *Rubritepida flocculans*. Morphology showed a significant but relatively low conservation but motility by flagella appeared certainly more conserved than cell length and particularly cell width.

Presence or absence of flagella also showed a better fit to the tree than the relationship to oxygen and the investigated chemotaxonomic features. Among these, presence or absence of carotenoids showed the highest conservation, followed by average number of isoprene residues in major ubiquinones and, finally, presence or absence of bacteriochlorophyll α. Particularly the chemotaxonomic features showed a low coverage in the taxon descriptions from the literature. The screening of the literature indicated that this may at least partially be due to the reluctance of researchers to report negative results. It is reasonable to assume that a perceived absence of bacteriochlorophyll α, carotenoids, or sphingolipids is not necessarily reported as such but simply omitted. In the case of sphingolipids the lack of reports for them outside *Sphingomonadales* even prevented the calculation of the metrics presented in Table 2. The less cautious coding that treated all missing values as indicating absence (Supplementary Table S1) yielded a high retention index for sphingolipids (0.739) whereas the retention indices of the other binary characters dropped compared to the values depicted in Table 2.

The gene-content analysis, specific aspects of which have already been discussed above, was largely in agreement with the GBDP tree even though certain parts of the trees were, predictably, in conflict (Supplementary File S2). For instance, using a branch-support threshold of 95% to indicate strong support or conflict, among the branches strongly supported by the GBDP analysis 40.6% were also strongly supported by the gene-content analysis, 6.7% were strongly contradicted and 52.7% of the cases the gene-content analysis remained neutral (Supplementary Table S1). Compared to the average branch support of the GBDP analysis of 92.1%, the gene-content analysis yielded 68.0% average support by itself and on average 45.2% support for the branches in the GBDP tree (Supplementary File S2).

## Discussion

### Causes of Conflict Between Phylogenomic Analyses and Taxonomic Classification

Most of the *Alphaproteobacteria* taxa already appeared monophyletic in this study eliminating need for a taxonomic revision. This partially reflects the work previously done by other authors where they proposed taxonomic changes within *Alphaproteobacteria* based on at least multi-gene if not phylogenomic datasets as, e.g., in the case of revisions of genera of *Rhodobacteraceae* (Breider et al., 2014; Wirth and Whitman, 2018). Much of the remaining discrepancies can be traced back to the way the 16S rRNA gene was interpreted in the taxonomic literature, which is discussed here while the interpretation of phenotypic features is treated in the next section.

In the case of some *Alphaproteobacteria* taxa such as *Mycoplana* (Urakami et al., 1990) and *Agrobacterium meteori* (Rüger and Höfle, 1992) the discrepancies between their classification and the modern methods was caused by the fact that they were described prior to the availability of 16S rRNA gene sequencing. In these low number of cases, the taxonomic conclusions that could be drawn with the 16S rRNA gene and those indicated by phylogenomic trees were essentially the same.

While in few cases, such as the original descriptions of *Angulomicrobium* and *Meganema*, branch support was not even calculated, most of the taxonomic discrepancies observed within the *Alphaproteobacteria* appeared to be caused by low resolution of the 16S rRNA genes used to propose the respective taxa. This held for genera such as *Alkalispirochaeta, Allorhizobium, Altererythrobacter, Celeribacter, Citreicella, Erythrobacter, Lutimaribacter, Mesorhizobium, Neorhizobium, Novosphingobium*, *Ochrobactrum, Porphyrobacter, Rhizobium, Roseomonas, Sphingomonas*, *Sphingopyxis, Thalassobius*, and *Tropicimonas*, as well as the families *Hyphomicrobiaceae, Phyllobacteriaceae, Rhodobiaceae, Rhodospirillaceae*, and *Sphingomonadaceae*. Appropriately calculating branch support is a necessary but not a sufficient prerequisite for safely generating monophyletic taxa. When drawing taxonomic conclusions from some phylogenetic tree, taxa must also be chosen so as to correspond to highly supported clades (Vences et al., 2013). Such clades are not always present in 16S rRNA genes although all species must be assigned to a genus in the Linnaean system.

The second most important cause of non-monophyly in the class *Alphaproteobacteria* detected in the current study was incomplete taxon sampling. Actually non-monophyletic taxa may easily appear monophyletic when species or strains of relevance, in particular type strains of type species of genera or even type genera of families or orders, are omitted from phylogenetic analysis. Incomplete taxon sampling affected the taxonomic assignment to families in the case of *Acuticoccus*, *Aquamicrobium*, *Breoghania*, *Cucumibacter*, *Devosia*, *Magnetovibrio*, *Maritalea*, *Marivibrio*, *Pelagibacterium*, *Reyranella*, *Tagaea*, *Terasakiella*, *Thalassospira*, and *Varunaivibrio*. Insufficient sampling also affected the assignment of species to genera at least in the case of *Novosphingobium tardaugens* and *Sphingopyxis baekryungensis*.

We found no evidence for a real conflict between the 16S rRNA gene and entire genomes in this study. In previous analyses (Hahnke et al., 2016; Nouioui et al., 2018) such conflicts already appeared to be rare compared to the overall number of taxa investigated and compared to the more common causes of taxonomic conflicts, which were the same as the ones listed above. Analyzing comprehensive sets of 16S rRNA gene sequences appears to be necessary unless a genome sequence is available for all type strains, particularly because taxonomic problems caused by insufficient taxon sampling seem to be quite common. The use of a backbone constraint can integrate information from analyses of more genes from few organisms into comprehensible sampled single-gene data (Liu X.-Z. et al., 2015; Hahnke et al., 2016; Nouioui et al., 2018). For example, the CCT was necessary to safely place type species that still lack a genome sequence and to detect instances where taxonomic conclusions would have been premature.

In previous studies (Hahnke et al., 2016; Nouioui et al., 2018) the taxonomic conclusions drawn from the GBDP tree were confirmed by corresponding supermatrix analyses in all of the cases investigated. In the present study it was also assessed whether conflict was evident between trees inferred from distinct data sets and, if so, to conduct analyses with alternative methods. We could not detect such conflict in *Alphaproteobacteria*, however, and accordingly restricted supermatrix analyses to cases in which the GBDP tree was insufficiently resolved for clarifying specific taxonomic questions. In these situations, conflict between the GBDP tree and the supermatrix analyses was not detected either. Our approach thus appeared to be as robust for *Alphaproteobacteria* as in previous studies on other phyla (Hahnke et al., 2016; Nouioui et al., 2018). Whole-genome methods, such as GBDP, yield truly genome-based phylogenies instead of approaches that use only a limited number of genes, which also rely on assumptions about the relative suitability of the selected genes compared to other genes (Lienau and DeSalle, 2009; Klenk and Göker, 2010). Distance methods for tree reconstruction still represent the most promising approach for accurately building phylogenies with a huge number of tips (Desper and Gascuel, 2004, 2006; Lefort et al., 2015). In an approach using GBDP in conjunction with FastME the time-consuming step is the calculation of the intergenomic distances, which can be done incrementally since the pairwise distances can be calculated independently of each other.

While the use of genome-scale data often yields more strongly resolved trees, it may also increase incongruities between distinct analyses (Jeffroy et al., 2006; Klenk and Göker, 2010). In this context overestimating phylogenetic confidence from genome-scale data must be avoided (Taylor and Piel, 2004). A reduction of the supposed incongruities between phylogenies and thereby more realistic support values for phylogenomic analyses can be obtained by bootstrapping entire genes instead of single alignment positions (Falush et al., 2006; Siddall, 2010; Hahnke et al., 2016; Simon et al., 2017). GBDP pseudo-bootstrapping in conjunction with the greedy-with-trimming algorithm (Meier-Kolthoff et al., 2014a; Meier-Kolthoff and Göker, 2019) is akin to such a “partition bootstrap.” The concept of hierarchical classification itself has been called into question based on topological incongruities between analyses of single genes that were attributed to horizontal gene transfer (Bapteste and Boucher, 2009; Klenk and Göker, 2010). However, the addition of more genes (Breider et al., 2014), up to virtually all available ones, as in the present study, yields strong support even when based on a careful approach to statistical resampling. This observation indicates a strong hierarchical signal and thus no reason to abandon the hierarchical system of taxonomic classification.

### Agreement and Conflict of Taxonomic Markers With Genome-Scale Phylogeny

Phylogenetic conservation of investigated phenotypic markers was detectable but varied and was on average not particularly pronounced (Table 2). This outcome is in overall agreement with an earlier study (Barberán et al., 2017) which compared a 16S rRNA gene tree to selected phenotypic features collected from recent taxonomic descriptions published in the *International Journal of Systematic and Evolutionary Microbiology* across phyla. While it comes as no surprise that cell shape is less conserved than presence or absence of flagella (Barberán et al., 2017), the relative performance of certain character deserves further discussion. Two distinct kinds of causes for the discrepancies between the phylogenomic trees and the traditional classification, which was at least partially based on phenotypic characters, are possible. The taxonomic interpretation of these characters could be insufficient (Montero-Calasanz et al., 2017; Nouioui et al., 2018), and the characters themselves could conflict with the phylogenomic trees. For distinguishing between these two options, the characters need to be examined as they were used in the taxonomic literature. For a historical assessment such as ours it makes no sense to analyze new, modified characters derived from the earlier ones. While it may be of interest in other respects to take the genetic background into account to form new characters, these new characters were not used for establishing the traditional classification in the first place.

Characters such as flagellum production or capability for photosynthesis are based on multiple genes usually arranged in gene clusters (Frank et al., 2015; Brinkmann et al., 2018) and thus can be assumed to be rather complex characters. According to Dollo’s law, complex features arise only once but can be lost several times in evolution (Le Quesne, 1974; Farris, 1977). Accordingly, a group of organisms which display a complex feature are expected to be monophyletic or paraphyletic in a tree, but not polyphyletic (Nouioui et al., 2018). The failure to properly distinguish between plesiomorphic (ancestral) character states, which indicate paraphyletic groups, and apomorphic (derived) character states, which indicate monophyletic groups (Hennig, 1965; Wiley and Lieberman, 2011), may account for some of the discrepancies between the current taxonomic classification and genome-scale trees (Montero-Calasanz et al., 2017; Nouioui et al., 2018). Homoplasy in the investigated characters most likely plays a role in other cases, and evolution according to Dollo’s law alone could cause homoplasy in a complex character. In the case of the photosynthesis in *Alphaproteobacteria*, however, it was shown that horizontal transfer of single genes as well as of entire photosynthesis operons occurred in addition to frequent losses of the capability for photosynthesis (Brinkmann et al., 2018). Such vertical transfer of complex characters may not only be facilitated by the arrangement of the according genes in gene clusters but also by the location of these gene clusters on extrachromosomal elements. This holds not only for the photosynthesis operon (Brinkmann et al., 2018) but also for the flagellum gene cluster (Frank et al., 2015). Losses and horizontal transfer can both contribute to the low phylogenetic conservation of phenotypic features, which in turn can contribute to discrepancies between the taxonomic classification and the phylogeny. It should not be overlooked, however, that certain phenotypic features can have an excellent fit to the phylogeny, as most likely in the case of the production of sphingolipids in *Alphaproteobacteria* (Supplementary File S2).

As for genomic features, bacterial G+C content and bacterial genome size appeared to be strongly phylogenetically conserved in *Alphaproteobacteria* (Supplementary File S2 and Table 2). For this reason, genome sizes have been added to the description of the reclassified and emended species as shown below. A significant correlation between genome size and G+C content was found in previous studies (Almpanis et al., 2018; Nouioui et al., 2018), which is not unexpected because symbiotic bacteria tend to have smaller genomes and to be richer in A+T content (Rocha and Danchin, 2002; Mann and Chen, 2010), an effect that may even impact the amino-acid content (Cole et al., 1998). Conversely, positive selection (Hildebrand et al., 2010) and G+C-biased gene conversion (Lassalle et al., 2015) can increase the G+C content. Although exceptions from the rule that reduced genomes have a low G+C content are known (McCutcheon et al., 2009), the rule was confirmed in the present study, particularly regarding genome size and G+C content reduction in pathogens such as *Bartonella*, *Borrelia* and *Rickettsiales* (Supplementary File S2). Because of this correlation caused by adaptive processes, genome size could thus be regarded as non-independent of G+C content, which would cast some doubt on its use as a taxonomic marker. Yet as shown previously (Nouioui et al., 2018) the overall correlation between G+C content and genome size is considerably reduced in strength after accounting for the impact of the phylogeny. This correlation may even only be due to G+C reduction effects in symbiotic bacteria with dramatically reduced genome sizes. The apparently non-random distribution with respect to the phylogeny of such reductions in *Alphaproteobacteria* (Supplementary File S2) also underlines the value of both G+C content and genome size as taxonomic markers in the class.

Now that it has been shown that within-species deviation in G+C content is at most 1% (Meier-Kolthoff et al., 2014c), many *Alphaproteobacteria* species descriptions were found to be inaccurate or too imprecise. The same observation was made in our earlier studies (Hahnke et al., 2016; Nouioui et al., 2018) on other groups of bacteria. As such values not only assist in detecting strains that do not belong to the same species but also show significant correlation to phylogenetic trees it is good practise to strengthen species descriptions in this way. In contrast, in most cases it is premature to redefine genera and higher taxa of *Alphaproteobacteria* in this way since additional type-strain genome sequences would be needed before this issue could be addressed. Only few genera were comprehensively sampled genomically in this study, which allows for providing respective emendations below.

Single characters may or may not be optimal as taxonomic markers, as they are often chosen for historical reasons. The application of gene-content phylogenies is more attractive for taxonomic purposes as they can be based on a huge number of characters now that genome sequences are available for many species. Using distance methods for inferring gene-content phylogenies is not a new idea; in particular, distance formulas were favoured which disregard double absence of genes to account for independent genome reduction (Wolf et al., 2002; Huson and Steel, 2004; Yang et al., 2005). GBDP formula *d3* follows the same approach (Henz et al., 2005). While more sophisticated approaches have been proposed (Gu and Zhang, 2004; Huson and Steel, 2004), we here used GBDP to infer gene-content phylogenies because the method is established. GBDP also allows for a direct comparison with methods based on sequence identity as only a single factor, the distance formula (*d*_3_ vs. *d*_5_), needs to be considered.

It must be taken into account that gene-content phylogenies may fail to recover the “true” tree for a variety of reasons, including horizontal gene transfer and gene loss; depending on the perspective, this may lead to big-genome attraction or small-genome attraction (Wolf et al., 2001; Lake and Rivera, 2004). Last but not least, many published genome sequences are not closed, which does not necessarily add a bias but most likely some noise to gene-content data. For these reasons we do not recommend gene-content approaches for inferring phylogenies although for some data sets they can certainly yield the same topology as approaches based on sequence alignment (Breider et al., 2014). Rather, we regard gene content as of interest for selecting branches from a phylogenetic tree, which was inferred by using a standard genome-scale approach, to obtain a taxonomic classification. Using the gene content directly for the purpose of classification irrespective of whether or not monophyletic taxa are generated (Zhu et al., 2015) is not in agreement with the principles of phylogenetic classification (Wiley and Lieberman, 2011). However, taking gene-content data into account is of interest because gene content conveys metabolic and other phenotypic capabilities (Zhu et al., 2015).

Our results indicate that gene content can be used in this manner to improve the taxonomic classification. In this respect is may even be advantageous if certain parts of the gene-content tree conflicted with the standard genome-scale analysis because this would narrow down the number of branches of the tree to be selected to form taxa. The results of the present study also indicated that gene-content analysis can provide useful insights into the evolution of *Alphaproteobacteria* and most likely also other groups of bacteria. For instance, the basal branches within *Rhodobacterales* are well supported in the gene-content analysis, reaching 100% for *Rhodobacteraceae*. This may be indicative of specific changes in gene content that occurred early on within the radiation of the order. As a variety of representatives of the family are dominant in marine habitats (Brinkhoff et al., 2008), these changes may be of considerably ecological interest. The genomic basis of the switches of *Rhodobacteraceae* between marine and non-marine habitats has recently been elucidated (Simon et al., 2017). A logical next step in future studies is to reconstruct the changes in gene content which took place at the basis of the *Rhodobacterales* part of the tree and led to the organisms that later on split into the numerous species of *Rhodobacteraceae*. For reasons of taxonomic conservatism, we here refrained from reclassifying monophyletic taxa solely based on their lack of support by the gene-content analysis. However, in this study the gene content appeared valuable for delineating new taxa in a variety of cases in which the existing taxa needed to be revised because they were evidently non-monophyletic. Considering gene content can apparently reduce arbitrariness in taxonomic decisions and increase the information content of the taxonomic system. It thus deserves attention in future studies on genome-based taxonomic classification of *Alphaproteobacteria* and other groups of organisms.

## Conclusion and Outlook

The results of this study provide a further improved framework for the classification of the class *Alphaproteobacteria*. The newly proposed taxonomic classification provides a sound basis for future studies on these bacteria, not least on those of ecological interest such as *Rhodobacteraceae*, which are major players in many marine habitats. Discrepancies of the results from the comparative phylogenomic approach with aspects of the previous taxonomic classification based on the 16S rRNA gene were mainly caused by insufficient taxon sampling and disregarded or overestimated branch support. Exceptions in which the 16S rRNA gene is in real conflict with genome-scale phylogenies were not observed. While they are, expectedly, not in full agreement, the relatively high correspondence between gene-content phylogeny and standard genome-scale analyses yields “grist to the taxonomic mill.” Also encouraging is the strong agreement between G+C content, genome size and selected phenotypic features on the one hand and truly genome-scale phylogenies of *Alphaproteobacteria* on the other hand. Future phylogenomic studies should try to link such key features, their genomic basis and their evolutionary relationships, and make an attempt to clarify evolutionary relationships that could as yet not be resolved. This affects particularly those groups that were thoroughly sampled but are not yet well covered by genome sequencing. Success in *Alphaproteobacteria* may help to revitalize prokaryotic systematics as a fundamental scientific discipline in other parts of the bacterial tree of life, particularly if it manages to integrate the distinct types of rich data that are nowadays available.

### Taxonomic Consequences: New Orders

#### Description of *Mariprofundales* ord. nov.

Ma.ri.pro.fun.da’les (N.L. masc. n. *Mariprofundus*, the type genus of the order; -*ales*, ending to denote an order; N.L. fem. pl. n. *Mariprofundales*, the order of *Mariprofundus*).

The description is the same as for the family *Mariprofundaceae* fam. nov. (Emerson et al., 2007), the sole family in the order, which was proposed earlier on but has not been validly published yet. The type genus of the order is *Mariprofundus.* Phylogenetic analyses of genome and 16S rRNA gene sequences indicate that the genus is best placed into an order of its own.

### Taxonomic Consequences: New Families

#### Description of *Afifellaceae* fam. nov.

A.fi.fel.la’ce.ae (N.L. fem. dim. n. *Afifella*, type genus of the family; *-aceae*, ending to denote a family; N.L. fem. pl. n. *Afifellaceae*, the *Afifella* family).

The description is as given for *Afifella* (Urdiain et al., 2008), which is the type and currently the sole genus of the family. This family has been separated from other families based on phylogenetic analyses of genome and 16S rRNA gene sequences.

#### Description of *Ahrensiaceae* fam. nov.

Ah.rens.i.a’ce.ae (N.L. fem. n. *Ahrensia*, type genus of the family; *-aceae*, ending to denote a family; N.L. fem. pl. n. *Ahrensiaceae*, the *Ahrensia* family).

Cells are Gram-negative, ovoid to rod-shaped, non-motile or motile by means of polar or peritrichous flagella. Aerobic, oxidase and catalase positive. The major ubiquinone is Q-10. The major named polar lipids are phosphatidylcholin, phosphatidylethanolamine, phosphatidylglycerol and diphosphatidylglycerol. The major fatty acid is C_18:1_ ω7c. The G+C content as calculated from genome sequences is around 48.1–50.1% while the range provided in the literature is 48.1–60.1 mol%. The family currently comprises the genera *Ahrensia* (the type genus) and *Pseudahrensia.*

#### Description of *Amorphaceae* fam. nov.

A.mor.pha’ce.ae (N.L. masc. n. *Amorphus*, type genus of the family; *-aceae*, ending to denote a family; N.L. fem. pl. n. *Amorphaceae*, the *Amorphus* family).

Cells are Gram-negative, aerobic, with variable morphology and non-flagellated. The major ubiquinone is Q-10. The major fatty acids are C_18:1_ ω7c and C_19:0_ cyclo ω8c. The family currently comprises the genera *Acuticoccus* and *Amorphus* (the type genus). This family has been separated from other families based on phylogenetic analyses of genome and 16S rRNA gene sequences.

#### Description of *Aurantimonadaceae* fam. nov.

Au.ran.ti.mo.na.da’ce.ae (N.L. fem. n. *Aurantimonas*, type genus of the family; *-aceae*, ending to denote a family; N.L. fem. pl. n. *Aurantimonadaceae*, the *Aurantimonas* family).

Cells are Gram-negative, rod-shaped, aerobic to facultatively aerobic and either non-motile or motile by means of flagella. The major ubiquinone is Q-10. Usually catalase- and oxidase positive. Growth occurs under mesophilic conditions. Carotenoids can be present. NaCl requirement is variable. The major named polar lipids are diphosphatidylglycerol, phosphatidylglycerol, phosphatidylcholin, phosphatidylethanolamine, phosphati- dylmonomethylethanolamine and aminolipids. The major named fatty acids are usually C_18:1_ ω7c and C_19:0_ cyclo ω8c. The family currently comprises the genera *Mangrovicella, Jiella, Aurantimonas* (the type genus), *Aureimonas, Fulvimarina* and *Consotaella.*

#### Description of *Azospirillaceae* fam. nov.

A.zo.spi.ril.la’ce.ae (N.L. neut. dim. n. *Azospirillum*, type genus of the family; *-aceae*, ending to denote a family; N.L. fem. pl. n. *Azospirillaceae*, the *Azospirillum* family).

Cells are Gram-negative, mostly rod-shaped, in general aerobic, usually motile by flagella, usually heterotrophic. The major ubiquinone is predominantly Q-10. The major named polar lipids are phosphatidylcholin, phosphatidylethanolamine, phosphatidylglycerol and diphosphatidylglycerol. The major fatty acids are usually C_18:1_ ω7c, C_18:1_ ω6c, C_16:0_ and C_16:1_ ω6c. The family currently comprises the genera *Niveispirillum, Azospirillum* (including *Conglomeromonas*), *Skermanella, Nitrospirillum*, *Rhodocista*, and *Desertibacter.* The genera *Elstera, Inquilinus* and *Lacibacterium* are tentatively assigned to this family. The type genus is *Azospirillum.*

#### Description of *Blastochloridaceae* fam. nov.

Blas.to.chlo.ri.da’ce.ae (N.L. fem. n. *Blastochloris*, type genus of the family; *-aceae*, ending to denote a family; N.L. fem. pl. n. *Blastochloridaceae*, the *Blastochloris* family).

The description is as given for *Blastochloris* (Hiraishi, 1997), which is the type and currently the sole genus of the family. This family has been separated from *Hyphomicrobiaceae* based on phylogenetic analyses of genome and 16S rRNA gene sequences.

#### Description of *Boseaceae* fam. nov.

Bo.se.a’ce.ae (N.L. fem. n. *Bosea*, type genus of the family; *-aceae*, ending to denote a family; N.L. fem. pl. n. *Boseaceae*, the *Bosea* family).

The description is as given for *Bosea* (Das et al., 1996; La Scola et al., 2003), which is the type genus and currently the sole genus of the family. This family has been separated from *Nitrobacteraceae* (whose illegitimate synonym is *Bradyrhizobiaceae*) based on phylogenetic analyses of genome and 16S rRNA gene sequences.

#### Description of *Breoghaniaceae* fam. nov.

Bre.o.gha.ni.a’ce.ae (N.L. fem. n. *Breoghania*, type genus of the family; *-aceae*, ending to denote a family; N.L. fem. pl. n. *Breoghaniaceae*, the *Breoghania* family).

The description is as given for *Breoghania* (Gallego et al., 2010),which is the type and currently the sole genus of the family. This family has been separated from other families based on phylogenetic analyses of genome and 16S rRNA gene sequences.

#### Description of *Devosiaceae* fam. nov.

De.vo.si.a’ce.ae (N.L. fem. n. *Devosia*, type genus of the family; *-aceae*, ending to denote a family; N.L. fem. pl. n. *Devosiaceae*, the *Devosia* family).

Cells are Gram-negative, predominantly rod-shaped, aerobic, usually motile by flagella, and heterotrophic. The major ubiquinone is predominantly Q-10 whereas Q-9 and Q-11 present as minor ubiquinone in some species. The major polar lipids are phosphatidylglycerol, diphosphatidylglycerol and glycolipid. The major fatty acids are usually C_18:1_ ω7c, 11-methyl C_18:1_ ω7c, C_18:1_ ω6c, C_18:0_, C_16:0_ ω6c and C_19:0_ cyclo ω8c. The family currently comprises the genera *Arsenicitalea*, *Cucumibacter*, *Devosia* (the type genus), *Maritalea* (including *Zhangella*), *Methyloterrigena*, *Paradevosia*, *Pelagibacterium*, and *Youhaiella.* This family has been separated from *Hyphomicrobiaceae* based on phylogenetic analyses of genome and 16S rRNA gene sequences.

#### Description of *Kaistiacea*e fam. nov.

Kais.ti.a’ce.ae (N.L. fem. n. *Kaistia*, type genus of the family; *-aceae*, ending to denote a family; N.L. fem. pl. n. *Kaistiacea*e, the *Kaistia* family).

Cells are Gram-negative, cocci or rod-shaped, non-motile, aerobic and chemoorganotrophic. The G+C content as calculated from genome sequences is around 64.5–67.1% while the range provided in the literature is 61.6–69.0 mol%. The family currently comprises the genera *Kaistia* (the type genus) and *Bauldia.*

#### Description of *Mariprofundaceae* fam. nov.

Ma.ri.pro.fun.da’ce.ae (N.L. masc. n. *Mariprofundus*, type genus of the family; -*aceae*, ending to denote a family; N.L. fem. pl. n. *Mariprofundaceae*, the *Mariprofundus* family).

The description is that for *Mariprofundus* (Emerson et al., 2007), which is the type and currently sole genus of the family. The family was proposed earlier on but has not been validly published yet. It has been separated from other families based on phylogenetic analyses of genome and 16S rRNA gene sequences.

#### Description of *Neomegalonemataceae* fam. nov.

Ne.o.me.ga.lo.ne.ma.ta’ce.ae (N.L. neut. n. *Neomegalonema*, type genus of the family; *-aceae*, ending to denote a family; N.L. fem. pl. n. *Neomegalonemataceae*, the *Neomegalonema* family).

The description is as given for *Neomegalonema* (Oren, 2017b), which is the type and currently the sole genus of the family. This family has been separated from *Hyphomicrobiales* (known under the illegitimate synonym *Rhizobiales*) and from *Rhodobacteraceae* based on phylogenetic analyses of genome and 16S rRNA gene sequences.

#### Description of *Parvibaculaceae* fam. nov.

Par.vi.ba.cu.la’ce.ae (N.L. neut. n. *Parvibaculum*, type genus of the family; *-aceae*, ending to denote a family; N.L. fem. pl. n. *Parvibaculaceae*, the *Parvibaculum* family).

Cells are Gram-negative, usually rod-shaped, generally aerobic, motile by flagella or non-motile and heterotrophic. The major ubiquinone is Q-10 or Q-11. The major polar lipids are diphosphatidylglycerol, phosphatidylethanolamine and phosphatidylglycerol. The major fatty acid is usually C_18:1_ ω7c and in some cases C_16:0_ and C_19:0_ cyclo. The family currently comprises the genera *Parvibaculum* (the type genus) and *Tepidicaulis*. *Anderseniella*, *Rhodoligotrophos* and *Pyruvatibacter* are tentatively assigned to this family as well.

#### Description of *Phreatobacteraceae* fam. nov.

Phre.a.to.bac.te.ra’ce.ae (N.L. masc. n. *Phreatobacter*, type genus of the family; *-aceae*, ending to denote a family; N.L. fem. pl. n. *Phreatobacteraceae*, the *Phreatobacter* family).

The description is as given for *Phreatobacter* (Tóth et al., 2014; Lee S.D. et al., 2017), which is the type and currently the sole genus of the family. This family is proposed based on phylogenetic analyses of genome and 16S rRNA gene sequences.

#### Description of *Pleomorphomonadaceae* fam. nov.

Ple.o.mor.pho.mo.na.da’ce.ae (N.L. fem. n. *Pleomorphomonas*, type genus of the family; -*aceae*, ending to denote a family; N.L. fem. pl. n. *Pleomorphomonadaceae*, the *Pleomorphomonas* family).

Cells are Gram-negative, usually rod-shaped or spherical, generally aerobic and mostly diazotrophic. The major ubiquinone is predominantly Q-10. The major polar lipids are phosphatidylglycerol, phosphatidylcholin, phosphatidylethanolamine and sometimes phospholipid. The major fatty are usually C_18:1_ ω7c and C_18:__0_. The family currently comprises the genera *Chthonobacter*, *Hartmannibacter*, *Methylobrevis, Mongoliimonas*, *Oharaeibacter*, and *Pleomorphomonas* (the type genus). This family has been separated from other families based on phylogenetic analyses of genome and 16S rRNA gene sequences.

#### Description of *Pseudoxanthobacteraceae* fam. nov.

Pseu.do.xan.tho.bac.te.ra’ce.ae (N.L. masc. n. *Pseudoxanthobacter*, type genus of the family; -*aceae*, ending to denote a family; N.L. fem. pl. n. *Pseudoxanthobacteraceae*, the *Pseudoxanthobacter* family).

The description is as given for *Pseudoxanthobacter* (Arun et al., 2008), which is the type and currently sole genus of the family. This family has been separated from other families based on phylogenetic analyses of genome and 16S rRNA gene sequences.

#### Description of *Reyranellaceae* fam. nov.

Rey.ra.nel.la’ce.ae (N.L. fem. dim. n. *Reyranella*, type genus of the family; -*aceae*, ending to denote a family; N.L. fem. pl. n. *Reyranellaceae*, the *Reyranella* family).

The description is as given for *Reyranella* (Pagnier et al., 2011) which is the type and currently sole genus of the family. This family has been separated from other families based on phylogenetic analyses of genome and 16S rRNA gene sequences.

#### Description of *Rhodovibrionaceae* fam. nov.

Rho.do.vi.bri.o.na’ce.ae (N.L. masc. n. *Rhodovibrio*, type genus of the family; -*aceae*, ending to denote a family; N.L. fem. pl. n. *Rhodovibrionaceae*, the *Rhodovibrio* family).

Gram-negative, vibrioid, spiral or rod-shaped, non-motile or motile by means of polar flagella. Aerobic, facultatively anaerobic or anaerobic, with chemoorganotrophic or photoorganoheterotrophic metabolism. The major ubiquinone is Q-10; MK-10 was reported for *Rhodovibrio*. The major fatty acids are C_19:0_ cyclo ω8c, C_18:1_ ω7c and C_18:0_. The G+C content provided in the literature is 61.5–69.0 mol%. The family currently comprises the genera *Fodinicurvata*, *Limimonas*, *Rhodovibrio* (the type genus) and *Tistlia*. *Limibacillus* and *Pelagibius* are tentatively assigned to this family.

#### Description of *Sphaerochaetaceae* fam. nov.

Sphae.ro.chae.ta’ce.ae (N.L. fem. n. *Sphaerochaeta*, type genus of the family; -*aceae*, ending to denote a family; N.L. fem. pl. n. *Sphaerochaetaceae*, the *Sphaerochaeta* family).

Cells are Gram-negative, mostly non-motile, pleomorphic, anaerobic and heterotrophic. The family currently comprises the genera *Pleomorphochaeta* and *Sphaerochaeta* (the type genus). This family has been separated from other families based on phylogenetic analyses of genome and 16S rRNA gene sequences.

#### Description of *Sphingosinicellaceae* fam. nov.

Sphin.go.si.ni.cel.la’ce.ae (N.L. fem. n. *Sphingosinicella*, type genus of the family; -*aceae*, ending to denote a family; N.L. fem. pl. n. *Sphingosinicellaceae*, the *Sphingosinicella family*).

Cells are Gram-negative, aerobic, rod-shaped, mostly motile heterotrophs. The major ubiquinone is Q-10. The major named polar lipids are phosphatidylethanolamine, phosphatidylglycerol and in some species diphosphatidylglycerol. The major fatty acids are usually C_18:1_ ω7c, C_16:0_ and C_16:1_ ω7c. The family currently comprises the genera *Pacificimonas*, *Sphingosinicella* (the type genus), *Sandaracinobacter* and *Sandarakinorhabdus*. *Polymorphobacter* and *Sphingoaurantiacus* are tentatively assigned to this family. This family has been separated from other families based on phylogenetic analyses of genome and 16S rRNA gene sequences.

#### Description of *Stappiaceae* fam. nov.

Stap.pi.a’ce.ae (N.L. fem. n. *Stappia*, type genus of the family; -*aceae*, ending to denote a family; N.L. fem. pl. n. *Stappiaceae*, the *Stappia* family).

Cells are Gram-negative, usually rod-shaped, motile, mostly aerobic or facultatively anaerobic and heterotroph. The major ubiquinone is usually Q-10. The major fatty acids are predominantly C_18:1_ ω7c and C_16:1_ ω7c. The family currently comprises the genera *Pannonibacter*, *Pseudovibrio*, *Roseibium* (including *Labrenzia*) and *Stappia* (the type genus). This family has been separated from other families based on phylogenetic analyses of genome and 16S rRNA gene sequences.

#### Description of *Stellaceae* fam. nov.

Stel.la’ce.ae (L. fem. n. *Stella*, type genus of the family; *-aceae*, ending to denote a family; N.L. fem. pl. n. *Stellaceae*, the *Stella* family).

The description is as given for *Stella* (Vasilyeva, 1985), which is the type genus of the family, with the following modification. Cells are six-pronged stars, rods or ovoids. The family houses *Stella* and tentatively also *Constrictibacter*. This family has been separated from *Acetobacteraceae* based on phylogenetic analyses of genome and 16S rRNA gene sequences.

#### Description of *Tepidamorphaceae* fam. nov.

Te.pid.a.mor.pha’ce.ae (N.L. masc. n. *Tepidamorphus*, type genus of the family; *-aceae*, ending to denote a family; N.L. fem. pl. n. *Tepidamorphaceae*, the *Tepidamorphus* family).

Cells are Gram-negative, either ovoid or rod-shaped, predominantly aerobic, mostly motile via flagella and generally chemoorganothrophs. The major ubiquinone is Q-10. The major named polar lipids are diphosphatidylglycerol, phosphatidylethanolamine, and phosphatidylglycerol. The major fatty acids are usually C_19:0_ cyclo ω8c, C_18:1_ ω7c, C_18:0_ and C_16:0_. The family currently comprises the genera *Butyratibacter*, *Lutibaculum*, *Microbaculum*, and *Tepidamorphus* (the type genus).

#### Description of *Terasakiellaceae* fam. nov.

Te.ra.sa.ki.el.la’ce.ae (N.L. fem. dim. n. *Terasakiella*, type genus of the family; -*aceae*, ending to denote a family; N.L. fem. pl. n. *Terasakiellaceae*, the *Terasakiella* family).

The description is as given for *Terasakiella* (Satomi et al., 2002; Han et al., 2016), which is the type and currently sole genus of the family. This family has been separated from other families based on phylogenetic analyses of genome and 16S rRNA gene sequences.

#### Description of *Thalassobaculaceae* fam. nov.

Tha.las.so.ba.cu.la’ce.ae (N.L. neut. n. *Thalassobaculum*, type genus of the family; -*aceae*, ending to denote a family; N.L. fem. pl. n. *Thalassobaculaceae*, the *Thalassobaculum* family).

Gram-negative, rod-shaped, motile by means of one polar flagellum. Aerobic or facultatively anaerobic respiration and chemoorganotrophic metabolism. The major ubiquinone is Q-10. The major fatty acids are C_18:1_ ω7c, C_16:1_ ω7c and C_16:0_. The G+C content as calculated from genome sequences is around 60.5–67.4% while the range provided in the literature is 60–68 mol%. The family currently comprises the genera *Nisaea*, *Oceanibaculum* and *Thalassobaculum* (the type genus).

#### Description of *Thalassospiraceae* fam. nov.

Tha.las.so.spi.ra’ce.ae (N.L. fem. n. *Thalassospira*, type genus of the family; -*aceae*, ending to denote a family; N.L. fem. pl. n. *Thalassospiraceae*, the *Thalassospira* family).

Cells are Gram-negative, usually motile by flagella, mostly aerobic or microaerophilic with a heterotrophic or autolithothrophic metabolism. The major ubiquinone is predominantly Q-10. The major polar lipids are phosphatidylethanolamine and phosphatidylglycerol. The major fatty acids are usually C_18:1_ ω7c, C_16:1_ ω7c and C_16:0_. The family currently comprises the genera *Magnetovibrio*, *Terasakiella*, *Thalassospira* (the type genus), *Varunaivibrio*. *Magnetospira* is tentatively assigned to this family. This family has been separated from other families based on phylogenetic analyses of genome and 16S rRNA gene sequences.

#### Description of *Zavarziniaceae* fam. nov.

Za.var.zi.ni.a’ce.ae (N.L. fem. n. *Zavarzinia*, type genus of the family; *-aceae*, ending to denote a family; N.L. fem. pl. n. *Zavarziniaceae*, the *Zavarzinia* family).

The description is as given for *Zavarzinia* (Meyer et al., 1993), which is the type and currently sole genus of the family. This family has been separated from *Acetobacteraceae* based on phylogenetic analyses of genome and 16S rRNA gene sequences.

#### Description of *Zymomonadaceae* fam. nov.

Zy.mo.mo.na.da’ce.ae (N.L. fem. n. *Zymomonas*, type genus of the family; -*aceae*, ending to denote a family; N.L. fem. pl. n. *Zymomonadaceae*, the *Zymomonas family*).

The description is as given for *Zymomonas* (Kluyver and van Niel, 1936), which is the type and currently sole genus of the family. This family has been separated from other families based on phylogenetic analyses of genome and 16S rRNA gene sequences.

### Taxonomic Consequences: New Genera

#### Description of *Albibacillus* gen. nov.

Al.bi.ba.cil’lus (L. masc. adj. *albus*, white; L. masc. n. *bacillus*, rod; N.L. masc. n. *Albibacillus*, white rod).

Gram-negative, rod-shaped, aerobic, oxidase and catalase positive. The major ubiquinone is Q-10. The major polar lipids are phosphatidylglycerol, phosphatidylethanolamine, phosphatidylmonomethylethanolamine and aminolipids. The major fatty acids are C_18:1_ ω7c and C_19:0_ cyclo ω8c. The G+C content is around 64-66%. The type species is *Albibacillus kandeliae, comb.* nov.

#### Description of *Allgaiera* gen. nov.

All.gai’er.a (N.L. fem. n. *Allgaiera*, named after the German microbiologist Martin Allgaier for his work on marine *Rhodobacteraceae*).

The description is as given for *Allgaiera indica, comb.* nov., which is the type species. The genus has been separated from *Defluviimonas* based on physiology and phylogenetic analyses of genome and 16S rRNA gene sequences.

#### Description of *Allo*s*ediminivita* gen. nov.

Al.lo.se.di.mi.ni.vi’ta (Gr. masc. adj. *allos*, another, other, different; N.L. fem. n. *Sediminivita*, a bacterial genus; N.L. fem. n. *Allosediminivita*, a genus different from *Sediminivita*).

The description is as given for *Allo*s*ediminivita pacifica, comb.* nov., which is the type species. The genus has been separated from *Roseivivax* based on physiology and phylogenetic analyses of genome and 16S rRNA gene sequences.

#### Description of *Allosphingosinicella* gen. nov.

Al.lo.sphin.go.si.ni.cel’la (Gr. masc. adj. *allos*, another, other, different; N.L. fem. n. S*phingosinicella*, a bacterial genus; N.L. fem. n. *Allosphingosinicella*, a genus different from S*phingosinicella*).

Cells are Gram-negative, non-motile, non-spore-forming and rod-shaped. Strictly aerobic. The major ubiquinone is Q-10. The major polar lipids are diphosphatidylglycerol, phosphatidylglycerol and phosphatidylethanolamine. The major fatty acids are C_18:1_ ω7c, C_16:1_ ω7c, C_14:0_ 2-OH and C_16:0_. The G+C content as calculated from genome sequences is around 62.4–67.0% while the range provided in the literature is 59.4–65.8 mol%. The type species is *Allosphingosinicella vermicomposti* comb. nov.

#### Description of *Bieblia* gen. nov.

Biebl’i.a (N.L. fem. n. *Bieblia*, named after the German microbiologist Hanno Biebl for his work on marine *Rhodobacteraceae*).

Facultatively aerobic or anaerobic, Gram-negative, non-motile, rods or ovoids, mostly autotrophs. bacteriochlorophyll αnd carotenoids present. The G+C content is 64–69%. The type species is *Bieblia veldkampii, comb.* nov. The genus has been separated from *Rhodobacter* based on physiology and phylogenetic analyses of genome and 16S rRNA gene sequences.

#### Description of *Caenibius* gen. nov.

Cae.ni’bi.us (L. neut. n. *caenum*, mud, referring to the isolation of the type strain from activated sludge; N.L. masc. n. *bius*, life (from Gr. n. *bios*); N.L. masc. n. *Caenibius*, sludge life).

The description is as given for *Caenibius tardaugens, comb.* nov., which is the type species. The genus has been separated from *Novosphingobium* based on physiology and phylogenetic analyses of genome and 16S rRNA gene sequences.

#### Description of *Cypionkella* gen. nov.

Cy.pi.on.kel’la (N.L. fem. dim. n. *Cypionkella*, named after the German microbiologist Heribert Cypionka for his work on marine *Rhodobacteraceae*).

Cells are Gram-negative, rod-shaped or oval, non-motile, mostly aerobic, heterotrophic. Catalase and oxidase positive. The predominant ubiquinone is Q-10. The major polar lipids are phosphatidylglycerol and phosphatidylcholin. The major fatty acid is C_18:1_ ω7c. The G+C content is 60–62%. The type species is *Cypionkella psychrotolerans*, comb. nov. The genus has been separated from *Pseudorhodobacter* based on phylogenetic analyses of genome and 16S rRNA gene sequences.

#### Description of *Elkelangia* gen. nov.

El.ke.lang’i.a (N.L. fem. n. *Elkelangia*, named after Elke Lang, a German microbiologist known for her work as long term curator of Gram negative bacteria at DSMZ).

The description is as given for *Elkelangia baekryungensis, comb.* nov., which is the type species. The genus has been separated from *Sphingopyxis* based on physiology and phylogenetic analyses of genome and 16S rRNA gene sequences.

#### Description of *Meinhardsimonia* gen. nov.

Mein.hard.si.mon’i.a (N.L. fem. n. *Meinhardsimonia*, named after the German microbiologist Meinhard Simon for his work on marine *Rhodobacteraceae*).

The description is as given for *Meinhardsimonia xiamenensis, comb.* nov., which is the type species. The genus has been separated from *Albidovulum* based on physiology and phylogenetic analyses of genome and 16S rRNA gene sequences.

#### Description of *Neopararhizobium* gen. nov.

Ne.o.pa.ra.rhi.zo’bi.um (Gr. pref. *neo*, new; N.L. neut. n. *Pararhizobium*, the genus *Pararhizobium;* N.L. neut. n. *Neopararhizobium*, new *Pararhizobium*).

The type species is *Neopararhizobium haloflavum, comb.* nov. The genus has been separated from *Pararhizobium* based on phylogenetic analyses of genome and 16S rRNA gene sequences.

#### Description of *Pacificitalea* gen. nov.

Pa.ci.fi.ci.ta’le.a (L. masc. adj. *pacificus*, peaceful, referring to the Pacific Ocean; L. fem. n. *talea*, a rod; N.L. fem. n. *Pacificitalea*, a rod isolated from the Pacific Ocean).

The description is as given for *Pacificitalea manganoxidans, comb.* nov., which is the type species. The genus has been separated from *Celeribacter* based on physiology and phylogenetic analyses of genome and 16S rRNA gene sequences.

#### Description of *Pseudoprimorskyibacter* gen. nov.

Pseu.do.pri.mor.sky.i.bac’ter (Gr. masc. adj. *pseudês*, false*;* N.L. n. *primorsky/-yos*, primorsky kray, a far-Eastern region of the Russian federation where the first strains were isolated; N.L. masc. n. *bacter*, a rod; N.L. masc. n. *Pseudoprimorskyibacter*, like *Primorskyibacter*, referring to the close relationship to the genus *Primorskyibacter*).

The description is as given for *Pseudoprimorskyibacter insulae comb.* nov., which is the sole and type species. The genus has been separated from *Primorskyibacter* based on physiology and phylogenetic analyses of genome and 16S rRNA gene sequences.

#### Description of *Vannielia* gen. nov.

Van.niel’i.a (N.L. fem. n. *Vannielia*, named to honor Cornelis Bernardus van Niel, and his many contributions to microbiology).

The description is as given for *Vannielia litorea, comb.* nov., which is the type species. The genus has been separated from *Oceanicola* based on physiology and phylogenetic analyses of genome and 16S rRNA gene sequences.

#### Description of *Wagnerdoeblera* gen. nov.

Wag.ner.doeb’ler.a (N.L. fem. n. *Wagnerdoeblera*, named after the German microbiologist Irene Wagner-Döbler for her work on marine *Rhodobacteraceae*).

Gram-negative, non-motile, non-spore-forming bacteria. Cells are irregular rod-shaped. Aerobic. Oxidase and catalase positive. The major ubiquinone is Q-10. The major polar lipids are phosphatidylethanolamine, phosphatidylglycerol and phosphatidylcholine. The major fatty acid is C_18:1_ ω7c. The G+C content as calculated from genome sequences is around 64.9–66.2% while the range provided in the literature is 61.4–64.5 mol%. The type species is *Wagnerdoeblera nectariphila, comb.* nov.

### Taxonomic Consequences: New (Combinations for) Species

#### Description of *Acetobacter ascendens* comb. nov., Change of Rank

A. as.cen’dens (L. part. adj. *ascendens*, ascending, climbing).

Basonym: *Acetobacter pasteurianus* subsp. *ascendens* De Ley and Frateur, 1974 (Approved Lists 1980)

The description is as given for *Acetobacter pasteurianus* subsp. *ascendens* (De Ley and Frateur, 1974). The type strain is CCM 3612 = LMG 1590 = NCCB 51001.

#### Description of *Acetobacter paradoxus* comb. nov., Change of Rank

A. pa.ra.do’xus (L. masc. adj. *paradoxus*, strange, contrary to all expectation, paradoxical).

Basonym: *Acetobacter pasteurianus* subsp. *ascendens* De Ley and Frateur, 1974 (Approved Lists 1980)

The description is as given for *Acetobacter pasteurianus* subsp. *paradoxus* (De Ley and Frateur, 1974). The type strain is LMG 1591 = NCCB 53006.

#### Description of *Actibacterium lipolyticum* comb. nov.

A. li.po.ly’ti.cum (Gr. neut. n. *lipos*, fat; Gr. masc. adj. *lytikos*, able to loosen, dissolving; N.L. neut. adj. *lipolyticum*, dissolving fat or lipid).

Basonym: *Confluentimicrobium lipolyticum* Parker et al., 2019

The description is as given for *Confluentimicrobium lipolyticum* (Park et al., 2014d). The type strain is SSK1-4 = CECT 8621 = KCTC 42136.

#### Description of *Actibacterium naphthalenivorans* comb. nov.

A. naph.tha.le.ni.vo’rans (N.L. neut. n. *naphthalenum*, naphthalene; L. part. adj. *vorans*, devouring; N.L. part. adj. *naphthalenivorans*, naphthalene-consuming).

Basonym: *Confluentimicrobium naphthalenivorans* Jeong et al., 2015

The description is as given for *Confluentimicrobium naphthalenivorans* (Jeong et al., 2015). The type strain is NS6 = DSM 105040 = JCM 30828.

#### Description of *Afipia carboxidovorans* comb. nov.

A. car.bo.xi.do’vo.rans (L. masc. n. *carbo*, charcoal, carbon; Gr. masc. adj. *oxys*, sour, acid; L. v. *voro*, devour; N.L. part. adj. *carboxidovorans*, carbon-acid devouring).

Basonym: *Oligotropha carboxidovorans* (ex Meyer and Schlegel 1978) Meyer et al. 1994.

The description is as given for *Oligotropha carboxidovorans* (Meyer et al., 1993). The type strain is OM5 = DSM 1227 = ATCC 49405.

#### Description of *Albibacillus kandeliae* comb. nov.

A. kan.de’li.ae (N.L. gen. n. *kandeliae*, of *Kandelia*, referring to a genus of mangrove plant).

Basonym: *Ruegeria kandeliae* Zhang L. et al., 2018

The description is as given for *Ruegeria kandeliae* (Zhang L. et al., 2018). The type strain is DSM 104293 = MCCC 1K03284.

#### Description of *Allgaiera indica* comb. nov.

A. in’di.ca (L. fem. adj. *indica*, referring to the Indian Ocean, where the type strain was first isolated).

Basonym: *Defluviimonas indica* Jiang et al., 2014

The description is as given for *Defluviimonas indica* (Jiang et al., 2014). The type strain is 20V17 = DSM 24802 = JCM 17871.

#### Description of *Allorhizobium oryziradicis* comb. nov.

A. o.ry.zi.ra’di.cis (L. fem. n. *oryza*, rice; L. fem. n. *radix/-icis*, root; N.L. gen. n. *oryziradicis*, of the rice root).

Basonym: *Rhizobium oryziradicis* Zhao et al., 2017

The description is as given for *Rhizobium oryziradicis* (Zhao et al., 2017). The genomic G+C content of the type strain is 55.1%. Its approximate genome size is 5.16 Mbp. The type strain is KCTC 52413.

#### Description of *Allorhizobium taibaishanense* comb. nov.

A. tai.bai.shan.en’se (N.L. neut. adj. *taibaishanense*, of or belonging to the Taibaishan Mountains in the Shaanxi province of China, where the bacterium was isolated).

Basonym: *Rhizobium taibaishanense* Yao et al., 2012

The description is as given for *Rhizobium taibaishanense* (Yao et al., 2012). The type strain is DSM 100021 = HAMBI 3214.

#### Description of *Allo*s*ediminivita pacifica* comb. nov.

A. pa.ci’fi.ca (L. fem. adj. *pacifica*, peaceful, pertaining to the Pacific Ocean).

Basonym: *Roseivivax pacificus* Wu et al., 2013

The description is as given for *Roseivivax pacificus* (Wu et al., 2013) with the following modification. The G+C content of the type-strain genome is 66.2%, its approximate size 4.84 Mbp. The type strain is 22DY03 = DSM 29329 = JCM 18866.

#### Description of *Allosphingosinicella indica* comb. nov.

A. in’di.ca (L. fem. adj. *indica*, of India, the origin of the type strain).

Basonym: *Sphingomonas indica* Niharika et al., 2012

The description is as given for *Sphingomonas indica* (Niharika et al., 2012). The genomic G+C content of the type strain is 67.0%. Its approximate genome size is 2.81 Mbp. The type strain is Dd16 = CCM 7882 = DSM 25434.

#### Description of *Allosphingosinicella vermicomposti* comb. nov.

A. ver.mi.com.pos’ti (L. neut. n. *vermis*, worm; N.L. neut. n. *compostum*, compost; N.L. gen. n. *vermicomposti*, of vermicompost, referring to the isolation of the type strain from vermicompost).

Basonym: *Sphingosinicella vermicomposti* Yasir et al., 2010

The description is as given for *Sphingosinicella vermicomposti* (Yasir et al., 2010) with the following modification. The G+C content of the type-strain genome is 62.4%, its approximate size 2.51 Mbp. The type strain is YC7378 = DSM 21593 = KCTC 22446.

#### Description of *Aminobacter carboxidus* comb. nov.

A. car.bo’xi.dus (L. masc. adj. *carboxidus*, intended to mean connected with carbon oxides).

Basonym: *Carbophilus carboxidus* (ex Nozhevnikova and Zavarzin 1974) Meyer et al. 1994.

The description is as given for *Carbophilus carboxidus* (Meyer et al., 1993). The type strain is ATCC 51424 = CIP 105722 = DSM 1086.

#### Description of *Bieblia veldkampii* comb. nov.

B. veld.kamp’i.i (N.L. gen. n. *veldkampii*, of Veldkamp, named after Hans Veldkamp, a Dutch microbiologist).

Basonym: *Rhodobacter veldkampii* Hansen and Imhoff, 1985

The description is as given for *Rhodobacter veldkampii* (Hansen and Imhoff, 1985) with the following restriction. The G+C content of the type-strain genome is 65.1%, its approximate size 3.26 Mbp. The type strain is BN 714 = DSM 11550 = ATCC 35703 = CIP 103912 = IFO 16458 = NBRC 16458.

#### Description of *Bieblia vinaykumarii* comb. nov.

B. vi.nay.ku.ma’ri.i (N.L. gen. n. *vinaykumarii*, of Vinaykumar, named after the late Dr. M. Vinaykumar, an Indian microbiologist and research supervisor of Ch. V. Ramana. and Ch. Sasikala, who initiated work on anoxygenic phototrophic bacteria in India).

Basonym: *Rhodobacter vinaykumarii* Srinivas et al., 2007a

The description is as given for *Rhodobacter vinaykumarii* (Srinivas et al., 2007b). The type strain is CCUG 54311 = DSM 18714 = JCM 14544.

#### Description of *Brucella anthropi* comb. nov.

B. an.thro’pi (Gr. masc. n. *anthropos*, a human being; N.L. gen. n. *anthropi*, of a human being).

Basonym: *Ochrobactrum anthropi* Holmes et al., 1988

The description is as given for *Ochrobactrum anthropi* (Holmes et al., 1988). The type strain is ATCC 49188 = CCUG 24695 = CIP 82.115 = DSMZ 6882 = IFO 15819 = JCM 21032 = LMG 3331 = NBRC 15819 = NCTC 12168.

#### Description of *Brucella ciceri* comb. nov.

B. ci.ce’ri (L. gen. n. *ciceri*, of chickpea (*Cicer arietinum*), referring to the habitat from which the type strain was isolated).

Basonym: *Ochrobactrum ciceri* Imran et al., 2010

The description is as given for *Ochrobactrum ciceri* (Imran et al., 2010). The type strain is CCUG 57879 = DSM 22292.

#### Description of *Brucella cytisi* comb. nov.

B. cy.ti’si (N.L. masc. n. *Cytisus*, botanical genus name of the legume *Cytisus scoparius*; N.L. gen. n. cytisi, of *Cytisus*, referring to the isolation source of the first strains, nodules of *Cytisus scoparius*).

Basonym: *Ochrobactrum cytisi* Zurdo-Piñeiro et al., 2007

The description is as given for *Ochrobactrum cytisi* (Zurdo-Piñeiro et al., 2007). The type strain is CECT 7172 = DSM 19778 = LMG 22713.

#### Description of *Brucella daejeonensis* comb. nov.

B. dae.jeon.en’sis (N.L. fem. adj. *daejeonensis*, of or pertaining to Daejeon, a city in South Korea, from where the type strain was isolated).

Basonym: *Ochrobactrum daejeonense* Woo et al., 2011

The description is as given for *Ochrobactrum daejeonense* (Woo et al., 2011). The type strain is DSM 26944 = JCM 16234 = KCTC 22458.

#### Description of *Brucella endophytica* comb. nov.

B. en.do.phy.ti’ca (Gr. pref. *endo*, within; Gr. neut. n. *phyton*, plant; L. neut. suff. *-icum*, adjectival suffix used with the sense of belonging to; N.L. fem. adj. *endophytica*, within plant, endophytic, because the type strain was isolated from the interior of a plant nodule).

Basonym: *Ochrobactrum endophyticum* Li L. et al., 2016

The description is as given for *Ochrobactrum endophyticum* (Li L. et al., 2016). The type strain is CGMCC 1.15082 = DSM 29930 = KCTC 42485.

#### Description of *Brucella gallinifaecis* comb. nov.

B. gal.li.ni.fae’cis (L. fem. n. *gallina*, hen; L. fem. n. *faex, faecis*, faeces; N.L. gen. n. *gallinifaecis*, of the faeces of a hen).

Basonym: *Ochrobactrum gallinifaecis* Kämpfer et al., 2003

The description is as given for *Ochrobactrum gallinifaecis* (Kämpfer et al., 2003). The type strain is CCUG 48291 = CIP 107753 = DSM 15295.

#### Description of *Brucella grignonensis* comb. nov.

B. gri.gnon.en’sis (N.L. fem. adj. *grignonensis*, pertaining to Grignon, region from which the strains were isolated).

Basonym: *Ochrobactrum grignonense* Lebuhn et al., 2000

The description is as given for *Ochrobactrum grignonense* (Lebuhn et al., 2000). The genomic G+C content of the type strain is 54.1%. Its approximate genome size is 4.84 Mbp. The type strain is CCUG 46362 = DSM 13338 = LMG 18954 = NBRC 102586.

#### Description of *Brucella haematophila* comb. nov.

B. hae.ma.to’phi.la (Gr. neut. n. *haima/-atos*, Latin transliteration *haema/-atos*, blood; N.L. adj. *philus/-a/-um*, from Greek adj. *philos/-ê/-on*, friend, loving; N.L. fem. adj. *haematophila*, blood-loving).

Basonym: *Ochrobactrum haematophilum* Kämpfer et al., 2007a

The description is as given for *Ochrobactrum haematophilum* (Kämpfer et al., 2007a). The type strain is CCUG 38531 = CIP 109452 = DSM 22355.

#### Description of *Brucella intermedia* comb. nov.

B. in.ter.me’di.a (L. fem. adj. *intermedia*, that is between, intermediate).

Basonym: *Ochrobactrum intermedium* Velasco et al., 1998

The description is as given for *Ochrobactrum intermedium* (Velasco et al., 1998). The type strain is CCUG 24694 = CIP 105838 = DSM 17986 = IFO 15820 = LMG 3301 = NBRC 15820 = NCTC 12171.

#### Description of *Brucella lupini* comb. nov.

B. lu.pi’ni (L. gen. n. *lupini*, of a lupine, referring to the isolation source of this microorganism, nodules of *Lupinus albus*).

Basonym: *Ochrobactrum lupini* Trujillo et al. 2006.

The description is as given for *Ochrobactrum lupini* (Trujillo et al., 2005). The type strain is DSM 16930 = LMG 22726 = NBRC 102587.

#### Description of *Brucella oryzae* comb. nov.

B. o.ry’zae (L. gen. n. *oryzae*, of rice, pertaining to the habitat from which the first strains were isolated).

Basonym: *Ochrobactrum oryzae* Tripathi et al., 2006

The description is as given for *Ochrobactrum oryzae* (Tripathi et al., 2006). The type strain is DSM 17471 = MTCC 4195 = NBRC 102588.

#### Description of *Brucella pecoris* comb. nov.

B. pe.co’ris (L. gen. n. *pecoris*, of livestock).

Basonym: *Ochrobactrum pecoris* Kämpfer et al., 2011

The description is as given for *Ochrobactrum pecoris* (Kämpfer et al., 2011). The type strain is CCM 7822 = CCUG 60088 = DSM 23868.

#### Description of *Brucella pituitosa* comb. nov.

B. pi.tu.i.to’sa (L. fem. adj. *pituitosa*, full of phlegm, *pituilous*, intended to mean slimy, referring to the consistency of the colonies after extended incubation).

Basonym: *Ochrobactrum pituitosum* Huber et al., 2010

The description is as given for *Ochrobactrum pituitosum* (Huber et al., 2010) with the following addition. The G+C content of the type-strain genome is 53.7%, its approximate size 4.28 Mbp. The type strain is CCUG 50899 = DSM 22207.

#### Description of *Brucella pseudintermedia* comb. nov.

B. pseud.in.ter.me’di.a (Gr. neut. adj. *pseudês*, false; L. fem. adj. *intermedia*, intermediate, and a specific epithet of the genus *Brucella*; N.L. fem. adj. *pseudintermedia*, a false *Brucella intermedia*).

Basonym: *Ochrobactrum pseudintermedium* Teyssier et al., 2007

The description is as given for *Ochrobactrum pseudintermedium* (Teyssier et al., 2007). The genomic G+C content of the type strain is 54.0%. Its approximate genome size is 5.53 Mbp. The type strain is CIP 109116 = DSM 17490.

#### Description of *Brucella pseudogrignonensis* comb. nov.

B. pseu.do.gri.gnon.en’sis (Gr. neut. adj. *pseudês*, false; N.L. fem. adj. *grignonensis*, a bacterial species epithet; N.L. fem. adj. *pseudogrignonensis*, a false *Brucella grignonensis*).

Basonym: *Ochrobactrum pseudogrignonense* Kämpfer et al., 2007a

The description is as given for *Ochrobactrum pseudogrignonense* (Kämpfer et al., 2007a) with the following addition. The G+C content of the type-strain genome is 54.0%, its approximate size 5.53 Mbp. The type strain is CCUG 30717 = CIP 109451 = DSM 22354.

#### Description of *Brucella rhizosphaerae* comb. nov.

B. rhi.zo.sphae’rae (Gr. fem. n. *rhiza*, root; L. fem. n. *sphaera*, a ball, sphere; N.L. n. *rhizosphaera*, rhizosphere; N.L. gen. n. *rhizosphaerae*, of the rhizosphere).

Basonym: *Ochrobactrum rhizosphaerae* Kämpfer et al., 2008

The description is as given for *Ochrobactrum rhizosphaerae* (Kämpfer et al., 2008). The genomic G+C content of the type strain is 53.0%. Its approximate genome size is 4.90 Mbp. The type strain is CCM 7493 = CCUG 55411 = DSM 19824.

#### Description of *Brucella thiophenivorans* comb. nov.

B. thi.o.phe.ni.vo’rans (N.L. neut. n. *thiophenum*, thiophene; L. pres. part. *vorans*, devouring; N.L. part. adj. *thiophenivorans*, thiophene-devouring, referring to the ability to utilize thiophene 2-carboxylate as a sole source of carbon and sulfur).

Basonym: *Ochrobactrum thiophenivorans* Kämpfer et al., 2008

The description is as given for *Ochrobactrum thiophenivorans* (Kämpfer et al., 2008). The genomic G+C content of the type strain is 51.6%. Its approximate genome size is 4.36 Mbp. The type strain is CCM 7492 = CCUG 55412 = DSM 7216.

#### Description of *Brucella tritici* comb. nov.

B. tri.ti’ci (L. neut. n. *triticum*, wheat, and also the generic name for wheat, *Triticum*; L. gen. n. *tritici*, of wheat, of *Triticum*, from which the strains were isolated).

Basonym: *Ochrobactrum tritici* Lebuhn et al., 2000

The description is as given for *Ochrobactrum tritici* (Lebuhn et al., 2000). The type strain is CCUG 47104 = DSM 13340 = LMG 18957 = NBRC 102585.

#### Description of *Caenibius tardaugens* comb. nov.

C. tard.au’gens (L. masc. adj. *tardus*, slow; L. pres. part. *augens*, growing; N.L. part. adj. *tardaugens*, slowly growing).

Basonym: *Novosphingobium tardaugens* Fujii et al., 2003

The description is as given for *Novosphingobium tardaugens* (Fujii et al., 2003). The type strain is ARI-1 = DSM 16702 = JCM 11434.

#### Description of *Celeribacter arenosi* comb. nov.

C. a.re.no’si (L. gen. n. *arenosi*, of a sandy place, dwelling in marine sand).

Basonym: *Vadicella arenosi* Romanenko et al., 2011c

The description is as given for *Vadicella arenosi* (Romanenko et al., 2011c). The type strain is JCM 17190 = KMM 9024 = NRIC 0787.

#### Description of *Cereibacter azotoformans* comb. nov.

C. a.zo.to.for’mans (N.L. neut. n. *azotum*, (from Fr. n. *azote*), nitrogen; L. pres. part. *formans*, forming; N.L. part. adj. *azotoformans*, nitrogen forming).

Basonym: *Rhodobacter azotoformans* Hiraishi et al. 1997.

The description is as given for *Rhodobacter azotoformans* (Hiraishi et al., 1996) with the following modification. The G+C content of the type-strain genome is 68.4%, its approximate size 4.41 Mbp. The type strain is KA25 = JCM 9340 = NBRC 16436.

#### Description of *Cereibacter johrii* comb. nov.

C. joh’ri.i (N.L. gen. n. *johrii*, of B. N. Johri, an eminent and well-known Indian microbiologist).

Basonym: *Rhodobacter johrii* Girija et al., 2010

The description is as given for *Rhodobacter johrii* (Girija et al., 2010). The genomic G+C content of the type strain is 69.1%. Its approximate genome size is 4.51 Mbp. The type strain is JA192 = DSM 18678 = JCM 14543.

#### Description of *Cereibacter ovatus* comb. nov.

C. o.va’tus (L. masc. adj. *ovatus*, egg-shaped, ovate).

Basonym: *Rhodobacter ovatus* Srinivas et al., 2008

The description is as given for *Rhodobacter ovatus* (Srinivas et al., 2008) with the following modification. The G+C content of the type-strain genome is 66.5%, its approximate size 3.81 Mbp. The type strain is JA234 = CCUG 55049 = JCM 14779.

#### Description of *Cereibacter sphaeroides* comb. nov.

C. sphae.ro.i’des (L. fem. n. *sphaera*, sphere, globe; L. suff. -*oides*, from Greek suffix *eides*, from Greek noun *eidos*, that which is seen, form, shape, figure, resembling, similar; N.L. masc. adj. *sphaeroides*, spherical).

Basonym: *Rhodopseudomonas sphaeroides* van Niel, 1944 (Approved Lists 1980)

The description is as given for *Rhodobacter sphaeroides* (Imhoff et al., 1984). The genomic G+C content of the type strain is 68.8%. Its approximate genome size is 4.60 Mbp. The type strain is CECT 300 = DSM 158 = JCM 6121.

#### Description of *Cypionkella aquatica* comb. nov.

C. a.qua’ti.ca (L. fem. adj. *aquatica*, growing or found in water).

Basonym: *Pseudorhodobacter aquaticus* Li L. et al., 2016

The description is as given for *Pseudorhodobacter aquaticus* (Li A.-H. et al., 2016). The type strain is DC2N1-10 = CGMCC 1.14433 = KCTC 52040.

#### Description of *Cypionkella collinsensis* comb. nov.

C. col.lins.en’sis (N.L. fem. adj. *collinsensis*, pertaining to Collins, an icecap of Antarctic, from where the type strain was isolated).

Basonym: *Pseudorhodobacter collinsensis* Zhang et al., 2016

The description is as given for *Pseudorhodobacter collinsensis* (Zhang et al., 2016). The type strain is 4-T-34 = CCTCC AB 2014005 = LMG 28256.

#### Description of *Cypionkella psychrotolerans* comb. nov.

C. psy.chro.to’le.rans (Gr. masc. adj. *psychros*, cold; L. pres. part. *tolerans*, tolerating; N.L. part. adj. *psychrotolerans*, cold-tolerating).

Basonym: *Pseudorhodobacter psychrotolerans* Lee et al., 2016

The description is as given for *Pseudorhodobacter psychrotolerans* (Lee et al., 2016). The type strain is JCM 30764 = KCTC 42640.

#### Description of *Cypionkella sinensis* comb. nov.

C. sin.en’sis (N.L. fem. adj. *sinensis*, pertaining to China, referring to the geographical origin of the type strain).

Basonym: *Pseudorhodobacter sinensis* Li L. et al., 2016

The description is as given for *Pseudorhodobacter sinensis* (Li A.-H. et al., 2016). The type strain is Y1R2-4 = CGMCC 1.14435 = KCTC 52039.

#### Description of *Elkelangia baekryungensis* comb. nov.

L. baek.ryung.en’sis (N.L. fem. adj. *baekryungensis*, of Baekryung Island, an island of the Yellow Sea in Korea where the type strain was isolated).

Basonym: *Sphingopyxis baekryungensis* Yoon et al., 2005a

The description is as given for *Sphingopyxis baekryungensis* (Yoon et al., 2005a). The type strain is DSM 16222 = KCTC 12231.

#### Description of *Magnetospirillum chandramohanii* comb. nov.

M. chan.dra.mo.han’i.i (N.L. gen. n. *chandramohanii*, of Chandramohan, named after Dr. D. Chandramohan, an Indian marine microbiologist, who has played a crucial role in transforming microbiological research at the National Institute of Oceanography, India, into technologically rewarding activities).

Basonym: *Phaeospirillum chandramohanii* Anil Kumar et al., 2009

The description is as given for *Phaeospirillum chandramohanii* (Anil Kumar et al., 2009). The type strain is JA145 = JCM 14933 = KCTC 5703.

#### Description of *Magnetospirillum fulvum* comb. nov.

M. ful’vum (L. neut. adj. *fulvum*, deep yellow, reddish yellow, tawny).

Basonym: *Rhodospirillum fulvum* van Niel, 1944 (Approved Lists 1980)

The description is as given for *Phaeospirillum fulvum* (Imhoff et al., 1998). The type strain is NCIMB 11762 = ATCC 15798 = DSM 113.

#### Description of *Magnetospirillum molischianum* comb. nov.

M. mo.lisch.i.a’num (N.L. neut. adj. *molischianum*, pertaining to Molisch, named for H. Molisch, an Austrian botanist).

Basonym: *Rhodospirillum molischianum* Giesberger, 1947 (Approved Lists 1980)

The description is as given for *Phaeospirillum molischianum* (Imhoff et al., 1998). The genomic G+C content of the type strain is 61.5%. Its approximate genome size is 3.81 Mbp. The type strain is ATCC 14031 = DSM 120 = LMG 4354.

#### Description of *Magnetospirillum oryzae* comb. nov.

M. o.ry’zae (L. gen. n. *oryzae*, of rice, pertaining to the isolation of the type strain from rice paddy soil).

Basonym: *Phaeospirillum oryzae* Lakshmi et al., 2011

The description is as given for *Phaeospirillum oryzae* (Lakshmi et al., 2011). The type strain is JA317 = KCTC 5704 = NBRC 104938.

#### Description of *Magnetospirillum tilakii* comb. nov.

M. ti.la’ki.i (N.L. gen. n. *tilakii*, of Tilak, named after Dr. K. V. B. R. Tilak, an eminent microbiologist in India).

Basonym: *Phaeospirillum tilakii* Raj et al., 2012

The description is as given for *Phaeospirillum tilakii* (Raj et al., 2012). The type strain is JA492 = KCTC 15012 = NBRC 107650.

#### Description of *Meinhardsimonia xiamenensis* comb. nov.

M. xia.men.en’sis (N.L. fem. adj. *xiamenensis*, of or pertaining to Xiamen, the city where the organism was first isolated).

Basonym: *Albidovulum xiamenense* Yin et al., 2012

The description is as given for *Albidovulum xiamenense* (Yin et al., 2012). The genomic G+C content of the type strain is 68.2%. Its approximate genome size is 3.13 Mbp. The type strain is CGMCC 1.10789 = DSM 24422 = LMG 26247 = MCCC 1A06317.

#### Description of *Mycoplana subbaraonis* comb. nov.

M. sub.ba.ra.o’nis (N.L. gen. n. *subbaraonis*, of Subba Rao, named after Professor N. S. Subba Rao, an eminent microbiologist who significantly contributed to the knowledge of *Rhizobium* biofertilizers in India).

Basonym: *Rhizobium subbaraonis* Ramana V.V. et al., 2013

The description is as given for *Rhizobium subbaraonis* (Ramana C.V. et al., 2013) with the following modification. The G+C content of the type-strain genome is 63.1%, its approximate size 6.58 Mbp. The type strain is DSM 24765 = KCTC 23614.

#### Description of *Neopararhizobium haloflavum* comb. nov.

N. ha.lo.fla’vum (Gr. masc. n. *hals*/*halos*, salt; L. masc. adj. *flavus*, yellow; N.L. neut. adj. *haloflavum*, salty and yellow).

Basonym: *Pararhizobium haloflavum* Shen et al., 2018

The description is as given for *Pararhizobium haloflavum* (Shen et al., 2018). The type strain is KCTC 52582 = MCCC 1K03228.

#### Description of *Neorhizobium vignae* comb. nov.

N. vi’gnae (N.L. gen. n. *vignae*, of *Vigna*, referring to the fact that the majority of strains were isolated from the mung bean, *Vigna radiata*).

Basonym: *Rhizobium vignae* Ren et al., 2011

The description is as given for *Rhizobium vignae* (Ren et al., 2011). The type strain is HAMBI 3039 = DSM 25378 = LMG 25447.

#### Description of *Niveispirillum irakense* comb. nov.

N. i.rak.en’se (N.L. neut. adj. *irakense*, pertaining to the country of Iraq).

Basonym: *Azospirillum irakense* Khammas et al. 1991.

The description is as given for *Azospirillum irakense* (Khammas et al., 1989). The genomic G+C content of the type strain is 63.0%. Its approximate genome size is 5.45 Mbp. The type strain is KBC1 = ATCC 51182 = DSM 11586.

#### Description of *Pacificitalea manganoxidans* comb. nov.

P. man.gan.o’xi.dans (N.L. neut. n. *manganum*, manganese; N.L. pres. part. *oxidans*, oxidizing; N.L. part. adj. *manganoxidans*, manganese-oxidizing).

Basonym: *Celeribacter manganoxidans* Wang Y.X. et al., 2015

The description is as given for *Celeribacter manganoxidans* (Wang L. et al., 2015). The type strain is DY2-5 = DSM 27541 = JCM 19384.

#### Description of *Pelagimonas phthalicica* comb. nov.

P. phtha.li’ci.ca (N.L. neut. n. *acidum phthalicum*, phthalic acid; L. fem. suff. -*ica*, suffix used with the sense of belonging to; N.L. fem. adj. *phthalicica*, belonging to phthalic acid, referring to the substrate *phthalic* acid that can be utilized by the species).

Basonym: *Tropicibacter phthalicicus* Iwaki et al., 2012

The description is as given for *Tropicibacter phthalicicus* (Iwaki et al., 2012). The type strain is DSM 26923 = JCM 17793 = KCTC 23703.

#### Description of *Pseudoprimorskyibacter insulae* comb. nov.

P. in’su.lae (L. gen. n. *insulae*, of an island, referring to the source of isolation of the type strain).

Basonym: *Primorskyibacter insulae* Parker et al., 2019

The description is as given for *Primorskyibacter insulae* (Park et al., 2015a). The type strain is CECT 8871 = KCTC 42602.

#### Description of *Pseudovibrio exalbescen*s comb. nov.

P. ex.al.bes’cens (L. part. adj. *exalbescens*, (from L. v. *exalbesco*) becoming white, growing white, referring to the fading color of maturing colonies).

Basonym: *Nesiotobacter exalbescens* Donachie et al., 2006

The description is as given for *Nesiotobacter exalbescens* (Donachie et al., 2006) with the following modification. The G+C content of the type-strain genome is 55.1%, its approximate size 4.15 Mbp. The type strain is LA33B = DSM 16456 = ATCC BAA-994.

#### Description of *Rhizorhabdus histidinilytica* comb. nov.

R. his.ti.di.ni.ly’ti.ca (N.L. neut. n. *histidinum*, histidine; N.L. fem. adj. lytica, from Greek fem. adj. *lytikê*, able to loose, able to dissolve; N.L. fem. adj. *histidinilytica*, histidine-dissolving).

Basonym: *Sphingomonas histidinilytica* Nigam et al., 2010

The description is as given for *Sphingomonas histidinilytica* (Nigam et al., 2010). The type strain is CCM 7545 = DSM 24951 = MTCC 9473.

#### Description of *Rhizorhabdus starnbergensis* comb. nov.

R. starn.berg.en’sis (N.L. fem. adj. *starnbergensis*, of or pertaining to Lake Starnberg, Bavaria, Germany, from where the organism was isolated).

Basonym: *Sphingomonas starnbergensis* Chen W.-M. et al., 2013

The description is as given for *Sphingomonas starnbergensis* (Chen H. et al., 2013). The type strain is DSM 25077 = LMG 26763.

#### Description of *Rhizorhabdus wittichii* comb. nov.

R. wit.tich.i’i (N.L. gen. n. *wittichii*, of Wittich, referring to Rolf-Michael Wittich, the German bacteriologist who first isolated this potent metabolizer of dibenzo-p-dioxin from the water of the river Elbe and described the metabolism of the compound by this organism).

Basonym: *Sphingomonas wittichii* Yabuuchi et al., 2001

The description is as given for *Sphingomonas wittichii* (Kim M.C. et al., 2016). The type strain is CCUG 31198 = DSM 6014 = JCM 10273.

#### Description of *Roseibium aggregatum* comb. nov.

R. ag.gre.ga’tum (L. neut. adj. *aggregatum*, joined together).

Basonym: *Stappia aggregata* (ex Ahrens 1968) Uchino et al. 1999.

The description is as given for *Labrenzia aggregata* (Biebl et al., 2007). The type strain is ATCC 25650 = DSM 13394 = IFO 16684 = JCM 20685 = LMG 122 = NBRC 16684 = NCIMB 2208.

#### Description of *Roseibium album* comb. nov.

R. al’bum (L. neut. adj. *album*, white).

Basonym: *Stappia alba* Pujalte et al. 2006.

The description is as given for *Labrenzia alba* (Biebl et al., 2007) with the following addition. The genomic G+C content of the type strain is 56.4%. Its approximate genome size is 6.90 Mbp. The type strain is CECT 5095 = CIP 108402 = DSM 18320 = DSM 18380.

#### Description of *Roseibium alexandrii* comb. nov.

R. a.le.xan’dri.i (N.L. gen. n. *alexandrii*, of *Alexandrium*, the genus name of the dinoflagellate *Alexandrium lusitanicum*, the source of isolation of the type strain).

Basonym: *Labrenzia alexandrii* Biebl et al., 2007

The description is as given for *Labrenzia alexandrii* (Biebl et al., 2007). The type strain is DSM 17067 = NCIMB 14079.

#### Description of *Roseibium marinum* comb. nov.

R. ma.ri’num (L. neut. adj. *marinum*, of the sea, marine).

Basonym: *Stappia marina* Kim et al. 2006.

The description is as given for *Labrenzia marina* (Biebl et al., 2007). The type strain is DSM 17023 = KCTC 12288.

#### Description of *Roseibium salinum* comb. nov.

R. sa.li’num (N.L. neut. adj. *salinum*, salted, referring to the saline habitat of the micro-organism).

Basonym: *Labrenzia salina* Camacho et al., 2016

The description is as given for *Labrenzia salina* (Camacho et al., 2016). The type strain is CECT 8816 = DSM 29163.

#### Description of *Roseibium suaedae* comb. nov.

R. su.ae’dae (N.L. gen. n. *suaedae*, of the plant *Suaeda corniculata*, referring to the isolation of the type strain from the roots of *Suaeda corniculata*).

Basonym: *Labrenzia suaedae* Bibi et al., 2014

The description is as given for *Labrenzia suaedae* (Bibi et al., 2014) with the following modification. The G+C content of the type-strain genome is 60.2%, its approximate size 5.14 Mbp. The type strain is DSM 22153 = KACC 13772.

#### Description of *Roseovarius litorisediminis* comb. nov.

R. li.to.ri.se.di’mi.nis (L. neut. n. *litus/-oris*, the seashore, coast; L. n. *sedimen/-inis*, sediment; N.L. gen. n. *litorisediminis*, of a coastal sediment, tidal flat sediment).

Basonym: *Pelagicola litorisediminis* Park et al. 2013.

The description is as given for *Pelagicola litorisediminis* (Park et al., 2013a). The type strain is D1-W8 = CECT 8287 = KCTC 32327.

#### Description of *Ruegeria meteori* comb. nov.

R. me.te’or.i (N.L. gen. n. *meteori*, of meteor, after the German research vessel Meteor).

Basonym: *Agrobacterium meteori* Rüger and Höfle, 1992

*Agrobacterium meteori* (Rüger and Höfle, 1992) with the following modification. The G+C content of the type-strain genome is 56.5%, its approximate size 4.83 Mbp. The type strain is ATCC 700001 = CECT 4293 = DSM 5824.

#### Description of *Salipiger pacificus* comb. nov.

S. pa.ci’fi.cus (L. masc. adj. *pacificus*, peacemaking, pacific, and by extension pertaining to the Pacific Ocean, the origin of the type strain).

Basonym: *Yangia pacifica* Dai et al., 2006

The description is as given for *Yangia pacifica* (Dai et al., 2006). The genomic G+C content of the type strain is 66.3%. Its approximate genome size is 6.14 Mbp. The type strain is CGMCC 1.3455 = DSM 26894 = JCM 12573.

#### Description of *Tabrizicola blastica* comb. nov.

T. bla’sti.cus (Gr. adj. *blastikos/-ê/-on*, budding, sprouting; N.L. fem. adj. *blastica*, budding, apt to bud).

Basonym: *Rhodopseudomonas blastica* Eckersley and Dow 1981.

The description is as given for *Rhodobacter blasticus* (Kawasaki et al., 1993). The type strain is ATCC 33485 = CIP 104374 = DSM 2131 = DSM 26431 = IFO 16437 = LMG 4305 = NBRC 16437 = NCIMB 11576.

#### Description of *Thalassobius litoralis* comb. nov.

T. li.to.ra’lis (L. masc. adj. *litoralis*, of or belonging to the seashore, referring to the supralitoral habitat from which the type strain was isolated).

Basonym: *Lutimaribacter litoralis* Iwaki et al., 2013

The description is as given for *Lutimaribacter litoralis* (Iwaki et al., 2013). The type strain is DSM 29506 = JCM 17792 = KCTC 23660.

#### Description of *Thalassobius taeanensis* comb. nov.

T. tae.an.en’sis (N.L. masc. adj. *taeanensis*, of or belonging to Taean, from where the organism was isolated).

Basonym: *Litorimicrobium taeanense* Jin et al., 2011

The description is as given for *Litorimicrobium taeanense* (Jin et al., 2011). The genomic G+C content of the type strain is 60.5%. Its approximate genome size is 4.02 Mbp. The type strain is DSM 22007 = KACC 13706.

#### Description of *Tranquillimonas rosea* comb. nov.

T. ro’se.a (L. fem. adj. *rosea*, rose-colored, pink).

Basonym: *Roseivivax roseus* Zhang et al., 2014a.

The description is as given for *Roseivivax roseus* (Zhang et al., 2014c) with the following modification. The G+C content of the type-strain genome is 67.8%, its approximate size 4.23 Mbp. The type strain is BH87090 = DSM 23042 = KCTC 22650.

#### Description of *Tritonibacter mobilis* comb. nov.

T. mo.bi’lis (L. masc. adj. *mobilis*, movable, motile).

Basonym: *Ruegeria mobilis* Muramatsu et al., 2007

The description is as given for *Epibacterium mobile* (Wirth and Whitman, 2018). The type strain is CIP 109181 = DSM 23403 = NBRC 101030.

#### Description of *Tritonibacter multivorans* comb. nov.

T. mul.ti.vo’rans (L. masc. adj. *multus*, many, numerous; L. v. *vorare*, to devour, swallow; N.L. part. adj. *multivorans*, devouring many, referring to the utilization of numerous different substrates for growth).

Basonym: *Tropicibacter multivorans* Lucena et al., 2012

The description is as given for *Epibacterium multivorans* (Wirth and Whitman, 2018). The genomic G+C content of the type strain is 59.7%. Its approximate genome size is 4.15 Mbp. The type strain is CECT 7557 = DSM 26470 = KCTC 23350.

#### Description of *Tritonibacter scottomollicae* comb. nov.

T. scot.to.mol’li.cae (N.L. gen. n. *scottomollicae*, of Scotto-Mollica, in honor of Dr. Victoria Scotto-Mollica and Dr. Alfonso Mollica, both of whom were pioneers in the field of microbe-induced corrosion of steels and the generation of electroactive seawater biofilms).

Basonym: *Ruegeria scottomollicae* Vandecandelaere et al. 2008

The description is as given for *Epibacterium scottomollicae* (Wirth and Whitman, 2018). The type strain is CCUG 55858 = DSM 25328 = LMG 24367.

#### Description of *Tritonibacter ulvae* comb. nov.

T. ul’vae (N.L. gen. n. *ulvae*, of *Ulva*, the name of a genus of green algae, as the type strain was isolated from a frond surface of *Ulva pertusa* Kjellman).

Basonym: *Epibacterium ulvae* Penesyan et al., 2013

The description is as given for *Epibacterium ulvae* (Penesyan et al., 2013). The type strain is DSM 24752 = LMG 26464.

#### Description of *Vannielia litorea* comb. nov.

V. li.to.re’a (L. fem. adj. *litorea*, living near the sea, of or belonging to the seashore).

Basonym: *Oceanicola litoreus* Park et al., 2013a

The description is as given for *Oceanicola litoreus* (Park et al., 2013c). The type strain is CCUG 62794 = DSM 29440 = KCTC 32083.

#### Description of *Wagnerdoeblera megaterium* comb. nov.

W. me.ga.te’ri.um (Gr. masc. adj. *megas*, large; Gr. neut. n. *teras/-atos*, monster, beast; N.L. neut. n. *megaterium*, big beast).

Basonym: *Gemmobacter megaterium* Liu K. et al., 2014

The description is as given for *Gemmobacter megaterium* (Liu J.-J. et al., 2014) with the following modification. The G+C content of the type-strain genome is 64.9%, its approximate size 4.17 Mbp. The type strain is CF17 = DSM 26375 = JCM 18498.

#### Description of *Wagnerdoeblera nectariphila* comb. nov.

W. nec.ta.ri’phi.la (L. n. *nectar*, nectar; N.L. fem. adj. *phila*, friend, loving (from Gr. masc. adj. *philos*); N.L. fem. adj. *nectariphila*, loving nectar, referring to the stimulation of growth by excretions of other bacteria).

Basonym: *Gemmobacter nectariphilus* (Tanaka et al., 2004) Chen W.-M. et al., 2013

The description is as given for *Gemmobacter nectariphilus* (Chen W.-M. et al., 2013) with the following modification. The G+C content of the type-strain genome is 66.2%, its approximate size 4.52 Mbp. The type strain is AST4 = DSM 15620 = JCM 11959.

### Taxonomic Consequences: New Subspecies

#### Description of *Borrelia garinii* subsp. *bavariensis* subsp. nov.

B. ga.ri’ni.i subsp. ba.va.ri.en’sis (N.L. fem. adj. *bavariensis*, of or belonging to Bavaria, from where the type strain was isolated).

The description is as given for *Borrelia bavariensis* (Margos et al., 2013). The type strain is PBi = ATCC BAA-2496 = DSM 23469.

#### Description of *Gluconobacter japonicus* subsp. *nephelii* subsp. nov.

G. ja.po.ni’cus subsp. ne.phe’li.i (N.L. neut. n. *Nephelium*, the generic name of rambutan, *Nephelium lappaceum*, a tropical fruit; N.L. gen. n. *nephelii*, of *Nephelium*, from which the type strain was isolated).

The description is as given for *Gluconobacter nephelii* (Kommanee et al., 2011). The type strain is RBY-1 = BCC 36733 = NBRC 106061.

#### Description of *Methylobacterium dichloromethanicum* subsp. *chloromethanicum* subsp. nov.

M. di.chlo.ro.me.tha’ni.cum subsp. chlo.ro.me.tha’ni.cum (N.L. neut. n. *chloromethanicum*, chloromethane-utilizing).

The description is as given for *Methylobacterium chloromethanicum* (McDonald et al., 2001). The type strain is CM4 = NCIMB 13688 = VKM B-2223.

#### Description of *Rhizobium marinum* subsp. *pelagicum* subsp. nov.

R. ma.ri’num subsp. pe.la.gi’cum (L. neut. adj. *pelagicum*, of or belonging to the sea).

The description is as given for *Pseudorhizobium pelagicum* (Kimes et al., 2015). The type strain is R1-200B4 = CECT 8629 = LMG 28314.

#### Description of *Rhizobium mongolense* subsp. *loessense* subsp. nov.

R. mon.go.len’se subsp. loess.en’se (N.L. neut. adj. *loessense*, referring to the Loess Plateau of China, where the bacterium was isolated).

The description is as given for *Rhizobium loessense* (Wei et al., 2003). The type strain is CGMCC 1.3401 = CIP 108030 = LMG 21975.

#### Description of *Rickettsia conorii* subsp. *gravesii* subsp. nov.

R. co.no’ri.i subsp. gra.ves’i.i (N.L. gen. n. *gravesii*, of Graves, named after Professor Stephen Graves, founder of the Australian Rickettsial Reference Laboratory and a major contributor to rickettsial research in Australia).

The description is as given for *Rickettsia gravesii* (Abdad et al., 2017). The type strain is BWI-1 = ATCC VR-1664 = CSUR R172.

#### Description of *Rickettsia conorii* subsp. *heilongjiangensis* subsp. nov.

R. co.no’ri.i subsp. hei.long.jiang.en’sis (N.L. fem. adj. *heilongjiangensis*, from Heilongjiang, the Chinese province where the *D. silvarum* tick providing the first isolate was collected).

The description is as given for *Rickettsia heilongjiangensis* (Fournier et al., 2003). The genomic G+C content of the type strain is 32.3%. Its approximate genome size is 1.28 Mbp. The type strain is 054 = ATCC VR-1524 = CSUR 054.

#### Description of *Rickettsia conorii* subsp. *raoultii* subsp. nov.

R. co.no’ri.i subsp. ra.oult’i.i (N.L. gen. n. *raoultii*, of Raoult, named after Professor Didier Raoult, founder of the WHO-Collaborative Centre for Rickettsioses, Borrelioses and Tick-borne Infections in Marseilles, France, and a major contributor to the study of rickettsiae).

The description is as given for *Rickettsia raoultii* (Mediannikov et al., 2008). The type strain is Khabarovsk = ATCC VR-1596 = CSUR R3.

#### Description of *Rickettsia tamurae* subsp. *buchneri* subsp. nov.

R. ta.mu’rae subsp. buch’ner.i (N.L. gen. n. *buchneri*, of Buchner, named in honor of Dr. Paul Buchner, a German biologist who made pioneering contributions to the identification of non-pathogenic tick endosymbionts that are transovarially transmitted).

The description is as given for *Rickettsia buchneri* (Kurtti et al., 2015). The type strain is ISO-7 = DSM 29016 = ATCC VR-1814.

#### Description of *Tritonibacter mobilis* subsp. *pelagius* subsp. nov.

T. mo’bi.lis subsp. pe.la’gi.us (L. masc. adj. *pelagius*, of the sea).

The description is as given for *Ruegeria pelagia* (Lee et al., 2007c). The type strain is HTCC2662 = KCCM 42378 = NBRC 102038.

### Taxonomic Consequences: Emendations of Orders

#### Emended Description of *Hyphomicrobiales* Douglas et al. 1957 (Approved Lists 1980)

The description is as given for *Rhizobiales* (Kuykendall, 2005), which is an illegitimate synonym, with the following additions. The order consists of the families *Ancalomicrobiaceae, Aurantimonadaceae*, *Bartonellaceae*, *Beijerinckiaceae*, *Nitrobacteraceae*, *Brucellaceae*, *Chelatococcaceae*, *Cohaesibacteraceae*, *Hyphomicrobiaceae*, *Methylobacteriaceae*, *Methylocystaceae*, *Notoacmeibacteraceae*, *Phyllobacteriaceae*, *Rhizobiaceae*, *Rhodobiaceae*, *Xanthobacteraceae*. In addition to the new families *Acuticoccaceae* fam. nov., *Afifellaceae* fam nov., *Ahrensiaceae* fam. nov., *Amorphaceae* fam. nov., *Blastochloridaceae* fam. nov., *Breoghaniaceae* fam. nov., *Devosiaceae* fam. nov., *Kaistiaceae* fam. nov., *Parvibaculaceae* fam. nov., *Phreatobacteraceae* fam. nov., *Pleomorphomonadaceae* fam. nov., *Pseudoxanthobacteraceae* fam. nov., *Stappiaceae* fam. nov. and *Tepidamorphaceae* fam. nov. The type genus is *Hyphomicrobium*.

#### Emended Description of Kordiimonadales Kwon et al. 2005

The description is as given before (Kwon et al., 2005) with the following modifications. Cells are Gram-negative, rod-shaped, motile, aerobic heterotrophs. This order houses *Kordiimonadaceae* fam. nov., which is currently the sole family of the order. The type genus is *Kordiimonas*.

#### Emended Description of *Rhodobacterales* Garrity et al. 2006

The description is as given before (Garrity et al., 2005d) with the following modifications. This order houses *Neomegalonemataceae* fam. nov. in addition to the previously included families. The type genus is *Rhodobacter*.

#### Emended Description of *Rhodospirillales* Pfennig and Trüper 1971 (Approved Lists 1980)

The description is as given before (Pfennig and Trüper, 1971) with the following modification. The order contains the families *Acetobacteraceae and Kiloniellaceae.* In addition to the new families *Azospirillaceae*, fam. nov., *Reyranellaceae* fam. nov., *Rhodospirillaceae*, *Rhodovibrionaceae*, fam. nov., *Stellaceae* fam. nov., *Terasakiellaceae* fam. nov., *Thalassobaculaceae* fam. nov., *Thalassospiraceae* fam. nov. and *Zavarziniaceae* fam. nov. The order contains heterotrophs as well as autotrophs. The type genus is *Rhodospirillum.*

#### Emended Description of *Sphingomonadales* Yabuuchi and Kosako 2006

The description is as given before (Garrity et al., 2005d) with the following modification. This order houses *Zymomonadaceae*, fam. nov. and *Sphingosinicellaceae*, fam. nov., in addition to the previously included families. The type genus is *Sphingomonas*.

#### Emended Description of *Spirochaetales* Buchanan 1917 (Approved Lists 1980) emend. Gupta et al., 2013

The description is as given before (Gupta et al., 2013) with the following modification. This order contains the families *Sphaerochaetaceae*, *Spirochaetaceae*, and *Treponemataceae*. The order comprises motile as well as non-motile bacteria. The type genus is *Spirochaeta*.

### Taxonomic Consequences: Emendations of Families

#### Emended Description of *Acetobacteraceae*(ex Henrici 1939) Gillis and De Ley 1980

The description is as given before (Gillis and De Ley, 1980) with the following modification. This family houses *Acidicaldus*, *Gluconacetobacter*, *Neoasaia*, *Roseococcus*, *Swaminathania*, *Rubritepida*, *Saccharibacter*, *Swingsia*, *Teichococcus*, *Dankookia*, *Crenalkalicoccus*, *Acetobacter*, *Ameyamaea*, *Asaia*, *Gluconobacter*, *Granulibacter*, *Humitalea*, *Kozakia*, *Muricoccus*, *Paracraurococcus*, *Tanticharoenia*, *Acidiphilium*, *Acidisoma*, *Acidisphaera*, *Acidocella*, *Acidomonas*, *Belnapia*, *Bombella*, *Craurococcus*, *Endobacter*, *Komagataeibacter*, *Neokomagataea*, *Nguyenibacter*, *Rhodopila*, *Rhodovarius*, *Roseomonas* (including *Muricoccus* and *Teichococcus*), *Caldovatus*, *Elioraea*, *Siccirubricoccus and Rhodovastum*. The type genus is *Acetobacter*.

#### Emended Description of *Beijerinckiaceae* Garrity et al. 2006 emend. Dedysh et al. 2016

The description is as given before (Garrity et al., 2005d; Dedysh et al., 2016), with the following modification. This family houses *Beijerinckia* (the type genus), *Methylocapsa*, *Methylocella*, *Methyloferula*, *Methylorosula*, and *Methylovirgula*.

#### Emended Description of *Cohaesibacteraceae* Hwang and Cho 2018 emend. Gallego et al. 2010

The description is as given before (Hwang and Cho, 2008a; Gallego et al., 2010) with the following modification. This family houses *Cohaesibacter*, which is the type and currently the sole genus of the family.

#### Emended Description of *Ehrlichiaceae* Moshkovski 1945 (Approved Lists 1980)

The description is as given for *Anaplasmataceae* (Philip, 1957; Dumler et al., 2001) with the following additions. This family houses *Anaplasma*, *Ehrlichia* (the type genus), *Lyticum and Neorickettsia.* The genera *Aegyptianella, Cowdria* and *Wolbachia* (for which cultures and 16S rRNA gene sequences of type strains are missing) are also tentatively assigned to this family.

#### Emended Description of *Erythrobacteraceae* Lee et al. 2005 emend. Xu et al. 2009

The description is as given before (Lee et al., 2005; Xu X.-W. et al., 2009) with the following modification. Some genera of this family contain carotenoids and bacteriochlorophyll α. This family houses *Novosphingobium*, in addition to the previously included genera. The type genus is *Erythrobacter*.

#### Emended Description of *Geminicoccaceae* Proença et al. 2018

The description is as given before (Proença et al., 2018) with the following modification. Cells are cocci, diplococci or rods. This family houses *Arboricoccus* and *Geminicoccus* (the type genus). *Defluviicoccus* and *Tistrella* are tentatively assigned to this family as well.

#### Emended Description of *Hyphomicrobiaceae* Babudieri 1950 (Approved Lists 1980)

Cells are Gram negative, mostly rod-shaped or ovoid, usually non-motile, predominantly aerobic. The family contains heterotrophic as well as phototrophic genera. The major ubiquinone is Q-10. This family houses *Caenibius, Dichotomicrobium*, *Filomicrobium*, *Hyphomicrobium* (the type genus), *Methyloceanibacter*, *Methyloligella*, *Pedomicrobium*, *Rhodomicrobium*, and *Seliberia*.

#### Emended Description of *Kiloniellaceae* Wiese et al. 2009

The description is as given before (Wiese et al., 2009) with the following modification. Cells are rod-, spiral- or vibrio-shaped. The G+C content of the DNA is 50-61%. This family houses *Kiloniella* (the type genus) and *Aestuariispira*. *Marivibrio* and *Thalassocola* are tentatively assigned to this family as well.

#### Emended Description of *Methylobacteriaceae* Garrity et al. 2006

The description is as before (Garrity et al., 2005c) with the following modification. Colonies are usually pink or cream colored. Cells are Gram-negative, are rod-shaped, non-motile or motile. Aerobic with Chemoorganoheterotrophic or chemolithoheterotrophic metabolism. The major cellular fatty acids are usually C_18:1_ ω7c and C_16:1_ ω7c. The major ubiquinone is Q-10. The G+C content is 60-70 mol%. The family currently comprises the genera *Microvirga*, *Methylobacterium* (the type genus), *Protomonas* and *Psychroglaciecola. Enterovirga* is tentatively assigned to this family.

#### Emended Description of *Methylocystaceae* Bowman 2006

The description is as given before (Bowman, 2005) with the following modification. This family houses *Methylocystis* (the type genus) and *Methylosinus*.

#### Emended Description of *Nitrobacteraceae* Buchanan 1917 (Approved Lists 1980)

The description is as given for *Bradyrhizobiaceae* (Garrity et al., 2005e), which is an illegitimate synonym of the family, with the following additions. This family houses *Afipia*, *Blastobacter*, *Bradyrhizobium* (including *Agromonas*), *Nitrobacter* (the type genus), *Pseudolabrys*, *Pseudorhodoplanes*, *Rhodopseudomonas*, *Tardiphaga* and *Variibacter*. *Rhodoplanes* is tentatively assigned to this family as well.

#### Emended Description of *Notoacmeibacteraceae* Huang et al. 2017

The description is as before (Huang et al., 2017), with the following modification. Gram-stain-negative, oxidase- and catalase-positive, aerobic or facultatively anaerobic heterotrophs. The predominant respiratory quinone is Q-10. The major fatty acids are usually C_18:1_ ω7c and C_18:1_ ω6c. The family contains *Mabikibacter*, *Notoacmeibacter* (the type genus), and *Zhengella*.

#### Emended Description of *Phyllobacteriaceae* Mergaert and Swings 2006

The description is as given for *Phyllobacteriaceae* (Mergaert and Swings, 2005) with the following additions. Cells are Gram-negative, rod, ovoid or coccoid shaped and usually motile by flagella. Predominently aerobic heterotrophs. Generally catalyze and oxidase positive. The predominant respiratory quinone is Q-10. The major polar lipids are phosphatidylcholin, phosphatidylethanolamine and phosphatidylglycerol. The major fatty acids is usually C_18:1_ ω7c. The G+C content of the DNA is 56–64%. This family houses *Aminobacter*, *Aquamicrobium*, *Chelativorans* (including *Thermovum*), *Chelatobacter*, *Corticibacterium*, *Defluvibacter*, *Mesorhizobium*, *Nitratireductor*, *Oricola*, *Phyllobacterium* (the type genus), *Pseudaminobacter*, *Pseudohoeflea*, *Roseitalea*, and *Tianweitania*.

#### Emended Description of *Rhizobiaceae* Conn 1938 (Approved Lists 1980)

The description is as before (Conn, 1938) with the following modification updated in accordance to later findings (Peix et al., 2005; Rahul et al., 2015). Cells are Gram-negative, mostly rod-shaped, usually motile, aerobic and generally heterotrophic with some autotrophic genera. The major respiratory quinone is Q-10. The G+C content is 49–68%. The family contains *Hoeflea*, *Lentilitoribacter*, *Martelella*, *Mycoplana*, and *Neopararhizobium* along with the previously included genera except for *Kaistia*, which has been removed from the family. The type genus is *Rhizobium.*

#### Emended Description of *Rhodobacteraceae* Garrity et al. 2006

The description is as given before (Garrity et al., 2005b) with the following modification. *Rhodobacteraceae* houses multiple and diverse genera but not *Ahrensia*, *Gemmobacter*, *Hyphomonas*, *Maricaulis*, *Methylarcula*, *Pannonibacter*, *Roseibium*, and *Stappia* as stated in the initial description. Additionally the following new genera are included to this family: *Albibacillus*, *Allgaiera*, *Allosediminivita*, *Bieblia*, *Cypionkella*, *Meinhardsimonia*, *Pacificitalea*, *Pseudoprimorskyibacter, Vannielia*, and *Wagnerdoeblera.* The type genus is *Rhodobacter*.

#### Emended Description of *Rhodobiaceae* Garrity et al. 2006

The description is as given before (Garrity et al., 2005f) with the following modification. This family houses *Rhodobium* which is the type and currently the sole genus of the family.

#### Emended Description of *Rhodospirillaceae* Pfennig and Trueper 1971 (Approved Lists 1980)

The description is as given before (Pfennig and Trüper, 1971), with the following modification. This family houses *Caenispirillum*, *Conglomeromonas*, *Haematospirillum*, *Insolitispirillum*, *Magnetospirillum*, *Marispirillum*, *Novispirillum*, *Pararhodospirillum*, *Phaeospirillum*, *Phaeovibrio*, *Rhodospira*, *Rhodospirillum* (the type genus), *Roseospira*, *Roseospirillum*, and *Telmatospirillum.*

#### Emended Description of *Roseiarcaceae* Kulichevskaya et al. 2014

The description is as before (Kulichevskaya et al., 2014) with the following modification after inclusion of *Rhodoblastus.* The major cellular fatty acids are usually C_16:0_, C_16:1_, C_18:1_ ω7c and sometimes C_19:0_ ω8c. The family contains *Roseiarcus* (the type genus) and *Rhodoblastus*.

#### Emended Description of *Sneathiellaceae* Kurahashi et al. 2008

The description is as before (Kurahashi et al., 2008) with the following modification. This family houses *Oceanibacterium* and *Sneathiella* (the type genus). *Ferrovibrio*, *Taonella* and *Marinibaculum* are tentatively assigned to this family.

#### Emended Description of *Sphingomonadaceae* Kosako et al. 2000

The description is as given before (Kosako et al., 2000) with the following modification. This family houses *Blastomonas* (including *Erythromonas*), *Hephaestia*, *Parablastomonas, Parasphingopyxis*, *Rhizorhabdus*, *Rhizorhapis* (including *Rhizomonas*), *Sphingobium*, *Sphingomicrobium*, *Sphingomonas* (the type genus), *Sphingopyxis*, *Sphingorhabdus* and *Stakelama.* Additionally the following new genera are included as well: *Allosphingosinicella* and *Elkelangia.*

#### Emended Description of *Spirochaetaceae* Swellengrebel 1907 (Approved Lists 1980) emend. Abt et al. 2012 emend. Gupta et al. 2013

The description is as given before (Gupta et al., 2013), with the following modification. This family houses *Alkalispirochaeta*, *Marispirochaeta*, *Oceanispirochaeta*, *Salinispira*, *Sediminispirochaeta*, and *Spirochaeta* (the type genus). The genera *Clevelandina*, *Diplocalyx*, *Hollandina* and *Pillotina* which lack published 16S rRNA gene sequences are also tentatively assigned to this family.

#### Emended Description of *Temperatibacteraceae* Teramoto and Nishijima 2014

Cells are Gram-negative, rod-shaped, motile, aerobic heterotrophs. The major ubiquinone is predominantly Q-10. The major polar lipids are phosphatidylethanolamine and phosphatidylglycerol. The major fatty acids are usually iso-C_19:1_ ω9c, iso-C_17:0_, C_18:1_ ω7c, C_17:1_ ω6c, and iso-C_15:0_. The family currently comprises the genera *Eilatimonas, Kordiimonas* and *Temperatibacter* (the type genus). The family *Kordiimonadaceae* was proposed earlier on, too (Xu et al., 2014), but has not been validly published yet. The emended family is based on phylogenetic analyses of genome and 16S rRNA gene sequences.

#### Emended Description of *Treponemataceae* Robinson 1948 (Approved Lists 1980)

The description is as given before (Robinson, 1948) with the following modification. Cells are mostly motile and of typical spirochaete-like, helical shape or rarely (in *Rectinema*) rod-shaped to spherical. The family contains *Rectinema and Treponema* (the type genus).

#### Emended Description of *Xanthobacteraceae* Lee et al. 2005

The description is as given before (Lee et al., 2005) with the following modification. Cells are rod-shaped, coccoid or ellipsoidal, aerobic and generally heterotrophic with some autotrophic genera. This family houses *Ancylobacter* (including *Microcyclus*), *Angulomicrobium*, *Aquabacter*, *Azorhizobium*, *Labrys*, *Methylorhabdus*, *Starkeya* and *Xanthobacter* (the type genus).

### Taxonomic Consequences: Emendations of Genera

#### Emended Description of *Actibacterium* Lucena et al. 2012 emend. Guo et al. 2017

The description is as given before (Lucena et al., 2012; Guo et al., 2017) with additions following the inclusion of *Confluentimicrobium lipolyticum* and *C. naphthalenivorans*. Cells can be motile. Additionally another major polar lipid is frequently sulphoquinovosyldiacylglyceride. The type species is *Actibacterium mucosum.*

#### Emended Description of *Allorhizobium* De Lajudie et al. 1998

The description is as given before (de Lajudie et al., 1998) with additions following the inclusion of *Rhizobium oryziradicis* and *Rhizobium taibaishanense*. The genomic G+C content is 55.1–62.8%. The type species is *Allorhizobium undicola*.

#### Emended Description of *Celeribacter* Ivanova et al. 2010 emend. Lee et al. 2012

The description is as given before (Ivanova et al., 2010; Lee et al., 2012) with additions that reflect developments in the composition of the genus, notably the removal of *Celeribacter manganoxidans* and addition of *Vadicella arenosi*. The genomic G+C content is 56.7–60.9%. The type species is *Celeribacter neptunius*.

#### Emended Description of *Cereibacter* Suresh et al. 2015

The description is as given before (Suresh et al., 2015) with additions following the inclusion of *Rhodobacter azotoformans*, *Rhodobacter johrii*, *Rhodobacter* ovatus and *Rhodobacter sphaeroides*. Consists of motile (via flagella) as well as non-motile species. The genomic G+C content is 66.5–69.1%. The type species is *Cereibacter changlensis*.

#### Emended Description of *Cognatishimia* Wirth and Whitman 2018

After the inclusion of *Thalassobius activus* (Arahal et al., 2019) the original description (Wirth and Whitman, 2018) needs to be modified as follows. Cells are Gram-negative, motile or non-motile, rod- or coccus-shaped, aerobic chemotrophs. Some species require sodium chloride for growth. The predominant ubiquinone is Q-10. The major polar lipids are phosphatidylcholine and phosphatidylglycerol. The major fatty acid is C_18:1_ ω7c and in some species also C_18:1_ ω6c as well as C_10:0_ 3OH. The G+C content is 54.4–56.3%. The type species is *Cognatishimia maritima*.

#### Emended Description of *Magnetospirillum* Schleifer et al. 1991

The description is as given before (Schleifer et al., 1991) with additions following the inclusion of *Phaeospirillum molischianum, Phaeospirillum chandramohanii, Phaeospirillum fulvum, Phaeospirillum oryzae*, and *Phaeospirillum tilakii*. Cells are vibrio-, spiral- or helix-shaped and motile by means of flagella. Includes photoorganoheterotrophic anaerobic and microaerophilic chemoorganotrophic species. Some species are magnetotactic and contain enveloped magnetosomes which are arranged in a chain within the cytoplasm. Mobility and magnetic behavior can be diminished or lost after several subcultivations. The G+C content is 60–71%. The type species is *Magnetospirillum gryphiswaldense*.

#### Emended Description of *Pelagimonas* Hahnke et al. 2013

The description is as given before (Hahnke et al., 2013) with additions following the inclusion of *Tropicibacter phthalicicus*. Oxidase and catalase variable. The G+C content is 55.2–57.9%. The type species is *Pelagimonas varians*.

#### Emended Description of *Pseudorhodobacter* Uchino et al. 2002 emend. Lee et al. 2013 emend. Lee et al. 2016

The description is as given before (Uchino et al., 2002; Lee et al., 2013, 2016) with additions that reflect developments in the composition of the genus, notably the removal of *Pseudorhodobacter aquaticus, P. collinsensis, P. psychrotolerans*, and *P. sinensis*. The G+C content is 58.0–63.1%. The type species is *Pseudorhodobacter ferrugineus*.

#### Emended Description of *Pseudovibrio* Shieh et al. 2004

The description is as given before (Shieh et al., 2004) with additions following the inclusion of *Nesiotobacter exalbescens*. Mesophilic, growing at 20–35°C, some species able to grow at 45°C. Halophilic, no growth in the absence of NaCl. The G+C content is 47.0–55.8%. The type species is *Pseudovibrio denitrificans*.

#### Emended Description of *Salipiger* Martínez-Cánovas et al. 2004 emend. Wirth and Whitman 2018

The description is as given before (Martínez-Cánovas et al., 2004; Wirth and Whitman, 2018) with additions following the inclusion of *Yangia pacifica*. Cells are rod-shaped and of variable sizes with widths up to 1 μm and lengths in the range of 1–2.5 μm. The G+C content is 64.3–67.3%. The type species is *Salipiger mucosus*.

#### Emended Description of *Sphingopyxis* Takeuchi et al. 2001 emend. Baik et al. 2013

The description is as given before (Takeuchi et al., 2001; Baik et al., 2013) with additions that reflect developments in the composition of the genus, notably the removal of *Sphingopyxis baekryungensis*. The G+C content is 63.3–66.4%. The type species is *Sphingopyxis macrogoltabida*.

#### Emended Description of *Tabrizicola* Tarhriz et al. 2013

The description is as given before (Tarhriz et al., 2013) with additions following the inclusion of *Rhodobacter blasticus*. Cells are ovoid to rod-shaped, aerobic or facultatively anaerobic. Includes chemoheterotrophic as well as photoorganotrophic species. The G+C content is 66.4–66.5%. The type species is *Tabrizicola aquatica*.

#### Emended Description of *Thalassobius* Arahal et al. 2005

The description is as given before (Arahal et al., 2005) with additions that reflect developments in the composition of the genus, notably the removal of *Thalassobius activus* and addition of *Litorimicrobium taeanense* as well as *Lutimaribacter litoralis*. Cells are coccoid, rod-shaped or ovoid. The G+C content is 58.5–66.5%. The type species is *Thalassobius mediterraneus*.

#### Emended Description of *Tranquillimonas* Harwati et al. 2008

The description is as given before (Harwati et al., 2008) with additions following the inclusion of *Roseivivax roseus*. Includes motile as well as non-motile species. Oxidase variable. Cells are rod-shaped and of variable sizes, 0.2–1.0 μm width and 1.1–2.8 μm in length. The G+C content is 67.3–67.8%. The type species is *Tranquillimonas alkanivorans*.

### Taxonomic Consequences: Emendations of Species

#### Emended Description of *Acetobacter aceti* (Pasteur 1864) Beijerinck 1898 (Approved Lists 1980)

The description is as before (Beijerinck, 1898) with the following addition. The G+C content of the type-strain genome is 57.1%, its approximate size 3.63 Mbp.

#### Emended Description of *Acetobacter cerevisiae* Cleenwerck et al. 2002

The description is as before (Cleenwerck et al., 2002) with the following restriction. The genomic G+C content of the type strain is 58.0%. Its approximate genome size is 3.09 Mbp.

#### Emended Description of *Acetobacter nitrogenifigens* Dutta and Gachhui 2006

The description is as before (Dutta and Gachhui, 2006) with the following modification. The genomic G+C content of the type strain is 60.5%. Its approximate genome size is 4.27 Mbp.

#### Emended Description of *Acetobacter okinawensis* Iino et al. 2013

The description is as before (Iino et al., 2012) with the following modification. The genomic G+C content of the type strain is 57.6%. Its approximate genome size is 3.17 Mbp.

#### Emended Description of *Acetobacter orleanensis* (Henneberg 1906) Lisdiyanti et al. 2001

The description is as before (Lisdiyanti et al., 2000) with the following addition. The genomic G+C content of the type strain is 56.4%. Its approximate genome size is 3.01 Mbp.

#### Emended Description of *Acetobacter papayae* Iino et al. 2013

The description is as before (Iino et al., 2012) with the following modification. The genomic G+C content of the type strain is 59.3%. Its approximate genome size is 3.04 Mbp.

#### Emended Description of *Acetobacter pasteurianus* (Hansen 1879) Beijerinck and Folpmers 1916 (Approved Lists 1980)

The description is as before (Beijerinck and Folpmers, 1916) with the following addition. The genomic G+C content of the type strain is 53.1%. Its approximate genome size is 2.98 Mbp.

#### Emended Description of *Acetobacter peroxydans* Visser’t Hooft 1925 (Approved Lists 1980)

The description is as before (Visser’t Hooft, 1925) with the following addition. The genomic G+C content of the type strain is 60.5%. Its approximate genome size is 2.71 Mbp.

#### Emended Description of *Acetobacter persici* Iino et al. 2013

The description is as before (Iino et al., 2012) with the following modification. The genomic G+C content of the type strain is 57.2%. Its approximate genome size is 3.70 Mbp.

#### Emended Description of *Acetobacter syzygii* Lisdiyanti et al. 2002

The description is as before (Lisdiyanti et al., 2001) with the following restriction. The G+C content of the type-strain genome is 55.5%, its approximate size 2.67 Mbp.

#### Emended Description of *Acidiphilium angustum* Wichlacz et al. 1986

The description is as before (Wichlacz et al., 1986) with the following modification. The genomic G+C content of the type strain is 63.6%. Its approximate genome size is 4.18 Mbp.

#### Emended Description of *Acidiphilium multivorum* Wakao et al. 1995

The description is as before (Wakao et al., 1994) with the following restriction. The genomic G+C content of the type strain is 67.0%. Its approximate genome size is 4.21 Mbp.

#### Emended Description of *Acidocella facilis* (Wichlacz et al. 1986) Kishimoto et al. 1996

The description is as before (Kishimoto et al., 1995) with the following restriction. The genomic G+C content of the type strain is 64.5%. Its approximate genome size is 3.40 Mbp.

#### Emended Description of *Acidomonas methanolica* (Uhlig et al. 1986) Urakami et al. 1989 emend. Yamashita et al. 2004

The description is as before (Yamashita et al., 2004) with the following modification. The G+C content of the type-strain genome is 64.7%, its approximate size 3.68 Mbp.

#### Emended Description of *Actibacterium ureilyticum* Lin et al. 2016

The description is as before (Lin et al., 2016) with the following modification. The genomic G+C content of the type strain is 64.3%. Its approximate genome size is 4.15 Mbp.

#### Emended Description of *Acuticoccus yangtzensis* Hou et al. 2017

The description is as before (Hou et al., 2015) with the following modification. The genomic G+C content of the type strain is 68.6%. Its approximate genome size is 5.04 Mbp.

#### Emended Description of *Aestuariivita boseongensis* Park et al. 2014

The description is as before (Park et al., 2014f) with the following modification. The G+C content of the type-strain genome is 61.1%, its approximate size 3.94 Mbp.

#### Emended Description of *Afifella marina* (Imhoff 1984) Urdiain et al. 2009

The description is as before (Urdiain et al., 2008) with the following restriction. The genomic G+C content of the type strain is 63.2%. Its approximate genome size is 3.96 Mbp.

#### Emended Description of *Afipia birgiae* La Scola et al. 2002

The description is as before (La Scola et al., 2002) with the following modification. The genomic G+C content of the type strain is 60.8%. Its approximate genome size is 5.30 Mbp.

#### Emended Description of *Afipia clevelandensis* Brenner et al. 1992

The description is as before (Brenner et al., 1991) with the following modification. The genomic G+C content of the type strain is 61.7%. Its approximate genome size is 4.39 Mbp.

#### Emended Description of *Afipia felis* Brenner et al. 1992

The description is as before (Brenner et al., 1991) with the following modification. The genomic G+C content of the type strain is 60.7%. Its approximate genome size is 4.20 Mbp.

#### Emended Description of *Agrobacterium larrymoorei* Bouzar and Jones 2001

The description is as before (Bouzar and Jones, 2001) with the following addition. The G+C content of the type-strain genome is 57.2%, its approximate size 5.16 Mbp.

#### Emended Description of *Agrobacterium nepotum* (Pulawska et al. 2012) Mousavi et al. 2016

The description is as before (Mousavi et al., 2015) with the following modification. The genomic G+C content of the type strain is 59.1%. Its approximate genome size is 5.33 Mbp.

#### Emended Description of *Agrobacterium radiobacter* (Beijerinck and van Delden 1902) Conn 1942 (Approved Lists 1980) emend. Zhang et al. 2014

The description is as before (Zhang et al., 2014a) with the following restriction. The genomic G+C content of the type strain is 59.4%. Its approximate genome size is 5.50 Mbp.

#### Emended Description of *Agrobacterium tumefaciens* (Smith and Townsend 1907) Conn 1942

The description is as before (Conn, 1942) with the following addition. The genomic G+C content of the type strain is 59.3%. Its approximate genome size is 5.66 Mbp.

#### Emended Description of *Albidovulum inexpectatum* Albuquerque et al. 2003

The description is as before (Albuquerque et al., 2002) with the following modification. The genomic G+C content of the type strain is 64.8%. Its approximate genome size is 3.00 Mbp.

#### Emended Description of *Albimonas pacifica* Li et al. 2013

The description is as before (Li G.-W. et al., 2013) with the following modification. The genomic G+C content of the type strain is 72.9%. Its approximate genome size is 6.03 Mbp.

#### Emended Description of *Aliiroseovarius halocynthiae* (Kim et al. 2012) Park et al. 2015

The description is as before (Park et al., 2015c) with the following modification. The G+C content of the type-strain genome is 57.1%, its approximate size 3.40 Mbp.

#### Emended Description of *Aliiroseovarius sediminilitoris* (Park and Yoon 2013) Park et al. 201

The description is as before (Park et al., 2015c) with the following modification. The G+C content of the type-strain genome is 58.7%, its approximate size 3.41 Mbp.

#### Emended Description of *Alkalimicrobium pacificum* Zhang et al. 2015

The description is as before (Zhang G. et al., 2015) with the following modification. The genomic G+C content of the type strain is 65.0%. Its approximate genome size is 5.79 Mbp.

#### Emended Description of *Alkalispirochaeta alkalica* (Zhilina et al. 1996) Sravanthi et al. 2016

The description is as before (Sravanthi et al., 2016) with the following modification. The genomic G+C content of the type strain is 60.5%. Its approximate genome size is 3.34 Mbp.

#### Emended Description of *Alkalispirochaeta americana* (Hoover et al. 2003) Sravanthi et al. 2016

The description is as before (Sravanthi et al., 2016) with the following modification. The genomic G+C content of the type strain is 57.5%. Its approximate genome size is 3.31 Mbp.

#### Emended Description of *Alkalispirochaeta sphaeroplastigenens* (Vishnuvardhan Reddy et al. 2013) Sravanthi et al. 2016

The description is as before (Sravanthi et al., 2016) with the following addition. The G+C content of the type-strain genome is 60.5%, its approximate size 3.35 Mbp.

#### Emended Description of *Allorhizobium oryzae* (Peng et al. 2008) Mousavi et al. 2016

The description is as before (Mousavi et al., 2015) with the following modification. The genomic G+C content of the type strain is 62.8%. Its approximate genome size is 5.39 Mbp.

#### Emended Description of *Allorhizobium vitis* (Ophel and Kerr 1990) Mousavi et al. 2016

The description is as before (Mousavi et al., 2015) with the following modification. The genomic G+C content of the type strain is 57.6%. Its approximate genome size is 5.74 Mbp.

#### Emended Description of *Altererythrobacter atlanticus* Wu et al. 2014

The description is as before (Wu Y.-H. et al., 2014) with the following modification. The genomic G+C content of the type strain is 61.9%. Its approximate genome size is 3.48 Mbp.

#### Emended Description of *Altererythrobacter ishigakiensis* Matsumoto et al. 2011

The description is as before (Matsumoto et al., 2011) with the following modification. The genomic G+C content of the type strain is 56.9%. Its approximate genome size is 2.67 Mbp.

#### Emended Description of *Altererythrobacter marensis* Seo and Lee 2010

The description is as before (Seo and Lee, 2010) with the following modification. The genomic G+C content of the type strain is 64.7%. Its approximate genome size is 2.89 Mbp.

#### Emended Description of *Altererythrobacter namhicola* Park et al. 2011

The description is as before (Park S.C. et al., 2011) with the following modification. The genomic G+C content of the type strain is 65.0%. Its approximate genome size is 2.59 Mbp.

#### Emended Description of *Amylibacter kogurei* Wong et al. 2018

The description is as before (Wong et al., 2018) with the following modification. The G+C content of the type-strain genome is 48.8%, its approximate size 2.98 Mbp.

#### Emended Description of *Ancylobacter aquaticus* (Ørskov 1928) Raj 1983

The description is as before (Raj, 1983) with the following addition. The G+C content of the type-strain genome is 67.0%, its approximate size 4.83 Mbp.

#### Emended Description of *Aquamicrobium aerolatum* Kämpfer et al. 2009

The description is as before (Kämpfer et al., 2009b) with the following addition. The genomic G+C content of the type strain is 60.1%. Its approximate genome size is 3.64 Mbp.

#### Emended Description of *Aquamicrobium defluvii* Bambauer et al. 1998

The description is as before (Bambauer et al., 1998) with the following modification. The G+C content of the type-strain genome is 63.2%, its approximate size 4.52 Mbp.

#### Emended Description of *Arboricoccus pini* Proença et al. 2018

The description is as before (Proença et al., 2018) with the following modification. The G+C content of the type-strain genome is 63.2%.

#### Emended Description of *Asaia prunellae* Suzuki et al. 2012

The description is as before (Suzuki et al., 2010) with the following modification. The genomic G+C content of the type strain is 55.8%. Its approximate genome size is 3.18 Mbp.

#### Emended Description of *Asticcacaulis benevestitus* Vasilyeva et al. 2006

The description is as before (Vasilyeva et al., 2006) with the following modification. The genomic G+C content of the type strain is 58.4%. Its approximate genome size is 4.99 Mbp.

#### Emended Description of *Asticcacaulis excentricus* Poindexter 1964 (Approved Lists 1980)

The description is as before (Poindexter, 1964) with the following addition. The genomic G+C content of the type strain is 59.5%. Its approximate genome size is 4.31 Mbp.

#### Emended Description of *Aurantimonas coralicida* Denner et al. 2003 emend. Rathsack et al. 2011

The description is as before (Rathsack et al., 2011) with the following addition. The genomic G+C content of the type strain is 66.7%. Its approximate genome size is 4.62 Mbp.

#### Emended Description of *Aureimonas altamirensis* (Jurado et al. 2006) Rathsack et al. 2011

The description is as before (Rathsack et al., 2011) with the following modification. The genomic G+C content of the type strain is 64.8%. Its approximate genome size is 4.19 Mbp.

#### Emended Description of *Aureimonas frigidaquae* (Kim et al. 2008) Rathsack et al. 2011

The description is as before (Rathsack et al., 2011) with the following modification. The G+C content of the type-strain genome is 66.1%, its approximate size 4.10 Mbp.

#### Emended Description of *Azorhizobium caulinodans* Dreyfus et al. 1988

The description is as before (Dreyfus et al., 1988) with the following modification. The genomic G+C content of the type strain is 67.3%. Its approximate genome size is 5.37 Mbp.

#### Emended Description of *Azorhizobium doebereinerae* Moreira et al. 2006

The description is as before (Moreira et al., 2006) with the following addition. The genomic G+C content of the type strain is 68.9%. Its approximate genome size is 5.82 Mbp.

#### Emended Description of *Azospirillum brasilense* Tarrand et al. 1979

The description is as before (Tarrand et al., 1978)with the following addition. The genomic G+C content of the type strain is 68.3%. Its approximate genome size is 7.14 Mbp.

#### Emended Description of *Azospirillum thiophilum* Lavrinenko et al. 2010

The description is as before (Lavrinenko et al., 2010) with the following modification. The genomic G+C content of the type strain is 68.2%. Its approximate genome size is 7.61 Mbp.

#### Emended Description of *Bartonella bacilliformis* (Strong et al. 1913) Strong et al. 1915 (Approved Lists 1980)

The description is as before (Strong et al., 1915) with the following addition. The genomic G+C content of the type strain is 38.2%. Its approximate genome size is 1.45 Mbp.

#### Emended Description of *Bartonella clarridgeiae* Lawson and Collins 1996

The description is as before (Lawson and Collins, 1996) with the following addition. The genomic G+C content of the type strain is 35.7%. Its approximate genome size is 1.49 Mbp.

#### Emended Description of *Bartonella doshiae* Birtles et al. 1995

The description is as before (Birtles et al., 1995) with the following modification. The genomic G+C content of the type strain is 37.9%. Its approximate genome size is 1.77 Mbp.

#### Emended Description of *Bartonella elizabethae* (Daly et al. 1993) Brenner et al. 1993

The description is as before (Brenner et al., 1993) with the following modification. The genomic G+C content of the type strain is 38.3%. Its approximate genome size is 1.96 Mbp.

#### Emended Description of *Bartonella grahamii* Birtles et al. 1995

The description is as before (Birtles et al., 1995) with the following modification. The genomic G+C content of the type strain is 38.0%. Its approximate genome size is 2.19 Mbp.

#### Emended Description of *Bartonella henselae* (Regnery et al. 1992) Brenner et al. 1993

The description is as before (Brenner et al., 1993) with the following modification. The genomic G+C content of the type strain is 38.2%. Its approximate genome size is 1.93 Mbp.

#### Emended Description of *Bartonella koehlerae* Droz et al. 2000

The description is as before (Droz et al., 1999) with the following addition. The genomic G+C content of the type strain is 37.6%. Its approximate genome size is 1.74 Mbp.

#### Emended Description of *Bartonella rattaustraliani* Gundi et al. 2009

The description is as before (Gundi et al., 2009) with the following addition. The genomic G+C content of the type strain is 38.8%. Its approximate genome size is 2.16 Mbp.

#### Emended Description of *Bartonella rochalimae* Eremeeva et al. 2012

The description is as before (Eremeeva et al., 2007) with the following addition. The genomic G+C content of the type strain is 35.8%. Its approximate genome size is 1.54 Mbp.

#### Emended Description of *Bartonella schoenbuchensis* Dehio et al. 2001

The description is as before (Dehio et al., 2001) with the following addition. The genomic G+C content of the type strain is 37.6%. Its approximate genome size is 1.74 Mbp.

#### Emended Description of *Bauldia litoralis* (Bauld et al. 1983) Yee et al. 2010

The description is as before (Yee et al., 2010) with the following modification. The genomic G+C content of the type strain is 65.8%. Its approximate genome size is 5.09 Mbp.

#### Emended Description of *Beijerinckia indica* (Starkey and De 1939) Derx 1950 (Approved Lists 1980)

The description is as before (Derx, 1950) with the following addition. The genomic G+C content of the type strain is 57.0%. Its approximate genome size is 4.42 Mbp.

#### Emended Description of *Beijerinckia mobilis* Derx 1950 (Approved Lists 1980)

The description is as before (Derx, 1950) with the following addition. The genomic G+C content of the type strain is 57.2%. Its approximate genome size is 4.32 Mbp.

#### Emended Description of *Belnapia moabensis* Reddy et al. 2006

The description is as before (Reddy et al., 2006) with the following modification. The genomic G+C content of the type strain is 68.9%. Its approximate genome size is 6.72 Mbp.

#### Emended Description of *Blastochloris viridis* (Drews and Giesbrecht 1966) Hiraishi 1997

The description is as before (Hiraishi, 1997) with the following addition. The genomic G+C content of the type strain is 67.9%. Its approximate genome size is 3.72 Mbp.

#### Emended Description of *Blastomonas natatoria* (Sly 1985) Sly and Cahill 1997 emend. Hiraishi et al. 2000

The description is as before (Hiraishi et al., 2000) with the following modification. The G+C content of the type-strain genome is 63.4%, its approximate size 4.05 Mbp.

#### Emended Description of *Borrelia bavariensis* Margos et al. 2013

The description is as before (Margos et al., 2013) with the following addition. The G+C content of the type-strain genome is 28.1%, its approximate size 0.99 Mbp.

#### Emended Description of *Borrelia bissettiae* Margos et al. 2016

The description is as before (Margos et al., 2016) with the following modification. The genomic G+C content of the type strain is 28.3%. Its approximate genome size is 1.40 Mbp.

#### Emended Description of *Borrelia burgdorferi* Johnson et al. 1984 emend. Baranton et al. 1992

The description is as before (Baranton et al., 1992) with the following restriction. The genomic G+C content of the type strain is 28.2%. Its approximate genome size is 1.52 Mbp.

#### Emended Description of *Borrelia coriaceae* Johnson et al. 1987

The description is as before (Johnson et al., 1987) with the following modification. The genomic G+C content of the type strain is 29.3%. Its approximate genome size is 1.57 Mbp.

#### Emended Description of *Borrelia mayonii* Pritt et al. 2016

The description is as before (Pritt et al., 2016) with the following addition. The genomic G+C content of the type strain is 27.9%. Its approximate genome size is 1.31 Mbp.

#### Emended Description of *Borrelia valaisiana* Wang et al. 1997

The description is as before (Wang et al., 1997) with the following addition. The genomic G+C content of the type strain is 27.5%. Its approximate genome size is 1.26 Mbp.

#### Emended Description of *Bosea lathyri* De Meyer and Willems 2012

The description is as before (De Meyer and Willems, 2012) with the following modification. The genomic G+C content of the type strain is 64.8%. Its approximate genome size is 5.92 Mbp.

#### Emended Description of *Bosea robiniae* De Meyer and Willems 2012

The description is as before (De Meyer and Willems, 2012) with the following modification. The genomic G+C content of the type strain is 66.3%. Its approximate genome size is 5.28 Mbp.

#### Emended Description of *Brachyspira alvinipulli* Stanton et al. 1998

The description is as before (Stanton et al., 1998) with the following modification. The genomic G+C content of the type strain is 26.9%. Its approximate genome size is 3.41 Mbp.

#### Emended Description of *Brachyspira hyodysenteriae* (Harris et al. 1972) Ochiai et al. 1998

The description is as before (Ochiai et al., 1997) with the following modification. The genomic G+C content of the type strain is 27.0%. Its approximate genome size is 3.05 Mbp.

#### Emended Description of *Brachyspira innocens* (Kinyon and Harris 1979) Ochiai et al. 1998

The description is as before (Ochiai et al., 1997) with the following modification. The genomic G+C content of the type strain is 27.7%. Its approximate genome size is 3.28 Mbp.

#### Emended Description of *Brachyspira intermedia* (Stanton et al. 1997) Hampson and La 2006

The description is as before (Hampson and La, 2006) with the following modification. The genomic G+C content of the type strain is 27.2%. Its approximate genome size is 3.31 Mbp.

#### Emended Description of *Brachyspira pilosicoli* (Trott et al. 1996) Ochiai et al. 1998

The description is as before (Ochiai et al., 1997) with the following modification. The genomic G+C content of the type strain is 27.9%. Its approximate genome size is 2.56 Mbp.

#### Emended Description of *Bradyrhizobium arachidis* Wang et al. 2015

The description is as before (Wang et al., 2013) with the following modification. The genomic G+C content of the type strain is 63.6%. Its approximate genome size is 9.79 Mbp.

#### Emended Description of *Bradyrhizobium elkanii* Kuykendall et al. 1993

The description is as before (Kuykendall et al., 1992) with the following addition. The genomic G+C content of the type strain is 63.7%. Its approximate genome size is 9.48 Mbp.

#### Emended Description of *Bradyrhizobium embrapense* Delamuta et al. 2015

The description is as before (Delamuta et al., 2015) with the following modification. The genomic G+C content of the type strain is 64.0%. Its approximate genome size is 8.27 Mbp.

#### Emended Description of *Bradyrhizobium japonicum* (Kirchner 1896) Jordan 1982

The description is as before (Jordan, 1982) with the following restriction. The genomic G+C content of the type strain is 63.7%. Its approximate genome size is 9.21 Mbp.

#### Emended Description of *Bradyrhizobium jicamae* Ramírez-Bahena et al. 2009

The description is as before (Ramírez-Bahena et al., 2009) with the following modification. The genomic G+C content of the type strain is 62.4%. Its approximate genome size is 8.71 Mbp.

#### Emended Description of *Bradyrhizobium lablabi* Chang et al. 2011

The description is as before (Chang et al., 2011) with the following modification. The genomic G+C content of the type strain is 62.6%. Its approximate genome size is 8.80 Mbp.

#### Emended Description of *Bradyrhizobium ottawaense* Yu et al. 2014

The description is as before (Yu et al., 2014) with the following modification. The genomic G+C content of the type strain is 63.8%. Its approximate genome size is 8.68 Mbp.

#### Emended Description of *Brevinema andersonii* Defosse et al. 1995

The description is as before (Defosse et al., 1995) with the following modification. The genomic G+C content of the type strain is 35.2%. Its approximate genome size is 1.50 Mbp.

#### Emended Description of *Brevirhabdus pacifica* Wu et al. 2015

The description is as before (Wu et al., 2015) with the following modification. The genomic G+C content of the type strain is 66.2%. Its approximate genome size is 3.30 Mbp.

#### Emended Description of *Brevundimonas aveniformis* Ryu et al. 2007

The description is as before (Ryu et al., 2007) with the following modification. The genomic G+C content of the type strain is 65.1%. Its approximate genome size is 2.58 Mbp.

#### Emended Description of *Brevundimonas bacteroides* (Poindexter 1964) Abraham et al. 1999

The description is as before (Abraham et al., 1999) with the following addition. The genomic G+C content of the type strain is 68.2%. Its approximate genome size is 3.22 Mbp.

#### Emended Description of *Brevundimonas diminuta* (Leifson and Hugh 1954) Segers et al. 1994

The description is as before (Segers et al., 1994) with the following restriction. The genomic G+C content of the type strain is 67.1%. Its approximate genome size is 3.24 Mbp.

#### Emended Description of *Brevundimonas subvibrioides* (Poindexter 1964) Abraham et al. 1999

The description is as before (Abraham et al., 1999) with the following addition. The genomic G+C content of the type strain is 68.4%. Its approximate genome size is 3.45 Mbp.

#### Emended Description of *Brevundimonas vesicularis* (Büsing et al. 1953) Segers et al. 1994

The description is as before (Segers et al., 1994) with the following modification. The genomic G+C content of the type strain is 66.3%. Its approximate genome size is 3.36 Mbp.

#### Emended Description of *Brevundimonas viscosa* Wang et al. 2012

The description is as before (Wang et al., 2012) with the following modification. The genomic G+C content of the type strain is 70.4%. Its approximate genome size is 3.00 Mbp.

#### Emended Description of *Brucella abortus* (Schmidt 1901) Meyer and Shaw 1920 (Approved Lists 1980)

The description is as before (Meyer and Shaw, 1920) with the following addition. The genomic G+C content of the type strain is 57.2%. Its approximate genome size is 3.29 Mbp.

#### Emended Description of *Brucella canis* Carmichael and Bruner 1968 (Approved Lists 1980)

The description is as before (Carmichael and Bruner, 1968) with the following addition. The genomic G+C content of the type strain is 57.2%. Its approximate genome size is 3.31 Mbp.

#### Emended Description of *Brucella ceti* Foster et al. 2007

The description is as before (Foster et al., 2007) with the following addition. The genomic G+C content of the type strain is 57.3%. Its approximate genome size is 3.27 Mbp.

#### Emended Description of *Brucella inopinata* Scholz et al. 2010

The description is as before (Scholz et al., 2010) with the following addition. The genomic G+C content of the type strain is 57.1%. Its approximate genome size is 3.37 Mbp.

#### Emended Description of *Brucella melitensis* (Hughes 1893) Meyer and Shaw 1920 (Approved Lists 1980) emend. Verger et al. 1985

The description is as before (Verger et al., 1985) with the following addition. The genomic G+C content of the type strain is 57.2%. Its approximate genome size is 3.29 Mbp.

#### Emended Description of *Brucella microti* Scholz et al. 2008

The description is as before (Scholz et al., 2008) with the following addition. The genomic G+C content of the type strain is 57.3%. Its approximate genome size is 3.34 Mbp.

#### Emended Description of *Brucella neotomae* Stoenner and Lackman 1957 (Approved Lists 1980)

The description is as before (Stoenner and Lackman, 1957) with the following addition. The genomic G+C content of the type strain is 57.3%. Its approximate genome size is 3.30 Mbp.

#### Emended Description of *Brucella ovis* Buddle 1956 (Approved Lists 1980)

The description is as before (Buddle, 1956) with the following addition. The genomic G+C content of the type strain is 57.2%. Its approximate genome size is 3.28 Mbp.

#### Emended Description of *Brucella suis* Huddleson 1929 (Approved Lists 1980)

The description is as before (Huddleson, 1929) with the following addition. The genomic G+C content of the type strain is 57.3%. Its approximate genome size is 3.32 Mbp.

#### Emended Description of *Caenispirillum salinarum* Ritika et al. 2012

The description is as before (Ritika et al., 2012) with the following modification. The genomic G+C content of the type strain is 68.8%. Its approximate genome size is 4.95 Mbp.

#### Emended Description of *Camelimonas lactis* Kämpfer et al. 2010

The description is as before (Kämpfer et al., 2010b) with the following modification. The G+C content of the type-strain genome is 66.3%, its approximate size 4.17 Mbp.

#### Emended Description of *Caulobacter crescentus* Poindexter 1964 (Approved Lists 1980)

The description is as before (Poindexter, 1964) with the following addition. The G+C content of the type-strain genome is 67.2%, its approximate size 4.12 Mbp.

#### Emended Description of *Caulobacter henricii* Poindexter 1964 (Approved Lists 1980)

The description is as before (Poindexter, 1964) with the following modification. The genomic G+C content of the type strain is 65.8%. Its approximate genome size is 3.96 Mbp.

#### Emended Description of *Caulobacter mirabilis* Abraham et al. 2008

The description is as before (Abraham et al., 2008) with the following modification. The G+C content of the type-strain genome is 69.3%, its approximate size 4.58 Mbp.

#### Emended Description of *Caulobacter vibrioides* Henrici and Johnson 1935 (Approved Lists 1980)

The description is as before (Henrici and Johnson, 1935) with the following addition. The G+C content of the type-strain genome is 67.2%, its approximate size 3.97 Mbp.

#### Emended Description of *Celeribacter baekdonensis* Lee et al. 2012

The description is as before (Lee et al., 2012) with the following modification. The genomic G+C content of the type strain is 58.1%. Its approximate genome size is 4.44 Mbp.

#### Emended Description of *Celeribacter halophilus* (Wang et al. 2012) Lai et al. 2014

The description is as before (Lai et al., 2014) with the following modification. The genomic G+C content of the type strain is 58.1%. Its approximate genome size is 3.87 Mbp.

#### Emended Description of *Celeribacter marinus* Baek et al. 2014

The description is as before (Baek et al., 2014) with the following modification. The genomic G+C content of the type strain is 56.2%. Its approximate genome size is 3.10 Mbp.

#### Emended Description of *Celeribacter neptunius* Ivanova et al. 2010

The description is as before (Ivanova et al., 2010) with the following modification. The genomic G+C content of the type strain is 61.7%. Its approximate genome size is 4.40 Mbp.

#### Emended Description of *Cereibacter changlensis* (Anil Kumar et al. 2007) Suresh et al. 2015

The description is as before (Suresh et al., 2015) with the following modification. The genomic G+C content of the type strain is 68.1%. Its approximate genome size is 4.92 Mbp.

#### Emended Description of *Ciceribacter lividus* Kathiravan et al. 2013

The description is as before (Kathiravan et al., 2013) with the following modification. The G+C content of the type-strain genome is 63.2%, its approximate size 4.52 Mbp.

#### Emended Description of *Citreicella aestuarii* Park et al. 2011

The description is as before (Park M.S. et al., 2011) with the following modification. The genomic G+C content of the type strain is 64.3%. Its approximate genome size is 4.66 Mbp.

#### Emended Description of *Citreicella thiooxidans* Sorokin et al. 2006

The description is as before (Sorokin et al., 2005a) with the following restriction. The genomic G+C content of the type strain is 67.3%. Its approximate genome size is 5.87 Mbp.

#### Emended Description of *Cognatiyoonia koreensis* (Weon et al. 2006) Wirth and Whitman 2018

The description is as before (Wirth and Whitman, 2018) with the following modification. The G+C content of the type-strain genome is 57.2%, its approximate size 3.65 Mbp.

#### Emended Description of *Cognatiyoonia sediminum* (Liang et al. 2015) Wirth and Whitman 2018

The description is as before (Wirth and Whitman, 2018) with the following modification. The G+C content of the type-strain genome is 54.4%, its approximate size 3.26 Mbp.

#### Emended Description of *Cohaesibacter gelatinilyticus* Hwang and Cho 2008 emend. Gallego et al. 2010

The description is as before (Gallego et al., 2010) with the following modification. The genomic G+C content of the type strain is 50.5%. Its approximate genome size is 5.20 Mbp.

#### Emended Description of *Cohaesibacter haloalkalitolerans* Sultanpuram et al. 2013

The description is as before (Sultanpuram et al., 2013) with the following modification. The G+C content of the type-strain genome is 57.1%, its approximate size 5.01 Mbp.

#### Emended Description of *Cohaesibacter marisflavi* Qu et al. 2011

The description is as before (Qu et al., 2011) with the following modification. The genomic G+C content of the type strain is 53.8%. Its approximate genome size is 5.34 Mbp.

#### Emended Description of *Cribrihabitans marinus* Chen et al. 2014

The description is as before (Chen et al., 2014) with the following modification. The genomic G+C content of the type strain is 66.0%. Its approximate genome size is 4.18 Mbp.

#### Emended Description of *Defluviimonas alba* Pan et al. 2015

The description is as before (Pan et al., 2015) with the following restriction. The genomic G+C content of the type strain is 66.5%. Its approximate genome size is 4.99 Mbp.

#### Emended Description of *Defluviimonas aquaemixtae* Jung et al. 2014

The description is as before (Jung et al., 2014b) with the following modification. The G+C content of the type-strain genome is 64.4%, its approximate size 4.24 Mbp.

#### Emended Description of *Devosia chinhatensis* Kumar et al. 2008

The description is as before (Kumar et al., 2008) with the following addition. The genomic G+C content of the type strain is 62.4%. Its approximate genome size is 3.50 Mbp.

#### Emended Description of *Devosia crocina* Verma et al. 2009

The description is as before (Verma et al., 2009) with the following addition. The genomic G+C content of the type strain is 61.3%. Its approximate genome size is 3.72 Mbp.

#### Emended Description of *Devosia elaeis* Mohd Nor et al. 2017

The description is as before (Mohd Nor et al., 2017) with the following modification. The G+C content of the type-strain genome is 64.1%, its approximate size 3.88 Mbp.

#### Emended Description of *Devosia epidermidihirudinis* Galatis et al. 2013

The description is as before (Galatis et al., 2013) with the following addition. The genomic G+C content of the type strain is 61.1%. Its approximate genome size is 3.86 Mbp.

#### Emended Description of *Devosia geojensis* Ryu et al. 2008

The description is as before (Ryu et al., 2008) with the following modification. The genomic G+C content of the type strain is 65.9%. Its approximate genome size is 4.47 Mbp.

#### Emended Description of *Devosia soli* Yoo et al. 2006

The description is as before (Yoo et al., 2006) with the following modification. The genomic G+C content of the type strain is 61.0%. Its approximate genome size is 4.14 Mbp.

#### Emended Description of *Devosia submarina* Romanenko et al. 2013

The description is as before (Romanenko et al., 2013) with the following addition. The G+C content of the type-strain genome is 60.4%, its approximate size 3.98 Mbp.

#### Emended Description of *Dichotomicrobium thermohalophilum* Hirsch and Hoffmann 1989

The description is as before (Hirsch and Hoffmann, 1989) with the following restriction. The genomic G+C content of the type strain is 64.3%. Its approximate genome size is 2.99 Mbp.

#### Emended Description of *Ehrlichia chaffeensis* Anderson et al. 1992 emend. Dumler et al. 2001

The description is as before (Dumler et al., 2001) with the following addition. The genomic G+C content of the type strain is 30.1%.

#### Emended Description of *Ehrlichia muris* Wen et al. 1995 emend. Dumler et al. 2001

The description is as before (Dumler et al., 2001) with the following addition. The genomic G+C content of the type strain is 29.7%. Its approximate genome size is 1.20 Mbp.

#### Emended Description of *Ehrlichia ruminantium* (Cowdry 1925) Dumler et al. 2001

The description is as before (Dumler et al., 2001) with the following addition. The genomic G+C content of the type strain is 27.5%. Its approximate genome size is 1.51 Mbp.

#### Emended Description of *Elioraea tepidiphila* Albuquerque et al. 2008

The description is as before (Albuquerque et al., 2008) with the following restriction. The genomic G+C content of the type strain is 71.3%. Its approximate genome size is 4.30 Mbp.

#### Emended Description of *Ensifer adhaerens* Casida 1982

The description is as before (Casida, 1982) with the following restriction. The genomic G+C content of the type strain is 62.3%. Its approximate genome size is 7.28 Mbp.

#### Emended Description of *Ensifer americanus* (Toledo et al. 2004) Wang et al. 2015

The description is as before (Wang Y.C. et al., 2013) with the following addition. The genomic G+C content of the type strain is 62.3%. Its approximate genome size is 6.65 Mbp.

#### Emended Description of *Ensifer arboris* (Nick et al. 1999) Young 2003

The description is as before (Young, 2003) with the following restriction. The genomic G+C content of the type strain is 62.0%. Its approximate genome size is 6.85 Mbp.

#### Emended Description of *Ensifer fredii* (Scholla and Elkan 1984) Young 2003

The description is as before (Young, 2003) with the following addition. The genomic G+C content of the type strain is 62.3%. Its approximate genome size is 6.58 Mbp.

#### Emended Description of *Ensifer saheli* (de Lajudie et al. 1994) Young 2003

The description is as before (Young, 2003) with the following modification. The genomic G+C content of the type strain is 63.6%. Its approximate genome size is 5.99 Mbp.

#### Emended Description of *Ensifer shofinae* Chen et al. 2017

The description is as before (Chen et al., 2017c) with the following modification. The G+C content of the type-strain genome is 61.6%.

#### Emended Description of *Ensifer sojae* Li et al. 2011

The description is as before (Li et al., 2011) with the following modification. The genomic G+C content of the type strain is 62.0%. Its approximate genome size is 5.96 Mbp.

#### Emended Description of *Epibacterium multivorans* (Lucena et al. 2012) Wirth and Whitman 2018

The description is as before (Wirth and Whitman, 2018) with the following modification. The G+C content of the type-strain genome is 59.7%, its approximate size 4.15 Mbp.

#### Emended Description of *Erythrobacter gangjinensis* Lee et al. 2010

The description is as before (Lee et al., 2010) with the following modification. The genomic G+C content of the type strain is 62.7%. Its approximate genome size is 2.72 Mbp.

#### Emended Description of *Erythrobacter litoralis* Yurkov et al. 1994

The description is as before (Yurkov et al., 1994) with the following modification. The genomic G+C content of the type strain is 65.2%. Its approximate genome size is 3.21 Mbp.

#### Emended Description of *Erythrobacter longus* Shiba and Simidu 1982

The description is as before (Shiba and Smidu, 1982) with the following modification. The genomic G+C content of the type strain is 57.4%. Its approximate genome size is 3.60 Mbp.

#### Emended Description of *Erythrobacter marinus* Jung et al. 2012

The description is as before (Jung et al., 2012b) with the following modification. The genomic G+C content of the type strain is 59.1%. Its approximate genome size is 2.84 Mbp.

#### Emended Description of *Erythrobacter nanhaisediminis* Xu et al. 2010

The description is as before (Xu et al., 2010) with the following modification. The genomic G+C content of the type strain is 62.0%. Its approximate genome size is 2.90 Mbp.

#### Emended Description of *Erythrobacter odishensis* Subhash et al. 2013

The description is as before (Subhash et al., 2013) with the following modification. The G+C content of the type-strain genome is 63.7%, its approximate size 3.19 Mbp.

#### Emended Description of *Falsochrobactrum ovis* Kämpfer et al. 2013

The description is as before (Kämpfer et al., 2013b) with the following modification. The G+C content of the type-strain genome is 50.7%, its approximate size 3.27 Mbp.

#### Emended Description of *Filomicrobium insigne* Wu et al. 2009

The description is as before (Wu et al., 2009) with the following modification. The genomic G+C content of the type strain is 57.4%. Its approximate genome size is 3.85 Mbp.

#### Emended Description of *Fodinicurvata fenggangensis* Wang et al. 2009

The description is as before (Wang Y. et al., 2009) with the following modification. The genomic G+C content of the type strain is 61.0%. Its approximate genome size is 3.77 Mbp.

#### Emended Description of *Fulvimarina manganoxydans* Ren et al. 2014

The description is as before (Ren et al., 2014) with the following modification. The genomic G+C content of the type strain is 62.9%. Its approximate genome size is 4.83 Mbp.

#### Emended Description of *Fulvimarina pelagi* Cho and Giovannoni 2003 emend. Rathsack et al. 2011

The description is as before (Rathsack et al., 2011) with the following modification. The genomic G+C content of the type strain is 61.2%. Its approximate genome size is 3.80 Mbp.

#### Emended Description of *Geminicoccus roseus* Foesel et al. 2008

The description is as before (Foesel et al., 2007) with the following modification. The genomic G+C content of the type strain is 68.5%. Its approximate genome size is 5.70 Mbp.

#### Emended Description of *Gemmobacter aquatilis* Rothe et al. 1988 emend. Chen et al. 2013

The description is as before (Chen W.-M. et al., 2013) with the following modification. The genomic G+C content of the type strain is 65.1%. Its approximate genome size is 3.96 Mbp.

#### Emended Description of *Gemmobacter caeni* (Zheng et al. 2011) Chen et al. 2013

The description is as before (Chen W.-M. et al., 2013) with the following modification. The genomic G+C content of the type strain is 64.7%. Its approximate genome size is 5.13 Mbp.

#### Emended Description of *Gluconacetobacter diazotrophicus* (Gillis et al. 1989) Yamada et al. 1998

The description is as before (Yamada et al., 1997) with the following restriction. The genomic G+C content of the type strain is 66.3%. Its approximate genome size is 3.91 Mbp.

#### Emended Description of *Gluconacetobacter entanii* Schüller et al. 2000

The description is as before (Schüller et al., 2000) with the following modification. The G+C content of the type-strain genome is 62.6%, its approximate size 3.59 Mbp.

#### Emended Description of *Gluconacetobacter liquefaciens* (Asai 1935) Yamada et al. 1998

The description is as before (Yamada et al., 1997) with the following restriction. The G+C content of the type-strain genome is 64.4%, its approximate size 4.18 Mbp.

#### Emended Description of *Gluconobacter cerinus* (ex Asai 1935) Yamada and Akita 1984 emend. Katsura et al. 2002

The description is as before (Katsura et al., 2002) with the following restriction. The G+C content of the type-strain genome is 55.6%, its approximate size 3.59 Mbp.

#### Emended Description of *Gluconobacter frateurii* Mason and Claus 1989

The description is as before (Mason and Claus, 1989) with the following restriction. The G+C content of the type-strain genome is 56.1%, its approximate size 3.31 Mbp.

#### Emended Description of *Gluconobacter kondonii* Malimas et al. 2008

The description is as before (Malimas et al., 2007) with the following modification. The G+C content of the type-strain genome is 58.3%, its approximate size 3.27 Mbp.

#### Emended Description of *Gluconobacter nephelii* Kommanee et al. 2011

The description is as before (Kommanee et al., 2011) with the following modification. The G+C content of the type-strain genome is 55.8%, its approximate size 3.16 Mbp.

#### Emended Description of *Gluconobacter oxydans* (Henneberg 1897) De Ley 1961 (Approved Lists 1980) emend. Mason and Claus 1989

The description is as before (Mason and Claus, 1989) with the following restriction. The G+C content of the type-strain genome is 60.8%, its approximate size 2.91 Mbp.

#### Emended Description of *Haematobacter massiliensis* (Greub and Raoult 2006) Helsel et al. 2007

The description is as before (Helsel et al., 2007) with the following addition. The genomic G+C content of the type strain is 64.6%. Its approximate genome size is 4.13 Mbp.

#### Emended Description of *Haematobacter missouriensis* Helsel et al. 2007

The description is as before (Helsel et al., 2007) with the following modification. The genomic G+C content of the type strain is 64.3%. Its approximate genome size is 3.96 Mbp.

#### Emended Description of *Haematospirillum jordaniae* Humrighouse et al. 2016

The description is as before (Humrighouse et al., 2016) with the following addition. The genomic G+C content of the type strain is 55.4%. Its approximate genome size is 2.47 Mbp.

#### Emended Description of *Hartmannibacter diazotrophicus* Suarez et al. 2014

The description is as before (Suarez et al., 2014) with the following restriction. The G+C content of the type-strain genome is 64.0%, its approximate size 5.45 Mbp.

#### Emended Description of *Hellea balneolensis* Alain et al. 2008

The description is as before (Alain et al., 2008) with the following modification. The genomic G+C content of the type strain is 48.4%. Its approximate genome size is 3.21 Mbp.

#### Emended Description of *Henriciella algicola* Abraham et al. 2017

The description is as before (Abraham et al., 2017) with the following modification. The G+C content of the type-strain genome is 60.4%, its approximate size 3.20 Mbp.

#### Emended Description of *Henriciella aquimarina* Lee et al. 2011

The description is as before (Lee et al., 2011) with the following modification. The genomic G+C content of the type strain is 62.2%. Its approximate genome size is 4.34 Mbp.

#### Emended Description of *Henriciella barbarensis* Abraham et al. 2017

The description is as before (Abraham et al., 2017) with the following modification. The G+C content of the type-strain genome is 59.2%, its approximate size 3.33 Mbp.

#### Emended Description of *Henriciella litoralis* Lee et al. 2011

The description is as before (Lee et al., 2011) with the following modification. The genomic G+C content of the type strain is 58.9%. Its approximate genome size is 3.78 Mbp.

#### Emended Description of *Henriciella marina* Quan et al. 2009

The description is as before (Quan et al., 2009) with the following modification. The genomic G+C content of the type strain is 59.9%. Its approximate genome size is 3.28 Mbp.

#### Emended Description of *Hoeflea halophila* Jung et al. 2013

The description is as before (Jung et al., 2013) with the following modification. The G+C content of the type-strain genome is 61.1%, its approximate size 4.19 Mbp.

#### Emended Description of *Hoeflea marina* Peix et al. 20055

The description is as before (Peix et al., 2005) with the following modification. The G+C content of the type-strain genome is 65.0%, its approximate size 5.26 Mbp.

#### Emended Description of *Hoeflea olei* Rahul et al. 2015

The description is as before (Rahul et al., 2015) with the following modification. The genomic G+C content of the type strain is 65.6%. Its approximate genome size is 4.72 Mbp.

#### Emended Description of *Humitalea rosea* Margesin and Zhang 2013

The description is as before (Margesin and Zhang, 2013) with the following modification. The genomic G+C content of the type strain is 69.6%. Its approximate genome size is 4.97 Mbp.

#### Emended Description of *Hwanghaeicola aestuarii* Kim et al. 2010

The description is as before (Kim J.M. et al., 2010) with the following modification. The genomic G+C content of the type strain is 66.0%. Its approximate genome size is 4.54 Mbp.

#### Emended Description of *Hyphomicrobium zavarzinii* Hirsch 1989

The description is as before (Hirsch, 1989) with the following modification. The genomic G+C content of the type strain is 63.7%. Its approximate genome size is 4.65 Mbp.

#### Emended Description of *Hyphomonas adhaerens* Weiner et al. 2000

The description is as before (Weiner et al., 2000) with the following modification. The genomic G+C content of the type strain is 61.3%. Its approximate genome size is 3.67 Mbp.

#### Emended Description of *Hyphomonas hirschiana* Weiner et al. 1985

The description is as before (Weiner et al., 1985) with the following modification. The genomic G+C content of the type strain is 61.9%. Its approximate genome size is 3.69 Mbp.

#### Emended Description of *Hyphomonas jannaschiana* Weiner et al. 1985

The description is as before (Weiner et al., 1985) with the following modification. The genomic G+C content of the type strain is 61.4%. Its approximate genome size is 3.64 Mbp.

#### Emended Description of *Hyphomonas johnsonii* Weiner et al. 2000

The description is as before (Weiner et al., 2000) with the following modification. The genomic G+C content of the type strain is 62.5%. Its approximate genome size is 3.62 Mbp.

#### Emended Description of *Hyphomonas oceanitis* Weiner et al. 1985

The description is as before (Weiner et al., 1985) with the following modification. The genomic G+C content of the type strain is 60.2%. Its approximate genome size is 4.28 Mbp.

#### Emended Description of *Hyphomonas polymorpha* (ex Pongratz 1957) Moore et al. 1984

The description is as before (Moore et al., 1984) with the following modification. The genomic G+C content of the type strain is 62.3%. Its approximate genome size is 4.05 Mbp.

#### Emended Description of *Jannaschia faecimaris* Jung and Yoon 2014

The description is as before (Jung and Yoon, 2014) with the following modification. The genomic G+C content of the type strain is 62.0%. Its approximate genome size is 3.81 Mbp.

#### Emended Description of *Jannaschia pohangensis* Kim et al. 2008

The description is as before (Kim B.Y. et al., 2008) with the following modification. The genomic G+C content of the type strain is 65.5%. Its approximate genome size is 3.73 Mbp.

#### Emended Description of *Jannaschia rubra* Macián et al. 2005

The description is as before (Macián et al., 2005) with the following modification. The genomic G+C content of the type strain is 67.9%. Its approximate genome size is 3.55 Mbp.

#### Emended Description of *Jannaschia seosinensis* Choi et al. 2006

The description is as before (Choi et al., 2006) with the following modification. The genomic G+C content of the type strain is 65.3%. Its approximate genome size is 3.83 Mbp.

#### Emended Description of *Jhaorihella thermophila* Rekha et al. 2011

The description is as before (Rekha et al., 2011) with the following modification. The genomic G+C content of the type strain is 66.0%. Its approximate genome size is 3.77 Mbp.

#### Emended Description of *Kaistia granuli* Lee et al. 2007

The description is as before (Lee et al., 2007a) with the following modification. The genomic G+C content of the type strain is 65.9%. Its approximate genome size is 4.77 Mbp.

#### Emended Description of *Kaistia soli* Weon et al. 2008

The description is as before (Weon et al., 2008b) with the following modification. The genomic G+C content of the type strain is 64.5%. Its approximate genome size is 5.24 Mbp.

#### Emended Description of *Kiloniella majae* Gerpe et al. 2017

The description is as before (Gerpe et al., 2017) with the following addition. The G+C content of the type-strain genome is 45.5%, its approximate size 4.39 Mbp.

#### Emended Description of *Komagataeibacter europaeus* (Sievers et al. 1992) Yamada et al. 2013

The description is as before (Yamada et al., 2012) with the following restriction. The genomic G+C content of the type strain is 61.3%. Its approximate genome size is 4.23 Mbp.

#### Emended Description of *Komagataeibacter kombuchae* (Dutta and Gachhui 2007) Yamada et al. 2013

The description is as before (Yamada et al., 2012) with the following modification. The genomic G+C content of the type strain is 59.4%. Its approximate genome size is 3.59 Mbp.

#### Emended Description of *Komagataeibacter oboediens* (Sokollek et al. 1998) Yamada et al. 2013

The description is as before (Yamada et al., 2012) with the following modification. The G+C content of the type-strain genome is 61.4%, its approximate size 3.75 Mbp.

#### Emended Description of *Komagataeibacter xylinus* (Brown 1886) Yamada et al. 2013

The description is as before (Yamada et al., 2012) with the following restriction. The genomic G+C content of the type strain is 62.3%. Its approximate genome size is 3.63 Mbp.

#### Emended Description of *Kordiimonas gwangyangensis* Kwon et al. 2005 emend. Yang et al. 2013

The description is as before (Yang et al., 2013) with the following restriction. The genomic G+C content of the type strain is 57.5%. Its approximate genome size is 4.08 Mbp.

#### Emended Description of *Kordiimonas lacus* Xu et al. 2011 emend. Wu et al. 2016

The description is as before (Wu et al., 2016) with the following modification. The genomic G+C content of the type strain is 57.2%. Its approximate genome size is 4.02 Mbp.

#### Emended Description of *Leisingera methylohalidivorans* Schaefer et al. 2002 emend. Vandecandelaere et al. 2008

The description is as before (Vandecandelaere et al., 2008b) with the following modification. The genomic G+C content of the type strain is 62.3%. Its approximate genome size is 4.65 Mbp.

#### Emended Description of *Lentibacter algarum* Li et al. 2012

The description is as before (Li et al., 2012) with the following modification. The genomic G+C content of the type strain is 55.7%. Its approximate genome size is 3.29 Mbp.

#### Emended Description of *Leptonema illini* Hovind-Hougen 1983

The description is as before (Hovind-Hougen, 1979) with the following restriction. The genomic G+C content of the type strain is 54.3%. Its approximate genome size is 4.52 Mbp.

#### Emended Description of *Leptospira alexanderi* Brenner et al. 1999

The description is as before (Brenner et al., 1999) with the following modification. The genomic G+C content of the type strain is 40.2%. Its approximate genome size is 4.22 Mbp.

#### Emended Description of *Leptospira alstonii* Smythe et al. 2013

The description is as before (Smythe et al., 2013) with the following restriction. The genomic G+C content of the type strain is 42.5%. Its approximate genome size is 4.44 Mbp.

#### Emended Description of *Leptospira biflexa* (Wolbach and Binger 1914) Noguchi 1918 (Approved Lists 1980) emend. Faine and Stallman 1982

The description is as before (Faine and Stallman, 1982) with the following addition. The genomic G+C content of the type strain is 38.9%. Its approximate genome size is 3.95 Mbp.

#### Emended Description of *Leptospira fainei* Perolat et al. 1998

The description is as before (Perolat et al., 1998) with the following addition. The genomic G+C content of the type strain is 43.5%. Its approximate genome size is 4.29 Mbp.

#### Emended Description of *Leptospira inadai* Yasuda et al. 1987

The description is as before (Yasuda et al., 1987) with the following restriction. The genomic G+C content of the type strain is 44.6%. Its approximate genome size is 4.46 Mbp.

#### Emended Description of *Leptospira interrogans* (Stimson 1907) Wenyon 1926 (Approved Lists 1980) emend. Faine and Stallman 1982

The description is as before (Faine and Stallman, 1982) with the following addition. The genomic G+C content of the type strain is 35.0%. Its approximate genome size is 4.60 Mbp.

#### Emended Description of *Leptospira kirschneri* Ramadass et al. 1992

The description is as before (Ramadass et al., 1992) with the following addition. The genomic G+C content of the type strain is 35.9%. Its approximate genome size is 4.41 Mbp.

#### Emended Description of *Leptospira kmetyi* Slack et al. 2009

The description is as before (Slack et al., 2009) with the following modification. The genomic G+C content of the type strain is 44.8%. Its approximate genome size is 4.42 Mbp.

#### Emended Description of *Leptospira licerasiae* Matthias et al. 2009

The description is as before (Matthias et al., 2008) with the following modification. The genomic G+C content of the type strain is 41.1%. Its approximate genome size is 4.21 Mbp.

#### Emended Description of *Leptospira meyeri* Yasuda et al. 1987

The description is as before (Yasuda et al., 1987) with the following modification. The genomic G+C content of the type strain is 38.0%. Its approximate genome size is 4.24 Mbp.

#### Emended Description of *Leptospira noguchii* Yasuda et al. 1987

The description is as before (Yasuda et al., 1987) with the following restriction. The genomic G+C content of the type strain is 35.5%. Its approximate genome size is 4.71 Mbp.

#### Emended Description of *Leptospira santarosai* Yasuda et al. 1987

The description is as before (Yasuda et al., 1987) with the following restriction. The genomic G+C content of the type strain is 41.8%. Its approximate genome size is 3.98 Mbp.

#### Emended Description of *Leptospira terpstrae* Smythe et al. 2013

The description is as before (Smythe et al., 2013) with the following restriction. The genomic G+C content of the type strain is 38.2%. Its approximate genome size is 4.09 Mbp.

#### Emended Description of *Leptospira vanthielii* Smythe et al. 2013

The description is as before (Smythe et al., 2013) with the following restriction. The genomic G+C content of the type strain is 38.9%. Its approximate genome size is 4.23 Mbp.

#### Emended Description of *Leptospira wolbachii* Yasuda et al. 1987

The description is as before (Yasuda et al., 1987) with the following modification. The genomic G+C content of the type strain is 39.2%. Its approximate genome size is 4.08 Mbp.

#### Emended Description of *Leptospira wolffii* Slack et al. 2008

The description is as before (Slack et al., 2008) with the following modification. The genomic G+C content of the type strain is 45.6%. Its approximate genome size is 4.40 Mbp.

#### Emended Description of *Leptospira yanagawae* Smythe et al. 2013

The description is as before (Smythe et al., 2013) with the following restriction. The genomic G+C content of the type strain is 38.2%. Its approximate genome size is 4.06 Mbp.

#### Emended Description of *Limimaricola cinnabarinus* (Tsubuchi et al. 2013) Wirth and Whitman 2018

The description is as before (Wirth and Whitman, 2018) with the following modification. The G+C content of the type-strain genome is 66.7%, its approximate size 3.90 Mbp.

#### Emended Description of *Limimaricola hongkongensis* (Lau et al. 2004) Wirth and Whitman 2018

The description is as before (Wirth and Whitman, 2018) with the following modification. The G+C content of the type-strain genome is 68.3%, its approximate size 3.19 Mbp.

#### Emended Description of *Limimaricola pyoseonensis* (Moon et al. 2010) Wirth and Whitman 2018

The description is as before (Wirth and Whitman, 2018) with the following modification. The G+C content of the type-strain genome is 70.3%, its approximate size 3.91 Mbp.

#### Emended Description of *Limimonas halophila* Amoozegar et al. 2013

The description is as before (Amoozegar et al., 2013) with the following modification. The genomic G+C content of the type strain is 69.5%. Its approximate genome size is 3.04 Mbp.

#### Emended Description of *Litoreibacter albidus* Romanenko et al. 2011

The description is as before (Romanenko et al., 2011a) with the following modification. The genomic G+C content of the type strain is 59.2%. Its approximate genome size is 3.58 Mbp.

#### Emended Description of *Litoreibacter arenae* (Kim et al. 2009) Kim et al. 2012)

The description is as before (Kim et al., 2012a) with the following modification. The genomic G+C content of the type strain is 60.2%. Its approximate genome size is 3.69 Mbp.

#### Emended Description of *Litoreibacter janthinus* Romanenko et al. 2011

The description is as before (Romanenko et al., 2011a) with the following modification. The genomic G+C content of the type strain is 57.5%. Its approximate genome size is 3.75 Mbp.

#### Emended Description of *Litorimonas taeanensis* Jung et al. 2011 emend. Nedashkovskaya et al. 2013

The description is as before (Nedashkovskaya et al., 2013) with the following modification. The G+C content of the type-strain genome is 46.9%, its approximate size 2.78 Mbp.

#### Emended Description of *Loktanella atrilutea* Hosoya and Yokota 2007

The description is as before (Hosoya and Yokota, 2007) with the following modification. The genomic G+C content of the type strain is 64.9%. Its approximate genome size is 4.21 Mbp.

#### Emended Description of *Loktanella cinnabarina* Tsubouchi et al. 2013

The description is as before (Tsubouchi et al., 2013) with the following modification. The genomic G+C content of the type strain is 66.7%. Its approximate genome size is 3.90 Mbp.

#### Emended Description of *Loktanella hongkongensis* Lau et al. 2004

The description is as before (Lau et al., 2004) with the following modification. The genomic G+C content of the type strain is 68.3%. Its approximate genome size is 3.19 Mbp.

#### Emended Description of *Loktanella koreensis* Weon et al. 2006

The description is as before (Weon et al., 2006) with the following modification. The genomic G+C content of the type strain is 57.2%. Its approximate genome size is 3.65 Mbp.

#### Emended Description of *Loktanella maritima* Tanaka et al. 2014

The description is as before (Tanaka et al., 2014) with the following addition. The genomic G+C content of the type strain is 53.4%. Its approximate genome size is 3.68 Mbp.

#### Emended Description of *Loktanella pyoseonensis* Moon et al. 2010

The description is as before (Moon et al., 2010) with the following modification. The genomic G+C content of the type strain is 70.3%. Its approximate genome size is 3.91 Mbp.

#### Emended Description of *Loktanella rosea* Ivanova et al. 2005

The description is as before (Ivanova et al., 2005) with the following restriction. The genomic G+C content of the type strain is 57.7%. Its approximate genome size is 3.51 Mbp.

#### Emended Description of *Loktanella sediminum* Liang et al. 2015

The description is as before (Liang et al., 2015) with the following modification. The genomic G+C content of the type strain is 54.4%. Its approximate genome size is 3.26 Mbp.

#### Emended Description of *Loktanella tamlensis* Lee 2012

The description is as before (Lee, 2012) with the following modification. The genomic G+C content of the type strain is 56.9%. Its approximate genome size is 3.19 Mbp.

#### Emended Description of *Loktanella vestfoldensis* Van Trappen et al. 2004

The description is as before (Van Trappen et al., 2004) with the following modification. The genomic G+C content of the type strain is 61.8%. Its approximate genome size is 3.72 Mbp.

#### Emended Description of *Lutibaculum baratangense* Anil Kumar et al. 2012

The description is as before (Anil Kumar et al., 2012) with the following modification. The genomic G+C content of the type strain is 68.6%. Its approximate genome size is 4.30 Mbp.

#### Emended Description of *Magnetospirillum gryphiswaldense* Schleifer et al. 1992

The description is as before (Schleifer et al., 1991) with the following modification. The genomic G+C content of the type strain is 63.3%. Its approximate genome size is 4.37 Mbp.

#### Emended Description of *Magnetospirillum marisnigri* Dziuba et al. 2016

The description is as before (Dziuba et al., 2016) with the following modification. The genomic G+C content of the type strain is 64.7%. Its approximate genome size is 4.62 Mbp.

#### Emended Description of *Magnetovibrio blakemorei* Bazylinski et al. 2013

The description is as before (Bazylinski et al., 2013b) with the following modification. The genomic G+C content of the type strain is 54.3%.

#### Emended Description of *Maliponia aquimaris* Jung et al. 2016

The description is as before (Jung et al., 2016) with the following modification. The G+C content of the type-strain genome is 67.2%, its approximate size 5.31 Mbp.

#### Emended Description of *Mameliella alba* Zheng et al. 2010

The description is as before (Zheng et al., 2010) with the following modification. The genomic G+C content of the type strain is 65.2%. Its approximate genome size is 5.26 Mbp.

#### Emended Description of *Mameliella atlantica* Xu et al. 2015

The description is as before (Xu et al., 2015) with the following modification. The genomic G+C content of the type strain is 65.0%. Its approximate genome size is 5.90 Mbp.

#### Emended Description of *Maribius salinus* Choi et al. 2007

The description is as before (Choi et al., 2007) with the following modification. The genomic G+C content of the type strain is 67.7%. Its approximate genome size is 3.57 Mbp.

#### Emended Description of *Marinovum algicola* (Lafay et al. 1995) Martens et al. 2006

The description is as before (Martens et al., 2006) with the following addition. The genomic G+C content of the type strain is 65.0%. Its approximate genome size is 5.39 Mbp.

#### Emended Description of *Maritimibacter alkaliphilus* Lee et al. 2007

The description is as before (Lee et al., 2007b) with the following modification. The genomic G+C content of the type strain is 64.1%. Its approximate genome size is 4.53 Mbp.

#### Emended Description of *Marivita hallyeonensis* Yoon et al. 2012

The description is as before (Yoon et al., 2012) with the following modification. The genomic G+C content of the type strain is 60.4%. Its approximate genome size is 4.19 Mbp.

#### Emended Description of *Martelella mediterranea* Rivas et al. 2005

The description is as before (Rivas et al., 2005) with the following modification. The genomic G+C content of the type strain is 62.4%. Its approximate genome size is 5.64 Mbp.

#### Emended Description of *Mesorhizobium albiziae* Wang et al. 2007

The description is as before (Wang et al., 2007) with the following modification. The genomic G+C content of the type strain is 62.1%. Its approximate genome size is 6.27 Mbp.

#### Emended Description of *Mesorhizobium alhagi* Chen et al. 2010

The description is as before (Chen W.-M. et al., 2010) with the following restriction. The genomic G+C content of the type strain is 62.7%. Its approximate genome size is 6.97 Mbp.

#### Emended Description of *Mesorhizobium erdmanii* Martínez-Hidalgo et al. 2015

The description is as before (Martínez-Hidalgo et al., 2015) with the following modification. The genomic G+C content of the type strain is 62.7%. Its approximate genome size is 7.02 Mbp.

#### Emended Description of *Mesorhizobium loti* (Jarvis et al. 1982) Jarvis et al. 1997 emend. Hameed et al. 2015

The description is as before (Hameed et al., 2015) with the following restriction. The G+C content of the type-strain genome is 62.4%, its approximate size 7.45 Mbp.

#### Emended Description of *Mesorhizobium mediterraneum* (Nour et al. 1995) Jarvis et al. 1997

The description is as before (Jarvis et al., 1997) with the following addition. The genomic G+C content of the type strain is 62.0%. Its approximate genome size is 7.20 Mbp.

#### Emended Description of *Mesorhizobium metallidurans* Vidal et al. 2009

The description is as before (Vidal et al., 2009) with the following addition. The genomic G+C content of the type strain is 62.5%. Its approximate genome size is 6.23 Mbp.

#### Emended Description of *Mesorhizobium muleiense* Zhang et al. 2012

The description is as before (Zhang J.J. et al., 2012) with the following modification. The genomic G+C content of the type strain is 62.3%. Its approximate genome size is 6.81 Mbp.

#### Emended Description of *Mesorhizobium qingshengii* Zheng et al. 2013

The description is as before (Zheng et al., 2013) with the following modification. The genomic G+C content of the type strain is 62.7%. Its approximate genome size is 7.06 Mbp.

#### Emended Description of *Mesorhizobium soli* Nguyen et al. 2015

The description is as before (Nguyen et al., 2015) with the following modification. The G+C content of the type-strain genome is 62.6%, its approximate size 6.27 Mbp.

#### Emended Description of *Mesorhizobium temperatum* Gao et al. 2004

The description is as before (Gao et al., 2004) with the following modification. The genomic G+C content of the type strain is 61.9%. Its approximate genome size is 7.17 Mbp.

#### Emended Description of *Methylarcula marina* Doronina et al. 2000

The description is as before (Doronina et al., 2000) with the following modification. The G+C content of the type-strain genome is 63.0%, its approximate size 4.23 Mbp.

#### Emended Description of *Methylobacterium chloromethanicum* McDonald et al. 2001

The description is as before (McDonald et al., 2001) with the following modification. The G+C content of the type-strain genome is 68.1%, its approximate size 6.18 Mbp.

#### Emended Description of *Methylobacterium gossipiicola* Madhaiyan et al. 2012

The description is as before (Madhaiyan et al., 2012) with the following modification. The genomic G+C content of the type strain is 68.7%. Its approximate genome size is 4.52 Mbp.

#### Emended Description of *Methylobacterium komagatae* Kato et al. 2008

The description is as before (Kato et al., 2008) with the following modification. The genomic G+C content of the type strain is 67.5%. Its approximate genome size is 5.50 Mbp.

#### Emended Description of *Methylobacterium nodulans* Jourand et al. 2004

The description is as before (Jourand et al., 2004) with the following addition. The genomic G+C content of the type strain is 68.4%. Its approximate genome size is 8.84 Mbp.

#### Emended Description of *Methylobacterium organophilum* Patt et al. 1976 (Approved Lists 1980)

The description is as before (Patt et al., 1976) with the following modification. The G+C content of the type-strain genome is 71.4%, its approximate size 6.75 Mbp.

#### Emended Description of *Methylobacterium oryzae* Madhaiyan et al. 2007

The description is as before (Madhaiyan et al., 2007) with the following modification. The genomic G+C content of the type strain is 69.5%. Its approximate genome size is 6.52 Mbp.

#### Emended Description of *Methylobacterium phyllosphaerae* Madhaiyan et al. 2009

The description is as before (Madhaiyan et al., 2009) with the following modification. The genomic G+C content of the type strain is 69.6%. Its approximate genome size is 6.20 Mbp.

#### Emended Description of *Methylobacterium phyllostachyos* Madhaiyan and Poonguzhali 2014

The description is as before (Madhaiyan and Poonguzhali, 2014) with the following modification. The genomic G+C content of the type strain is 68.7%. Its approximate genome size is 6.01 Mbp.

#### Emended Description of *Methylobacterium platani* Kang et al. 2007

The description is as before (Kang et al., 2007) with the following modification. The genomic G+C content of the type strain is 71.2%. Its approximate genome size is 7.01 Mbp.

#### Emended Description of *Methylobacterium pseudosasicola* Madhaiyan and Poonguzhali 2014

The description is as before (Madhaiyan and Poonguzhali, 2014) with the following modification. The genomic G+C content of the type strain is 68.4%. Its approximate genome size is 6.85 Mbp.

#### Emended Description of *Methylobacterium radiotolerans* (Ito and Iizuka 1971) Green and Bousfield 1983

The description is as before (Green and Bousfield, 1983) with the following addition. The genomic G+C content of the type strain is 71.0%. Its approximate genome size is 6.90 Mbp.

#### Emended Description of *Methylobacterium tarhaniae* Veyisoglu et al. 2013

The description is as before (Veyisoglu et al., 2013) with the following modification. The genomic G+C content of the type strain is 70.4%. Its approximate genome size is 6.74 Mbp.

#### Emended Description of *Methylobrevis pamukkalensis* Poroshina et al. 2015

The description is as before (Poroshina et al., 2015) with the following modification. The genomic G+C content of the type strain is 68.9%. Its approximate genome size is 4.38 Mbp.

#### Emended Description of *Methylocapsa acidiphila* Dedysh et al. 2002

The description is as before (Dedysh et al., 2002) with the following modification. The genomic G+C content of the type strain is 61.8%. Its approximate genome size is 4.10 Mbp.

#### Emended Description of *Methyloceanibacter caenitepidi* Takeuchi et al. 2014

The description is as before (Takeuchi et al., 2014) with the following modification. The genomic G+C content of the type strain is 62.8%. Its approximate genome size is 3.42 Mbp.

#### Emended Description of *Methylocella silvestris* Dunfield et al. 2003

The description is as before (Dunfield et al., 2003) with the following modification. The genomic G+C content of the type strain is 63.1%. Its approximate genome size is 4.31 Mbp.

#### Emended Description of *Methylocystis parvus* (ex Whittenbury et al. 1970) Bowman et al. 1993

The description is as before (Bowman et al., 1993) with the following restriction. The genomic G+C content of the type strain is 63.4%. Its approximate genome size is 4.48 Mbp.

#### Emended Description of *Methyloferula stellata* Vorobev et al. 2011

The description is as before (Vorobev et al., 2011) with the following addition. The genomic G+C content of the type strain is 59.5%. Its approximate genome size is 4.24 Mbp.

#### Emended Description of *Methyloligella halotolerans* Doronina et al. 2014

The description is as before (Doronina et al., 2013) with the following modification. The genomic G+C content of the type strain is 63.6%. Its approximate genome size is 3.19 Mbp.

#### Emended Description of *Methylorubrum extorquens* (Urakami and Komagata 1984) Green and Ardley 2018

The description is as before (Green and Ardley, 2018) with the following restriction. The G+C content of the type-strain genome is 68.3%, its approximate size 5.72 Mbp.

#### Emended Description of *Methylorubrum populi* (Van Aken et al. 2004) Green and Ardley 2018

The description is as before (Green and Ardley, 2018) with the following modification. The genomic G+C content of the type strain is 69.4%. Its approximate genome size is 5.85 Mbp.

#### Emended Description of *Methylorubrum salsuginis* (Wang et al. 2007) Green and Ardley 2018

The description is as before (Green and Ardley, 2018) with the following addition. The genomic G+C content of the type strain is 69.6%. Its approximate genome size is 5.32 Mbp.

#### Emended Description of *Methylosinus sporium* (ex Whittenbury et al. 1970) Bowman et al. 1993

The description is as before (Bowman et al., 1993) with the following restriction. The G+C content of the type-strain genome is 65.2%, its approximate size 3.79 Mbp.

#### Emended Description of *Methylosinus trichosporium* (ex Whittenbury et al. 1970) Bowman et al. 1993

The description is as before (Bowman et al., 1993) with the following modification. The genomic G+C content of the type strain is 65.9%. Its approximate genome size is 4.96 Mbp.

#### Emended Description of *Microvirga guangxiensis* Zhang et al. 2009

The description is as before (Zhang et al., 2009) with the following modification. The genomic G+C content of the type strain is 61.4%. Its approximate genome size is 4.72 Mbp.

#### Emended Description of *Microvirga subterranea* Kanso and Patel 2003

The description is as before (Kanso and Patel, 2003) with the following modification. The G+C content of the type-strain genome is 65.1%, its approximate size 5.15 Mbp.

#### Emended Description of *Microvirga vignae* Radl et al. 2014

The description is as before (Radl et al., 2014) with the following modification. The genomic G+C content of the type strain is 61.1%. Its approximate genome size is 6.47 Mbp.

#### Emended Description of *Monaibacterium marinum* Chernikova et al. 2017

The description is as before (Chernikova et al., 2017) with the following modification. The G+C content of the type-strain genome is 58.9%, its approximate size 3.73 Mbp.

#### Emended Description of *Nautella italica* Vandecandelaere et al. 2009

The description is as before (Vandecandelaere et al., 2009) with the following restriction. The genomic G+C content of the type strain is 60.0%. Its approximate genome size is 4.06 Mbp.

#### Emended Description of *Neoasaia chiangmaiensis* Yukphan et al. 2006

The description is as before (Yukphan et al., 2005) with the following addition. The genomic G+C content of the type strain is 61.5%. Its approximate genome size is 3.41 Mbp.

#### Emended Description of *Neokomagataea thailandica* Yukphan et al. 2011

The description is as before (Yukphan et al., 2011) with the following modification. The genomic G+C content of the type strain is 52.4%. Its approximate genome size is 2.49 Mbp.

#### Emended Description of *Neomegalonema perideroedes* (Thomsen et al. 2006) Oren 2017

The description is as before (Oren, 2017b) with the following modification. The G+C content of the type-strain genome is 67.2%, its approximate size 3.41 Mbp.

#### Emended Description of *Neorhizobium alkalisoli* (Lu et al. 2009) Mousavi et al. 2015

The description is as before (Mousavi et al., 2014) with the following modification. The G+C content of the type-strain genome is 60.3%, its approximate size 5.87 Mbp.

#### Emended Description of *Neorhizobium galegae* (Lindström 1989) Mousavi et al. 2015

The description is as before (Mousavi et al., 2014) with the following restriction. The genomic G+C content of the type strain is 61.2%. Its approximate genome size is 6.46 Mbp.

#### Emended Description of *Neorhizobium huautlense* (Wang et al. 1998) Mousavi et al. 2015

The description is as before (Mousavi et al., 2014) with the following restriction. The G+C content of the type-strain genome is 60.0%, its approximate size 5.73 Mbp.

#### Emended Description of *Neorickettsia risticii* (Holland et al. 1985) Dumler et al. 2001

The description is as before (Dumler et al., 2001) with the following addition. The genomic G+C content of the type strain is 41.3%.

#### Emended Description of *Neorickettsia sennetsu* (Misao and Kobayashi 1956) Dumler et al. 2001

The description is as before (Dumler et al., 2001) with the following addition. The genomic G+C content of the type strain is 41.1%.

#### Emended Description of *Neptunicoccus sediminis* Zhang et al. 2018

The description is as before (Zhang Y.-J. et al., 2018) with the following addition. The G+C content of the type-strain genome is 57.5%, its approximate size 3.86 Mbp.

#### Emended Description of *Nereida ignava* Pujalte et al. 2005

The description is as before (Pujalte et al., 2005) with the following modification. The genomic G+C content of the type strain is 54.1%. Its approximate genome size is 2.84 Mbp.

#### Emended Description of *Nitratireductor aquibiodomus* Labbé et al. 2004

The description is as before (Labbé et al., 2004) with the following modification. The genomic G+C content of the type strain is 61.3%. Its approximate genome size is 4.52 Mbp.

#### Emended Description of *Nitratireductor indicus* Lai et al. 2011

The description is as before (Lai et al., 2011c) with the following modification. The genomic G+C content of the type strain is 60.9%. Its approximate genome size is 4.99 Mbp.

#### Emended Description of *Nitratireductor pacificus* Lai et al. 2011

The description is as before (Lai et al., 2011b) with the following modification. The genomic G+C content of the type strain is 65.5%. Its approximate genome size is 4.47 Mbp.

#### Emended Description of *Nitrobacter winogradskyi* Winslow et al. 1917 (Approved Lists 1980)

The description is as before (Winslow et al., 1917) with the following addition. The genomic G+C content of the type strain is 62.0%. Its approximate genome size is 3.40 Mbp.

#### Emended Description of *Novosphingobium acidiphilum* Glaeser et al. 2009

The description is as before (Glaeser et al., 2009) with the following addition. The genomic G+C content of the type strain is 64.3%. Its approximate genome size is 3.71 Mbp.

#### Emended Description of *Novosphingobium aromaticivorans* (Balkwill et al. 1997) Takeuchi et al. 2001

The description is as before (Takeuchi et al., 2001) with the following restriction. The genomic G+C content of the type strain is 65.1%. Its approximate genome size is 4.23 Mbp.

#### Emended Description of *Novosphingobium barchaimii* Niharika et al. 2013

The description is as before (Niharika et al., 2013b) with the following modification. The genomic G+C content of the type strain is 64.0%. Its approximate genome size is 5.31 Mbp.

#### Emended Description of *Novosphingobium capsulatum* (Leifson 1962) Takeuchi et al. 2001

The description is as before (Takeuchi et al., 2001) with the following modification. The genomic G+C content of the type strain is 65.7%. Its approximate genome size is 4.84 Mbp.

#### Emended Description of *Novosphingobium fuchskuhlense* Glaeser et al. 2013

The description is as before (Glaeser et al., 2013) with the following addition. The genomic G+C content of the type strain is 65.4%. Its approximate genome size is 3.96 Mbp.

#### Emended Description of *Novosphingobium guangzhouense* Sha et al. 2017

The description is as before (Sha et al., 2017) with the following modification. The G+C content of the type-strain genome is 63.5%, its approximate size 5.97 Mbp.

#### Emended Description of *Novosphingobium lindaniclasticum* Saxena et al. 2013

The description is as before (Saxena et al., 2013) with the following modification. The genomic G+C content of the type strain is 64.6%. Its approximate genome size is 4.86 Mbp.

#### Emended Description of *Novosphingobium mathurense* Gupta et al. 2009

The description is as before (Gupta et al., 2009) with the following addition. The genomic G+C content of the type strain is 63.3%. Its approximate genome size is 4.84 Mbp.

#### Emended Description of *Novosphingobium nitrogenifigens* Addison et al. 2007

The description is as before (Addison et al., 2007) with the following addition. The genomic G+C content of the type strain is 64.0%. Its approximate genome size is 4.15 Mbp.

#### Emended Description of *Novosphingobium pentaromativorans* Sohn et al. 2004

The description is as before (Sohn et al., 2004) with the following modification. The genomic G+C content of the type strain is 63.0%. Its approximate genome size is 5.46 Mbp.

#### Emended Description of *Novosphingobium subarcticum* (Nohynek et al. 1996) Takeuchi et al. 2001

The description is as before (Takeuchi et al., 2001) with the following restriction. The genomic G+C content of the type strain is 65.1%. Its approximate genome size is 6.30 Mbp.

#### Emended Description of *Novosphingobium subterraneum* (Balkwill et al. 1997) Takeuchi et al. 2001

The description is as before (Takeuchi et al., 2001) with the following modification. The genomic G+C content of the type strain is 63.3%. Its approximate genome size is 4.70 Mbp.

#### Emended Description of *Oceanibacterium hippocampi* Balcázar et al. 2013

The description is as before (Balcázar et al., 2012) with the following modification. The genomic G+C content of the type strain is 66.7%. Its approximate genome size is 4.83 Mbp.

#### Emended Description of *Oceanibaculum pacificum* Dong et al. 2010

The description is as before (Dong et al., 2010) with the following modification. The genomic G+C content of the type strain is 65.7%. Its approximate genome size is 3.89 Mbp.

#### Emended Description of *Oceanicella actignis* Albuquerque et al. 2012

The description is as before (Albuquerque et al., 2012) with the following modification. The genomic G+C content of the type strain is 72.4%. Its approximate genome size is 3.28 Mbp.

#### Emended Description of *Oceanicola granulosus* Cho and Giovannoni 2004

The description is as before (Cho and Giovannoni, 2004) with the following modification. The genomic G+C content of the type strain is 70.4%. Its approximate genome size is 4.04 Mbp.

#### Emended Description of *Octadecabacter antarcticus* Gosink et al. 1998 emend. Billerbeck et al. 2015

The description is as before (Billerbeck et al., 2015) with the following modification. The genomic G+C content of the type strain is 54.6%. Its approximate genome size is 4.88 Mbp.

#### Emended Description of *Octadecabacter arcticus* Gosink et al. 1998 emend. Billerbeck et al. 2015

The description is as before (Billerbeck et al., 2015) with the following modification. The genomic G+C content of the type strain is 55.1%. Its approximate genome size is 5.48 Mbp.

#### Emended Description of *Octadecabacter ascidiaceicola* Kim et al. 2016

The description is as before (Kim et al., 2016a) with the following modification. The G+C content of the type-strain genome is 54.9%, its approximate size 3.23 Mbp.

#### Emended Description of *Oharaeibacter diazotrophicus* Lv et al. 2017

The description is as before (Lv et al., 2017) with the following modification. The G+C content of the type-strain genome is 71.6%, its approximate size 4.99 Mbp.

#### Emended Description of *Orientia tsutsugamushi* (Hayashi 1920) Tamura et al. 1995

The description is as before (Tamura et al., 1995) with the following restriction. The genomic G+C content of the type strain is 29.9%. Its approximate genome size is 1.45 Mbp.

#### Emended Description of *Pacificibacter marinus* (Jung et al. 2011) Park et al. 2015

The description is as before (Park et al., 2014c) with the following modification. The genomic G+C content of the type strain is 52.0%. Its approximate genome size is 3.87 Mbp.

#### Emended Description of *Palleronia marisminoris* Martínez-Checa et al. 2005 emend. Albuquerque et al. 2015

The description is as before (Albuquerque et al., 2015) with the following modification. The genomic G+C content of the type strain is 66.2%. Its approximate genome size is 3.94 Mbp.

#### Emended Description of *Pannonibacter phragmitetus* Borsodi et al. 2003

The description is as before (Borsodi et al., 2003) with the following modification. The genomic G+C content of the type strain is 63.1%. Its approximate genome size is 4.78 Mbp.

#### Emended Description of *Paracoccus aestuarii* Roh et al. 2009

The description is as before (Roh et al., 2009) with the following modification. The G+C content of the type-strain genome is 67.7%, its approximate size 3.74 Mbp.

#### Emended Description of *Paracoccus alcaliphilus* Urakami et al. 1989

The description is as before (Urakami et al., 1989) with the following restriction. The genomic G+C content of the type strain is 64.3%. Its approximate genome size is 4.61 Mbp.

#### Emended Description of *Paracoccus alkenifer* Lipski et al. 1998

The description is as before (Lipski et al., 1998) with the following addition. The genomic G+C content of the type strain is 67.3%. Its approximate genome size is 3.19 Mbp.

#### Emended Description of *Paracoccus bengalensis* Ghosh et al. 2006

The description is as before (Ghosh et al., 2006) with the following modification. The G+C content of the type-strain genome is 67.4%, its approximate size 4.99 Mbp.

#### Emended Description of *Paracoccus contaminans* Kämpfer et al. 2016

The description is as before (Kämpfer et al., 2016) with the following modification. The genomic G+C content of the type strain is 68.7%. Its approximate genome size is 3.03 Mbp.

#### Emended Description of *Paracoccus denitrificans* (Beijerinck and Minkman 1910) Davis 1969 (Approved Lists 1980) emend. Rainey et al., 1999

The description is as before (Rainey et al., 1999) with the following restriction. The genomic G+C content of the type strain is 66.8%. Its approximate genome size is 5.19 Mbp.

#### Emended Description of *Paracoccus halophilus* Liu et al. 2008

The description is as before (Liu et al., 2008) with the following modification. The genomic G+C content of the type strain is 65.2%. Its approximate genome size is 4.01 Mbp.

#### Emended Description of *Paracoccus isoporae* Chen et al. 2011

The description is as before (Chen et al., 2011a) with the following modification. The genomic G+C content of the type strain is 65.8%. Its approximate genome size is 3.52 Mbp.

#### Emended Description of *Paracoccus pantotrophus* (Robertson and Kuenen 1984) Rainey et al. 1999

The description is as before (Rainey et al., 1999) with the following modification. The G+C content of the type-strain genome is 67.6%, its approximate size 4.41 Mbp.

#### Emended Description of *Paracoccus sediminis* Pan et al. 2014

The description is as before (Pan et al., 2014) with the following modification. The genomic G+C content of the type strain is 66.0%. Its approximate genome size is 3.65 Mbp.

#### Emended Description of *Paracoccus seriniphilus* Pukall et al. 2003

The description is as before (Pukall et al., 2003) with the following modification. The genomic G+C content of the type strain is 61.6%. Its approximate genome size is 4.20 Mbp.

#### Emended Description of *Paracoccus solventivorans* Siller et al. 1996 emend. Lipski et al. 1998

The description is as before (Lipski et al., 1998) with the following restriction. The genomic G+C content of the type strain is 68.7%. Its approximate genome size is 3.38 Mbp.

#### Emended Description of *Paracoccus sphaerophysae* Deng et al. 2011

The description is as before (Deng et al., 2011) with the following modification. The genomic G+C content of the type strain is 69.1%. Its approximate genome size is 3.36 Mbp.

#### Emended Description of *Paracoccus yeei* Daneshvar et al. 2003

The description is as before (Daneshvar et al., 2003) with the following modification. The genomic G+C content of the type strain is 67.5%. Its approximate genome size is 4.43 Mbp.

#### Emended Description of *Pararhizobium giardinii* (Amarger et al. 1997) Mousavi et al. 2016

The description is as before (Mousavi et al., 2015) with the following addition. The genomic G+C content of the type strain is 60.7%. Its approximate genome size is 6.81 Mbp.

#### Emended Description of *Pararhodospirillum photometricum* (Molisch 1907) Lakshmi et al. 2014

The description is as before (Lakshmi et al., 2014) with the following addition. The genomic G+C content of the type strain is 64.7%. Its approximate genome size is 3.88 Mbp.

#### Emended Description of *Parvibaculum lavamentivorans* Schleheck et al. 2004

The description is as before (Schleheck et al., 2004) with the following modification. The genomic G+C content of the type strain is 62.3%. Its approximate genome size is 3.91 Mbp.

#### Emended Description of *Pelagibaca bermudensis* Cho and Giovannoni 2006

The description is as before (Cho and Giovannoni, 2006) with the following modification. The genomic G+C content of the type strain is 66.4%. Its approximate genome size is 5.43 Mbp.

#### Emended Description of *Pelagibacterium halotolerans* Xu et al. 2011

The description is as before (Xu et al., 2011) with the following modification. The genomic G+C content of the type strain is 61.4%. Its approximate genome size is 3.95 Mbp.

#### Emended Description of *Pelagibacterium luteolum* Xu et al. 2011

The description is as before (Xu et al., 2011) with the following modification. The genomic G+C content of the type strain is 60.4%. Its approximate genome size is 4.30 Mbp.

#### Emended Description of *Pelagicola litoralis* Kim et al. 2008

The description is as before (Kim Y.-G. et al., 2008) with the following modification. The genomic G+C content of the type strain is 54.8%. Its approximate genome size is 3.64 Mbp.

#### Emended Description of *Phaeobacter gallaeciensis* (Ruiz-Ponte et al. 1998) Martens et al. 2006

The description is as before (Martens et al., 2006) with the following modification. The genomic G+C content of the type strain is 59.4%. Its approximate genome size is 4.54 Mbp.

#### Emended Description of *Phaeobacter italicus* (Vandecandelaere et al. 2009) Wirth and Whitman 2018

The description is as before (Wirth and Whitman, 2018) with the following restriction. The G+C content of the type-strain genome is 60.0%, its approximate size 4.06 Mbp.

#### Emended Description of *Phenylobacterium composti* Weon et al. 2008

The description is as before (Weon et al., 2008a) with the following modification. The genomic G+C content of the type strain is 70.0%. Its approximate genome size is 3.30 Mbp.

#### Emended Description of *Phenylobacterium deserti* Khan et al. 2017

The description is as before (Khan et al., 2017) with the following modification. The G+C content of the type-strain genome is 68.2%, its approximate size 3.87 Mbp.

#### Emended Description of *Phenylobacterium immobile* Lingens et al. 1985

The description is as before (Lingens et al., 1985) with the following modification. The genomic G+C content of the type strain is 66.7%. Its approximate genome size is 3.33 Mbp.

#### Emended Description of *Phenylobacterium kunshanense* Chu et al. 2015

The description is as before (Chu et al., 2015) with the following modification. The G+C content of the type-strain genome is 69.3%, its approximate size 4.18 Mbp.

#### Emended Description of *Phyllobacterium brassicacearum* Mantelin et al. 2006

The description is as before (Mantelin et al., 2006) with the following modification. The G+C content of the type-strain genome is 57.3%, its approximate size 5.79 Mbp.

#### Emended Description of *Phyllobacterium endophyticum* Flores-Félix et al. 2013

The description is as before (Flores-Félix et al., 2013) with the following modification. The G+C content of the type-strain genome is 57.0%, its approximate size 5.51 Mbp.

#### Emended Description of *Phyllobacterium leguminum* Mantelin et al. 2006

The description is as before (Mantelin et al., 2006) with the following modification. The G+C content of the type-strain genome is 59.8%, its approximate size 3.72 Mbp.

#### Emended Description of *Phyllobacterium rubiacearum* (ex von Faber 1912) Knösel 1984

The description is as before (Knösel, 1984) with the following modification. The G+C content of the type-strain genome is 59.2%, its approximate size 5.58 Mbp.

#### Emended Description of *Phyllobacterium salinisoli* León-Barrios et al. 2018

The description is as before (León-Barrios et al., 2018) with the following modification. The G+C content of the type-strain genome is 59.9%, its approximate size 5.04 Mbp.

#### Emended Description of *Phyllobacterium sophorae* Jiao et al. 2015

The description is as before (Jiao et al., 2015) with the following modification. The G+C content of the type-strain genome is 57.0%, its approximate size 6.36 Mbp.

#### Emended Description of *Pleomorphomonas diazotrophica* Madhaiyan et al. 2013

The description is as before (Madhaiyan et al., 2013) with the following modification. The G+C content of the type-strain genome is 65.1%, its approximate size 4.53 Mbp.

#### Emended Description of *Pleomorphomonas koreensis* Im et al. 2006

The description is as before (Im et al., 2006) with the following modification. The genomic G+C content of the type strain is 67.2%. Its approximate genome size is 4.62 Mbp.

#### Emended Description of *Pontibaca methylaminivorans* Kim et al. 2010

The description is as before (Kim K.K. et al., 2010) with the following modification. The genomic G+C content of the type strain is 66.5%. Its approximate genome size is 2.65 Mbp.

#### Emended Description of *Ponticaulis koreensis* Kang and Lee 2009

The description is as before (Kang and Lee, 2009) with the following modification. The genomic G+C content of the type strain is 54.8%. Its approximate genome size is 3.40 Mbp.

#### Emended Description of *Ponticoccus lacteus* Yang et al. 2015

The description is as before (Yang Y. et al., 2015) with the following modification. The genomic G+C content of the type strain is 65.1%. Its approximate genome size is 5.42 Mbp.

#### Emended Description of *Porphyrobacter cryptus* da Costa et al. 2003

The description is as before (Rainey et al., 2003) with the following modification. The genomic G+C content of the type strain is 67.9%. Its approximate genome size is 2.95 Mbp.

#### Emended Description of *Porphyrobacter neustonensis* Fuerst et al. 1993

The description is as before (Fuerst et al., 1993) with the following modification. The genomic G+C content of the type strain is 65.3%. Its approximate genome size is 3.09 Mbp.

#### Emended Description of *Primorskyibacter sedentarius* Romanenko et al. 2011

The description is as before (Romanenko et al., 2011b) with the following restriction. The G+C content of the type-strain genome is 60.8%, its approximate size 5.06 Mbp.

#### Emended Description of *Prosthecomicrobium hirschii* Staley 1984

The description is as before (Staley, 1984) with the following restriction. The genomic G+C content of the type strain is 68.9%. Its approximate genome size is 6.46 Mbp.

#### Emended Description of *Pseudaminobacter salicylatoxidans* Kämpfer et al. 1999

The description is as before (Kämpfer et al., 1999) with the following modification. The G+C content of the type-strain genome is 62.7%, its approximate size 4.84 Mbp.

#### Emended Description of *Pseudodonghicola xiamenensis* (Tan et al. 2009) Hameed et al. 2014

The description is as before (Hameed et al., 2014) with the following modification. The genomic G+C content of the type strain is 63.6%. Its approximate genome size is 4.73 Mbp.

#### Emended Description of *Pseudolabrys taiwanensis* Kämpfer et al. 2006

The description is as before (Kämpfer et al., 2006) with the following modification. The G+C content of the type-strain genome is 64.4%, its approximate size 5.59 Mbp.

#### Emended Description of *Pseudooceanicola antarcticus* (Huo et al. 2014) Lai et al. 2015

The description is as before (Lai et al., 2015) with the following modification. The G+C content of the type-strain genome is 66.3%, its approximate size 4.23 Mbp.

#### Emended Description of *Pseudooceanicola batsensis* (Cho and Giovannoni 2004) Lai et al. 2015

The description is as before (Lai et al., 2015) with the following modification. The genomic G+C content of the type strain is 66.1%. Its approximate genome size is 4.44 Mbp.

#### Emended Description of *Pseudooceanicola marinus* (Lin et al. 2007) Lai et al. 2015

The description is as before (Lai et al., 2015) with the following modification. The genomic G+C content of the type strain is 66.8%. Its approximate genome size is 4.53 Mbp.

#### Emended Description of *Pseudooceanicola nanhaiensis* (Gu et al. 2007) Lai et al. 2015

The description is as before (Lai et al., 2015) with the following modification. The genomic G+C content of the type strain is 67.9%. Its approximate genome size is 4.66 Mbp.

#### Emended Description of *Pseudooceanicola nitratireducens* (Zheng et al. 2010) Lai et al. 2015

The description is as before (Lai et al., 2015) with the following modification. The genomic G+C content of the type strain is 64.2%. Its approximate genome size is 4.07 Mbp.

#### Emended Description of *Pseudorhodobacter antarcticus* Chen et al. 2013

The description is as before (Chen C.-X. et al., 2013) with the following modification. The genomic G+C content of the type strain is 59.1%. Its approximate genome size is 3.88 Mbp.

#### Emended Description of *Pseudorhodobacter aquimaris* Jung et al. 2012

The description is as before (Jung et al., 2012a) with the following modification. The genomic G+C content of the type strain is 58.7%. Its approximate genome size is 3.82 Mbp.

#### Emended Description of *Pseudorhodobacter wandonensis* Lee et al. 2013

The description is as before (Lee et al., 2013) with the following modification. The genomic G+C content of the type strain is 57.8%. Its approximate genome size is 3.89 Mbp.

#### Emended Description of *Pseudoroseovarius halocynthiae* (Kim et al. 2012) Sun et al. 2015

The description is as before (Sun et al., 2015) with the following modification. The genomic G+C content of the type strain is 57.1%. Its approximate genome size is 3.40 Mbp.

#### Emended Description of *Pseudoroseovarius sediminilitoris* (Park and Yoon 2013) Sun et al. 2015

The description is as before (Sun et al., 2015) with the following modification. The genomic G+C content of the type strain is 58.7%. Its approximate genome size is 3.41 Mbp.

#### Emended Description of *Pseudoruegeria haliotis* Hyun et al. 2013

The description is as before (Hyun et al., 2013b) with the following modification. The genomic G+C content of the type strain is 63.0%. Its approximate genome size is 5.04 Mbp.

#### Emended Description of *Pseudoruegeria lutimaris* Jung et al. 2010

The description is as before (Jung et al., 2010) with the following modification. The genomic G+C content of the type strain is 62.3%. Its approximate genome size is 5.81 Mbp.

#### Emended Description of *Pseudoruegeria sabulilitoris* Park et al. 2014

The description is as before (Park et al., 2014a) with the following modification. The genomic G+C content of the type strain is 62.4%. Its approximate genome size is 5.32 Mbp.

#### Emended Description of *Puniceibacterium sediminis* Zhang et al. 2015

The description is as before (Zhang D.-C. et al., 2015) with the following modification. The genomic G+C content of the type strain is 60.8%. Its approximate genome size is 4.64 Mbp.

#### Emended Description of *Reyranella massiliensis* Pagnier et al. 2011

The description is as before (Pagnier et al., 2011) with the following addition. The genomic G+C content of the type strain is 64.9%. Its approximate genome size is 5.77 Mbp.

#### Emended Description of *Rhizobium altiplani* Baraúna et al. 2016

The description is as before (Baraúna et al., 2016) with the following modification. The G+C content of the type-strain genome is 59.5%, its approximate size 8.10 Mbp.

#### Emended Description of *Rhizobium esperanzae* Cordeiro et al. 2017

The description is as before (Cordeiro et al., 2017) with the following modification. The G+C content of the type-strain genome is 61.1%, its approximate size 6.29 Mbp.

#### Emended Description of *Rhizobium etli* Segovia et al. 1993

The description is as before (Segovia et al., 1993) with the following addition. The genomic G+C content of the type strain is 61.0%. Its approximate genome size is 6.53 Mbp.

#### Emended Description of *Rhizobium flavum* Gu et al. 2014

The description is as before (Gu et al., 2014) with the following modification. The genomic G+C content of the type strain is 61.6%. Its approximate genome size is 4.66 Mbp.

#### Emended Description of *Rhizobium hainanense* Chen et al. 1997

The description is as before (Chen et al., 1997) with the following modification. The genomic G+C content of the type strain is 59.6%. Its approximate genome size is 7.25 Mbp.

#### Emended Description of *Rhizobium jaguaris* Rincón-Rosales et al. 2013

The description is as before (Rincón-Rosales et al., 2013) with the following addition. The G+C content of the type-strain genome is 59.4%, its approximate size 8.03 Mbp.

#### Emended Description of *Rhizobium leguminosarum* (Frank 1879) Frank 1889 (Approved Lists 1980) emend. Ramírez-Bahena et al. 2008

The description is as before (Ramírez-Bahena et al., 2008) with the following modification. The genomic G+C content of the type strain is 60.6%. Its approximate genome size is 7.81 Mbp.

#### Emended Description of *Rhizobium leucaenae* Ribeiro et al. 2012

The description is as before (Ribeiro et al., 2012) with the following modification. The genomic G+C content of the type strain is 59.4%. Its approximate genome size is 6.68 Mbp.

#### Emended Description of *Rhizobium lusitanum* Valverde et al. 2006

The description is as before (Valverde et al., 2006) with the following modification. The genomic G+C content of the type strain is 59.6%. Its approximate genome size is 7.92 Mbp.

#### Emended Description of *Rhizobium miluonense* Gu et al. 2008

The description is as before (Gu et al., 2008) with the following modification. The genomic G+C content of the type strain is 59.7%. Its approximate genome size is 6.81 Mbp.

#### Emended Description of *Rhizobium mongolense* van Berkum et al. 1998

The description is as before (van Berkum et al., 1998) with the following addition. The genomic G+C content of the type strain is 59.5%. Its approximate genome size is 7.17 Mbp.

#### Emended Description of *Rhizobium multihospitium* Han et al. 2008

The description is as before (Han et al., 2008) with the following modification. The genomic G+C content of the type strain is 59.8%. Its approximate genome size is 7.32 Mbp.

#### Emended Description of *Rhizobium rhizogenes* (Riker et al. 1930) Young et al. 2001

The description is as before (Young et al., 2001) with the following restriction. The genomic G+C content of the type strain is 59.9%. Its approximate genome size is 7.04 Mbp.

#### Emended Description of *Rhizobium selenitireducens* Hunter et al. 2008

The description is as before (Hunter et al., 2007) with the following modification. The genomic G+C content of the type strain is 63.5%. Its approximate genome size is 4.97 Mbp.

#### Emended Description of *Rhizobium tropici* Martínez-Romero et al. 1991

The description is as before (Martínez-Romero et al., 1991) with the following addition. The genomic G+C content of the type strain is 59.5%. Its approximate genome size is 6.69 Mbp.

#### Emended Description of *Rhodobacter aestuarii* Venkata Ramana et al. 2009

The description is as before (Venkata Ramana et al., 2009) with the following modification. The genomic G+C content of the type strain is 61.1%. Its approximate genome size is 3.84 Mbp.

#### Emended Description of *Rhodobacter blasticus* (Eckersley and Dow 1981) Kawasaki et al. 1994

The description is as before (Kawasaki et al., 1993) with the following modification. The G+C content of the type-strain genome is 66.5%, its approximate size 3.59 Mbp.

#### Emended Description of *Rhodobacter capsulatus* (Molisch 1907) Imhoff et al. 1984

The description is as before (Imhoff et al., 1984) with the following addition. The genomic G+C content of the type strain is 66.5%. Its approximate genome size is 3.67 Mbp.

#### Emended Description of *Rhodobacter maris* Venkata Ramana et al. 2008

The description is as before (Venkata Ramana et al., 2008) with the following modification. The G+C content of the type-strain genome is 65.1%, its approximate size 3.83 Mbp.

#### Emended Description of *Rhodobacter megalophilus* Arunasri et al. 2008

The description is as before (Arunasri et al., 2008) with the following modification. The genomic G+C content of the type strain is 68.8%. Its approximate genome size is 4.86 Mbp.

#### Emended Description of *Rhodobacter viridis* Shalem Raj et al. 2013

The description is as before (Shalem Raj et al., 2013) with the following modification. The G+C content of the type-strain genome is 65.4%, its approximate size 3.86 Mbp.

#### Emended Description of *Rhodoblastus acidophilus* (Pfennig 1969) Imhoff 2001

The description is as before (Imhoff, 2001) with the following restriction. The genomic G+C content of the type strain is 65.2%. Its approximate genome size is 4.71 Mbp.

#### Emended Description of *Rhodoblastus sphagnicola* Kulichevskaya et al. 2006

The description is as before (Kulichevskaya et al., 2006) with the following modification. The G+C content of the type-strain genome is 63.9%, its approximate size 5.09 Mbp.

#### Emended Description of *Rhodomicrobium udaipurense* Ramana et al. 2013

The description is as before (Ramana V.V. et al., 2013) with the following modification. The genomic G+C content of the type strain is 62.5%. Its approximate genome size is 3.63 Mbp.

#### Emended Description of *Rhodomicrobium vannielii* Duchow and Douglas 1949 (Approved Lists 1980)

The description is as before (Duchow and Douglas, 1949) with the following addition. The genomic G+C content of the type strain is 62.2%. Its approximate genome size is 4.01 Mbp.

#### Emended Description of *Rhodopseudomonas pentothenatexigens* Kumar et al. 2013

The description is as before (Kumar et al., 2013) with the following modification. The G+C content of the type-strain genome is 66.0%, its approximate size 5.38 Mbp.

#### Emended Description of *Rhodopseudomonas pseudopalustris* Venkata Ramana et al. 2012

The description is as before (Venkata Ramana et al., 2012) with the following modification. The genomic G+C content of the type strain is 64.6%. Its approximate genome size is 5.27 Mbp.

#### Emended Description of *Rhodospira trueperi* Pfennig et al. 1998

The description is as before (Pfennig et al., 1997) with the following modification. The genomic G+C content of the type strain is 67.0%. Its approximate genome size is 4.21 Mbp.

#### Emended Description of *Rhodospirillum rubrum* (Esmarch 1887) Molisch 1907 (Approved Lists 1980)

The description is as before (Molisch, 1907) with the following addition. The genomic G+C content of the type strain is 65.4%. Its approximate genome size is 4.41 Mbp.

#### Emended Description of *Rhodothalassium salexigens* (Drews 1982) Imhoff et al. 1998

The description is as before (Imhoff et al., 1998) with the following restriction. The G+C content of the type-strain genome is 68.6%, its approximate size 3.35 Mbp.

#### Emended Description of *Rhodovibrio salinarum* (Nissen and Dundas 1985) Imhoff et al. 1998

The description is as before (Imhoff et al., 1998) with the following modification. The genomic G+C content of the type strain is 66.0%. Its approximate genome size is 4.18 Mbp.

#### Emended Description of *Rhodovulum euryhalinum* (Kompantseva 1989) Hiraishi and Ueda 1994

The description is as before (Hiraishi and Ueda, 1994a) with the following modification. The G+C content of the type-strain genome is 67.9%, its approximate size 3.78 Mbp.

#### Emended Description of *Rhodovulum imhoffii* Srinivas et al. 2007

The description is as before (Srinivas et al., 2007a) with the following modification. The genomic G+C content of the type strain is 63.1%. Its approximate genome size is 2.91 Mbp.

#### Emended Description of *Rhodovulum kholense* Anil Kumar et al. 2008

The description is as before (Anil Kumar et al., 2008) with the following modification. The genomic G+C content of the type strain is 67.8%. Its approximate genome size is 4.46 Mbp.

#### Emended Description of *Rhodovulum marinum* Srinivas et al. 2006

The description is as before (Srinivas et al., 2006) with the following modification. The G+C content of the type-strain genome is 67.2%, its approximate size 3.94 Mbp.

#### Emended Description of *Rhodovulum viride* Srinivas et al. 2014

The description is as before (Srinivas et al., 2014) with the following modification. The G+C content of the type-strain genome is 67.7%, its approximate size 4.52 Mbp.

#### Emended Description of *Rickettsia australis* Philip 1950 (Approved Lists 1980)

The description is as before (Philip, 1950) with the following addition. The genomic G+C content of the type strain is 32.3%. Its approximate genome size is 1.32 Mbp.

#### Emended Description of *Rickettsia bellii* Philip et al. 1983

The description is as before (Philip et al., 1983) with the following modification. The genomic G+C content of the type strain is 31.6%. Its approximate genome size is 1.52 Mbp.

#### Emended Description of *Rickettsia conorii* Brumpt 1932 (Approved Lists 1980)

The description is as before (Brumpt, 1932) with the following addition. The genomic G+C content of the type strain is 32.4%. Its approximate genome size is 1.27 Mbp.

#### Emended Description of *Rickettsia heilongjiangensis* Fournier et al. 2006

The description is as before (Fournier et al., 2003) with the following addition. The G+C content of the type-strain genome is 32.3%, its approximate size 1.28 Mbp.

#### Emended Description of *Rickettsia honei* Stenos et al. 1998

The description is as before (Stenos et al., 1998) with the following addition. The genomic G+C content of the type strain is 32.4%. Its approximate genome size is 1.27 Mbp.

#### Emended Description of *Rickettsia hoogstraalii* Duh et al. 2010

The description is as before (Duh et al., 2010) with the following addition. The genomic G+C content of the type strain is 32.4%. Its approximate genome size is 1.48 Mbp.

#### Emended Description of *Rickettsia japonica* Uchida et al. 1992

The description is as before (Uchida et al., 1992) with the following restriction. The genomic G+C content of the type strain is 32.4%. Its approximate genome size is 1.28 Mbp.

#### Emended Description of *Rickettsia prowazekii* Rocha-Lima 1916 (Approved Lists 1980)

The description is as before (da Rocha-Lima, 1916) with the following addition. The genomic G+C content of the type strain is 29.0%. Its approximate genome size is 1.11 Mbp.

#### Emended Description of *Rickettsia raoultii* Mediannikov et al. 2008

The description is as before (Mediannikov et al., 2008) with the following modification. The G+C content of the type-strain genome is 32.6%, its approximate size 1.48 Mbp.

#### Emended Description of *Rickettsia sibirica* Zdrodovskii 1948 (Approved Lists 1980)

The description is as before (Zdrodovskii, 1948) with the following addition. The genomic G+C content of the type strain is 32.5%. Its approximate genome size is 1.25 Mbp.

#### Emended Description of *Rickettsia slovaca* Sekeyová et al. 1998

The description is as before (Sekeyová et al., 1998) with the following addition. The genomic G+C content of the type strain is 32.5%. Its approximate genome size is 1.28 Mbp.

#### Emended Description of *Rickettsia tamurae* Fournier et al. 2006

The description is as before (Fournier et al., 2006) with the following addition. The genomic G+C content of the type strain is 32.5%. Its approximate genome size is 1.45 Mbp.

#### Emended Description of *Rickettsia typhi* (Wolbach and Todd 1920) Philip 1943 (Approved Lists 1980)

The description is as before (Philip, 1943) with the following addition. The genomic G+C content of the type strain is 28.9%. Its approximate genome size is 1.11 Mbp.

#### Emended Description of *Robiginitomaculum antarcticum* Lee et al. 2007

The description is as before (Lee et al., 2007d) with the following modification. The genomic G+C content of the type strain is 52.5%. Its approximate genome size is 2.77 Mbp.

#### Emended Description of *Roseiarcus fermentans* Kulichevskaya et al. 2014

The description is as before (Kulichevskaya et al., 2014) with the following modification. The G+C content of the type-strain genome is 68.2%, its approximate size 6.79 Mbp.

#### Emended Description of *Roseibacterium elongatum* Suzuki et al. 2006

The description is as before (Suzuki et al., 2006) with the following modification. The genomic G+C content of the type strain is 65.7%. Its approximate genome size is 3.56 Mbp.

#### Emended Description of *Roseibium denhamense* Suzuki et al. 2000 emend. Biebl et al. 2007

The description is as before (Biebl et al., 2007) with the following restriction. The G+C content of the type-strain genome is 57.3%, its approximate size 4.85 Mbp.

#### Emended Description of *Roseibium hamelinense* Suzuki et al. 2000 emend. Biebl et al. 2007

The description is as before (Biebl et al., 2007) with the following restriction. The G+C content of the type-strain genome is 56.4%, its approximate size 4.72 Mbp.

#### Emended Description of *Roseinatronobacter thiooxidans* Sorokin et al. 2000

The description is as before (Sorokin et al., 2000) with the following modification. The genomic G+C content of the type strain is 60.1%. Its approximate genome size is 3.68 Mbp.

#### Emended Description of *Roseivivax halodurans* Suzuki et al. 1999

The description is as before (Suzuki et al., 1999a) with the following modification. The genomic G+C content of the type strain is 66.3%. Its approximate genome size is 4.49 Mbp.

#### Emended Description of *Roseivivax halotolerans* Suzuki et al. 1999

The description is as before (Suzuki et al., 1999a) with the following modification. The genomic G+C content of the type strain is 64.1%. Its approximate genome size is 3.77 Mbp.

#### Emended Description of *Roseivivax isoporae* Chen et al. 2012

The description is as before (Chen M.-H. et al., 2012) with the following restriction. The genomic G+C content of the type strain is 69.8%. Its approximate genome size is 4.90 Mbp.

#### Emended Description of *Roseivivax jejudonensis* Jung et al. 2015

The description is as before (Jung et al., 2014a) with the following modification. The genomic G+C content of the type strain is 68.0%. Its approximate genome size is 4.31 Mbp.

#### Emended Description of *Roseivivax lentus* Park et al. 2010

The description is as before (Park et al., 2010) with the following modification. The genomic G+C content of the type strain is 66.1%. Its approximate genome size is 4.44 Mbp.

#### Emended Description of *Roseobacter denitrificans* Shiba 1991

The description is as before (Shiba, 1991) with the following modification. The genomic G+C content of the type strain is 58.9%. Its approximate genome size is 4.32 Mbp.

#### Emended Description of *Roseobacter litoralis* Shiba 1991

The description is as before (Shiba, 1991) with the following restriction. The genomic G+C content of the type strain is 57.2%. Its approximate genome size is 4.75 Mbp.

#### Emended Description of *Roseomonas aerilata* Yoo et al. 2008

The description is as before (Yoo et al., 2008) with the following modification. The genomic G+C content of the type strain is 69.7%. Its approximate genome size is 6.43 Mbp.

#### Emended Description of *Roseomonas cervicalis* Rihs et al. 1998 emend. Sánchez-Porro et al. 2009

The description is as before (Sánchez-Porro et al., 2009) with the following modification. The genomic G+C content of the type strain is 72.0%. Its approximate genome size is 4.44 Mbp.

#### Emended Description of *Roseomonas lacus* Jiang et al. 2006 emend. Sánchez-Porro et al. 2009

The description is as before (Sánchez-Porro et al., 2009) with the following modification. The genomic G+C content of the type strain is 68.7%. Its approximate genome size is 6.39 Mbp.

#### Emended Description of *Roseomonas mucosa* Han et al. 2003 emend. Sánchez-Porro et al. 2009

The description is as before (Sánchez-Porro et al., 2009) with the following addition. The genomic G+C content of the type strain is 70.4%. Its approximate genome size is 4.86 Mbp.

#### Emended Description of *Roseomonas rosea* (Kämpfer et al. 2003) Sánchez-Porro et al. 2009

The description is as before (Sánchez-Porro et al., 2009) with the following modification. The genomic G+C content of the type strain is 70.8%. Its approximate genome size is 5.34 Mbp.

#### Emended Description of *Roseomonas stagni* Furuhata et al. 2008

The description is as before (Furuhata et al., 2008) with the following modification. The genomic G+C content of the type strain is 70.7%. Its approximate genome size is 6.38 Mbp.

#### Emended Description of *Roseomonas vinacea* Zhang et al. 2008

The description is as before (Zhang Y.-Q. et al., 2008) with the following modification. The genomic G+C content of the type strain is 70.3%. Its approximate genome size is 6.35 Mbp.

#### Emended Description of *Roseospirillum parvum* Glaeser and Overmann 2001

The description is as before (Glaeser and Overmann, 1999) with the following modification. The genomic G+C content of the type strain is 69.9%. Its approximate genome size is 3.50 Mbp.

#### Emended Description of *Roseovarius azorensis* Rajasabapathy et al. 2014

The description is as before (Rajasabapathy et al., 2014) with the following modification. The genomic G+C content of the type strain is 63.1%. Its approximate genome size is 3.86 Mbp.

#### Emended Description of *Roseovarius confluentis* Jia et al. 2017

The description is as before (Jia et al., 2017) with the following modification. The G+C content of the type-strain genome is 63.2%, its approximate size 4.46 Mbp.

#### Emended Description of *Roseovarius halotolerans* Oh et al. 2009

The description is as before (Oh et al., 2009) with the following modification. The genomic G+C content of the type strain is 63.8%. Its approximate genome size is 3.73 Mbp.

#### Emended Description of *Roseovarius lutimaris* Choi et al. 2013

The description is as before (Choi et al., 2013) with the following modification. The genomic G+C content of the type strain is 59.9%. Its approximate genome size is 4.28 Mbp.

#### Emended Description of *Roseovarius marisflavi* Li et al. 2014

The description is as before (Li Z. et al., 2013) with the following modification. The genomic G+C content of the type strain is 60.3%. Its approximate genome size is 4.08 Mbp.

#### Emended Description of *Roseovarius nanhaiticus* Wang et al. 2010

The description is as before (Wang et al., 2010) with the following modification. The genomic G+C content of the type strain is 63.3%. Its approximate genome size is 3.70 Mbp.

#### Emended Description of *Roseovarius nubinhibens* González et al. 2003

The description is as before (González et al., 2003) with the following modification. The genomic G+C content of the type strain is 63.9%. Its approximate genome size is 3.67 Mbp.

#### Emended Description of *Roseovarius tolerans* Labrenz et al. 1999

The description is as before (Labrenz et al., 1999) with the following restriction. The genomic G+C content of the type strain is 63.1%. Its approximate genome size is 3.77 Mbp.

#### Emended Description of *Rubrimonas cliftonensis* Suzuki et al. 1999

The description is as before (Suzuki et al., 1999b) with the following modification. The genomic G+C content of the type strain is 71.6%. Its approximate genome size is 4.86 Mbp.

#### Emended Description of *Rubritepida flocculans* Alarico et al. 2002

The description is as before (Alarico et al., 2002) with the following modification. The genomic G+C content of the type strain is 73.4%. Its approximate genome size is 3.84 Mbp.

#### Emended Description of *Ruegeria atlantica* (Rüger and Höfle 1992) Uchino et al. 1999 emend. Vandecandelaere et al. 2008

The description is as before (Vandecandelaere et al., 2008a) with the following restriction. The genomic G+C content of the type strain is 56.4%. Its approximate genome size is 4.82 Mbp.

#### Emended Description of *Ruegeria faecimaris* Oh et al. 2011

The description is as before (Oh et al., 2011) with the following modification. The genomic G+C content of the type strain is 56.7%. Its approximate genome size is 4.12 Mbp.

#### Emended Description of *Ruegeria halocynthiae* Kim et al. 2012

The description is as before (Kim et al., 2012b) with the following modification. The genomic G+C content of the type strain is 56.5%. Its approximate genome size is 4.24 Mbp.

#### Emended Description of *Ruegeria intermedia* Kämpfer et al. 2013

The description is as before (Kämpfer et al., 2013a) with the following addition. The genomic G+C content of the type strain is 64.0%. Its approximate genome size is 3.86 Mbp.

#### Emended Description of *Ruegeria lacuscaerulensis* (Petursdottir and Kristjansson 1999) Yi et al. 2007 emend. Vandecandelaere et al. 2008

The description is as before (Vandecandelaere et al., 2008a) with the following modification. The genomic G+C content of the type strain is 63.0%. Its approximate genome size is 3.52 Mbp.

#### Emended Description of *Ruegeria pomeroyi* (González et al. 2003) Yi et al. 2007 emend. Vandecandelaere et al. 2008

The description is as before (Vandecandelaere et al., 2008a) with the following modification. The genomic G+C content of the type strain is 64.1%. Its approximate genome size is 4.60 Mbp.

#### Emended Description of *Saccharibacter floricola* Jojima et al. 2004

The description is as before (Jojima et al., 2004) with the following modification. The genomic G+C content of the type strain is 51.2%. Its approximate genome size is 2.38 Mbp.

#### Emended Description of *Salipiger aestuarii* (Park et al. 2011) Wirth and Whitman 2018

The description is as before (Wirth and Whitman, 2018) with the following modification. The G+C content of the type-strain genome is 64.3%, its approximate size 4.66 Mbp.

#### Emended Description of *Salipiger bermudensis* (Cho and Giovannoni 2006) Wirth and Whitman 2018

The description is as before (Wirth and Whitman, 2018) with the following modification. The G+C content of the type-strain genome is 66.4%, its approximate size 5.43 Mbp.

#### Emended Description of *Salipiger mucosus* Martínez-Cánovas et al. 2004

The description is as before (Martínez-Cánovas et al., 2004) with the following modification. The genomic G+C content of the type strain is 67.3%. Its approximate genome size is 5.67 Mbp.

#### Emended Description of *Salipiger nanhaiensis* Dai et al. 2015

The description is as before (Dai et al., 2015) with the following modification. The genomic G+C content of the type strain is 66.9%. Its approximate genome size is 5.16 Mbp.

#### Emended Description of *Salipiger thiooxidans* (Sorokin et al. 2006) Wirth and Whitman 2018

The description is as before (Wirth and Whitman, 2018) with the following restriction. The G+C content of the type-strain genome is 67.3%, its approximate size 5.87 Mbp.

#### Emended Description of *Sediminispirochaeta smaragdinae* (Magot et al. 1998) Shivani et al. 2016

The description is as before (Shivani et al., 2016) with the following modification. The genomic G+C content of the type strain is 49.0%. Its approximate genome size is 4.65 Mbp.

#### Emended Description of *Shimia abyssi* (Nogi et al. 2016) Wirth and Whitman 2018

The description is as before (Wirth and Whitman, 2018) with the following modification. The G+C content of the type-strain genome is 56.2%, its approximate size 4.73 Mbp.

#### Emended Description of *Shimia haliotis* Hyun et al. 2013

The description is as before (Hyun et al., 2013a) with the following modification. The genomic G+C content of the type strain is 58.0%. Its approximate genome size is 4.00 Mbp.

#### Emended Description of *Shimia isoporae* Chen et al. 2011

The description is as before (Chen et al., 2011b) with the following modification. The genomic G+C content of the type strain is 56.7%. Its approximate genome size is 4.23 Mbp.

#### Emended Description of *Skermanella stibiiresistens* Luo et al. 2012

The description is as before (Luo et al., 2012) with the following modification. The genomic G+C content of the type strain is 65.9%. Its approximate genome size is 7.87 Mbp.

#### Emended Description of *Sneathiella glossodoripedis* Kurahashi et al. 2008

The description is as before (Kurahashi et al., 2008) with the following modification. The genomic G+C content of the type strain is 46.9%. Its approximate genome size is 3.63 Mbp.

#### Emended Description of *Sphaerochaeta coccoides* (Dröge et al. 2006) Abt et al. 2012

The description is as before (Abt et al., 2012) with the following modification. The genomic G+C content of the type strain is 50.6%. Its approximate genome size is 2.23 Mbp.

#### Emended Description of *Sphaerochaeta globosa* Ritalahti et al. 2012

The description is as before (Ritalahti et al., 2012) with the following modification. The genomic G+C content of the type strain is 48.9%. Its approximate genome size is 3.32 Mbp.

#### Emended Description of *Sphaerochaeta pleomorpha* Ritalahti et al. 2012

The description is as before (Ritalahti et al., 2012) with the following restriction. The genomic G+C content of the type strain is 46.2%. Its approximate genome size is 3.59 Mbp.

#### Emended Description of *Sphingobium abikonense* Kumari et al. 2009

The description is as before (Kumari et al., 2009) with the following addition. The genomic G+C content of the type strain is 63.5%. Its approximate genome size is 3.75 Mbp.

#### Emended Description of *Sphingobium amiense* Ushiba et al. 2003

The description is as before (Ushiba et al., 2003) with the following modification. The genomic G+C content of the type strain is 64.7%. Its approximate genome size is 4.54 Mbp.

#### Emended Description of *Sphingobium baderi* Kaur et al. 2013

The description is as before (Kaur et al., 2013) with the following modification. The genomic G+C content of the type strain is 63.6%. Its approximate genome size is 4.69 Mbp.

#### Emended Description of *Sphingobium chinhatense* Dadhwal et al. 2009

The description is as before (Dadhwal et al., 2009) with the following addition. The genomic G+C content of the type strain is 64.1%. Its approximate genome size is 5.85 Mbp.

#### Emended Description of *Sphingobium chlorophenolicum* (Nohynek et al. 1996) Takeuchi et al. 2001

The description is as before (Takeuchi et al., 2001) with the following restriction. The genomic G+C content of the type strain is 64.3%. Its approximate genome size is 4.80 Mbp.

#### Emended Description of *Sphingobium cloacae* (Fujii et al. 2001) Prakash and Lal 2006

The description is as before (Prakash and Lal, 2006) with the following modification. The genomic G+C content of the type strain is 64.6%. Its approximate genome size is 4.29 Mbp.

#### Emended Description of *Sphingobium czechense* Niharika et al. 2013

The description is as before (Niharika et al., 2013a) with the following modification. The genomic G+C content of the type strain is 63.6%. Its approximate genome size is 4.66 Mbp.

#### Emended Description of *Sphingobium faniae* Guo et al. 2010

The description is as before (Guo et al., 2010) with the following restriction. The genomic G+C content of the type strain is 63.3%. Its approximate genome size is 4.84 Mbp.

#### Emended Description of *Sphingobium herbicidovorans* (Zipper et al. 1997) Takeuchi et al. 2001

The description is as before (Takeuchi et al., 2001) with the following addition. The genomic G+C content of the type strain is 62.4%. Its approximate genome size is 4.03 Mbp.

#### Emended Description of *Sphingobium hydrophobicum* Chen et al. 2016

The description is as before (Chen et al., 2016) with the following modification. The genomic G+C content of the type strain is 63.2%. Its approximate genome size is 4.60 Mbp.

#### Emended Description of *Sphingobium indicum* Pal et al. 2005

The description is as before (Pal et al., 2005) with the following addition. The genomic G+C content of the type strain is 65.0%. Its approximate genome size is 4.08 Mbp.

#### Emended Description of *Sphingobium lactosutens* Kumari et al. 2009

The description is as before (Kumari et al., 2009) with the following addition. The genomic G+C content of the type strain is 63.0%. Its approximate genome size is 5.36 Mbp.

#### Emended Description of *Sphingobium lucknowense* Garg et al. 2012

The description is as before (Garg et al., 2012) with the following modification. The genomic G+C content of the type strain is 64.3%. Its approximate genome size is 4.44 Mbp.

#### Emended Description of *Sphingobium quisquiliarum* Bala et al. 2010

The description is as before (Bala et al., 2010) with the following modification. The genomic G+C content of the type strain is 64.0%. Its approximate genome size is 4.17 Mbp.

#### Emended Description of *Sphingobium ummariense* Singh and Lal 2009

The description is as before (Singh and Lal, 2009) with the following modification. The genomic G+C content of the type strain is 65.0%. Its approximate genome size is 4.75 Mbp.

#### Emended Description of *Sphingobium yanoikuyae* (Yabuuchi et al. 1990) Takeuchi et al. 2001

The description is as before (Takeuchi et al., 2001) with the following modification. The genomic G+C content of the type strain is 64.4%. Its approximate genome size is 5.50 Mbp.

#### Emended Description of *Sphingomonas aerolata* Busse et al. 2003

The description is as before (Busse et al., 2003) with the following modification. The G+C content of the type-strain genome is 66.5%, its approximate size 3.83 Mbp.

#### Emended Description of *Sphingomonas aestuarii* Roh et al. 2009

The description is as before (Roh et al., 2009) with the following addition. The genomic G+C content of the type strain is 64.5%. Its approximate genome size is 2.99 Mbp.

#### Emended Description of *Sphingomonas aurantiaca* Busse et al. 2003

The description is as before (Busse et al., 2003) with the following modification. The G+C content of the type-strain genome is 66.2%, its approximate size 4.41 Mbp.

#### Emended Description of *Sphingomonas azotifigens* Xie and Yokota 2006

The description is as before (Xie and Yokota, 2006) with the following restriction. The genomic G+C content of the type strain is 67.3%. Its approximate genome size is 5.15 Mbp.

#### Emended Description of *Sphingomonas echinoides* (Heumann 1962) Denner et al. 1999

The description is as before (Denner et al., 1999) with the following modification. The genomic G+C content of the type strain is 64.7%. Its approximate genome size is 4.18 Mbp.

#### Emended Description of *Sphingomonas faeni* Busse et al. 2003

The description is as before (Busse et al., 2003) with the following modification. The G+C content of the type-strain genome is 64.8%, its approximate size 4.38 Mbp.

#### Emended Description of *Sphingomonas guangdongensis* Feng et al. 2014

The description is as before (Feng et al., 2014) with the following restriction. The G+C content of the type-strain genome is 68.6%, its approximate size 3.54 Mbp.

#### Emended Description of *Sphingomonas hengshuiensis* Wei et al. 2015

The description is as before (Wei et al., 2015) with the following modification. The genomic G+C content of the type strain is 66.7%. Its approximate genome size is 5.23 Mbp.

#### Emended Description of *Sphingomonas jaspsi* Asker et al. 2007

The description is as before (Asker et al., 2007) with the following modification. The genomic G+C content of the type strain is 64.7%. Its approximate genome size is 2.55 Mbp.

#### Emended Description of *Sphingomonas jatrophae* Madhaiyan et al. 2017

The description is as before (Madhaiyan et al., 2013) with the following modification. The G+C content of the type-strain genome is 68.5%, its approximate size 4.06 Mbp.

#### Emended Description of *Sphingomonas laterariae* Kaur et al. 2012

The description is as before (Kaur et al., 2012) with the following modification. The genomic G+C content of the type strain is 65.5%. Its approximate genome size is 4.42 Mbp.

#### Emended Description of *Sphingomonas mali* Takeuchi et al. 1995

The description is as before (Takeuchi et al., 1995) with the following modification. The genomic G+C content of the type strain is 64.9%. Its approximate genome size is 5.23 Mbp.

#### Emended Description of *Sphingomonas melonis* Buonaurio et al. 2002

The description is as before (Buonaurio et al., 2002) with the following modification. The genomic G+C content of the type strain is 67.0%. Its approximate genome size is 4.10 Mbp.

#### Emended Description of *Sphingomonas mucosissima* Reddy and Garcia-Pichel 2007

The description is as before (Reddy and Garcia-Pichel, 2007) with the following addition. The genomic G+C content of the type strain is 65.1%. Its approximate genome size is 3.58 Mbp.

#### Emended Description of *Sphingomonas panacis* Singh et al. 2017

The description is as before (Singh et al., 2016) with the following modification. The genomic G+C content of the type strain is 65.5%. Its approximate genome size is 5.32 Mbp.

#### Emended Description of *Sphingomonas parapaucimobilis* Yabuuchi et al. 1990

The description is as before (Yabuuchi et al., 1990) with the following modification. The genomic G+C content of the type strain is 66.4%. Its approximate genome size is 3.99 Mbp.

#### Emended Description of *Sphingomonas paucimobilis* (Holmes et al. 1977) Yabuuchi et al. 1990

The description is as before (Yabuuchi et al., 1990) with the following restriction. The genomic G+C content of the type strain is 65.7%. Its approximate genome size is 4.33 Mbp.

#### Emended Description of *Sphingomonas phyllosphaerae* Rivas et al. 2004

The description is as before (Rivas et al., 2004) with the following modification. The genomic G+C content of the type strain is 67.2%. Its approximate genome size is 3.92 Mbp.

#### Emended Description of *Sphingomonas pituitosa* Denner et al. 2001

The description is as before (Denner et al., 2001) with the following modification. The genomic G+C content of the type strain is 67.1%. Its approximate genome size is 4.74 Mbp.

#### Emended Description of *Sphingomonas rubra* Huo et al. 2011

The description is as before (Huo et al., 2011) with the following modification. The genomic G+C content of the type strain is 68.8%. Its approximate genome size is 3.20 Mbp.

#### Emended Description of *Sphingomonas sanguinis* Takeuchi et al. 1993

The description is as before (Takeuchi et al., 1993) with the following modification. The genomic G+C content of the type strain is 66.1%. Its approximate genome size is 4.05 Mbp.

#### Emended Description of *Sphingomonas soli* Yang et al. 2006

The description is as before (Yang et al., 2006) with the following modification. The genomic G+C content of the type strain is 65.1%. Its approximate genome size is 3.51 Mbp.

#### Emended Description of *Sphingopyxis bauzanensis* Zhang et al. 2010

The description is as before (Zhang et al., 2010) with the following modification. The genomic G+C content of the type strain is 63.3%. Its approximate genome size is 4.26 Mbp.

#### Emended Description of *Sphingopyxis granuli* Kim et al. 2011

The description is as before (Kim et al., 2005) with the following modification. The genomic G+C content of the type strain is 66.4%. Its approximate genome size is 4.26 Mbp.

#### Emended Description of *Sphingopyxis indica* Jindal et al. 2013

The description is as before (Jindal et al., 2013) with the following modification. The genomic G+C content of the type strain is 65.7%. Its approximate genome size is 4.15 Mbp.

#### Emended Description of *Sphingopyxis macrogoltabida* (Takeuchi et al. 1993) Takeuchi et al. 2001

The description is as before (Takeuchi et al., 2001) with the following restriction. The genomic G+C content of the type strain is 64.9%. Its approximate genome size is 5.75 Mbp.

#### Emended Description of *Sphingopyxis terrae* (Takeuchi et al. 1993) Takeuchi et al. 2001

The description is as before (Takeuchi et al., 2001) with the following restriction. The genomic G+C content of the type strain is 64.6%. Its approximate genome size is 3.98 Mbp.

#### Emended Description of *Sphingopyxis ummariensis* Sharma et al. 2010

The description is as before (Sharma et al., 2010) with the following modification. The genomic G+C content of the type strain is 65.2%. Its approximate genome size is 3.58 Mbp.

#### Emended Description of *Sphingopyxis witflariensis* Kämpfer et al. 2002

The description is as before (Kämpfer et al., 2002b) with the following addition. The genomic G+C content of the type strain is 63.3%. Its approximate genome size is 4.31 Mbp.

#### Emended Description of *Sphingorhabdus litoris* (Kim et al. 2008) Jogler et al. 2013

The description is as before (Jogler et al., 2013) with the following addition. The genomic G+C content of the type strain is 52.7%. Its approximate genome size is 3.61 Mbp.

#### Emended Description of *Sphingorhabdus marina* (Kim et al. 2008) Jogler et al. 2013

The description is as before (Jogler et al., 2013) with the following addition. The genomic G+C content of the type strain is 57.4%. Its approximate genome size is 3.55 Mbp.

#### Emended Description of *Spirochaeta africana* Zhilina et al. 1996

The description is as before (Zhilina et al., 1996) with the following modification. The genomic G+C content of the type strain is 57.8%. Its approximate genome size is 3.29 Mbp.

#### Emended Description of *Spirochaeta cellobiosiphila* Breznak and Warnecke 2008

The description is as before (Breznak and Warnecke, 2008) with the following modification. The genomic G+C content of the type strain is 37.0%. Its approximate genome size is 3.95 Mbp.

#### Emended Description of *Spirochaeta thermophila* Aksenova et al. 1992

The description is as before (Aksenova et al., 1992) with the following modification. The genomic G+C content of the type strain is 60.9%. Its approximate genome size is 2.56 Mbp.

#### Emended Description of *Stakelama pacifica* Chen et al. 2010

The description is as before (Chen C. et al., 2010) with the following modification. The G+C content of the type-strain genome is 62.9%, its approximate size 3.98 Mbp.

#### Emended Description of *Stappia stellulata* (Rüger and Höfle 1992) Uchino et al. 1999 emend. Biebl et al. 2007

The description is as before (Biebl et al., 2007) with the following modification. The genomic G+C content of the type strain is 64.7%. Its approximate genome size is 4.62 Mbp.

#### Emended Description of *Starkeya novella* (Starkey 1934) Kelly et al. 2000

The description is as before (Kelly et al., 2000) with the following restriction. The genomic G+C content of the type strain is 67.9%. Its approximate genome size is 4.77 Mbp.

#### Emended Description of *Stella humosa* Vasilyeva 1985

The description is as before (Vasilyeva, 1985) with the following restriction. The G+C content of the type-strain genome is 69.9%, its approximate size 5.82 Mbp.

#### Emended Description of *Sulfitobacter donghicola* Yoon et al. 2007

The description is as before (Yoon et al., 2007a) with the following modification. The genomic G+C content of the type strain is 55.2%. Its approximate genome size is 3.54 Mbp.

#### Emended Description of *Sulfitobacter dubius* Ivanova et al. 2004

The description is as before (Ivanova et al., 2004) with the following modification. The genomic G+C content of the type strain is 60.2%. Its approximate genome size is 3.67 Mbp.

#### Emended Description of *Sulfitobacter guttiformis* (Labrenz et al. 2000) Yoon et al. 2007

The description is as before (Yoon et al., 2007a) with the following restriction. The genomic G+C content of the type strain is 56.1%. Its approximate genome size is 3.98 Mbp.

#### Emended Description of *Sulfitobacter litoralis* Park et al. 2007

The description is as before (Park et al., 2007) with the following addition. The genomic G+C content of the type strain is 58.5%. Its approximate genome size is 3.68 Mbp.

#### Emended Description of *Sulfitobacter pontiacus* Sorokin 1996

The description is as before (Sorokin, 1995) with the following modification. The genomic G+C content of the type strain is 60.3%. Its approximate genome size is 3.76 Mbp.

#### Emended Description of *Tanticharoenia sakaeratensis* Yukphan et al. 2008

The description is as before (Yukphan et al., 2008) with the following modification. The G+C content of the type-strain genome is 64.2%, its approximate size 3.50 Mbp.

#### Emended Description of *Telmatospirillum siberiense* Sizova et al. 2007

The description is as before (Sizova et al., 2007) with the following modification. The G+C content of the type-strain genome is 62.3%, its approximate size 6.20 Mbp.

#### Emended Description of *Terasakiella pusilla* (Terasaki 1973) Satomi et al. 2002 emend. Han et al. 2016

The description is as before (Han et al., 2016) with the following modification. The genomic G+C content of the type strain is 50.0%. Its approximate genome size is 4.05 Mbp.

#### Emended Description of *Thalassobaculum salexigens* Urios et al. 2010

The description is as before (Urios et al., 2010) with the following modification. The genomic G+C content of the type strain is 67.4%. Its approximate genome size is 5.08 Mbp.

#### Emended Description of *Thalassobius abyssi* Nogi et al. 2016

The description is as before (Nogi et al., 2016) with the following modification. The genomic G+C content of the type strain is 56.2%. Its approximate genome size is 4.73 Mbp.

#### Emended Description of *Thalassobius mediterraneus* Arahal et al. 2005

The description is as before (Arahal et al., 2005) with the following modification. The genomic G+C content of the type strain is 58.7%. Its approximate genome size is 3.41 Mbp.

#### Emended Description of *Thalassospira alkalitolerans* Tsubouchi et al. 2014

The description is as before (Tsubouchi et al., 2014) with the following modification. The genomic G+C content of the type strain is 53.1%. Its approximate genome size is 4.79 Mbp.

#### Emended Description of *Thalassospira lucentensis* López-López et al. 2002

The description is as before (López-López et al., 2002) with the following modification. The genomic G+C content of the type strain is 53.4%. Its approximate genome size is 4.75 Mbp.

#### Emended Description of *Thalassospira povalilytica* Nogi et al. 2014

The description is as before (Nogi et al., 2014) with the following modification. The G+C content of the type-strain genome is 53.6%, its approximate size 4.72 Mbp.

#### Emended Description of *Thalassospira profundimaris* Liu et al. 2007

The description is as before (Liu et al., 2007) with the following modification. The genomic G+C content of the type strain is 55.2%. Its approximate genome size is 4.38 Mbp.

#### Emended Description of *Thalassospira xiamenensis* Liu et al. 2007

The description is as before (Liu et al., 2007) with the following modification. The genomic G+C content of the type strain is 54.7%. Its approximate genome size is 4.77 Mbp.

#### Emended Description of *Thioclava electrotropha* Chang et al. 2018

The description is as before (Chang et al., 2018) with the following addition. The G+C content of the type-strain genome is 63.8%, its approximate size 4.41 Mbp.

#### Emended Description of *Thioclava pacifica* Sorokin et al. 2005

The description is as before (Sorokin et al., 2005b) with the following modification. The genomic G+C content of the type strain is 63.9%. Its approximate genome size is 3.73 Mbp.

#### Emended Description of *Tistlia consotensis* Díaz-Cárdenas et al. 2010

The description is as before (Díaz-Cárdenas et al., 2010) with the following restriction. The genomic G+C content of the type strain is 71.5%. Its approximate genome size is 6.93 Mbp.

#### Emended Description of *Tranquillimonas alkanivorans* Harwati et al. 2008

The description is as before (Harwati et al., 2008) with the following modification. The genomic G+C content of the type strain is 67.3%. Its approximate genome size is 4.54 Mbp.

#### Emended Description of *Treponema berlinense* Nordhoff et al. 2005

The description is as before (Nordhoff et al., 2005) with the following addition. The genomic G+C content of the type strain is 39.4%. Its approximate genome size is 2.52 Mbp.

#### Emended Description of *Treponema brennaborense* Schrank et al. 1999

The description is as before (Schrank et al., 1999) with the following addition. The genomic G+C content of the type strain is 51.5%. Its approximate genome size is 3.06 Mbp.

#### Emended Description of *Treponema denticola* (ex Flügge 1886) Chan et al. 1993

The description is as before (Chan et al., 1993) with the following restriction. The genomic G+C content of the type strain is 37.9%. Its approximate genome size is 2.84 Mbp.

#### Emended Description of *Treponema lecithinolyticum* Wyss et al. 1999

The description is as before (Wyss et al., 1999) with the following addition. The genomic G+C content of the type strain is 43.8%. Its approximate genome size is 2.33 Mbp.

#### Emended Description of *Treponema maltophilum* Wyss et al. 1996

The description is as before (Wyss et al., 1996) with the following addition. The genomic G+C content of the type strain is 47.9%. Its approximate genome size is 2.53 Mbp.

#### Emended Description of *Treponema medium* Umemoto et al. 1997

The description is as before (Umemoto et al., 1997) with the following modification. The genomic G+C content of the type strain is 44.3%. Its approximate genome size is 2.72 Mbp.

#### Emended Description of *Treponema porcinum* Nordhoff et al. 2005

The description is as before (Nordhoff et al., 2005) with the following addition. The genomic G+C content of the type strain is 42.5%. Its approximate genome size is 2.51 Mbp.

#### Emended Description of *Treponema putidum* Wyss et al. 2004

The description is as before (Wyss et al., 2004) with the following addition. The genomic G+C content of the type strain is 37.3%. Its approximate genome size is 2.77 Mbp.

#### Emended Description of *Treponema socranskii* Smibert et al. 1984

The description is as before (Smibert et al., 1984) with the following modification. The genomic G+C content of the type strain is 49.4%. Its approximate genome size is 2.80 Mbp.

#### Emended Description of *Treponema succinifaciens* Cwyk and Canale-Parola 1981

The description is as before (Cwyk and Canale-Parola, 1979) with the following modification. The genomic G+C content of the type strain is 39.2%. Its approximate genome size is 2.73 Mbp.

#### Emended Description of *Tropicibacter naphthalenivorans* Harwati et al. 2009

The description is as before (Harwati et al., 2009b) with the following modification. The genomic G+C content of the type strain is 63.2%. Its approximate genome size is 4.45 Mbp.

#### Emended Description of *Tropicimonas isoalkanivorans* Harwati et al. 2009

The description is as before (Harwati et al., 2009a) with the following modification. The genomic G+C content of the type strain is 64.6%. Its approximate genome size is 4.98 Mbp.

#### Emended Description of *Tropicimonas sediminicola* Shin et al. 2012

The description is as before (Shin et al., 2012) with the following modification. The genomic G+C content of the type strain is 66.4%. Its approximate genome size is 5.17 Mbp.

#### Emended Description of *Turneriella parva* (Hovind-Hougen et al. 1982) Levett et al. 2005

The description is as before (Levett et al., 2005) with the following modification. The genomic G+C content of the type strain is 53.6%. Its approximate genome size is 4.41 Mbp.

#### Emended Description of *Wenxinia marina* Ying et al. 2007

The description is as before (Ying et al., 2007) with the following modification. The genomic G+C content of the type strain is 70.5%. Its approximate genome size is 4.18 Mbp.

#### Emended Description of *Wenxinia saemankumensis* Park et al. 2014

The description is as before (Park et al., 2014b) with the following modification. The genomic G+C content of the type strain is 71.2%. Its approximate genome size is 3.58 Mbp.

#### Emended Description of *Woodsholea maritima* Abraham et al. 2004

The description is as before (Abraham et al., 2004) with the following modification. The genomic G+C content of the type strain is 55.7%. Its approximate genome size is 3.10 Mbp.

#### Emended Description of *Xanthobacter autotrophicus* (Baumgarten et al. 1974) Wiegel et al. 1978 (Approved Lists 1980)

The description is as before (Wiegel et al., 1978) with the following addition. The genomic G+C content of the type strain is 67.5%. Its approximate genome size is 5.03 Mbp.

#### Emended Description of *Yoonia maritima* (Tanaka et al. 2014) Wirth and Whitman 2018

The description is as before (Wirth and Whitman, 2018) with the following addition. The G+C content of the type-strain genome is 53.4%, its approximate size 3.68 Mbp.

#### Emended Description of *Yoonia rosea* (Ivanova et al. 2005) Wirth and Whitman 2018

The description is as before (Wirth and Whitman, 2018) with the following restriction. The G+C content of the type-strain genome is 57.7%, its approximate size 3.51 Mbp.

#### Emended Description of *Yoonia tamlensis* (Lee 2012) Wirth and Whitman 2018

The description is as before (Wirth and Whitman, 2018) with the following modification. The G+C content of the type-strain genome is 56.9%, its approximate size 3.19 Mbp.

#### Emended Description of *Yoonia vestfoldensis* (Van Trappen et al. 2004) Wirth and Whitman 2018

The description is as before (Wirth and Whitman, 2018) with the following modification. The G+C content of the type-strain genome is 61.8%, its approximate size 3.72 Mbp.

#### Emended Description of *Zavarzinia compransoris* (ex Nozhevnikova and Zavarzin 1974) Meyer et al. 1994

The description is as before (Meyer et al., 1993) with the following modification. The G+C content of the type-strain genome is 68.1%, its approximate size 4.75 Mbp.

#### Emended Description of *Zymomonas mobilis* (Lindner 1928) De Ley and Swings 1976 (Approved Lists 1980)

The description is as before (De Ley and Swings, 1976) with the following addition. The genomic G+C content of the type strain is 46.1%. Its approximate genome size is 2.14 Mbp.

#### Emended Description of *Acetobacter pasteurianus* subsp. *ascendens* (Henneberg 1898) De Ley and Frateur 1974 (Approved Lists 1980)

The description is as before (De Ley and Frateur, 1974) with the following addition. The G+C content of the type-strain genome is 53.2%, its approximate size 3.00 Mbp.

#### Emended Description of *Acetobacter pasteurianus* subsp. *paradoxus* (Frateur 1950) De Ley and Frateur 1974 (Approved Lists 1980)

The description is as before (De Ley and Frateur, 1974) with the following addition. The G+C content of the type-strain genome is 53.3%, its approximate size 3.22 Mbp.

#### Emended Description of *Bartonella vinsonii* subsp. *arupensis* Welch et al. 2000

The description is as before (Welch et al., 1999) with the following addition. The G+C content of the type-strain genome is 38.6%, its approximate size 1.75 Mbp.

#### Emended Description of *Bartonella vinsonii* subsp. *berkhoffii* Kordick et al. 1996

The description is as before (Kordick et al., 1996) with the following addition. The G+C content of the type-strain genome is 38.9%, its approximate size 1.79 Mbp.

#### Emended Description of *Insolitispirillum peregrinum* subsp. *integrum* (Terasaki 1973) Yoon et al. 2007

The description is as before (Yoon et al., 2007b) with the following modification. The G+C content of the type-strain genome is 61.9%, its approximate size 4.64 Mbp.

#### Emended Description of *Roseomonas gilardii* subsp. *rosea* Han et al. 2003 emend. Sánchez-Porro et al. 2009

The description is as before (Sánchez-Porro et al., 2009) with the following addition. The G+C content of the type-strain genome is 70.7%, its approximate size 4.61 Mbp.

#### Emended Description of *Treponema socranskii* subsp. *paredis* Smibert et al. 1984

The description is as before (Smibert et al., 1984) with the following addition. The G+C content of the type-strain genome is 48.3%, its approximate size 2.73 Mbp.

#### Emended Description of *Zymomonas mobilis* subsp. *pomaceae* (Millis 1956) De Ley and Swings 1976 (Approved Lists 1980) emend. Coton et al. 2006

The description is as before (Coton et al., 2006) with the following addition. The G+C content of the type-strain genome is 44.0%, its approximate size 2.06 Mbp.

The datasets generated for this study can be found in the INSDC databases, in the IMG database and in the TYGS database.

## Author Contributions

BT, SG, and MG organized strain selection, cultivation, DNA preparation, and quality control at DSMZ. TW and NK organized genome sequencing, assembly, annotation, and quality control at JGI. JM-K and MG phylogenetically and statistically analyzed the data. AH and JM-K prepared the figures. L-MW, MS, ML, and AH collected the phenotypic information. AH, ML, MG, and MS interpreted the phenotypic information. AH, ML, MG, and JM-K wrote the manuscript. All authors read and approved the final manuscript.

## Conflict of Interest

The authors declare that the research was conducted in the absence of any commercial or financial relationships that could be construed as a potential conflict of interest.
