# Supplementary material for: Analysis of 1,000+ Type-Strain Genomes Substantially Improves Taxonomic Classification of Alphaproteobacteria
Source: Front Microbiol. 2020 Apr 7;11:468. doi: 10.3389/fmicb.2020.00468 (PMC7179689; doi:10.3389/fmicb.2020.00468)
Supplement: Supplementary file 2 [file Data_Sheet_1.PDF]

Analysis of 1,000 Type-Strain Genomes Improves Taxonomic Classification of Alphaproteobacteria

Supplementary File S2

List of Figures

1 Complete phylogenomic GBDP tree of the *Alphaproteobacteria* genome dataset 16
2 Backbone-constrained 16S rRNA gene ML tree (CCT) of *Alphaproteobacteria* 39
3 Unconstrained 16S rRNA gene ML tree (UCT) of *Alphaproteobacteria* . . 60
4 Supermatrix-based *Labrenzia* ML tree 61
5 Supermatrix-based *Sphingomonadales* ML tree 62

Abbreviations

CCT constrained, comprehensive tree inferred with ML and MP using the bipartitions of the GBDP tree with ≥95% support as backbone constraint

GBDP Genome BLAST Distance Phylogeny

ML Maximum Likelihood

UCT unconstrained, comprehensive 16S rRNA gene tree

Type species
- no
- yes

Phylum
- *Proteobacteria*
- *Spirochaetes*-Phylum

Class
- *Alphaproteobacteria*
- *Mariprofundia*
- *Spirochaetes*

Order
- *Brachyspirales*
- *Brevinematales*
- *Caulobacterales*
- *Emcibacterales*
- *Kiloniellales*
- *Kordiimonadales*
- *Leptospirales*
- *Magnetococcales*
- *Mariprofundales*
- *Parvularculales*
- *Rhizobiales*
- *Rhodobacterales*
- *Rhodospirillales*
- *Rhodothalassiales*
- *Rickettsiales*
- *Sneathiellales*
- *Sphingomonadales*
- *Spirochaetales*
- NA

Family
- *Acetobacteraceae*
- *Anaplasmataceae*
- *Aurantimonadaceae*
- *Bartonellaceae*
- *Beijerinckiaceae*
- *Borreliaceae*
- *Brachyspiraceae*
- *Bradyrhizobiaceae*
- *Brevinemataceae*
- *Brucellaceae*
- *Caulobacteraceae*
- *Chelatococcaceae*
- *Cohaesibacteraceae*
- *Emcibacteraceae*
- *Erythrobacteraceae*
- *Geminicoccaceae*
- *Hyphomicrobiaceae*
- *Hyphomonadaceae*
- *Kiloniellaceae*
- *Kordiimonadaceae*
- *Leptospiraceae*
- *Magnetococcaceae*
- *Mariprofundaceae*
- *Methylobacteriaceae*
- *Methylocystaceae*
- *Notoacmeibacteraceae*
- *Parvularculaceae*
- *Phyllobacteriaceae*
- *Rhizobiaceae*
- *Rhodobacteraceae*
- *Rhodobiaceae*
- *Rhodospirillaceae*
- *Rhodothalassiaceae*
- *Rickettsiaceae*
- *Roseiarcaceae*
- *Salinarimonadaceae*
- *Sneathiellaceae*
- *Sphingomonadaceae*
- *Spirochaetaceae*
- *Xanthobacteraceae*
- NA

Cell length
- Min. (0.5)
- Max. (125.0)

Cell width
- Min. (0.1)
- Max. (2.1)

Flagella
- no
- yes
- NA

Oxygen
- aerobic
- anaerobic
- facultatively aerobic
- facultatively anaerobic
- microaerophilic
- NA

Carotenoids
- no
- yes
- NA

Chlorophyll
- no
- yes
- NA

Ubiquinones
- Min. (8.0)
- Max. (11.0)

Sphingolipids
- no
- yes
- NA

Percent G+C
- Min. (26.9)
- Max. (73.4)

Sequence length (in bp)
- Min. (859,006)
- Max. (9,792,874)

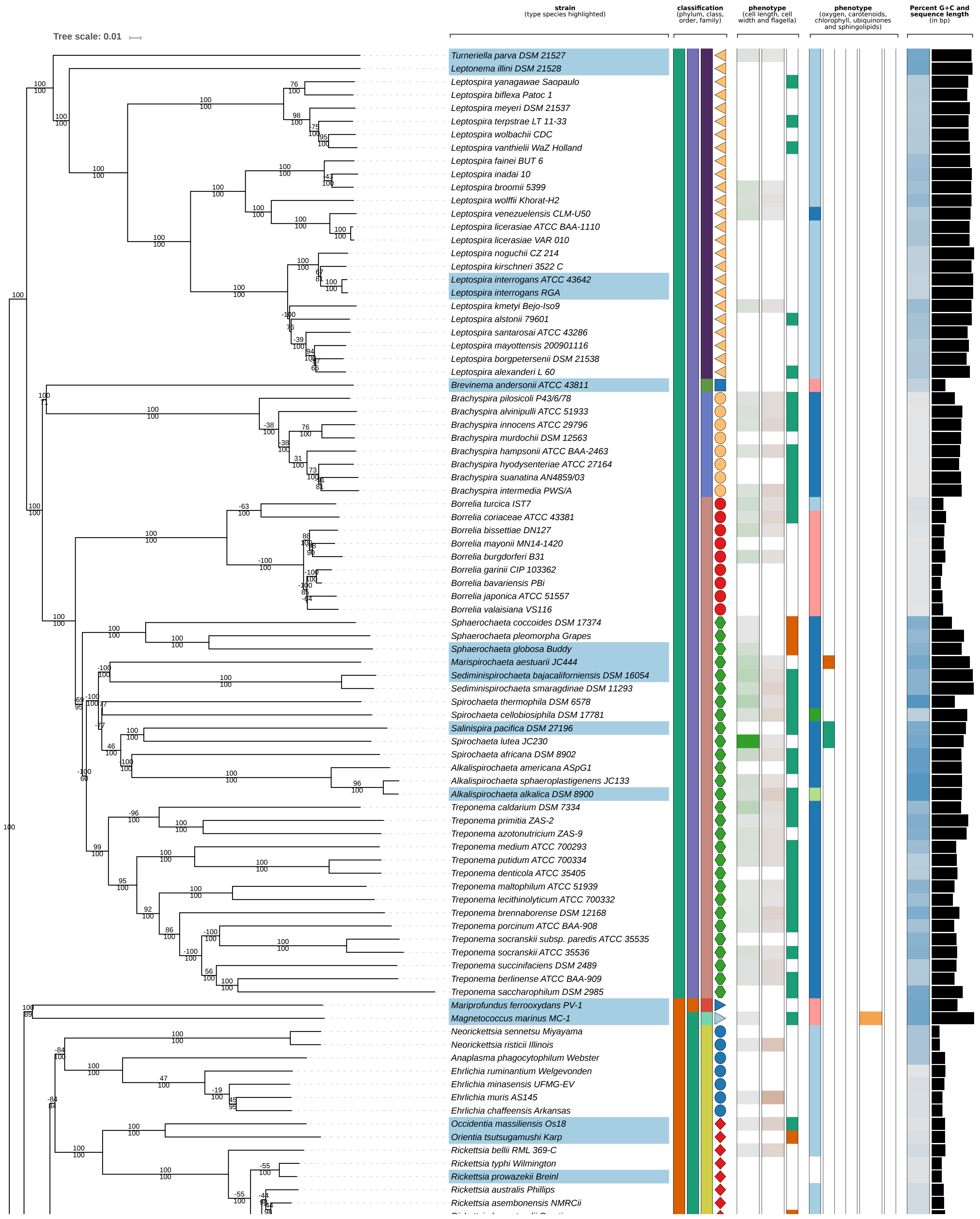
strain
(type species highlighted)
classification
(phylum, class, order, family)
phenotype
(cell length, cell width and flagella)
phenotype
(oxygen, carotenoids, chlorophyll, ubiquinones and sphingolipids)
Percent G+C and sequence length
(in bp)
Tree scale: 0.01
Turneriella parva DSM 21527
Leptonema illini DSM 21528
Leptospira yanagawae Saopaulo
Leptospira biflexa Patoc 1
Leptospira meyeri DSM 21537
Leptospira terpstrae LT 11-33
Leptospira wolbachii CDC
Leptospira vanthielii WaZ Holland
Leptospira fainei BUT 6
Leptospira inadai 10
Leptospira broomii 5399
Leptospira wolffii Khorat-H2
Leptospira venezuelensis CLM-U50
Leptospira licerasiae ATCC BAA-1110
Leptospira licerasiae VAR 010
Leptospira noguchii CZ 214
Leptospira kirschneri 3522 C
Leptospira interrogans ATCC 43642
Leptospira interrogans RGA
Leptospira kmetyi Bejo-Iso9
Leptospira alstonii 79601
Leptospira santarosai ATCC 43286
Leptospira mayottensis 200901116
Leptospira borgpetersenii DSM 21538
Leptospira alexanderi L 60
Brevinema andersonii ATCC 43811
Brachyspira pilosicoli P43/6/78
Brachyspira alvinipulli ATCC 51933
Brachyspira innocens ATCC 29796
Brachyspira murdochii DSM 12563
Brachyspira hampsonii ATCC BAA-2463
Brachyspira hyodysenteriae ATCC 27164
Brachyspira suanatina AN4859/03
Brachyspira intermedia PWS/A
Borrelia turcica IST7
Borrelia coriaceae ATCC 43381
Borrelia bissettiae DN127
Borrelia mayonii MN14-1420
Borrelia burgdorferi B31
Borrelia garinii CIP 103362
Borrelia bavariensis PBi
Borrelia japonica ATCC 51557
Borrelia valaisiana VS116
Sphaerochaeta coccoides DSM 17374
Sphaerochaeta pleomorpha Grapes
Sphaerochaeta globosa Buddy
Marispirochaeta aestuarii JC444
Sediminispirochaeta bajacaliforniensis DSM 16054
Sediminispirochaeta smaragdinae DSM 11293
Spirochaeta thermophila DSM 6578
Spirochaeta cellobiosiphila DSM 17781
Salinispira pacifica DSM 27196
Spirochaeta lutea JC230
Spirochaeta africana DSM 8902
Alkalispirochaeta americana ASpG1
Alkalispirochaeta sphaeroplastigenens JC133
Alkalispirochaeta alkalica DSM 8900
Treponema caldarium DSM 7334
Treponema primitia ZAS-2
Treponema azotonutricium ZAS-9
Treponema medium ATCC 700293
Treponema putidum ATCC 700334
Treponema denticola ATCC 35405
Treponema maltophilum ATCC 51939
Treponema lecithinolyticum ATCC 700332
Treponema brennaborense DSM 12168
Treponema porcinum ATCC BAA-908
Treponema socranskii subsp. paredis ATCC 35535
Treponema socranskii ATCC 35536
Treponema succinifaciens DSM 2489
Treponema berlinense ATCC BAA-909
Treponema saccharophilum DSM 2985
Mariprofundus ferrooxydans PV-1
Magnetococcus marinus MC-1
Neorickettsia sennetsu Miyayama
Neorickettsia risticii Illinois
Anaplasma phagocytophilum Webster
Ehrlichia ruminantium Welgevonden
Ehrlichia minasensis UFMG-EV
Ehrlichia muris AS145
Ehrlichia chaffeensis Arkansas
Occidentia massiliensis Os18
Orientia tsutsugamushi Karp
Rickettsia bellii RML 369-C
Rickettsia typhi Wilmington
Rickettsia prowazekii Breinl
Rickettsia australis Phillips
Rickettsia asembonensis NMRCii

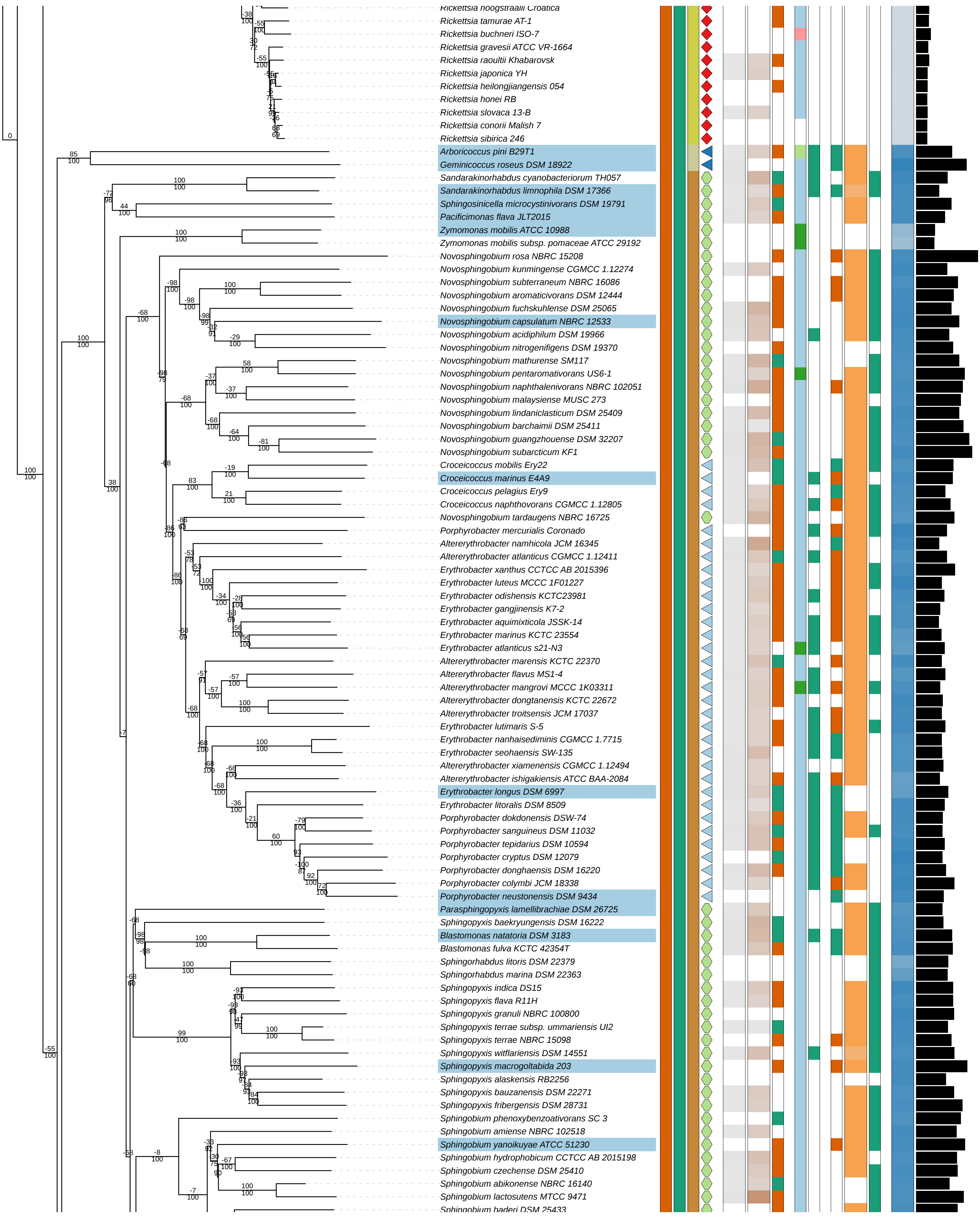

Rickettsia tamurae AT-1
Rickettsia buchneri ISO-7
Rickettsia gravesii ATCC VR-1664
Rickettsia raoultii Khabarovsk
Rickettsia japonica YH
Rickettsia heilongjiangensis 054
Rickettsia honei RB
Rickettsia slovaca 13-B
Rickettsia conorii Malish 7
Rickettsia sibirica 246
Arboricoccus pini B29T1
Geminicoccus roseus DSM 18922
Sandarakinorhabdus cyanobacteriorum TH057
Sandarakinorhabdus limnophila DSM 17366
Sphingosinicella microcystinivorans DSM 19791
Pacificimonas flava JLT2015
Zymomonas mobilis ATCC 10988
Zymomonas mobilis subsp. pomaceae ATCC 29192
Novosphingobium rosa NBRC 15208
Novosphingobium kunmingense CGMCC 1.12274
Novosphingobium subterraneum NBRC 16086
Novosphingobium aromaticivorans DSM 12444
Novosphingobium fuchskuhlense DSM 25065
Novosphingobium capsulatum NBRC 12533
Novosphingobium acidiphilum DSM 19966
Novosphingobium nitrogenifigens DSM 19370
Novosphingobium mathurense SM117
Novosphingobium pentaromativorans US6-1
Novosphingobium naphthalenivorans NBRC 102051
Novosphingobium malaysiense MUSC 273
Novosphingobium lindaniclasticum DSM 25409
Novosphingobium barchaimii DSM 25411
Novosphingobium guangzhouense DSM 32207
Novosphingobium subarcticum KF1
Croceicoccus mobilis Ery22
Croceicoccus marinus E4A9
Croceicoccus pelagius Ery9
Croceicoccus naphthovorans CGMCC 1.12805
Novosphingobium tardaugens NBRC 16725
Porphyrobacter mercurialis Coronado
Altererythrobacter namhicola JCM 16345
Altererythrobacter atlanticus CGMCC 1.12411
Erythrobacter xanthus CCTCC AB 2015396
Erythrobacter luteus MCCC 1F01227
Erythrobacter odishensis KCTC23981
Erythrobacter gangjinensis K7-2
Erythrobacter aquimixticola JSSK-14
Erythrobacter marinus KCTC 23554
Erythrobacter atlanticus s21-N3
Altererythrobacter marensis KCTC 22370
Altererythrobacter flavus MS1-4
Altererythrobacter mangrovi MCCC 1K03311
Altererythrobacter dongtanensis KCTC 22672
Altererythrobacter troitsensis JCM 17037
Erythrobacter lutimaris S-5
Erythrobacter nanhaisediminis CGMCC 1.7715
Erythrobacter seohaensis SW-135
Altererythrobacter xiamenensis CGMCC 1.12494
Altererythrobacter ishigakiensis ATCC BAA-2084
Erythrobacter longus DSM 6997
Erythrobacter litoralis DSM 8509
Porphyrobacter dokdonensis DSW-74
Porphyrobacter sanguineus DSM 11032
Porphyrobacter tepidarius DSM 10594
Porphyrobacter cryptus DSM 12079
Porphyrobacter donghaensis DSM 16220
Porphyrobacter colymbi JCM 18338
Porphyrobacter neustonensis DSM 9434
Parasphingopyxis lamellibrachiae DSM 26725
Sphingopyxis baekryungensis DSM 16222
Blastomonas natatoria DSM 3183
Blastomonas fulva KCTC 42354T
Sphingorhabdus litoris DSM 22379
Sphingorhabdus marina DSM 22363
Sphingopyxis indica DS15
Sphingopyxis flava R11H
Sphingopyxis granuli NBRC 100800
Sphingopyxis terrae subsp. ummariensis UI2
Sphingopyxis terrae NBRC 15098
Sphingopyxis witflariensis DSM 14551
Sphingopyxis macrogoltabida 203
Sphingopyxis alaskensis RB2256
Sphingopyxis bauzanensis DSM 22271
Sphingopyxis fribergensis DSM 28731
Sphingobium phenoxybenzoativorans SC 3
Sphingobium amiense NBRC 102518
Sphingobium yanoikuyae ATCC 51230
Sphingobium hydrophobicum CCTCC AB 2015198
Sphingobium czechense DSM 25410
Sphingobium abikonense NBRC 16140
Sphingobium lactosutens MTCC 9471
Sphingobium baderi DSM 25433

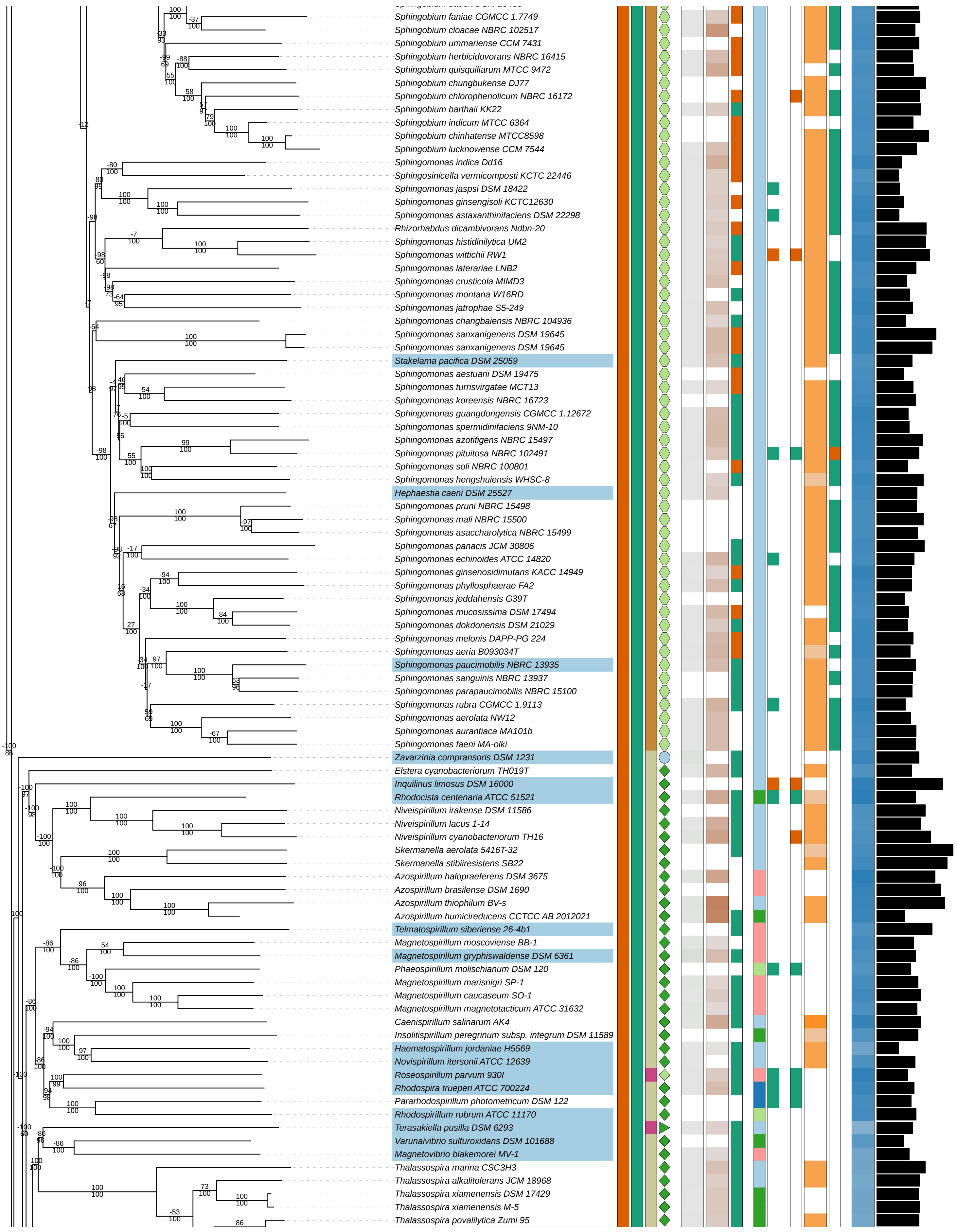

Sphingobium faniae CGMCC 1.7749
Sphingobium cloacae NBRC 102517
Sphingobium ummariense CCM 7431
Sphingobium herbicidovorans NBRC 16415
Sphingobium quisquiliarum MTCC 9472
Sphingobium chungbukense DJ77
Sphingobium chlorophenolicum NBRC 16172
Sphingobium barthaii KK22
Sphingobium indicum MTCC 6364
Sphingobium chinhatense MTCC8598
Sphingobium lucknowense CCM 7544
Sphingomonas indica Dd16
Sphingosinicella vermicomposti KCTC 22446
Sphingomonas jaspsi DSM 18422
Sphingomonas ginsengisoli KCTC12630
Sphingomonas astaxanthinifaciens DSM 22298
Rhizorhabdus dicambivorans Ndbn-20
Sphingomonas histidinilytica UM2
Sphingomonas wittichii RW1
Sphingomonas laterariae LNB2
Sphingomonas crusticola MIMD3
Sphingomonas montana W16RD
Sphingomonas jatrophae S5-249
Sphingomonas changbaiensis NBRC 104936
Sphingomonas sanxanigenens DSM 19645
Sphingomonas sanxanigenens DSM 19645
Stakelama pacifica DSM 25059
Sphingomonas aestuarii DSM 19475
Sphingomonas turrisvirgatae MCT13
Sphingomonas koreensis NBRC 16723
Sphingomonas guangdongensis CGMCC 1.12672
Sphingomonas spermidinifaciens 9NM-10
Sphingomonas azotifigens NBRC 15497
Sphingomonas pituitosa NBRC 102491
Sphingomonas soli NBRC 100801
Sphingomonas hengshuiensis WHSC-8
Hephaestia caeni DSM 25527
Sphingomonas pruni NBRC 15498
Sphingomonas mali NBRC 15500
Sphingomonas asaccharolytica NBRC 15499
Sphingomonas panacis JCM 30806
Sphingomonas echinoides ATCC 14820
Sphingomonas ginsenosidimutans KACC 14949
Sphingomonas phyllosphaerae FA2
Sphingomonas jeddahensis G39T
Sphingomonas mucosissima DSM 17494
Sphingomonas dokdonensis DSM 21029
Sphingomonas melonis DAPP-PG 224
Sphingomonas aeria B093034T
Sphingomonas paucimobilis NBRC 13935
Sphingomonas sanguinis NBRC 13937
Sphingomonas parapaucimobilis NBRC 15100
Sphingomonas rubra CGMCC 1.9113
Sphingomonas aerolata NW12
Sphingomonas aurantiaca MA101b
Sphingomonas faeni MA-olki
Zavarzinia compransoris DSM 1231
Elstera cyanobacteriorum TH019T
Inquilinus limosus DSM 16000
Rhodocista centenaria ATCC 51521
Niveispirillum irakense DSM 11586
Niveispirillum lacus 1-14
Niveispirillum cyanobacteriorum TH16
Skermanella aerolata 5416T-32
Skermanella stibiiresistens SB22
Azospirillum halopraeferens DSM 3675
Azospirillum brasilense DSM 1690
Azospirillum thiophilum BV-s
Azospirillum humicireducens CCTCC AB 2012021
Telmatospirillum siberiense 26-4b1
Magnetospirillum moscoviense BB-1
Magnetospirillum gryphiswaldense DSM 6361
Phaeospirillum molischianum DSM 120
Magnetospirillum marisnigri SP-1
Magnetospirillum caucaseum SO-1
Magnetospirillum magnetotacticum ATCC 31632
Caenispirillum salinarum AK4
Insolitispirillum peregrinum subsp. integrum DSM 11589
Haematospirillum jordaniae H5569
Novispirillum itersonii ATCC 12639
Roseospirillum parvum 930I
Rhodospira trueperi ATCC 700224
Pararhodospirillum photometricum DSM 122
Rhodospirillum rubrum ATCC 11170
Terasakiella pusilla DSM 6293
Varunaivibrio sulfuroxidans DSM 101688
Magnetovibrio blakemorei MV-1
Thalassospira marina CSC3H3
Thalassospira alkalitolerans JCM 18968
Thalassospira xiamenensis DSM 17429
Thalassospira xiamenensis M-5
Thalassospira povalilytica Zumi 95

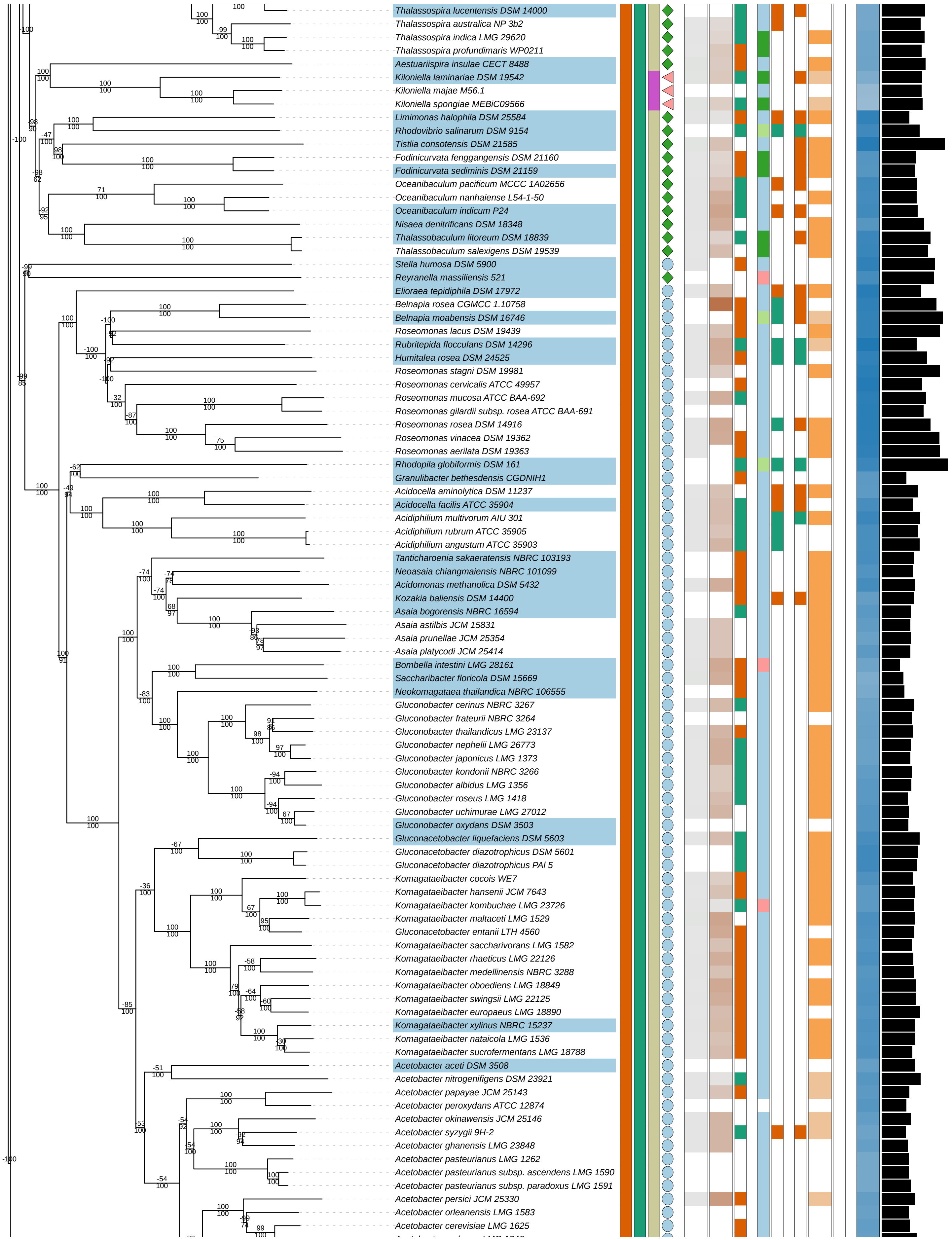
Thalassospira lucentensis DSM 14000
Thalassospira australica NP 3b2
Thalassospira indica LMG 29620
Thalassospira profundimaris WP0211
Aestuariispira insulae CECT 8488
Kiloniella laminariae DSM 19542
Kiloniella majae M56.1
Kiloniella spongiae MEBiC09566
Limimonas halophila DSM 25584
Rhodovibrio salinarum DSM 9154
Tistlia consotensis DSM 21585
Fodinicurvata fenggangensis DSM 21160
Fodinicurvata sediminis DSM 21159
Oceanibaculum pacificum MCCC 1A02656
Oceanibaculum nanhaiense L54-1-50
Oceanibaculum indicum P24
Nisaea denitrificans DSM 18348
Thalassobaculum litoreum DSM 18839
Thalassobaculum salexigens DSM 19539
Stella humosa DSM 5900
Reyranella massiliensis 521
Elioraea tepidiphila DSM 17972
Belnapia rosea CGMCC 1.10758
Belnapia moabensis DSM 16746
Roseomonas lacus DSM 19439
Rubritepida flocculans DSM 14296
Humitalea rosea DSM 24525
Roseomonas stagni DSM 19981
Roseomonas cervicalis ATCC 49957
Roseomonas mucosa ATCC BAA-692
Roseomonas gilardii subsp. rosea ATCC BAA-691
Roseomonas rosea DSM 14916
Roseomonas vinacea DSM 19362
Roseomonas aerilata DSM 19363
Rhodopila globiformis DSM 161
Granulibacter bethesdensis CGDNIH1
Acidocella aminolytica DSM 11237
Acidocella facilis ATCC 35904
Acidiphilium multivorum AIU 301
Acidiphilium rubrum ATCC 35905
Acidiphilium angustum ATCC 35903
Tanticharoenia sakaeratensis NBRC 103193
Neoasaia chiangmaiensis NBRC 101099
Acidomonas methanolica DSM 5432
Kozakia baliensis DSM 14400
Asaia bogorensis NBRC 16594
Asaia astilbis JCM 15831
Asaia prunellae JCM 25354
Asaia platycodi JCM 25414
Bombella intestini LMG 28161
Saccharibacter floricola DSM 15669
Neokomagataea thailandica NBRC 106555
Gluconobacter cerinus NBRC 3267
Gluconobacter frateurii NBRC 3264
Gluconobacter thailandicus LMG 23137
Gluconobacter nephelii LMG 26773
Gluconobacter japonicus LMG 1373
Gluconobacter kondonii NBRC 3266
Gluconobacter albidus LMG 1356
Gluconobacter roseus LMG 1418
Gluconobacter uchimurae LMG 27012
Gluconobacter oxydans DSM 3503
Gluconacetobacter liquefaciens DSM 5603
Gluconacetobacter diazotrophicus DSM 5601
Gluconacetobacter diazotrophicus PAl 5
Komagataeibacter cocois WE7
Komagataeibacter hansenii JCM 7643
Komagataeibacter kombuchae LMG 23726
Komagataeibacter maltaceti LMG 1529
Gluconacetobacter entanii LTH 4560
Komagataeibacter saccharivorans LMG 1582
Komagataeibacter rhaeticus LMG 22126
Komagataeibacter medellinensis NBRC 3288
Komagataeibacter oboediens LMG 18849
Komagataeibacter swingsii LMG 22125
Komagataeibacter europaeus LMG 18890
Komagataeibacter xylinus NBRC 15237
Komagataeibacter nataicola LMG 1536
Komagataeibacter sucrofermentans LMG 18788
Acetobacter aceti DSM 3508
Acetobacter nitrogenifigens DSM 23921
Acetobacter papayae JCM 25143
Acetobacter peroxydans ATCC 12874
Acetobacter okinawensis JCM 25146
Acetobacter syzygii 9H-2
Acetobacter ghanensis LMG 23848
Acetobacter pasteurianus LMG 1262
Acetobacter pasteurianus subsp. ascendens LMG 1590
Acetobacter pasteurianus subsp. paradoxus LMG 1591
Acetobacter persici JCM 25330
Acetobacter orleanensis LMG 1583
Acetobacter cerevisiae LMG 1625

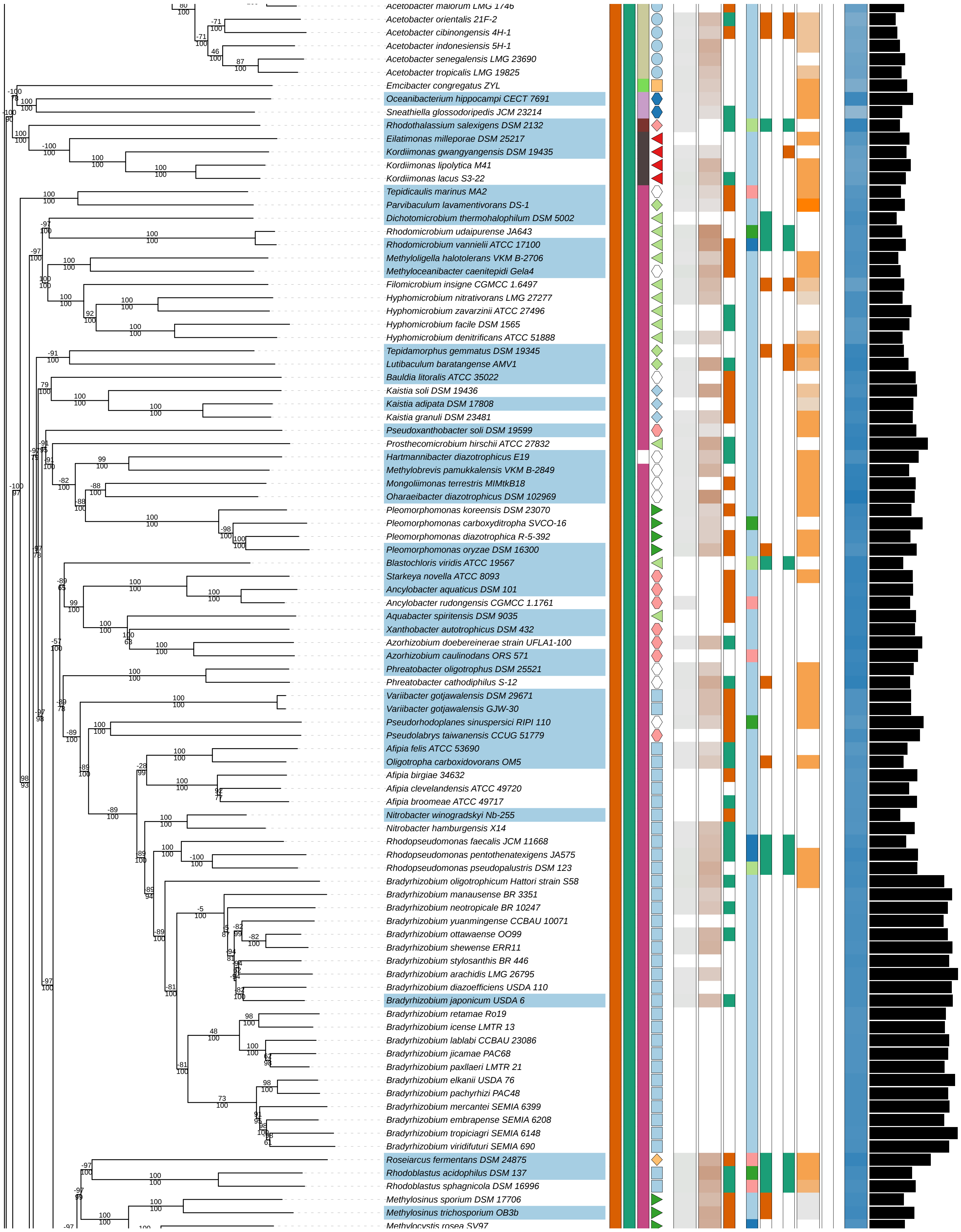
Acetobacter malorum LMG 1746
Acetobacter orientalis 21F-2
Acetobacter cibinongensis 4H-1
Acetobacter indonesiensis 5H-1
Acetobacter senegalensis LMG 23690
Acetobacter tropicalis LMG 19825
Emcibacter congregatus ZYL
Oceanibacterium hippocampi CECT 7691
Sneathiella glossodoripedis JCM 23214
Rhodothalassium salexigens DSM 2132
Eilatimonas milleporae DSM 25217
Kordiimonas gwangyangensis DSM 19435
Kordiimonas lipolytica M41
Kordiimonas lacus S3-22
Tepidicaulis marinus MA2
Parvibaculum lavamentivorans DS-1
Dichotomicrobium thermohalophilum DSM 5002
Rhodomicrobium udaipurense JA643
Rhodomicrobium vannielii ATCC 17100
Methyloligella halotolerans VKM B-2706
Methyloceanibacter caenitepidi Gela4
Filomicrobium insigne CGMCC 1.6497
Hyphomicrobium nitrativorans LMG 27277
Hyphomicrobium zavarzinii ATCC 27496
Hyphomicrobium facile DSM 1565
Hyphomicrobium denitrificans ATCC 51888
Tepidamorphus gemmatus DSM 19345
Lutibaculum baratangense AMV1
Bauldia litoralis ATCC 35022
Kaistia soli DSM 19436
Kaistia adipata DSM 17808
Kaistia granuli DSM 23481
Pseudoxanthobacter soli DSM 19599
Prosthecomicrobium hirschii ATCC 27832
Hartmannibacter diazotrophicus E19
Methylobrevis pamukkalensis VKM B-2849
Mongoliimonas terrestris MIMtkB18
Oharaeibacter diazotrophicus DSM 102969
Pleomorphomonas koreensis DSM 23070
Pleomorphomonas carboxyditropha SVCO-16
Pleomorphomonas diazotrophica R-5-392
Pleomorphomonas oryzae DSM 16300
Blastochloris viridis ATCC 19567
Starkeya novella ATCC 8093
Ancylobacter aquaticus DSM 101
Ancylobacter rudongensis CGMCC 1.1761
Aquabacter spiritensis DSM 9035
Xanthobacter autotrophicus DSM 432
Azorhizobium doebereinerae strain UFLA1-100
Azorhizobium caulinodans ORS 571
Phreatobacter oligotrophus DSM 25521
Phreatobacter cathodiphilus S-12
Variibacter gotjawalensis DSM 29671
Variibacter gotjawalensis GJW-30
Pseudorhodoplanes sinuspersici RIPI 110
Pseudolabrys taiwanensis CCUG 51779
Afipia felis ATCC 53690
Oligotropha carboxidovorans OM5
Afipia birgiae 34632
Afipia clevelandensis ATCC 49720
Afipia broomeae ATCC 49717
Nitrobacter winogradskyi Nb-255
Nitrobacter hamburgensis X14
Rhodopseudomonas faecalis JCM 11668
Rhodopseudomonas pentothenatexigens JA575
Rhodopseudomonas pseudopalustris DSM 123
Bradyrhizobium oligotrophicum Hattori strain S58
Bradyrhizobium manausense BR 3351
Bradyrhizobium neotropicale BR 10247
Bradyrhizobium yuanmingense CCBAU 10071
Bradyrhizobium ottawaense OO99
Bradyrhizobium shewense ERR11
Bradyrhizobium stylosanthis BR 446
Bradyrhizobium arachidis LMG 26795
Bradyrhizobium diazoefficiens USDA 110
Bradyrhizobium japonicum USDA 6
Bradyrhizobium retamae Ro19
Bradyrhizobium icense LMTR 13
Bradyrhizobium lablabi CCBAU 23086
Bradyrhizobium jicamae PAC68
Bradyrhizobium paxllaeri LMTR 21
Bradyrhizobium elkanii USDA 76
Bradyrhizobium pachyrhizi PAC48
Bradyrhizobium mercantei SEMIA 6399
Bradyrhizobium embrapense SEMIA 6208
Bradyrhizobium tropiciagri SEMIA 6148
Bradyrhizobium viridifuturi SEMIA 690
Roseiarcus fermentans DSM 24875
Rhodoblastus acidophilus DSM 137
Rhodoblastus sphagnicola DSM 16996
Methylosinus sporium DSM 17706
Methylosinus trichosporium OB3b
Methylocystis rosea SV97

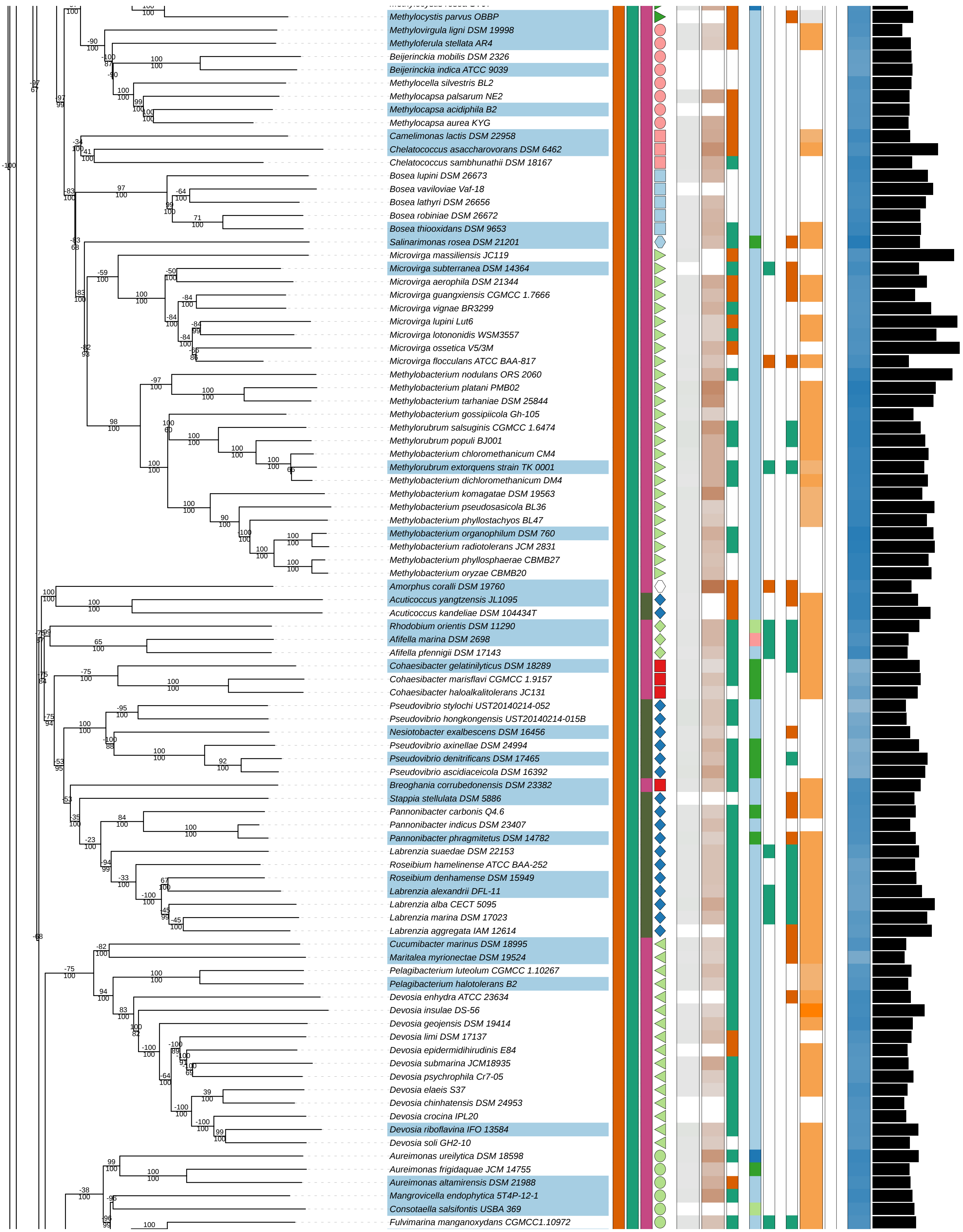
Methylocystis parvus OBBP
Methylovirgula ligni DSM 19998
Methyloferula stellata AR4
Beijerinckia mobilis DSM 2326
Beijerinckia indica ATCC 9039
Methylocella silvestris BL2
Methylocapsa palsarum NE2
Methylocapsa acidiphila B2
Methylocapsa aurea KYG
Camelimonas lactis DSM 22958
Chelatococcus asaccharovorans DSM 6462
Chelatococcus sambhunathii DSM 18167
Bosea lupini DSM 26673
Bosea vaviloviae Vaf-18
Bosea lathyri DSM 26656
Bosea robiniae DSM 26672
Bosea thiooxidans DSM 9653
Salinarimonas rosea DSM 21201
Microvirga massiliensis JC119
Microvirga subterranea DSM 14364
Microvirga aerophila DSM 21344
Microvirga guangxiensis CGMCC 1.7666
Microvirga vignae BR3299
Microvirga lupini Lut6
Microvirga lotononidis WSM3557
Microvirga ossetica V5/3M
Microvirga flocculans ATCC BAA-817
Methylobacterium nodulans ORS 2060
Methylobacterium platani PMB02
Methylobacterium tarhaniae DSM 25844
Methylobacterium gossipiicola Gh-105
Methylorubrum salsuginis CGMCC 1.6474
Methylorubrum populi BJ001
Methylobacterium chloromethanicum CM4
Methylorubrum extorquens strain TK 0001
Methylobacterium dichloromethanicum DM4
Methylobacterium komagatae DSM 19563
Methylobacterium pseudosasicola BL36
Methylobacterium phyllostachyos BL47
Methylobacterium organophilum DSM 760
Methylobacterium radiotolerans JCM 2831
Methylobacterium phyllosphaerae CBMB27
Methylobacterium oryzae CBMB20
Amorphus coralli DSM 19760
Acuticoccus yangtzensis JL1095
Acuticoccus kandeliae DSM 104434T
Rhodobium orientis DSM 11290
Afifella marina DSM 2698
Afifella pfennigii DSM 17143
Cohaesibacter gelatinilyticus DSM 18289
Cohaesibacter marisflavi CGMCC 1.9157
Cohaesibacter haloalkalitolerans JC131
Pseudovibrio stylochi UST20140214-052
Pseudovibrio hongkongensis UST20140214-015B
Nesiotobacter exalbescens DSM 16456
Pseudovibrio axinellae DSM 24994
Pseudovibrio denitrificans DSM 17465
Pseudovibrio ascidiaceicola DSM 16392
Breoghania corrubedonensis DSM 23382
Stappia stellulata DSM 5886
Pannonibacter carbonis Q4.6
Pannonibacter indicus DSM 23407
Pannonibacter phragmitetus DSM 14782
Labrenzia suaedae DSM 22153
Roseibium hamelinense ATCC BAA-252
Roseibium denhamense DSM 15949
Labrenzia alexandrii DFL-11
Labrenzia alba CECT 5095
Labrenzia marina DSM 17023
Labrenzia aggregata IAM 12614
Cucumibacter marinus DSM 18995
Maritalea myrionectae DSM 19524
Pelagibacterium luteolum CGMCC 1.10267
Pelagibacterium halotolerans B2
Devosia enhydra ATCC 23634
Devosia insulae DS-56
Devosia geojensis DSM 19414
Devosia limi DSM 17137
Devosia epidermidihirudinis E84
Devosia submarina JCM18935
Devosia psychrophila Cr7-05
Devosia elaeis S37
Devosia chinhatensis DSM 24953
Devosia crocina IPL20
Devosia riboflavina IFO 13584
Devosia soli GH2-10
Aureimonas ureilytica DSM 18598
Aureimonas frigidaquae JCM 14755
Aureimonas altamirensis DSM 21988
Mangrovicella endophytica 5T4P-12-1
Consotaella salsifontis USBA 369
Fulvimarina manganoxydans CGMCC1.10972

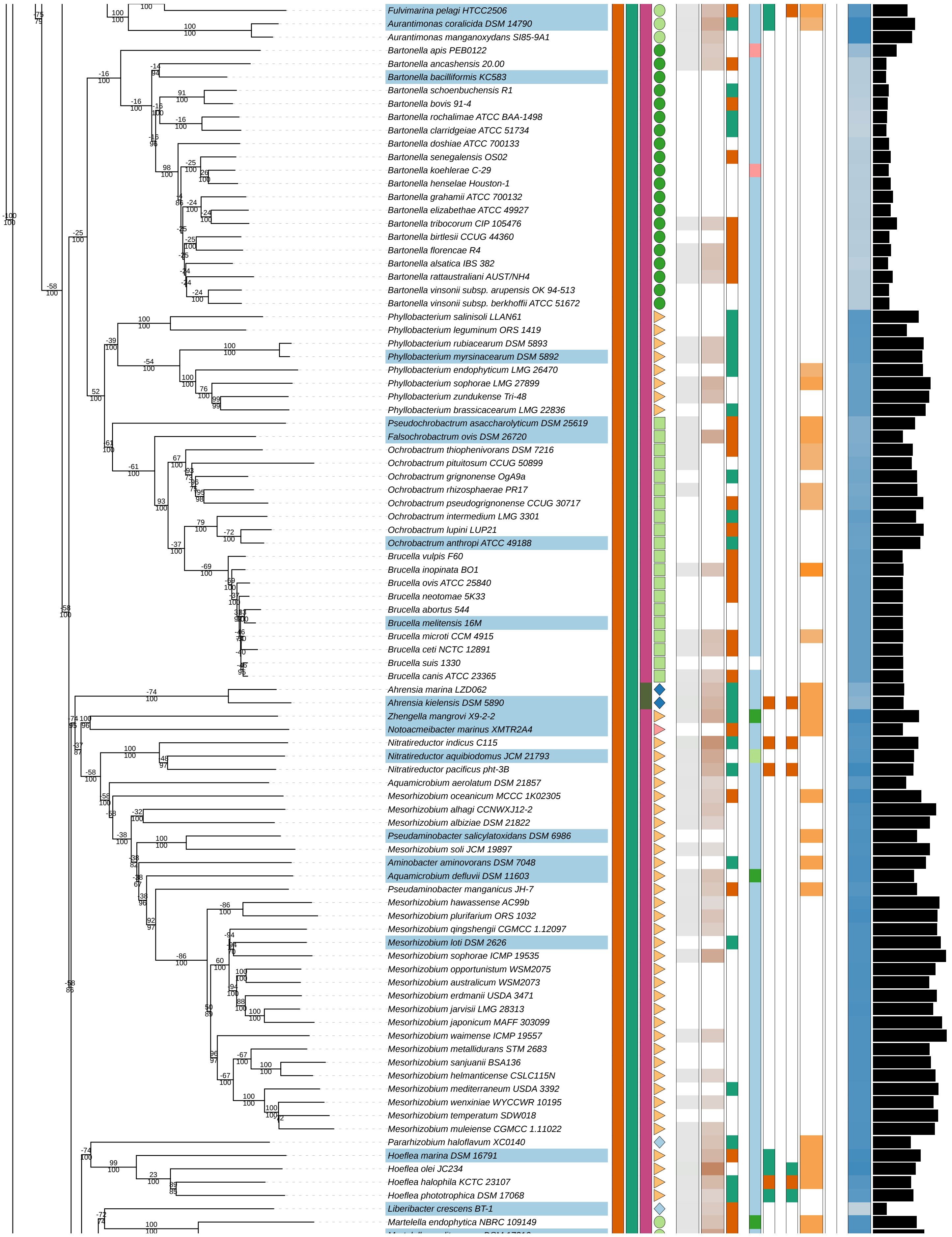
Fulvimarina pelagi HTCC2506
Aurantimonas coralicida DSM 14790
Aurantimonas manganoxydans SI85-9A1
Bartonella apis PEB0122
Bartonella ancashensis 20.00
Bartonella bacilliformis KC583
Bartonella schoenbuchensis R1
Bartonella bovis 91-4
Bartonella rochalimae ATCC BAA-1498
Bartonella clarridgeiae ATCC 51734
Bartonella doshiae ATCC 700133
Bartonella senegalensis OS02
Bartonella koehlerae C-29
Bartonella henselae Houston-1
Bartonella grahamii ATCC 700132
Bartonella elizabethae ATCC 49927
Bartonella tribocorum CIP 105476
Bartonella birtlesii CCUG 44360
Bartonella florencae R4
Bartonella alsatica IBS 382
Bartonella rattaustraliani AUST/NH4
Bartonella vinsonii subsp. arupensis OK 94-513
Bartonella vinsonii subsp. berkhoffii ATCC 51672
Phyllobacterium salinisoli LLAN61
Phyllobacterium leguminum ORS 1419
Phyllobacterium rubiacearum DSM 5893
Phyllobacterium myrsinacearum DSM 5892
Phyllobacterium endophyticum LMG 26470
Phyllobacterium sophorae LMG 27899
Phyllobacterium zundukense Tri-48
Phyllobacterium brassicacearum LMG 22836
Pseudochrobactrum asaccharolyticum DSM 25619
Falsochrobactrum ovis DSM 26720
Ochrobactrum thiophenivorans DSM 7216
Ochrobactrum pituitosum CCUG 50899
Ochrobactrum grignonense OgA9a
Ochrobactrum rhizosphaerae PR17
Ochrobactrum pseudogrignonense CCUG 30717
Ochrobactrum intermedium LMG 3301
Ochrobactrum lupini LUP21
Ochrobactrum anthropi ATCC 49188
Brucella vulpis F60
Brucella inopinata BO1
Brucella ovis ATCC 25840
Brucella neotomae 5K33
Brucella abortus 544
Brucella melitensis 16M
Brucella microti CCM 4915
Brucella ceti NCTC 12891
Brucella suis 1330
Brucella canis ATCC 23365
Ahrensia marina LZD062
Ahrensia kielensis DSM 5890
Zhengella mangrovi X9-2-2
Notoacmeibacter marinus XMTR2A4
Nitratireductor indicus C115
Nitratireductor aquibiodomus JCM 21793
Nitratireductor pacificus pht-3B
Aquamicrobium aerolatum DSM 21857
Mesorhizobium oceanicum MCCC 1K02305
Mesorhizobium alhagi CCNWXJ12-2
Mesorhizobium albiziae DSM 21822
Pseudaminobacter salicylatoxidans DSM 6986
Mesorhizobium soli JCM 19897
Aminobacter aminovorans DSM 7048
Aquamicrobium defluvii DSM 11603
Pseudaminobacter manganicus JH-7
Mesorhizobium hawassense AC99b
Mesorhizobium plurifarium ORS 1032
Mesorhizobium qingshengii CGMCC 1.12097
Mesorhizobium loti DSM 2626
Mesorhizobium sophorae ICMP 19535
Mesorhizobium opportunistum WSM2075
Mesorhizobium australicum WSM2073
Mesorhizobium erdmanii USDA 3471
Mesorhizobium jarvisii LMG 28313
Mesorhizobium japonicum MAFF 303099
Mesorhizobium waimense ICMP 19557
Mesorhizobium metallidurans STM 2683
Mesorhizobium sanjuanii BSA136
Mesorhizobium helmanticense CSLC115N
Mesorhizobium mediterraneum USDA 3392
Mesorhizobium wenxiniae WYCCWR 10195
Mesorhizobium temperatum SDW018
Mesorhizobium muleiense CGMCC 1.11022
Pararhizobium haloflavum XC0140
Hoeflea marina DSM 16791
Hoeflea olei JC234
Hoeflea halophila KCTC 23107
Hoeflea phototrophica DSM 17068
Liberibacter crescens BT-1
Martelella endophytica NBRC 109149

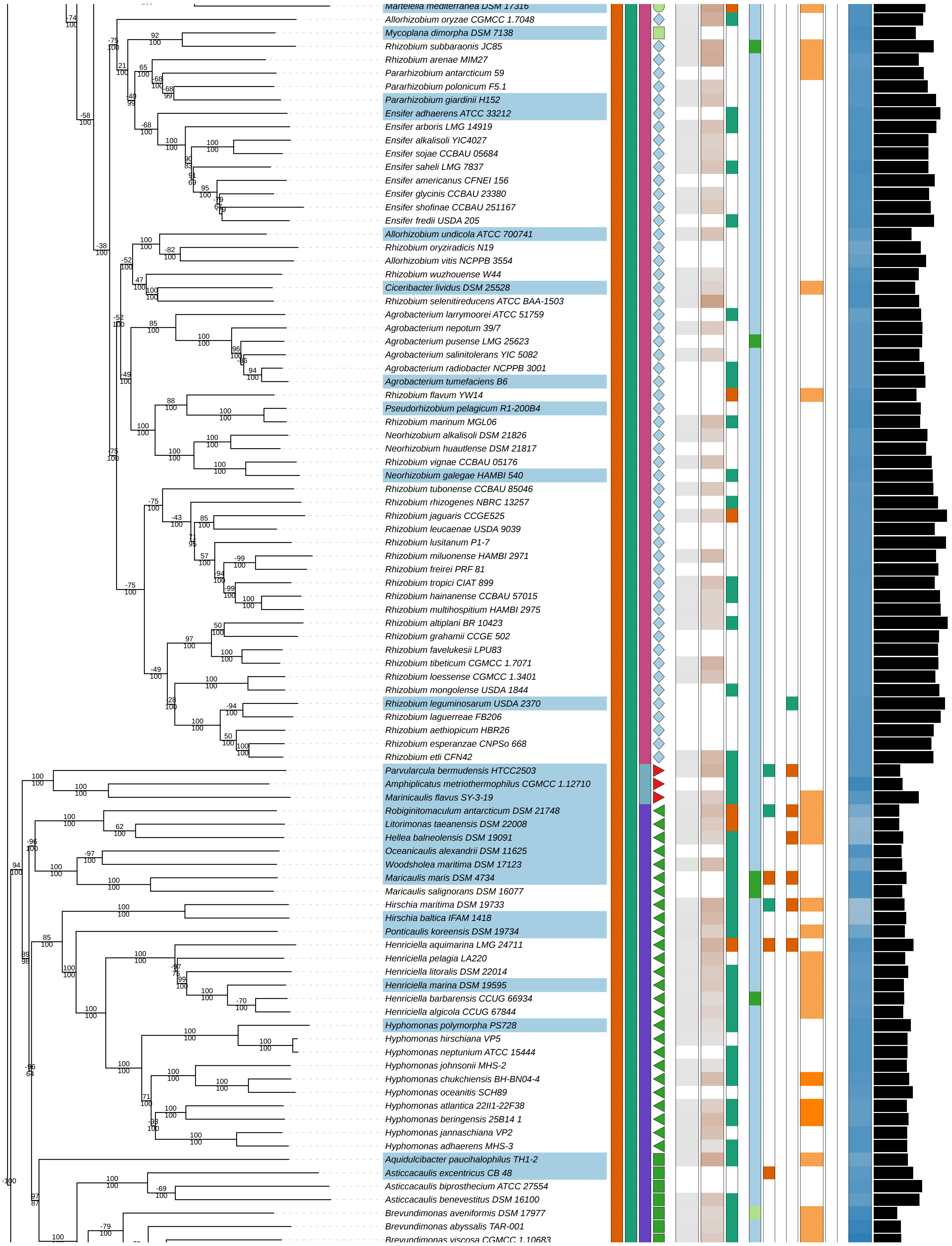
Martelella mediterranea DSM 17316
Allorhizobium oryzae CGMCC 1.7048
Mycoplana dimorpha DSM 7138
Rhizobium subbaraonis JC85
Rhizobium arenae MIM27
Pararhizobium antarcticum 59
Pararhizobium polonicum F5.1
Pararhizobium giardinii H152
Ensifer adhaerens ATCC 33212
Ensifer arboris LMG 14919
Ensifer alkalisoli YIC4027
Ensifer sojae CCBAU 05684
Ensifer saheli LMG 7837
Ensifer americanus CFNEI 156
Ensifer glycinis CCBAU 23380
Ensifer shofinae CCBAU 251167
Ensifer fredii USDA 205
Allorhizobium undicola ATCC 700741
Rhizobium oryziradicis N19
Allorhizobium vitis NCPPB 3554
Rhizobium wuzhouense W44
Ciceribacter lividus DSM 25528
Rhizobium selenitireducens ATCC BAA-1503
Agrobacterium larrymoorei ATCC 51759
Agrobacterium nepotum 39/7
Agrobacterium pusense LMG 25623
Agrobacterium salinitolerans YIC 5082
Agrobacterium radiobacter NCPPB 3001
Agrobacterium tumefaciens B6
Rhizobium flavum YW14
Pseudorhizobium pelagicum R1-200B4
Rhizobium marinum MGL06
Neorhizobium alkalisoli DSM 21826
Neorhizobium huautlense DSM 21817
Rhizobium vignae CCBAU 05176
Neorhizobium galegae HAMBI 540
Rhizobium tubonense CCBAU 85046
Rhizobium rhizogenes NBRC 13257
Rhizobium jaguaris CCGE525
Rhizobium leucaenae USDA 9039
Rhizobium lusitanum P1-7
Rhizobium miluonense HAMBI 2971
Rhizobium freirei PRF 81
Rhizobium tropici CIAT 899
Rhizobium hainanense CCBAU 57015
Rhizobium multihospitium HAMBI 2975
Rhizobium altiplani BR 10423
Rhizobium grahamii CCGE 502
Rhizobium favelukesii LPU83
Rhizobium tibeticum CGMCC 1.7071
Rhizobium loessense CGMCC 1.3401
Rhizobium mongolense USDA 1844
Rhizobium leguminosarum USDA 2370
Rhizobium laguerreae FB206
Rhizobium aethiopicum HBR26
Rhizobium esperanzae CNPSo 668
Rhizobium etli CFN42
Parvularcula bermudensis HTCC2503
Amphiplicatus metriothermophilus CGMCC 1.12710
Marinicaulis flavus SY-3-19
Robiginitomaculum antarcticum DSM 21748
Litorimonas taeanensis DSM 22008
Hellea balneolensis DSM 19091
Oceanicaulis alexandrii DSM 11625
Woodsholea maritima DSM 17123
Maricaulis maris DSM 4734
Maricaulis salignorans DSM 16077
Hirschia maritima DSM 19733
Hirschia baltica IFAM 1418
Ponticaulis koreensis DSM 19734
Henriciella aquimarina LMG 24711
Henriciella pelagia LA220
Henriciella litoralis DSM 22014
Henriciella marina DSM 19595
Henriciella barbarensis CCUG 66934
Henriciella algicola CCUG 67844
Hyphomonas polymorpha PS728
Hyphomonas hirschiana VP5
Hyphomonas neptunium ATCC 15444
Hyphomonas johnsonii MHS-2
Hyphomonas chukchiensis BH-BN04-4
Hyphomonas oceanitis SCH89
Hyphomonas atlantica 22II1-22F38
Hyphomonas beringensis 25B14 1
Hyphomonas jannaschiana VP2
Hyphomonas adhaerens MHS-3
Aquidulcibacter paucihalophilus TH1-2
Asticcacaulis excentricus CB 48
Asticcacaulis biprosthecium ATCC 27554
Asticcacaulis benevestitus DSM 16100
Brevundimonas aveniformis DSM 17977
Brevundimonas abyssalis TAR-001
Brevundimonas viscosa CGMCC 1.10683

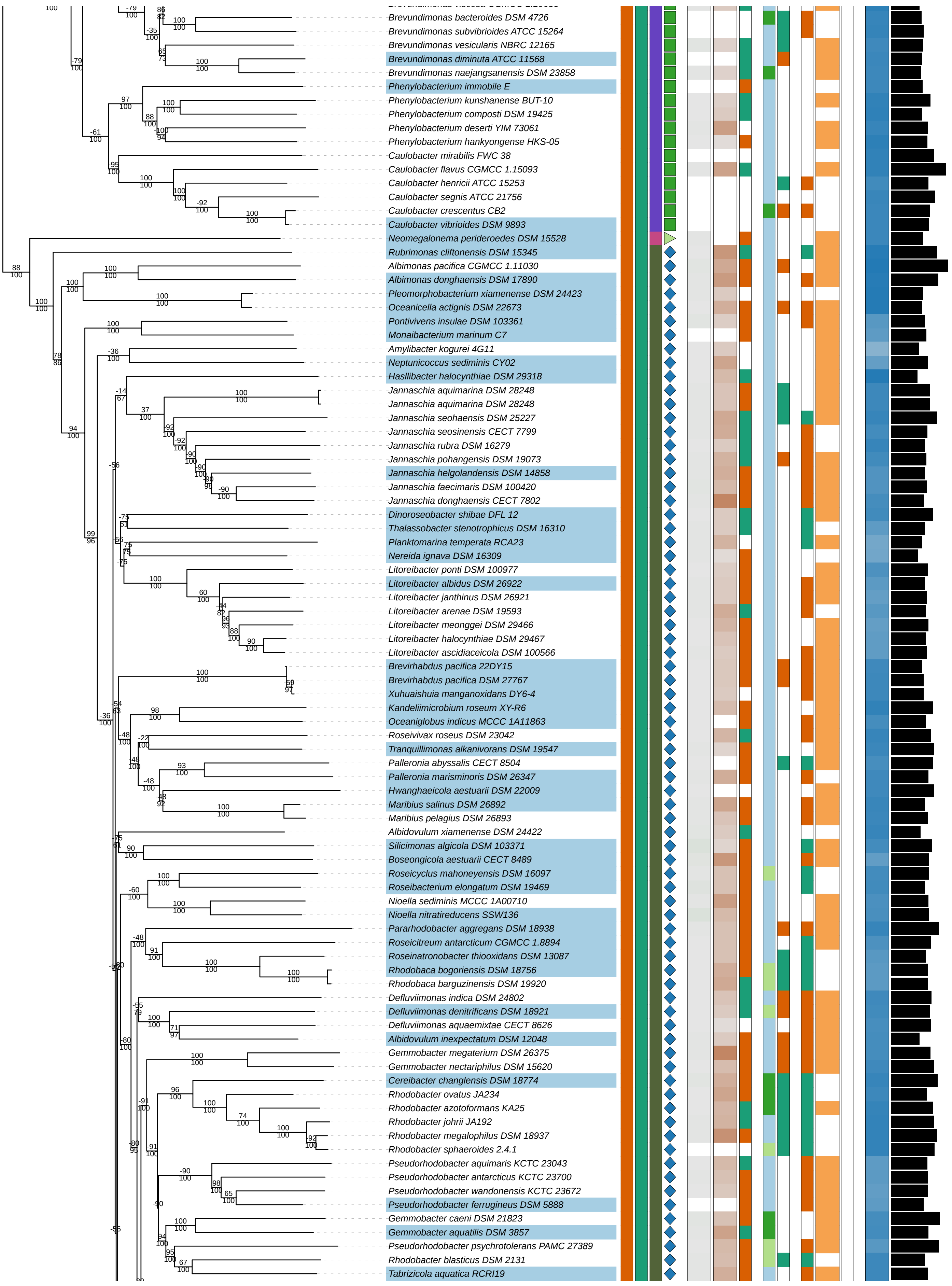

Brevundimonas bacteroides DSM 4726
Brevundimonas subvibrioides ATCC 15264
Brevundimonas vesicularis NBRC 12165
Brevundimonas diminuta ATCC 11568
Brevundimonas naejangsanensis DSM 23858
Phenylobacterium immobile E
Phenylobacterium kunshanense BUT-10
Phenylobacterium composti DSM 19425
Phenylobacterium deserti YIM 73061
Phenylobacterium hankyongense HKS-05
Caulobacter mirabilis FWC 38
Caulobacter flavus CGMCC 1.15093
Caulobacter henricii ATCC 15253
Caulobacter segnis ATCC 21756
Caulobacter crescentus CB2
Caulobacter vibrioides DSM 9893
Neomegalonema perideroedes DSM 15528
Rubrimonas cliftonensis DSM 15345
Albimonas pacifica CGMCC 1.11030
Albimonas donghaensis DSM 17890
Pleomorphobacterium xiamenense DSM 24423
Oceanicella actignis DSM 22673
Pontivivens insulae DSM 103361
Monaibacterium marinum C7
Amylibacter kogurei 4G11
Neptunicoccus sediminis CY02
Hasllibacter halocynthiae DSM 29318
Jannaschia aquimarina DSM 28248
Jannaschia aquimarina DSM 28248
Jannaschia seohaensis DSM 25227
Jannaschia seosinensis CECT 7799
Jannaschia rubra DSM 16279
Jannaschia pohangensis DSM 19073
Jannaschia helgolandensis DSM 14858
Jannaschia faecimaris DSM 100420
Jannaschia donghaensis CECT 7802
Dinoroseobacter shibae DFL 12
Thalassobacter stenotrophicus DSM 16310
Planktomarina temperata RCA23
Nereida ignava DSM 16309
Litoreibacter ponti DSM 100977
Litoreibacter albidus DSM 26922
Litoreibacter janthinus DSM 26921
Litoreibacter arenae DSM 19593
Litoreibacter meonggei DSM 29466
Litoreibacter halocynthiae DSM 29467
Litoreibacter ascidiaceicola DSM 100566
Brevirhabdus pacifica 22DY15
Brevirhabdus pacifica DSM 27767
Xuhuaishuia manganoxidans DY6-4
Kandeliimicrobium roseum XY-R6
Oceaniglobus indicus MCCC 1A11863
Roseivivax roseus DSM 23042
Tranquillimonas alkanivorans DSM 19547
Palleronia abyssalis CECT 8504
Palleronia marisminoris DSM 26347
Hwanghaeicola aestuarii DSM 22009
Maribius salinus DSM 26892
Maribius pelagius DSM 26893
Albidovulum xiamenense DSM 24422
Silicimonas algicola DSM 103371
Boseongicola aestuarii CECT 8489
Roseicyclus mahoneyensis DSM 16097
Roseibacterium elongatum DSM 19469
Nioella sediminis MCCC 1A00710
Nioella nitratireducens SSW136
Pararhodobacter aggregans DSM 18938
Roseicitreum antarcticum CGMCC 1.8894
Roseinatronobacter thiooxidans DSM 13087
Rhodobaca bogoriensis DSM 18756
Rhodobaca barguzinensis DSM 19920
Defluviimonas indica DSM 24802
Defluviimonas denitrificans DSM 18921
Defluviimonas aquaemixtae CECT 8626
Albidovulum inexpectatum DSM 12048
Gemmobacter megaterium DSM 26375
Gemmobacter nectariphilus DSM 15620
Cereibacter changlensis DSM 18774
Rhodobacter ovatus JA234
Rhodobacter azotoformans KA25
Rhodobacter johrii JA192
Rhodobacter megalophilus DSM 18937
Rhodobacter sphaeroides 2.4.1
Pseudorhodobacter aquimaris KCTC 23043
Pseudorhodobacter antarcticus KCTC 23700
Pseudorhodobacter wandonensis KCTC 23672
Pseudorhodobacter ferrugineus DSM 5888
Gemmobacter caeni DSM 21823
Gemmobacter aquatilis DSM 3857
Pseudorhodobacter psychrotolerans PAMC 27389
Rhodobacter blasticus DSM 2131
Tabrizicola aquatica RCRI19

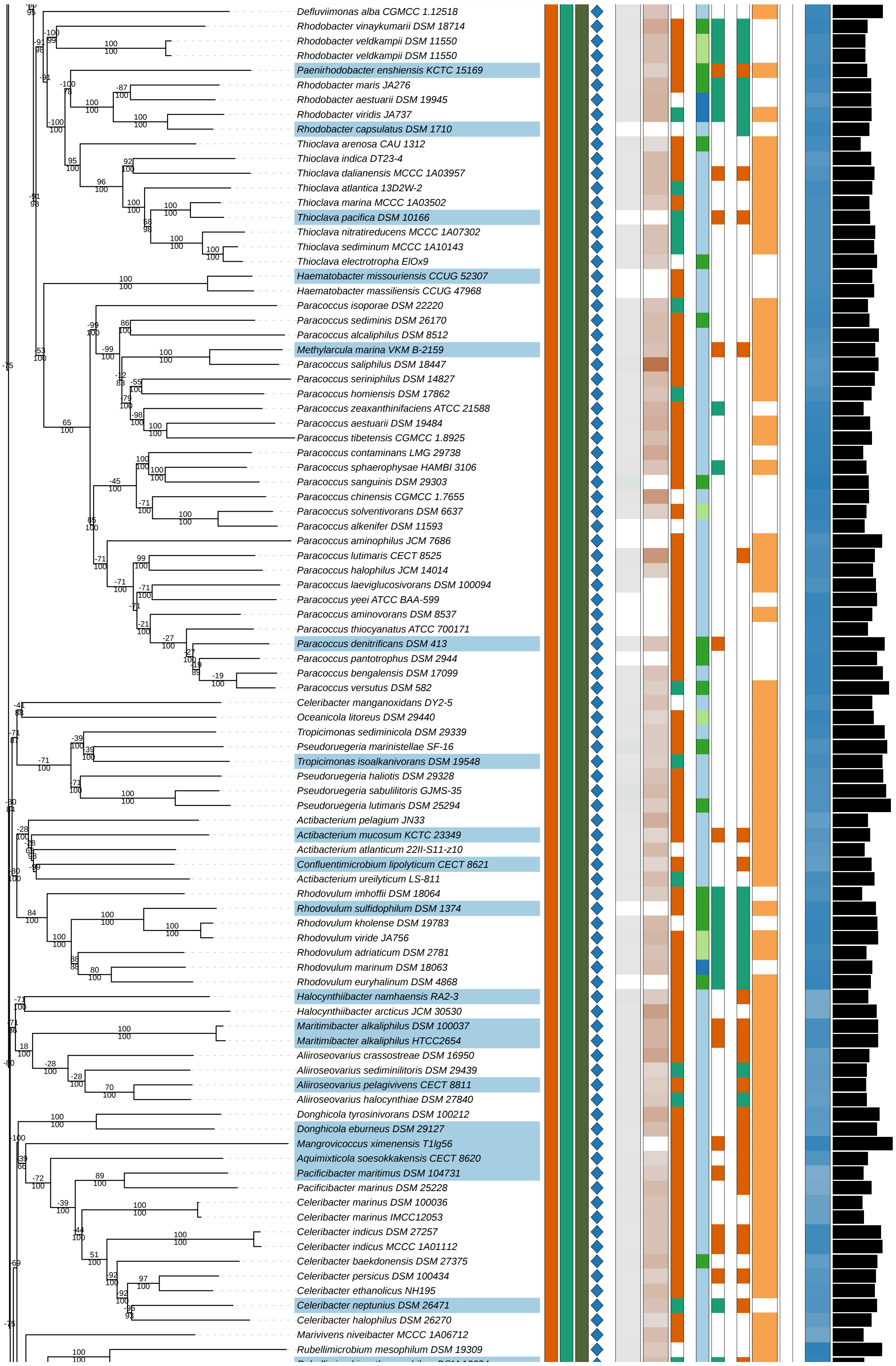
Defluviimonas alba CGMCC 1.12518
Rhodobacter vinaykumarii DSM 18714
Rhodobacter veldkampii DSM 11550
Rhodobacter veldkampii DSM 11550
Paenirhodobacter enshiensis KCTC 15169
Rhodobacter maris JA276
Rhodobacter aestuarii DSM 19945
Rhodobacter viridis JA737
Rhodobacter capsulatus DSM 1710
Thioclava arenosa CAU 1312
Thioclava indica DT23-4
Thioclava dalianensis MCCC 1A03957
Thioclava atlantica 13D2W-2
Thioclava marina MCCC 1A03502
Thioclava pacifica DSM 10166
Thioclava nitratireducens MCCC 1A07302
Thioclava sediminum MCCC 1A10143
Thioclava electrotropha ElOx9
Haematobacter missouriensis CCUG 52307
Haematobacter massiliensis CCUG 47968
Paracoccus isoporae DSM 22220
Paracoccus sediminis DSM 26170
Paracoccus alcaliphilus DSM 8512
Methylarcula marina VKM B-2159
Paracoccus saliphilus DSM 18447
Paracoccus seriniphilus DSM 14827
Paracoccus homiensis DSM 17862
Paracoccus zeaxanthinifaciens ATCC 21588
Paracoccus aestuarii DSM 19484
Paracoccus tibetensis CGMCC 1.8925
Paracoccus contaminans LMG 29738
Paracoccus sphaerophysae HAMBI 3106
Paracoccus sanguinis DSM 29303
Paracoccus chinensis CGMCC 1.7655
Paracoccus solventivorans DSM 6637
Paracoccus alkenifer DSM 11593
Paracoccus aminophilus JCM 7686
Paracoccus lutimaris CECT 8525
Paracoccus halophilus JCM 14014
Paracoccus laeviglucosivorans DSM 100094
Paracoccus yeei ATCC BAA-599
Paracoccus aminovorans DSM 8537
Paracoccus thiocyanatus ATCC 700171
Paracoccus denitrificans DSM 413
Paracoccus pantotrophus DSM 2944
Paracoccus bengalensis DSM 17099
Paracoccus versutus DSM 582
Celeribacter manganoxidans DY2-5
Oceanicola litoreus DSM 29440
Tropicimonas sediminicola DSM 29339
Pseudoruegeria marinistellae SF-16
Tropicimonas isoalkanivorans DSM 19548
Pseudoruegeria haliotis DSM 29328
Pseudoruegeria sabulilitoris GJMS-35
Pseudoruegeria lutimaris DSM 25294
Actibacterium pelagium JN33
Actibacterium mucosum KCTC 23349
Actibacterium atlanticum 22II-S11-z10
Confluentimicrobium lipolyticum CECT 8621
Actibacterium ureilyticum LS-811
Rhodovulum imhoffii DSM 18064
Rhodovulum sulfidophilum DSM 1374
Rhodovulum kholense DSM 19783
Rhodovulum viride JA756
Rhodovulum adriaticum DSM 2781
Rhodovulum marinum DSM 18063
Rhodovulum euryhalinum DSM 4868
Halocynthiibacter namhaensis RA2-3
Halocynthiibacter arcticus JCM 30530
Maritimibacter alkaliphilus DSM 100037
Maritimibacter alkaliphilus HTCC2654
Aliiroseovarius crassostreae DSM 16950
Aliiroseovarius sediminilitoris DSM 29439
Aliiroseovarius pelagivivens CECT 8811
Aliiroseovarius halocynthiae DSM 27840
Donghicola tyrosinivorans DSM 100212
Donghicola eburneus DSM 29127
Mangrovicoccus ximenensis T1lg56
Aquimixticola soesokkakensis CECT 8620
Pacificibacter maritimus DSM 104731
Pacificibacter marinus DSM 25228
Celeribacter marinus DSM 100036
Celeribacter marinus IMCC12053
Celeribacter indicus DSM 27257
Celeribacter indicus MCCC 1A01112
Celeribacter baekdonensis DSM 27375
Celeribacter persicus DSM 100434
Celeribacter ethanolicus NH195
Celeribacter neptunius DSM 26471
Celeribacter halophilus DSM 26270
Marivivens niveibacter MCCC 1A06712
Rubellimicrobium mesophilum DSM 19309

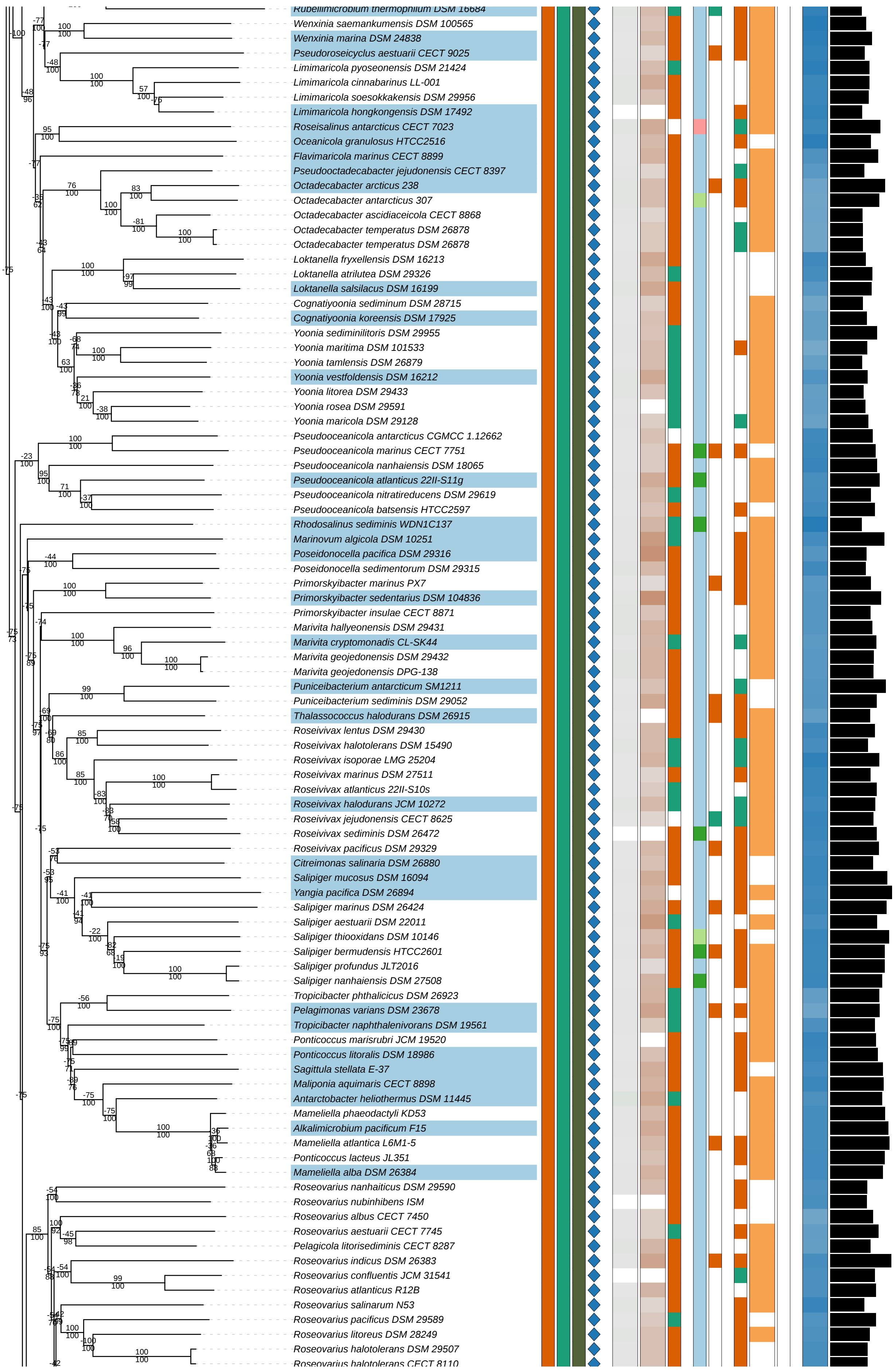
Rubellimicrobium thermophilum DSM 16684
Wenxinia saemankumensis DSM 100565
Wenxinia marina DSM 24838
Pseudoroseicyclus aestuarii CECT 9025
Limimaricola pyoseonensis DSM 21424
Limimaricola cinnabarinus LL-001
Limimaricola soesokkakensis DSM 29956
Limimaricola hongkongensis DSM 17492
Roseisalinus antarcticus CECT 7023
Oceanicola granulosus HTCC2516
Flavimaricola marinus CECT 8899
Pseudooctadecabacter jejudonensis CECT 8397
Octadecabacter arcticus 238
Octadecabacter antarcticus 307
Octadecabacter ascidiaceicola CECT 8868
Octadecabacter temperatus DSM 26878
Octadecabacter temperatus DSM 26878
Loktanella fryxellensis DSM 16213
Loktanella atrilutea DSM 29326
Loktanella salsilacus DSM 16199
Cognatiyoonia sediminum DSM 28715
Cognatiyoonia koreensis DSM 17925
Yoonia sediminilitoris DSM 29955
Yoonia maritima DSM 101533
Yoonia tamlensis DSM 26879
Yoonia vestfoldensis DSM 16212
Yoonia litorea DSM 29433
Yoonia rosea DSM 29591
Yoonia maricola DSM 29128
Pseudooceanicola antarcticus CGMCC 1.12662
Pseudooceanicola marinus CECT 7751
Pseudooceanicola nanhaiensis DSM 18065
Pseudooceanicola atlanticus 22II-S11g
Pseudooceanicola nitratireducens DSM 29619
Pseudooceanicola batsensis HTCC2597
Rhodosalinus sediminis WDN1C137
Marinovum algicola DSM 10251
Poseidonocella pacifica DSM 29316
Poseidonocella sedimentorum DSM 29315
Primorskyibacter marinus PX7
Primorskyibacter sedentarius DSM 104836
Primorskyibacter insulae CECT 8871
Marivita hallyeonensis DSM 29431
Marivita cryptomonadis CL-SK44
Marivita geojedonensis DSM 29432
Marivita geojedonensis DPG-138
Puniceibacterium antarcticum SM1211
Puniceibacterium sediminis DSM 29052
Thalassococcus halodurans DSM 26915
Roseivivax lentus DSM 29430
Roseivivax halotolerans DSM 15490
Roseivivax isoporae LMG 25204
Roseivivax marinus DSM 27511
Roseivivax atlanticus 22II-S10s
Roseivivax halodurans JCM 10272
Roseivivax jejudonensis CECT 8625
Roseivivax sediminis DSM 26472
Roseivivax pacificus DSM 29329
Citreimonas salinaria DSM 26880
Salipiger mucosus DSM 16094
Yangia pacifica DSM 26894
Salipiger marinus DSM 26424
Salipiger aestuarii DSM 22011
Salipiger thiooxidans DSM 10146
Salipiger bermudensis HTCC2601
Salipiger profundus JLT2016
Salipiger nanhaiensis DSM 27508
Tropicibacter phthalicicus DSM 26923
Pelagimonas varians DSM 23678
Tropicibacter naphthalenivorans DSM 19561
Ponticoccus marisrubri JCM 19520
Ponticoccus litoralis DSM 18986
Sagittula stellata E-37
Maliponia aquimaris CECT 8898
Antarctobacter heliothermus DSM 11445
Mameliella phaeodactyli KD53
Alkalimicrobium pacificum F15
Mameliella atlantica L6M1-5
Ponticoccus lacteus JL351
Mameliella alba DSM 26384
Roseovarius nanhaiticus DSM 29590
Roseovarius nubinhibens ISM
Roseovarius albus CECT 7450
Roseovarius aestuarii CECT 7745
Pelagicola litorisediminis CECT 8287
Roseovarius indicus DSM 26383
Roseovarius confluentis JCM 31541
Roseovarius atlanticus R12B
Roseovarius salinarum N53
Roseovarius pacificus DSM 29589
Roseovarius litoreus DSM 28249
Roseovarius halotolerans DSM 29507
Roseovarius halotolerans CECT 8110

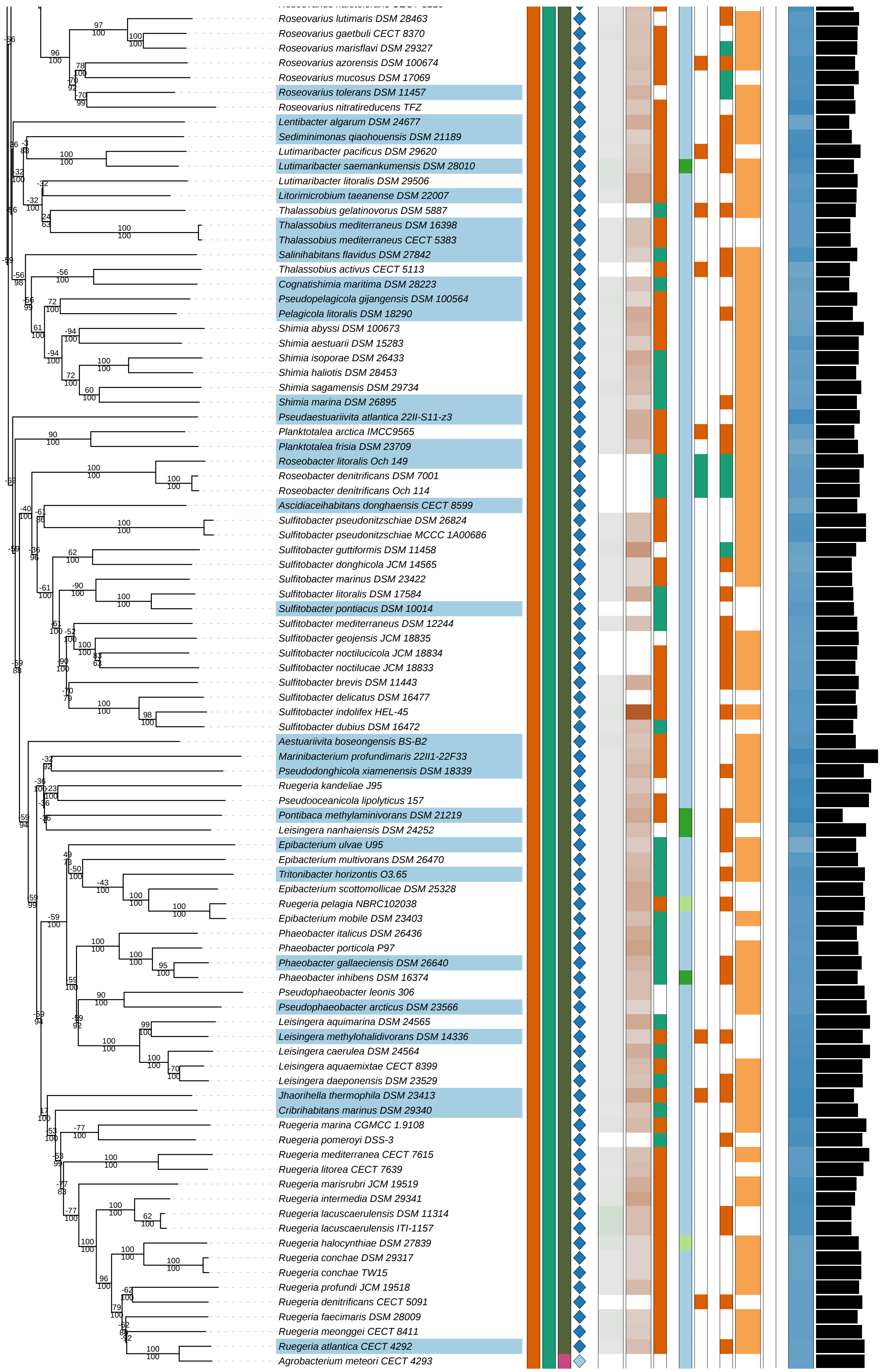
Roseovarius lutimaris DSM 28463
Roseovarius gaetbuli CECT 8370
Roseovarius marisflavi DSM 29327
Roseovarius azorensis DSM 100674
Roseovarius mucosus DSM 17069
Roseovarius tolerans DSM 11457
Roseovarius nitratireducens TFZ
Lentibacter algarum DSM 24677
Sediminimonas qiaohouensis DSM 21189
Lutimaribacter pacificus DSM 29620
Lutimaribacter saemankumensis DSM 28010
Lutimaribacter litoralis DSM 29506
Litorimicrobium taeanense DSM 22007
Thalassobius gelatinovorus DSM 5887
Thalassobius mediterraneus DSM 16398
Thalassobius mediterraneus CECT 5383
Salinihabitans flavidus DSM 27842
Thalassobius activus CECT 5113
Cognatishimia maritima DSM 28223
Pseudopelagicola gijangensis DSM 100564
Pelagicola litoralis DSM 18290
Shimia abyssi DSM 100673
Shimia aestuarii DSM 15283
Shimia isoporae DSM 26433
Shimia haliotis DSM 28453
Shimia sagamensis DSM 29734
Shimia marina DSM 26895
Pseudaestuariivita atlantica 22II-S11-z3
Planktotalea arctica IMCC9565
Planktotalea frisia DSM 23709
Roseobacter litoralis Och 149
Roseobacter denitrificans DSM 7001
Roseobacter denitrificans Och 114
Ascidiaceihabitans donghaensis CECT 8599
Sulfitobacter pseudonitzschiae DSM 26824
Sulfitobacter pseudonitzschiae MCCC 1A00686
Sulfitobacter guttiformis DSM 11458
Sulfitobacter donghicola JCM 14565
Sulfitobacter marinus DSM 23422
Sulfitobacter litoralis DSM 17584
Sulfitobacter pontiacus DSM 10014
Sulfitobacter mediterraneus DSM 12244
Sulfitobacter geojensis JCM 18835
Sulfitobacter noctilucicola JCM 18834
Sulfitobacter noctilucae JCM 18833
Sulfitobacter brevis DSM 11443
Sulfitobacter delicatus DSM 16477
Sulfitobacter indolifex HEL-45
Sulfitobacter dubius DSM 16472
Aestuariivita boseongensis BS-B2
Marinibacterium profundimaris 22II1-22F33
Pseudodonghicola xiamenensis DSM 18339
Ruegeria kandeliae J95
Pseudooceanicola lipolyticus 157
Pontibaca methylaminivorans DSM 21219
Leisingera nanhaiensis DSM 24252
Epibacterium ulvae U95
Epibacterium multivorans DSM 26470
Tritonibacter horizontis O3.65
Epibacterium scottomollicae DSM 25328
Ruegeria pelagia NBRC102038
Epibacterium mobile DSM 23403
Phaeobacter italicus DSM 26436
Phaeobacter porticola P97
Phaeobacter gallaeciensis DSM 26640
Phaeobacter inhibens DSM 16374
Pseudophaeobacter leonis 306
Pseudophaeobacter arcticus DSM 23566
Leisingera aquimarina DSM 24565
Leisingera methylohalidivorans DSM 14336
Leisingera caerulea DSM 24564
Leisingera aquaemixtae CECT 8399
Leisingera daeponensis DSM 23529
Jhaorihella thermophila DSM 23413
Cribrihabitans marinus DSM 29340
Ruegeria marina CGMCC 1.9108
Ruegeria pomeroyi DSS-3
Ruegeria mediterranea CECT 7615
Ruegeria litorea CECT 7639
Ruegeria marisrubri JCM 19519
Ruegeria intermedia DSM 29341
Ruegeria lacuscaerulensis DSM 11314
Ruegeria lacuscaerulensis ITI-1157
Ruegeria halocynthiae DSM 27839
Ruegeria conchae DSM 29317
Ruegeria conchae TW15
Ruegeria profundi JCM 19518
Ruegeria denitrificans CECT 5091
Ruegeria faecimaris DSM 28009
Ruegeria meonggei CECT 8411
Ruegeria atlantica CECT 4292
Agrobacterium meteori CECT 4293

Figure 1: The figure shows the complete, uncollapsed GBDP tree of the *Alphaproteobacteria* genome dataset, which had to be distributed across Figures 1-9 in the main manuscript. Tree inferred with FastME from GBDP distances calculated from whole proteomes. The branches are scaled in terms of log-transformed intergenomic distances (GBDP formula d_5). The numbers below branches are GBDP pseudo-bootstrap support values from 100 replications. The numbers above branches are support (positive) or conflict (negative) values from the gene-content analysis. Tip colors indicate type species, colors to the right of the tips indicate, from left to right, phylum (1), class (2), order (3) and family (4). The blocks labelled as "phenotype" display phenotypic information (5-13), whereas the the blue gradient scale (14) indicates the exact G+C content as calculated from the genome sequences. Genome size (15) is displayed at the right-hand side. See the embedded legend for details. *Jannaschia aquimarina* DSM 28248 is represented by two distinct GenBank biosample accessions (above: SAMN05421775, below: SAMN03329626). The same holds true for *Octadecabacter temperatus* DSM 26878 (above: SAMN03891579, below: SAMN05444287), *Rhodobacter veldkampii* DSM 11550 (above: SAMN10866319, below: SAMN08535030) and *Sphingomonas sanxanigenens* DSM 19645 (above: SAMN02641489, below: SAMN02745820).

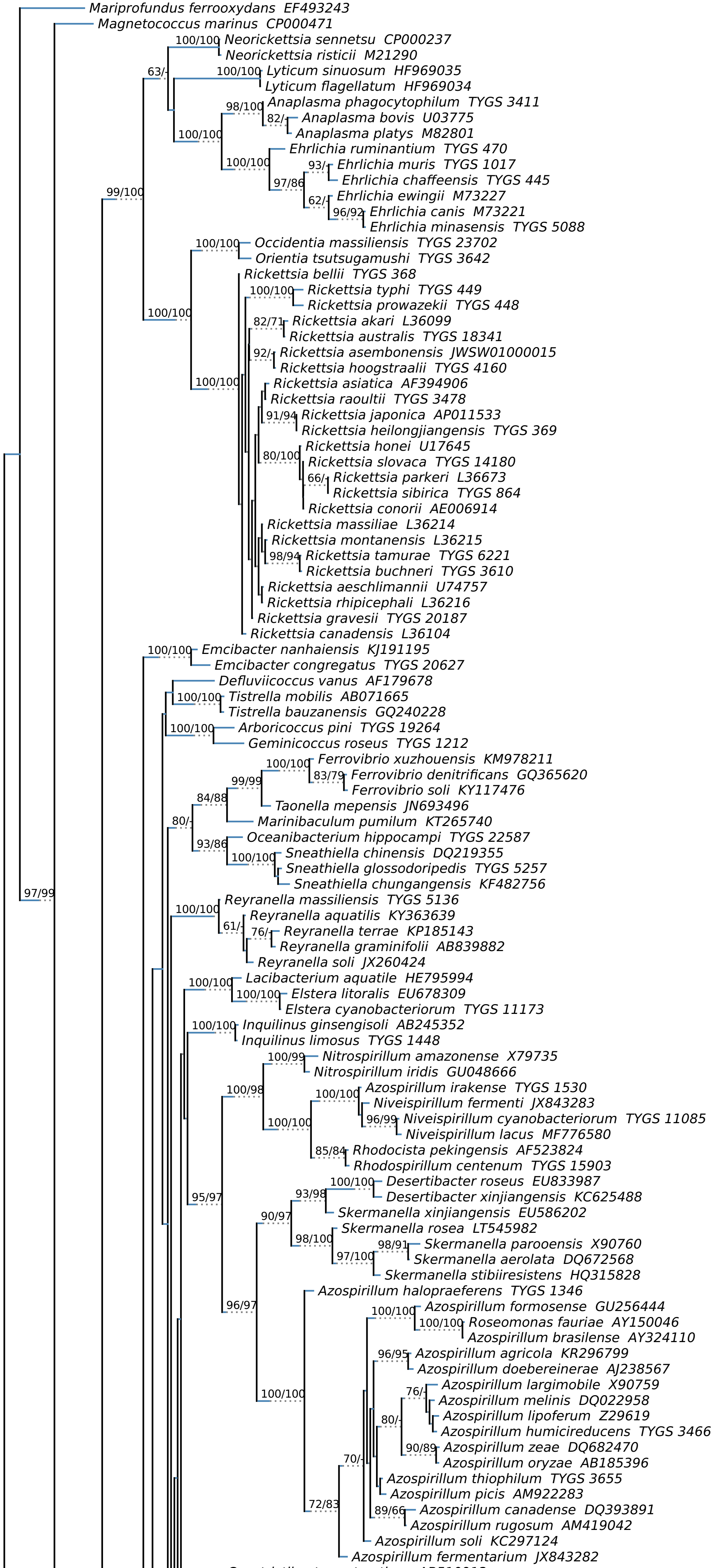
Mariprofundus ferrooxydans EF493243
Magnetococcus marinus CP000471
Neorickettsia sennetsu CP000237
Neorickettsia risticii M21290
Lyticum sinuosum HF969035
Lyticum flagellatum HF969034
Anaplasma phagocytophilum TYGS 3411
Anaplasma bovis U03775
Anaplasma platys M82801
Ehrlichia ruminantium TYGS 470
Ehrlichia muris TYGS 1017
Ehrlichia chaffeensis TYGS 445
Ehrlichia ewingii M73227
Ehrlichia canis M73221
Ehrlichia minasensis TYGS 5088
Occidentia massiliensis TYGS 23702
Orientia tsutsugamushi TYGS 3642
Rickettsia bellii TYGS 368
Rickettsia typhi TYGS 449
Rickettsia prowazekii TYGS 448
Rickettsia akari L36099
Rickettsia australis TYGS 18341
Rickettsia asembonensis JWSW01000015
Rickettsia hoogstraalii TYGS 4160
Rickettsia asiatica AF394906
Rickettsia raoultii TYGS 3478
Rickettsia japonica AP011533
Rickettsia heilongjiangensis TYGS 369
Rickettsia honei U17645
Rickettsia slovaca TYGS 14180
Rickettsia parkeri L36673
Rickettsia sibirica TYGS 864
Rickettsia conorii AE006914
Rickettsia massiliae L36214
Rickettsia montanensis L36215
Rickettsia tamurae TYGS 6221
Rickettsia buchneri TYGS 3610
Rickettsia aeschlimannii U74757
Rickettsia rhipicephali L36216
Rickettsia gravesii TYGS 20187
Rickettsia canadensis L36104
Emcibacter nanhaiensis KJ191195
Emcibacter congregatus TYGS 20627
Defluviicoccus vanus AF179678
Tistrella mobilis AB071665
Tistrella bauzanensis GQ240228
Arboricoccus pini TYGS 19264
Geminicoccus roseus TYGS 1212
Ferrovibrio xuzhouensis KM978211
Ferrovibrio denitrificans GQ365620
Ferrovibrio soli KY117476
Taonella mepensis JN693496
Marinibaculum pumilum KT265740
Oceanibacterium hippocampi TYGS 22587
Sneathiella chinensis DQ219355
Sneathiella glossodoripedis TYGS 5257
Sneathiella chungangensis KF482756
Reyranella massiliensis TYGS 5136
Reyranella aquatilis KY363639
Reyranella terrae KP185143
Reyranella graminifolii AB839882
Reyranella soli JX260424
Lacibacterium aquatile HE795994
Elstera litoralis EU678309
Elstera cyanobacteriorum TYGS 11173
Inquilinus ginsengisoli AB245352
Inquilinus limosus TYGS 1448
Nitrospirillum amazonense X79735
Nitrospirillum iridis GU048666
Azospirillum irakense TYGS 1530
Niveispirillum fermenti JX843283
Niveispirillum cyanobacteriorum TYGS 11085
Niveispirillum lacus MF776580
Rhodocista pekingensis AF523824
Rhodospirillum centenum TYGS 15903
Desertibacter roseus EU833987
Desertibacter xinjiangensis KC625488
Skermanella xinjiangensis EU586202
Skermanella rosea LT545982
Skermanella parooensis X90760
Skermanella aerolata DQ672568
Skermanella stibiiresistens HQ315828
Azospirillum halopraeferens TYGS 1346
Azospirillum formosense GU256444
Roseomonas fauriae AY150046
Azospirillum brasilense AY324110
Azospirillum agricola KR296799
Azospirillum doebereinerae AJ238567
Azospirillum largimobile X90759
Azospirillum melinis DQ022958
Azospirillum lipoferum Z29619
Azospirillum humicireducens TYGS 3466
Azospirillum zeae DQ682470
Azospirillum oryzae AB185396
Azospirillum thiophilum TYGS 3655
Azospirillum picis AM922283
Azospirillum canadense DQ393891
Azospirillum rugosum AM419042
Azospirillum soli KC297124
Azospirillum fermentarium JX843282

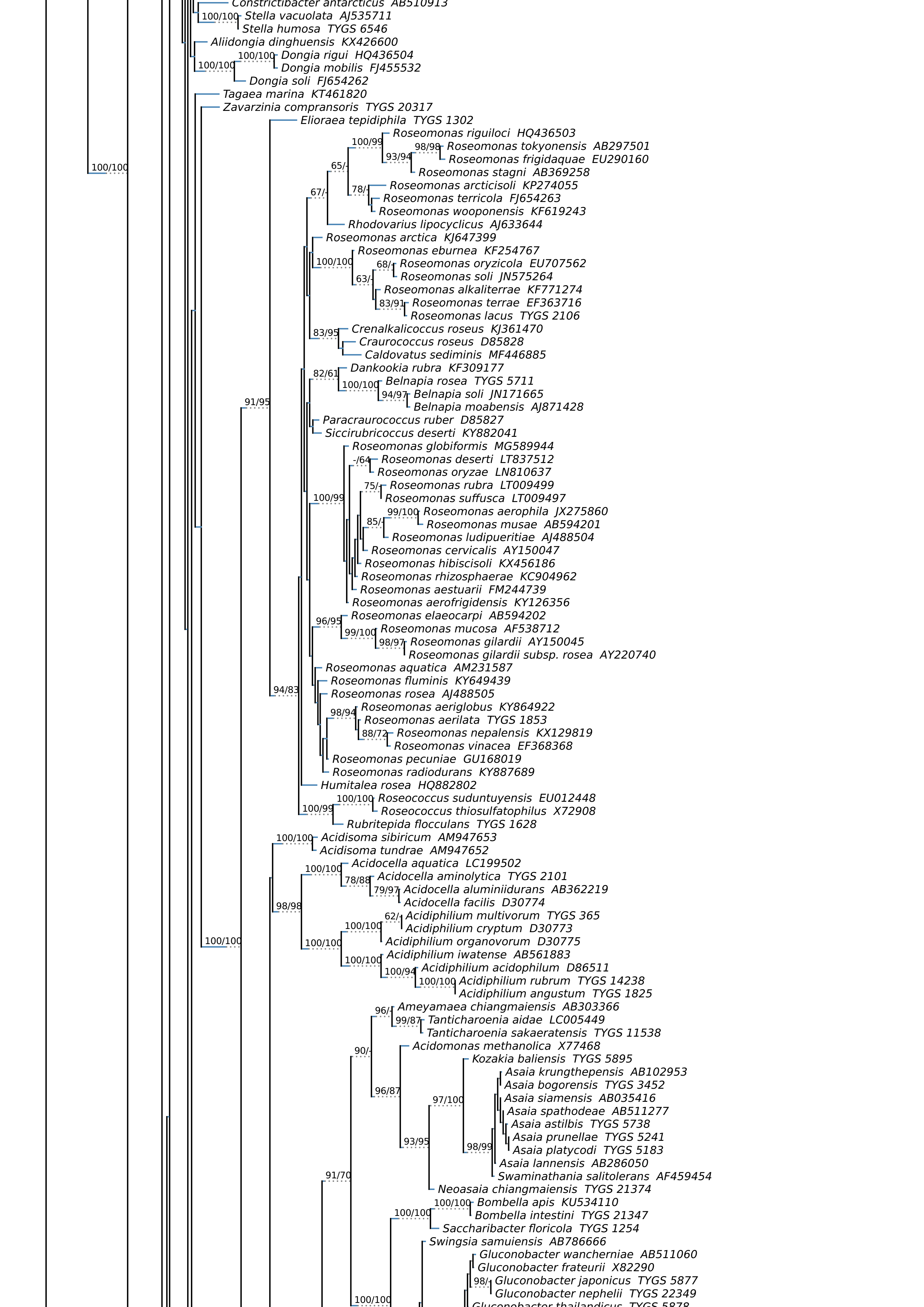
Constrictibacter antarcticus AB510913
Stella vacuolata AJ535711
Stella humosa TYGS 6546
Aliidongia dinghuensis KX426600
Dongia rigui HQ436504
Dongia mobilis FJ455532
Dongia soli FJ654262
Tagaea marina KT461820
Zavarzinia compransoris TYGS 20317
Elioraea tepidiphila TYGS 1302
Roseomonas riguiloci HQ436503
Roseomonas tokyonensis AB297501
Roseomonas frigidaquae EU290160
Roseomonas stagni AB369258
Roseomonas arcticisoli KP274055
Roseomonas terricola FJ654263
Roseomonas wooponensis KF619243
Rhodovarius lipocyclicus AJ633644
Roseomonas arctica KJ647399
Roseomonas eburnea KF254767
Roseomonas oryzicola EU707562
Roseomonas soli JN575264
Roseomonas alkaliterrae KF771274
Roseomonas terrae EF363716
Roseomonas lacus TYGS 2106
Crenalkalicoccus roseus KJ361470
Craurococcus roseus D85828
Caldovatus sediminis MF446885
Dankookia rubra KF309177
Belnapia rosea TYGS 5711
Belnapia soli JN171665
Belnapia moabensis AJ871428
Paracraurococcus ruber D85827
Siccirubricoccus deserti KY882041
Roseomonas globiformis MG589944
Roseomonas deserti LT837512
Roseomonas oryzae LN810637
Roseomonas rubra LT009499
Roseomonas suffusca LT009497
Roseomonas aerophila JX275860
Roseomonas musae AB594201
Roseomonas ludipueritiae AJ488504
Roseomonas cervicalis AY150047
Roseomonas hibiscisoli KX456186
Roseomonas rhizosphaerae KC904962
Roseomonas aestuarii FM244739
Roseomonas aerofrigidensis KY126356
Roseomonas elaeocarpi AB594202
Roseomonas mucosa AF538712
Roseomonas gilardii AY150045
Roseomonas gilardii subsp. rosea AY220740
Roseomonas aquatica AM231587
Roseomonas fluminis KY649439
Roseomonas rosea AJ488505
Roseomonas aeriglobus KY864922
Roseomonas aerilata TYGS 1853
Roseomonas nepalensis KX129819
Roseomonas vinacea EF368368
Roseomonas pecuniae GU168019
Roseomonas radiodurans KY887689
Humitalea rosea HQ882802
Roseococcus suduntuyensis EU012448
Roseococcus thiosulfatophilus X72908
Rubritepida flocculans TYGS 1628
Acidisoma sibiricum AM947653
Acidisoma tundrae AM947652
Acidocella aquatica LC199502
Acidocella aminolytica TYGS 2101
Acidocella aluminiidurans AB362219
Acidocella facilis D30774
Acidiphilium multivorum TYGS 365
Acidiphilium cryptum D30773
Acidiphilium organovorum D30775
Acidiphilium iwatense AB561883
Acidiphilium acidophilum D86511
Acidiphilium rubrum TYGS 14238
Acidiphilium angustum TYGS 1825
Ameyamaea chiangmaiensis AB303366
Tanticharoenia aidae LC005449
Tanticharoenia sakaeratensis TYGS 11538
Acidomonas methanolica X77468
Kozakia baliensis TYGS 5895
Asaia krungthepensis AB102953
Asaia bogorensis TYGS 3452
Asaia siamensis AB035416
Asaia spathodeae AB511277
Asaia astilbis TYGS 5738
Asaia prunellae TYGS 5241
Asaia platycodi TYGS 5183
Asaia lannensis AB286050
Swaminathania salitolerans AF459454
Neoasaia chiangmaiensis TYGS 21374
Bombella apis KU534110
Bombella intestini TYGS 21347
Saccharibacter floricola TYGS 1254
Swingsia samuiensis AB786666
Gluconobacter wancherniae AB511060
Gluconobacter frateurii X82290
Gluconobacter japonicus TYGS 5877
Gluconobacter nephelii TYGS 22349
Gluconobacter thailandicus TYGS 5878

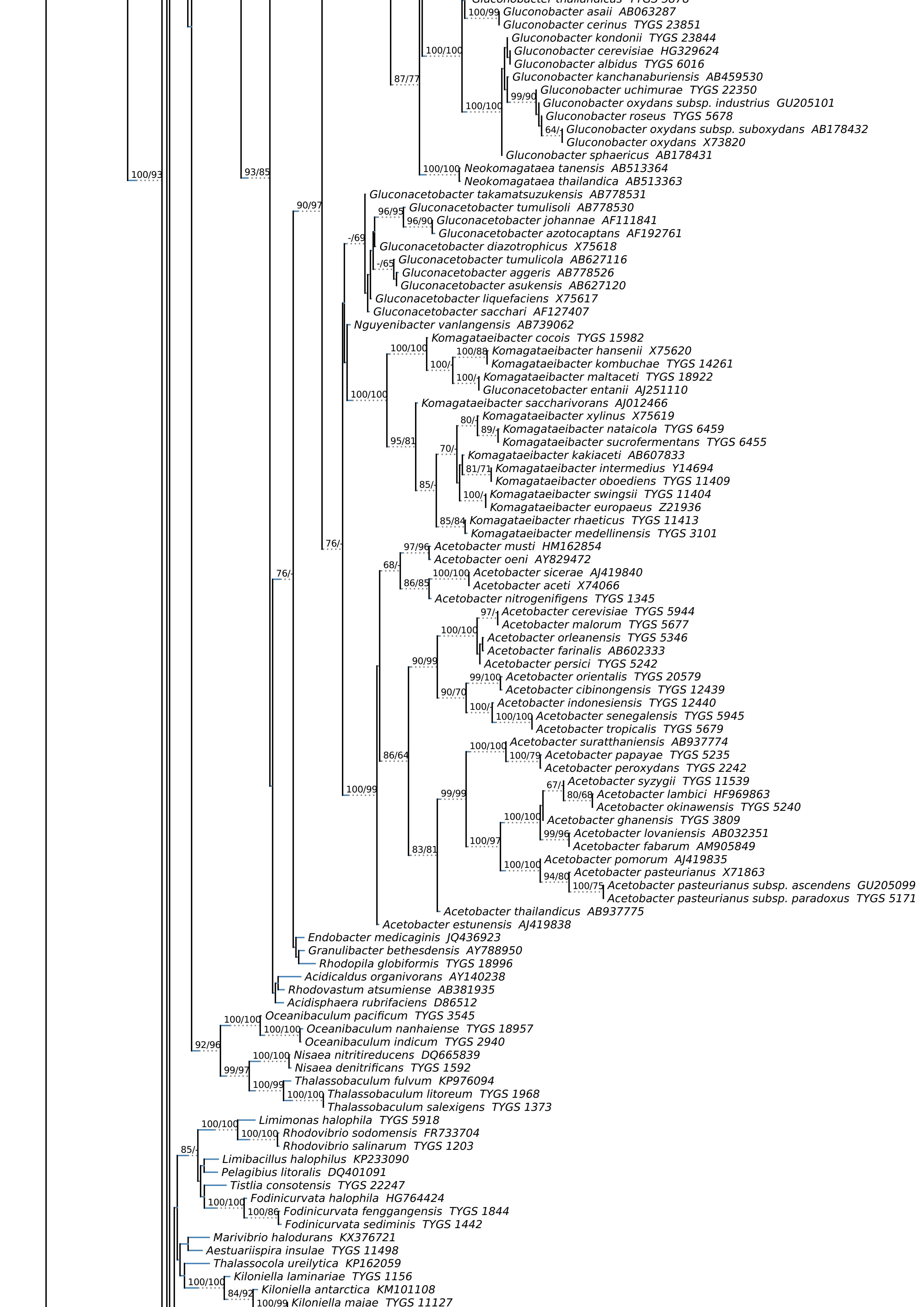

100/99 Gluconobacter asaii AB063287
Gluconobacter cerinus TYGS 23851
Gluconobacter kondonii TYGS 23844
100/100
Gluconobacter cerevisiae HG329624
Gluconobacter albidus TYGS 6016
87/77
Gluconobacter kanchanaburiensis AB459530
99/90 Gluconobacter uchimurae TYGS 22350
100/100
Gluconobacter oxydans subsp. industrius GU205101
Gluconobacter roseus TYGS 5678
64/- Gluconobacter oxydans subsp. suboxydans AB178432
Gluconobacter oxydans X73820
Gluconobacter sphaericus AB178431
100/93
93/85
100/100 Neokomagataea tanensis AB513364
Neokomagataea thailandica AB513363
90/97
Gluconacetobacter takamatsuzukensis AB778531
96/95 Gluconacetobacter tumulisoli AB778530
96/90 Gluconacetobacter johannae AF111841
Gluconacetobacter azotocaptans AF192761
-/69
Gluconacetobacter diazotrophicus X75618
-/65 Gluconacetobacter tumulicola AB627116
Gluconacetobacter aggeris AB778526
Gluconacetobacter asukensis AB627120
Gluconacetobacter liquefaciens X75617
Gluconacetobacter sacchari AF127407
Nguyenibacter vanlangensis AB739062
Komagataeibacter cocois TYGS 15982
100/100
100/88 Komagataeibacter hansenii X75620
100/- Komagataeibacter kombuchae TYGS 14261
100/- Komagataeibacter maltaceti TYGS 18922
100/100
Gluconacetobacter entanii AJ251110
Komagataeibacter saccharivorans AJ012466
80/- Komagataeibacter xylinus X75619
89/- Komagataeibacter nataicola TYGS 6459
95/81
Komagataeibacter sucrofermentans TYGS 6455
70/-
Komagataeibacter kakiaceti AB607833
81/71 Komagataeibacter intermedius Y14694
Komagataeibacter oboediens TYGS 11409
85/-
100/- Komagataeibacter swingsii TYGS 11404
Komagataeibacter europaeus Z21936
85/84 Komagataeibacter rhaeticus TYGS 11413
Komagataeibacter medellinensis TYGS 3101
76/-
97/96 Acetobacter musti HM162854
Acetobacter oeni AY829472
68/-
100/100 Acetobacter sicerae AJ419840
76/-
Acetobacter aceti X74066
86/85
Acetobacter nitrogenifigens TYGS 1345
97/- Acetobacter cerevisiae TYGS 5944
Acetobacter malorum TYGS 5677
100/100
Acetobacter orleanensis TYGS 5346
Acetobacter farinalis AB602333
Acetobacter persici TYGS 5242
90/99
99/100 Acetobacter orientalis TYGS 20579
Acetobacter cibinongensis TYGS 12439
90/70
100/- Acetobacter indonesiensis TYGS 12440
100/100 Acetobacter senegalensis TYGS 5945
Acetobacter tropicalis TYGS 5679
100/100 Acetobacter suratthaniensis AB937774
86/64
100/79 Acetobacter papayae TYGS 5235
Acetobacter peroxydans TYGS 2242
67/- Acetobacter syzygii TYGS 11539
99/99
80/68 Acetobacter lambici HF969863
100/99
Acetobacter okinawensis TYGS 5240
100/100 Acetobacter ghanensis TYGS 3809
99/96 Acetobacter lovaniensis AB032351
100/97
Acetobacter fabarum AM905849
83/81
100/100 Acetobacter pomorum AJ419835
94/80 Acetobacter pasteurianus X71863
100/75 Acetobacter pasteurianus subsp. ascendens GU205099
Acetobacter pasteurianus subsp. paradoxus TYGS 5171
Acetobacter thailandicus AB937775
Acetobacter estunensis AJ419838
Endobacter medicaginis JQ436923
Granulibacter bethesdensis AY788950
Rhodopila globiformis TYGS 18996
Acidicaldus organivorans AY140238
Rhodovastum atsumiense AB381935
Acidisphaera rubrifaciens D86512
100/100 Oceanibaculum pacificum TYGS 3545
100/100 Oceanibaculum nanhaiense TYGS 18957
Oceanibaculum indicum TYGS 2940
92/96
100/100 Nisaea nitritireducens DQ665839
Nisaea denitrificans TYGS 1592
99/97
100/99 Thalassobaculum fulvum KP976094
100/100 Thalassobaculum litoreum TYGS 1968
Thalassobaculum salexigens TYGS 1373
100/100 Limimonas halophila TYGS 5918
100/100 Rhodovibrio sodomensis FR733704
85/-
Rhodovibrio salinarum TYGS 1203
Limibacillus halophilus KP233090
Pelagibius litoralis DQ401091
Tistlia consotensis TYGS 22247
100/100 Fodinicurvata halophila HG764424
100/86 Fodinicurvata fenggangensis TYGS 1844
Fodinicurvata sediminis TYGS 1442
Marivibrio halodurans KX376721
Aestuariispira insulae TYGS 11498
Thalassocola ureilytica KP162059
100/100 Kiloniella laminariae TYGS 1156
84/92 Kiloniella antarctica KM101108
100/99 Kiloniella majae TYGS 11127

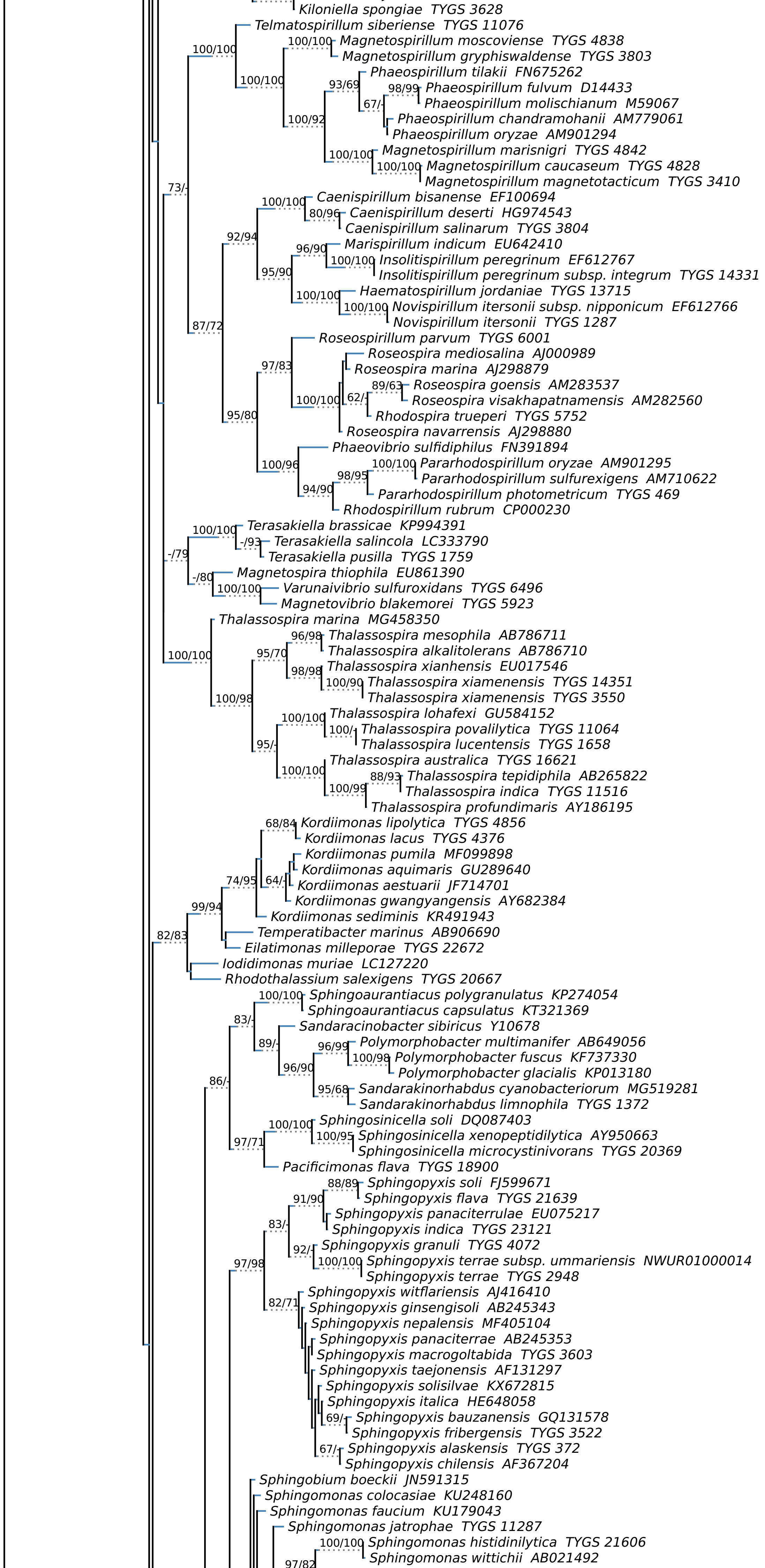
Kiloniella spongiae TYGS 3628
Telmatospirillum siberiense TYGS 11076
Magnetospirillum moscoviense TYGS 4838
Magnetospirillum gryphiswaldense TYGS 3803
Phaeospirillum tilakii FN675262
Phaeospirillum fulvum D14433
Phaeospirillum molischianum M59067
Phaeospirillum chandramohanii AM779061
Phaeospirillum oryzae AM901294
Magnetospirillum marisnigri TYGS 4842
Magnetospirillum caucaseum TYGS 4828
Magnetospirillum magnetotacticum TYGS 3410
Caenispirillum bisanense EF100694
Caenispirillum deserti HG974543
Caenispirillum salinarum TYGS 3804
Marispirillum indicum EU642410
Insolitispirillum peregrinum EF612767
Insolitispirillum peregrinum subsp. integrum TYGS 14331
Haematospirillum jordaniae TYGS 13715
Novispirillum itersonii subsp. nipponicum EF612766
Novispirillum itersonii TYGS 1287
Roseospirillum parvum TYGS 6001
Roseospira mediosalina AJ000989
Roseospira marina AJ298879
Roseospira goensis AM283537
Roseospira visakhapatnamensis AM282560
Rhodospira trueperi TYGS 5752
Roseospira navarrensis AJ298880
Phaeovibrio sulfidiphilus FN391894
Pararhodospirillum oryzae AM901295
Pararhodospirillum sulfurexigens AM710622
Pararhodospirillum photometricum TYGS 469
Rhodospirillum rubrum CP000230
Terasakiella brassicae KP994391
Terasakiella salincola LC333790
Terasakiella pusilla TYGS 1759
Magnetospira thiophila EU861390
Varunaivibrio sulfuroxidans TYGS 6496
Magnetovibrio blakemorei TYGS 5923
Thalassospira marina MG458350
Thalassospira mesophila AB786711
Thalassospira alkalitolerans AB786710
Thalassospira xianhensis EU017546
Thalassospira xiamenensis TYGS 14351
Thalassospira xiamenensis TYGS 3550
Thalassospira lohafexi GU584152
Thalassospira povalilytica TYGS 11064
Thalassospira lucentensis TYGS 1658
Thalassospira australica TYGS 16621
Thalassospira tepidiphila AB265822
Thalassospira indica TYGS 11516
Thalassospira profundimaris AY186195
Kordiimonas lipolytica TYGS 4856
Kordiimonas lacus TYGS 4376
Kordiimonas pumila MF099898
Kordiimonas aquimaris GU289640
Kordiimonas aestuarii JF714701
Kordiimonas gwangyangensis AY682384
Kordiimonas sediminis KR491943
Temperatibacter marinus AB906690
Eilatimonas milleporae TYGS 22672
Iodidimonas muriae LC127220
Rhodothalassium salexigens TYGS 20667
Sphingoaurantiacus polygranulatus KP274054
Sphingoaurantiacus capsulatus KT321369
Sandaracinobacter sibiricus Y10678
Polymorphobacter multimanifer AB649056
Polymorphobacter fuscus KF737330
Polymorphobacter glacialis KP013180
Sandarakinorhabdus cyanobacteriorum MG519281
Sandarakinorhabdus limnophila TYGS 1372
Sphingosinicella soli DQ087403
Sphingosinicella xenopeptidilytica AY950663
Sphingosinicella microcystinivorans TYGS 20369
Pacificimonas flava TYGS 18900
Sphingopyxis soli FJ599671
Sphingopyxis flava TYGS 21639
Sphingopyxis panaciterrulae EU075217
Sphingopyxis indica TYGS 23121
Sphingopyxis granuli TYGS 4072
Sphingopyxis terrae subsp. ummariensis NWUR01000014
Sphingopyxis terrae TYGS 2948
Sphingopyxis witflariensis AJ416410
Sphingopyxis ginsengisoli AB245343
Sphingopyxis nepalensis MF405104
Sphingopyxis panaciterrae AB245353
Sphingopyxis macrogoltabida TYGS 3603
Sphingopyxis taejonensis AF131297
Sphingopyxis solisilvae KX672815
Sphingopyxis italica HE648058
Sphingopyxis bauzanensis GQ131578
Sphingopyxis fribergensis TYGS 3522
Sphingopyxis alaskensis TYGS 372
Sphingopyxis chilensis AF367204
Sphingobium boeckii JN591315
Sphingomonas colocasiae KU248160
Sphingomonas faucium KU179043
Sphingomonas jatrophae TYGS 11287
Sphingomonas histidinilytica TYGS 21606
Sphingomonas wittichii AB021492

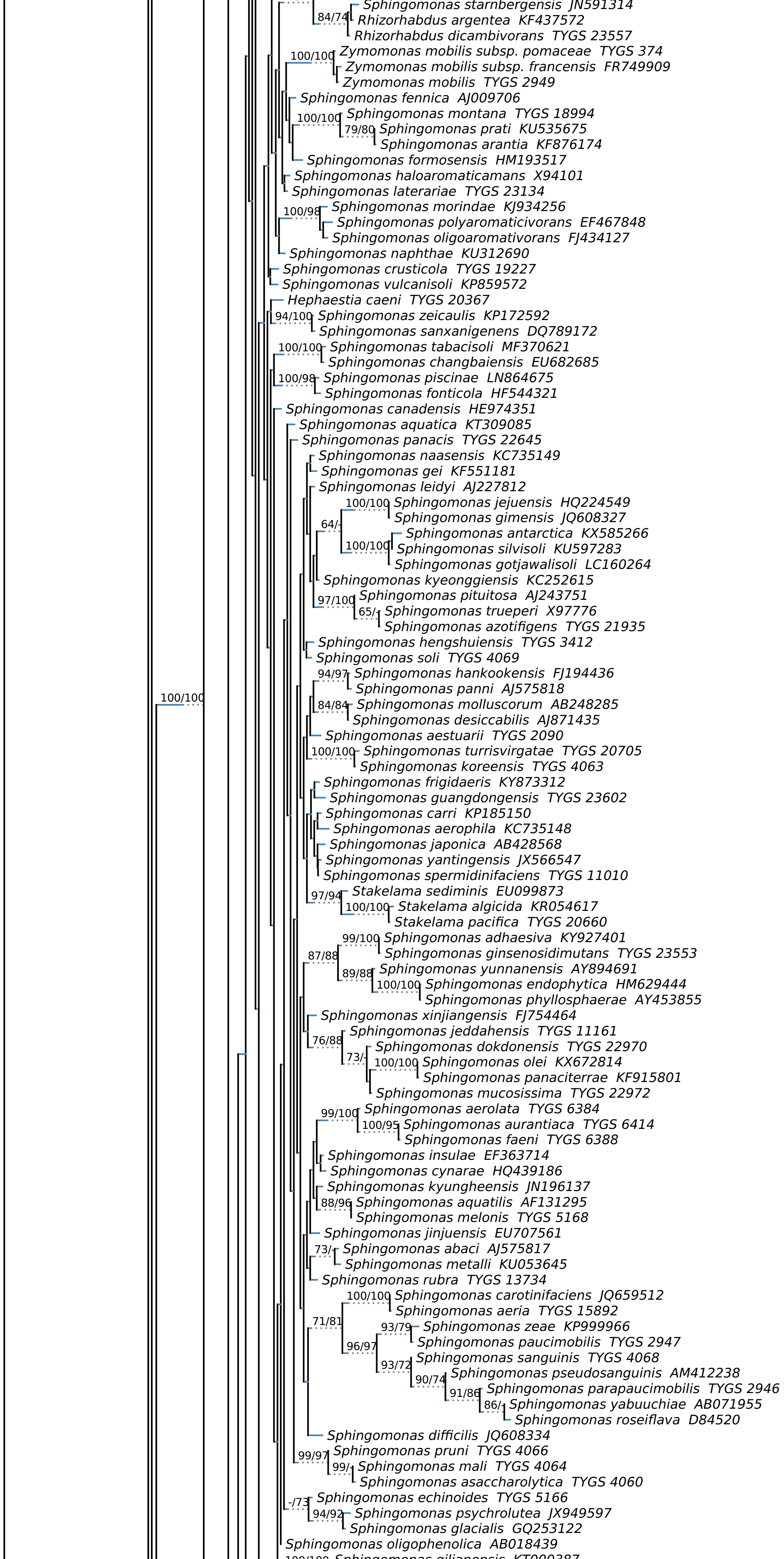
Sphingomonas starnbergensis JN591314
84/74
Rhizorhabdus argentea KF437572
Rhizorhabdus dicambivorans TYGS 23557
100/100
Zymomonas mobilis subsp. pomaceae TYGS 374
Zymomonas mobilis subsp. francensis FR749909
Zymomonas mobilis TYGS 2949
Sphingomonas fennica AJ009706
100/100
Sphingomonas montana TYGS 18994
79/80
Sphingomonas prati KU535675
Sphingomonas arantia KF876174
Sphingomonas formosensis HM193517
Sphingomonas haloaromaticamans X94101
Sphingomonas laterariae TYGS 23134
100/98
Sphingomonas morindae KJ934256
Sphingomonas polyaromaticivorans EF467848
Sphingomonas oligoaromativorans FJ434127
Sphingomonas naphthae KU312690
Sphingomonas crusticola TYGS 19227
Sphingomonas vulcanisoli KP859572
Hephaestia caeni TYGS 20367
94/100
Sphingomonas zeicaulis KP172592
Sphingomonas sanxanigenens DQ789172
100/100
Sphingomonas tabacisoli MF370621
Sphingomonas changbaiensis EU682685
100/98
Sphingomonas piscinae LN864675
Sphingomonas fonticola HF544321
Sphingomonas canadensis HE974351
Sphingomonas aquatica KT309085
Sphingomonas panacis TYGS 22645
Sphingomonas naasensis KC735149
Sphingomonas gei KF551181
Sphingomonas leidyi AJ227812
100/100
Sphingomonas jejuensis HQ224549
64/-
Sphingomonas gimensis JQ608327
Sphingomonas antarctica KX585266
100/100
Sphingomonas silvisoli KU597283
Sphingomonas gotjawalisoli LC160264
Sphingomonas kyeonggiensis KC252615
97/100
Sphingomonas pituitosa AJ243751
65/-
Sphingomonas trueperi X97776
Sphingomonas azotifigens TYGS 21935
Sphingomonas hengshuiensis TYGS 3412
Sphingomonas soli TYGS 4069
94/97
Sphingomonas hankookensis FJ194436
Sphingomonas panni AJ575818
84/84
Sphingomonas molluscorum AB248285
Sphingomonas desiccabilis AJ871435
Sphingomonas aestuarii TYGS 2090
100/100
100/100
Sphingomonas turrisvirgatae TYGS 20705
Sphingomonas koreensis TYGS 4063
Sphingomonas frigidaeris KY873312
Sphingomonas guangdongensis TYGS 23602
Sphingomonas carri KP185150
Sphingomonas aerophila KC735148
Sphingomonas japonica AB428568
Sphingomonas yantingensis JX566547
Sphingomonas spermidinifaciens TYGS 11010
97/94
Stakelama sediminis EU099873
100/100
Stakelama algicida KR054617
Stakelama pacifica TYGS 20660
99/100
Sphingomonas adhaesiva KY927401
87/88
Sphingomonas ginsenosidimutans TYGS 23553
89/88
Sphingomonas yunnanensis AY894691
100/100
Sphingomonas endophytica HM629444
Sphingomonas phyllosphaerae AY453855
Sphingomonas xinjiangensis FJ754464
Sphingomonas jeddahensis TYGS 11161
76/88
Sphingomonas dokdonensis TYGS 22970
73/-
100/100
Sphingomonas olei KX672814
Sphingomonas panaciterrae KF915801
Sphingomonas mucosissima TYGS 22972
99/100
Sphingomonas aerolata TYGS 6384
100/95
Sphingomonas aurantiaca TYGS 6414
Sphingomonas faeni TYGS 6388
Sphingomonas insulae EF363714
Sphingomonas cynarae HQ439186
Sphingomonas kyungheensis JN196137
88/96
Sphingomonas aquatilis AF131295
Sphingomonas melonis TYGS 5168
Sphingomonas jinjuensis EU707561
73/-
Sphingomonas abaci AJ575817
Sphingomonas metalli KU053645
Sphingomonas rubra TYGS 13734
100/100
Sphingomonas carotinifaciens JQ659512
Sphingomonas aeria TYGS 15892
71/81
93/79
Sphingomonas zeae KP999966
96/97
Sphingomonas paucimobilis TYGS 2947
93/72
Sphingomonas sanguinis TYGS 4068
90/74
Sphingomonas pseudosanguinis AM412238
91/86
Sphingomonas parapaucimobilis TYGS 2946
86/-
Sphingomonas yabuuchiae AB071955
Sphingomonas roseiflava D84520
Sphingomonas difficilis JQ608334
99/97
Sphingomonas pruni TYGS 4066
99/-
Sphingomonas mali TYGS 4064
Sphingomonas asaccharolytica TYGS 4060
-/73
Sphingomonas echinoides TYGS 5166
94/92
Sphingomonas psychrolutea JX949597
Sphingomonas glacialis GQ253122
Sphingomonas oligophenolica AB018439

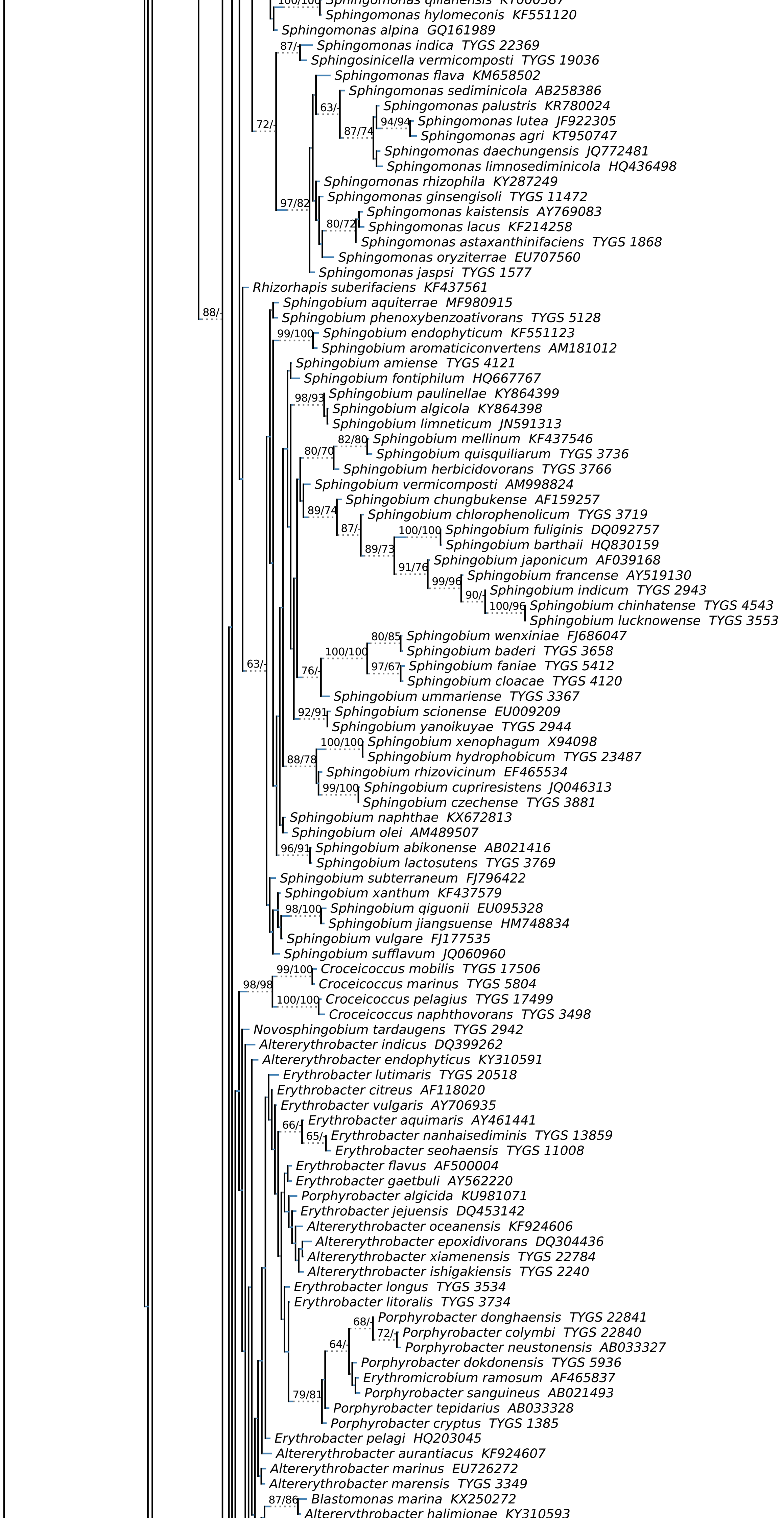

Sphingomonas hylomeconis KF551120
Sphingomonas alpina GQ161989
Sphingomonas indica TYGS 22369
Sphingosinicella vermicomposti TYGS 19036
Sphingomonas flava KM658502
Sphingomonas sediminicola AB258386
Sphingomonas palustris KR780024
Sphingomonas lutea JF922305
Sphingomonas agri KT950747
Sphingomonas daechungensis JQ772481
Sphingomonas limnosediminicola HQ436498
Sphingomonas rhizophila KY287249
Sphingomonas ginsengisoli TYGS 11472
Sphingomonas kaistensis AY769083
Sphingomonas lacus KF214258
Sphingomonas astaxanthinifaciens TYGS 1868
Sphingomonas oryziterrae EU707560
Sphingomonas jaspsi TYGS 1577
Rhizorhapis suberifaciens KF437561
Sphingobium aquiterrae MF980915
Sphingobium phenoxybenzoativorans TYGS 5128
Sphingobium endophyticum KF551123
Sphingobium aromaticiconvertens AM181012
Sphingobium amiense TYGS 4121
Sphingobium fontiphilum HQ667767
Sphingobium paulinellae KY864399
Sphingobium algicola KY864398
Sphingobium limneticum JN591313
Sphingobium mellinum KF437546
Sphingobium quisquiliarum TYGS 3736
Sphingobium herbicidovorans TYGS 3766
Sphingobium vermicomposti AM998824
Sphingobium chungbukense AF159257
Sphingobium chlorophenolicum TYGS 3719
Sphingobium fuliginis DQ092757
Sphingobium barthaii HQ830159
Sphingobium japonicum AF039168
Sphingobium francense AY519130
Sphingobium indicum TYGS 2943
Sphingobium chinhatense TYGS 4543
Sphingobium lucknowense TYGS 3553
Sphingobium wenxiniae FJ686047
Sphingobium baderi TYGS 3658
Sphingobium faniae TYGS 5412
Sphingobium cloacae TYGS 4120
Sphingobium ummariense TYGS 3367
Sphingobium scionense EU009209
Sphingobium yanoikuyae TYGS 2944
Sphingobium xenophagum X94098
Sphingobium hydrophobicum TYGS 23487
Sphingobium rhizovicinum EF465534
Sphingobium cupriresistens JQ046313
Sphingobium czechense TYGS 3881
Sphingobium naphthae KX672813
Sphingobium olei AM489507
Sphingobium abikonense AB021416
Sphingobium lactosutens TYGS 3769
Sphingobium subterraneum FJ796422
Sphingobium xanthum KF437579
Sphingobium qiguonii EU095328
Sphingobium jiangsuense HM748834
Sphingobium vulgare FJ177535
Sphingobium sufflavum JQ060960
Croceicoccus mobilis TYGS 17506
Croceicoccus marinus TYGS 5804
Croceicoccus pelagius TYGS 17499
Croceicoccus naphthovorans TYGS 3498
Novosphingobium tardaugens TYGS 2942
Altererythrobacter indicus DQ399262
Altererythrobacter endophyticus KY310591
Erythrobacter lutimaris TYGS 20518
Erythrobacter citreus AF118020
Erythrobacter vulgaris AY706935
Erythrobacter aquimaris AY461441
Erythrobacter nanhaisediminis TYGS 13859
Erythrobacter seohaensis TYGS 11008
Erythrobacter flavus AF500004
Erythrobacter gaetbuli AY562220
Porphyrobacter algicida KU981071
Erythrobacter jejuensis DQ453142
Altererythrobacter oceanensis KF924606
Altererythrobacter epoxidivorans DQ304436
Altererythrobacter xiamenensis TYGS 22784
Altererythrobacter ishigakiensis TYGS 2240
Erythrobacter longus TYGS 3534
Erythrobacter litoralis TYGS 3734
Porphyrobacter donghaensis TYGS 22841
Porphyrobacter colymbi TYGS 22840
Porphyrobacter neustonensis AB033327
Porphyrobacter dokdonensis TYGS 5936
Erythromicrobium ramosum AF465837
Porphyrobacter sanguineus AB021493
Porphyrobacter tepidarius AB033328
Porphyrobacter cryptus TYGS 1385
Erythrobacter pelagi HQ203045
Altererythrobacter aurantiacus KF924607
Altererythrobacter marinus EU726272
Altererythrobacter marensis TYGS 3349
Blastomonas marina KX250272
Altererythrobacter halimionae KY310593

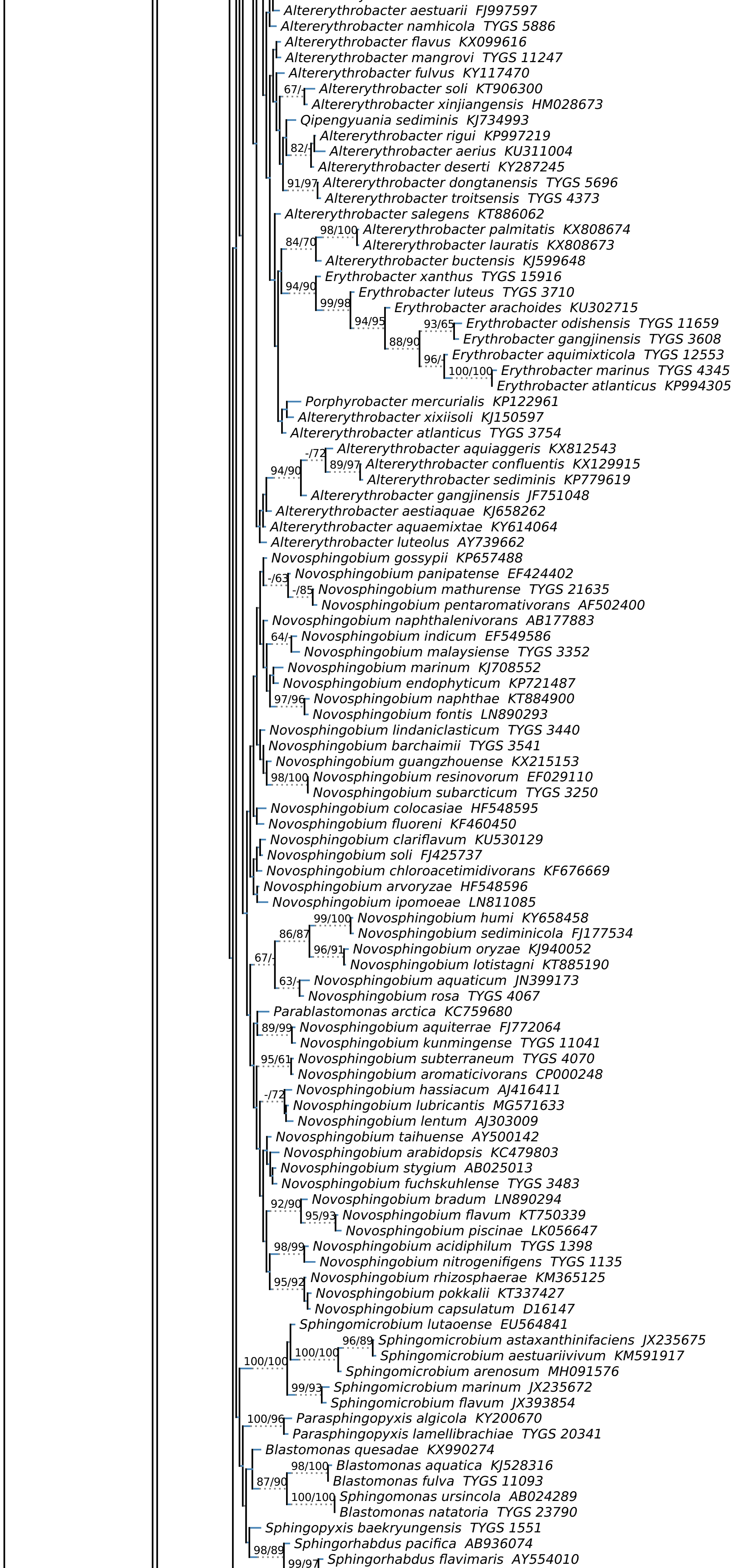
Altererythrobacter aestuarii FJ997597
Altererythrobacter namhicola TYGS 5886
Altererythrobacter flavus KX099616
Altererythrobacter mangrovi TYGS 11247
Altererythrobacter fulvus KY117470
67/-
Altererythrobacter soli KT906300
Altererythrobacter xinjiangensis HM028673
Qipengyuania sediminis KJ734993
Altererythrobacter rigui KP997219
82/-
Altererythrobacter aerius KU311004
Altererythrobacter deserti KY287245
91/97
Altererythrobacter dongtanensis TYGS 5696
Altererythrobacter troitsensis TYGS 4373
Altererythrobacter salegens KT886062
98/100
Altererythrobacter palmitatis KX808674
84/70
Altererythrobacter lauratis KX808673
Altererythrobacter buctensis KJ599648
94/90
Erythrobacter xanthus TYGS 15916
99/98
Erythrobacter luteus TYGS 3710
Erythrobacter arachoides KU302715
94/95
93/65
Erythrobacter odishensis TYGS 11659
88/90
Erythrobacter gangjinensis TYGS 3608
96/-
Erythrobacter aquimixticola TYGS 12553
100/100
Erythrobacter marinus TYGS 4345
Erythrobacter atlanticus KP994305
Porphyrobacter mercurialis KP122961
Altererythrobacter xixiisoli KJ150597
Altererythrobacter atlanticus TYGS 3754
-/72
Altererythrobacter aquiaggeris KX812543
94/90
89/97
Altererythrobacter confluentis KX129915
Altererythrobacter sediminis KP779619
Altererythrobacter gangjinensis JF751048
Altererythrobacter aestiaquae KJ658262
Altererythrobacter aquaemixtae KY614064
Altererythrobacter luteolus AY739662
Novosphingobium gossypii KP657488
-/63
Novosphingobium panipatense EF424402
-/85
Novosphingobium mathurense TYGS 21635
Novosphingobium pentaromativorans AF502400
Novosphingobium naphthalenivorans AB177883
64/-
Novosphingobium indicum EF549586
Novosphingobium malaysiense TYGS 3352
Novosphingobium marinum KJ708552
Novosphingobium endophyticum KP721487
97/96
Novosphingobium naphthae KT884900
Novosphingobium fontis LN890293
Novosphingobium lindaniclasticum TYGS 3440
Novosphingobium barchaimii TYGS 3541
Novosphingobium guangzhouense KX215153
98/100
Novosphingobium resinovorum EF029110
Novosphingobium subarcticum TYGS 3250
Novosphingobium colocasiae HF548595
Novosphingobium fluoreni KF460450
Novosphingobium clariflavum KU530129
Novosphingobium soli FJ425737
Novosphingobium chloroacetimidivorans KF676669
Novosphingobium arvoryzae HF548596
Novosphingobium ipomoeae LN811085
99/100
Novosphingobium humi KY658458
86/87
Novosphingobium sediminicola FJ177534
67/-
96/91
Novosphingobium oryzae KJ940052
Novosphingobium lotistagni KT885190
63/-
Novosphingobium aquaticum JN399173
Novosphingobium rosa TYGS 4067
Parablastomonas arctica KC759680
89/99
Novosphingobium aquiterrae FJ772064
Novosphingobium kunmingense TYGS 11041
95/61
Novosphingobium subterraneum TYGS 4070
Novosphingobium aromaticivorans CP000248
Novosphingobium hassiacum AJ416411
-/72
Novosphingobium lubricantis MG571633
Novosphingobium lentum AJ303009
Novosphingobium taihuense AY500142
Novosphingobium arabidopsis KC479803
Novosphingobium stygium AB025013
Novosphingobium fuchskuhlense TYGS 3483
92/90
Novosphingobium bradum LN890294
95/93
Novosphingobium flavum KT750339
Novosphingobium piscinae LK056647
98/99
Novosphingobium acidiphilum TYGS 1398
Novosphingobium nitrogenifigens TYGS 1135
95/92
Novosphingobium rhizosphaerae KM365125
Novosphingobium pokkalii KT337427
Novosphingobium capsulatum D16147
Sphingomicrobium lutaoense EU564841
96/89
Sphingomicrobium astaxanthinifaciens JX235675
100/100
100/100
Sphingomicrobium aestuariivivum KM591917
Sphingomicrobium arenosum MH091576
99/93
Sphingomicrobium marinum JX235672
Sphingomicrobium flavum JX393854
100/96
Parasphingopyxis algicola KY200670
Parasphingopyxis lamellibrachiae TYGS 20341
Blastomonas quesadae KX990274
98/100
Blastomonas aquatica KJ528316
87/90
Blastomonas fulva TYGS 11093
100/100
Sphingomonas ursincola AB024289
Blastomonas natatoria TYGS 23790
Sphingopyxis baekryungensis TYGS 1551
98/89
Sphingorhabdus pacifica AB936074
99/97
Sphingorhabdus flavimaris AY554010

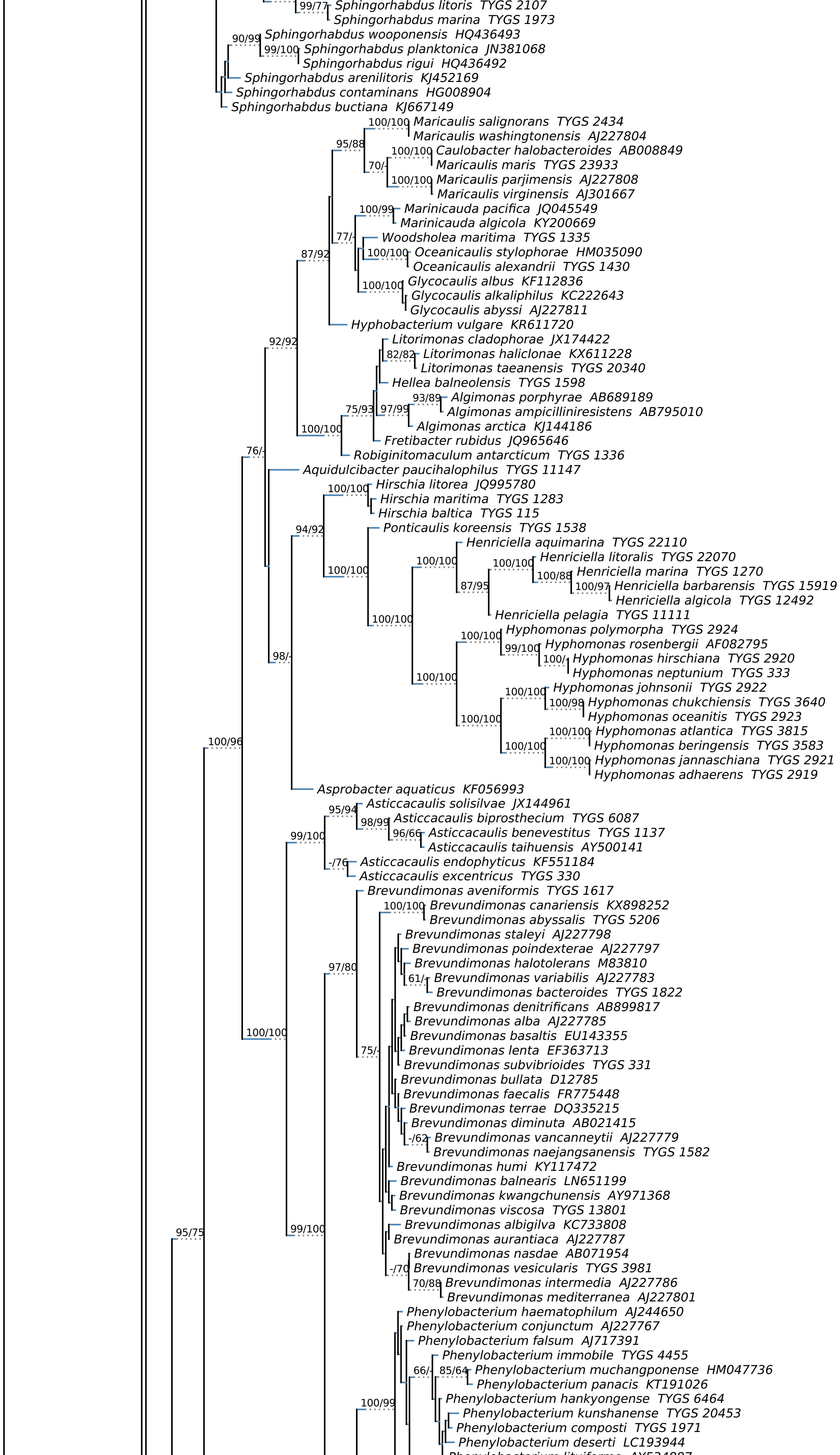

Sphingorhabdus litoris TYGS 2107
Sphingorhabdus marina TYGS 1973
Sphingorhabdus wooponensis HQ436493
Sphingorhabdus planktonica JN381068
Sphingorhabdus rigui HQ436492
Sphingorhabdus arenilitoris KJ452169
Sphingorhabdus contaminans HG008904
Sphingorhabdus buctiana KJ667149
Maricaulis salignorans TYGS 2434
Maricaulis washingtonensis AJ227804
Caulobacter halobacteroides AB008849
Maricaulis maris TYGS 23933
Maricaulis parjimensis AJ227808
Maricaulis virginensis AJ301667
Marinicauda pacifica JQ045549
Marinicauda algicola KY200669
Woodsholea maritima TYGS 1335
Oceanicaulis stylophorae HM035090
Oceanicaulis alexandrii TYGS 1430
Glycocaulis albus KF112836
Glycocaulis alkaliphilus KC222643
Glycocaulis abyssi AJ227811
Hyphobacterium vulgare KR611720
Litorimonas cladophorae JX174422
Litorimonas haliclonae KX611228
Litorimonas taeanensis TYGS 20340
Hellea balneolensis TYGS 1598
Algimonas porphyrae AB689189
Algimonas ampicilliniresistens AB795010
Algimonas arctica KJ144186
Fretibacter rubidus JQ965646
Robiginitomaculum antarcticum TYGS 1336
Aquidulcibacter paucihalophilus TYGS 11147
Hirschia litorea JQ995780
Hirschia maritima TYGS 1283
Hirschia baltica TYGS 115
Ponticaulis koreensis TYGS 1538
Henriciella aquimarina TYGS 22110
Henriciella litoralis TYGS 22070
Henriciella marina TYGS 1270
Henriciella barbarensis TYGS 15919
Henriciella algicola TYGS 12492
Henriciella pelagia TYGS 11111
Hyphomonas polymorpha TYGS 2924
Hyphomonas rosenbergii AF082795
Hyphomonas hirschiana TYGS 2920
Hyphomonas neptunium TYGS 333
Hyphomonas johnsonii TYGS 2922
Hyphomonas chukchiensis TYGS 3640
Hyphomonas oceanitis TYGS 2923
Hyphomonas atlantica TYGS 3815
Hyphomonas beringensis TYGS 3583
Hyphomonas jannaschiana TYGS 2921
Hyphomonas adhaerens TYGS 2919
Asprobacter aquaticus KF056993
Asticcacaulis solisilvae JX144961
Asticcacaulis biprosthecium TYGS 6087
Asticcacaulis benevestitus TYGS 1137
Asticcacaulis taihuensis AY500141
Asticcacaulis endophyticus KF551184
Asticcacaulis excentricus TYGS 330
Brevundimonas aveniformis TYGS 1617
Brevundimonas canariensis KX898252
Brevundimonas abyssalis TYGS 5206
Brevundimonas staleyi AJ227798
Brevundimonas poindexterae AJ227797
Brevundimonas halotolerans M83810
Brevundimonas variabilis AJ227783
Brevundimonas bacteroides TYGS 1822
Brevundimonas denitrificans AB899817
Brevundimonas alba AJ227785
Brevundimonas basaltis EU143355
Brevundimonas lenta EF363713
Brevundimonas subvibrioides TYGS 331
Brevundimonas bullata D12785
Brevundimonas faecalis FR775448
Brevundimonas terrae DQ335215
Brevundimonas diminuta AB021415
Brevundimonas vancanneytii AJ227779
Brevundimonas naejangsanensis TYGS 1582
Brevundimonas humi KY117472
Brevundimonas balnearis LN651199
Brevundimonas kwangchunensis AY971368
Brevundimonas viscosa TYGS 13801
Brevundimonas albigilva KC733808
Brevundimonas aurantiaca AJ227787
Brevundimonas nasdae AB071954
Brevundimonas vesicularis TYGS 3981
Brevundimonas intermedia AJ227786
Brevundimonas mediterranea AJ227801
Phenylobacterium haematophilum AJ244650
Phenylobacterium conjunctum AJ227767
Phenylobacterium falsum AJ717391
Phenylobacterium immobile TYGS 4455
Phenylobacterium muchangponense HM047736
Phenylobacterium panacis KT191026
Phenylobacterium hankyongense TYGS 6464
Phenylobacterium kunshanense TYGS 20453
Phenylobacterium composti TYGS 1971
Phenylobacterium deserti LC193944

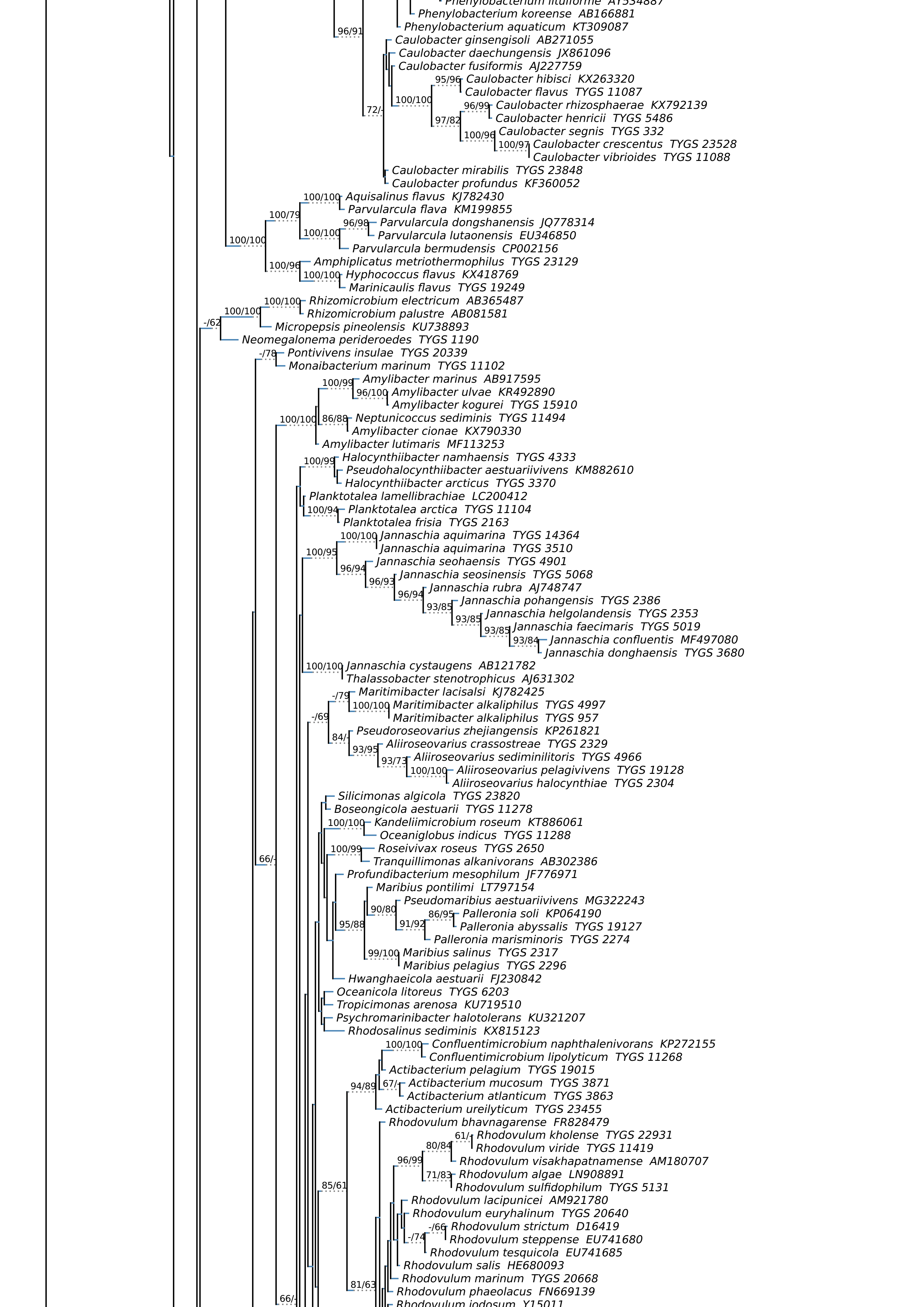
Phenylobacterium koreense AB166881
Phenylobacterium aquaticum KT309087
Caulobacter ginsengisoli AB271055
Caulobacter daechungensis JX861096
Caulobacter fusiformis AJ227759
Caulobacter hibisci KX263320
Caulobacter flavus TYGS 11087
Caulobacter rhizosphaerae KX792139
Caulobacter henricii TYGS 5486
Caulobacter segnis TYGS 332
Caulobacter crescentus TYGS 23528
Caulobacter vibrioides TYGS 11088
Caulobacter mirabilis TYGS 23848
Caulobacter profundus KF360052
Aquisalinus flavus KJ782430
Parvularcula flava KM199855
Parvularcula dongshanensis JQ778314
Parvularcula lutaonensis EU346850
Parvularcula bermudensis CP002156
Amphiplicatus metriothermophilus TYGS 23129
Hyphococcus flavus KX418769
Marinicaulis flavus TYGS 19249
Rhizomicrobium electricum AB365487
Rhizomicrobium palustre AB081581
Micropepsis pineolensis KU738893
Neomegalonema perideroedes TYGS 1190
Pontivivens insulae TYGS 20339
Monaibacterium marinum TYGS 11102
Amylibacter marinus AB917595
Amylibacter ulvae KR492890
Amylibacter kogurei TYGS 15910
Neptunicoccus sediminis TYGS 11494
Amylibacter cionae KX790330
Amylibacter lutimaris MF113253
Halocynthiibacter namhaensis TYGS 4333
Pseudohalocynthiibacter aestuariivivens KM882610
Halocynthiibacter arcticus TYGS 3370
Planktotalea lamellibrachiae LC200412
Planktotalea arctica TYGS 11104
Planktotalea frisia TYGS 2163
Jannaschia aquimarina TYGS 14364
Jannaschia aquimarina TYGS 3510
Jannaschia seohaensis TYGS 4901
Jannaschia seosinensis TYGS 5068
Jannaschia rubra AJ748747
Jannaschia pohangensis TYGS 2386
Jannaschia helgolandensis TYGS 2353
Jannaschia faecimaris TYGS 5019
Jannaschia confluentis MF497080
Jannaschia donghaensis TYGS 3680
Jannaschia cystaugens AB121782
Thalassobacter stenotrophicus AJ631302
Maritimibacter lacisalsi KJ782425
Maritimibacter alkaliphilus TYGS 4997
Maritimibacter alkaliphilus TYGS 957
Pseudoroseovarius zhejiangensis KP261821
Aliiroseovarius crassostreae TYGS 2329
Aliiroseovarius sediminilitoris TYGS 4966
Aliiroseovarius pelagivivens TYGS 19128
Aliiroseovarius halocynthiae TYGS 2304
Silicimonas algicola TYGS 23820
Boseongicola aestuarii TYGS 11278
Kandeliimicrobium roseum KT886061
Oceaniglobus indicus TYGS 11288
Roseivivax roseus TYGS 2650
Tranquillimonas alkanivorans AB302386
Profundibacterium mesophilum JF776971
Maribius pontilimi LT797154
Pseudomaribius aestuariivivens MG322243
Palleronia soli KP064190
Palleronia abyssalis TYGS 19127
Palleronia marisminoris TYGS 2274
Maribius salinus TYGS 2317
Maribius pelagius TYGS 2296
Hwanghaeicola aestuarii FJ230842
Oceanicola litoreus TYGS 6203
Tropicimonas arenosa KU719510
Psychromarinibacter halotolerans KU321207
Rhodosalinus sediminis KX815123
Confluentimicrobium naphthalenivorans KP272155
Confluentimicrobium lipolyticum TYGS 11268
Actibacterium pelagium TYGS 19015
Actibacterium mucosum TYGS 3871
Actibacterium atlanticum TYGS 3863
Actibacterium ureilyticum TYGS 23455
Rhodovulum bhavnagarense FR828479
Rhodovulum kholense TYGS 22931
Rhodovulum viride TYGS 11419
Rhodovulum visakhapatnamense AM180707
Rhodovulum algae LN908891
Rhodovulum sulfidophilum TYGS 5131
Rhodovulum lacipunicei AM921780
Rhodovulum euryhalinum TYGS 20640
Rhodovulum strictum D16419
Rhodovulum steppense EU741680
Rhodovulum tesquicola EU741685
Rhodovulum salis HE680093
Rhodovulum marinum TYGS 20668
Rhodovulum phaeolacus FN669139
Rhodovulum iodosum Y15011

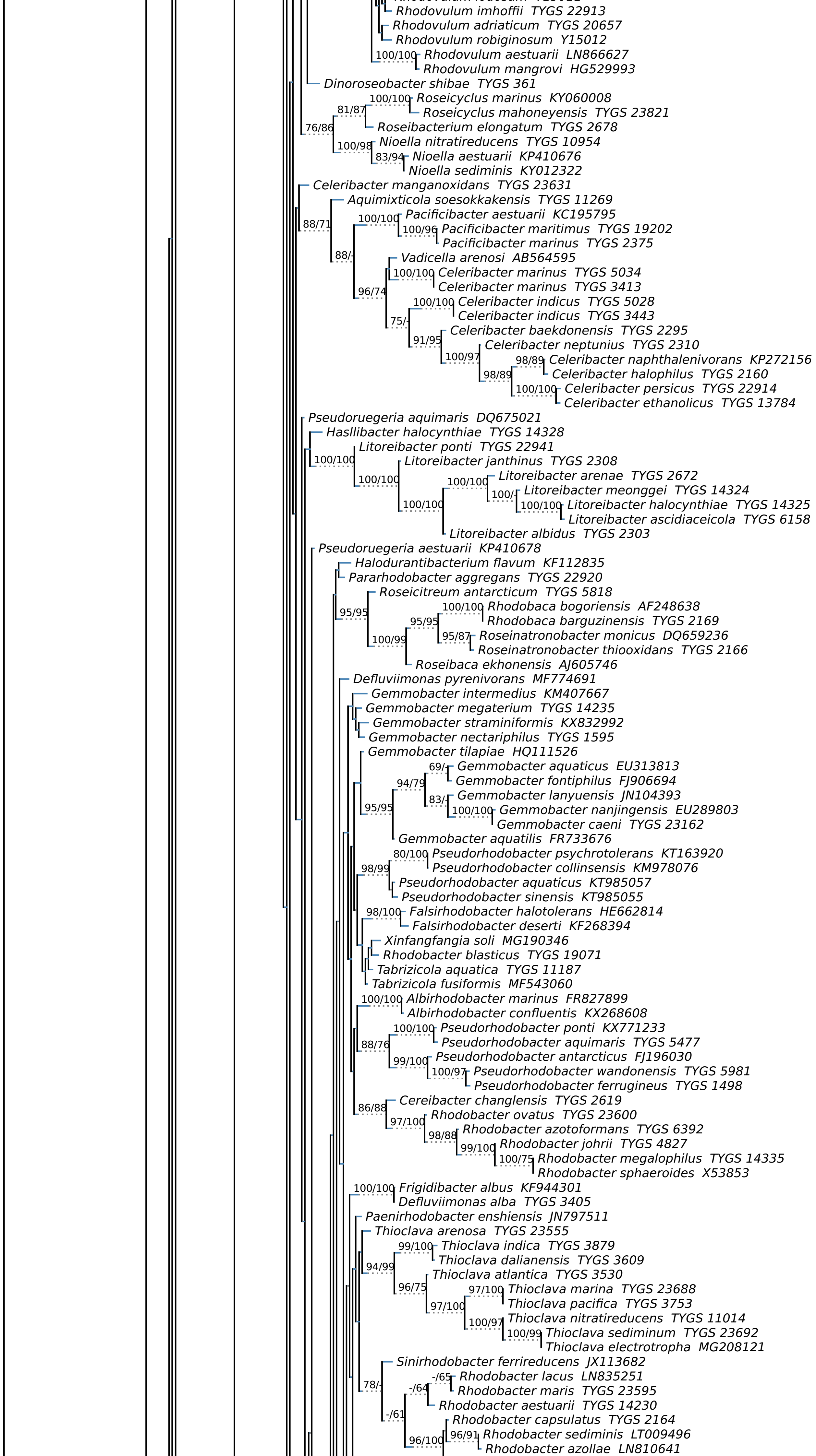
Rhodovulum imhoffii TYGS 22913
Rhodovulum adriaticum TYGS 20657
Rhodovulum robiginosum Y15012
Rhodovulum aestuarii LN866627
Rhodovulum mangrovi HG529993
Dinoroseobacter shibae TYGS 361
Roseicyclus marinus KY060008
Roseicyclus mahoneyensis TYGS 23821
Roseibacterium elongatum TYGS 2678
Nioella nitratireducens TYGS 10954
Nioella aestuarii KP410676
Nioella sediminis KY012322
Celeribacter manganoxidans TYGS 23631
Aquimixticola soesokkakensis TYGS 11269
Pacificibacter aestuarii KC195795
Pacificibacter maritimus TYGS 19202
Pacificibacter marinus TYGS 2375
Vadicella arenosi AB564595
Celeribacter marinus TYGS 5034
Celeribacter marinus TYGS 3413
Celeribacter indicus TYGS 5028
Celeribacter indicus TYGS 3443
Celeribacter baekdonensis TYGS 2295
Celeribacter neptunius TYGS 2310
Celeribacter naphthalenivorans KP272156
Celeribacter halophilus TYGS 2160
Celeribacter persicus TYGS 22914
Celeribacter ethanolicus TYGS 13784
Pseudoruegeria aquimaris DQ675021
Hasllibacter halocynthiae TYGS 14328
Litoreibacter ponti TYGS 22941
Litoreibacter janthinus TYGS 2308
Litoreibacter arenae TYGS 2672
Litoreibacter meonggei TYGS 14324
Litoreibacter halocynthiae TYGS 14325
Litoreibacter ascidiaceicola TYGS 6158
Litoreibacter albidus TYGS 2303
Pseudoruegeria aestuarii KP410678
Halodurantibacterium flavum KF112835
Pararhodobacter aggregans TYGS 22920
Roseicitreum antarcticum TYGS 5818
Rhodobaca bogoriensis AF248638
Rhodobaca barguzinensis TYGS 2169
Roseinatronobacter monicus DQ659236
Roseinatronobacter thiooxidans TYGS 2166
Roseibaca ekhonensis AJ605746
Defluviimonas pyrenivorans MF774691
Gemmobacter intermedius KM407667
Gemmobacter megaterium TYGS 14235
Gemmobacter straminiformis KX832992
Gemmobacter nectariphilus TYGS 1595
Gemmobacter tilapiae HQ111526
Gemmobacter aquaticus EU313813
Gemmobacter fontiphilus FJ906694
Gemmobacter lanyuensis JN104393
Gemmobacter nanjingensis EU289803
Gemmobacter caeni TYGS 23162
Gemmobacter aquatilis FR733676
Pseudorhodobacter psychrotolerans KT163920
Pseudorhodobacter collinsensis KM978076
Pseudorhodobacter aquaticus KT985057
Pseudorhodobacter sinensis KT985055
Falsirhodobacter halotolerans HE662814
Falsirhodobacter deserti KF268394
Xinfangfangia soli MG190346
Rhodobacter blasticus TYGS 19071
Tabrizicola aquatica TYGS 11187
Tabrizicola fusiformis MF543060
Albirhodobacter marinus FR827899
Albirhodobacter confluentis KX268608
Pseudorhodobacter ponti KX771233
Pseudorhodobacter aquimaris TYGS 5477
Pseudorhodobacter antarcticus FJ196030
Pseudorhodobacter wandonensis TYGS 5981
Pseudorhodobacter ferrugineus TYGS 1498
Cereibacter changlensis TYGS 2619
Rhodobacter ovatus TYGS 23600
Rhodobacter azotoformans TYGS 6392
Rhodobacter johrii TYGS 4827
Rhodobacter megalophilus TYGS 14335
Rhodobacter sphaeroides X53853
Frigidibacter albus KF944301
Defluviimonas alba TYGS 3405
Paenirhodobacter enshiensis JN797511
Thioclava arenosa TYGS 23555
Thioclava indica TYGS 3879
Thioclava dalianensis TYGS 3609
Thioclava atlantica TYGS 3530
Thioclava marina TYGS 23688
Thioclava pacifica TYGS 3753
Thioclava nitratireducens TYGS 11014
Thioclava sediminum TYGS 23692
Thioclava electrotropha MG208121
Sinirhodobacter ferrireducens JX113682
Rhodobacter lacus LN835251
Rhodobacter maris TYGS 23595
Rhodobacter aestuarii TYGS 14230
Rhodobacter capsulatus TYGS 2164
Rhodobacter sediminis LT009496
Rhodobacter azollae LN810641

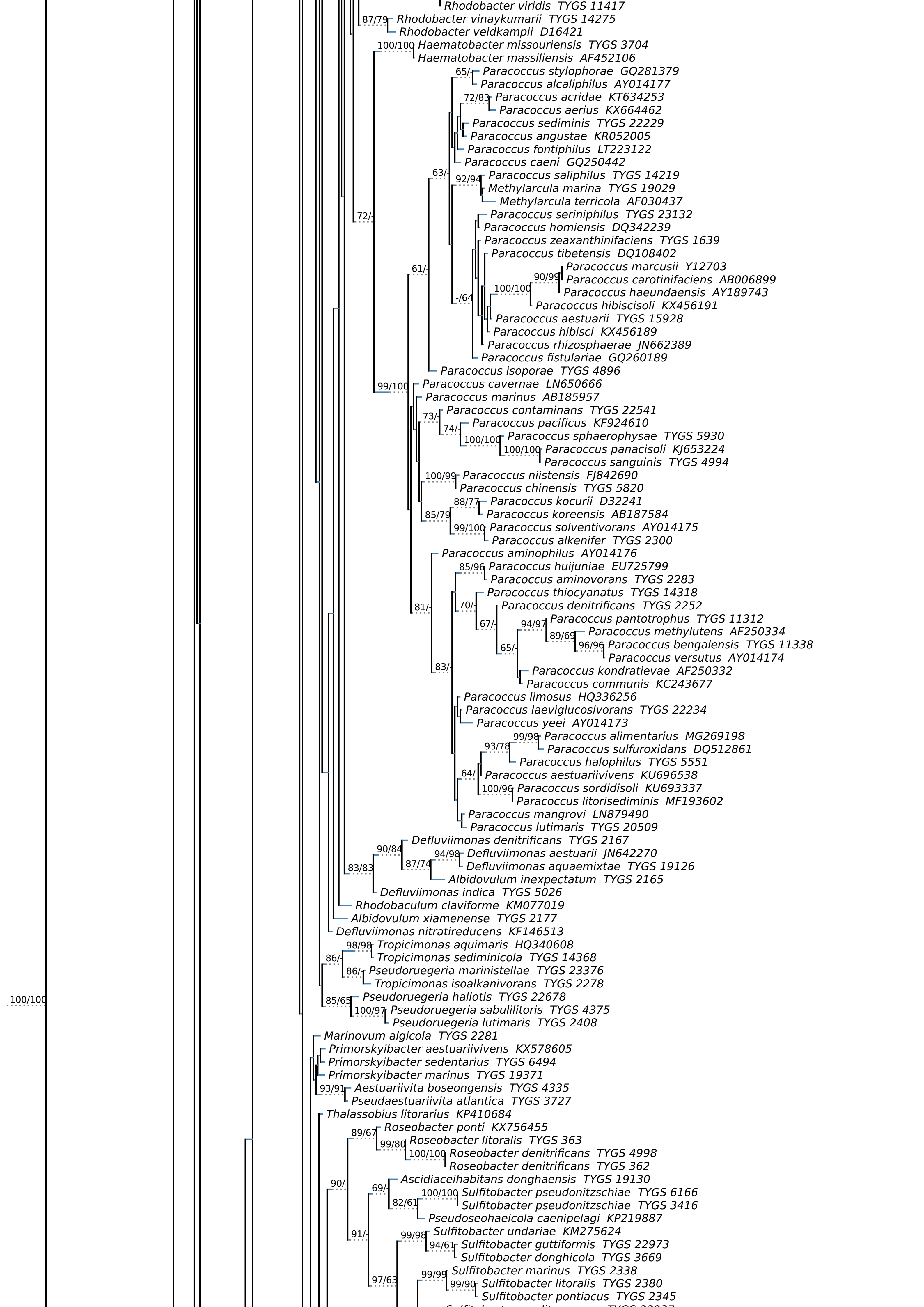
Rhodobacter viridis TYGS 11417
Rhodobacter vinaykumarii TYGS 14275
Rhodobacter veldkampii D16421
Haematobacter missouriensis TYGS 3704
Haematobacter massiliensis AF452106
Paracoccus stylophorae GQ281379
Paracoccus alcaliphilus AY014177
Paracoccus acridae KT634253
Paracoccus aerius KX664462
Paracoccus sediminis TYGS 22229
Paracoccus angustae KR052005
Paracoccus fontiphilus LT223122
Paracoccus caeni GQ250442
Paracoccus saliphilus TYGS 14219
Methylarcula marina TYGS 19029
Methylarcula terricola AF030437
Paracoccus seriniphilus TYGS 23132
Paracoccus homiensis DQ342239
Paracoccus zeaxanthinifaciens TYGS 1639
Paracoccus tibetensis DQ108402
Paracoccus marcusii Y12703
Paracoccus carotinifaciens AB006899
Paracoccus haeundaensis AY189743
Paracoccus hibiscisoli KX456191
Paracoccus aestuarii TYGS 15928
Paracoccus hibisci KX456189
Paracoccus rhizosphaerae JN662389
Paracoccus fistulariae GQ260189
Paracoccus isoporae TYGS 4896
Paracoccus cavernae LN650666
Paracoccus marinus AB185957
Paracoccus contaminans TYGS 22541
Paracoccus pacificus KF924610
Paracoccus sphaerophysae TYGS 5930
Paracoccus panacisoli KJ653224
Paracoccus sanguinis TYGS 4994
Paracoccus niistensis FJ842690
Paracoccus chinensis TYGS 5820
Paracoccus kocurii D32241
Paracoccus koreensis AB187584
Paracoccus solventivorans AY014175
Paracoccus alkenifer TYGS 2300
Paracoccus aminophilus AY014176
Paracoccus huijuniae EU725799
Paracoccus aminovorans TYGS 2283
Paracoccus thiocyanatus TYGS 14318
Paracoccus denitrificans TYGS 2252
Paracoccus pantotrophus TYGS 11312
Paracoccus methylutens AF250334
Paracoccus bengalensis TYGS 11338
Paracoccus versutus AY014174
Paracoccus kondratievae AF250332
Paracoccus communis KC243677
Paracoccus limosus HQ336256
Paracoccus laeviglucosivorans TYGS 22234
Paracoccus yeei AY014173
Paracoccus alimentarius MG269198
Paracoccus sulfuroxidans DQ512861
Paracoccus halophilus TYGS 5551
Paracoccus aestuariivivens KU696538
Paracoccus sordidisoli KU693337
Paracoccus litorisediminis MF193602
Paracoccus mangrovi LN879490
Paracoccus lutimaris TYGS 20509
Defluviimonas denitrificans TYGS 2167
Defluviimonas aestuarii JN642270
Defluviimonas aquaemixtae TYGS 19126
Albidovulum inexpectatum TYGS 2165
Defluviimonas indica TYGS 5026
Rhodobaculum claviforme KM077019
Albidovulum xiamenense TYGS 2177
Defluviimonas nitratireducens KF146513
Tropicimonas aquimaris HQ340608
Tropicimonas sediminicola TYGS 14368
Pseudoruegeria marinistellae TYGS 23376
Tropicimonas isoalkanivorans TYGS 2278
Pseudoruegeria haliotis TYGS 22678
Pseudoruegeria sabulilitoris TYGS 4375
Pseudoruegeria lutimaris TYGS 2408
Marinovum algicola TYGS 2281
Primorskyibacter aestuariivivens KX578605
Primorskyibacter sedentarius TYGS 6494
Primorskyibacter marinus TYGS 19371
Aestuariivita boseongensis TYGS 4335
Pseudaestuariivita atlantica TYGS 3727
Thalassobius litorarius KP410684
Roseobacter ponti KX756455
Roseobacter litoralis TYGS 363
Roseobacter denitrificans TYGS 4998
Roseobacter denitrificans TYGS 362
Ascidiaceihabitans donghaensis TYGS 19130
Sulfitobacter pseudonitzschiae TYGS 6166
Sulfitobacter pseudonitzschiae TYGS 3416
Pseudoseohaeicola caenipelagi KP219887
Sulfitobacter undariae KM275624
Sulfitobacter guttiformis TYGS 22973
Sulfitobacter donghicola TYGS 3669
Sulfitobacter marinus TYGS 2338
Sulfitobacter litoralis TYGS 2380
Sulfitobacter pontiacus TYGS 2345

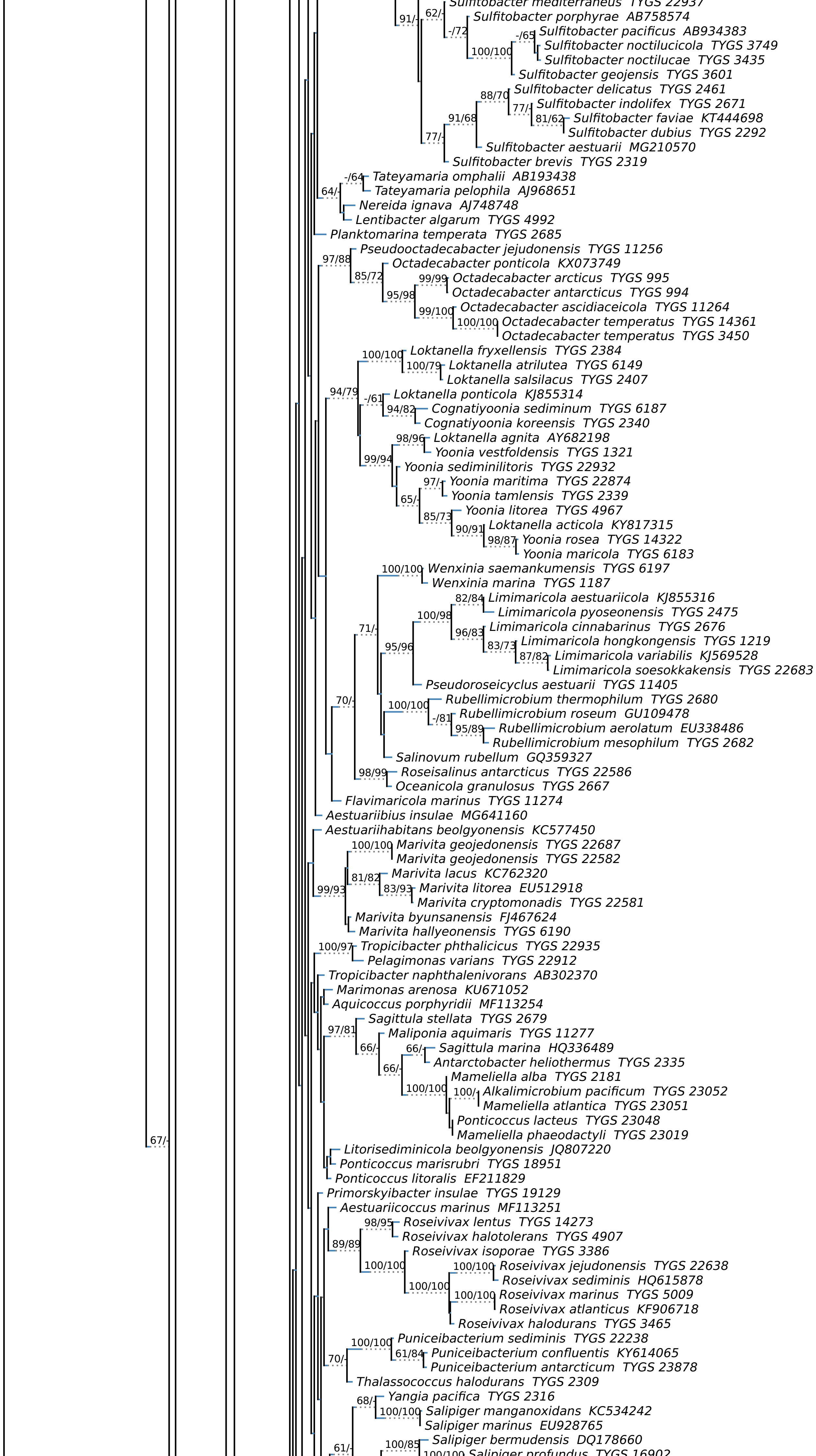
Sulfitobacter mediterraneus TYGS 22937
Sulfitobacter porphyrae AB758574
Sulfitobacter pacificus AB934383
Sulfitobacter noctilucicola TYGS 3749
Sulfitobacter noctilucae TYGS 3435
Sulfitobacter geojensis TYGS 3601
Sulfitobacter delicatus TYGS 2461
Sulfitobacter indolifex TYGS 2671
Sulfitobacter faviae KT444698
Sulfitobacter dubius TYGS 2292
Sulfitobacter aestuarii MG210570
Sulfitobacter brevis TYGS 2319
Tateyamaria omphalii AB193438
Tateyamaria pelophila AJ968651
Nereida ignava AJ748748
Lentibacter algarum TYGS 4992
Planktomarina temperata TYGS 2685
Pseudooctadecabacter jejudonensis TYGS 11256
Octadecabacter ponticola KX073749
Octadecabacter arcticus TYGS 995
Octadecabacter antarcticus TYGS 994
Octadecabacter ascidiaceicola TYGS 11264
Octadecabacter temperatus TYGS 14361
Octadecabacter temperatus TYGS 3450
Loktanella fryxellensis TYGS 2384
Loktanella atrilutea TYGS 6149
Loktanella salsilacus TYGS 2407
Loktanella ponticola KJ855314
Cognatiyoonia sediminum TYGS 6187
Cognatiyoonia koreensis TYGS 2340
Loktanella agnita AY682198
Yoonia vestfoldensis TYGS 1321
Yoonia sediminilitoris TYGS 22932
Yoonia maritima TYGS 22874
Yoonia tamlensis TYGS 2339
Yoonia litorea TYGS 4967
Loktanella acticola KY817315
Yoonia rosea TYGS 14322
Yoonia maricola TYGS 6183
Wenxinia saemankumensis TYGS 6197
Wenxinia marina TYGS 1187
Limimaricola aestuariicola KJ855316
Limimaricola pyoseonensis TYGS 2475
Limimaricola cinnabarinus TYGS 2676
Limimaricola hongkongensis TYGS 1219
Limimaricola variabilis KJ569528
Limimaricola soesokkakensis TYGS 22683
Pseudoroseicyclus aestuarii TYGS 11405
Rubellimicrobium thermophilum TYGS 2680
Rubellimicrobium roseum GU109478
Rubellimicrobium aerolatum EU338486
Rubellimicrobium mesophilum TYGS 2682
Salinovum rubellum GQ359327
Roseisalinus antarcticus TYGS 22586
Oceanicola granulosus TYGS 2667
Flavimaricola marinus TYGS 11274
Aestuariibius insulae MG641160
Aestuariihabitans beolgyonensis KC577450
Marivita geojedonensis TYGS 22687
Marivita geojedonensis TYGS 22582
Marivita lacus KC762320
Marivita litorea EU512918
Marivita cryptomonadis TYGS 22581
Marivita byunsanensis FJ467624
Marivita hallyeonensis TYGS 6190
Tropicibacter phthalicicus TYGS 22935
Pelagimonas varians TYGS 22912
Tropicibacter naphthalenivorans AB302370
Marimonas arenosa KU671052
Aquicoccus porphyridii MF113254
Sagittula stellata TYGS 2679
Maliponia aquimaris TYGS 11277
Sagittula marina HQ336489
Antarctobacter heliothermus TYGS 2335
Mameliella alba TYGS 2181
Alkalimicrobium pacificum TYGS 23052
Mameliella atlantica TYGS 23051
Ponticoccus lacteus TYGS 23048
Mameliella phaeodactyli TYGS 23019
Litorisediminicola beolgyonensis JQ807220
Ponticoccus marisrubri TYGS 18951
Ponticoccus litoralis EF211829
Primorskyibacter insulae TYGS 19129
Aestuariicoccus marinus MF113251
Roseivivax lentus TYGS 14273
Roseivivax halotolerans TYGS 4907
Roseivivax isoporae TYGS 3386
Roseivivax jejudonensis TYGS 22638
Roseivivax sediminis HQ615878
Roseivivax marinus TYGS 5009
Roseivivax atlanticus KF906718
Roseivivax halodurans TYGS 3465
Puniceibacterium sediminis TYGS 22238
Puniceibacterium confluentis KY614065
Puniceibacterium antarcticum TYGS 23878
Thalassococcus halodurans TYGS 2309
Yangia pacifica TYGS 2316
Salipiger manganoxidans KC534242
Salipiger marinus EU928765
Salipiger bermudensis DQ178660
Salipiger profundus TYGS 16902

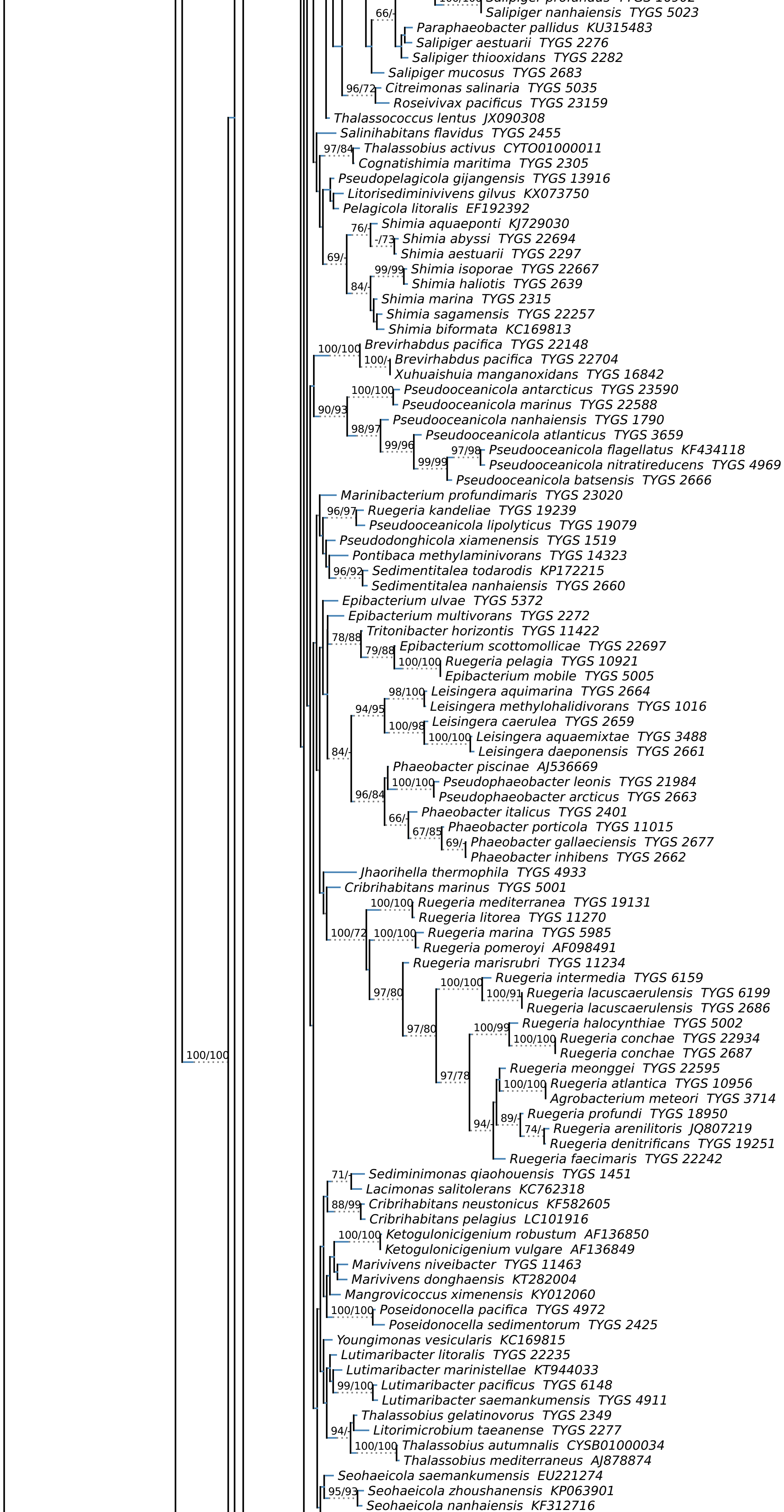

66/-
Salipiger nanhaiensis TYGS 5023
Paraphaeobacter pallidus KU315483
Salipiger aestuarii TYGS 2276
Salipiger thiooxidans TYGS 2282
Salipiger mucosus TYGS 2683
96/72 Citreimonas salinaria TYGS 5035
Roseivivax pacificus TYGS 23159
Thalassococcus lentus JX090308
Salinihabitans flavidus TYGS 2455
97/84 Thalassobius activus CYTO01000011
Cognatishimia maritima TYGS 2305
Pseudopelagicola gijangensis TYGS 13916
Litorisediminivivens gilvus KX073750
Pelagicola litoralis EF192392
76/- Shimia aquaeponti KJ729030
-/73 Shimia abyssi TYGS 22694
69/- Shimia aestuarii TYGS 2297
99/99 Shimia isoporae TYGS 22667
84/- Shimia haliotis TYGS 2639
Shimia marina TYGS 2315
Shimia sagamensis TYGS 22257
Shimia biformata KC169813
100/100 Brevirhabdus pacifica TYGS 22148
100/- Brevirhabdus pacifica TYGS 22704
Xuhuaishuia manganoxidans TYGS 16842
100/100 Pseudooceanicola antarcticus TYGS 23590
90/93 Pseudooceanicola marinus TYGS 22588
98/97 Pseudooceanicola nanhaiensis TYGS 1790
99/96 Pseudooceanicola atlanticus TYGS 3659
97/98 Pseudooceanicola flagellatus KF434118
99/99 Pseudooceanicola nitratireducens TYGS 4969
Pseudooceanicola batsensis TYGS 2666
Marinibacterium profundimaris TYGS 23020
96/97 Ruegeria kandeliae TYGS 19239
Pseudooceanicola lipolyticus TYGS 19079
Pseudodonghicola xiamenensis TYGS 1519
Pontibaca methylaminivorans TYGS 14323
96/92 Sedimentitalea todarodis KP172215
Sedimentitalea nanhaiensis TYGS 2660
Epibacterium ulvae TYGS 5372
Epibacterium multivorans TYGS 2272
78/88 Tritonibacter horizontis TYGS 11422
79/88 Epibacterium scottomollicae TYGS 22697
100/100 Ruegeria pelagia TYGS 10921
Epibacterium mobile TYGS 5005
98/100 Leisingera aquimarina TYGS 2664
94/95 Leisingera methylohalidivorans TYGS 1016
100/98 Leisingera caerulea TYGS 2659
100/100 Leisingera aquaemixtae TYGS 3488
84/- Leisingera daeponensis TYGS 2661
Phaeobacter piscinae AJ536669
100/100 Pseudophaeobacter leonis TYGS 21984
96/84 Pseudophaeobacter arcticus TYGS 2663
66/- Phaeobacter italicus TYGS 2401
67/85 Phaeobacter porticola TYGS 11015
69/- Phaeobacter gallaeciensis TYGS 2677
Phaeobacter inhibens TYGS 2662
Jhaorihella thermophila TYGS 4933
Cribrihabitans marinus TYGS 5001
100/100 Ruegeria mediterranea TYGS 19131
Ruegeria litorea TYGS 11270
100/72 100/100 Ruegeria marina TYGS 5985
Ruegeria pomeroyi AF098491
Ruegeria marisrubri TYGS 11234
100/100 Ruegeria intermedia TYGS 6159
97/80 100/91 Ruegeria lacuscaerulensis TYGS 6199
Ruegeria lacuscaerulensis TYGS 2686
97/80 100/99 Ruegeria halocynthiae TYGS 5002
100/100 Ruegeria conchae TYGS 22934
100/100 Ruegeria conchae TYGS 2687
Ruegeria meonggei TYGS 22595
97/78 100/100 Ruegeria atlantica TYGS 10956
Agrobacterium meteori TYGS 3714
89/- Ruegeria profundi TYGS 18950
94/- 74/- Ruegeria arenilitoris JQ807219
Ruegeria denitrificans TYGS 19251
Ruegeria faecimaris TYGS 22242
71/- Sediminimonas qiaohouensis TYGS 1451
Lacimonas salitolerans KC762318
88/99 Cribrihabitans neustonicus KF582605
Cribrihabitans pelagius LC101916
100/100 Ketogulonicigenium robustum AF136850
Ketogulonicigenium vulgare AF136849
Marivivens niveibacter TYGS 11463
Marivivens donghaensis KT282004
Mangrovicoccus ximenensis KY012060
100/100 Poseidonocella pacifica TYGS 4972
Poseidonocella sedimentorum TYGS 2425
Youngimonas vesicularis KC169815
Lutimaribacter litoralis TYGS 22235
Lutimaribacter marinistellae KT944033
99/100 Lutimaribacter pacificus TYGS 6148
Lutimaribacter saemankumensis TYGS 4911
Thalassobius gelatinovorus TYGS 2349
94/- Litorimicrobium taeanense TYGS 2277
100/100 Thalassobius autumnalis CYSB01000034
Thalassobius mediterraneus AJ878874
Seohaeicola saemankumensis EU221274
95/93 Seohaeicola zhoushanensis KP063901
Seohaeicola nanhaiensis KF312716

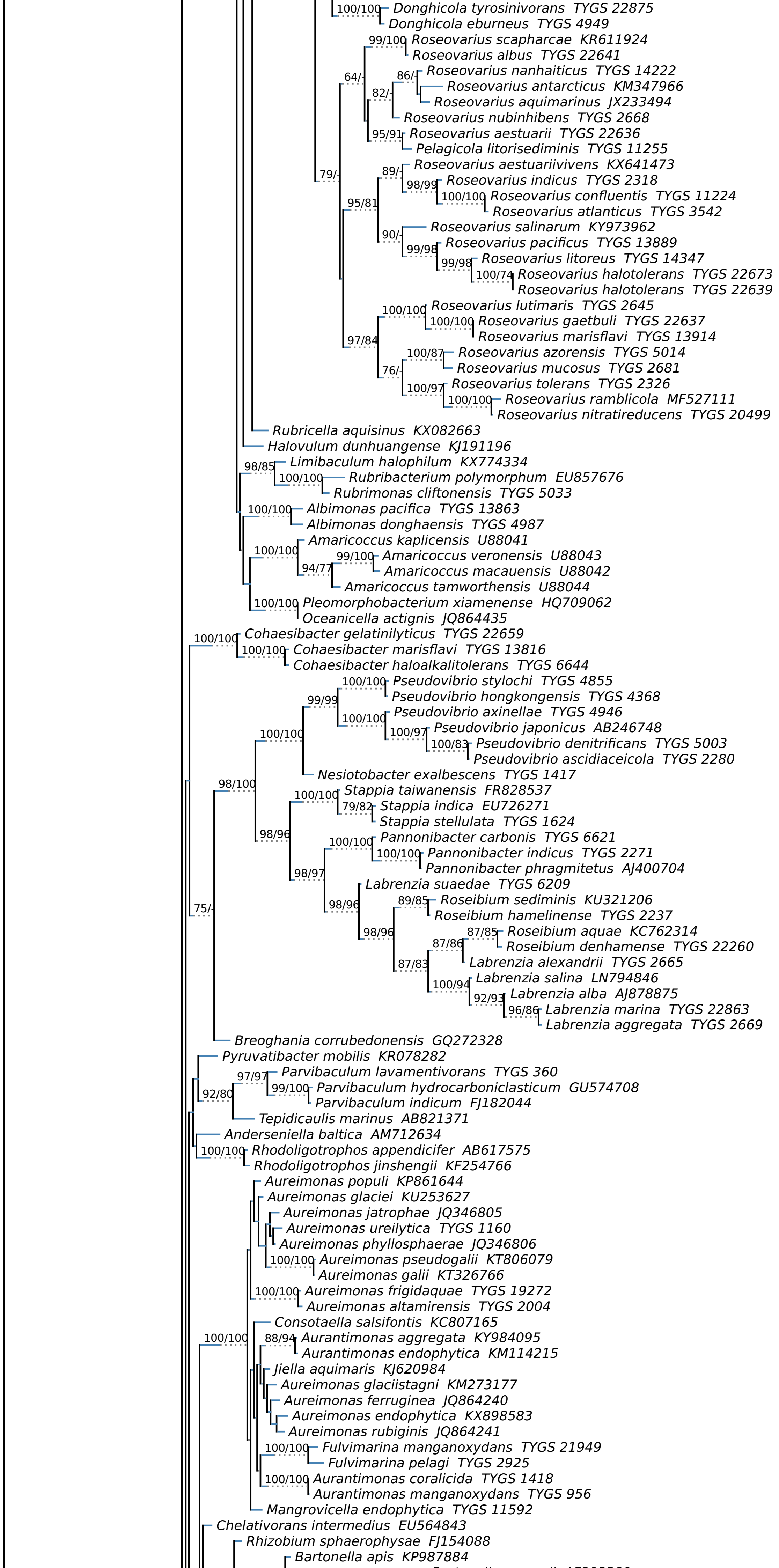
Donghicola tyrosinivorans TYGS 22875
Donghicola eburneus TYGS 4949
Roseovarius scapharcae KR611924
Roseovarius albus TYGS 22641
Roseovarius nanhaiticus TYGS 14222
Roseovarius antarcticus KM347966
Roseovarius aquimarinus JX233494
Roseovarius nubinhibens TYGS 2668
Roseovarius aestuarii TYGS 22636
Pelagicola litorisediminis TYGS 11255
Roseovarius aestuariivivens KX641473
Roseovarius indicus TYGS 2318
Roseovarius confluentis TYGS 11224
Roseovarius atlanticus TYGS 3542
Roseovarius salinarum KY973962
Roseovarius pacificus TYGS 13889
Roseovarius litoreus TYGS 14347
Roseovarius halotolerans TYGS 22673
Roseovarius halotolerans TYGS 22639
Roseovarius lutimaris TYGS 2645
Roseovarius gaetbuli TYGS 22637
Roseovarius marisflavi TYGS 13914
Roseovarius azorensis TYGS 5014
Roseovarius mucosus TYGS 2681
Roseovarius tolerans TYGS 2326
Roseovarius ramblicola MF527111
Roseovarius nitratireducens TYGS 20499
Rubricella aquisinus KX082663
Halovulum dunhuangense KJ191196
Limibaculum halophilum KX774334
Rubribacterium polymorphum EU857676
Rubrimonas cliftonensis TYGS 5033
Albimonas pacifica TYGS 13863
Albimonas donghaensis TYGS 4987
Amaricoccus kaplicensis U88041
Amaricoccus veronensis U88043
Amaricoccus macauensis U88042
Amaricoccus tamworthensis U88044
Pleomorphobacterium xiamenense HQ709062
Oceanicella actignis JQ864435
Cohaesibacter gelatinilyticus TYGS 22659
Cohaesibacter marisflavi TYGS 13816
Cohaesibacter haloalkalitolerans TYGS 6644
Pseudovibrio stylochi TYGS 4855
Pseudovibrio hongkongensis TYGS 4368
Pseudovibrio axinellae TYGS 4946
Pseudovibrio japonicus AB246748
Pseudovibrio denitrificans TYGS 5003
Pseudovibrio ascidiaceicola TYGS 2280
Nesiotobacter exalbescens TYGS 1417
Stappia taiwanensis FR828537
Stappia indica EU726271
Stappia stellulata TYGS 1624
Pannonibacter carbonis TYGS 6621
Pannonibacter indicus TYGS 2271
Pannonibacter phragmitetus AJ400704
Labrenzia suaedae TYGS 6209
Roseibium sediminis KU321206
Roseibium hamelinense TYGS 2237
Roseibium aquae KC762314
Roseibium denhamense TYGS 22260
Labrenzia alexandrii TYGS 2665
Labrenzia salina LN794846
Labrenzia alba AJ878875
Labrenzia marina TYGS 22863
Labrenzia aggregata TYGS 2669
Breoghania corrubedonensis GQ272328
Pyruvatibacter mobilis KR078282
Parvibaculum lavamentivorans TYGS 360
Parvibaculum hydrocarboniclasticum GU574708
Parvibaculum indicum FJ182044
Tepidicaulis marinus AB821371
Anderseniella baltica AM712634
Rhodoligotrophos appendicifer AB617575
Rhodoligotrophos jinshengii KF254766
Aureimonas populi KP861644
Aureimonas glaciei KU253627
Aureimonas jatrophae JQ346805
Aureimonas ureilytica TYGS 1160
Aureimonas phyllosphaerae JQ346806
Aureimonas pseudogalii KT806079
Aureimonas galii KT326766
Aureimonas frigidaquae TYGS 19272
Aureimonas altamirensis TYGS 2004
Consotaella salsifontis KC807165
Aurantimonas aggregata KY984095
Aurantimonas endophytica KM114215
Jiella aquimaris KJ620984
Aureimonas glaciistagni KM273177
Aureimonas ferruginea JQ864240
Aureimonas endophytica KX898583
Aureimonas rubiginis JQ864241
Fulvimarina manganoxydans TYGS 21949
Fulvimarina pelagi TYGS 2925
Aurantimonas coralicida TYGS 1418
Aurantimonas manganoxydans TYGS 956
Mangrovicella endophytica TYGS 11592
Chelativorans intermedius EU564843
Rhizobium sphaerophysae FJ154088
Bartonella apis KP987884

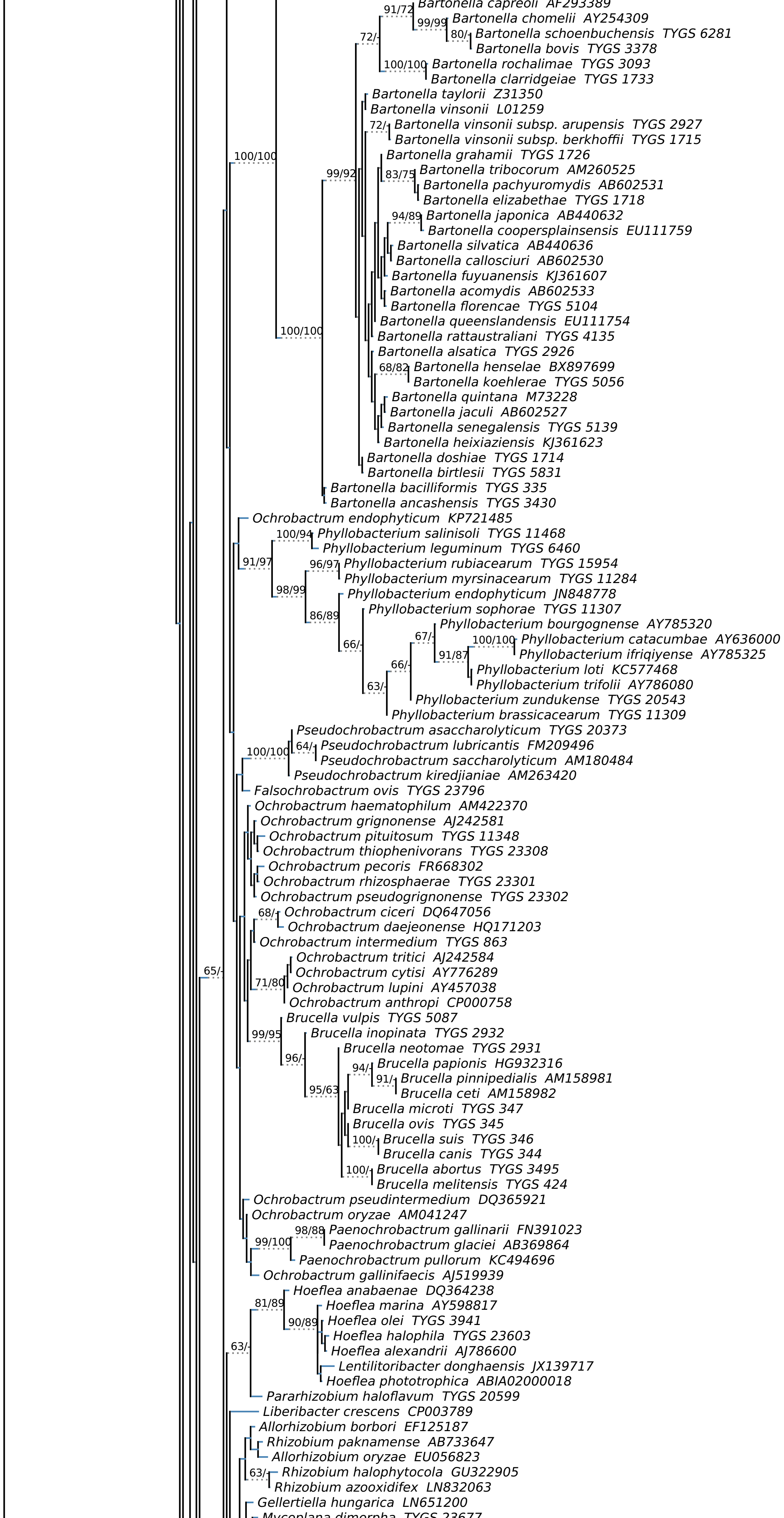
Bartonella capreoli AF293389
Bartonella chomelii AY254309
Bartonella schoenbuchensis TYGS 6281
Bartonella bovis TYGS 3378
Bartonella rochalimae TYGS 3093
Bartonella clarridgeiae TYGS 1733
Bartonella taylorii Z31350
Bartonella vinsonii L01259
Bartonella vinsonii subsp. arupensis TYGS 2927
Bartonella vinsonii subsp. berkhoffii TYGS 1715
Bartonella grahamii TYGS 1726
Bartonella tribocorum AM260525
Bartonella pachyuromydis AB602531
Bartonella elizabethae TYGS 1718
Bartonella japonica AB440632
Bartonella coopersplainsensis EU111759
Bartonella silvatica AB440636
Bartonella callosciuri AB602530
Bartonella fuyuanensis KJ361607
Bartonella acomydis AB602533
Bartonella florencae TYGS 5104
Bartonella queenslandensis EU111754
Bartonella rattaustraliani TYGS 4135
Bartonella alsatica TYGS 2926
Bartonella henselae BX897699
Bartonella koehlerae TYGS 5056
Bartonella quintana M73228
Bartonella jaculi AB602527
Bartonella senegalensis TYGS 5139
Bartonella heixiaziensis KJ361623
Bartonella doshiae TYGS 1714
Bartonella birtlesii TYGS 5831
Bartonella bacilliformis TYGS 335
Bartonella ancashensis TYGS 3430
Ochrobactrum endophyticum KP721485
Phyllobacterium salinisoli TYGS 11468
Phyllobacterium leguminum TYGS 6460
Phyllobacterium rubiacearum TYGS 15954
Phyllobacterium myrsinacearum TYGS 11284
Phyllobacterium endophyticum JN848778
Phyllobacterium sophorae TYGS 11307
Phyllobacterium bourgognense AY785320
Phyllobacterium catacumbae AY636000
Phyllobacterium ifriqiyense AY785325
Phyllobacterium loti KC577468
Phyllobacterium trifolii AY786080
Phyllobacterium zundukense TYGS 20543
Phyllobacterium brassicacearum TYGS 11309
Pseudochrobactrum asaccharolyticum TYGS 20373
Pseudochrobactrum lubricantis FM209496
Pseudochrobactrum saccharolyticum AM180484
Pseudochrobactrum kiredjianiae AM263420
Falsochrobactrum ovis TYGS 23796
Ochrobactrum haematophilum AM422370
Ochrobactrum grignonense AJ242581
Ochrobactrum pituitosum TYGS 11348
Ochrobactrum thiophenivorans TYGS 23308
Ochrobactrum pecoris FR668302
Ochrobactrum rhizosphaerae TYGS 23301
Ochrobactrum pseudogrignonense TYGS 23302
Ochrobactrum ciceri DQ647056
Ochrobactrum daejeonense HQ171203
Ochrobactrum intermedium TYGS 863
Ochrobactrum tritici AJ242584
Ochrobactrum cytisi AY776289
Ochrobactrum lupini AY457038
Ochrobactrum anthropi CP000758
Brucella vulpis TYGS 5087
Brucella inopinata TYGS 2932
Brucella neotomae TYGS 2931
Brucella papionis HG932316
Brucella pinnipedialis AM158981
Brucella ceti AM158982
Brucella microti TYGS 347
Brucella ovis TYGS 345
Brucella suis TYGS 346
Brucella canis TYGS 344
Brucella abortus TYGS 3495
Brucella melitensis TYGS 424
Ochrobactrum pseudintermedium DQ365921
Ochrobactrum oryzae AM041247
Paenochrobactrum gallinarii FN391023
Paenochrobactrum glaciei AB369864
Paenochrobactrum pullorum KC494696
Ochrobactrum gallinifaecis AJ519939
Hoeflea anabaenae DQ364238
Hoeflea marina AY598817
Hoeflea olei TYGS 3941
Hoeflea halophila TYGS 23603
Hoeflea alexandrii AJ786600
Lentilitoribacter donghaensis JX139717
Hoeflea phototrophica ABIA02000018
Pararhizobium haloflavum TYGS 20599
Liberibacter crescens CP003789
Allorhizobium borbori EF125187
Rhizobium paknamense AB733647
Allorhizobium oryzae EU056823
Rhizobium halophytocola GU322905
Rhizobium azooxidifex LN832063
Gellertiella hungarica LN651200
Mycoplana dimorpha TYGS 23677

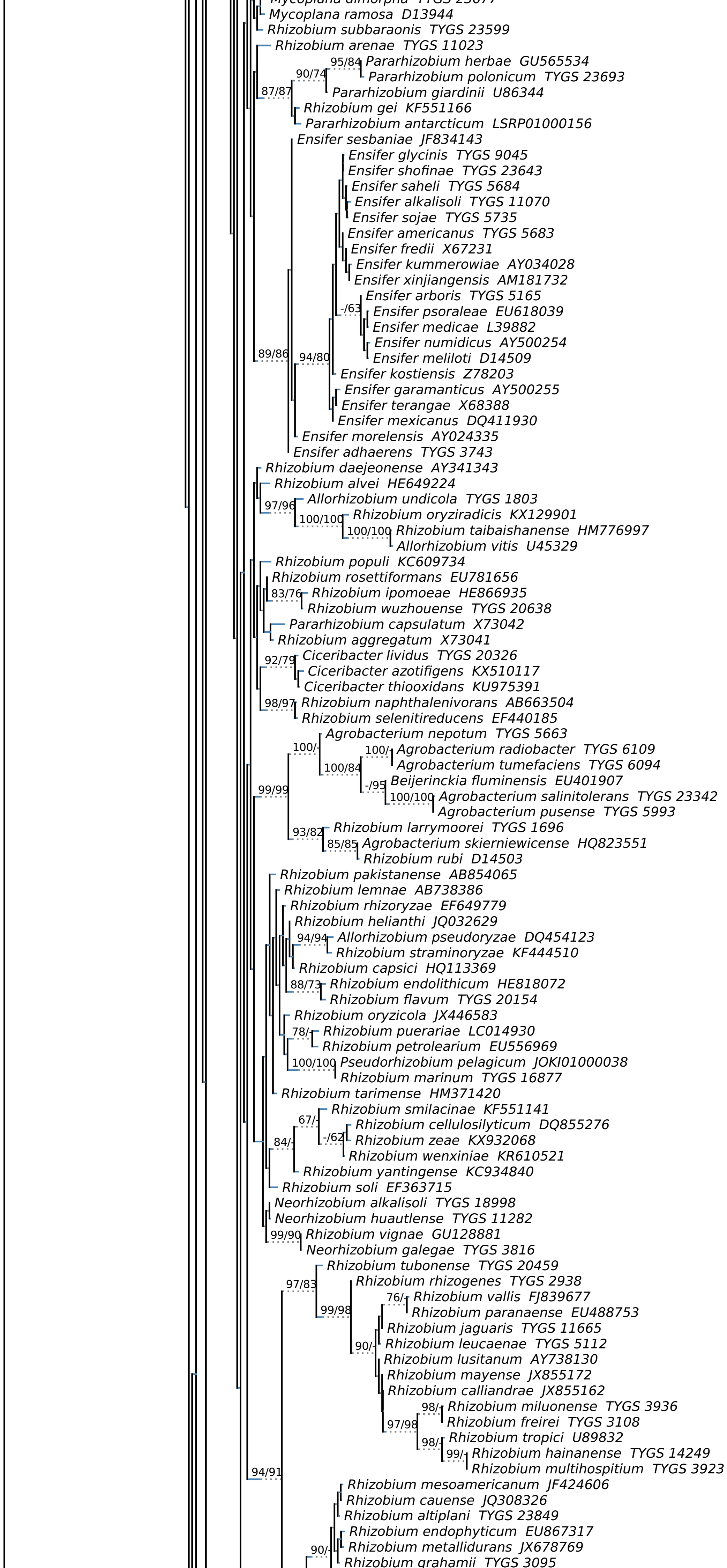
Mycoplana ramosa D13944
Rhizobium subbaraonis TYGS 23599
Rhizobium arenae TYGS 11023
95/84
Pararhizobium herbae GU565534
90/74
Pararhizobium polonicum TYGS 23693
87/87
Pararhizobium giardinii U86344
Rhizobium gei KF551166
Pararhizobium antarcticum LSRP01000156
Ensifer sesbaniae JF834143
Ensifer glycinis TYGS 9045
Ensifer shofinae TYGS 23643
Ensifer saheli TYGS 5684
Ensifer alkalisoli TYGS 11070
Ensifer sojae TYGS 5735
Ensifer americanus TYGS 5683
Ensifer fredii X67231
Ensifer kummerowiae AY034028
Ensifer xinjiangensis AM181732
Ensifer arboris TYGS 5165
-/63
Ensifer psoraleae EU618039
Ensifer medicae L39882
Ensifer numidicus AY500254
89/86
94/80
Ensifer meliloti D14509
Ensifer kostiensis Z78203
Ensifer garamanticus AY500255
Ensifer terangae X68388
Ensifer mexicanus DQ411930
Ensifer morelensis AY024335
Ensifer adhaerens TYGS 3743
Rhizobium daejeonense AY341343
Rhizobium alvei HE649224
Allorhizobium undicola TYGS 1803
97/96
Rhizobium oryziradicis KX129901
100/100
100/100
Rhizobium taibaishanense HM776997
Allorhizobium vitis U45329
Rhizobium populi KC609734
Rhizobium rosettiformans EU781656
83/76
Rhizobium ipomoeae HE866935
Rhizobium wuzhouense TYGS 20638
Pararhizobium capsulatum X73042
Rhizobium aggregatum X73041
92/79
Ciceribacter lividus TYGS 20326
Ciceribacter azotifigens KX510117
Ciceribacter thiooxidans KU975391
98/97
Rhizobium naphthalenivorans AB663504
Rhizobium selenitireducens EF440185
Agrobacterium nepotum TYGS 5663
100/-
100/-
Agrobacterium radiobacter TYGS 6109
100/84
Agrobacterium tumefaciens TYGS 6094
Beijerinckia fluminensis EU401907
-/95
99/99
100/100
Agrobacterium salinitolerans TYGS 23342
Agrobacterium pusense TYGS 5993
93/82
Rhizobium larrymoorei TYGS 1696
85/85
Agrobacterium skierniewicense HQ823551
Rhizobium rubi D14503
Rhizobium pakistanense AB854065
Rhizobium lemnae AB738386
Rhizobium rhizoryzae EF649779
Rhizobium helianthi JQ032629
94/94
Allorhizobium pseudoryzae DQ454123
Rhizobium straminoryzae KF444510
Rhizobium capsici HQ113369
88/73
Rhizobium endolithicum HE818072
Rhizobium flavum TYGS 20154
Rhizobium oryzicola JX446583
78/-
Rhizobium puerariae LC014930
Rhizobium petrolearium EU556969
100/100
Pseudorhizobium pelagicum JOKI01000038
Rhizobium marinum TYGS 16877
Rhizobium tarimense HM371420
Rhizobium smilacinae KF551141
67/-
Rhizobium cellulosilyticum DQ855276
84/-
-/62
Rhizobium zeae KX932068
Rhizobium wenxiniae KR610521
Rhizobium yantingense KC934840
Rhizobium soli EF363715
Neorhizobium alkalisoli TYGS 18998
Neorhizobium huautlense TYGS 11282
99/90
Rhizobium vignae GU128881
Neorhizobium galegae TYGS 3816
Rhizobium tubonense TYGS 20459
97/83
Rhizobium rhizogenes TYGS 2938
76/-
Rhizobium vallis FJ839677
99/98
Rhizobium paranaense EU488753
Rhizobium jaguaris TYGS 11665
90/-
Rhizobium leucaenae TYGS 5112
Rhizobium lusitanum AY738130
Rhizobium mayense JX855172
Rhizobium calliandrae JX855162
98/-
Rhizobium miluonense TYGS 3936
97/98
Rhizobium freirei TYGS 3108
98/-
Rhizobium tropici U89832
99/-
Rhizobium hainanense TYGS 14249
94/91
Rhizobium multihospitium TYGS 3923
Rhizobium mesoamericanum JF424606
Rhizobium cauense JQ308326
Rhizobium altiplani TYGS 23849
Rhizobium endophyticum EU867317
90/-
Rhizobium metallidurans JX678769
Rhizobium grahamii TYGS 3095

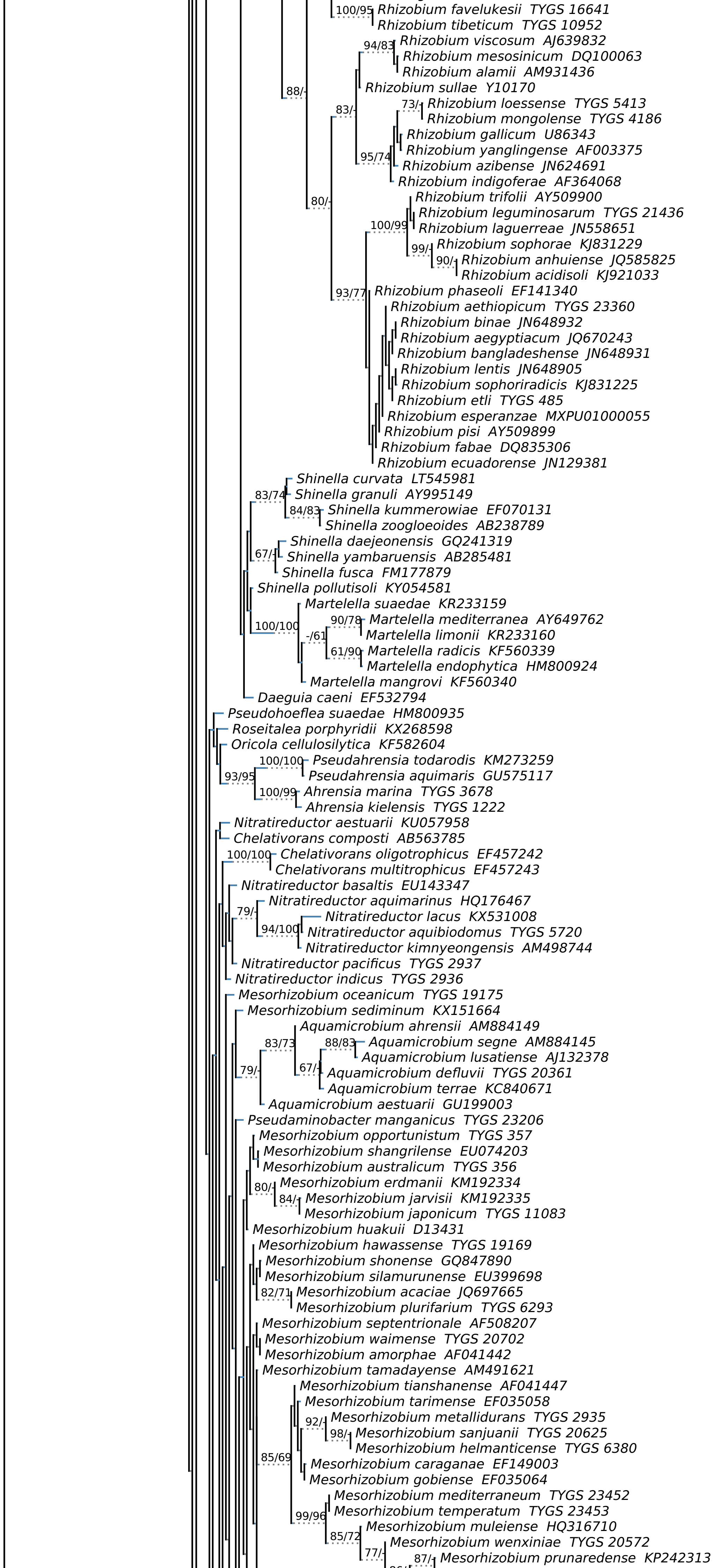

Rhizobium favelukesii TYGS 16641
Rhizobium tibeticum TYGS 10952
Rhizobium viscosum AJ639832
Rhizobium mesosinicum DQ100063
Rhizobium alamii AM931436
Rhizobium sullae Y10170
Rhizobium loessense TYGS 5413
Rhizobium mongolense TYGS 4186
Rhizobium gallicum U86343
Rhizobium yanglingense AF003375
Rhizobium azibense JN624691
Rhizobium indigoferae AF364068
Rhizobium trifolii AY509900
Rhizobium leguminosarum TYGS 21436
Rhizobium laguerreae JN558651
Rhizobium sophorae KJ831229
Rhizobium anhuiense JQ585825
Rhizobium acidisoli KJ921033
Rhizobium phaseoli EF141340
Rhizobium aethiopicum TYGS 23360
Rhizobium binae JN648932
Rhizobium aegyptiacum JQ670243
Rhizobium bangladeshense JN648931
Rhizobium lentis JN648905
Rhizobium sophoriradicis KJ831225
Rhizobium etli TYGS 485
Rhizobium esperanzae MXPU01000055
Rhizobium pisi AY509899
Rhizobium fabae DQ835306
Rhizobium ecuadorense JN129381
Shinella curvata LT545981
Shinella granuli AY995149
Shinella kummerowiae EF070131
Shinella zoogloeoides AB238789
Shinella daejeonensis GQ241319
Shinella yambaruensis AB285481
Shinella fusca FM177879
Shinella pollutisoli KY054581
Martelella suaedae KR233159
Martelella mediterranea AY649762
Martelella limonii KR233160
Martelella radicis KF560339
Martelella endophytica HM800924
Martelella mangrovi KF560340
Daeguia caeni EF532794
Pseudohoeflea suaedae HM800935
Roseitalea porphyridii KX268598
Oricola cellulosilytica KF582604
Pseudahrensia todarodis KM273259
Pseudahrensia aquimaris GU575117
Ahrensia marina TYGS 3678
Ahrensia kielensis TYGS 1222
Nitratireductor aestuarii KU057958
Chelativorans composti AB563785
Chelativorans oligotrophicus EF457242
Chelativorans multitrophicus EF457243
Nitratireductor basaltis EU143347
Nitratireductor aquimarinus HQ176467
Nitratireductor lacus KX531008
Nitratireductor aquibiodomus TYGS 5720
Nitratireductor kimnyeongensis AM498744
Nitratireductor pacificus TYGS 2937
Nitratireductor indicus TYGS 2936
Mesorhizobium oceanicum TYGS 19175
Mesorhizobium sediminum KX151664
Aquamicrobium ahrensii AM884149
Aquamicrobium segne AM884145
Aquamicrobium lusatiense AJ132378
Aquamicrobium defluvii TYGS 20361
Aquamicrobium terrae KC840671
Aquamicrobium aestuarii GU199003
Pseudaminobacter manganicus TYGS 23206
Mesorhizobium opportunistum TYGS 357
Mesorhizobium shangrilense EU074203
Mesorhizobium australicum TYGS 356
Mesorhizobium erdmanii KM192334
Mesorhizobium jarvisii KM192335
Mesorhizobium japonicum TYGS 11083
Mesorhizobium huakuii D13431
Mesorhizobium hawassense TYGS 19169
Mesorhizobium shonense GQ847890
Mesorhizobium silamurunense EU399698
Mesorhizobium acaciae JQ697665
Mesorhizobium plurifarium TYGS 6293
Mesorhizobium septentrionale AF508207
Mesorhizobium waimense TYGS 20702
Mesorhizobium amorphae AF041442
Mesorhizobium tamadayense AM491621
Mesorhizobium tianshanense AF041447
Mesorhizobium tarimense EF035058
Mesorhizobium metallidurans TYGS 2935
Mesorhizobium sanjuanii TYGS 20625
Mesorhizobium helmanticense TYGS 6380
Mesorhizobium caraganae EF149003
Mesorhizobium gobiense EF035064
Mesorhizobium mediterraneum TYGS 23452
Mesorhizobium temperatum TYGS 23453
Mesorhizobium muleiense HQ316710
Mesorhizobium wenxiniae TYGS 20572
Mesorhizobium prunaredense KP242313

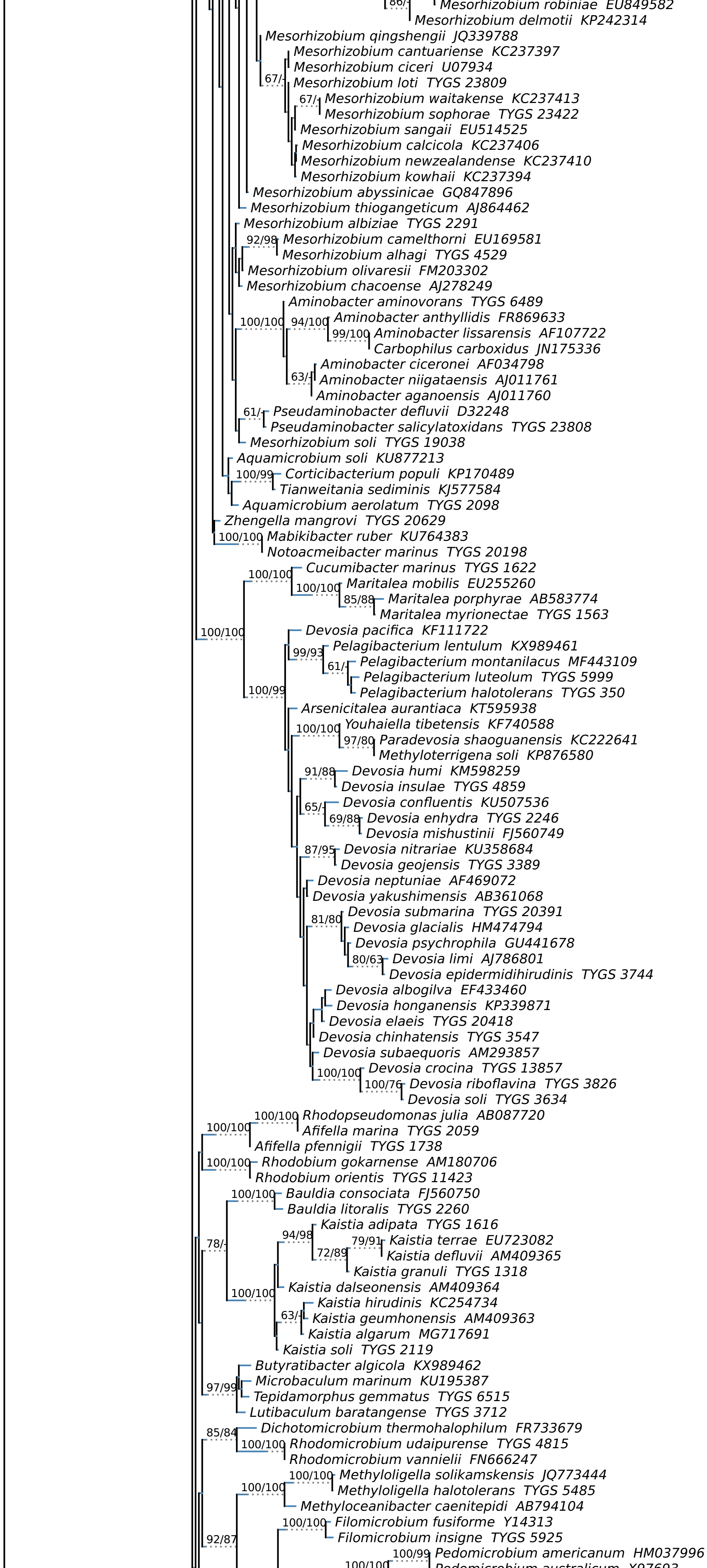

Mesorhizobium robiniae EU849582
Mesorhizobium delmotii KP242314
Mesorhizobium qingshengii JQ339788
Mesorhizobium cantuariense KC237397
Mesorhizobium ciceri U07934
Mesorhizobium loti TYGS 23809
Mesorhizobium waitakense KC237413
Mesorhizobium sophorae TYGS 23422
Mesorhizobium sangaii EU514525
Mesorhizobium calcicola KC237406
Mesorhizobium newzealandense KC237410
Mesorhizobium kowhaii KC237394
Mesorhizobium abyssinicae GQ847896
Mesorhizobium thiogangeticum AJ864462
Mesorhizobium albiziae TYGS 2291
Mesorhizobium camelthorni EU169581
Mesorhizobium alhagi TYGS 4529
Mesorhizobium olivaresii FM203302
Mesorhizobium chacoense AJ278249
Aminobacter aminovorans TYGS 6489
Aminobacter anthyllidis FR869633
Aminobacter lissarensis AF107722
Carbophilus carboxidus JN175336
Aminobacter ciceronei AF034798
Aminobacter niigataensis AJ011761
Aminobacter aganoensis AJ011760
Pseudaminobacter defluvii D32248
Pseudaminobacter salicylatoxidans TYGS 23808
Mesorhizobium soli TYGS 19038
Aquamicrobium soli KU877213
Corticibacterium populi KP170489
Tianweitania sediminis KJ577584
Aquamicrobium aerolatum TYGS 2098
Zhengella mangrovi TYGS 20629
Mabikibacter ruber KU764383
Notoacmeibacter marinus TYGS 20198
Cucumibacter marinus TYGS 1622
Maritalea mobilis EU255260
Maritalea porphyrae AB583774
Maritalea myrionectae TYGS 1563
Devosia pacifica KF111722
Pelagibacterium lentulum KX989461
Pelagibacterium montanilacus MF443109
Pelagibacterium luteolum TYGS 5999
Pelagibacterium halotolerans TYGS 350
Arsenicitalea aurantiaca KT595938
Youhaiella tibetensis KF740588
Paradevosia shaoguanensis KC222641
Methyloterrigena soli KP876580
Devosia humi KM598259
Devosia insulae TYGS 4859
Devosia confluentis KU507536
Devosia enhydra TYGS 2246
Devosia mishustinii FJ560749
Devosia nitrariae KU358684
Devosia geojensis TYGS 3389
Devosia neptuniae AF469072
Devosia yakushimensis AB361068
Devosia submarina TYGS 20391
Devosia glacialis HM474794
Devosia psychrophila GU441678
Devosia limi AJ786801
Devosia epidermidihirudinis TYGS 3744
Devosia albogilva EF433460
Devosia honganensis KP339871
Devosia elaeis TYGS 20418
Devosia chinhatensis TYGS 3547
Devosia subaequoris AM293857
Devosia crocina TYGS 13857
Devosia riboflavina TYGS 3826
Devosia soli TYGS 3634
Rhodopseudomonas julia AB087720
Afifella marina TYGS 2059
Afifella pfennigii TYGS 1738
Rhodobium gokarnense AM180706
Rhodobium orientis TYGS 11423
Bauldia consociata FJ560750
Bauldia litoralis TYGS 2260
Kaistia adipata TYGS 1616
Kaistia terrae EU723082
Kaistia defluvii AM409365
Kaistia granuli TYGS 1318
Kaistia dalseonensis AM409364
Kaistia hirudinis KC254734
Kaistia geumhonensis AM409363
Kaistia algarum MG717691
Kaistia soli TYGS 2119
Butyratibacter algicola KX989462
Microbaculum marinum KU195387
Tepidamorphus gemmatus TYGS 6515
Lutibaculum baratangense TYGS 3712
Dichotomicrobium thermohalophilum FR733679
Rhodomicrobium udaipurense TYGS 4815
Rhodomicrobium vannielii FN666247
Methyloligella solikamskensis JQ773444
Methyloligella halotolerans TYGS 5485
Methyloceanibacter caenitepidi AB794104
Filomicrobium fusiforme Y14313
Filomicrobium insigne TYGS 5925
Pedomicrobium americanum HM037996

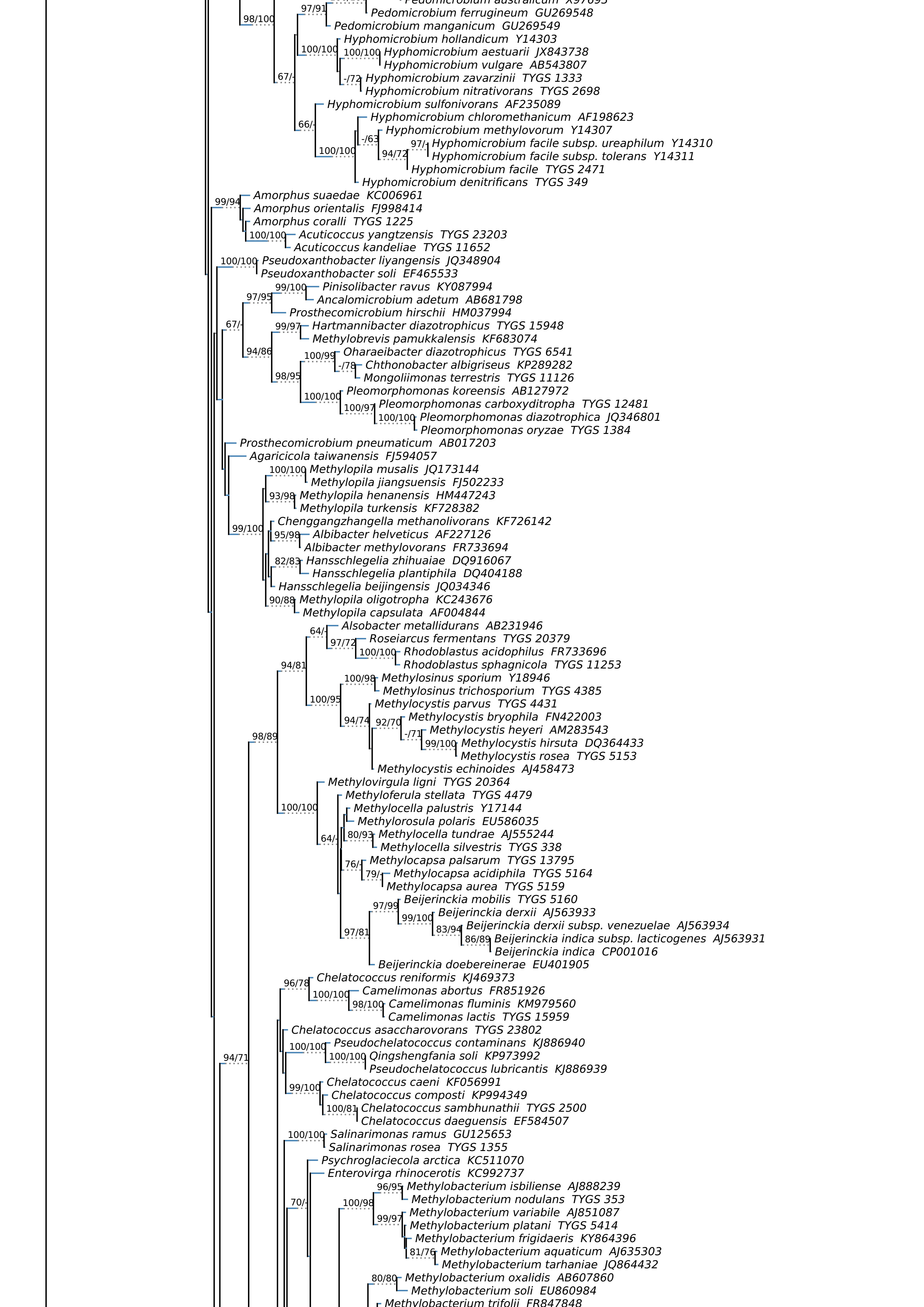
Pedomicrobium ferrugineum GU269548
Pedomicrobium manganicum GU269549
Hyphomicrobium hollandicum Y14303
Hyphomicrobium aestuarii JX843738
Hyphomicrobium vulgare AB543807
Hyphomicrobium zavarzinii TYGS 1333
Hyphomicrobium nitrativorans TYGS 2698
Hyphomicrobium sulfonivorans AF235089
Hyphomicrobium chloromethanicum AF198623
Hyphomicrobium methylovorum Y14307
Hyphomicrobium facile subsp. ureaphilum Y14310
Hyphomicrobium facile subsp. tolerans Y14311
Hyphomicrobium facile TYGS 2471
Hyphomicrobium denitrificans TYGS 349
Amorphus suaedae KC006961
Amorphus orientalis FJ998414
Amorphus coralli TYGS 1225
Acuticoccus yangtzensis TYGS 23203
Acuticoccus kandeliae TYGS 11652
Pseudoxanthobacter liyangensis JQ348904
Pseudoxanthobacter soli EF465533
Pinisolibacter ravus KY087994
Ancalomicrobium adetum AB681798
Prosthecomicrobium hirschii HM037994
Hartmannibacter diazotrophicus TYGS 15948
Methylobrevis pamukkalensis KF683074
Oharaeibacter diazotrophicus TYGS 6541
Chthonobacter albigriseus KP289282
Mongoliimonas terrestris TYGS 11126
Pleomorphomonas koreensis AB127972
Pleomorphomonas carboxyditropha TYGS 12481
Pleomorphomonas diazotrophica JQ346801
Pleomorphomonas oryzae TYGS 1384
Prosthecomicrobium pneumaticum AB017203
Agaricicola taiwanensis FJ594057
Methylopila musalis JQ173144
Methylopila jiangsuensis FJ502233
Methylopila henanensis HM447243
Methylopila turkensis KF728382
Chenggangzhangella methanolivorans KF726142
Albibacter helveticus AF227126
Albibacter methylovorans FR733694
Hansschlegelia zhihuaiae DQ916067
Hansschlegelia plantiphila DQ404188
Hansschlegelia beijingensis JQ034346
Methylopila oligotropha KC243676
Methylopila capsulata AF004844
Alsobacter metallidurans AB231946
Roseiarcus fermentans TYGS 20379
Rhodoblastus acidophilus FR733696
Rhodoblastus sphagnicola TYGS 11253
Methylosinus sporium Y18946
Methylosinus trichosporium TYGS 4385
Methylocystis parvus TYGS 4431
Methylocystis bryophila FN422003
Methylocystis heyeri AM283543
Methylocystis hirsuta DQ364433
Methylocystis rosea TYGS 5153
Methylocystis echinoides AJ458473
Methylovirgula ligni TYGS 20364
Methyloferula stellata TYGS 4479
Methylocella palustris Y17144
Methylorosula polaris EU586035
Methylocella tundrae AJ555244
Methylocella silvestris TYGS 338
Methylocapsa palsarum TYGS 13795
Methylocapsa acidiphila TYGS 5164
Methylocapsa aurea TYGS 5159
Beijerinckia mobilis TYGS 5160
Beijerinckia derxii AJ563933
Beijerinckia derxii subsp. venezuelae AJ563934
Beijerinckia indica subsp. lacticogenes AJ563931
Beijerinckia indica CP001016
Beijerinckia doebereinerae EU401905
Chelatococcus reniformis KJ469373
Camelimonas abortus FR851926
Camelimonas fluminis KM979560
Camelimonas lactis TYGS 15959
Chelatococcus asaccharovorans TYGS 23802
Pseudochelatococcus contaminans KJ886940
Qingshengfania soli KP973992
Pseudochelatococcus lubricantis KJ886939
Chelatococcus caeni KF056991
Chelatococcus composti KP994349
Chelatococcus sambhunathii TYGS 2500
Chelatococcus daeguensis EF584507
Salinarimonas ramus GU125653
Salinarimonas rosea TYGS 1355
Psychroglaciecola arctica KC511070
Enterovirga rhinocerotis KC992737
Methylobacterium isbiliense AJ888239
Methylobacterium nodulans TYGS 353
Methylobacterium variabile AJ851087
Methylobacterium platani TYGS 5414
Methylobacterium frigidaeris KY864396
Methylobacterium aquaticum AJ635303
Methylobacterium tarhaniae JQ864432
Methylobacterium oxalidis AB607860
Methylobacterium soli EU860984
Methylobacterium trifolii FR847848

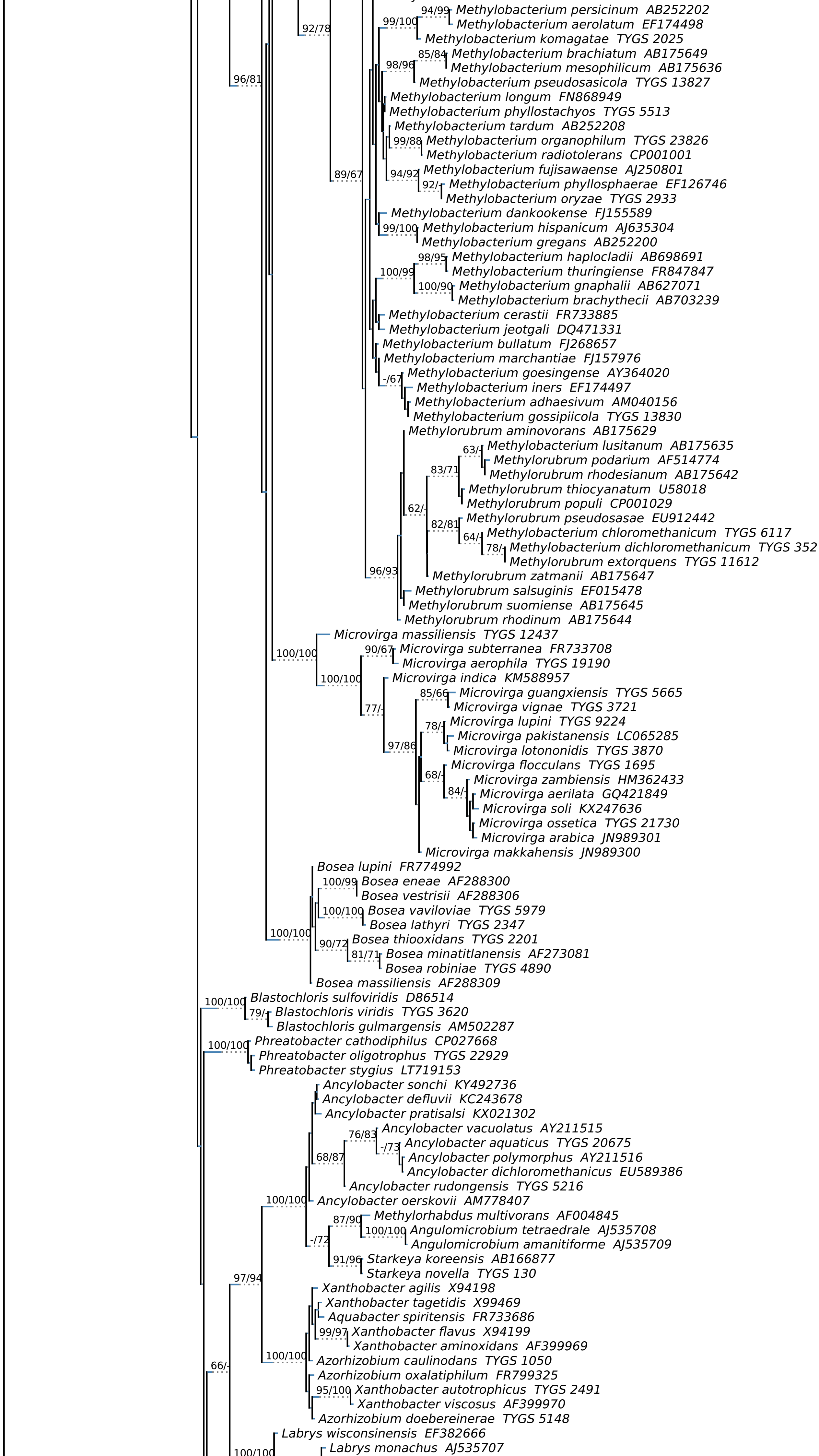
94/99
Methylobacterium persicinum AB252202
Methylobacterium aerolatum EF174498
99/100
92/78
Methylobacterium komagatae TYGS 2025
85/84
Methylobacterium brachiatum AB175649
98/96
Methylobacterium mesophilicum AB175636
96/81
Methylobacterium pseudosasicola TYGS 13827
Methylobacterium longum FN868949
Methylobacterium phyllostachyos TYGS 5513
Methylobacterium tardum AB252208
99/88
Methylobacterium organophilum TYGS 23826
Methylobacterium radiotolerans CP001001
94/92
Methylobacterium fujisawaense AJ250801
89/67
92/-
Methylobacterium phyllosphaerae EF126746
Methylobacterium oryzae TYGS 2933
Methylobacterium dankookense FJ155589
99/100
Methylobacterium hispanicum AJ635304
Methylobacterium gregans AB252200
98/95
Methylobacterium haplocladii AB698691
100/99
Methylobacterium thuringiense FR847847
100/90
Methylobacterium gnaphalii AB627071
Methylobacterium brachythecii AB703239
Methylobacterium cerastii FR733885
Methylobacterium jeotgali DQ471331
Methylobacterium bullatum FJ268657
Methylobacterium marchantiae FJ157976
Methylobacterium goesingense AY364020
-/67
Methylobacterium iners EF174497
Methylobacterium adhaesivum AM040156
Methylobacterium gossipiicola TYGS 13830
Methylorubrum aminovorans AB175629
63/-
Methylobacterium lusitanum AB175635
Methylorubrum podarium AF514774
83/71
Methylorubrum rhodesianum AB175642
Methylorubrum thiocyanatum U58018
62/-
Methylorubrum populi CP001029
Methylorubrum pseudosasae EU912442
82/81
64/-
Methylobacterium chloromethanicum TYGS 6117
78/-
Methylobacterium dichloromethanicum TYGS 352
Methylorubrum extorquens TYGS 11612
96/93
Methylorubrum zatmanii AB175647
Methylorubrum salsuginis EF015478
Methylorubrum suomiense AB175645
Methylorubrum rhodinum AB175644
Microvirga massiliensis TYGS 12437
100/100
90/67
Microvirga subterranea FR733708
Microvirga aerophila TYGS 19190
100/100
Microvirga indica KM588957
85/66
Microvirga guangxiensis TYGS 5665
77/-
Microvirga vignae TYGS 3721
78/-
Microvirga lupini TYGS 9224
Microvirga pakistanensis LC065285
97/86
Microvirga lotononidis TYGS 3870
Microvirga flocculans TYGS 1695
68/-
Microvirga zambiensis HM362433
84/-
Microvirga aerilata GQ421849
Microvirga soli KX247636
Microvirga ossetica TYGS 21730
Microvirga arabica JN989301
Microvirga makkahensis JN989300
Bosea lupini FR774992
100/99
Bosea eneae AF288300
Bosea vestrisii AF288306
100/100
Bosea vaviloviae TYGS 5979
Bosea lathyri TYGS 2347
100/100
Bosea thiooxidans TYGS 2201
90/72
81/71
Bosea minatitlanensis AF273081
Bosea robiniae TYGS 4890
Bosea massiliensis AF288309
100/100
Blastochloris sulfoviridis D86514
79/-
Blastochloris viridis TYGS 3620
Blastochloris gulmargensis AM502287
100/100
Phreatobacter cathodiphilus CP027668
Phreatobacter oligotrophus TYGS 22929
Phreatobacter stygius LT719153
Ancylobacter sonchi KY492736
Ancylobacter defluvii KC243678
Ancylobacter pratisalsi KX021302
76/83
Ancylobacter vacuolatus AY211515
-/73
Ancylobacter aquaticus TYGS 20675
68/87
Ancylobacter polymorphus AY211516
Ancylobacter dichloromethanicus EU589386
Ancylobacter rudongensis TYGS 5216
100/100
Ancylobacter oerskovii AM778407
87/90
Methylorhabdus multivorans AF004845
100/100
Angulomicrobium tetraedrale AJ535708
-/72
Angulomicrobium amanitiforme AJ535709
91/96
Starkeya koreensis AB166877
Starkeya novella TYGS 130
97/94
Xanthobacter agilis X94198
Xanthobacter tagetidis X99469
Aquabacter spiritensis FR733686
99/97
Xanthobacter flavus X94199
Xanthobacter aminoxidans AF399969
100/100
Azorhizobium caulinodans TYGS 1050
66/-
Azorhizobium oxalatiphilum FR799325
95/100
Xanthobacter autotrophicus TYGS 2491
Xanthobacter viscosus AF399970
Azorhizobium doebereinerae TYGS 5148
Labrys wisconsinensis EF382666
100/100
Labrys monachus AJ535707

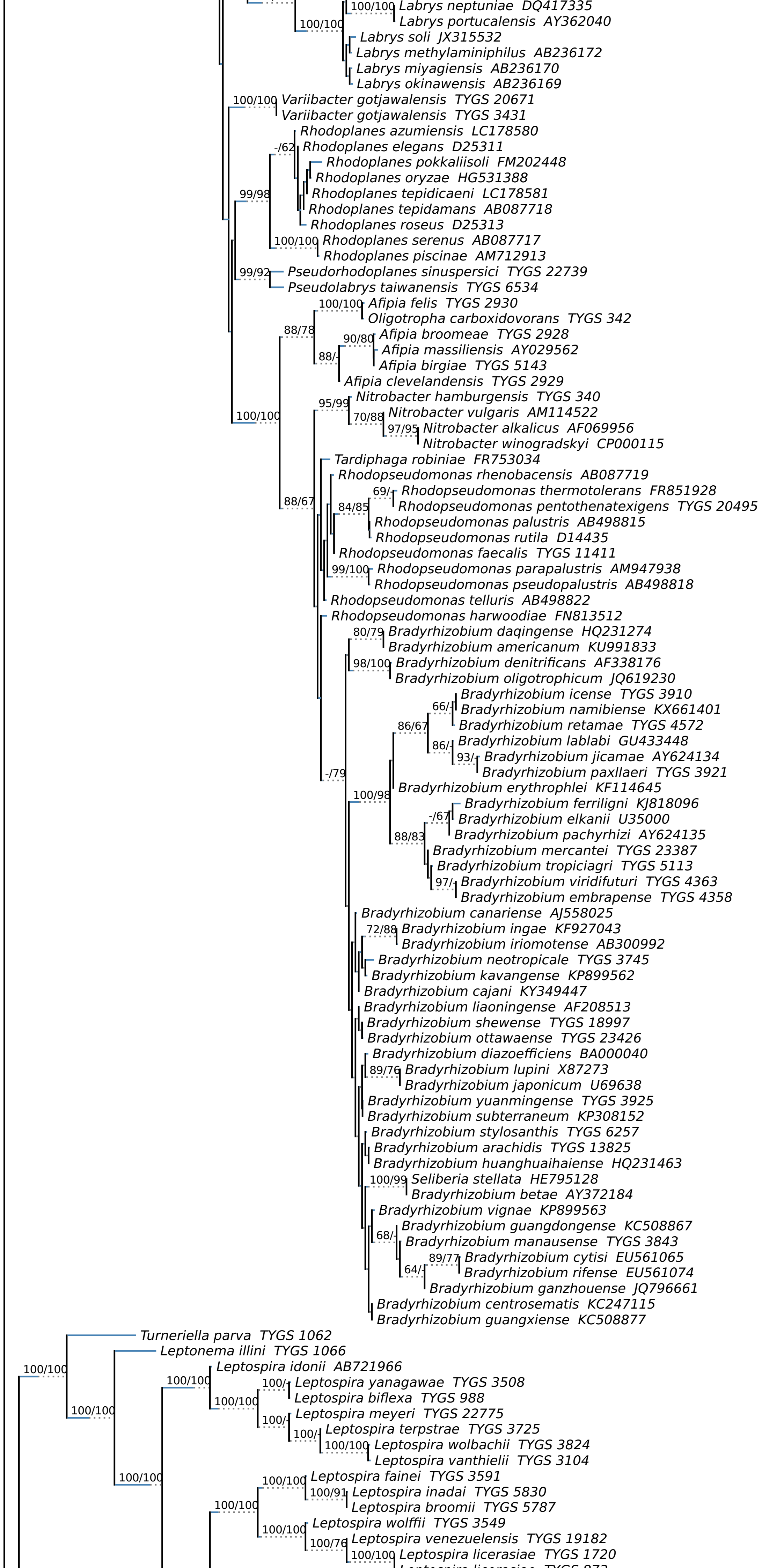
100/100 Labrys neptuniae DQ417335
Labrys portucalensis AY362040
100/100
Labrys soli JX315532
Labrys methylaminiphilus AB236172
Labrys miyagiensis AB236170
Labrys okinawensis AB236169
100/100 Variibacter gotjawalensis TYGS 20671
Variibacter gotjawalensis TYGS 3431
Rhodoplanes azumiensis LC178580
-/62 Rhodoplanes elegans D25311
Rhodoplanes pokkaliisoli FM202448
Rhodoplanes oryzae HG531388
99/98 Rhodoplanes tepidicaeni LC178581
Rhodoplanes tepidamans AB087718
Rhodoplanes roseus D25313
100/100 Rhodoplanes serenus AB087717
Rhodoplanes piscinae AM712913
99/92 Pseudorhodoplanes sinuspersici TYGS 22739
Pseudolabrys taiwanensis TYGS 6534
100/100 Afipia felis TYGS 2930
Oligotropha carboxidovorans TYGS 342
88/78
90/80 Afipia broomeae TYGS 2928
Afipia massiliensis AY029562
88/- Afipia birgiae TYGS 5143
Afipia clevelandensis TYGS 2929
95/99 Nitrobacter hamburgensis TYGS 340
100/100
70/88 Nitrobacter vulgaris AM114522
97/95 Nitrobacter alkalicus AF069956
Nitrobacter winogradskyi CP000115
Tardiphaga robiniae FR753034
Rhodopseudomonas rhenobacensis AB087719
69/- Rhodopseudomonas thermotolerans FR851928
88/67
84/85 Rhodopseudomonas pentothenatexigens TYGS 20495
Rhodopseudomonas palustris AB498815
Rhodopseudomonas rutila D14435
Rhodopseudomonas faecalis TYGS 11411
99/100 Rhodopseudomonas parapalustris AM947938
Rhodopseudomonas pseudopalustris AB498818
Rhodopseudomonas telluris AB498822
Rhodopseudomonas harwoodiae FN813512
80/79 Bradyrhizobium daqingense HQ231274
Bradyrhizobium americanum KU991833
98/100 Bradyrhizobium denitrificans AF338176
Bradyrhizobium oligotrophicum JQ619230
Bradyrhizobium icense TYGS 3910
66/- Bradyrhizobium namibiense KX661401
86/67
Bradyrhizobium retamae TYGS 4572
86/- Bradyrhizobium lablabi GU433448
93/- Bradyrhizobium jicamae AY624134
-/79
Bradyrhizobium paxllaeri TYGS 3921
100/98
Bradyrhizobium erythrophlei KF114645
Bradyrhizobium ferriligni KJ818096
-/67 Bradyrhizobium elkanii U35000
88/83 Bradyrhizobium pachyrhizi AY624135
Bradyrhizobium mercantei TYGS 23387
Bradyrhizobium tropiciagri TYGS 5113
97/- Bradyrhizobium viridifuturi TYGS 4363
Bradyrhizobium embrapense TYGS 4358
Bradyrhizobium canariense AJ558025
72/88 Bradyrhizobium ingae KF927043
Bradyrhizobium iriomotense AB300992
Bradyrhizobium neotropicale TYGS 3745
Bradyrhizobium kavangense KP899562
Bradyrhizobium cajani KY349447
Bradyrhizobium liaoningense AF208513
Bradyrhizobium shewense TYGS 18997
Bradyrhizobium ottawaense TYGS 23426
Bradyrhizobium diazoefficiens BA000040
89/76 Bradyrhizobium lupini X87273
Bradyrhizobium japonicum U69638
Bradyrhizobium yuanmingense TYGS 3925
Bradyrhizobium subterraneum KP308152
Bradyrhizobium stylosanthis TYGS 6257
Bradyrhizobium arachidis TYGS 13825
Bradyrhizobium huanghuaihaiense HQ231463
100/99 Seliberia stellata HE795128
Bradyrhizobium betae AY372184
Bradyrhizobium vignae KP899563
Bradyrhizobium guangdongense KC508867
68/- Bradyrhizobium manausense TYGS 3843
89/77 Bradyrhizobium cytisi EU561065
64/- Bradyrhizobium rifense EU561074
Bradyrhizobium ganzhouense JQ796661
Bradyrhizobium centrosematis KC247115
Bradyrhizobium guangxiense KC508877
Turneriella parva TYGS 1062
Leptonema illini TYGS 1066
100/100
Leptospira idonii AB721966
100/100
100/- Leptospira yanagawae TYGS 3508
100/100
100/100 Leptospira biflexa TYGS 988
Leptospira meyeri TYGS 22775
100/-
100/- Leptospira terpstrae TYGS 3725
100/100 Leptospira wolbachii TYGS 3824
Leptospira vanthielii TYGS 3104
100/100
Leptospira fainei TYGS 3591
100/100
100/91 Leptospira inadai TYGS 5830
100/100
Leptospira broomii TYGS 5787
Leptospira wolffii TYGS 3549
100/100
100/76 Leptospira venezuelensis TYGS 19182
100/100 Leptospira licerasiae TYGS 1720

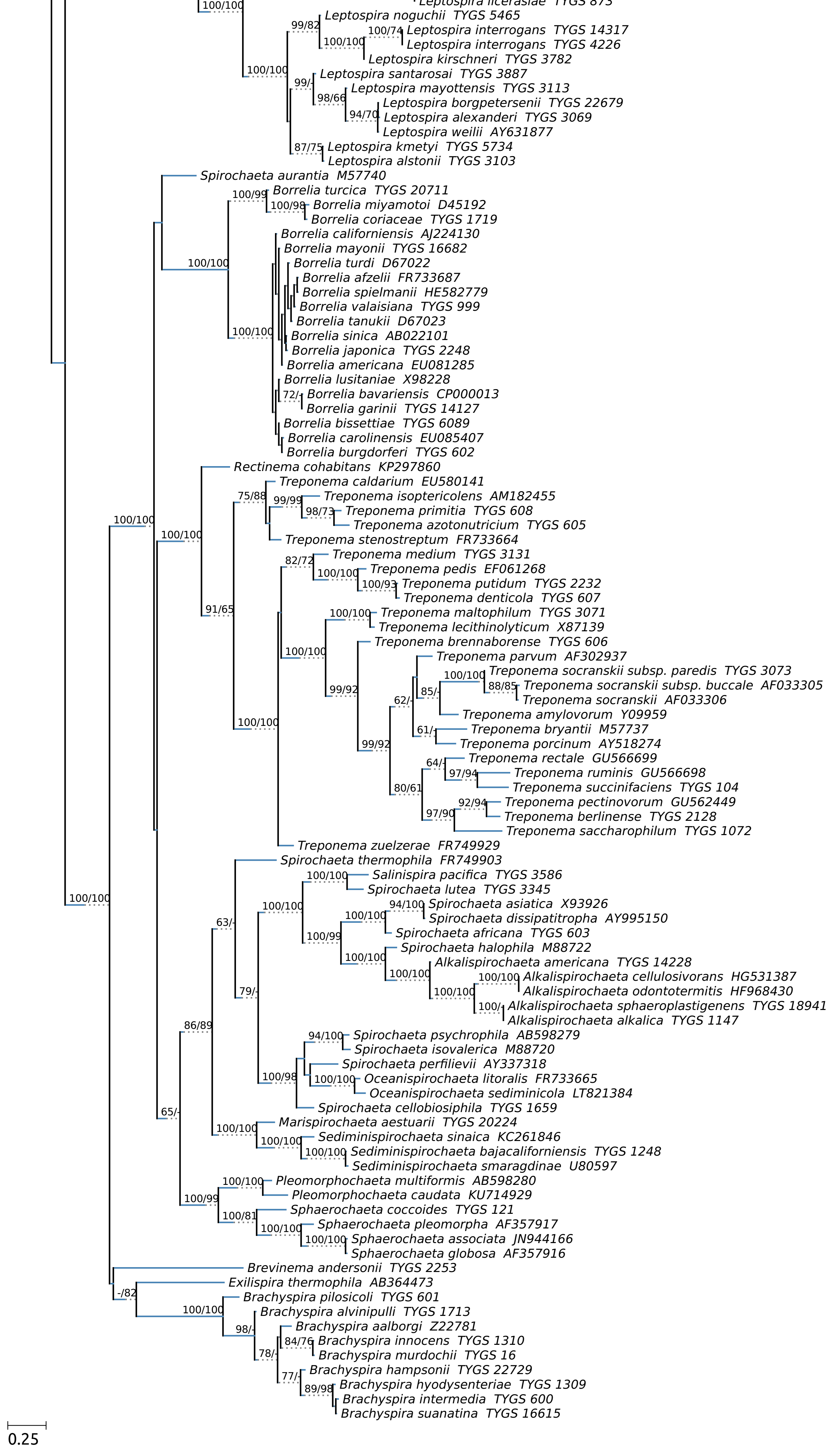
Leptospira noguchii TYGS 5465
Leptospira interrogans TYGS 14317
Leptospira interrogans TYGS 4226
Leptospira kirschneri TYGS 3782
Leptospira santarosai TYGS 3887
Leptospira mayottensis TYGS 3113
Leptospira borgpetersenii TYGS 22679
Leptospira alexanderi TYGS 3069
Leptospira weilii AY631877
Leptospira kmetyi TYGS 5734
Leptospira alstonii TYGS 3103
Spirochaeta aurantia M57740
Borrelia turcica TYGS 20711
Borrelia miyamotoi D45192
Borrelia coriaceae TYGS 1719
Borrelia californiensis AJ224130
Borrelia mayonii TYGS 16682
Borrelia turdi D67022
Borrelia afzelii FR733687
Borrelia spielmanii HE582779
Borrelia valaisiana TYGS 999
Borrelia tanukii D67023
Borrelia sinica AB022101
Borrelia japonica TYGS 2248
Borrelia americana EU081285
Borrelia lusitaniae X98228
Borrelia bavariensis CP000013
Borrelia garinii TYGS 14127
Borrelia bissettiae TYGS 6089
Borrelia carolinensis EU085407
Borrelia burgdorferi TYGS 602
Rectinema cohabitans KP297860
Treponema caldarium EU580141
Treponema isoptericolens AM182455
Treponema primitia TYGS 608
Treponema azotonutricium TYGS 605
Treponema stenostreptum FR733664
Treponema medium TYGS 3131
Treponema pedis EF061268
Treponema putidum TYGS 2232
Treponema denticola TYGS 607
Treponema maltophilum TYGS 3071
Treponema lecithinolyticum X87139
Treponema brennaborense TYGS 606
Treponema parvum AF302937
Treponema socranskii subsp. paredis TYGS 3073
Treponema socranskii subsp. buccale AF033305
Treponema socranskii AF033306
Treponema amylovorum Y09959
Treponema bryantii M57737
Treponema porcinum AY518274
Treponema rectale GU566699
Treponema ruminis GU566698
Treponema succinifaciens TYGS 104
Treponema pectinovorum GU562449
Treponema berlinense TYGS 2128
Treponema saccharophilum TYGS 1072
Treponema zuelzerae FR749929
Spirochaeta thermophila FR749903
Salinispira pacifica TYGS 3586
Spirochaeta lutea TYGS 3345
Spirochaeta asiatica X93926
Spirochaeta dissipatitropha AY995150
Spirochaeta africana TYGS 603
Spirochaeta halophila M88722
Alkalispirochaeta americana TYGS 14228
Alkalispirochaeta cellulosivorans HG531387
Alkalispirochaeta odontotermitis HF968430
Alkalispirochaeta sphaeroplastigenens TYGS 18941
Alkalispirochaeta alkalica TYGS 1147
Spirochaeta psychrophila AB598279
Spirochaeta isovalerica M88720
Spirochaeta perfilievii AY337318
Oceanispirochaeta litoralis FR733665
Oceanispirochaeta sediminicola LT821384
Spirochaeta cellobiosiphila TYGS 1659
Marispirochaeta aestuarii TYGS 20224
Sediminispirochaeta sinaica KC261846
Sediminispirochaeta bajacaliforniensis TYGS 1248
Sediminispirochaeta smaragdinae U80597
Pleomorphochaeta multiformis AB598280
Pleomorphochaeta caudata KU714929
Sphaerochaeta coccoides TYGS 121
Sphaerochaeta pleomorpha AF357917
Sphaerochaeta associata JN944166
Sphaerochaeta globosa AF357916
Brevinema andersonii TYGS 2253
Exilispira thermophila AB364473
Brachyspira pilosicoli TYGS 601
Brachyspira alvinipulli TYGS 1713
Brachyspira aalborgi Z22781
Brachyspira innocens TYGS 1310
Brachyspira murdochii TYGS 16
Brachyspira hampsonii TYGS 22729
Brachyspira hyodysenteriae TYGS 1309
Brachyspira intermedia TYGS 600
Brachyspira suanatina TYGS 16615
0.25

Figure 2: Backbone-constrained comprehensive 16S rRNA gene ML tree (CCT) of *Alphaproteobacteria* inferred under the GTR+CAT model. The branches are scaled in terms of the expected number of substitutions per site. The numbers above the branches are support values when larger than 60% from ML (left) and MP (right) bootstrapping. Dotted parts of branches are filled in to allow proper placement of bootstrap values and are not part of the actual branch length. Numbers preceeded by the term 'TYGS' in labels refer to the genome IDs as found in Supplementary Table S1 (first sheet). Each tip label ends with the family of the respective taxon.

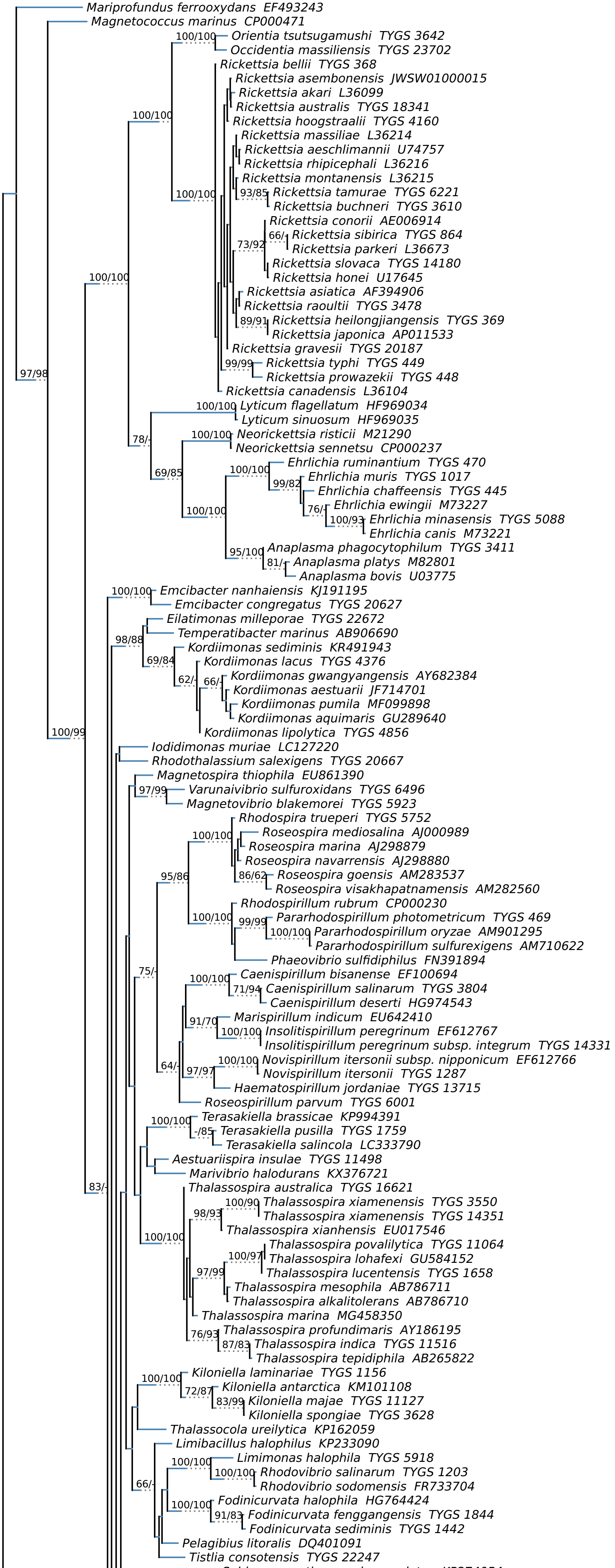

Mariprofundus ferrooxydans EF493243
Magnetococcus marinus CP000471
100/100
Orientia tsutsugamushi TYGS 3642
Occidentia massiliensis TYGS 23702
Rickettsia bellii TYGS 368
Rickettsia asembonensis JWSW01000015
Rickettsia akari L36099
Rickettsia australis TYGS 18341
100/100
Rickettsia hoogstraalii TYGS 4160
Rickettsia massiliae L36214
Rickettsia aeschlimannii U74757
Rickettsia rhipicephali L36216
Rickettsia montanensis L36215
93/85
Rickettsia tamurae TYGS 6221
100/100
Rickettsia buchneri TYGS 3610
Rickettsia conorii AE006914
66/-
Rickettsia sibirica TYGS 864
73/92
Rickettsia parkeri L36673
Rickettsia slovaca TYGS 14180
100/100
Rickettsia honei U17645
Rickettsia asiatica AF394906
Rickettsia raoultii TYGS 3478
89/91
Rickettsia heilongjiangensis TYGS 369
Rickettsia japonica AP011533
Rickettsia gravesii TYGS 20187
99/99
Rickettsia typhi TYGS 449
97/98
Rickettsia prowazekii TYGS 448
Rickettsia canadensis L36104
100/100
Lyticum flagellatum HF969034
Lyticum sinuosum HF969035
100/100
Neorickettsia risticii M21290
78/-
Neorickettsia sennetsu CP000237
Ehrlichia ruminantium TYGS 470
100/100
69/85
Ehrlichia muris TYGS 1017
99/82
Ehrlichia chaffeensis TYGS 445
76/-
Ehrlichia ewingii M73227
100/100
100/93
Ehrlichia minasensis TYGS 5088
Ehrlichia canis M73221
95/100
Anaplasma phagocytophilum TYGS 3411
81/-
Anaplasma platys M82801
Anaplasma bovis U03775
100/100
Emcibacter nanhaiensis KJ191195
Emcibacter congregatus TYGS 20627
Eilatimonas milleporae TYGS 22672
Temperatibacter marinus AB906690
98/88
Kordiimonas sediminis KR491943
69/84
Kordiimonas lacus TYGS 4376
66/-
Kordiimonas gwangyangensis AY682384
62/-
Kordiimonas aestuarii JF714701
Kordiimonas pumila MF099898
Kordiimonas aquimaris GU289640
100/99
Kordiimonas lipolytica TYGS 4856
Iodidimonas muriae LC127220
Rhodothalassium salexigens TYGS 20667
Magnetospira thiophila EU861390
97/99
Varunaivibrio sulfuroxidans TYGS 6496
Magnetovibrio blakemorei TYGS 5923
Rhodospira trueperi TYGS 5752
100/100
Roseospira mediosalina AJ000989
Roseospira marina AJ298879
Roseospira navarrensis AJ298880
95/86
86/62
Roseospira goensis AM283537
Roseospira visakhapatnamensis AM282560
Rhodospirillum rubrum CP000230
100/100
Pararhodospirillum photometricum TYGS 469
99/99
100/100
Pararhodospirillum oryzae AM901295
Pararhodospirillum sulfurexigens AM710622
75/-
Phaeovibrio sulfidiphilus FN391894
100/100
Caenispirillum bisanense EF100694
71/94
Caenispirillum salinarum TYGS 3804
Caenispirillum deserti HG974543
91/70
Marispirillum indicum EU642410
100/100
Insolitispirillum peregrinum EF612767
Insolitispirillum peregrinum subsp. integrum TYGS 14331
100/100
Novispirillum itersonii subsp. nipponicum EF612766
64/-
97/97
Novispirillum itersonii TYGS 1287
Haematospirillum jordaniae TYGS 13715
Roseospirillum parvum TYGS 6001
100/100
Terasakiella brassicae KP994391
-/85
Terasakiella pusilla TYGS 1759
Terasakiella salincola LC333790
Aestuariispira insulae TYGS 11498
Marivibrio halodurans KX376721
83/-
Thalassospira australica TYGS 16621
100/90
Thalassospira xiamenensis TYGS 3550
98/93
Thalassospira xiamenensis TYGS 14351
Thalassospira xianhensis EU017546
100/100
Thalassospira povalilytica TYGS 11064
100/97
Thalassospira lohafexi GU584152
97/99
Thalassospira lucentensis TYGS 1658
Thalassospira mesophila AB786711
Thalassospira alkalitolerans AB786710
Thalassospira marina MG458350
76/93
Thalassospira profundimaris AY186195
87/83
Thalassospira indica TYGS 11516
Thalassospira tepidiphila AB265822
100/100
Kiloniella laminariae TYGS 1156
72/87
Kiloniella antarctica KM101108
83/99
Kiloniella majae TYGS 11127
Kiloniella spongiae TYGS 3628
Thalassocola ureilytica KP162059
Limibacillus halophilus KP233090
100/100
Limimonas halophila TYGS 5918
100/100
Rhodovibrio salinarum TYGS 1203
66/-
Rhodovibrio sodomensis FR733704
100/100
Fodinicurvata halophila HG764424
91/83
Fodinicurvata fenggangensis TYGS 1844
Fodinicurvata sediminis TYGS 1442
Pelagibius litoralis DQ401091
Tistlia consotensis TYGS 22247

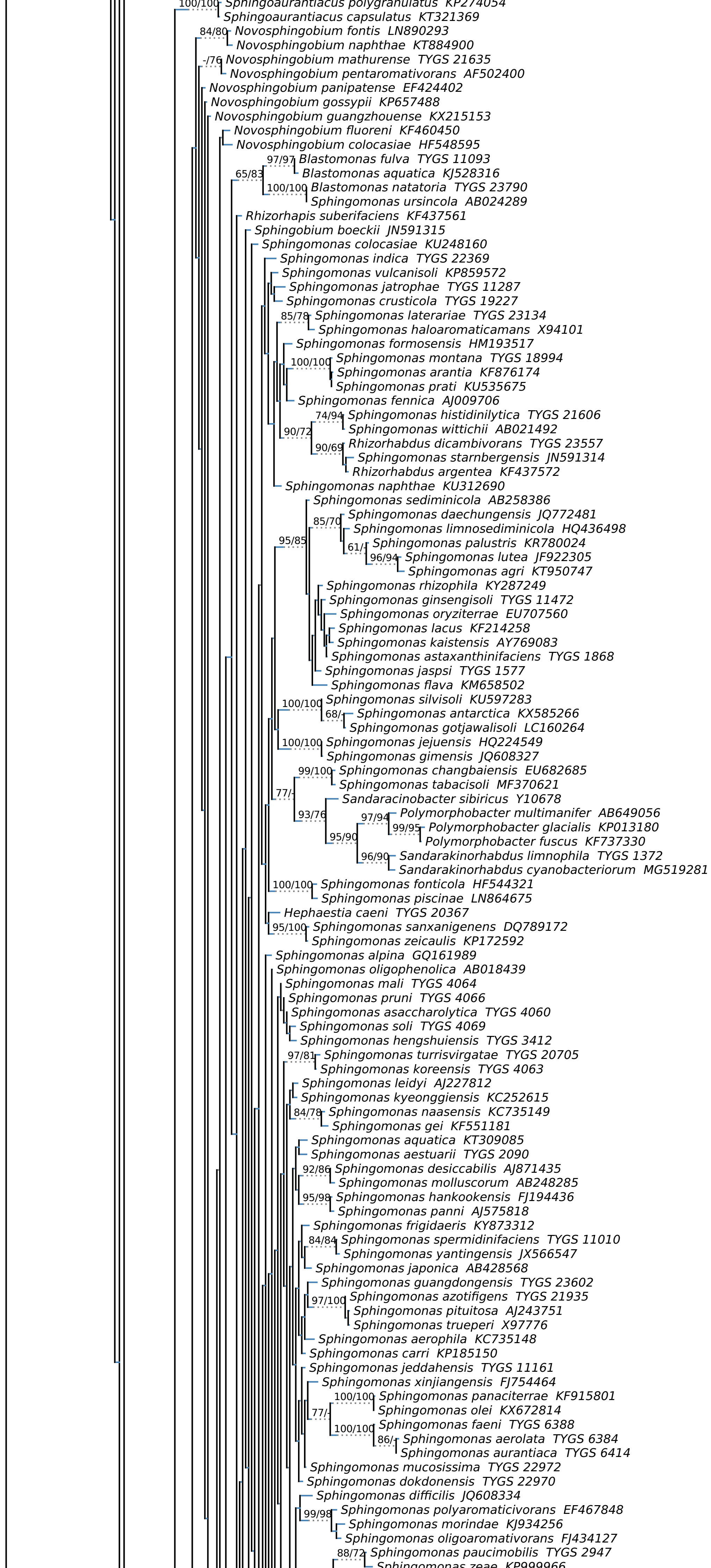
100/100 Sphingoaurantiacus polygranulatus KP274054
Sphingoaurantiacus capsulatus KT321369
84/80 Novosphingobium fontis LN890293
Novosphingobium naphthae KT884900
-/76 Novosphingobium mathurense TYGS 21635
Novosphingobium pentaromativorans AF502400
Novosphingobium panipatense EF424402
Novosphingobium gossypii KP657488
Novosphingobium guangzhouense KX215153
Novosphingobium fluoreni KF460450
Novosphingobium colocasiae HF548595
97/97 Blastomonas fulva TYGS 11093
65/83 Blastomonas aquatica KJ528316
100/100 Blastomonas natatoria TYGS 23790
Sphingomonas ursincola AB024289
Rhizorhapis suberifaciens KF437561
Sphingobium boeckii JN591315
Sphingomonas colocasiae KU248160
Sphingomonas indica TYGS 22369
Sphingomonas vulcanisoli KP859572
Sphingomonas jatrophae TYGS 11287
Sphingomonas crusticola TYGS 19227
85/78 Sphingomonas laterariae TYGS 23134
Sphingomonas haloaromaticamans X94101
Sphingomonas formosensis HM193517
100/100 Sphingomonas montana TYGS 18994
Sphingomonas arantia KF876174
Sphingomonas prati KU535675
Sphingomonas fennica AJ009706
74/94 Sphingomonas histidinilytica TYGS 21606
90/72 Sphingomonas wittichii AB021492
90/69 Rhizorhabdus dicambivorans TYGS 23557
Sphingomonas starnbergensis JN591314
Rhizorhabdus argentea KF437572
Sphingomonas naphthae KU312690
Sphingomonas sediminicola AB258386
85/70 Sphingomonas daechungensis JQ772481
Sphingomonas limnosediminicola HQ436498
95/85 61/- Sphingomonas palustris KR780024
96/94 Sphingomonas lutea JF922305
Sphingomonas agri KT950747
Sphingomonas rhizophila KY287249
Sphingomonas ginsengisoli TYGS 11472
Sphingomonas oryziterrae EU707560
Sphingomonas lacus KF214258
Sphingomonas kaistensis AY769083
Sphingomonas astaxanthinifaciens TYGS 1868
Sphingomonas jaspsi TYGS 1577
Sphingomonas flava KM658502
100/100 Sphingomonas silvisoli KU597283
68/- Sphingomonas antarctica KX585266
Sphingomonas gotjawalisoli LC160264
100/100 Sphingomonas jejuensis HQ224549
Sphingomonas gimensis JQ608327
99/100 Sphingomonas changbaiensis EU682685
Sphingomonas tabacisoli MF370621
77/- Sandaracinobacter sibiricus Y10678
93/76 97/94 Polymorphobacter multimanifer AB649056
99/95 Polymorphobacter glacialis KP013180
95/90 Polymorphobacter fuscus KF737330
96/90 Sandarakinorhabdus limnophila TYGS 1372
Sandarakinorhabdus cyanobacteriorum MG519281
100/100 Sphingomonas fonticola HF544321
Sphingomonas piscinae LN864675
Hephaestia caeni TYGS 20367
95/100 Sphingomonas sanxanigenens DQ789172
Sphingomonas zeicaulis KP172592
Sphingomonas alpina GQ161989
Sphingomonas oligophenolica AB018439
Sphingomonas mali TYGS 4064
Sphingomonas pruni TYGS 4066
Sphingomonas asaccharolytica TYGS 4060
Sphingomonas soli TYGS 4069
Sphingomonas hengshuiensis TYGS 3412
97/81 Sphingomonas turrisvirgatae TYGS 20705
Sphingomonas koreensis TYGS 4063
Sphingomonas leidyi AJ227812
Sphingomonas kyeonggiensis KC252615
84/78 Sphingomonas naasensis KC735149
Sphingomonas gei KF551181
Sphingomonas aquatica KT309085
Sphingomonas aestuarii TYGS 2090
92/86 Sphingomonas desiccabilis AJ871435
Sphingomonas molluscorum AB248285
95/98 Sphingomonas hankookensis FJ194436
Sphingomonas panni AJ575818
Sphingomonas frigidaeris KY873312
84/84 Sphingomonas spermidinifaciens TYGS 11010
Sphingomonas yantingensis JX566547
Sphingomonas japonica AB428568
Sphingomonas guangdongensis TYGS 23602
97/100 Sphingomonas azotifigens TYGS 21935
Sphingomonas pituitosa AJ243751
Sphingomonas trueperi X97776
Sphingomonas aerophila KC735148
Sphingomonas carri KP185150
Sphingomonas jeddahensis TYGS 11161
Sphingomonas xinjiangensis FJ754464
100/100 Sphingomonas panaciterrae KF915801
77/- Sphingomonas olei KX672814
100/100 Sphingomonas faeni TYGS 6388
86/- Sphingomonas aerolata TYGS 6384
Sphingomonas aurantiaca TYGS 6414
Sphingomonas mucosissima TYGS 22972
Sphingomonas dokdonensis TYGS 22970
Sphingomonas difficilis JQ608334
Sphingomonas polyaromaticivorans EF467848
99/98 Sphingomonas morindae KJ934256
Sphingomonas oligoaromativorans FJ434127
88/72 Sphingomonas paucimobilis TYGS 2947
Sphingomonas zeae KP999966

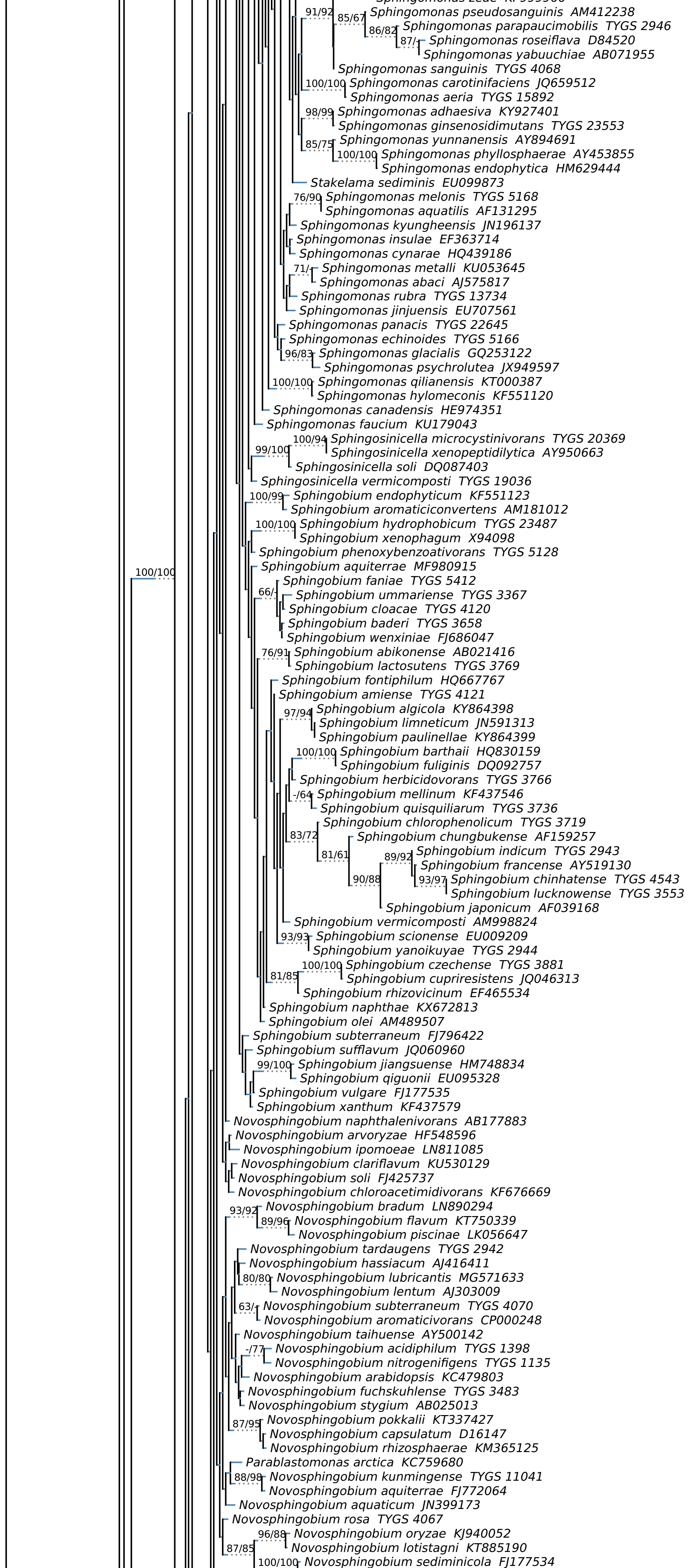
91/92
85/67
Sphingomonas pseudosanguinis AM412238
86/82
Sphingomonas parapaucimobilis TYGS 2946
87/-
Sphingomonas roseiflava D84520
Sphingomonas yabuuchiae AB071955
Sphingomonas sanguinis TYGS 4068
100/100
Sphingomonas carotinifaciens JQ659512
Sphingomonas aeria TYGS 15892
98/99
Sphingomonas adhaesiva KY927401
Sphingomonas ginsenosidimutans TYGS 23553
85/75
Sphingomonas yunnanensis AY894691
100/100
Sphingomonas phyllosphaerae AY453855
Sphingomonas endophytica HM629444
Stakelama sediminis EU099873
76/90
Sphingomonas melonis TYGS 5168
Sphingomonas aquatilis AF131295
Sphingomonas kyungheensis JN196137
Sphingomonas insulae EF363714
Sphingomonas cynarae HQ439186
71/-
Sphingomonas metalli KU053645
Sphingomonas abaci AJ575817
Sphingomonas rubra TYGS 13734
Sphingomonas jinjuensis EU707561
Sphingomonas panacis TYGS 22645
Sphingomonas echinoides TYGS 5166
96/83
Sphingomonas glacialis GQ253122
Sphingomonas psychrolutea JX949597
100/100
Sphingomonas qilianensis KT000387
Sphingomonas hylomeconis KF551120
Sphingomonas canadensis HE974351
Sphingomonas faucium KU179043
100/94
Sphingosinicella microcystinivorans TYGS 20369
99/100
Sphingosinicella xenopeptidilytica AY950663
Sphingosinicella soli DQ087403
Sphingosinicella vermicomposti TYGS 19036
100/99
Sphingobium endophyticum KF551123
Sphingobium aromaticiconvertens AM181012
100/100
Sphingobium hydrophobicum TYGS 23487
Sphingobium xenophagum X94098
Sphingobium phenoxybenzoativorans TYGS 5128
100/100
Sphingobium aquiterrae MF980915
Sphingobium faniae TYGS 5412
66/-
Sphingobium ummariense TYGS 3367
Sphingobium cloacae TYGS 4120
Sphingobium baderi TYGS 3658
Sphingobium wenxiniae FJ686047
76/91
Sphingobium abikonense AB021416
Sphingobium lactosutens TYGS 3769
Sphingobium fontiphilum HQ667767
Sphingobium amiense TYGS 4121
97/94
Sphingobium algicola KY864398
Sphingobium limneticum JN591313
Sphingobium paulinellae KY864399
100/100
Sphingobium barthaii HQ830159
Sphingobium fuliginis DQ092757
Sphingobium herbicidovorans TYGS 3766
-/64
Sphingobium mellinum KF437546
Sphingobium quisquiliarum TYGS 3736
Sphingobium chlorophenolicum TYGS 3719
83/72
Sphingobium chungbukense AF159257
81/61
89/92
Sphingobium indicum TYGS 2943
Sphingobium francense AY519130
90/88
93/97
Sphingobium chinhatense TYGS 4543
Sphingobium lucknowense TYGS 3553
Sphingobium japonicum AF039168
Sphingobium vermicomposti AM998824
93/93
Sphingobium scionense EU009209
Sphingobium yanoikuyae TYGS 2944
100/100
Sphingobium czechense TYGS 3881
81/85
Sphingobium cupriresistens JQ046313
Sphingobium rhizovicinum EF465534
Sphingobium naphthae KX672813
Sphingobium olei AM489507
Sphingobium subterraneum FJ796422
Sphingobium sufflavum JQ060960
99/100
Sphingobium jiangsuense HM748834
Sphingobium qiguonii EU095328
Sphingobium vulgare FJ177535
Sphingobium xanthum KF437579
Novosphingobium naphthalenivorans AB177883
Novosphingobium arvoryzae HF548596
Novosphingobium ipomoeae LN811085
Novosphingobium clariflavum KU530129
Novosphingobium soli FJ425737
Novosphingobium chloroacetimidivorans KF676669
93/92
Novosphingobium bradum LN890294
89/96
Novosphingobium flavum KT750339
Novosphingobium piscinae LK056647
Novosphingobium tardaugens TYGS 2942
Novosphingobium hassiacum AJ416411
80/80
Novosphingobium lubricantis MG571633
Novosphingobium lentum AJ303009
63/-
Novosphingobium subterraneum TYGS 4070
Novosphingobium aromaticivorans CP000248
Novosphingobium taihuense AY500142
-/77
Novosphingobium acidiphilum TYGS 1398
Novosphingobium nitrogenifigens TYGS 1135
Novosphingobium arabidopsis KC479803
Novosphingobium fuchskuhlense TYGS 3483
Novosphingobium stygium AB025013
87/95
Novosphingobium pokkalii KT337427
Novosphingobium capsulatum D16147
Novosphingobium rhizosphaerae KM365125
Parablastomonas arctica KC759680
88/98
Novosphingobium kunmingense TYGS 11041
Novosphingobium aquiterrae FJ772064
Novosphingobium aquaticum JN399173
Novosphingobium rosa TYGS 4067
96/88
Novosphingobium oryzae KJ940052
87/85
Novosphingobium lotistagni KT885190
100/100
Novosphingobium sediminicola FJ177534

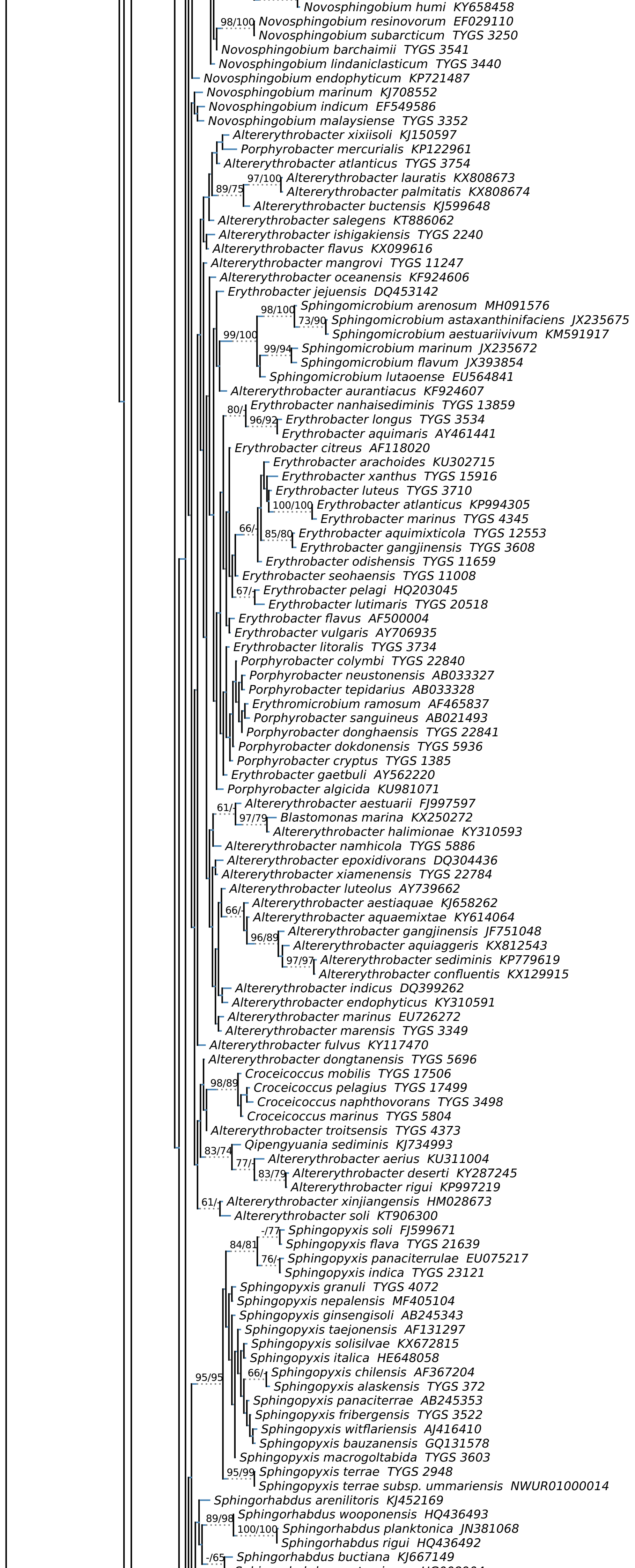

Novosphingobium humi KY658458
98/100 Novosphingobium resinovorum EF029110
Novosphingobium subarcticum TYGS 3250
Novosphingobium barchaimii TYGS 3541
Novosphingobium lindaniclasticum TYGS 3440
Novosphingobium endophyticum KP721487
Novosphingobium marinum KJ708552
Novosphingobium indicum EF549586
Novosphingobium malaysiense TYGS 3352
Altererythrobacter xixiisoli KJ150597
Porphyrobacter mercurialis KP122961
Altererythrobacter atlanticus TYGS 3754
97/100 Altererythrobacter lauratis KX808673
89/75 Altererythrobacter palmitatis KX808674
Altererythrobacter buctensis KJ599648
Altererythrobacter salegens KT886062
Altererythrobacter ishigakiensis TYGS 2240
Altererythrobacter flavus KX099616
Altererythrobacter mangrovi TYGS 11247
Altererythrobacter oceanensis KF924606
Erythrobacter jejuensis DQ453142
98/100 Sphingomicrobium arenosum MH091576
73/90 Sphingomicrobium astaxanthinifaciens JX235675
99/100 Sphingomicrobium aestuariivivum KM591917
99/94 Sphingomicrobium marinum JX235672
Sphingomicrobium flavum JX393854
Sphingomicrobium lutaoense EU564841
Altererythrobacter aurantiacus KF924607
80/- Erythrobacter nanhaisediminis TYGS 13859
96/92 Erythrobacter longus TYGS 3534
Erythrobacter aquimaris AY461441
Erythrobacter citreus AF118020
Erythrobacter arachoides KU302715
Erythrobacter xanthus TYGS 15916
Erythrobacter luteus TYGS 3710
100/100 Erythrobacter atlanticus KP994305
Erythrobacter marinus TYGS 4345
66/- 85/80 Erythrobacter aquimixticola TYGS 12553
Erythrobacter gangjinensis TYGS 3608
Erythrobacter odishensis TYGS 11659
Erythrobacter seohaensis TYGS 11008
67/- Erythrobacter pelagi HQ203045
Erythrobacter lutimaris TYGS 20518
Erythrobacter flavus AF500004
Erythrobacter vulgaris AY706935
Erythrobacter litoralis TYGS 3734
Porphyrobacter colymbi TYGS 22840
Porphyrobacter neustonensis AB033327
Porphyrobacter tepidarius AB033328
Erythromicrobium ramosum AF465837
Porphyrobacter sanguineus AB021493
Porphyrobacter donghaensis TYGS 22841
Porphyrobacter dokdonensis TYGS 5936
Porphyrobacter cryptus TYGS 1385
Erythrobacter gaetbuli AY562220
Porphyrobacter algicida KU981071
61/- Altererythrobacter aestuarii FJ997597
97/79 Blastomonas marina KX250272
Altererythrobacter halimionae KY310593
Altererythrobacter namhicola TYGS 5886
Altererythrobacter epoxidivorans DQ304436
Altererythrobacter xiamenensis TYGS 22784
Altererythrobacter luteolus AY739662
Altererythrobacter aestiaquae KJ658262
66/- Altererythrobacter aquaemixtae KY614064
96/89 Altererythrobacter gangjinensis JF751048
Altererythrobacter aquiaggeris KX812543
97/97 Altererythrobacter sediminis KP779619
Altererythrobacter confluentis KX129915
Altererythrobacter indicus DQ399262
Altererythrobacter endophyticus KY310591
Altererythrobacter marinus EU726272
Altererythrobacter marensis TYGS 3349
Altererythrobacter fulvus KY117470
Altererythrobacter dongtanensis TYGS 5696
Croceicoccus mobilis TYGS 17506
98/89 Croceicoccus pelagius TYGS 17499
Croceicoccus naphthovorans TYGS 3498
Croceicoccus marinus TYGS 5804
Altererythrobacter troitsensis TYGS 4373
83/74 Qipengyuania sediminis KJ734993
77/- Altererythrobacter aerius KU311004
83/79 Altererythrobacter deserti KY287245
Altererythrobacter rigui KP997219
61/- Altererythrobacter xinjiangensis HM028673
Altererythrobacter soli KT906300
-/77 Sphingopyxis soli FJ599671
84/81 Sphingopyxis flava TYGS 21639
76/- Sphingopyxis panaciterrulae EU075217
Sphingopyxis indica TYGS 23121
Sphingopyxis granuli TYGS 4072
Sphingopyxis nepalensis MF405104
Sphingopyxis ginsengisoli AB245343
Sphingopyxis taejonensis AF131297
Sphingopyxis solisilvae KX672815
Sphingopyxis italica HE648058
66/- Sphingopyxis chilensis AF367204
95/95 Sphingopyxis alaskensis TYGS 372
Sphingopyxis panaciterrae AB245353
Sphingopyxis fribergensis TYGS 3522
Sphingopyxis witflariensis AJ416410
Sphingopyxis bauzanensis GQ131578
Sphingopyxis macrogoltabida TYGS 3603
95/99 Sphingopyxis terrae TYGS 2948
Sphingopyxis terrae subsp. ummariensis NWUR01000014
Sphingorhabdus arenilitoris KJ452169
89/98 Sphingorhabdus wooponensis HQ436493
100/100 Sphingorhabdus planktonica JN381068
Sphingorhabdus rigui HQ436492
-/65 Sphingorhabdus buctiana KJ667149

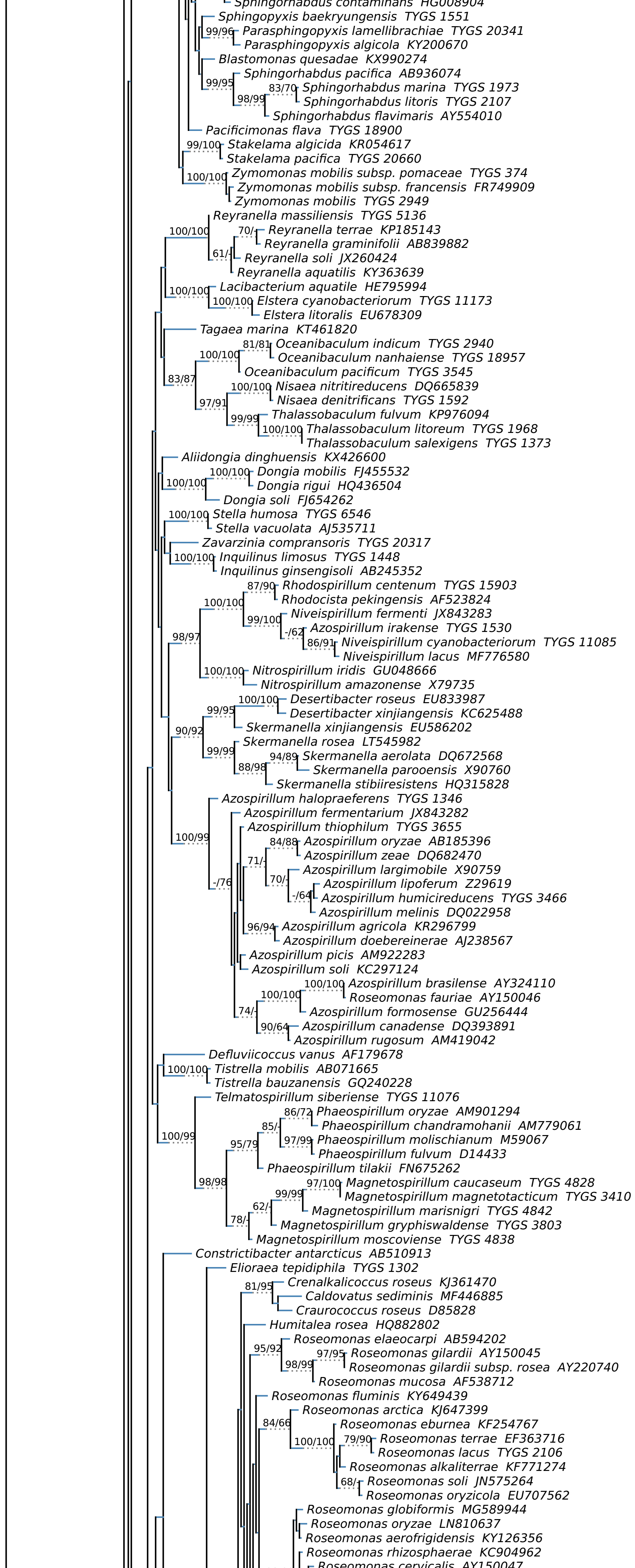
Sphingorhabdus contaminans HG008904
Sphingopyxis baekryungensis TYGS 1551
Parasphingopyxis lamellibrachiae TYGS 20341
Parasphingopyxis algicola KY200670
Blastomonas quesadae KX990274
Sphingorhabdus pacifica AB936074
Sphingorhabdus marina TYGS 1973
Sphingorhabdus litoris TYGS 2107
Sphingorhabdus flavimaris AY554010
Pacificimonas flava TYGS 18900
Stakelama algicida KR054617
Stakelama pacifica TYGS 20660
Zymomonas mobilis subsp. pomaceae TYGS 374
Zymomonas mobilis subsp. francensis FR749909
Zymomonas mobilis TYGS 2949
Reyranella massiliensis TYGS 5136
Reyranella terrae KP185143
Reyranella graminifolii AB839882
Reyranella soli JX260424
Reyranella aquatilis KY363639
Lacibacterium aquatile HE795994
Elstera cyanobacteriorum TYGS 11173
Elstera litoralis EU678309
Tagaea marina KT461820
Oceanibaculum indicum TYGS 2940
Oceanibaculum nanhaiense TYGS 18957
Oceanibaculum pacificum TYGS 3545
Nisaea nitritireducens DQ665839
Nisaea denitrificans TYGS 1592
Thalassobaculum fulvum KP976094
Thalassobaculum litoreum TYGS 1968
Thalassobaculum salexigens TYGS 1373
Aliidongia dinghuensis KX426600
Dongia mobilis FJ455532
Dongia rigui HQ436504
Dongia soli FJ654262
Stella humosa TYGS 6546
Stella vacuolata AJ535711
Zavarzinia compransoris TYGS 20317
Inquilinus limosus TYGS 1448
Inquilinus ginsengisoli AB245352
Rhodospirillum centenum TYGS 15903
Rhodocista pekingensis AF523824
Niveispirillum fermenti JX843283
Azospirillum irakense TYGS 1530
Niveispirillum cyanobacteriorum TYGS 11085
Niveispirillum lacus MF776580
Nitrospirillum iridis GU048666
Nitrospirillum amazonense X79735
Desertibacter roseus EU833987
Desertibacter xinjiangensis KC625488
Skermanella xinjiangensis EU586202
Skermanella rosea LT545982
Skermanella aerolata DQ672568
Skermanella parooensis X90760
Skermanella stibiiresistens HQ315828
Azospirillum halopraeferens TYGS 1346
Azospirillum fermentarium JX843282
Azospirillum thiophilum TYGS 3655
Azospirillum oryzae AB185396
Azospirillum zeae DQ682470
Azospirillum largimobile X90759
Azospirillum lipoferum Z29619
Azospirillum humicireducens TYGS 3466
Azospirillum melinis DQ022958
Azospirillum agricola KR296799
Azospirillum doebereinerae AJ238567
Azospirillum picis AM922283
Azospirillum soli KC297124
Azospirillum brasilense AY324110
Roseomonas fauriae AY150046
Azospirillum formosense GU256444
Azospirillum canadense DQ393891
Azospirillum rugosum AM419042
Defluviicoccus vanus AF179678
Tistrella mobilis AB071665
Tistrella bauzanensis GQ240228
Telmatospirillum siberiense TYGS 11076
Phaeospirillum oryzae AM901294
Phaeospirillum chandramohanii AM779061
Phaeospirillum molischianum M59067
Phaeospirillum fulvum D14433
Phaeospirillum tilakii FN675262
Magnetospirillum caucaseum TYGS 4828
Magnetospirillum magnetotacticum TYGS 3410
Magnetospirillum marisnigri TYGS 4842
Magnetospirillum gryphiswaldense TYGS 3803
Magnetospirillum moscoviense TYGS 4838
Constrictibacter antarcticus AB510913
Elioraea tepidiphila TYGS 1302
Crenalkalicoccus roseus KJ361470
Caldovatus sediminis MF446885
Craurococcus roseus D85828
Humitalea rosea HQ882802
Roseomonas elaeocarpi AB594202
Roseomonas gilardii AY150045
Roseomonas gilardii subsp. rosea AY220740
Roseomonas mucosa AF538712
Roseomonas fluminis KY649439
Roseomonas arctica KJ647399
Roseomonas eburnea KF254767
Roseomonas terrae EF363716
Roseomonas lacus TYGS 2106
Roseomonas alkaliterrae KF771274
Roseomonas soli JN575264
Roseomonas oryzicola EU707562
Roseomonas globiformis MG589944
Roseomonas oryzae LN810637
Roseomonas aerofrigidensis KY126356
Roseomonas rhizosphaerae KC904962
Roseomonas cervicalis AY150047

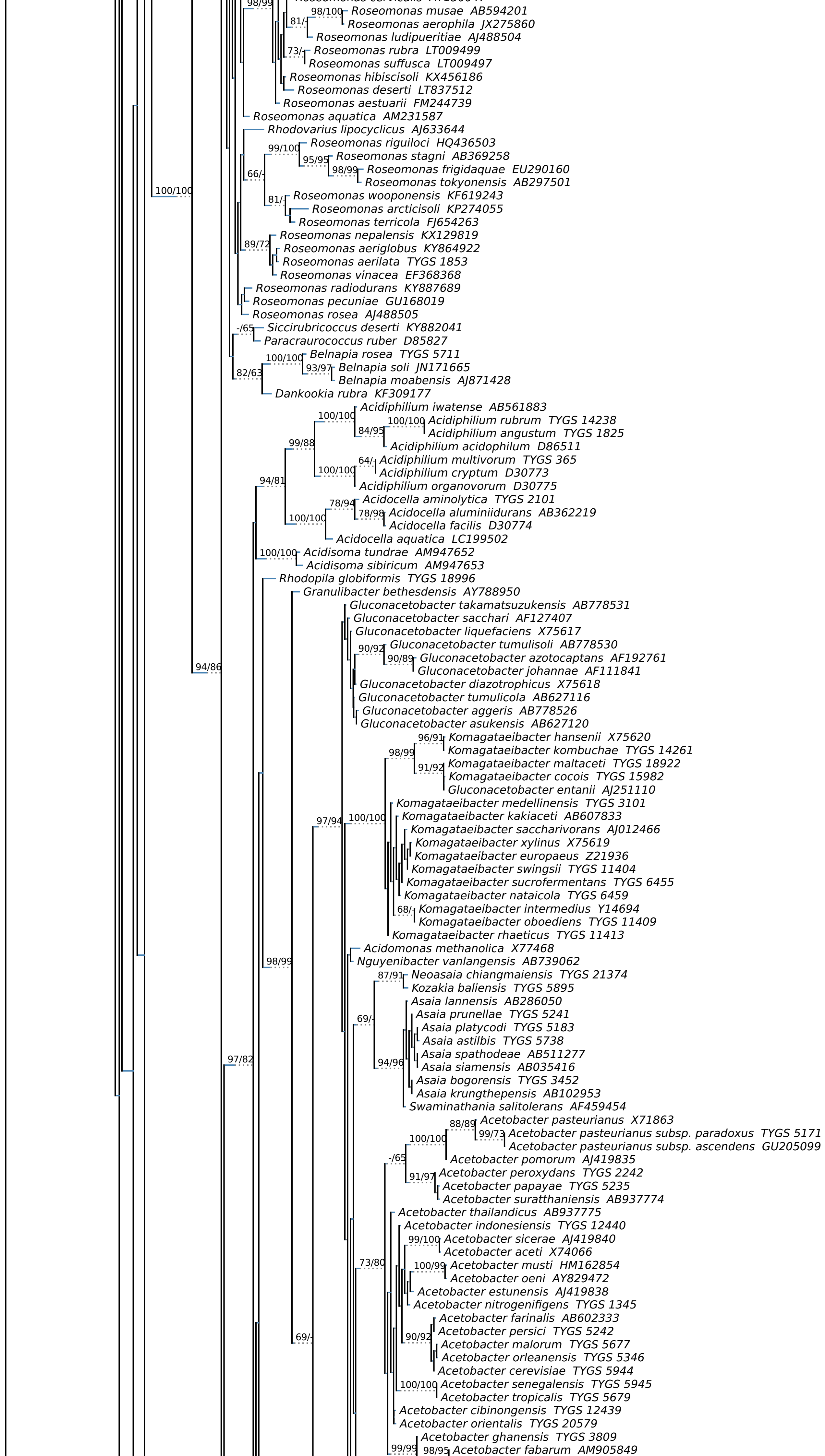

Roseomonas musae AB594201
Roseomonas aerophila JX275860
Roseomonas ludipueritiae AJ488504
Roseomonas rubra LT009499
Roseomonas suffusca LT009497
Roseomonas hibiscisoli KX456186
Roseomonas deserti LT837512
Roseomonas aestuarii FM244739
Roseomonas aquatica AM231587
Rhodovarius lipocyclicus AJ633644
Roseomonas riguiloci HQ436503
Roseomonas stagni AB369258
Roseomonas frigidaquae EU290160
Roseomonas tokyonensis AB297501
Roseomonas wooponensis KF619243
Roseomonas arcticisoli KP274055
Roseomonas terricola FJ654263
Roseomonas nepalensis KX129819
Roseomonas aeriglobus KY864922
Roseomonas aerilata TYGS 1853
Roseomonas vinacea EF368368
Roseomonas radiodurans KY887689
Roseomonas pecuniae GU168019
Roseomonas rosea AJ488505
Siccirubricoccus deserti KY882041
Paracraurococcus ruber D85827
Belnapia rosea TYGS 5711
Belnapia soli JN171665
Belnapia moabensis AJ871428
Dankookia rubra KF309177
Acidiphilium iwatense AB561883
Acidiphilium rubrum TYGS 14238
Acidiphilium angustum TYGS 1825
Acidiphilium acidophilum D86511
Acidiphilium multivorum TYGS 365
Acidiphilium cryptum D30773
Acidiphilium organovorum D30775
Acidocella aminolytica TYGS 2101
Acidocella aluminiidurans AB362219
Acidocella facilis D30774
Acidocella aquatica LC199502
Acidisoma tundrae AM947652
Acidisoma sibiricum AM947653
Rhodopila globiformis TYGS 18996
Granulibacter bethesdensis AY788950
Gluconacetobacter takamatsuzukensis AB778531
Gluconacetobacter sacchari AF127407
Gluconacetobacter liquefaciens X75617
Gluconacetobacter tumulisoli AB778530
Gluconacetobacter azotocaptans AF192761
Gluconacetobacter johannae AF111841
Gluconacetobacter diazotrophicus X75618
Gluconacetobacter tumulicola AB627116
Gluconacetobacter aggeris AB778526
Gluconacetobacter asukensis AB627120
Komagataeibacter hansenii X75620
Komagataeibacter kombuchae TYGS 14261
Komagataeibacter maltaceti TYGS 18922
Komagataeibacter cocois TYGS 15982
Gluconacetobacter entanii AJ251110
Komagataeibacter medellinensis TYGS 3101
Komagataeibacter kakiaceti AB607833
Komagataeibacter saccharivorans AJ012466
Komagataeibacter xylinus X75619
Komagataeibacter europaeus Z21936
Komagataeibacter swingsii TYGS 11404
Komagataeibacter sucrofermentans TYGS 6455
Komagataeibacter nataicola TYGS 6459
Komagataeibacter intermedius Y14694
Komagataeibacter oboediens TYGS 11409
Komagataeibacter rhaeticus TYGS 11413
Acidomonas methanolica X77468
Nguyenibacter vanlangensis AB739062
Neoasaia chiangmaiensis TYGS 21374
Kozakia baliensis TYGS 5895
Asaia lannensis AB286050
Asaia prunellae TYGS 5241
Asaia platycodi TYGS 5183
Asaia astilbis TYGS 5738
Asaia spathodeae AB511277
Asaia siamensis AB035416
Asaia bogorensis TYGS 3452
Asaia krungthepensis AB102953
Swaminathania salitolerans AF459454
Acetobacter pasteurianus X71863
Acetobacter pasteurianus subsp. paradoxus TYGS 5171
Acetobacter pasteurianus subsp. ascendens GU205099
Acetobacter pomorum AJ419835
Acetobacter peroxydans TYGS 2242
Acetobacter papayae TYGS 5235
Acetobacter suratthaniensis AB937774
Acetobacter thailandicus AB937775
Acetobacter indonesiensis TYGS 12440
Acetobacter sicerae AJ419840
Acetobacter aceti X74066
Acetobacter musti HM162854
Acetobacter oeni AY829472
Acetobacter estunensis AJ419838
Acetobacter nitrogenifigens TYGS 1345
Acetobacter farinalis AB602333
Acetobacter persici TYGS 5242
Acetobacter malorum TYGS 5677
Acetobacter orleanensis TYGS 5346
Acetobacter cerevisiae TYGS 5944
Acetobacter senegalensis TYGS 5945
Acetobacter tropicalis TYGS 5679
Acetobacter cibinongensis TYGS 12439
Acetobacter orientalis TYGS 20579
Acetobacter ghanensis TYGS 3809
Acetobacter fabarum AM905849

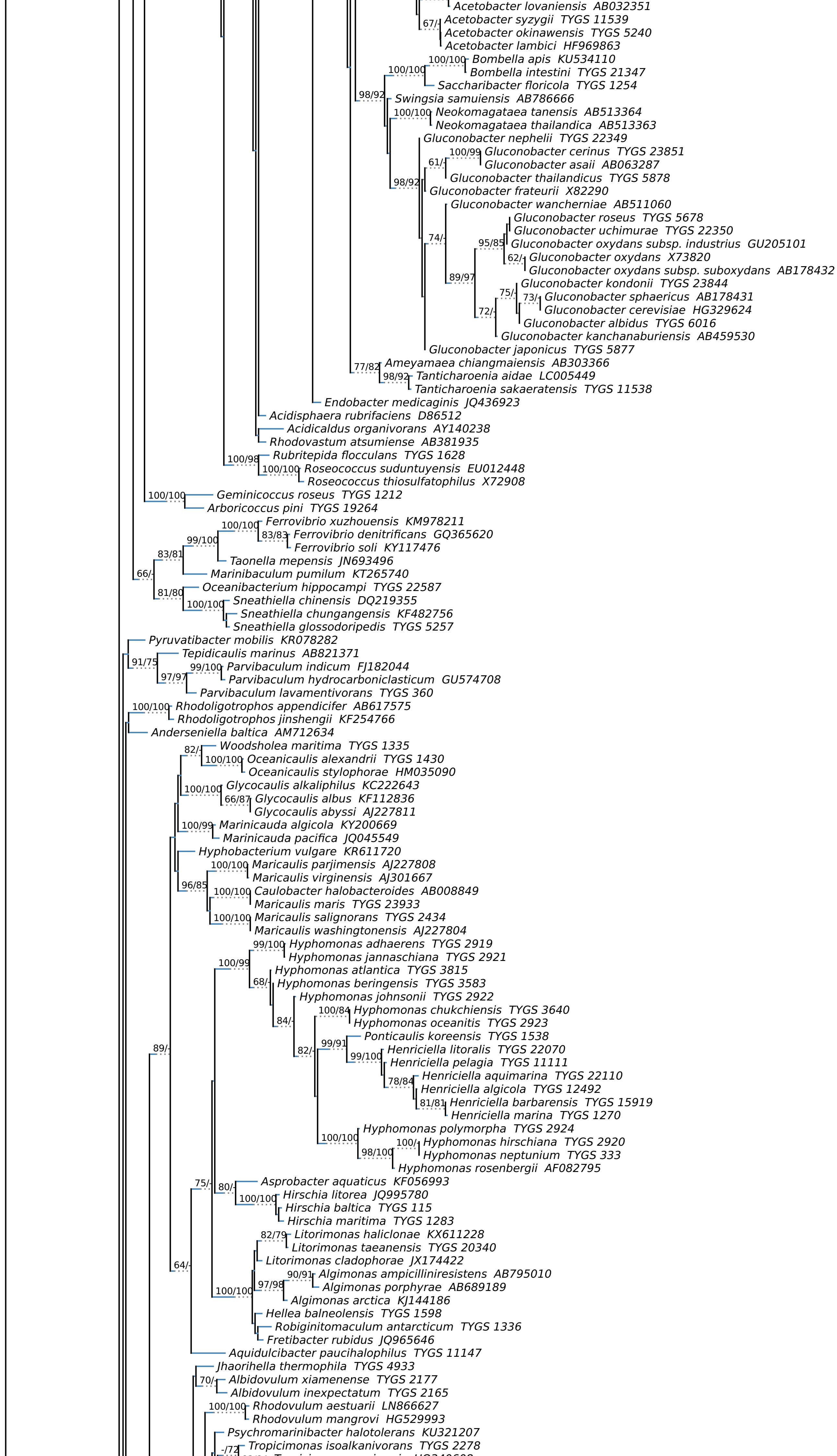
Acetobacter lovaniensis AB032351
Acetobacter syzygii TYGS 11539
Acetobacter okinawensis TYGS 5240
Acetobacter lambici HF969863
Bombella apis KU534110
Bombella intestini TYGS 21347
Saccharibacter floricola TYGS 1254
Swingsia samuiensis AB786666
Neokomagataea tanensis AB513364
Neokomagataea thailandica AB513363
Gluconobacter nephelii TYGS 22349
Gluconobacter cerinus TYGS 23851
Gluconobacter asaii AB063287
Gluconobacter thailandicus TYGS 5878
Gluconobacter frateurii X82290
Gluconobacter wancherniae AB511060
Gluconobacter roseus TYGS 5678
Gluconobacter uchimurae TYGS 22350
Gluconobacter oxydans subsp. industrius GU205101
Gluconobacter oxydans X73820
Gluconobacter oxydans subsp. suboxydans AB178432
Gluconobacter kondonii TYGS 23844
Gluconobacter sphaericus AB178431
Gluconobacter cerevisiae HG329624
Gluconobacter albidus TYGS 6016
Gluconobacter kanchanaburiensis AB459530
Gluconobacter japonicus TYGS 5877
Ameyamaea chiangmaiensis AB303366
Tanticharoenia aidae LC005449
Tanticharoenia sakaeratensis TYGS 11538
Endobacter medicaginis JQ436923
Acidisphaera rubrifaciens D86512
Acidicaldus organivorans AY140238
Rhodovastum atsumiense AB381935
Rubritepida flocculans TYGS 1628
Roseococcus suduntuyensis EU012448
Roseococcus thiosulfatophilus X72908
Geminicoccus roseus TYGS 1212
Arboricoccus pini TYGS 19264
Ferrovibrio xuzhouensis KM978211
Ferrovibrio denitrificans GQ365620
Ferrovibrio soli KY117476
Taonella mepensis JN693496
Marinibaculum pumilum KT265740
Oceanibacterium hippocampi TYGS 22587
Sneathiella chinensis DQ219355
Sneathiella chungangensis KF482756
Sneathiella glossodoripedis TYGS 5257
Pyruvatibacter mobilis KR078282
Tepidicaulis marinus AB821371
Parvibaculum indicum FJ182044
Parvibaculum hydrocarboniclasticum GU574708
Parvibaculum lavamentivorans TYGS 360
Rhodoligotrophos appendicifer AB617575
Rhodoligotrophos jinshengii KF254766
Anderseniella baltica AM712634
Woodsholea maritima TYGS 1335
Oceanicaulis alexandrii TYGS 1430
Oceanicaulis stylophorae HM035090
Glycocaulis alkaliphilus KC222643
Glycocaulis albus KF112836
Glycocaulis abyssi AJ227811
Marinicauda algicola KY200669
Marinicauda pacifica JQ045549
Hyphobacterium vulgare KR611720
Maricaulis parjimensis AJ227808
Maricaulis virginensis AJ301667
Caulobacter halobacteroides AB008849
Maricaulis maris TYGS 23933
Maricaulis salignorans TYGS 2434
Maricaulis washingtonensis AJ227804
Hyphomonas adhaerens TYGS 2919
Hyphomonas jannaschiana TYGS 2921
Hyphomonas atlantica TYGS 3815
Hyphomonas beringensis TYGS 3583
Hyphomonas johnsonii TYGS 2922
Hyphomonas chukchiensis TYGS 3640
Hyphomonas oceanitis TYGS 2923
Ponticaulis koreensis TYGS 1538
Henriciella litoralis TYGS 22070
Henriciella pelagia TYGS 11111
Henriciella aquimarina TYGS 22110
Henriciella algicola TYGS 12492
Henriciella barbarensis TYGS 15919
Henriciella marina TYGS 1270
Hyphomonas polymorpha TYGS 2924
Hyphomonas hirschiana TYGS 2920
Hyphomonas neptunium TYGS 333
Hyphomonas rosenbergii AF082795
Asprobacter aquaticus KF056993
Hirschia litorea JQ995780
Hirschia baltica TYGS 115
Hirschia maritima TYGS 1283
Litorimonas haliclonae KX611228
Litorimonas taeanensis TYGS 20340
Litorimonas cladophorae JX174422
Algimonas ampicilliniresistens AB795010
Algimonas porphyrae AB689189
Algimonas arctica KJ144186
Hellea balneolensis TYGS 1598
Robiginitomaculum antarcticum TYGS 1336
Fretibacter rubidus JQ965646
Aquidulcibacter paucihalophilus TYGS 11147
Jhaorihella thermophila TYGS 4933
Albidovulum xiamenense TYGS 2177
Albidovulum inexpectatum TYGS 2165
Rhodovulum aestuarii LN866627
Rhodovulum mangrovi HG529993
Psychromarinibacter halotolerans KU321207
Tropicimonas isoalkanivorans TYGS 2278

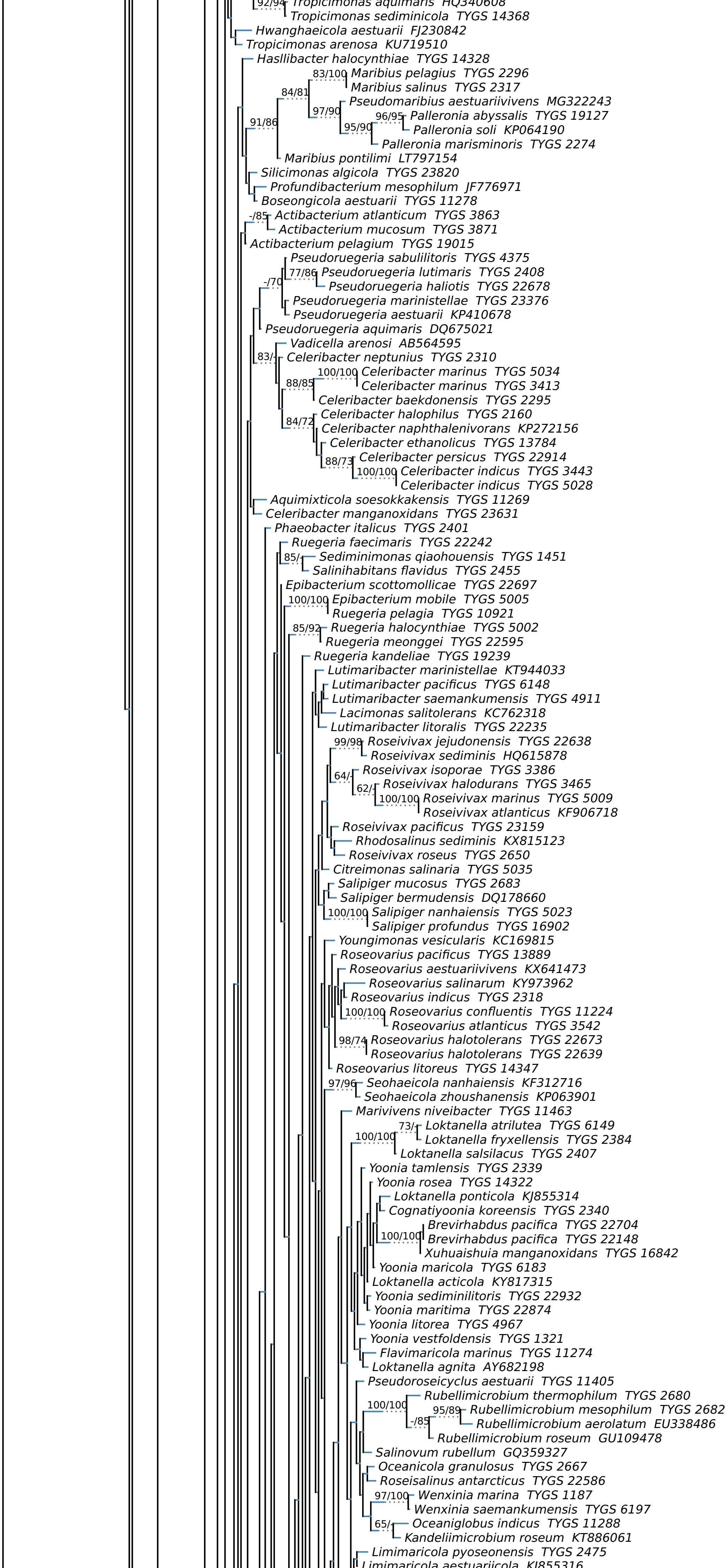
Tropicimonas aquimaris HQ340608
Tropicimonas sediminicola TYGS 14368
Hwanghaeicola aestuarii FJ230842
Tropicimonas arenosa KU719510
Hasllibacter halocynthiae TYGS 14328
Maribius pelagius TYGS 2296
Maribius salinus TYGS 2317
Pseudomaribius aestuariivivens MG322243
Palleronia abyssalis TYGS 19127
Palleronia soli KP064190
Palleronia marisminoris TYGS 2274
Maribius pontilimi LT797154
Silicimonas algicola TYGS 23820
Profundibacterium mesophilum JF776971
Boseongicola aestuarii TYGS 11278
Actibacterium atlanticum TYGS 3863
Actibacterium mucosum TYGS 3871
Actibacterium pelagium TYGS 19015
Pseudoruegeria sabulilitoris TYGS 4375
Pseudoruegeria lutimaris TYGS 2408
Pseudoruegeria haliotis TYGS 22678
Pseudoruegeria marinistellae TYGS 23376
Pseudoruegeria aestuarii KP410678
Pseudoruegeria aquimaris DQ675021
Vadicella arenosi AB564595
Celeribacter neptunius TYGS 2310
Celeribacter marinus TYGS 5034
Celeribacter marinus TYGS 3413
Celeribacter baekdonensis TYGS 2295
Celeribacter halophilus TYGS 2160
Celeribacter naphthalenivorans KP272156
Celeribacter ethanolicus TYGS 13784
Celeribacter persicus TYGS 22914
Celeribacter indicus TYGS 3443
Celeribacter indicus TYGS 5028
Aquimixticola soesokkakensis TYGS 11269
Celeribacter manganoxidans TYGS 23631
Phaeobacter italicus TYGS 2401
Ruegeria faecimaris TYGS 22242
Sediminimonas qiaohouensis TYGS 1451
Salinihabitans flavidus TYGS 2455
Epibacterium scottomollicae TYGS 22697
Epibacterium mobile TYGS 5005
Ruegeria pelagia TYGS 10921
Ruegeria halocynthiae TYGS 5002
Ruegeria meonggei TYGS 22595
Ruegeria kandeliae TYGS 19239
Lutimaribacter marinistellae KT944033
Lutimaribacter pacificus TYGS 6148
Lutimaribacter saemankumensis TYGS 4911
Lacimonas salitolerans KC762318
Lutimaribacter litoralis TYGS 22235
Roseivivax jejudonensis TYGS 22638
Roseivivax sediminis HQ615878
Roseivivax isoporae TYGS 3386
Roseivivax halodurans TYGS 3465
Roseivivax marinus TYGS 5009
Roseivivax atlanticus KF906718
Roseivivax pacificus TYGS 23159
Rhodosalinus sediminis KX815123
Roseivivax roseus TYGS 2650
Citreimonas salinaria TYGS 5035
Salipiger mucosus TYGS 2683
Salipiger bermudensis DQ178660
Salipiger nanhaiensis TYGS 5023
Salipiger profundus TYGS 16902
Youngimonas vesicularis KC169815
Roseovarius pacificus TYGS 13889
Roseovarius aestuariivivens KX641473
Roseovarius salinarum KY973962
Roseovarius indicus TYGS 2318
Roseovarius confluentis TYGS 11224
Roseovarius atlanticus TYGS 3542
Roseovarius halotolerans TYGS 22673
Roseovarius halotolerans TYGS 22639
Roseovarius litoreus TYGS 14347
Seohaeicola nanhaiensis KF312716
Seohaeicola zhoushanensis KP063901
Marivivens niveibacter TYGS 11463
Loktanella atrilutea TYGS 6149
Loktanella fryxellensis TYGS 2384
Loktanella salsilacus TYGS 2407
Yoonia tamlensis TYGS 2339
Yoonia rosea TYGS 14322
Loktanella ponticola KJ855314
Cognatiyoonia koreensis TYGS 2340
Brevirhabdus pacifica TYGS 22704
Brevirhabdus pacifica TYGS 22148
Xuhuaishuia manganoxidans TYGS 16842
Yoonia maricola TYGS 6183
Loktanella acticola KY817315
Yoonia sediminilitoris TYGS 22932
Yoonia maritima TYGS 22874
Yoonia litorea TYGS 4967
Yoonia vestfoldensis TYGS 1321
Flavimaricola marinus TYGS 11274
Loktanella agnita AY682198
Pseudoroseicyclus aestuarii TYGS 11405
Rubellimicrobium thermophilum TYGS 2680
Rubellimicrobium mesophilum TYGS 2682
Rubellimicrobium aerolatum EU338486
Rubellimicrobium roseum GU109478
Salinovum rubellum GQ359327
Oceanicola granulosus TYGS 2667
Roseisalinus antarcticus TYGS 22586
Wenxinia marina TYGS 1187
Wenxinia saemankumensis TYGS 6197
Oceaniglobus indicus TYGS 11288
Kandeliimicrobium roseum KT886061
Limimaricola pyoseonensis TYGS 2475
Limimaricola aestuariicola KJ855316

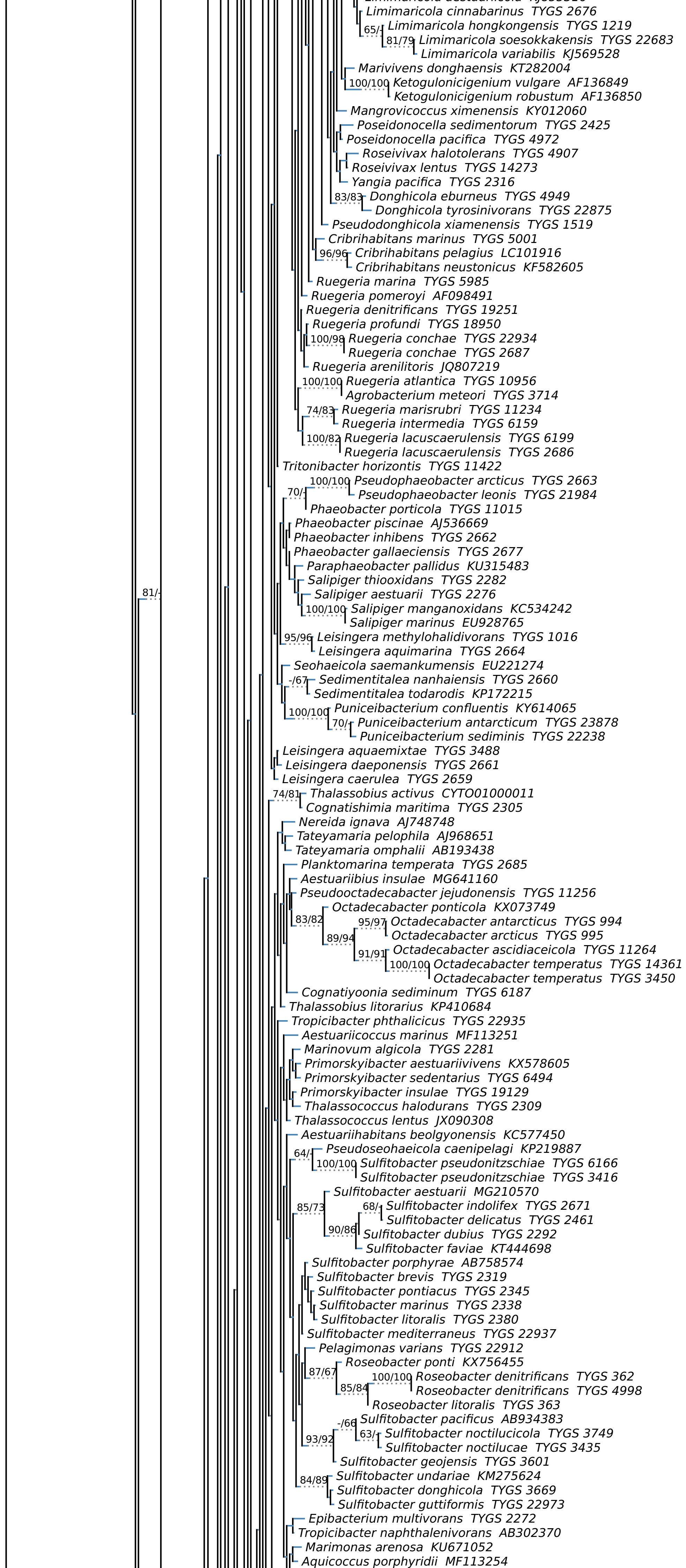

Limimaricola cinnabarinus TYGS 2676
65/-
Limimaricola hongkongensis TYGS 1219
81/79
Limimaricola soesokkakensis TYGS 22683
Limimaricola variabilis KJ569528
Marivivens donghaensis KT282004
100/100
Ketogulonicigenium vulgare AF136849
Ketogulonicigenium robustum AF136850
Mangrovicoccus ximenensis KY012060
Poseidonocella sedimentorum TYGS 2425
Poseidonocella pacifica TYGS 4972
Roseivivax halotolerans TYGS 4907
Roseivivax lentus TYGS 14273
Yangia pacifica TYGS 2316
83/83
Donghicola eburneus TYGS 4949
Donghicola tyrosinivorans TYGS 22875
Pseudodonghicola xiamenensis TYGS 1519
Cribrihabitans marinus TYGS 5001
96/96
Cribrihabitans pelagius LC101916
Cribrihabitans neustonicus KF582605
Ruegeria marina TYGS 5985
Ruegeria pomeroyi AF098491
Ruegeria denitrificans TYGS 19251
Ruegeria profundi TYGS 18950
100/98
Ruegeria conchae TYGS 22934
Ruegeria conchae TYGS 2687
Ruegeria arenilitoris JQ807219
100/100
Ruegeria atlantica TYGS 10956
Agrobacterium meteori TYGS 3714
74/83
Ruegeria marisrubri TYGS 11234
Ruegeria intermedia TYGS 6159
100/82
Ruegeria lacuscaerulensis TYGS 6199
Ruegeria lacuscaerulensis TYGS 2686
Tritonibacter horizontis TYGS 11422
100/100
Pseudophaeobacter arcticus TYGS 2663
70/-
Pseudophaeobacter leonis TYGS 21984
Phaeobacter porticola TYGS 11015
Phaeobacter piscinae AJ536669
Phaeobacter inhibens TYGS 2662
Phaeobacter gallaeciensis TYGS 2677
81/-
Paraphaeobacter pallidus KU315483
Salipiger thiooxidans TYGS 2282
Salipiger aestuarii TYGS 2276
100/100
Salipiger manganoxidans KC534242
Salipiger marinus EU928765
95/96
Leisingera methylohalidivorans TYGS 1016
Leisingera aquimarina TYGS 2664
Seohaeicola saemankumensis EU221274
-/67
Sedimentitalea nanhaiensis TYGS 2660
Sedimentitalea todarodis KP172215
100/100
Puniceibacterium confluentis KY614065
70/-
Puniceibacterium antarcticum TYGS 23878
Puniceibacterium sediminis TYGS 22238
Leisingera aquaemixtae TYGS 3488
Leisingera daeponensis TYGS 2661
Leisingera caerulea TYGS 2659
74/81
Thalassobius activus CYTO01000011
Cognatishimia maritima TYGS 2305
Nereida ignava AJ748748
Tateyamaria pelophila AJ968651
Tateyamaria omphalii AB193438
Planktomarina temperata TYGS 2685
Aestuariibius insulae MG641160
Pseudooctadecabacter jejudonensis TYGS 11256
83/82
Octadecabacter ponticola KX073749
95/97
Octadecabacter antarcticus TYGS 994
89/94
Octadecabacter arcticus TYGS 995
91/91
Octadecabacter ascidiaceicola TYGS 11264
100/100
Octadecabacter temperatus TYGS 14361
Octadecabacter temperatus TYGS 3450
Cognatiyoonia sediminum TYGS 6187
Thalassobius litorarius KP410684
Tropicibacter phthalicicus TYGS 22935
Aestuariicoccus marinus MF113251
Marinovum algicola TYGS 2281
Primorskyibacter aestuariivivens KX578605
Primorskyibacter sedentarius TYGS 6494
Primorskyibacter insulae TYGS 19129
Thalassococcus halodurans TYGS 2309
Thalassococcus lentus JX090308
Aestuariihabitans beolgyonensis KC577450
64/-
Pseudoseohaeicola caenipelagi KP219887
100/100
Sulfitobacter pseudonitzschiae TYGS 6166
Sulfitobacter pseudonitzschiae TYGS 3416
Sulfitobacter aestuarii MG210570
85/73
68/-
Sulfitobacter indolifex TYGS 2671
Sulfitobacter delicatus TYGS 2461
90/86
Sulfitobacter dubius TYGS 2292
Sulfitobacter faviae KT444698
Sulfitobacter porphyrae AB758574
Sulfitobacter brevis TYGS 2319
Sulfitobacter pontiacus TYGS 2345
Sulfitobacter marinus TYGS 2338
Sulfitobacter litoralis TYGS 2380
Sulfitobacter mediterraneus TYGS 22937
Pelagimonas varians TYGS 22912
Roseobacter ponti KX756455
87/67
100/100
Roseobacter denitrificans TYGS 362
85/84
Roseobacter denitrificans TYGS 4998
Roseobacter litoralis TYGS 363
-/66
Sulfitobacter pacificus AB934383
63/-
Sulfitobacter noctilucicola TYGS 3749
93/92
Sulfitobacter noctilucae TYGS 3435
Sulfitobacter geojensis TYGS 3601
84/89
Sulfitobacter undariae KM275624
Sulfitobacter donghicola TYGS 3669
Sulfitobacter guttiformis TYGS 22973
Epibacterium multivorans TYGS 2272
Tropicibacter naphthalenivorans AB302370
Marimonas arenosa KU671052
Aquicoccus porphyridii MF113254

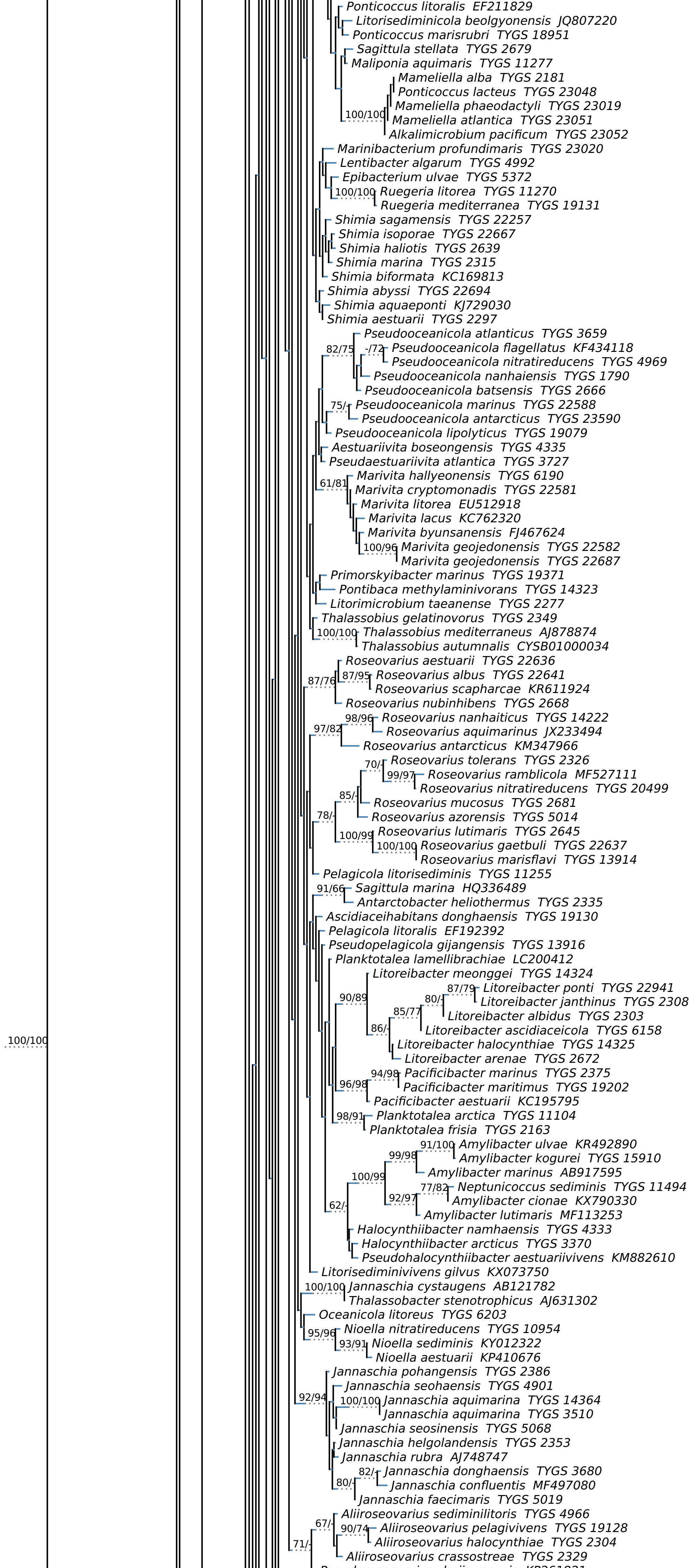

Ponticoccus litoralis EF211829
Litorisediminicola beolgyonensis JQ807220
Ponticoccus marisrubri TYGS 18951
Sagittula stellata TYGS 2679
Maliponia aquimaris TYGS 11277
Mameliella alba TYGS 2181
Ponticoccus lacteus TYGS 23048
Mameliella phaeodactyli TYGS 23019
100/100
Mameliella atlantica TYGS 23051
Alkalimicrobium pacificum TYGS 23052
Marinibacterium profundimaris TYGS 23020
Lentibacter algarum TYGS 4992
Epibacterium ulvae TYGS 5372
100/100
Ruegeria litorea TYGS 11270
Ruegeria mediterranea TYGS 19131
Shimia sagamensis TYGS 22257
Shimia isoporae TYGS 22667
Shimia haliotis TYGS 2639
Shimia marina TYGS 2315
Shimia biformata KC169813
Shimia abyssi TYGS 22694
Shimia aquaeponti KJ729030
Shimia aestuarii TYGS 2297
Pseudooceanicola atlanticus TYGS 3659
82/75
-/72
Pseudooceanicola flagellatus KF434118
Pseudooceanicola nitratireducens TYGS 4969
Pseudooceanicola nanhaiensis TYGS 1790
Pseudooceanicola batsensis TYGS 2666
75/-
Pseudooceanicola marinus TYGS 22588
Pseudooceanicola antarcticus TYGS 23590
Pseudooceanicola lipolyticus TYGS 19079
Aestuariivita boseongensis TYGS 4335
Pseudaestuariivita atlantica TYGS 3727
Marivita hallyeonensis TYGS 6190
61/81
Marivita cryptomonadis TYGS 22581
Marivita litorea EU512918
Marivita lacus KC762320
Marivita byunsanensis FJ467624
100/96
Marivita geojedonensis TYGS 22582
Marivita geojedonensis TYGS 22687
Primorskyibacter marinus TYGS 19371
Pontibaca methylaminivorans TYGS 14323
Litorimicrobium taeanense TYGS 2277
Thalassobius gelatinovorus TYGS 2349
100/100
Thalassobius mediterraneus AJ878874
Thalassobius autumnalis CYSB01000034
Roseovarius aestuarii TYGS 22636
87/76
87/95
Roseovarius albus TYGS 22641
Roseovarius scapharcae KR611924
Roseovarius nubinhibens TYGS 2668
98/96
Roseovarius nanhaiticus TYGS 14222
97/82
Roseovarius aquimarinus JX233494
Roseovarius antarcticus KM347966
70/-
Roseovarius tolerans TYGS 2326
99/97
Roseovarius ramblicola MF527111
Roseovarius nitratireducens TYGS 20499
85/-
Roseovarius mucosus TYGS 2681
78/-
Roseovarius azorensis TYGS 5014
100/99
Roseovarius lutimaris TYGS 2645
100/100
Roseovarius gaetbuli TYGS 22637
Roseovarius marisflavi TYGS 13914
Pelagicola litorisediminis TYGS 11255
91/66
Sagittula marina HQ336489
Antarctobacter heliothermus TYGS 2335
Ascidiaceihabitans donghaensis TYGS 19130
Pelagicola litoralis EF192392
Pseudopelagicola gijangensis TYGS 13916
Planktotalea lamellibrachiae LC200412
Litoreibacter meonggei TYGS 14324
87/79
Litoreibacter ponti TYGS 22941
90/89
80/-
Litoreibacter janthinus TYGS 2308
85/77
Litoreibacter albidus TYGS 2303
86/-
Litoreibacter ascidiaceicola TYGS 6158
100/100
Litoreibacter halocynthiae TYGS 14325
Litoreibacter arenae TYGS 2672
94/98
Pacificibacter marinus TYGS 2375
96/98
Pacificibacter maritimus TYGS 19202
Pacificibacter aestuarii KC195795
98/91
Planktotalea arctica TYGS 11104
Planktotalea frisia TYGS 2163
91/100
Amylibacter ulvae KR492890
99/98
Amylibacter kogurei TYGS 15910
100/99
Amylibacter marinus AB917595
77/82
Neptunicoccus sediminis TYGS 11494
92/97
Amylibacter cionae KX790330
62/-
Amylibacter lutimaris MF113253
Halocynthiibacter namhaensis TYGS 4333
Halocynthiibacter arcticus TYGS 3370
Pseudohalocynthiibacter aestuariivivens KM882610
Litorisediminivivens gilvus KX073750
100/100
Jannaschia cystaugens AB121782
Thalassobacter stenotrophicus AJ631302
Oceanicola litoreus TYGS 6203
95/96
Nioella nitratireducens TYGS 10954
93/91
Nioella sediminis KY012322
Nioella aestuarii KP410676
Jannaschia pohangensis TYGS 2386
Jannaschia seohaensis TYGS 4901
92/94
100/100
Jannaschia aquimarina TYGS 14364
Jannaschia aquimarina TYGS 3510
Jannaschia seosinensis TYGS 5068
Jannaschia helgolandensis TYGS 2353
Jannaschia rubra AJ748747
82/-
Jannaschia donghaensis TYGS 3680
80/-
Jannaschia confluentis MF497080
Jannaschia faecimaris TYGS 5019
67/-
Aliiroseovarius sediminilitoris TYGS 4966
90/74
Aliiroseovarius pelagivivens TYGS 19128
71/-
Aliiroseovarius halocynthiae TYGS 2304
Aliiroseovarius crassostreae TYGS 2329

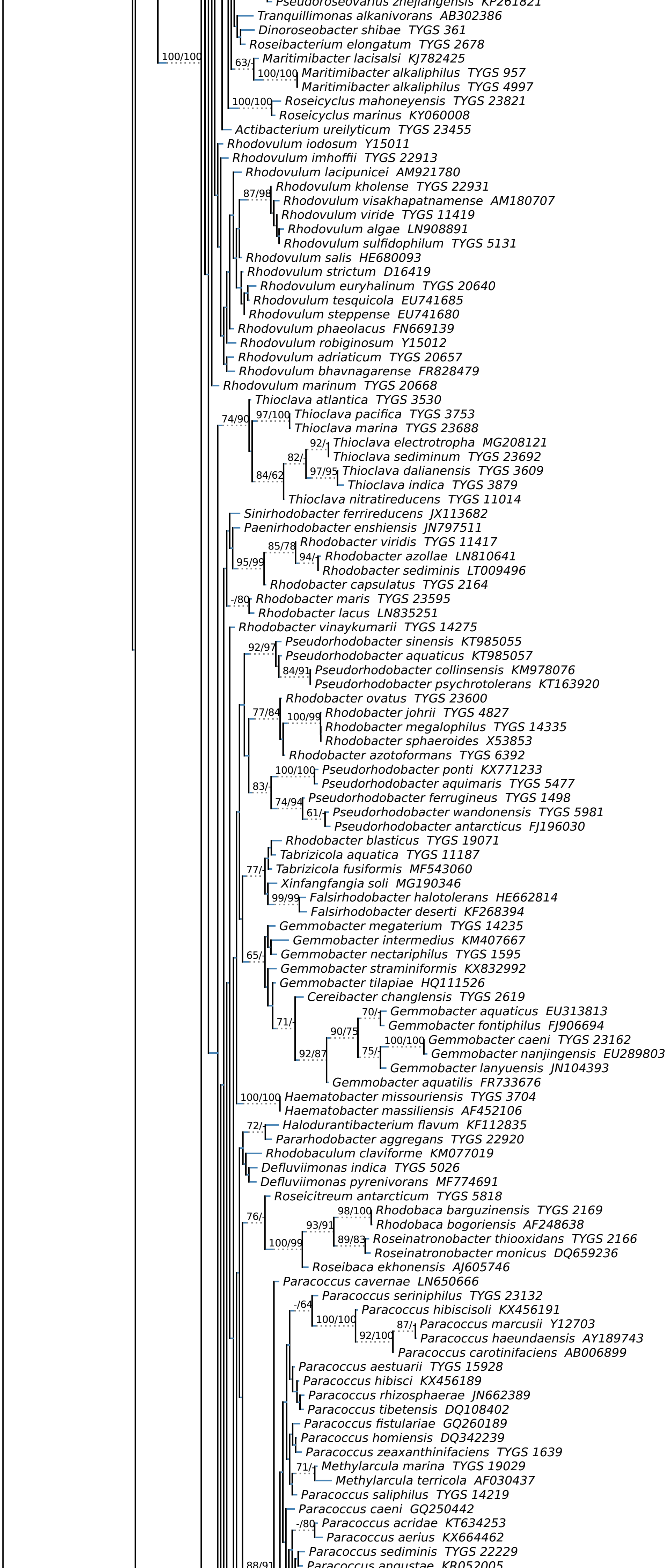
Pseudoroseovarius zhejiangensis KP261821
Tranquillimonas alkanivorans AB302386
Dinoroseobacter shibae TYGS 361
Roseibacterium elongatum TYGS 2678
100/100
63/-
Maritimibacter lacisalsi KJ782425
100/100
Maritimibacter alkaliphilus TYGS 957
Maritimibacter alkaliphilus TYGS 4997
100/100
Roseicyclus mahoneyensis TYGS 23821
Roseicyclus marinus KY060008
Actibacterium ureilyticum TYGS 23455
Rhodovulum iodosum Y15011
Rhodovulum imhoffii TYGS 22913
Rhodovulum lacipunicei AM921780
87/98
Rhodovulum kholense TYGS 22931
Rhodovulum visakhapatnamense AM180707
Rhodovulum viride TYGS 11419
Rhodovulum algae LN908891
Rhodovulum sulfidophilum TYGS 5131
Rhodovulum salis HE680093
Rhodovulum strictum D16419
Rhodovulum euryhalinum TYGS 20640
Rhodovulum tesquicola EU741685
Rhodovulum steppense EU741680
Rhodovulum phaeolacus FN669139
Rhodovulum robiginosum Y15012
Rhodovulum adriaticum TYGS 20657
Rhodovulum bhavnagarense FR828479
Rhodovulum marinum TYGS 20668
Thioclava atlantica TYGS 3530
74/90
97/100
Thioclava pacifica TYGS 3753
Thioclava marina TYGS 23688
92/-
Thioclava electrotropha MG208121
82/-
Thioclava sediminum TYGS 23692
97/95
Thioclava dalianensis TYGS 3609
84/62
Thioclava indica TYGS 3879
Thioclava nitratireducens TYGS 11014
Sinirhodobacter ferrireducens JX113682
Paenirhodobacter enshiensis JN797511
85/78
Rhodobacter viridis TYGS 11417
94/-
Rhodobacter azollae LN810641
95/99
Rhodobacter sediminis LT009496
Rhodobacter capsulatus TYGS 2164
-/80
Rhodobacter maris TYGS 23595
Rhodobacter lacus LN835251
Rhodobacter vinaykumarii TYGS 14275
92/97
Pseudorhodobacter sinensis KT985055
Pseudorhodobacter aquaticus KT985057
84/91
Pseudorhodobacter collinsensis KM978076
Pseudorhodobacter psychrotolerans KT163920
Rhodobacter ovatus TYGS 23600
77/84
Rhodobacter johrii TYGS 4827
100/99
Rhodobacter megalophilus TYGS 14335
Rhodobacter sphaeroides X53853
Rhodobacter azotoformans TYGS 6392
100/100
Pseudorhodobacter ponti KX771233
Pseudorhodobacter aquimaris TYGS 5477
83/-
74/94
Pseudorhodobacter ferrugineus TYGS 1498
61/-
Pseudorhodobacter wandonensis TYGS 5981
Pseudorhodobacter antarcticus FJ196030
Rhodobacter blasticus TYGS 19071
Tabrizicola aquatica TYGS 11187
77/-
Tabrizicola fusiformis MF543060
Xinfangfangia soli MG190346
99/99
Falsirhodobacter halotolerans HE662814
Falsirhodobacter deserti KF268394
Gemmobacter megaterium TYGS 14235
Gemmobacter intermedius KM407667
65/-
Gemmobacter nectariphilus TYGS 1595
Gemmobacter straminiformis KX832992
Gemmobacter tilapiae HQ111526
Cereibacter changlensis TYGS 2619
70/-
Gemmobacter aquaticus EU313813
71/-
Gemmobacter fontiphilus FJ906694
90/75
100/100
Gemmobacter caeni TYGS 23162
92/87
75/-
Gemmobacter nanjingensis EU289803
Gemmobacter lanyuensis JN104393
Gemmobacter aquatilis FR733676
100/100
Haematobacter missouriensis TYGS 3704
Haematobacter massiliensis AF452106
72/-
Halodurantibacterium flavum KF112835
Pararhodobacter aggregans TYGS 22920
Rhodobaculum claviforme KM077019
Defluviimonas indica TYGS 5026
Defluviimonas pyrenivorans MF774691
Roseicitreum antarcticum TYGS 5818
76/-
98/100
Rhodobaca barguzinensis TYGS 2169
93/91
Rhodobaca bogoriensis AF248638
89/83
Roseinatronobacter thiooxidans TYGS 2166
100/99
Roseinatronobacter monicus DQ659236
Roseibaca ekhonensis AJ605746
Paracoccus cavernae LN650666
-/64
Paracoccus seriniphilus TYGS 23132
100/100
Paracoccus hibiscisoli KX456191
87/-
Paracoccus marcusii Y12703
92/100
Paracoccus haeundaensis AY189743
Paracoccus carotinifaciens AB006899
Paracoccus aestuarii TYGS 15928
Paracoccus hibisci KX456189
Paracoccus rhizosphaerae JN662389
Paracoccus tibetensis DQ108402
Paracoccus fistulariae GQ260189
Paracoccus homiensis DQ342239
Paracoccus zeaxanthinifaciens TYGS 1639
71/-
Methylarcula marina TYGS 19029
Methylarcula terricola AF030437
Paracoccus saliphilus TYGS 14219
Paracoccus caeni GQ250442
-/80
Paracoccus acridae KT634253
Paracoccus aerius KX664462
Paracoccus sediminis TYGS 22229
88/91
Paracoccus angustae KR052005

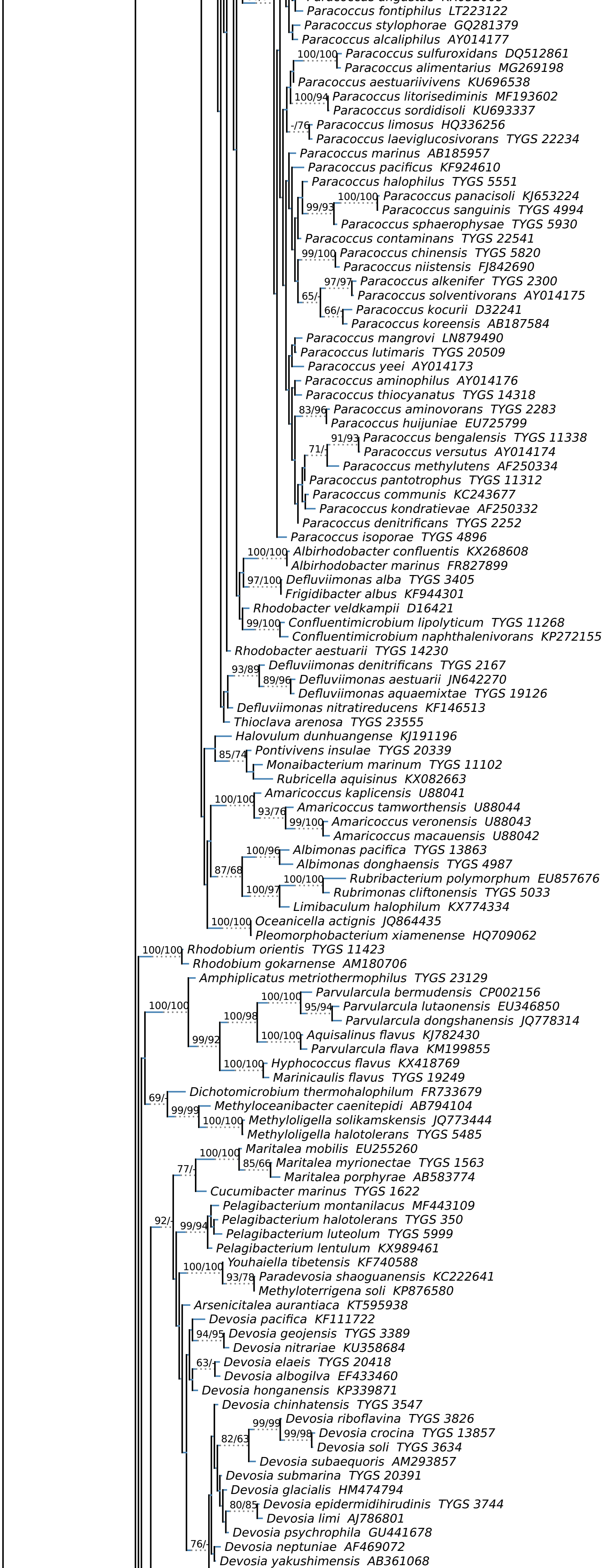

Paracoccus fontiphilus LT223122
Paracoccus stylophorae GQ281379
Paracoccus alcaliphilus AY014177
100/100
Paracoccus sulfuroxidans DQ512861
Paracoccus alimentarius MG269198
Paracoccus aestuariivivens KU696538
100/94
Paracoccus litorisediminis MF193602
Paracoccus sordidisoli KU693337
-/76
Paracoccus limosus HQ336256
Paracoccus laeviglucosivorans TYGS 22234
Paracoccus marinus AB185957
Paracoccus pacificus KF924610
Paracoccus halophilus TYGS 5551
100/100
Paracoccus panacisoli KJ653224
99/93
Paracoccus sanguinis TYGS 4994
Paracoccus sphaerophysae TYGS 5930
Paracoccus contaminans TYGS 22541
99/100
Paracoccus chinensis TYGS 5820
Paracoccus niistensis FJ842690
97/97
Paracoccus alkenifer TYGS 2300
65/-
Paracoccus solventivorans AY014175
66/-
Paracoccus kocurii D32241
Paracoccus koreensis AB187584
Paracoccus mangrovi LN879490
Paracoccus lutimaris TYGS 20509
Paracoccus yeei AY014173
Paracoccus aminophilus AY014176
Paracoccus thiocyanatus TYGS 14318
83/96
Paracoccus aminovorans TYGS 2283
Paracoccus huijuniae EU725799
91/93
Paracoccus bengalensis TYGS 11338
71/-
Paracoccus versutus AY014174
Paracoccus methylutens AF250334
Paracoccus pantotrophus TYGS 11312
Paracoccus communis KC243677
Paracoccus kondratievae AF250332
Paracoccus denitrificans TYGS 2252
Paracoccus isoporae TYGS 4896
100/100
Albirhodobacter confluentis KX268608
Albirhodobacter marinus FR827899
97/100
Defluviimonas alba TYGS 3405
Frigidibacter albus KF944301
Rhodobacter veldkampii D16421
99/100
Confluentimicrobium lipolyticum TYGS 11268
Confluentimicrobium naphthalenivorans KP272155
Rhodobacter aestuarii TYGS 14230
93/89
Defluviimonas denitrificans TYGS 2167
89/96
Defluviimonas aestuarii JN642270
Defluviimonas aquaemixtae TYGS 19126
Defluviimonas nitratireducens KF146513
Thioclava arenosa TYGS 23555
Halovulum dunhuangense KJ191196
85/74
Pontivivens insulae TYGS 20339
Monaibacterium marinum TYGS 11102
Rubricella aquisinus KX082663
100/100
Amaricoccus kaplicensis U88041
93/76
Amaricoccus tamworthensis U88044
99/100
Amaricoccus veronensis U88043
Amaricoccus macauensis U88042
100/96
Albimonas pacifica TYGS 13863
87/68
Albimonas donghaensis TYGS 4987
100/100
Rubribacterium polymorphum EU857676
100/97
Rubrimonas cliftonensis TYGS 5033
Limibaculum halophilum KX774334
100/100
Oceanicella actignis JQ864435
Pleomorphobacterium xiamenense HQ709062
100/100
Rhodobium orientis TYGS 11423
Rhodobium gokarnense AM180706
Amphiplicatus metriothermophilus TYGS 23129
100/100
100/100
Parvularcula bermudensis CP002156
95/94
Parvularcula lutaonensis EU346850
100/98
Parvularcula dongshanensis JQ778314
99/92
100/100
Aquisalinus flavus KJ782430
Parvularcula flava KM199855
100/100
Hyphococcus flavus KX418769
Marinicaulis flavus TYGS 19249
Dichotomicrobium thermohalophilum FR733679
69/-
99/99
Methyloceanibacter caenitepidi AB794104
100/100
Methyloligella solikamskensis JQ773444
Methyloligella halotolerans TYGS 5485
100/100
Maritalea mobilis EU255260
85/66
Maritalea myrionectae TYGS 1563
77/-
Maritalea porphyrae AB583774
Cucumibacter marinus TYGS 1622
Pelagibacterium montanilacus MF443109
92/-
Pelagibacterium halotolerans TYGS 350
99/94
Pelagibacterium luteolum TYGS 5999
Pelagibacterium lentulum KX989461
100/100
Youhaiella tibetensis KF740588
93/78
Paradevosia shaoguanensis KC222641
Methyloterrigena soli KP876580
Arsenicitalea aurantiaca KT595938
Devosia pacifica KF111722
94/95
Devosia geojensis TYGS 3389
Devosia nitrariae KU358684
63/-
Devosia elaeis TYGS 20418
Devosia albogilva EF433460
Devosia honganensis KP339871
Devosia chinhatensis TYGS 3547
99/99
Devosia riboflavina TYGS 3826
82/63
99/98
Devosia crocina TYGS 13857
Devosia soli TYGS 3634
Devosia subaequoris AM293857
Devosia submarina TYGS 20391
Devosia glacialis HM474794
80/85
Devosia epidermidihirudinis TYGS 3744
Devosia limi AJ786801
Devosia psychrophila GU441678
76/-
Devosia neptuniae AF469072
Devosia yakushimensis AB361068

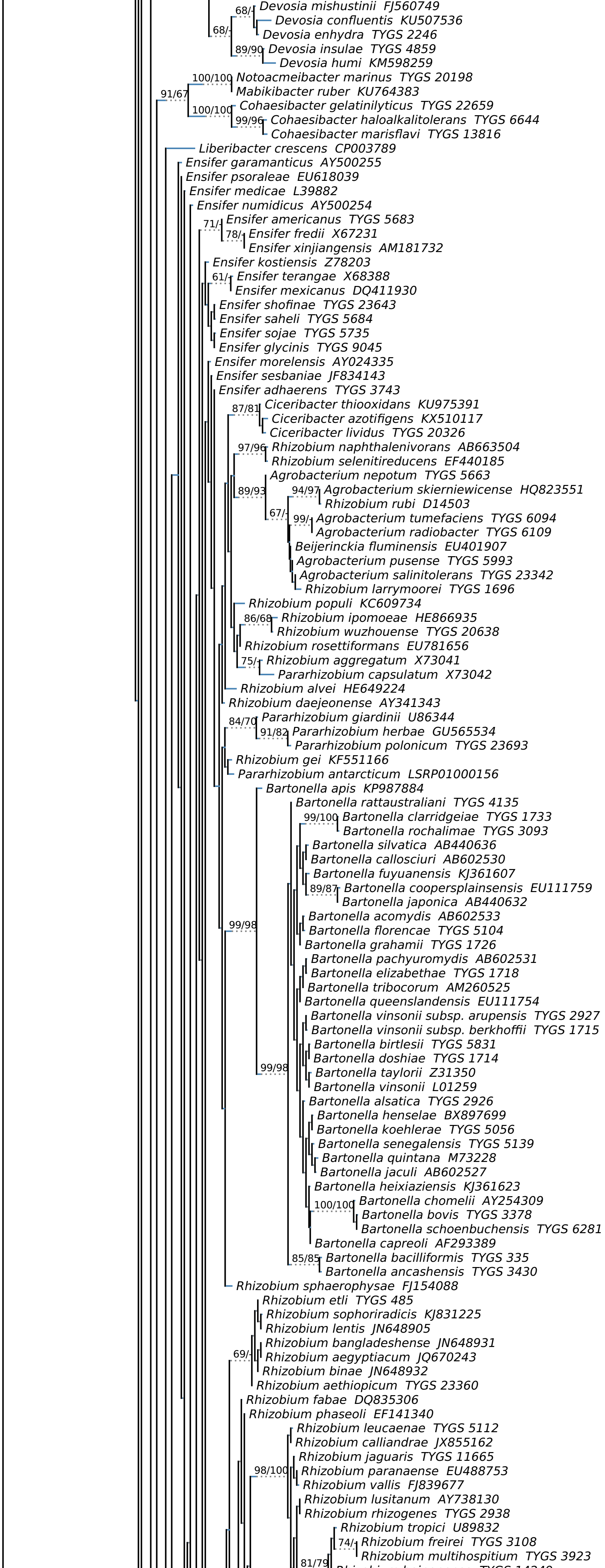
Devosia mishustinii FJ560749
Devosia confluentis KU507536
Devosia enhydra TYGS 2246
Devosia insulae TYGS 4859
Devosia humi KM598259
Notoacmeibacter marinus TYGS 20198
Mabikibacter ruber KU764383
Cohaesibacter gelatinilyticus TYGS 22659
Cohaesibacter haloalkalitolerans TYGS 6644
Cohaesibacter marisflavi TYGS 13816
Liberibacter crescens CP003789
Ensifer garamanticus AY500255
Ensifer psoraleae EU618039
Ensifer medicae L39882
Ensifer numidicus AY500254
Ensifer americanus TYGS 5683
Ensifer fredii X67231
Ensifer xinjiangensis AM181732
Ensifer kostiensis Z78203
Ensifer terangae X68388
Ensifer mexicanus DQ411930
Ensifer shofinae TYGS 23643
Ensifer saheli TYGS 5684
Ensifer sojae TYGS 5735
Ensifer glycinis TYGS 9045
Ensifer morelensis AY024335
Ensifer sesbaniae JF834143
Ensifer adhaerens TYGS 3743
Ciceribacter thiooxidans KU975391
Ciceribacter azotifigens KX510117
Ciceribacter lividus TYGS 20326
Rhizobium naphthalenivorans AB663504
Rhizobium selenitireducens EF440185
Agrobacterium nepotum TYGS 5663
Agrobacterium skierniewicense HQ823551
Rhizobium rubi D14503
Agrobacterium tumefaciens TYGS 6094
Agrobacterium radiobacter TYGS 6109
Beijerinckia fluminensis EU401907
Agrobacterium pusense TYGS 5993
Agrobacterium salinitolerans TYGS 23342
Rhizobium larrymoorei TYGS 1696
Rhizobium populi KC609734
Rhizobium ipomoeae HE866935
Rhizobium wuzhouense TYGS 20638
Rhizobium rosettiformans EU781656
Rhizobium aggregatum X73041
Pararhizobium capsulatum X73042
Rhizobium alvei HE649224
Rhizobium daejeonense AY341343
Pararhizobium giardinii U86344
Pararhizobium herbae GU565534
Pararhizobium polonicum TYGS 23693
Rhizobium gei KF551166
Pararhizobium antarcticum LSRP01000156
Bartonella apis KP987884
Bartonella rattaustraliani TYGS 4135
Bartonella clarridgeiae TYGS 1733
Bartonella rochalimae TYGS 3093
Bartonella silvatica AB440636
Bartonella callosciuri AB602530
Bartonella fuyuanensis KJ361607
Bartonella coopersplainsensis EU111759
Bartonella japonica AB440632
Bartonella acomydis AB602533
Bartonella florencae TYGS 5104
Bartonella grahamii TYGS 1726
Bartonella pachyuromydis AB602531
Bartonella elizabethae TYGS 1718
Bartonella tribocorum AM260525
Bartonella queenslandensis EU111754
Bartonella vinsonii subsp. arupensis TYGS 2927
Bartonella vinsonii subsp. berkhoffii TYGS 1715
Bartonella birtlesii TYGS 5831
Bartonella doshiae TYGS 1714
Bartonella taylorii Z31350
Bartonella vinsonii L01259
Bartonella alsatica TYGS 2926
Bartonella henselae BX897699
Bartonella koehlerae TYGS 5056
Bartonella senegalensis TYGS 5139
Bartonella quintana M73228
Bartonella jaculi AB602527
Bartonella heixiaziensis KJ361623
Bartonella chomelii AY254309
Bartonella bovis TYGS 3378
Bartonella schoenbuchensis TYGS 6281
Bartonella capreoli AF293389
Bartonella bacilliformis TYGS 335
Bartonella ancashensis TYGS 3430
Rhizobium sphaerophysae FJ154088
Rhizobium etli TYGS 485
Rhizobium sophoriradicis KJ831225
Rhizobium lentis JN648905
Rhizobium bangladeshense JN648931
Rhizobium aegyptiacum JQ670243
Rhizobium binae JN648932
Rhizobium aethiopicum TYGS 23360
Rhizobium fabae DQ835306
Rhizobium phaseoli EF141340
Rhizobium leucaenae TYGS 5112
Rhizobium calliandrae JX855162
Rhizobium jaguaris TYGS 11665
Rhizobium paranaense EU488753
Rhizobium vallis FJ839677
Rhizobium lusitanum AY738130
Rhizobium rhizogenes TYGS 2938
Rhizobium tropici U89832
Rhizobium freirei TYGS 3108
Rhizobium multihospitium TYGS 3923

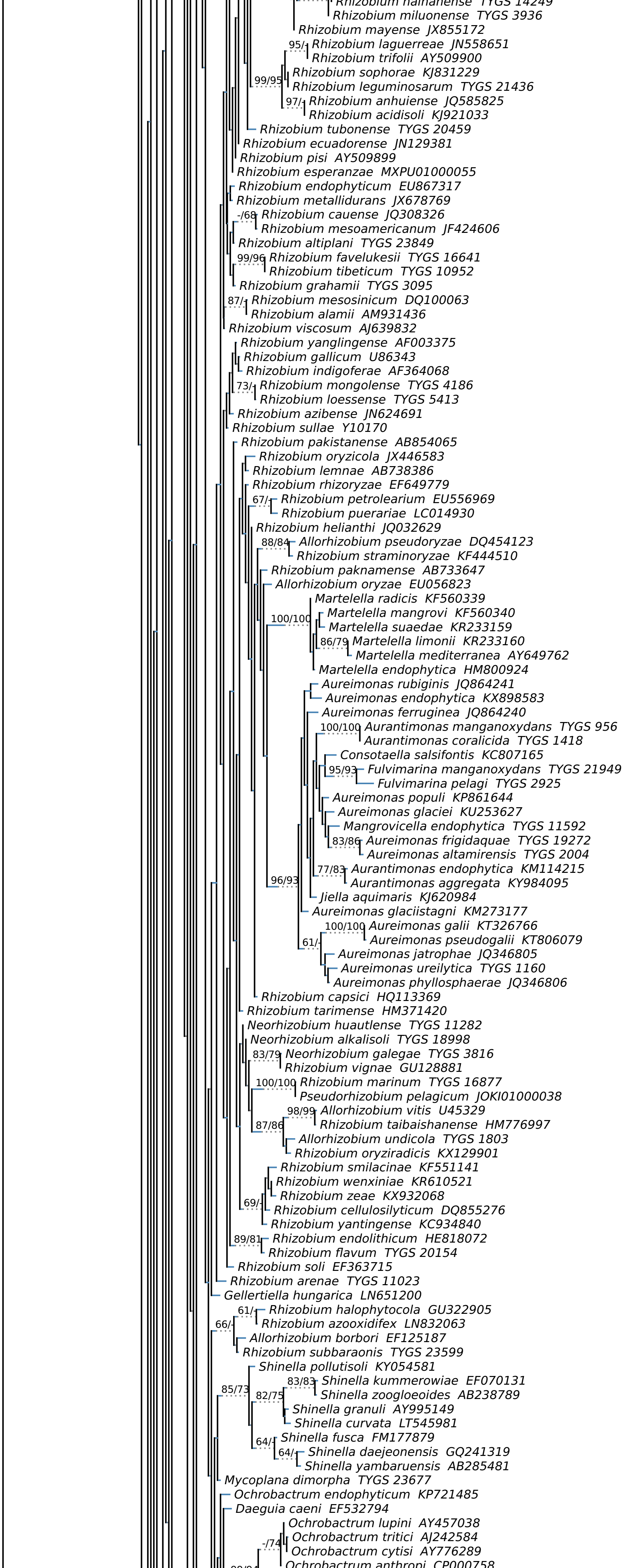
Rhizobium miluonense TYGS 3936
Rhizobium mayense JX855172
Rhizobium laguerreae JN558651
Rhizobium trifolii AY509900
Rhizobium sophorae KJ831229
Rhizobium leguminosarum TYGS 21436
Rhizobium anhuiense JQ585825
Rhizobium acidisoli KJ921033
Rhizobium tubonense TYGS 20459
Rhizobium ecuadorense JN129381
Rhizobium pisi AY509899
Rhizobium esperanzae MXPU01000055
Rhizobium endophyticum EU867317
Rhizobium metallidurans JX678769
Rhizobium cauense JQ308326
Rhizobium mesoamericanum JF424606
Rhizobium altiplani TYGS 23849
Rhizobium favelukesii TYGS 16641
Rhizobium tibeticum TYGS 10952
Rhizobium grahamii TYGS 3095
Rhizobium mesosinicum DQ100063
Rhizobium alamii AM931436
Rhizobium viscosum AJ639832
Rhizobium yanglingense AF003375
Rhizobium gallicum U86343
Rhizobium indigoferae AF364068
Rhizobium mongolense TYGS 4186
Rhizobium loessense TYGS 5413
Rhizobium azibense JN624691
Rhizobium sullae Y10170
Rhizobium pakistanense AB854065
Rhizobium oryzicola JX446583
Rhizobium lemnae AB738386
Rhizobium rhizoryzae EF649779
Rhizobium petrolearium EU556969
Rhizobium puerariae LC014930
Rhizobium helianthi JQ032629
Allorhizobium pseudoryzae DQ454123
Rhizobium straminoryzae KF444510
Rhizobium paknamense AB733647
Allorhizobium oryzae EU056823
Martelella radicis KF560339
Martelella mangrovi KF560340
Martelella suaedae KR233159
Martelella limonii KR233160
Martelella mediterranea AY649762
Martelella endophytica HM800924
Aureimonas rubiginis JQ864241
Aureimonas endophytica KX898583
Aureimonas ferruginea JQ864240
Aurantimonas manganoxydans TYGS 956
Aurantimonas coralicida TYGS 1418
Consotaella salsifontis KC807165
Fulvimarina manganoxydans TYGS 21949
Fulvimarina pelagi TYGS 2925
Aureimonas populi KP861644
Aureimonas glaciei KU253627
Mangrovicella endophytica TYGS 11592
Aureimonas frigidaquae TYGS 19272
Aureimonas altamirensis TYGS 2004
Aurantimonas endophytica KM114215
Aurantimonas aggregata KY984095
Jiella aquimaris KJ620984
Aureimonas glaciistagni KM273177
Aureimonas galii KT326766
Aureimonas pseudogalii KT806079
Aureimonas jatrophae JQ346805
Aureimonas ureilytica TYGS 1160
Aureimonas phyllosphaerae JQ346806
Rhizobium capsici HQ113369
Rhizobium tarimense HM371420
Neorhizobium huautlense TYGS 11282
Neorhizobium alkalisoli TYGS 18998
Neorhizobium galegae TYGS 3816
Rhizobium vignae GU128881
Rhizobium marinum TYGS 16877
Pseudorhizobium pelagicum JOKI01000038
Allorhizobium vitis U45329
Rhizobium taibaishanense HM776997
Allorhizobium undicola TYGS 1803
Rhizobium oryziradicis KX129901
Rhizobium smilacinae KF551141
Rhizobium wenxiniae KR610521
Rhizobium zeae KX932068
Rhizobium cellulosilyticum DQ855276
Rhizobium yantingense KC934840
Rhizobium endolithicum HE818072
Rhizobium flavum TYGS 20154
Rhizobium soli EF363715
Rhizobium arenae TYGS 11023
Gellertiella hungarica LN651200
Rhizobium halophytocola GU322905
Rhizobium azooxidifex LN832063
Allorhizobium borbori EF125187
Rhizobium subbaraonis TYGS 23599
Shinella pollutisoli KY054581
Shinella kummerowiae EF070131
Shinella zoogloeoides AB238789
Shinella granuli AY995149
Shinella curvata LT545981
Shinella fusca FM177879
Shinella daejeonensis GQ241319
Shinella yambaruensis AB285481
Mycoplana dimorpha TYGS 23677
Ochrobactrum endophyticum KP721485
Daeguia caeni EF532794
Ochrobactrum lupini AY457038
Ochrobactrum tritici AJ242584
Ochrobactrum cytisi AY776289
Ochrobactrum anthropi CP000758

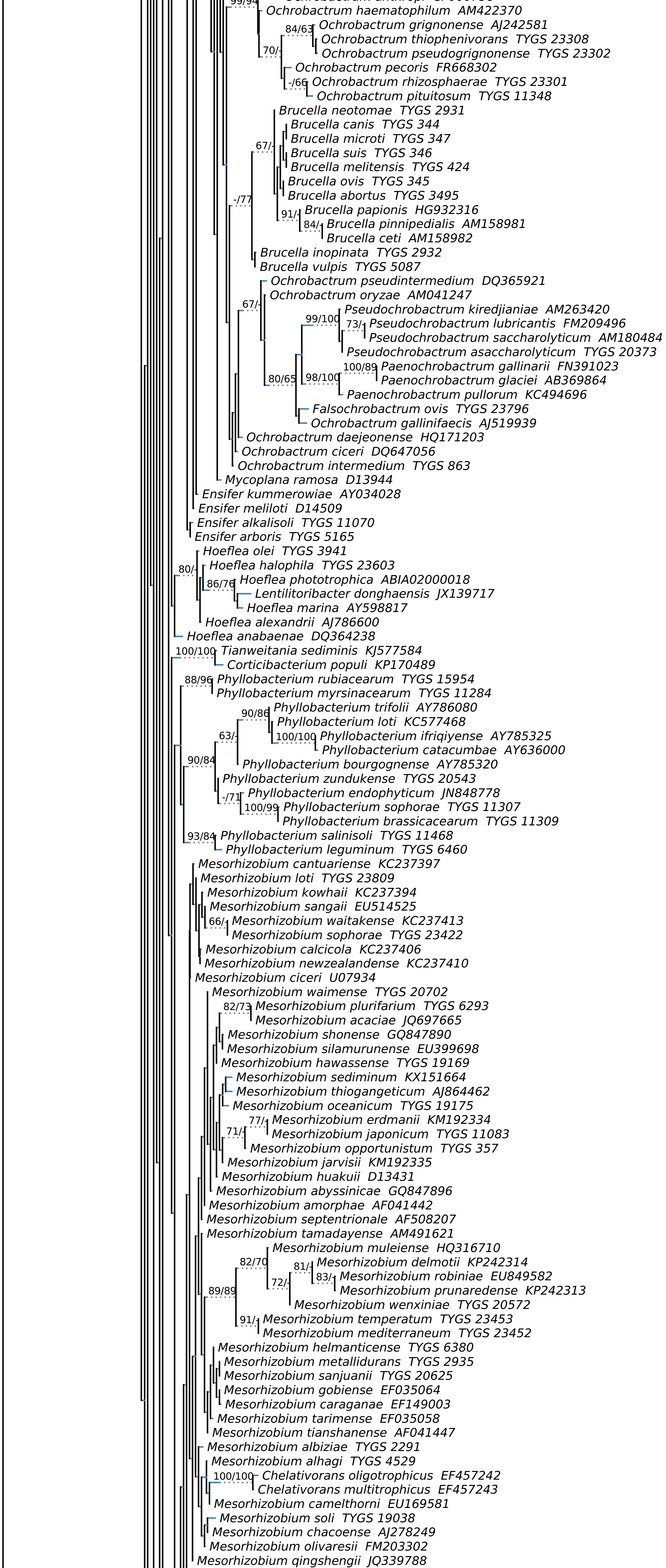
Ochrobactrum haematophilum AM422370
Ochrobactrum grignonense AJ242581
Ochrobactrum thiophenivorans TYGS 23308
Ochrobactrum pseudogrignonense TYGS 23302
Ochrobactrum pecoris FR668302
Ochrobactrum rhizosphaerae TYGS 23301
Ochrobactrum pituitosum TYGS 11348
Brucella neotomae TYGS 2931
Brucella canis TYGS 344
Brucella microti TYGS 347
Brucella suis TYGS 346
Brucella melitensis TYGS 424
Brucella ovis TYGS 345
Brucella abortus TYGS 3495
Brucella papionis HG932316
Brucella pinnipedialis AM158981
Brucella ceti AM158982
Brucella inopinata TYGS 2932
Brucella vulpis TYGS 5087
Ochrobactrum pseudintermedium DQ365921
Ochrobactrum oryzae AM041247
Pseudochrobactrum kiredjianiae AM263420
Pseudochrobactrum lubricantis FM209496
Pseudochrobactrum saccharolyticum AM180484
Pseudochrobactrum asaccharolyticum TYGS 20373
Paenochrobactrum gallinarii FN391023
Paenochrobactrum glaciei AB369864
Paenochrobactrum pullorum KC494696
Falsochrobactrum ovis TYGS 23796
Ochrobactrum gallinifaecis AJ519939
Ochrobactrum daejeonense HQ171203
Ochrobactrum ciceri DQ647056
Ochrobactrum intermedium TYGS 863
Mycoplana ramosa D13944
Ensifer kummerowiae AY034028
Ensifer meliloti D14509
Ensifer alkalisoli TYGS 11070
Ensifer arboris TYGS 5165
Hoeflea olei TYGS 3941
Hoeflea halophila TYGS 23603
Hoeflea phototrophica ABIA02000018
Lentilitoribacter donghaensis JX139717
Hoeflea marina AY598817
Hoeflea alexandrii AJ786600
Hoeflea anabaenae DQ364238
Tianweitania sediminis KJ577584
Corticibacterium populi KP170489
Phyllobacterium rubiacearum TYGS 15954
Phyllobacterium myrsinacearum TYGS 11284
Phyllobacterium trifolii AY786080
Phyllobacterium loti KC577468
Phyllobacterium ifriqiyense AY785325
Phyllobacterium catacumbae AY636000
Phyllobacterium bourgognense AY785320
Phyllobacterium zundukense TYGS 20543
Phyllobacterium endophyticum JN848778
Phyllobacterium sophorae TYGS 11307
Phyllobacterium brassicacearum TYGS 11309
Phyllobacterium salinisoli TYGS 11468
Phyllobacterium leguminum TYGS 6460
Mesorhizobium cantuariense KC237397
Mesorhizobium loti TYGS 23809
Mesorhizobium kowhaii KC237394
Mesorhizobium sangaii EU514525
Mesorhizobium waitakense KC237413
Mesorhizobium sophorae TYGS 23422
Mesorhizobium calcicola KC237406
Mesorhizobium newzealandense KC237410
Mesorhizobium ciceri U07934
Mesorhizobium waimense TYGS 20702
Mesorhizobium plurifarium TYGS 6293
Mesorhizobium acaciae JQ697665
Mesorhizobium shonense GQ847890
Mesorhizobium silamurunense EU399698
Mesorhizobium hawassense TYGS 19169
Mesorhizobium sediminum KX151664
Mesorhizobium thiogangeticum AJ864462
Mesorhizobium oceanicum TYGS 19175
Mesorhizobium erdmanii KM192334
Mesorhizobium japonicum TYGS 11083
Mesorhizobium opportunistum TYGS 357
Mesorhizobium jarvisii KM192335
Mesorhizobium huakuii D13431
Mesorhizobium abyssinicae GQ847896
Mesorhizobium amorphae AF041442
Mesorhizobium septentrionale AF508207
Mesorhizobium tamadayense AM491621
Mesorhizobium muleiense HQ316710
Mesorhizobium delmotii KP242314
Mesorhizobium robiniae EU849582
Mesorhizobium prunaredense KP242313
Mesorhizobium wenxiniae TYGS 20572
Mesorhizobium temperatum TYGS 23453
Mesorhizobium mediterraneum TYGS 23452
Mesorhizobium helmanticense TYGS 6380
Mesorhizobium metallidurans TYGS 2935
Mesorhizobium sanjuanii TYGS 20625
Mesorhizobium gobiense EF035064
Mesorhizobium caraganae EF149003
Mesorhizobium tarimense EF035058
Mesorhizobium tianshanense AF041447
Mesorhizobium albiziae TYGS 2291
Mesorhizobium alhagi TYGS 4529
Chelativorans oligotrophicus EF457242
Chelativorans multitrophicus EF457243
Mesorhizobium camelthorni EU169581
Mesorhizobium soli TYGS 19038
Mesorhizobium chacoense AJ278249
Mesorhizobium olivaresii FM203302
Mesorhizobium qingshengii JQ339788

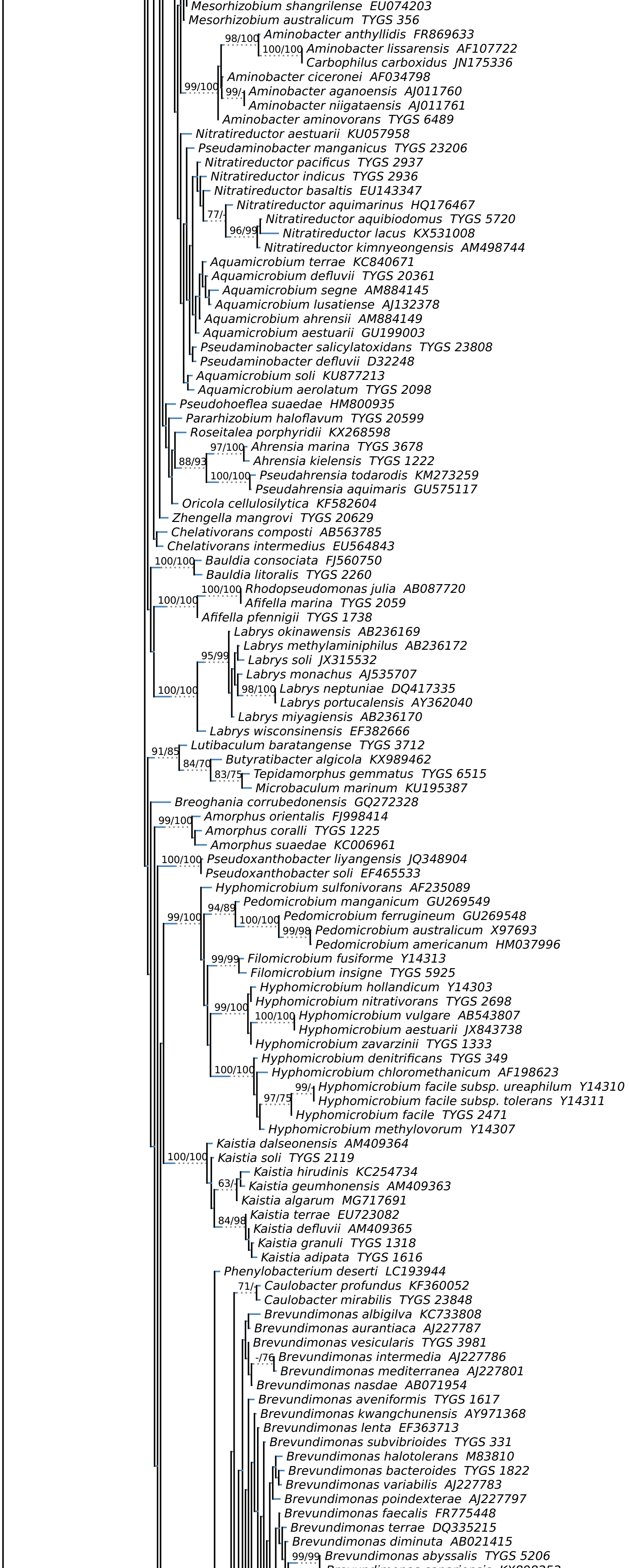
Mesorhizobium shangrilense EU074203
Mesorhizobium australicum TYGS 356
98/100
Aminobacter anthyllidis FR869633
100/100
Aminobacter lissarensis AF107722
Carbophilus carboxidus JN175336
Aminobacter ciceronei AF034798
99/100
99/-
Aminobacter aganoensis AJ011760
Aminobacter niigataensis AJ011761
Aminobacter aminovorans TYGS 6489
Nitratireductor aestuarii KU057958
Pseudaminobacter manganicus TYGS 23206
Nitratireductor pacificus TYGS 2937
Nitratireductor indicus TYGS 2936
Nitratireductor basaltis EU143347
Nitratireductor aquimarinus HQ176467
77/-
Nitratireductor aquibiodomus TYGS 5720
96/99
Nitratireductor lacus KX531008
Nitratireductor kimnyeongensis AM498744
Aquamicrobium terrae KC840671
Aquamicrobium defluvii TYGS 20361
Aquamicrobium segne AM884145
Aquamicrobium lusatiense AJ132378
Aquamicrobium ahrensii AM884149
Aquamicrobium aestuarii GU199003
Pseudaminobacter salicylatoxidans TYGS 23808
Pseudaminobacter defluvii D32248
Aquamicrobium soli KU877213
Aquamicrobium aerolatum TYGS 2098
Pseudohoeflea suaedae HM800935
Pararhizobium haloflavum TYGS 20599
Roseitalea porphyridii KX268598
97/100
Ahrensia marina TYGS 3678
88/93
Ahrensia kielensis TYGS 1222
100/100
Pseudahrensia todarodis KM273259
Pseudahrensia aquimaris GU575117
Oricola cellulosilytica KF582604
Zhengella mangrovi TYGS 20629
Chelativorans composti AB563785
Chelativorans intermedius EU564843
100/100
Bauldia consociata FJ560750
Bauldia litoralis TYGS 2260
100/100
Rhodopseudomonas julia AB087720
100/100
Afifella marina TYGS 2059
Afifella pfennigii TYGS 1738
Labrys okinawensis AB236169
Labrys methylaminiphilus AB236172
95/99
Labrys soli JX315532
Labrys monachus AJ535707
98/100
Labrys neptuniae DQ417335
100/100
Labrys portucalensis AY362040
Labrys miyagiensis AB236170
Labrys wisconsinensis EF382666
91/85
Lutibaculum baratangense TYGS 3712
84/70
Butyratibacter algicola KX989462
83/75
Tepidamorphus gemmatus TYGS 6515
Microbaculum marinum KU195387
Breoghania corrubedonensis GQ272328
99/100
Amorphus orientalis FJ998414
Amorphus coralli TYGS 1225
Amorphus suaedae KC006961
100/100
Pseudoxanthobacter liyangensis JQ348904
Pseudoxanthobacter soli EF465533
Hyphomicrobium sulfonivorans AF235089
94/89
Pedomicrobium manganicum GU269549
99/100
100/100
Pedomicrobium ferrugineum GU269548
99/98
Pedomicrobium australicum X97693
Pedomicrobium americanum HM037996
99/99
Filomicrobium fusiforme Y14313
Filomicrobium insigne TYGS 5925
Hyphomicrobium hollandicum Y14303
99/100
Hyphomicrobium nitrativorans TYGS 2698
100/100
Hyphomicrobium vulgare AB543807
Hyphomicrobium aestuarii JX843738
Hyphomicrobium zavarzinii TYGS 1333
100/100
Hyphomicrobium denitrificans TYGS 349
Hyphomicrobium chloromethanicum AF198623
99/-
Hyphomicrobium facile subsp. ureaphilum Y14310
97/75
Hyphomicrobium facile subsp. tolerans Y14311
Hyphomicrobium facile TYGS 2471
Hyphomicrobium methylovorum Y14307
Kaistia dalseonensis AM409364
100/100
Kaistia soli TYGS 2119
Kaistia hirudinis KC254734
63/-
Kaistia geumhonensis AM409363
Kaistia algarum MG717691
84/98
Kaistia terrae EU723082
Kaistia defluvii AM409365
Kaistia granuli TYGS 1318
Kaistia adipata TYGS 1616
Phenylobacterium deserti LC193944
71/-
Caulobacter profundus KF360052
Caulobacter mirabilis TYGS 23848
Brevundimonas albigilva KC733808
Brevundimonas aurantiaca AJ227787
Brevundimonas vesicularis TYGS 3981
-/76
Brevundimonas intermedia AJ227786
Brevundimonas mediterranea AJ227801
Brevundimonas nasdae AB071954
Brevundimonas aveniformis TYGS 1617
Brevundimonas kwangchunensis AY971368
Brevundimonas lenta EF363713
Brevundimonas subvibrioides TYGS 331
Brevundimonas halotolerans M83810
Brevundimonas bacteroides TYGS 1822
Brevundimonas variabilis AJ227783
Brevundimonas poindexterae AJ227797
Brevundimonas faecalis FR775448
Brevundimonas terrae DQ335215
Brevundimonas diminuta AB021415
99/99
Brevundimonas abyssalis TYGS 5206

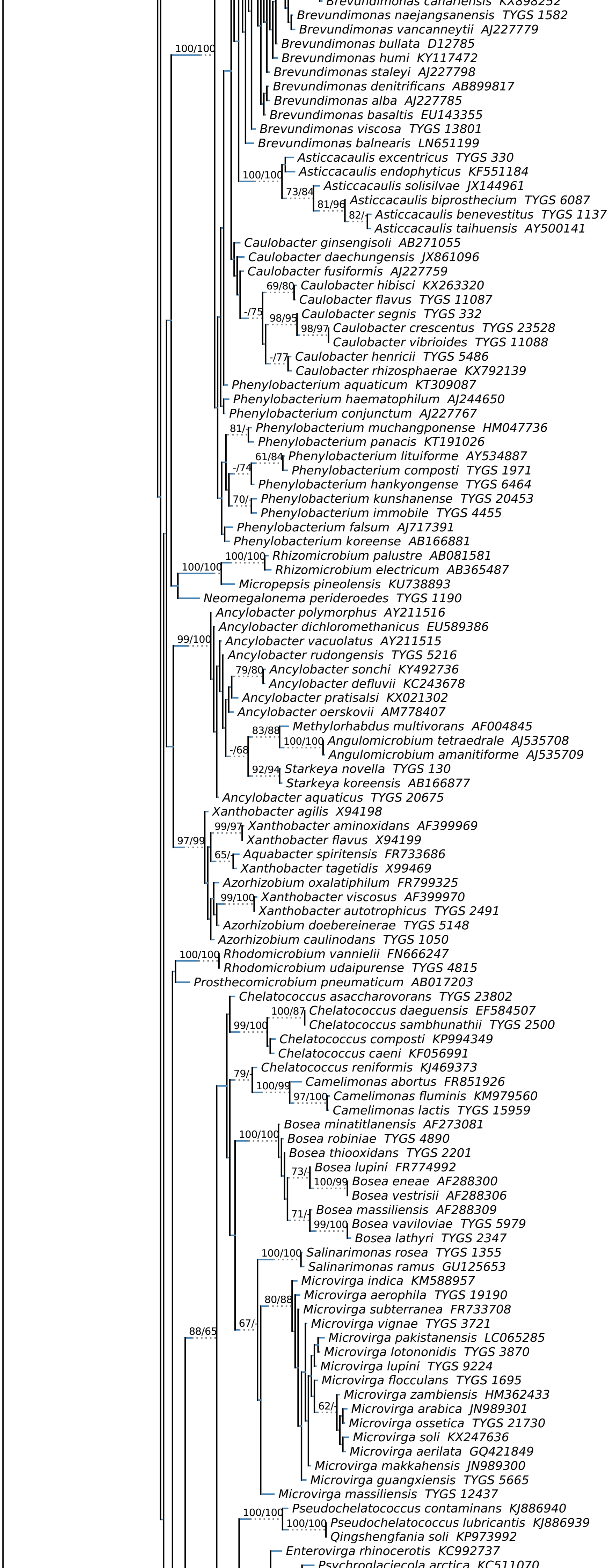

Brevundimonas canariensis KX898252
Brevundimonas naejangsanensis TYGS 1582
Brevundimonas vancanneytii AJ227779
100/100
Brevundimonas bullata D12785
Brevundimonas humi KY117472
Brevundimonas staleyi AJ227798
Brevundimonas denitrificans AB899817
Brevundimonas alba AJ227785
Brevundimonas basaltis EU143355
Brevundimonas viscosa TYGS 13801
Brevundimonas balnearis LN651199
Asticcacaulis excentricus TYGS 330
100/100
Asticcacaulis endophyticus KF551184
73/84
Asticcacaulis solisilvae JX144961
81/96
Asticcacaulis biprosthecium TYGS 6087
82/-
Asticcacaulis benevestitus TYGS 1137
Asticcacaulis taihuensis AY500141
Caulobacter ginsengisoli AB271055
Caulobacter daechungensis JX861096
Caulobacter fusiformis AJ227759
69/80
Caulobacter hibisci KX263320
Caulobacter flavus TYGS 11087
-/75
98/95
Caulobacter segnis TYGS 332
98/97
Caulobacter crescentus TYGS 23528
Caulobacter vibrioides TYGS 11088
-/77
Caulobacter henricii TYGS 5486
Caulobacter rhizosphaerae KX792139
Phenylobacterium aquaticum KT309087
Phenylobacterium haematophilum AJ244650
Phenylobacterium conjunctum AJ227767
81/-
Phenylobacterium muchangponense HM047736
Phenylobacterium panacis KT191026
61/84
Phenylobacterium lituiforme AY534887
-/74
Phenylobacterium composti TYGS 1971
Phenylobacterium hankyongense TYGS 6464
70/-
Phenylobacterium kunshanense TYGS 20453
Phenylobacterium immobile TYGS 4455
Phenylobacterium falsum AJ717391
Phenylobacterium koreense AB166881
100/100
Rhizomicrobium palustre AB081581
100/100
Rhizomicrobium electricum AB365487
Micropepsis pineolensis KU738893
Neomegalonema perideroedes TYGS 1190
Ancylobacter polymorphus AY211516
Ancylobacter dichloromethanicus EU589386
99/100
Ancylobacter vacuolatus AY211515
Ancylobacter rudongensis TYGS 5216
79/80
Ancylobacter sonchi KY492736
Ancylobacter defluvii KC243678
Ancylobacter pratisalsi KX021302
Ancylobacter oerskovii AM778407
83/88
Methylorhabdus multivorans AF004845
100/100
Angulomicrobium tetraedrale AJ535708
-/68
Angulomicrobium amanitiforme AJ535709
92/94
Starkeya novella TYGS 130
Starkeya koreensis AB166877
Ancylobacter aquaticus TYGS 20675
Xanthobacter agilis X94198
99/97
Xanthobacter aminoxidans AF399969
97/99
Xanthobacter flavus X94199
65/-
Aquabacter spiritensis FR733686
Xanthobacter tagetidis X99469
Azorhizobium oxalatiphilum FR799325
99/100
Xanthobacter viscosus AF399970
Xanthobacter autotrophicus TYGS 2491
Azorhizobium doebereinerae TYGS 5148
Azorhizobium caulinodans TYGS 1050
100/100
Rhodomicrobium vannielii FN666247
Rhodomicrobium udaipurense TYGS 4815
Prosthecomicrobium pneumaticum AB017203
Chelatococcus asaccharovorans TYGS 23802
100/87
Chelatococcus daeguensis EF584507
99/100
Chelatococcus sambhunathii TYGS 2500
Chelatococcus composti KP994349
Chelatococcus caeni KF056991
79/-
Chelatococcus reniformis KJ469373
100/99
Camelimonas abortus FR851926
97/100
Camelimonas fluminis KM979560
Camelimonas lactis TYGS 15959
Bosea minatitlanensis AF273081
100/100
Bosea robiniae TYGS 4890
Bosea thiooxidans TYGS 2201
73/-
Bosea lupini FR774992
100/99
Bosea eneae AF288300
Bosea vestrisii AF288306
71/-
Bosea massiliensis AF288309
99/100
Bosea vaviloviae TYGS 5979
Bosea lathyri TYGS 2347
100/100
Salinarimonas rosea TYGS 1355
Salinarimonas ramus GU125653
Microvirga indica KM588957
80/88
Microvirga aerophila TYGS 19190
Microvirga subterranea FR733708
67/-
Microvirga vignae TYGS 3721
88/65
Microvirga pakistanensis LC065285
Microvirga lotononidis TYGS 3870
Microvirga lupini TYGS 9224
Microvirga flocculans TYGS 1695
Microvirga zambiensis HM362433
62/-
Microvirga arabica JN989301
Microvirga ossetica TYGS 21730
Microvirga soli KX247636
Microvirga aerilata GQ421849
Microvirga makkahensis JN989300
Microvirga guangxiensis TYGS 5665
Microvirga massiliensis TYGS 12437
100/100
Pseudochelatococcus contaminans KJ886940
100/100
Pseudochelatococcus lubricantis KJ886939
Qingshengfania soli KP973992
Enterovirga rhinocerotis KC992737
Psychroglaciecola arctica KC511070

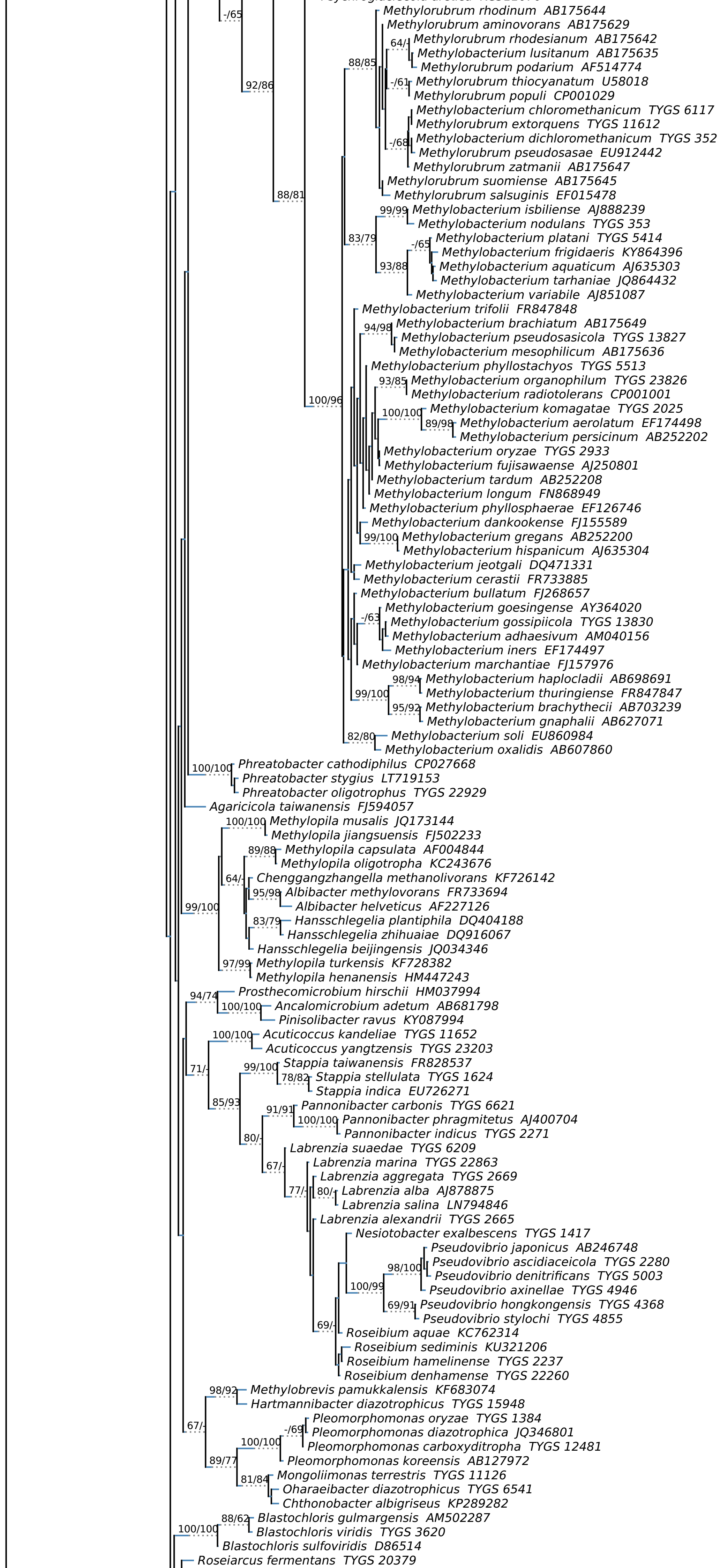
Methylorubrum rhodinum AB175644
Methylorubrum aminovorans AB175629
Methylorubrum rhodesianum AB175642
Methylobacterium lusitanum AB175635
Methylorubrum podarium AF514774
Methylorubrum thiocyanatum U58018
Methylorubrum populi CP001029
Methylobacterium chloromethanicum TYGS 6117
Methylorubrum extorquens TYGS 11612
Methylobacterium dichloromethanicum TYGS 352
Methylorubrum pseudosasae EU912442
Methylorubrum zatmanii AB175647
Methylorubrum suomiense AB175645
Methylorubrum salsuginis EF015478
Methylobacterium isbiliense AJ888239
Methylobacterium nodulans TYGS 353
Methylobacterium platani TYGS 5414
Methylobacterium frigidaeris KY864396
Methylobacterium aquaticum AJ635303
Methylobacterium tarhaniae JQ864432
Methylobacterium variabile AJ851087
Methylobacterium trifolii FR847848
Methylobacterium brachiatum AB175649
Methylobacterium pseudosasicola TYGS 13827
Methylobacterium mesophilicum AB175636
Methylobacterium phyllostachyos TYGS 5513
Methylobacterium organophilum TYGS 23826
Methylobacterium radiotolerans CP001001
Methylobacterium komagatae TYGS 2025
Methylobacterium aerolatum EF174498
Methylobacterium persicinum AB252202
Methylobacterium oryzae TYGS 2933
Methylobacterium fujisawaense AJ250801
Methylobacterium tardum AB252208
Methylobacterium longum FN868949
Methylobacterium phyllosphaerae EF126746
Methylobacterium dankookense FJ155589
Methylobacterium gregans AB252200
Methylobacterium hispanicum AJ635304
Methylobacterium jeotgali DQ471331
Methylobacterium cerastii FR733885
Methylobacterium bullatum FJ268657
Methylobacterium goesingense AY364020
Methylobacterium gossipiicola TYGS 13830
Methylobacterium adhaesivum AM040156
Methylobacterium iners EF174497
Methylobacterium marchantiae FJ157976
Methylobacterium haplocladii AB698691
Methylobacterium thuringiense FR847847
Methylobacterium brachythecii AB703239
Methylobacterium gnaphalii AB627071
Methylobacterium soli EU860984
Methylobacterium oxalidis AB607860
Phreatobacter cathodiphilus CP027668
Phreatobacter stygius LT719153
Phreatobacter oligotrophus TYGS 22929
Agaricicola taiwanensis FJ594057
Methylopila musalis JQ173144
Methylopila jiangsuensis FJ502233
Methylopila capsulata AF004844
Methylopila oligotropha KC243676
Chenggangzhangella methanolivorans KF726142
Albibacter methylovorans FR733694
Albibacter helveticus AF227126
Hansschlegelia plantiphila DQ404188
Hansschlegelia zhihuaiae DQ916067
Hansschlegelia beijingensis JQ034346
Methylopila turkensis KF728382
Methylopila henanensis HM447243
Prosthecomicrobium hirschii HM037994
Ancalomicrobium adetum AB681798
Pinisolibacter ravus KY087994
Acuticoccus kandeliae TYGS 11652
Acuticoccus yangtzensis TYGS 23203
Stappia taiwanensis FR828537
Stappia stellulata TYGS 1624
Stappia indica EU726271
Pannonibacter carbonis TYGS 6621
Pannonibacter phragmitetus AJ400704
Pannonibacter indicus TYGS 2271
Labrenzia suaedae TYGS 6209
Labrenzia marina TYGS 22863
Labrenzia aggregata TYGS 2669
Labrenzia alba AJ878875
Labrenzia salina LN794846
Labrenzia alexandrii TYGS 2665
Nesiotobacter exalbescens TYGS 1417
Pseudovibrio japonicus AB246748
Pseudovibrio ascidiaceicola TYGS 2280
Pseudovibrio denitrificans TYGS 5003
Pseudovibrio axinellae TYGS 4946
Pseudovibrio hongkongensis TYGS 4368
Pseudovibrio stylochi TYGS 4855
Roseibium aquae KC762314
Roseibium sediminis KU321206
Roseibium hamelinense TYGS 2237
Roseibium denhamense TYGS 22260
Methylobrevis pamukkalensis KF683074
Hartmannibacter diazotrophicus TYGS 15948
Pleomorphomonas oryzae TYGS 1384
Pleomorphomonas diazotrophica JQ346801
Pleomorphomonas carboxyditropha TYGS 12481
Pleomorphomonas koreensis AB127972
Mongoliimonas terrestris TYGS 11126
Oharaeibacter diazotrophicus TYGS 6541
Chthonobacter albigriseus KP289282
Blastochloris gulmargensis AM502287
Blastochloris viridis TYGS 3620
Blastochloris sulfoviridis D86514
Roseiarcus fermentans TYGS 20379

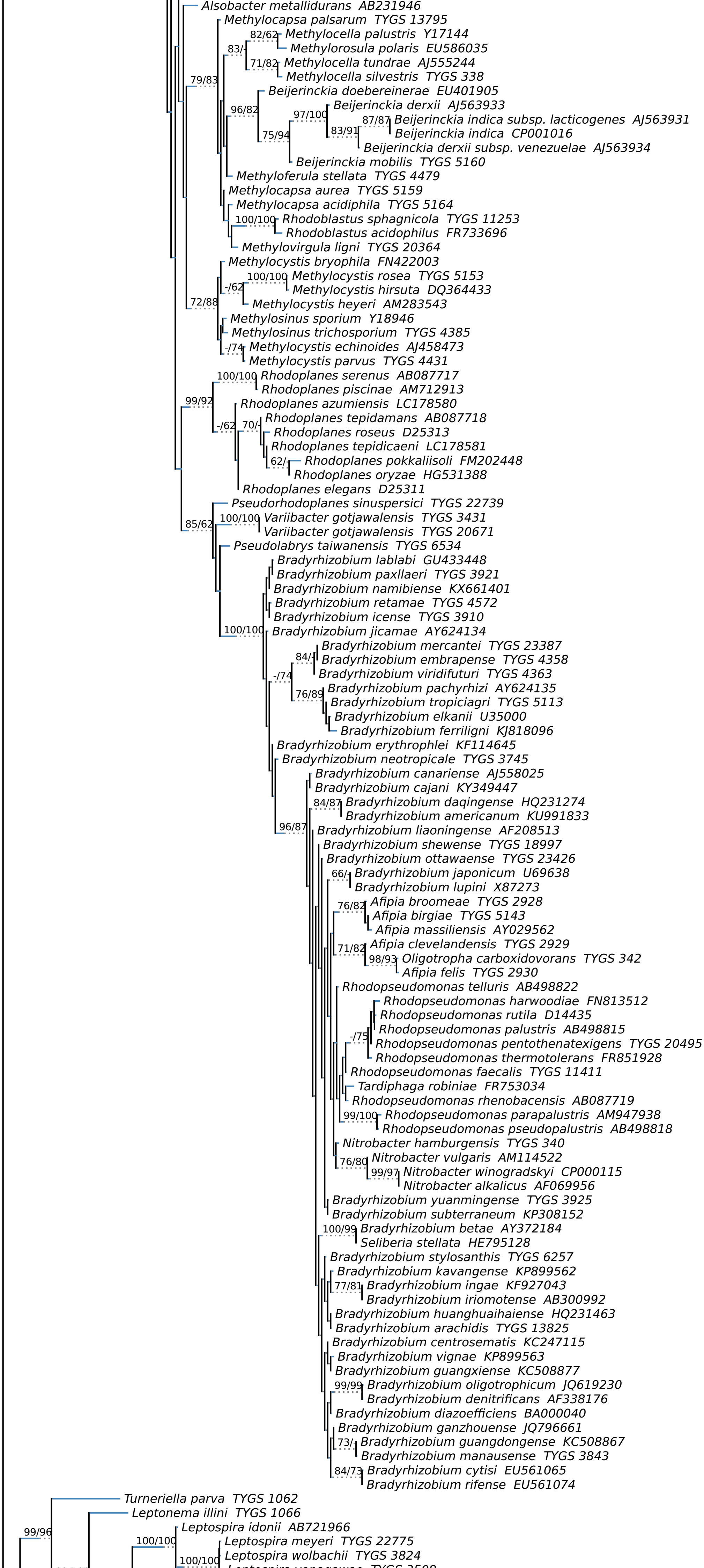
Alsobacter metallidurans AB231946
Methylocapsa palsarum TYGS 13795
82/62
Methylocella palustris Y17144
83/-
Methylorosula polaris EU586035
71/82
Methylocella tundrae AJ555244
79/83
Methylocella silvestris TYGS 338
Beijerinckia doebereinerae EU401905
Beijerinckia derxii AJ563933
97/100
96/82
87/87
Beijerinckia indica subsp. lacticogenes AJ563931
83/91
Beijerinckia indica CP001016
75/94
Beijerinckia derxii subsp. venezuelae AJ563934
Beijerinckia mobilis TYGS 5160
Methyloferula stellata TYGS 4479
Methylocapsa aurea TYGS 5159
Methylocapsa acidiphila TYGS 5164
100/100
Rhodoblastus sphagnicola TYGS 11253
Rhodoblastus acidophilus FR733696
Methylovirgula ligni TYGS 20364
Methylocystis bryophila FN422003
100/100
Methylocystis rosea TYGS 5153
-/62
Methylocystis hirsuta DQ364433
72/88
Methylocystis heyeri AM283543
Methylosinus sporium Y18946
Methylosinus trichosporium TYGS 4385
-/74
Methylocystis echinoides AJ458473
Methylocystis parvus TYGS 4431
100/100
Rhodoplanes serenus AB087717
Rhodoplanes piscinae AM712913
99/92
Rhodoplanes azumiensis LC178580
Rhodoplanes tepidamans AB087718
-/62
70/-
Rhodoplanes roseus D25313
Rhodoplanes tepidicaeni LC178581
62/-
Rhodoplanes pokkaliisoli FM202448
Rhodoplanes oryzae HG531388
Rhodoplanes elegans D25311
Pseudorhodoplanes sinuspersici TYGS 22739
100/100
Variibacter gotjawalensis TYGS 3431
85/62
Variibacter gotjawalensis TYGS 20671
Pseudolabrys taiwanensis TYGS 6534
Bradyrhizobium lablabi GU433448
Bradyrhizobium paxllaeri TYGS 3921
Bradyrhizobium namibiense KX661401
Bradyrhizobium retamae TYGS 4572
Bradyrhizobium icense TYGS 3910
100/100
Bradyrhizobium jicamae AY624134
Bradyrhizobium mercantei TYGS 23387
84/-
Bradyrhizobium embrapense TYGS 4358
-/74
Bradyrhizobium viridifuturi TYGS 4363
Bradyrhizobium pachyrhizi AY624135
76/89
Bradyrhizobium tropiciagri TYGS 5113
Bradyrhizobium elkanii U35000
Bradyrhizobium ferriligni KJ818096
Bradyrhizobium erythrophlei KF114645
Bradyrhizobium neotropicale TYGS 3745
Bradyrhizobium canariense AJ558025
Bradyrhizobium cajani KY349447
84/87
Bradyrhizobium daqingense HQ231274
Bradyrhizobium americanum KU991833
96/87
Bradyrhizobium liaoningense AF208513
Bradyrhizobium shewense TYGS 18997
Bradyrhizobium ottawaense TYGS 23426
66/-
Bradyrhizobium japonicum U69638
Bradyrhizobium lupini X87273
76/82
Afipia broomeae TYGS 2928
Afipia birgiae TYGS 5143
Afipia massiliensis AY029562
71/82
Afipia clevelandensis TYGS 2929
98/93
Oligotropha carboxidovorans TYGS 342
Afipia felis TYGS 2930
Rhodopseudomonas telluris AB498822
Rhodopseudomonas harwoodiae FN813512
Rhodopseudomonas rutila D14435
Rhodopseudomonas palustris AB498815
-/75
Rhodopseudomonas pentothenatexigens TYGS 20495
Rhodopseudomonas thermotolerans FR851928
Rhodopseudomonas faecalis TYGS 11411
Tardiphaga robiniae FR753034
Rhodopseudomonas rhenobacensis AB087719
99/100
Rhodopseudomonas parapalustris AM947938
Rhodopseudomonas pseudopalustris AB498818
Nitrobacter hamburgensis TYGS 340
76/80
Nitrobacter vulgaris AM114522
99/97
Nitrobacter winogradskyi CP000115
Nitrobacter alkalicus AF069956
Bradyrhizobium yuanmingense TYGS 3925
Bradyrhizobium subterraneum KP308152
100/99
Bradyrhizobium betae AY372184
Seliberia stellata HE795128
Bradyrhizobium stylosanthis TYGS 6257
Bradyrhizobium kavangense KP899562
77/81
Bradyrhizobium ingae KF927043
Bradyrhizobium iriomotense AB300992
Bradyrhizobium huanghuaihaiense HQ231463
Bradyrhizobium arachidis TYGS 13825
Bradyrhizobium centrosematis KC247115
Bradyrhizobium vignae KP899563
Bradyrhizobium guangxiense KC508877
99/99
Bradyrhizobium oligotrophicum JQ619230
Bradyrhizobium denitrificans AF338176
Bradyrhizobium diazoefficiens BA000040
Bradyrhizobium ganzhouense JQ796661
73/-
Bradyrhizobium guangdongense KC508867
Bradyrhizobium manausense TYGS 3843
84/73
Bradyrhizobium cytisi EU561065
Bradyrhizobium rifense EU561074
Turneriella parva TYGS 1062
99/96
Leptonema illini TYGS 1066
100/100
Leptospira idonii AB721966
Leptospira meyeri TYGS 22775
100/100
Leptospira wolbachii TYGS 3824

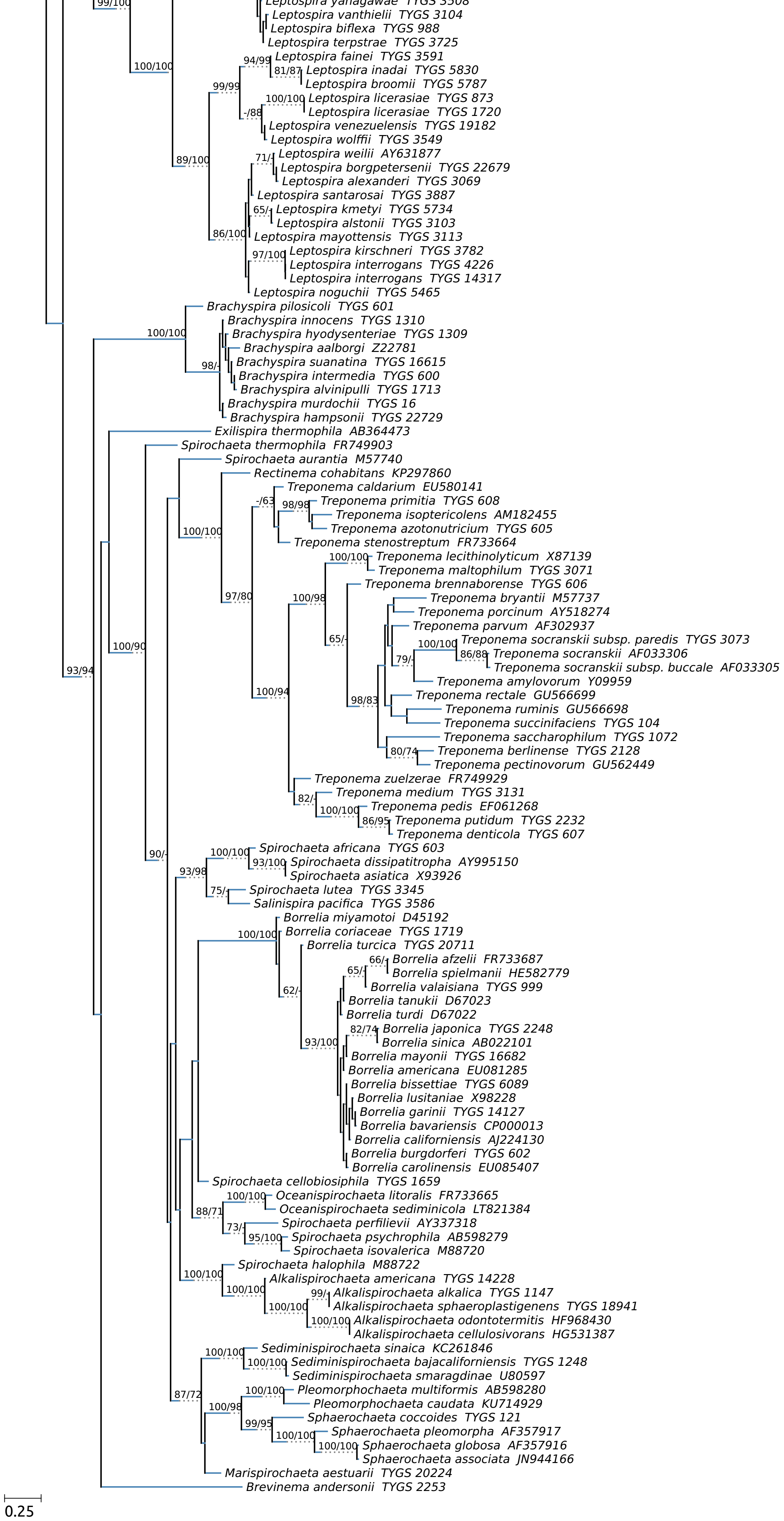

Leptospira vanthielii TYGS 3104
Leptospira biflexa TYGS 988
Leptospira terpstrae TYGS 3725
Leptospira fainei TYGS 3591
Leptospira inadai TYGS 5830
Leptospira broomii TYGS 5787
Leptospira licerasiae TYGS 873
Leptospira licerasiae TYGS 1720
Leptospira venezuelensis TYGS 19182
Leptospira wolffii TYGS 3549
Leptospira weilii AY631877
Leptospira borgpetersenii TYGS 22679
Leptospira alexanderi TYGS 3069
Leptospira santarosai TYGS 3887
Leptospira kmetyi TYGS 5734
Leptospira alstonii TYGS 3103
Leptospira mayottensis TYGS 3113
Leptospira kirschneri TYGS 3782
Leptospira interrogans TYGS 4226
Leptospira interrogans TYGS 14317
Leptospira noguchii TYGS 5465
Brachyspira pilosicoli TYGS 601
Brachyspira innocens TYGS 1310
Brachyspira hyodysenteriae TYGS 1309
Brachyspira aalborgi Z22781
Brachyspira suanatina TYGS 16615
Brachyspira intermedia TYGS 600
Brachyspira alvinipulli TYGS 1713
Brachyspira murdochii TYGS 16
Brachyspira hampsonii TYGS 22729
Exilispira thermophila AB364473
Spirochaeta thermophila FR749903
Spirochaeta aurantia M57740
Rectinema cohabitans KP297860
Treponema caldarium EU580141
Treponema primitia TYGS 608
Treponema isoptericolens AM182455
Treponema azotonutricium TYGS 605
Treponema stenostreptum FR733664
Treponema lecithinolyticum X87139
Treponema maltophilum TYGS 3071
Treponema brennaborense TYGS 606
Treponema bryantii M57737
Treponema porcinum AY518274
Treponema parvum AF302937
Treponema socranskii subsp. paredis TYGS 3073
Treponema socranskii AF033306
Treponema socranskii subsp. buccale AF033305
Treponema amylovorum Y09959
Treponema rectale GU566699
Treponema ruminis GU566698
Treponema succinifaciens TYGS 104
Treponema saccharophilum TYGS 1072
Treponema berlinense TYGS 2128
Treponema pectinovorum GU562449
Treponema zuelzerae FR749929
Treponema medium TYGS 3131
Treponema pedis EF061268
Treponema putidum TYGS 2232
Treponema denticola TYGS 607
Spirochaeta africana TYGS 603
Spirochaeta dissipatitropha AY995150
Spirochaeta asiatica X93926
Spirochaeta lutea TYGS 3345
Salinispira pacifica TYGS 3586
Borrelia miyamotoi D45192
Borrelia coriaceae TYGS 1719
Borrelia turcica TYGS 20711
Borrelia afzelii FR733687
Borrelia spielmanii HE582779
Borrelia valaisiana TYGS 999
Borrelia tanukii D67023
Borrelia turdi D67022
Borrelia japonica TYGS 2248
Borrelia sinica AB022101
Borrelia mayonii TYGS 16682
Borrelia americana EU081285
Borrelia bissettiae TYGS 6089
Borrelia lusitaniae X98228
Borrelia garinii TYGS 14127
Borrelia bavariensis CP000013
Borrelia californiensis AJ224130
Borrelia burgdorferi TYGS 602
Borrelia carolinensis EU085407
Spirochaeta cellobiosiphila TYGS 1659
Oceanispirochaeta litoralis FR733665
Oceanispirochaeta sediminicola LT821384
Spirochaeta perfilievii AY337318
Spirochaeta psychrophila AB598279
Spirochaeta isovalerica M88720
Spirochaeta halophila M88722
Alkalispirochaeta americana TYGS 14228
Alkalispirochaeta alkalica TYGS 1147
Alkalispirochaeta sphaeroplastigenens TYGS 18941
Alkalispirochaeta odontotermitis HF968430
Alkalispirochaeta cellulosivorans HG531387
Sediminispirochaeta sinaica KC261846
Sediminispirochaeta bajacaliforniensis TYGS 1248
Sediminispirochaeta smaragdinae U80597
Pleomorphochaeta multiformis AB598280
Pleomorphochaeta caudata KU714929
Sphaerochaeta coccoides TYGS 121
Sphaerochaeta pleomorpha AF357917
Sphaerochaeta globosa AF357916
Sphaerochaeta associata JN944166
Marispirochaeta aestuarii TYGS 20224
Brevinema andersonii TYGS 2253
0.25

Figure 3: Unconstrained comprehensive 16S rRNA gene ML tree (UCT) of *Alphaproteobacteria* inferred under the GTR+CAT model. The branches are scaled in terms of the expected number of substitutions per site. The numbers above the branches are support values when larger than 60% from ML (left) and MP (right) bootstrapping. Dotted parts of branches are filled in to allow proper placement of bootstrap values and are not part of the actual branch length. Numbers preceeded by the term 'TYGS' in labels refer to the genome IDs as found in Supplementary Table S1 (first sheet). Each tip label ends with the family of the respective taxon.

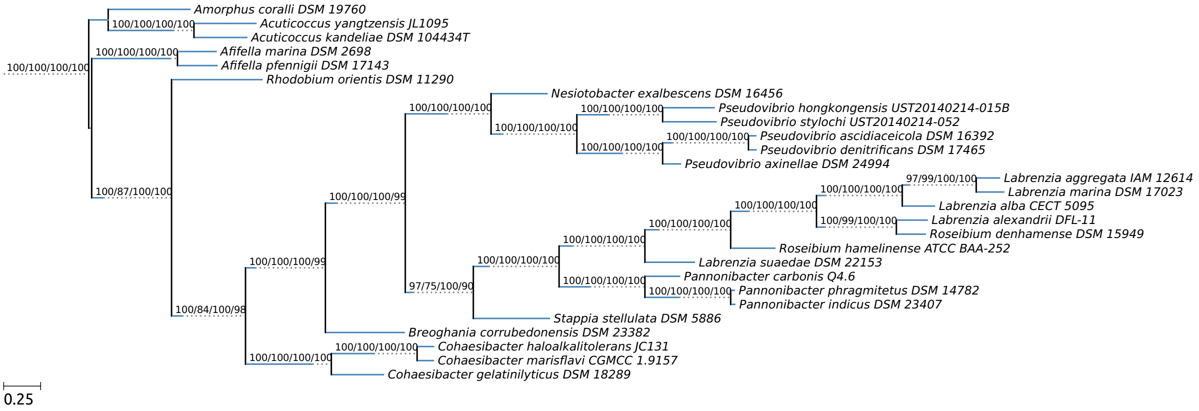

Figure 4: Phylogenetic tree inferred with RAxML from the *Labrenzia* supermatrix including single-copy core genes. Branches are scaled in terms of the expected number of changes per site. The first two numbers above branches (left to right) are partition bootstrap support values from (i) RAxML (ML) analysis and (ii) TNT (MP) analysis of the supermatrix that included single-copy genes that occurred in all of the genomes. The last two numbers above branches are partition bootstrap support values from (iii) RAxML (ML) analysis and (iv) TNT (MP) analysis of the supermatrix that included single-copy genes that occurred in at least four of the genomes.

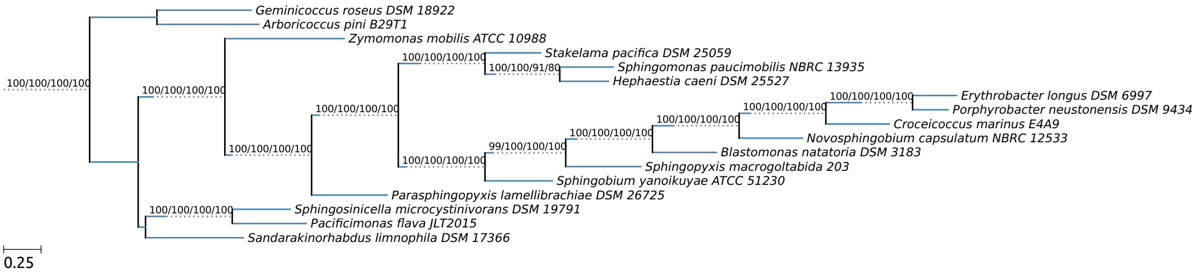

Figure 5: Phylogenetic ML tree inferred with RAxML from the *Sphingomonadales* supermatrix including single-copy core genes. Branches are scaled in terms of the expected number of changes per site. The first two numbers above branches (left to right) are partition bootstrap support values from (i) RAxML (ML) analysis and (ii) TNT (MP) analysis of the supermatrix that included single-copy genes that occurred in all of the genomes. The last two numbers above branches are partition bootstrap support values from (iii) RAxML (ML) analysis and (iv) TNT (MP) analysis of the supermatrix that included single-copy genes that occurred in at least four of the genomes.
